# Formal Markovnikov Hydroazolation of Alkenes via Reductive Activation of Alkylthianthrenium Salts

**DOI:** 10.1021/jacs.6c08262

**Published:** 2026-07-23

**Authors:** Adrian D. Matthews, John R. Lorenz, Zachary K. Wickens

**Affiliations:** Department of Chemistry, 5228 University of Wisconsin-Madison, Madison, Wisconsin 53706, United States

## Abstract

Herein, we introduce a new synthetic protocol for azole alkylation. Our strategy first engages azoles using alkenylthianthrenium electrophiles to provide 1,2-azolothianthrenium salts, then selectively excises the sulfonium moiety via single electron transfer (SET). Given that alkenylthianthrenium salts can be readily accessed from alkenes, the net azole addition-hydrogenolysis sequence unlocks a versatile approach to formal Markovnikov alkene hydroazolation. Moreover, the key radical intermediate formed after reduction can be engaged in reactions beyond hydrogen atom transfer (HAT), providing access to carboazolation products that would be otherwise prohibitively difficult to prepare. Mechanistic studies into the reduction of the alkylthianthrenium electrophile implicate an unusual silyl radical attack on DMF that generates a strong SET reductant in situ under otherwise exceedingly mild conditions. Overall, this work introduces new approaches to transform alkylsulfonium building blocks and immediately expands the range of *N*-alkyl azole molecules that can be readily prepared and studied.

## Graphical abstract

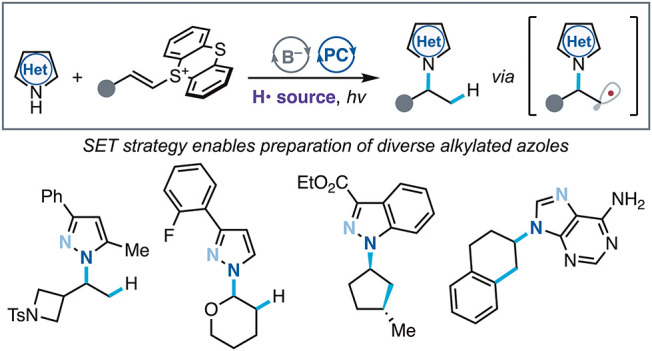

Azoles are important structural motifs with applications across fields ranging from agro- to medicinal chemistry (Figure 1A).^1^ The development of modular synthetic tactics to prepare diverse *N*-alkyl azoles, however, requires mechanistic manifolds that simultaneously accommodate the tremendous steric and electronic diversity of azoles as well as alkyl substituents.^2^ As a consequence, only a handful of methods have been identified that deliver structurally diverse *N*-alkyl azoles and, within each, conspicuous limitations remain. Classically, the preparation of *N*-alkyl azole compounds relies on substitution chemistry that is largely constrained to the installation of primary alkyl groups due to its sensitivity to steric hindrance.^3,4^ Furthermore, substitution reactions regularly result in unselective mixtures of *N*-regioisomers.^5^ Recently, approaches to azole alkylation which generate the C–N bond using carbon-centered radical intermediates have enabled the integration of alkyl groups from diverse sources, including benzylic C–H bonds, alkyl halides, NHP-esters, and carboxylic acids.^6^ While these represent valuable advances, examples of these processes are dominated by alkyl substituents that correspond to stabilized radical intermediates (e.g., tertiary and benzylic), reflecting their conserved radical mechanistic manifold.^7^

**Figure 1:**
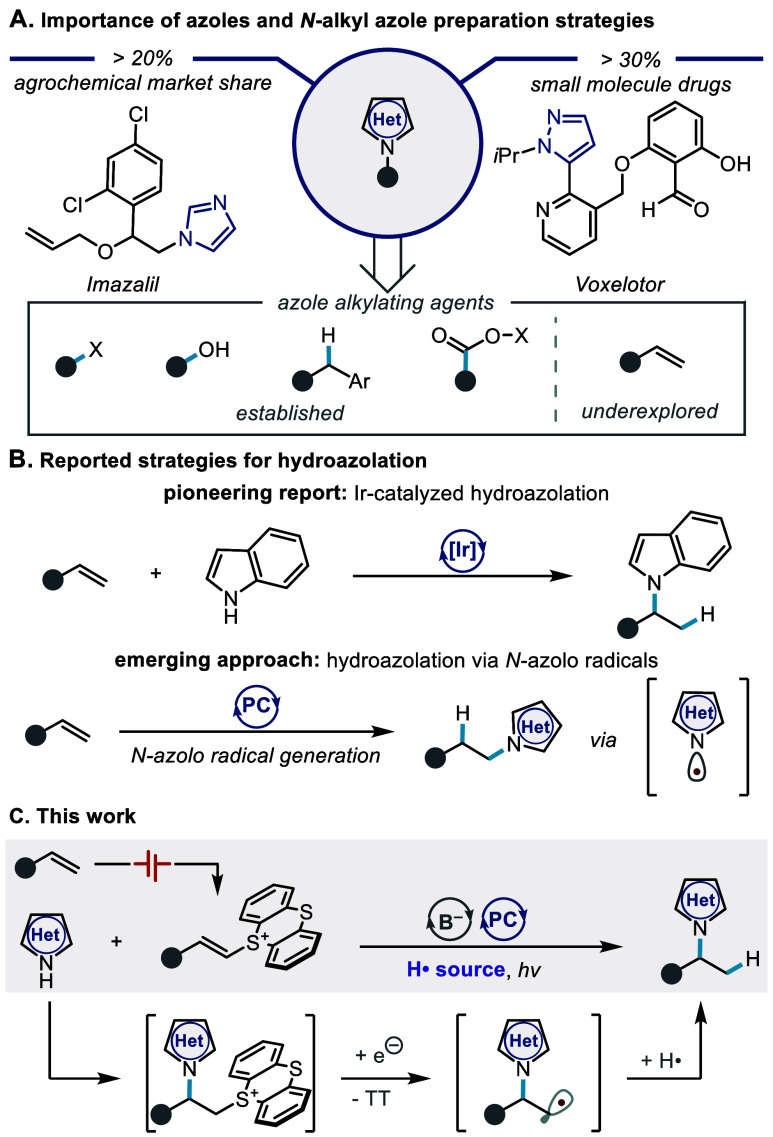
Overview. PC = photocatalyst; B^–^ = base; TT = thianthrene.

The hydroazolation of unactivated alkenes represents an appealing alternative route to *N*-alkyl azoles. However, in sharp contrast with the wide range of mechanistically diverse approaches to alkene hydroamination that have emerged over the past decade (e.g., additions of amines and their surrogates across alkenes), alkene hydroazolation reactions remain conspicuously underdeveloped (Figure 1B).^8^ The first example, an isolated report in 2014 from Hartwig, described an iridium-catalyzed Markovnikov addition of indoles across unactivated alkenes.^9^ This remained the only method until recent pioneering work from Zhang, Doyle, and Knowles introduced strategies to transform diverse azole nucleophiles into *N*-azolo radicals, unlocking a collection of anti-Markovnikov hydroazolation reactions.^10^ Very recently, Doyle discovered that their radical hydroazolation protocol can be shifted to favor Markovnikov products by a catalyst-induced change in mechanism.^11^ Overall, the introduction of hydroazolation reactions that proceed through distinct mechanistic manifolds remains poised for significant impact.

We questioned whether hydroazolation via alkene activation might offer a versatile complement to emerging azole activation strategies. Recently, we identified that the transformation of alkenes into alkenylthianthrenium electrophiles offers a platform to access β-functionalized *N*-alkyl azoles, exploiting 1,2-azolothianthrenium building blocks formed through conjugate addition.^12^ We recognized that, in principle, the reduction of the key 1,2-azolothianthrenium intermediate would offer a deceptively simple approach to net hydroazolation products, complementing both substitution reactivity and emerging radical hydroazolation methods. However, the reduction of hindered alkylthianthrenium species remains unexplored, and prior work has established that alkylthianthrenium salts are prone to competitive, base-induced elimination pathways.^13^ Herein, we illustrate that SET reduction of azolothianthrenium salts circumvents the elimination challenges that stymie polar reaction manifolds and provides a general, modular strategy for the net Markovnikov hydroazolation of alkenes (Figure 1C).

We selected 1,2-azolothianthrenium **1**—formed from 4-Ph-pyrazole, 1-butene, and **TT**—as a model substrate to study the key C–S reduction step (Scheme 1A). We began by evaluating a range of mild borohydrides in polar aprotic solvents,^14^ drawing on the conditions recently reported by Soós and Varga to be effective for primary alkyl thianthrenium displacement by hydrides.^15^ Unfortunately, the more hindered electrophile, **1**, underwent rapid decomposition, providing at most a modest yield of the desired product. While the specific distribution of products varied dramatically across the conditions, the dominant byproducts could be clearly traced to elimination pathways (e.g., alkenyl and allylic azoles).^16^ Use of protic solvents that are more conventional for hydride delivery with other electrophiles led to modestly improved yields, but elimination pathways remained a persistent problem even with this simple model substrate (see the Supporting Information (SI) for details).

**Scheme 1:**
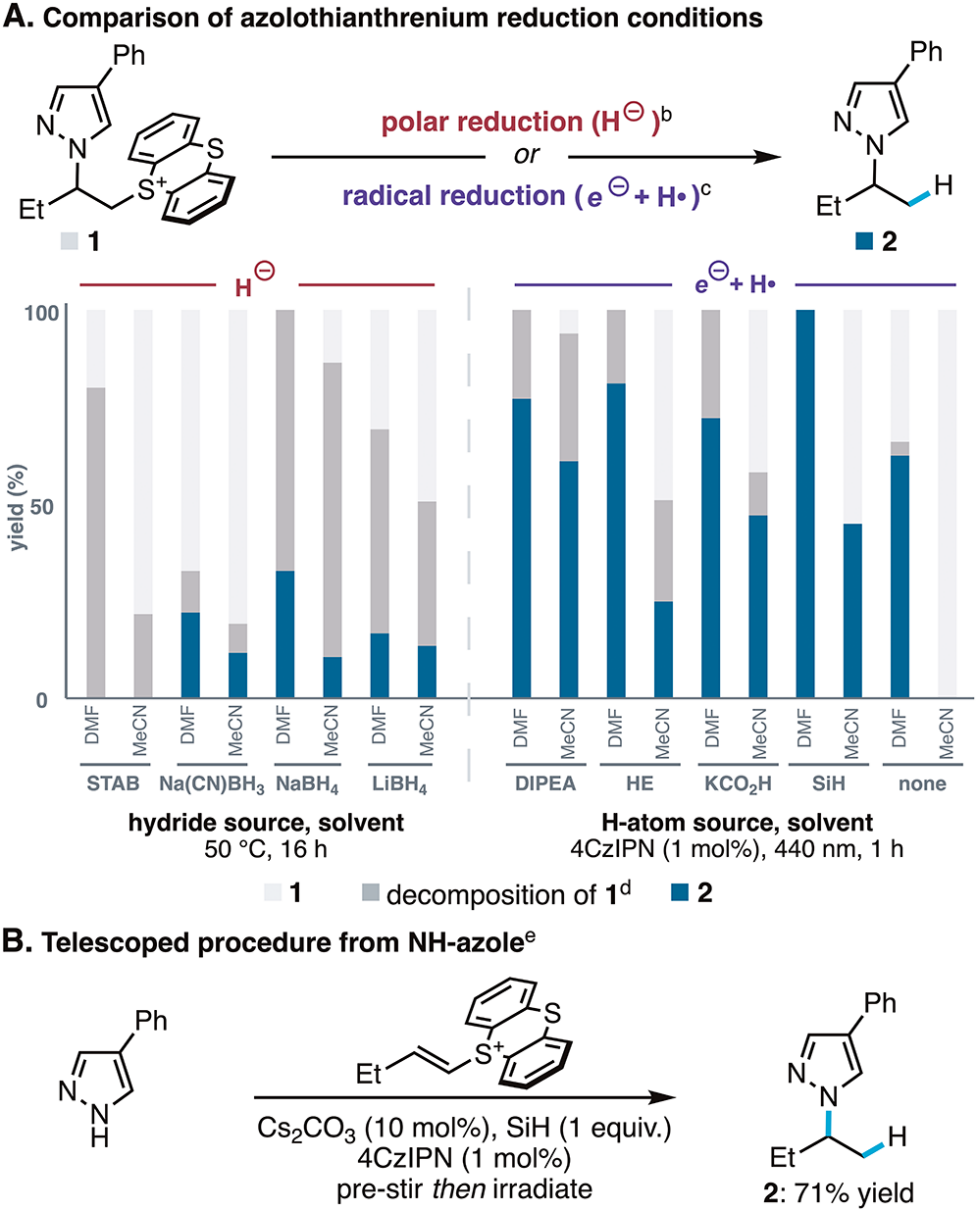
Development of a Reduction Procedure^a^

a Reactions were conducted on a 0.05 mmol scale under argon unless otherwise noted. Yields were determined by ^1^H NMR.
b Reactions were conducted using 1.1 equiv of hydride reagent and solvent (0.05 M) at 50 °C for 16 h.
c Reactions were conducted using hydrogen atom source (1 equiv), 4CzIPN (1 mol %) as the photocatalyst, and solvent (0.05 M) under 440 nm irradiation for 1 h.
d Decomposition of **1** refers to any conversion of **1** which did not result in **2**. For further details regarding specific byproducts observed, see SI.
e Reaction was conducted under air using azole (1 equiv), alkenylthianthrenium salt (1 equiv), Cs_2_CO_3_ (10 mol %), 4CzIPN (1 mol %), and HSi­(TMS)_3_. After 3 h of stirring, the mixture was irradiated at 440 nm for a subsequent 3 h. STAB = sodium triacetoxyborohydride; DIPEA = *N*,*N*-diisopropylethylamine; HE = Hantzsch ester (Diethyl 1,4-dihydro-2,6-dimethyl-3,5-pyridinedicarboxylate); SiH = tris­(trimethylsilyl)­silane; 4CzIPN =1,2,3,5-Tetrakis­(carbazol-9-yl)-4,6-dicyanobenzene.

We next questioned whether sequential one electron reduction steps could unlock formal hydride delivery without promoting elimination pathways. We were inspired by the use of arylthianthrenium salts as radical precursors across mechanistically diverse reactions,^13a,17,18^ as well as emerging reports that alkylthianthrenium salts can be converted to radical intermediates.^19^ Gratifyingly, we found that a wide variety of photocatalytic conditions furnished the desired product in moderate to high yield. While common reductive quenchers in photoredox catalysis (*e.g*., trialkylamines, Hantzsch-type esters, and formate salts) offer stark improvements over polar hydride reagents, these electron-rich reductants each still promote meaningful decomposition of electrophilic **1**.^20^ In contrast, however, tris­(trimethylsilyl)­silane acts as an innocent H-atom source and provides quantitative C–S reduction. In fact, even using DMF as the solvent under photocatalytic conditions results in selective protodethianthrenation, albeit with a meaningfully reduced rate, presumably with DMF acting as an H-atom source.^21^

Having identified conditions that promote clean reduction of 1,2-azolothianthrenium salts, we next aimed to develop an operationally simple protocol to carry out the complete azole alkylation-reduction sequence in one-pot (Scheme 1B). We suspected that, without irradiation, the silane and photocatalyst would have no impact on the addition of azoles across alkenyl thianthrenium salts. Consistent with this supposition, we devised a straightforward procedure where the components of both steps are added at the outset, with a prestir in the dark followed by irradiation. We found that this protocol delivers the alkylated azole product in high yield without any precautions to exclude air or moisture.

With an established one-pot azole alkylation procedure, we next explored the scope of this protocol (Table 1). We first evaluated the range of *N*-alkyl steric profiles that are accessible via this transformation. The method delivered azoles with *N*-alkyl substituents ranging from primary to tertiary (**3**–**6**). Moreover, we found that we could leverage internal alkenyl thianthrenium electrophiles to deliver both cyclic and acyclic *N*-alkyl substituents (**7**–**9**). Successful transformation of the secondary sulfonium intermediate sharply contrasts prior work, which established that substitution of these hindered electrophiles is precluded by competitive elimination pathways even with non-basic nucleophiles.^22^ With respect to the azole nucleophiles, we found the process to be broadly effective, productively engaging azaindoles (**3**), imidazoles (**4**, **15**), pyrazoles (**5**–**11**, **13**), triazoles (**12**), indazoles (**14**, **16**), and purines (**17**). Of note, due to the reversible conjugate addition of ambident nucleophiles into alkenylthianthrenium salts, the net azole alkylation occurs with exquisite *N*-regioselectivity.^23^ Finally, the synthetic sequence proved compatible with diverse functional groups across both coupling partners, including trifluoromethyl groups (**5**, **10**), unsaturated (**6**, **13**) and saturated (**9, 11**–**13**) heterocycles, esters (**7**, **16**), phthalimides (**10**, **15**), aryl and alkyl halides (**9**, **10**, **15**, **17**), sulfonamides (**11**), and boronic esters (**14**).

**Table 1:**
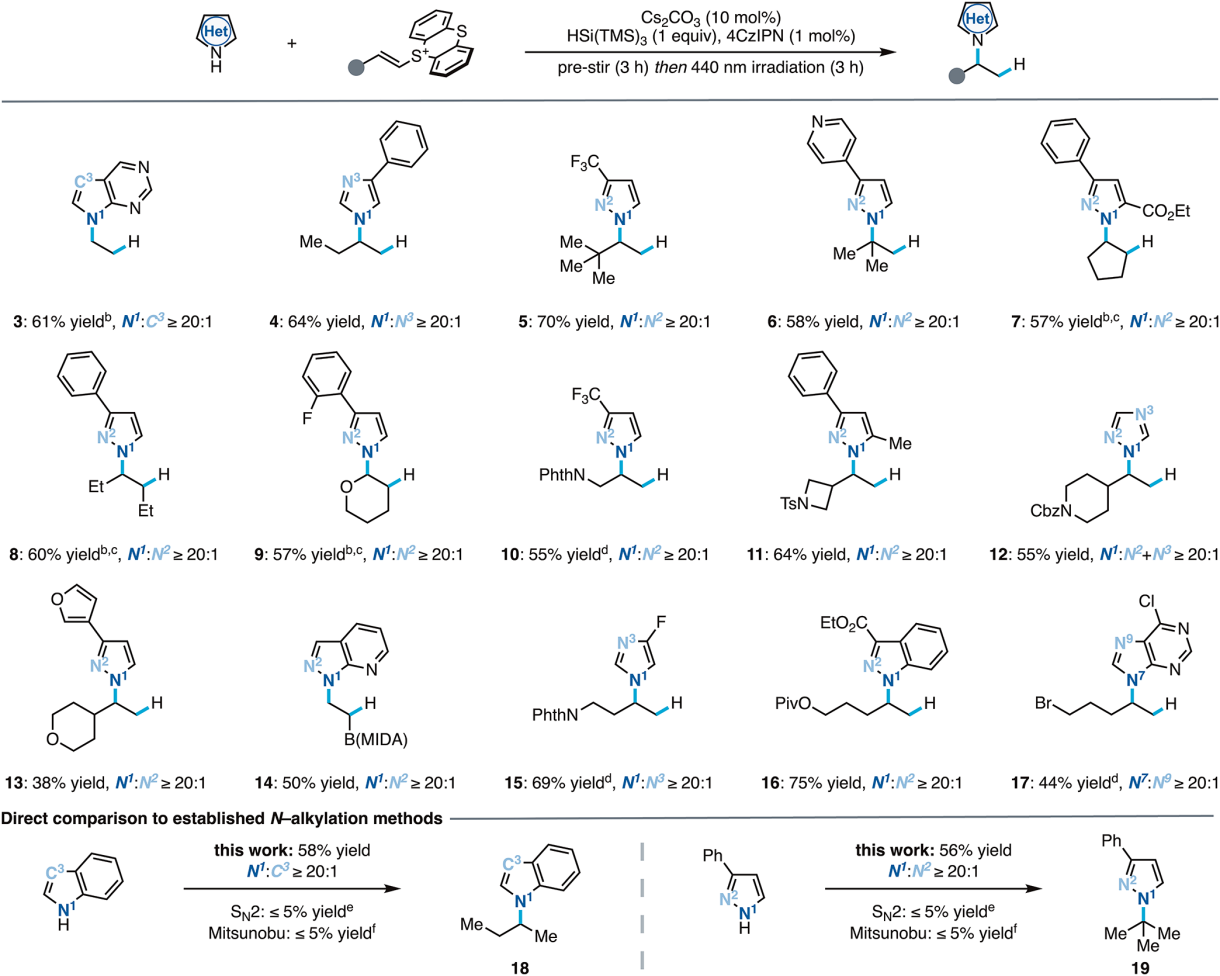
Scope of Azole Alkylation Reaction and Comparison to Established *N*-Alkylation Methods^a^

a Reactions were run under air with 10 mol % Cs_2_CO_3_, 1 mol % 4CzIPN, 1 equiv. HSi­(TMS)_3_ in 2:1 DMF:MeCN (0.05 M) at room temperature for 3 h (dark) followed by 3 h of irradiation at 440 nm. Yields are of purified material unless otherwise noted. See SI for further details. 4CzIPN = 1,2,3,5-tetrakis­(carbazol-9-yl)-4,6-dicyanobenzene; Phth = phthalimide; Ts = toluenesulfonyl; Cbz = benzylcarbonate; MIDA = *N*-methyliminodiacetic acid; Piv = pivaloyl.

b LiOtBu (20 mol %) instead of Cs_2_CO_3_

c MeCN (0.05 M) instead of DMF:MeCN mixture, yield determined by ^1^H NMR

d HSi­(TMS)_3_ excluded from the reaction mixture.

e A variety of S_N_2 conditions reported in the literature were tested. See SI for full experimental details.

f A variety of Mitsunobu conditions reported in the literature were tested. See SI for full experimental details.

As illustrative examples contextualizing how our new protocol complements classic alkylation methods, we targeted the synthesis of two deceptively simple *N*-alkylated heterocycles, **18** and **19**. Indole **18** was previously prepared in 6% yield using a classic S_N_2 disconnection,^24^ and, surprisingly, **19** remains an unknown compound. In both cases, the new hydroazolation-reduction protocol delivered alkylation products in synthetically useful yields, whereas varied S_N_2 and Mitsunobu conditions categorically failed to promote alkylation (see SI for details). Taken together with our broader evaluation of the scope, these data indicate that this new process represents a versatile route to *N*-alkylated heterocycles that complements established methodologies.

We next questioned whether we could leverage this SET reduction of 1,2-azolothianthrenium intermediates to promote C–C bond-forming radical reactivity, rather than HAT (Table 2). In principle, this would provide modular routes to *N*-alkyl azole products where the corresponding internal alkenes for hydroazolation would be laborious to prepare, pose liabilities,^25^ or both. Successful realization of this strategy, however, would require that radical reactivity outcompetes HAT to an unstabilized primary radical. We found that omitting labile hydrogen atom sources entirely enabled the photocatalytic Minisci-type radical cyclization into (hetero)­aromatic systems, furnishing azoles bearing partially saturated bicyclic systems as *N*-substituents (**20**–**21**). For net-reductive radical derivatization, we identified that Hantzsch ester is a well-matched photosensitizer and terminal H-atom source,^18h,18i,19b,19c^ promoting the cyclization of 1,2-azolothianthrenium salts bearing pendant alkenes and alkynes to (hetero)­cyclic products (**22**–**25**). Intermolecular variants of these classic radical reactions were similarly effective, allowing introduction of alkyl (**26**–**27**), alkenyl (**28**), and both electron-rich and -deficient heteroaryl groups (**2930**). Across both intra- and intermolecular reactivity, these C–C bond-forming processes were compatible with diverse azoles, including triazoles (**20**, **26**), purines (**21**), azaindoles (**22)**, pyrazoles (**23**, **27**, **28**), indazoles (**24**, **30**), and imidazoles (**25**, **29**). These reactions delivered products bearing a diverse range of functional groups on both the azole and *N*-alkyl substituent, including aryl bromides (**20**), organofluorides (**23**, **25**), azetidines (**26**), sulfones (**27**), ketones (**28**), nitriles (**29**), piperidines (**29**), and sulfonamides (**23**, **26**).

**Table 2:**
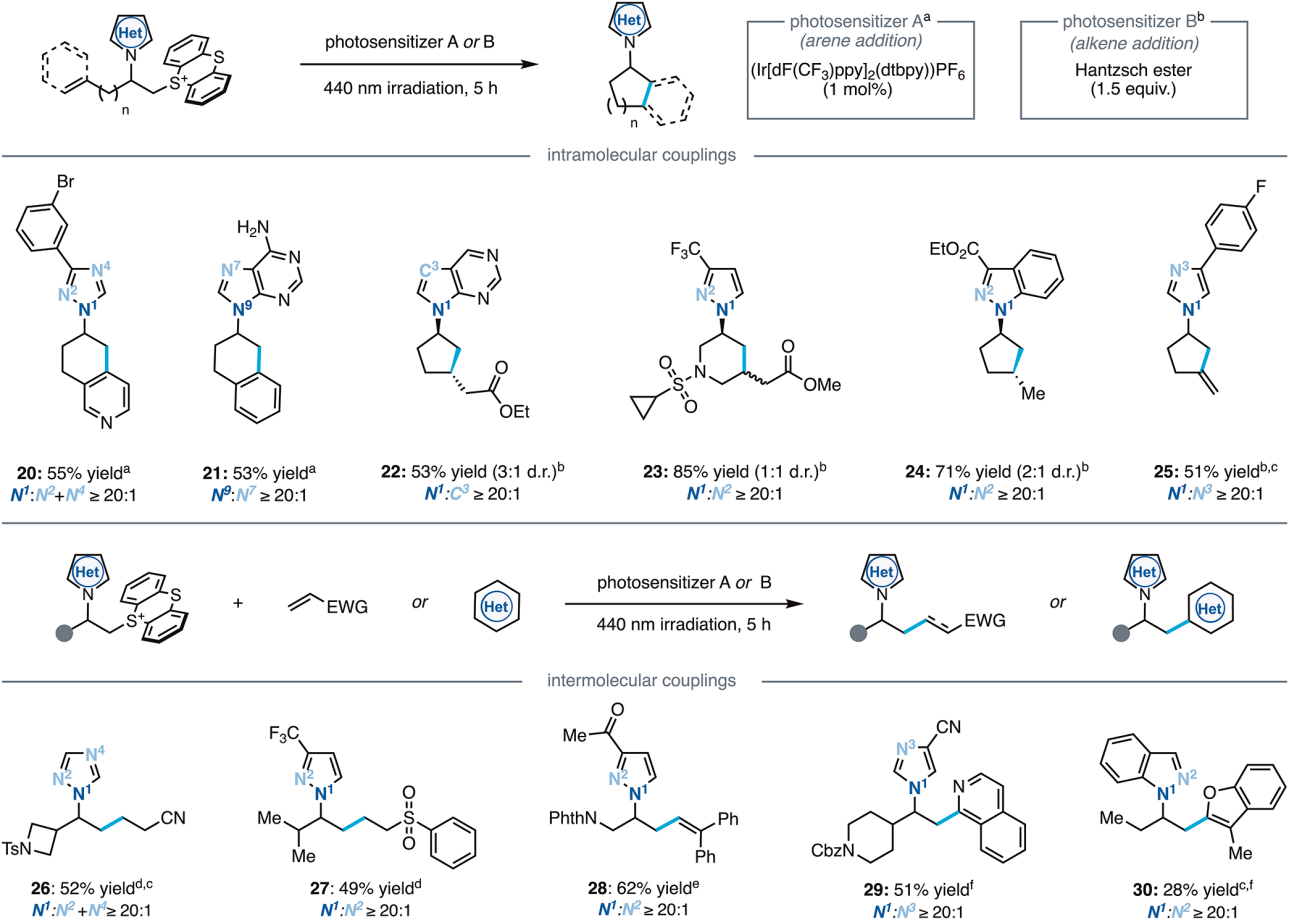
Scope of C–C Bond Forming Reactions

a Reaction was run under argon using (Ir­[dF­(CF_3_)­ppy]_2_(dtbpy))­PF_6_ (1 mol %) in MeCN (0.05 M) with 440 nm irradiation for 5 h.

b Reaction was run under argon using Hantzsch ester (1.5 equiv) in DMA (0.1 M) with 440 nm irradiation for 5 h.

c Yield determined by ^1^H NMR.

d Reaction was run according to conditions A with alkene (2 equiv) added.

e Reaction was run under argon using 4CzIPN (1 mol %) and diphenylethylene (2 equiv) in DMA (0.1 M) with 440 nm irradiation for 5 h.

f Reaction was run according to conditions B with heteroarene (1.5 equiv) in DMSO (0.05 M).

Having established the synthetic utility of the SET activation of 1,2-azolothianthrenium intermediates, we next aimed to understand the hydrogenolysis conditions that underpin the hydroazolation protocol. While our optimization studies identified that both tris­(trimethylsilyl)­silane and DMF (as solvent) are competent hydrogen-atom donors, we observed that they have an unexpected synergistic impact on reaction rate (Figure 2A). We questioned whether, rather than simply serving as sources of hydrogen atoms, their combination actually accelerates a step other than HAT. To probe whether the HAT step was rate-determining, we performed a series of kinetic isotope effect (KIE) studies with deuterated hydrogen atom sources (Figure 2B). While the one-pot competition experiment showed the expected primary KIE of 2.6 ± 0.2 for HAT,^26^ two-pot competition experiments revealed that deuteration of the hydrogen atom sources had a negligible impact on reaction rate (KIE of 0.90 ± 0.38). These data clearly indicate that the HAT step, while irreversible, is not rate-determining.^27^

**Figure 2:**
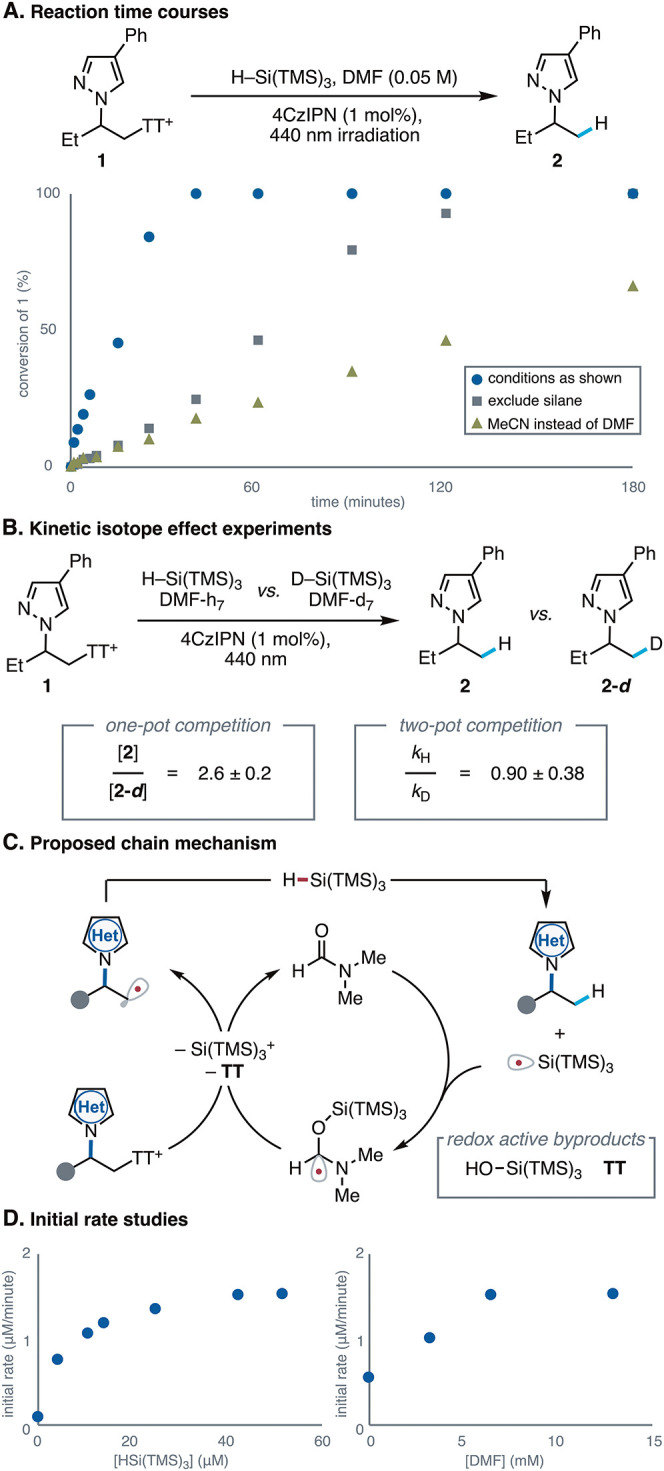
Mechanistic studies.

Given that accelerating HAT would have no impact on rate, we suspected that silane and DMF cooperatively generate the key radical intermediate from the 1,2-azolothianthrenium electrophile. This conclusion led to a working mechanistic model wherein: (1) addition of a silyl radical into the carbonyl of DMF generates an active reductant; (2) rate-determining SET from this adduct to the alkylthianthrenium salt produces a primary radical; (3) HAT from silane to this radical liberates product and regenerates a silyl radical to propagate a chain (Figure 2C). Consistent with this proposed rate-determining SET from a silane-DMF adduct, initial rate studies independently varying concentrations of silane or DMF reveal ordered regimes for each of these reagents at low concentration followed by saturation kinetics under optimal conditions (Figure 2D).^28^ Interestingly, the byproducts expected under this proposed mechanism—thianthrene and silanol formed via silylium hydrolysis^200^—possess appropriate redox potentials for reductive quenching of 4CzIPN*.^29^ We suspect that these redox active byproducts serve a critical role by sustaining photocatalytic reinitiation since neither tris­(trimethylsilyl)­silane nor **1** quenches 4CzIPN* (see SI for details).^30^ While the precise initiation mechanism remains unclear, inefficient photolysis of **1** is a plausible pathway to initially generate a radical intermediate.^31^ Taken together, these studies outline a new mechanistic manifold to promote radical hydrogenolysis of electrophiles that are sensitive to more conventional Brønsted basic terminal reductants.

Overall, we have introduced a mechanistically distinct approach for the synthesis of previously difficult-to-access *N*-alkyl azoles via azole attack on alkenylthianthrenium electrophiles followed by SET reduction. This unlocks a formal Markovnikov hydroazolation protocol given that alkenylthianthrenium salts are readily derived from unactivated alkenes. Furthermore, extending the radical diversification to C–C bond-forming reactivity gives rise to unsymmetrical *N*-alkylated azole structures which would be particularly challenging to access via any existing method. Mechanistic investigations reveal an unusual reductive activation mode, wherein silane and DMF react to form a transient adduct that serves as a strong reductant under otherwise mild conditions. This study fundamentally expands the scope of *N*-alkyl azoles which can readily be prepared and studied, and, more broadly, provides a new approach to diversify elimination-prone alkylthianthrenium salts, an emerging class of electrophiles.

## Supplementary Material



## Notes

Experimental procedures, characterization data, and spectra (PDF)
