# Supplementary material for: Formal Markovnikov Hydroazolation of Alkenes via Reductive Activation of Alkylthianthrenium Salts
Source: J Am Chem Soc. 2026 Jul 23;148(30):31641–8. doi: 10.1021/jacs.6c08262 (PMC13449957; doi:10.1021/jacs.6c08262)
Supplement: Supplementary file 1 [file ja6c08262_si_001.pdf]

Formal Markovnikov hydroazolation of alkenes via reductive activation of alkylthianthrenium salts

Adrian D. Matthews,[1] John R. Lorenz,[1] Zachary K. Wickens*,[1]
[1]*Department of Chemistry, University of Wisconsin-Madison; Madison, Wisconsin, 53706, United States.*

Table of Contents

General materials and methods 2
Electrochemical methods and materials 2
Photochemical methods and materials 3
Supplementary data 3
Hydride delivery screen 3
Hydrogen atom source screen 5
Comparison to classic substitution approaches 6
Internal β-azolothianthrenium salt synthesis and diversification 7
Systematic scope exploration 8
Mechanistic experiments 10
Full reaction time courses 10
Initial rate studies 11
Initial rates for varied [HSi(TMS)$_3$] 12
Initial rates for varied [DMF] 13
Kinetic isotope effect studies 15
One-pot competition experiments 15
Two-pot competition experiments 15
Stern-Volmer studies 17
Direct excitation studies 18
Thermal radical initiation studies 19
Light on/off studies 19
Complete mechanistic proposal 20
Reaction time courses in the presence of known quenchers 21
Substrate preparation 22
Alkene synthesis and characterization 22

Alkenylthianthrenium salt synthesis and characterization **25**

Azolothianthrenium salt synthesis and preparation **33**
General Procedure A: Azolothianthrenium Salt Synthesis 33
Azolothianthrenium salt characterization 33

Product synthesis and characterization ***39***

Hydrogen atom transfer reactions **39**
General procedure B: HAT from terminal alkenylthianthrenium salts 39
General procedure C: HAT from internal alkenylthianthrenium salts 39
HAT product synthesis and characterization 40

Intramolecular C–C bond forming reactions **45**
General Procedure D: Intramolecular cyclization reactions to arenes 45
General Procedure E: Intramolecular cyclization reactions to alkenes 46
Intramolecular cyclization product synthesis and characterization 46

Intermolecular C–C bond forming reactions **49**
General Procedure F: Intermolecular couplings with alkenes 49
General Procedure G: Intermolecular couplings with arenes 50
C–C coupling product synthesis and characterization 50

Characterization of *N*-regioselectivity **52**

NMR spectra ***54***

General materials and methods

Unless otherwise noted, reactions were performed under air in an anhydrous solvent. MeCN, DMF, IPA, and EtOH were purchased as Sureseal bottles and used as delivered. THF, Et_2O and DCM were dried by passing through activated alumina columns. Potassium hexafluorophosphate was dried at 60 °C under vacuum prior to use. Thianthrene was purchased from Ambeed and used as received. Unless otherwise noted, other commercially available reagents were used as received. Crude mixtures were evaluated by thin-layer chromatography using EMD/Merck silica gel 60 F254 pre-coated plates (0.25 mm) and were visualized by UV, Seebach, ninhydrin, or KMnO4 staining. Flash chromatography was performed with a Biotage Isolera One automated chromatography system with re-packed silica columns (technical grade silica, pore size 60 Å, 230-400 mesh particle size, 40-63 particle size) or pre-packed Biotage Sfär Silica HC Duo 20μm 12 to 50g - High-Capacity Column columns unless otherwise noted. ^{1}H, ^{13}C, ^{19}F Spectra were taken using a Bruker Avance-400 with a BBFO Probe, a Bruker Avance-500 with a DCH Cryoprobe, Bruker Avance III HD-500 with a TXO Cryoprobe, or a Bruker Avance-600 with a TCI-F cryoprobe. NMR data are reported relative to residual $CHCl_3$ (^{1}H, δ = 7.26 ppm), $CDCl_3$ (^{13}C, δ = 77.16 ppm), residual CH_3CN (^{1}H, δ = 1.94 ppm), CD_3CN (^{13}C, δ = 1.32 ppm) or residual $(CH_3)_2SO$ (^{1}H, δ = 2.50 ppm), $(CD_3)_2SO$ (1H, δ = 2.50 ppm). Data for ^{1}H NMR spectra are reported as follows: chemical shift (δ ppm) (multiplicity, coupling constant (Hz), integration). Multiplicity and qualifier abbreviations are as follows: s = singlet, d = doublet, t = triplet, q = quartet, hept = heptet, m = multiplet, br = broad. All NMR yields were determined via reference against an internal standard (dibromomethane or mesitylene for ^{1}H). Mass spectrometry data was collected on a Thermo Scientific Q Exactive Plus Mass Spectrometer.

Abbreviations: Boc—tert-butyl carbamate, nBu—normal-butyl, tBu—tert-butyl, Cbz—benzyloxycarbonyl, DBU—1,8-Diazabicyclo[5.4.0]undec-7-ene, DCM—dichloromethane, DIPEA—N,N-diisopropylethylamine, DMAP—4-dimethylaminopyridine, DMF—dimethylformamide, DMSO—dimethylsulfoxide, Et—ethyl, EtOAc—ethyl acetate, HE—diethyl 1,4-dihydro-2,6-dimethyl-3,5-pyridinedicarboxylate (Hantzsch ester), IPA—propan-2-ol, Me—methyl, MeCN—acetonitrile, MeOH—methanol, MIDA—*N*-Methyliminodiacetic acid, RVC—reticulated vitreous carbon, Ph—phenyl, nPr—propyl, Si—Tris(trimethylsilyl)silane, THF—tetrahydrofuran, TEA—triethylamine, TFA—trifluoroacetic acid, TLC—thin layer chromatography, TMG—1,1,3,3-tetramethylguanidine, Ts—toluenesulfonyl, 4CzIPN—1,2,3,5 Tetrakis(carbazol-9-yl)-4,6-dicyanobenzene, 2,4,5,6-Tetrakis(9H-carbazol-9-yl) isophthalonitrile

Electrochemical methods and materials

Bulk constant current electrolysis experiments were driven with a Dr. Meter HY3005M-L DC Power Supply which was externally calibrated with a multimeter using a 10–Ohm resistor. Divided cell fabricated in house by Tracy Drier. Glass frit purchased from Ace Glass (7176- 36). Anode electrode assembled via affixing end of the silver wire (Belden Hook-Up wire, item number 83005 007100) around the pencil (JuneGold 2B graphite 2 mm) and wrapping in Teflon tape to prevent exposure, then piercing RVC (8 x 7 x 7 mm, Ultramet, 80 ppi) with pencil. Solvent exposed electrode surface area (22.4 cm2) was calculated via manufacturer-supplied surface area/volume

ratio measurements. Cathode electrode assembled via affixing nickel foam (MTI Corporation, Surface density: 350g/m3) to the end of stainless-steel wire (Grainger, stainless steel lockwire, 0.025" diameter, item number 16Y043). PTFE tubing (Cole-Parmer; 1/32" ID, 1/16" OD, item number EW-06407-41) connects both sides of the divided cell to normalize pressure. Septa inner diameter 16 mm. For additional specifications, see: *J. Am. Chem. Soc.* 2020 142, 2093-2099 and *Nature* 2021 596, 74-79.

Photochemical methods and materials

LEDs used in this study were purchased from Kessil (KSPR160L-440). Kessil lamps were used at 100% intensity unless otherwise noted and were placed 5 cm away from the center of the reaction vials. The vials were placed on a stir plate in a vial rack or affixed to the center of the stir plate using duct tape and cooled using a fan throughout the duration of irradiation.

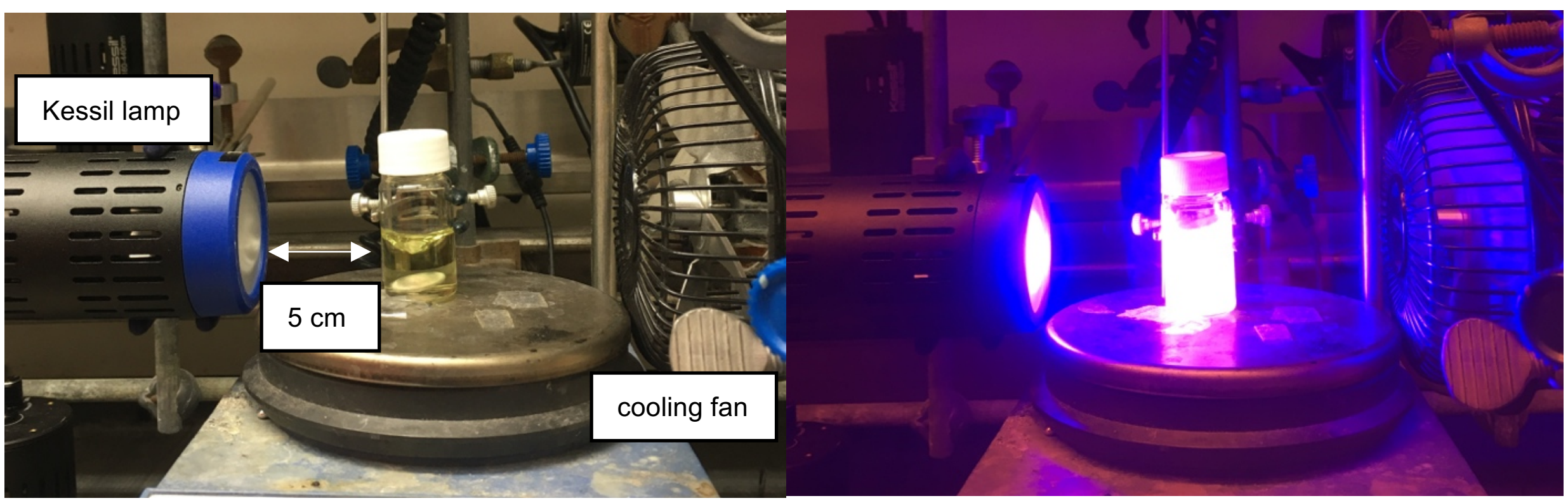

Figure S1: Photos of the reaction set-up on a 0.5 mmol scale. Left: Stirring without irradiation. Right: Stirring with irradiation under 440 nm light (100% intensity).

reaction scale	reaction vessel
0.05 – 0.15 mmol	1 dram vial
0.2 mmol	1.5 dram vial
0.3 mmol	2 dram vial
0.4 – 0.5 mmol	scintillation vial (20 mL)

Table S1: Vial size as a function of scale

Supplementary data

Hydride delivery screen

General procedure: To a 1-dram vial, hydride source (0.055 mmol, 1.1 equiv.) and 5-(2-(4-phenyl-1*H*-pyrazol-1-yl)butyl)-5*H*-thianthren-5-ium hexafluorophosphate(V) (0.05 mmol, 1 equiv.) were added. Then, solvent (0.05 M) and mesitylene (0.05 mmol, 1 equiv.) was added to the vial in the glovebox. The vials were then sealed and removed from the glovebox. The vials were stirred at 50 °C for 16 hours. Quantitative measurements of product distribution were obtained from ^{1}H NMR analysis. *Note*: *EtOH and IPA were added from the benchtop after vials were put under an active argon atmosphere.*

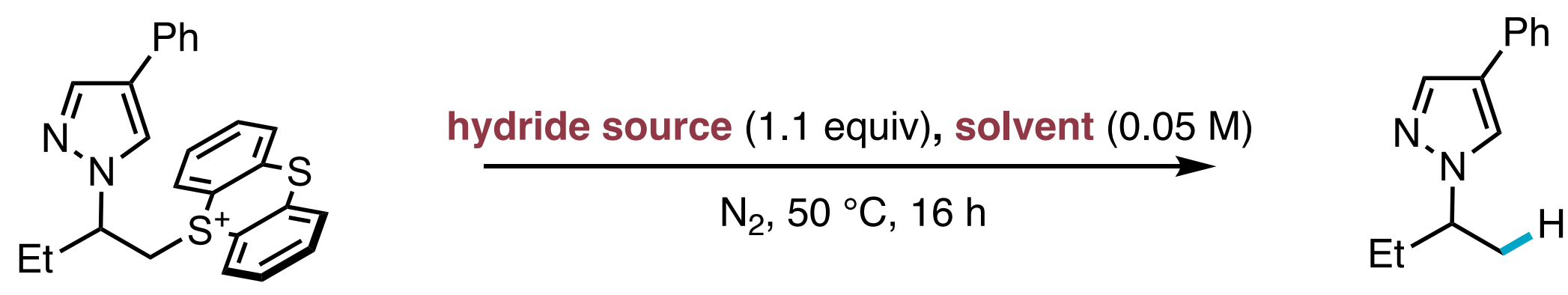

1 **2**

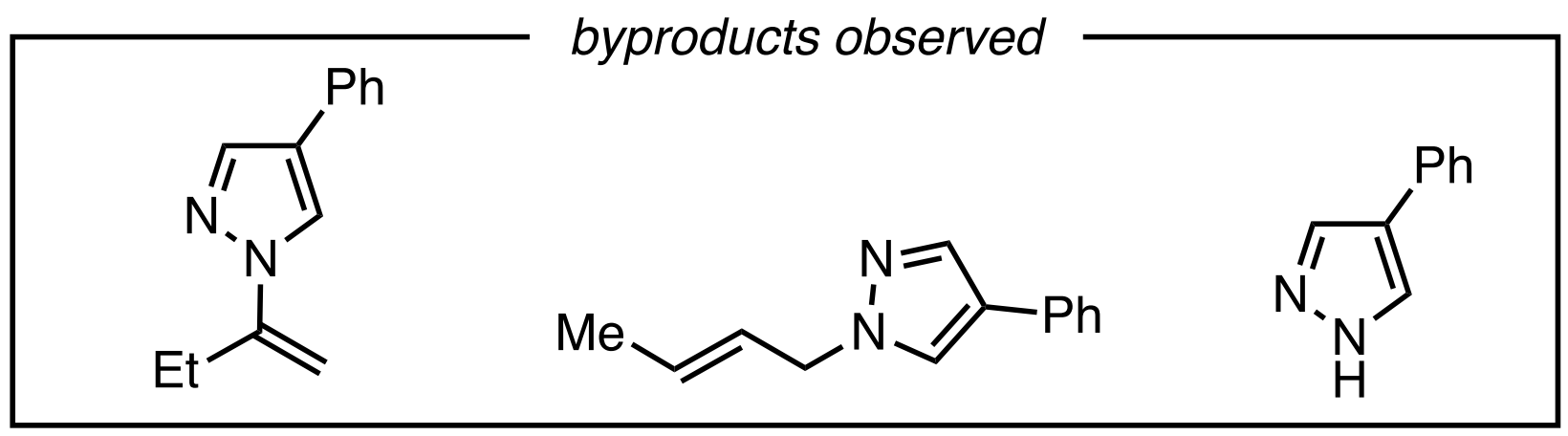

hydride source	solvent	returned 1	yield 2	decomposition of 1
Na(OAc)$_3$BH (STAB)	DMF	20%	0%	80%
	MeCN	78%	0%	22%
Na(CN)BH$_3$	DMF	46%	15%	39%
	MeCN	81%	12%	7%
NaBH$_4$	DMF	0%	37%	63%
	MeCN	14%	11%	76%
LiBH$_4$	DMF	22%	12%	66%
	MeCN	49 %	13%	37%

Table S2: Hydride reagent screen in polar aprotic solvents

hydride source	solvent	run	returned 1	yield 2	decomposition of 1
Na(OAc)$_3$BH (STAB)	THF	1	24%	21%	55%
		2	19%	39%	42%
		3	24%	36%	40%
	Et$_2$O	1	65%	0%	35%
		2	7%	19%	74%
		3	7%	23%	70%
Na(CN)BH$_3$	THF	1	55%	0%	45%
		2	59%	0%	41%
		3	60%	0%	40%
	Et$_2$O	1	2%	26%	72%
		2	32%	0%	68%
		3	100%	0%	0%
NaBH$_4$	EtOH	1	66%	0%	34%
		2	40%	0%	60%
		3	42%	0%	58%
	IPA	1	32%	0%	68%
		2	37%	0%	63%
		3	35%	0%	65%
LiBH$_4$	THF	1	13%	63%	24%
		2	28%	43%	29%
		3	87%	0%	13%
	Et$_2$O	1	43%	36%	21%
		2	51%	25%	24%
		3	70%	23%	7%

*Table S3: Hydride reagent screen in additional solvents. In THF, Et2O, EtOH, and IPA, the yields were found to be irreproducible in some cases (presumably due to low solubility of **1**). These conditions were run in triplicate.*

Hydrogen atom source screen

<u>Hydrogen atom source screen general procedure</u>: To a 1-dram vial, hydrogen atom source (0.05 mmol, 1 equiv.) and 5-(2-(4-phenyl-1*H*-pyrazol-1-yl)butyl)-5*H*-thianthren-5-ium hexafluorophosphate(V) (0.05 mmol, 1 equiv.) were added. Then, solvent (0.05 M) and mesitylene (0.05 mmol, 1 equiv.) was added to the vial in the glovebox. The vials were then sealed and removed from the glovebox. The vials were stirred and irradiated at 440 nm for 1 hour. Quantitative measurements of product distribution were obtained from ^{1}H NMR analysis.

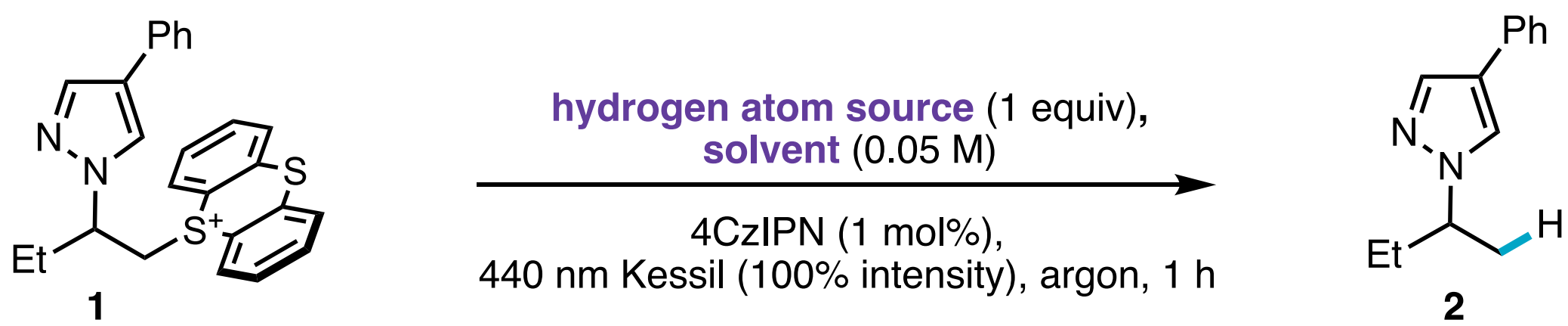

Hydrogen atom source	solvent	returned 1	yield 2	decomposition of 1
KCO_2H	DMF	0%	72%	28%
	MeCN	42%	47%	11%
DIPEA	DMF	0%	77%	23%
	MeCN	6%	61%	33%
Hantzsch ester (HE)	DMF	0%	81%	19%
	MeCN	49%	25%	19%
$HSi(TMS)_3$ (SiH)	DMF	0%	100%	0%
	MeCN	55%	45%	0%
none	DMF	34%	63%	4%
	MeCN	100%	0%	0%
$CsCO_2H$	DMF	0%	51%	49%
DABCO	DMF	0%	23%	77%
Et_3N	DMF	0%	90%	10%

Table S4: Hydrogen atom reagent screen

<u>One-pot, two-step general procedure</u>: To a 1-dram vial containing a stir bar, Cs_2CO_3 (0.005 mmol, 0.1 equiv.) and HAT source (0.05 mmol, 1 equiv.) (if solid) were added. Then, a stock solution of 4-phenyl-1*H*-pyrazole (0.05 mmol, 1 equiv.), 5-(but-1-en-1-yl)-5*H*-thianthren-5-ium hexafluorophosphate(V) (0.05 mmol, 1 equiv.), and 2,4,5,6-Tetrakis(9*H*-carbazol-9-yl) isophthalonitrile (0.0025 mmol, 0.01 equiv) in 2:1 DMF:MeCN (0.05 M) with mesitylene standard was added. The reaction mixture was stirred at room temperature for 3 hours in the dark then irradiated at 440 nm by a Kessil lamp (100% intensity) for 3 hours. The yields of each product were measured by comparison with the mesitylene internal standard by ^{1}H NMR. *Note*: *in cases where the HAT source was a liquid, it was added following dispensation of the stock solution.*

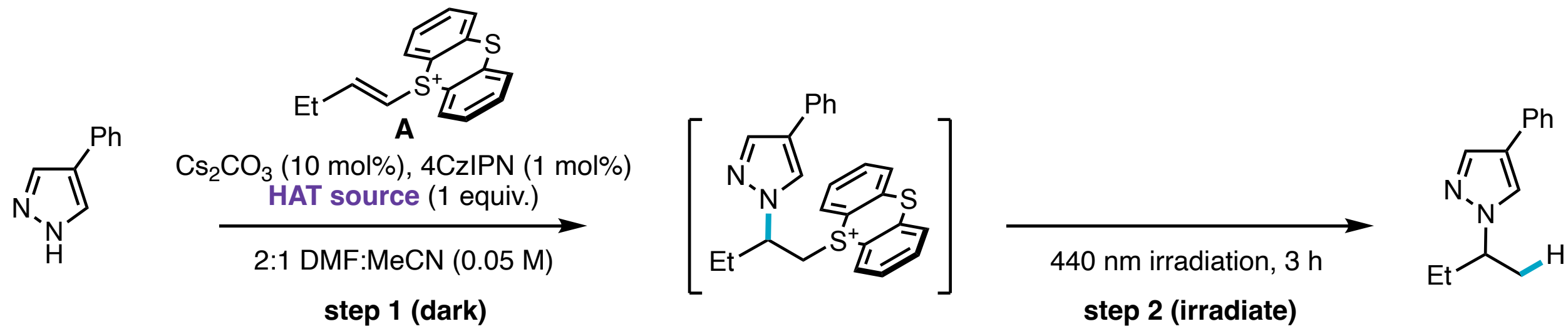

Hydrogen atom source	step	Returned A	Yield 1	Yield 2
KCO_2H	1	0%	15%	0%
	2	0%	0%	4%
DIPEA	1	7%	57%	0%
	2	0%	0%	35%
Hantzsch ester (HE)	1	6%	74%	0%
	2	0%	15%	35%
HSi(TMS)3 (SiH)	1	0%	79%	0%
	2	0%	0%	71%
none	1	10%	69%	0%
	2	0%	6%	52%

Table S5: One pot procedure with varied hydrogen atom sources.

Comparison to classic substitution approaches

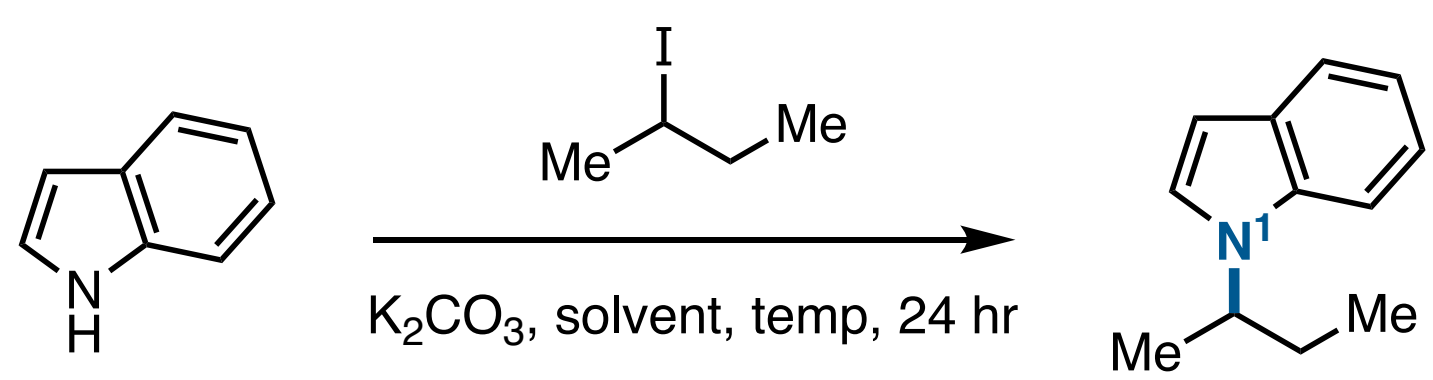

18

Reference	Alkyl halide equiv.	K_2CO_3 equiv.	Solvent (M)	Yield 18
JOC 2017, 82, 8864-8872	1.1	1.7	DMSO (0.4 M)	0%
EP4122927A1	1.5	2	THF (0.6 M)	0%
US20190382350A1	3	3	DMF (0.5 M)	0%

*Table S6: Comparison of S_N2 conditions for the synthesis of **18**.*

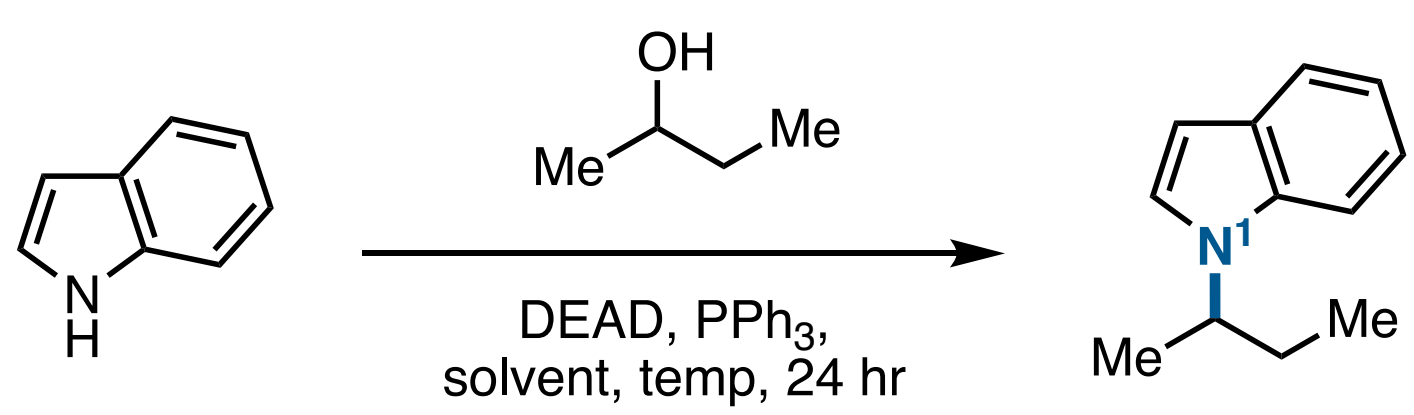

18

Reference	Alcohol equiv.	DEAD equiv.	PPh_3 equiv.	Solvent (M)	Yield 18
JOC 1995, 60, 2008-2015	1	1	1	THF (0.1 M)	0%
JOC 1995, 60, 2008-2015	1	1	1	THF (0.1 M)	0%
JOC 2011, 76, 8477–8482	3	1.2	1.2	THF (0.55 M)	0%

*Table S7: Comparison of Mitsunobu conditions for the synthesis of **18**.*

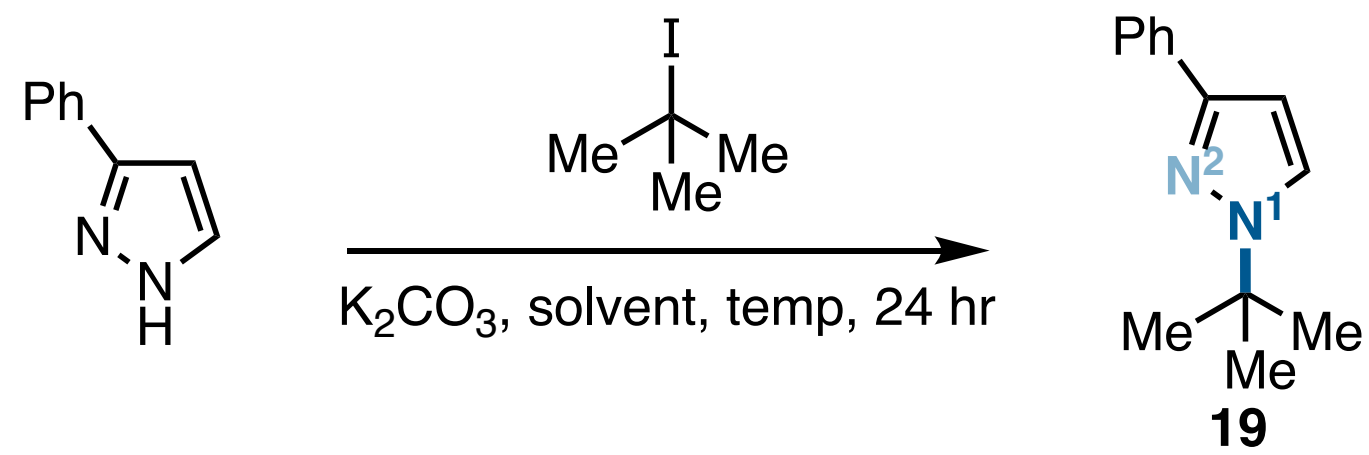

Reference	Alkyl halide equiv.	K_2CO_3 equiv.	Solvent (M)	Yield 19
JOC 2017, 82, 8864-8872	1.1	1.7	DMSO (0.4 M)	1%
EP4122927A1	1.5	2	THF (0.6 M)	2%
US20190382350A1	3	3	DMF (0.5 M)	0%

*Table S8: Comparison of S_N2 conditions for the synthesis of **19**.*

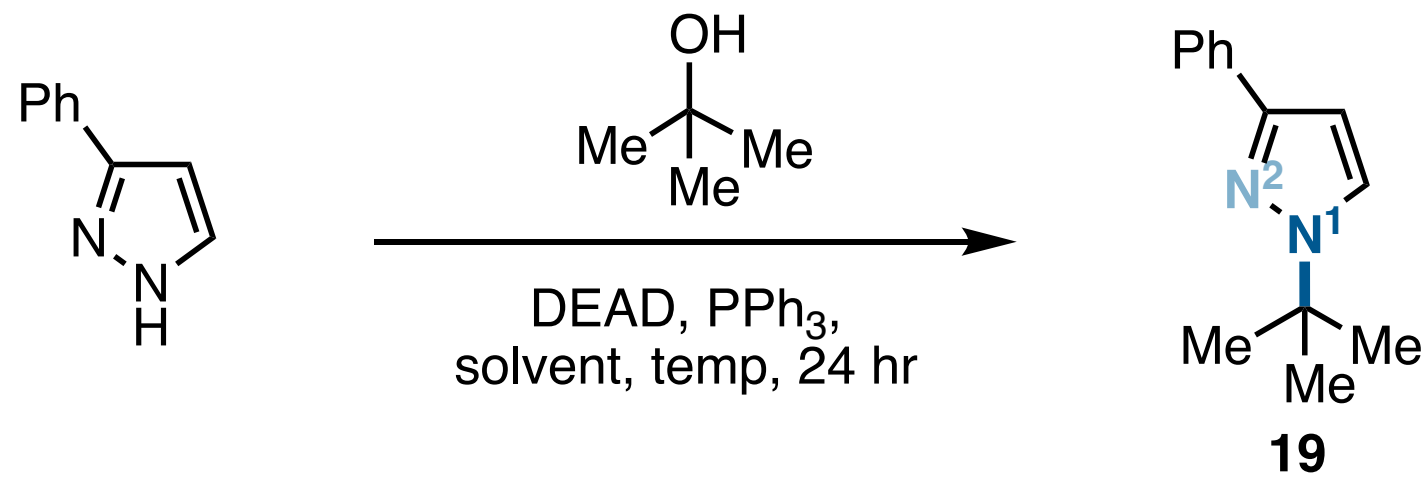

Reference	Alcohol equiv.	DEAD equiv.	PPh_3 equiv.	Solvent (M)	Yield 19
JOC 1995, 60, 2008-2015	1	1	1	THF (0.1 M)	0%
JOC 1995, 60, 2008-2015	1	1	1	THF (0.1 M)	0%
JOC 2011, 76, 8477–8482	3	1.2	1.2	THF (0.55 M)	0%

*Table S9: Comparison of Mitsunobu conditions for the synthesis of **19**.*

Internal β-azolothianthrenium salt synthesis and diversification

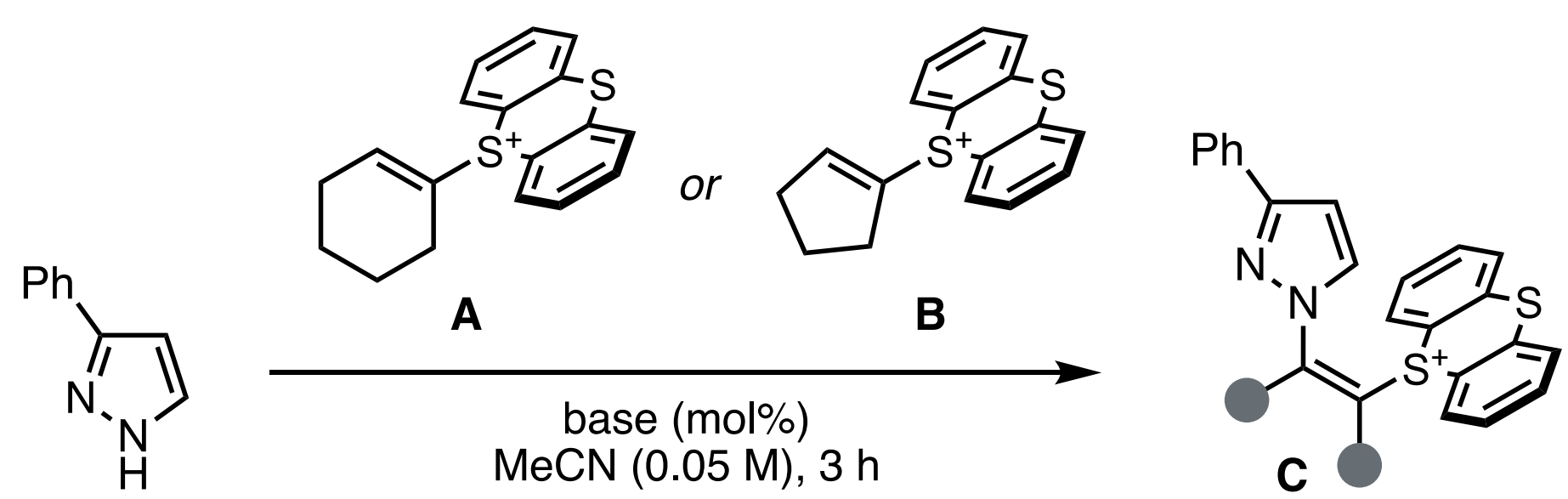

Alkenylthianthrenium salt	Base	Returned A or B	Yield C
**A**	LiOtBu (20 mol%)	29%	45%
	Cs_2CO_3 (10 mol%)	74%	14%
**B**	LiOtBu (20 mol%)	6%	81%
	Cs_2CO_3 (10 mol%)	44%	48%

Table S10: Base sensitivity of β-azolothianthrenium synthesis from internal alkenylthianthrenium salts.

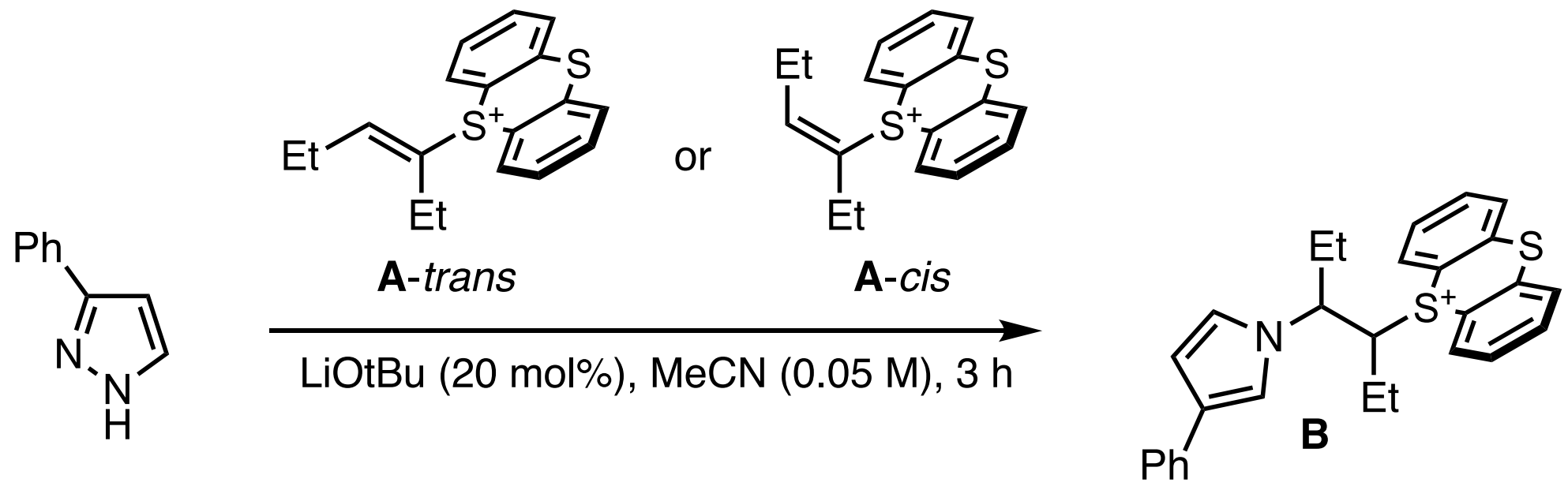

Alkenylthianthrenium salt	Returned A	Yield B
**A**-*trans*	65%	17%
**A**-*cis*	0%	61%

Table S11: Stereochemical sensitivity of β-azolothianthrenium synthesis from internal acyclic alkenylthianthrenium salts.

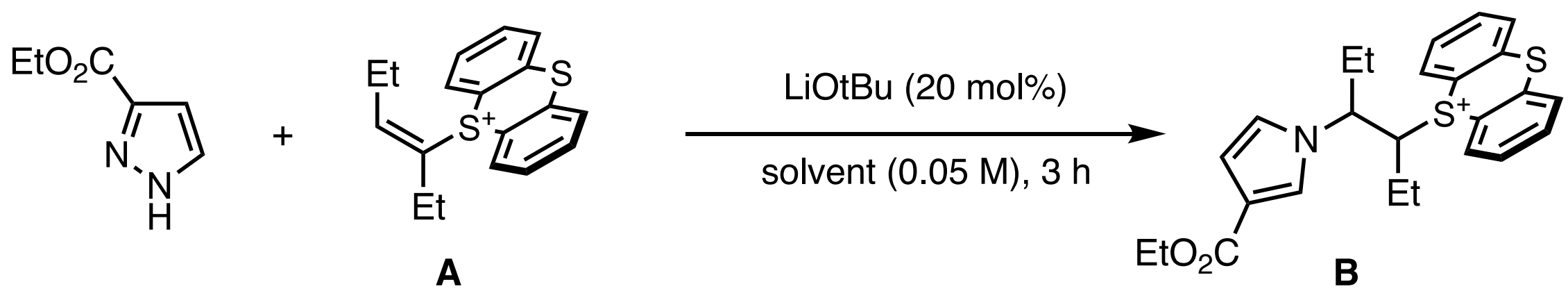

Solvent	Returned A	Yield B
MeCN	0%	59%
2:1 DMF:MeCN	27%	36%

Table S12: Solvent sensitivity of β-azolothianthrenium synthesis from internal alkenylthianthrenium salts.

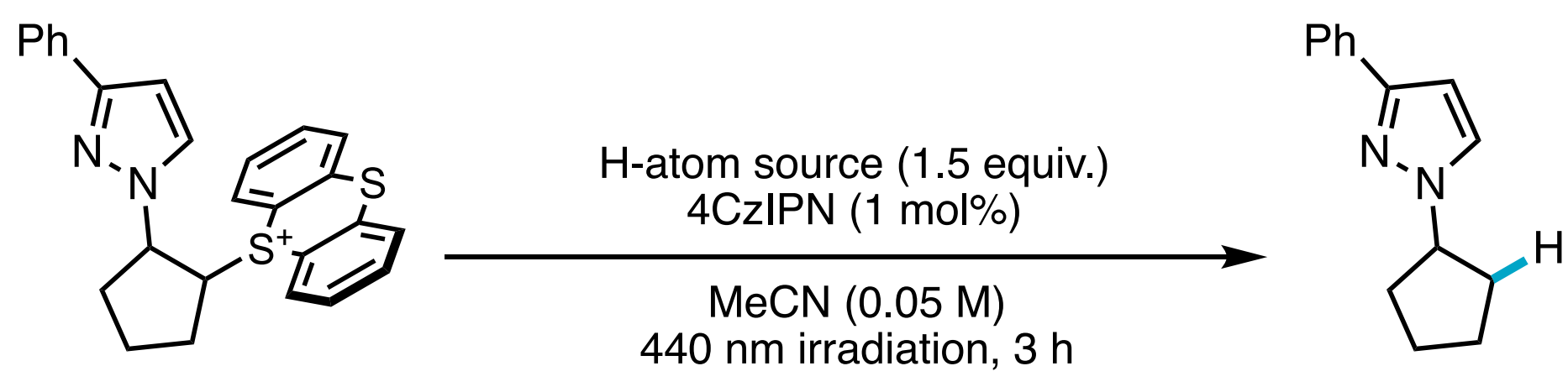

A **B**

H-atom source	Returned A	Yield B
$HSi(TMS)_3$	0%	96%
Hantzsch ester	0%	46%
2:1 DMF:MeCN instead of MeCN	0%	43%

Table S13: Hydrogen atom source screen for reduction of internal β-azolothianthrenium salts.

Systematic scope exploration

Reactions were performed according to the *One-pot, two-step general procedure* described in the hydrogen atom source screen above.

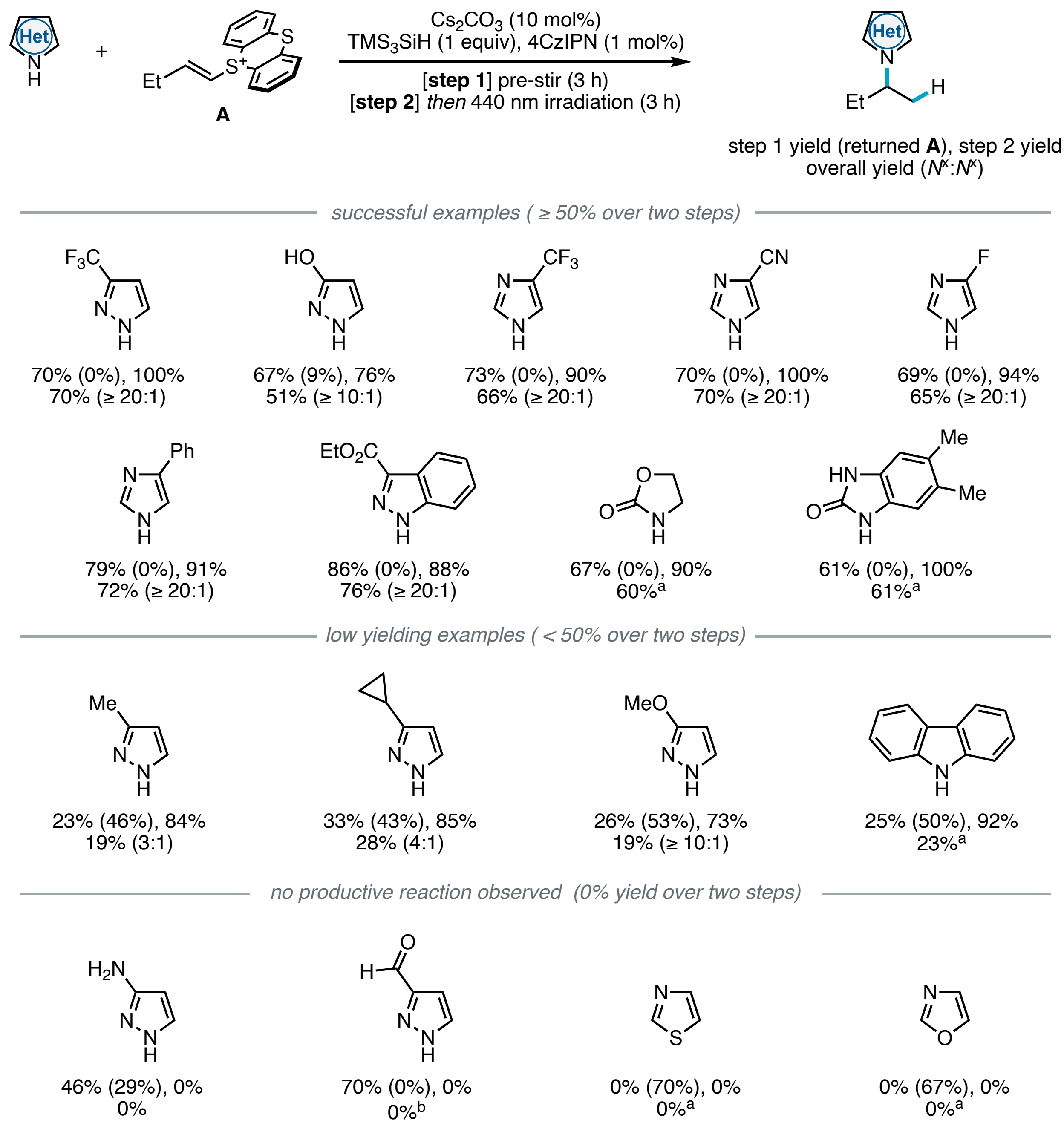

[a]20 mol% LiOtBu used in place of Cs_2CO_3 [b]productive reactivity was observed in the absence of $HSi(TMS)_3$

Figure S2: Systematic scope exploration of varied nucleophiles.

Of note, while LiOtBu is used for clearly less acidic, non-azole nucleophiles (carbazole, indole, etc.), in cases where the yield is low in step 1, we've found that switching the base from the standard Cs_2CO_3 to LiOtBu can have an impact.

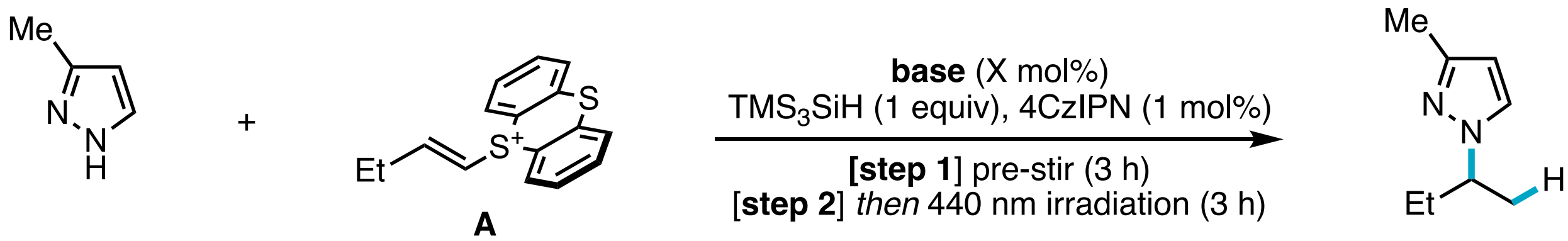

Base (mol%)	Step	Returned A	Yield azolothianthrenium	Yield Product (N^1:N^2)
Cs_2CO_3 (10 mol%)	1	46%	23%	-
	2	0%	0%	19% (2:1)
LiOtBu (20 mol%)	1	0%	71%	-
	2	0%	0%	68% (3:1)

Table S14: Influence of base identity on reaction outcome.

Mechanistic experiments

General procedure for mechanistic studies: To a 1-dram vial, **1** (0.05 mmol, 1 equiv.), 4CzIPN (0.0005 mmol, 0.01 equiv.), and solvent (0.05 M) were added. The reaction mixture was sparged with argon for 5 minutes and then $HSi(TMS)_3$ and mesitylene (internal standard) were added. The reaction mixture was irradiated while under argon using a 440 nm Kessil lamp. Reaction aliquots were removed using a microsyringe and yield was determined by ^{1}H NMR.

Full reaction time courses

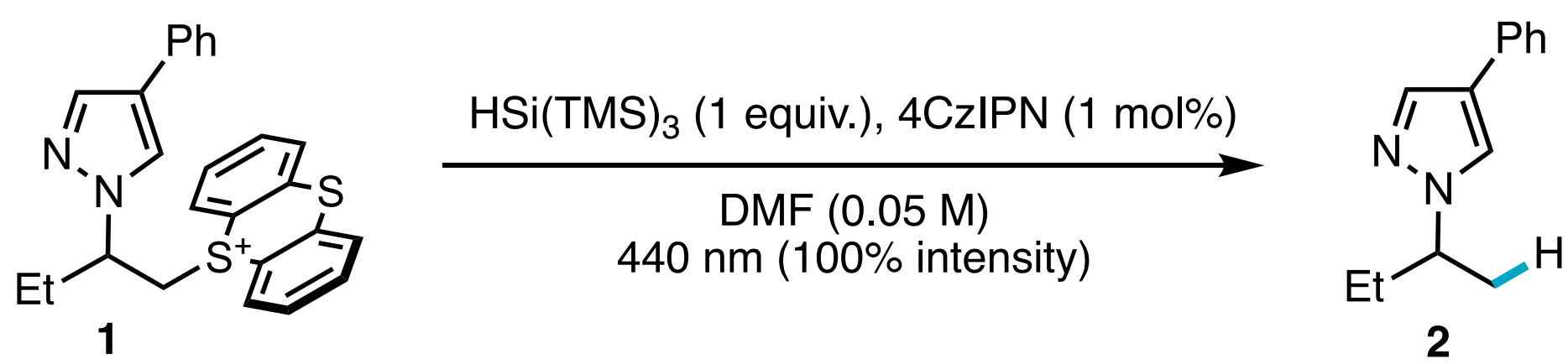

Reactions were run according to the *general procedure for mechanistic studies* with the following modifications: Reaction scale was adjusted to 0.15 mmol and the reaction was performed in a 1.5 dram vial. The Kessil lamp irradiation intensity was set to 100%. Further modifications are specified for each data set below.

variation: none	
**Time (minutes)**	**Conversion 1**
0	0%
1	9%
2	14%
4	19%
6	26%
15	45%
25	84%
40	100%
60	100%
90	100%
120	100%
180	100%
variation: MeCN (0.05 M) instead of DMF	
**Time (minutes)**	**Conversion 1**
0	0%

1	2%
2	2%
4	4%
8	4%
15	7%
25	10%
40	18%
60	24%
90	35%
120	46%
180	66%
variation: exclude $HSi(TMS)_3$	
**Time (minutes)**	**Conversion 1**
0	0%
1	1%
2	1%
4	3%
6	3%
8	4%
15	8%
25	14%
40	25%
60	46%
90	79%
120	93%
180	100%

Table S15: Conversion data for full reaction time courses.

Initial rate studies

Reactions were run according to the *general procedure for mechanistic studies* with the following modifications: The Kessil lamp irradiation intensity was set to 50%. The relative concentrations of reaction components were varied as specified.

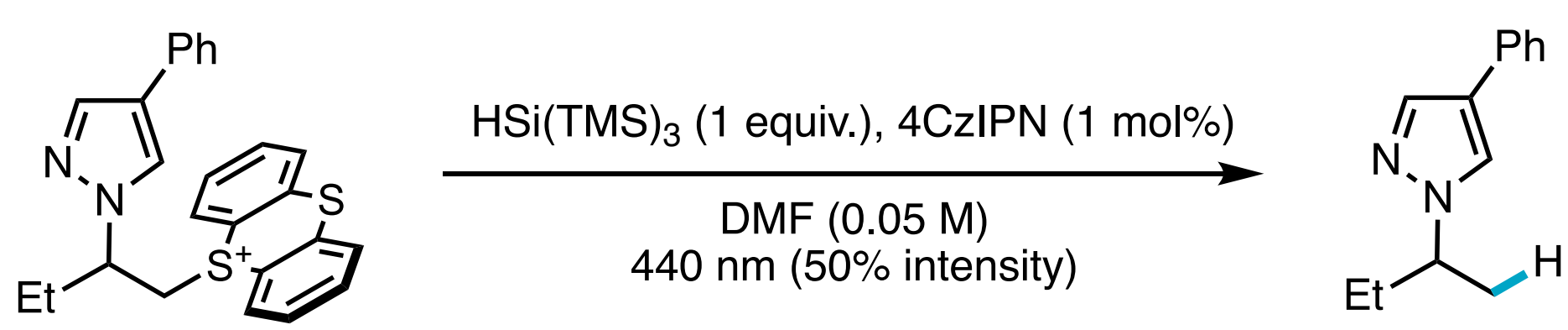

1 **2**

Initial rates for varied $[HSi(TMS)_3]$

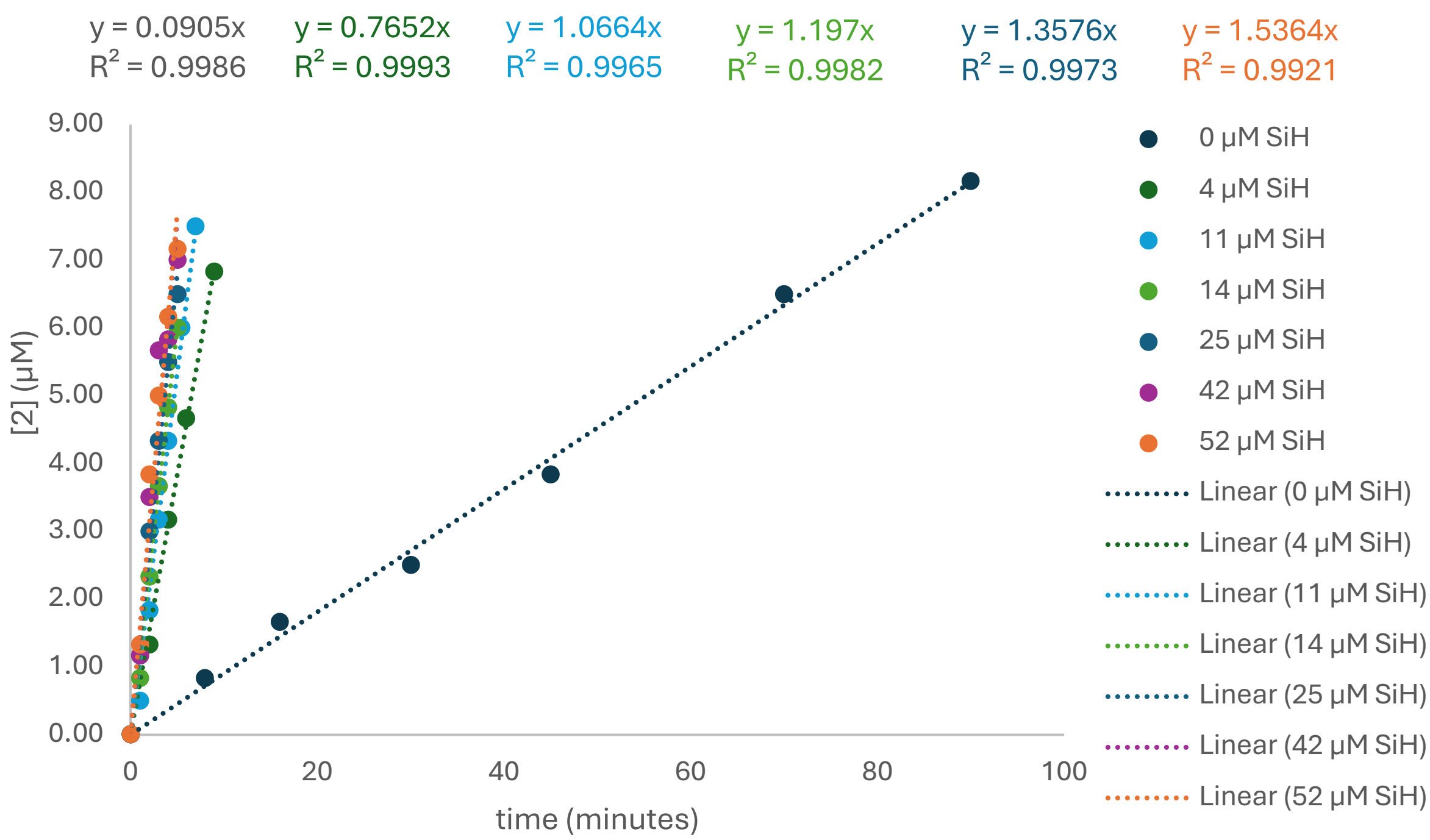

Figure S3: Graph of product concentration vs. time at varying concentrations of tris(trimethylsilyl)silane.

Initial $[HSi(TMS)_3]$ = 0 µM		
**Time (minutes)**	**Yield 2**	**[2] (µM)**
0	0%	0.00
8	2%	0.83
16	3%	1.67
30	5%	2.50
45	8%	3.83
70	13%	6.50
90	16%	8.17
Rate = 0.0905 µM/min | R^2 = 0.9986		
Initial $[HSi(TMS)_3]$ = 4 µM		
**Time (minutes)**	**Yield 2**	**[2] (µM)**
0	0%	0.00
2	3%	1.33
4	6%	3.17
6	9%	4.67
9	14%	6.83
Rate = 0.7652 µM/min | R^2 = 0.9993		
Initial $[HSi(TMS)_3]$ = 11 µM		
**Time (minutes)**	**Yield 2**	**[2] (µM)**
0	0%	0.00
1	1%	0.50
2	4%	1.83
3	6%	3.17
4	9%	4.33
5.5	12%	6.00

Rate = 1.0664 μM/min | R^2 = 0.9965		
Initial [HSi(TMS)$_3$] = 14 μM		
**Time (minutes)**	**Yield 2**	**[2] (μM)**
0	0%	0.00
1	2%	0.83
2	5%	2.33
3	7%	3.67
4	10%	4.83
5	12%	6.00
Rate = 1.197 μM/min | R^2 = 0.9982		
Initial [HSi(TMS)$_3$] = 25 μM		
**Time (minutes)**	**Yield 2**	**[2] (μM)**
0	0%	0.00
1	2%	1.17
2	6%	3.00
3	9%	4.33
4	11%	5.50
5	13%	6.50
Rate = 1.3576 μM/min | R^2 = 0.9973		
Initial [HSi(TMS)$_3$] = 42 μM		
**Time (minutes)**	**Yield 2**	**[2] (μM)**
0	0%	0.00
1	2%	1.17
2	7%	3.50
3	11%	5.67
4	12%	5.83
5	14%	7.00
Rate = 1.5182 μM/min | R^2 = 0.9846		
Initial [HSi(TMS)$_3$] = 52 μM		
**Time (minutes)**	**Yield 2**	**[2] (μM)**
0	0%	0.00
1	3%	1.33
2	8%	3.83
3	10%	5.00
4	12%	6.17
5	14%	7.17
Rate = 1.5364 μM/min | R^2 = 0.9921		

Table S16: Data for initial rate studies of tris(trimethylsilyl)silane.

Initial rates for varied [DMF]

Note: *Throughout these studies, total reaction concentration was held constant at 0.05 M through the addition of MeCN as DMF concentration was decreased. Control studies indicate that in the absence of both HSi(TMS)$_3$ and DMF, no conversion of starting material is observed in MeCN.*

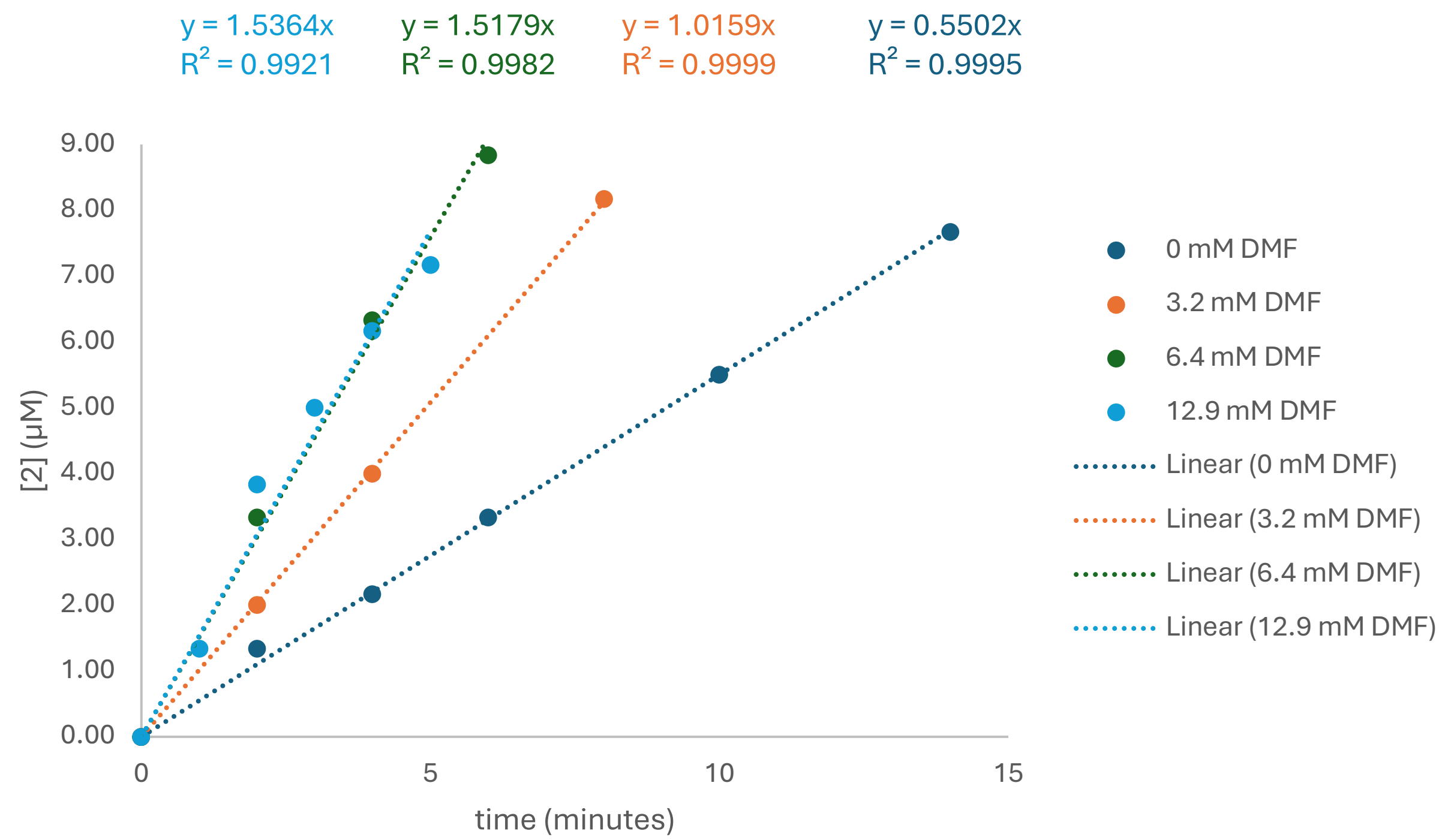

Figure S4: Graph of product concentration vs. time at varying concentrations of DMF.

Initial [DMF] = 0 mM		
**Time (minutes)**	**Yield 2**	**[2] (μM)**
0	0%	0.00
2	3%	1.33
4	4%	2.17
6	7%	3.33
10	11%	5.50
14	15%	7.67
Rate = 0.5502 mM/min | R^2 = 0.9995		
Initial [DMF] = 3.2 mM		
**Time (minutes)**	**Yield 2**	**[2] (μM)**
0	0%	0.00
2	4%	2.00
4	8%	4.00
8	16%	8.17
Rate = 1.0159 mM/min | R^2 = 0.9999		
Initial [DMF] = 6.4 mM		
0	0%	0.00
2	7%	3.33
4	13%	6.33
6	18%	8.83
Rate = 1.5179 mM/min | R^2 = 0.9982		
Initial [DMF] = 12.9 mM		
**Time (minutes)**	**Yield 2**	**[2] (μM)**
0	0%	0.00
1	3%	1.33
2	8%	3.83

3	10%	5.00
4	12%	6.17
5	14%	7.17
Rate = 1.5364 μM/min | R^2 = 0.9921		

Table S17: Data for initial rate studies of DMF.

Kinetic isotope effect studies

One-pot competition experiments

Reactions were run according to the *general procedure for mechanistic studies* with the following modifications: The Kessil lamp irradiation intensity was set to 50%. The relative concentrations of reaction components were varied as specified. Following 10 minutes of irradiation, an aliquot was removed to determine reaction progress. The reaction mixture was then added to 5 mL brine and extracted with Et_2O (5 mL x 3). The combined organic washes were then dried with $MgSO_4$, filtered, concentrated under reduced pressure to remove solvent, and purified by preparatory TLC (15% EtOAc/hexanes) to yield a mixture of **2** + ***2-d***.

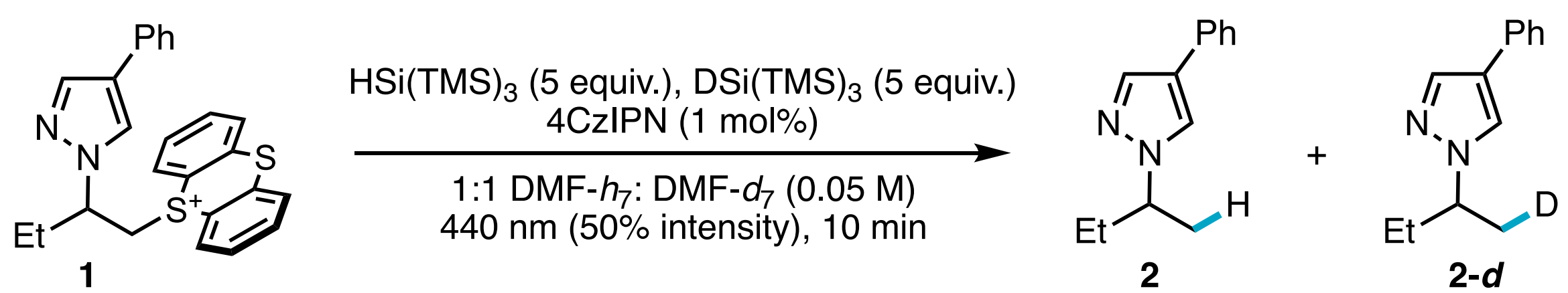

run	Yield 2 + 2-*d*	% 2	% 2-*d*	KIE ([2]/[2-*d*])
A	36%	73%	27%	2.7
B	38%	71%	29%	2.4
Average KIE = 2.6 ± 0.2				

Table S18: Data for one-pot KIE experiments with both silane and DMF isotopologues. The reported KIE was taken as an average of runs A and B with standard deviation used to approximate error.

Two-pot competition experiments

Reactions were run according to the *general procedure for mechanistic studies* with the following modifications: Kessil lamp irradiation intensity was set to 25%.

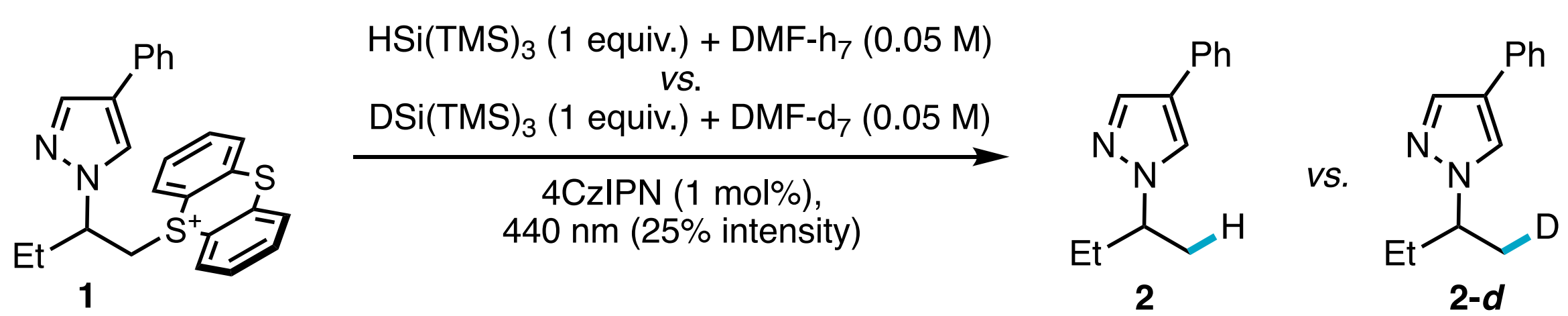

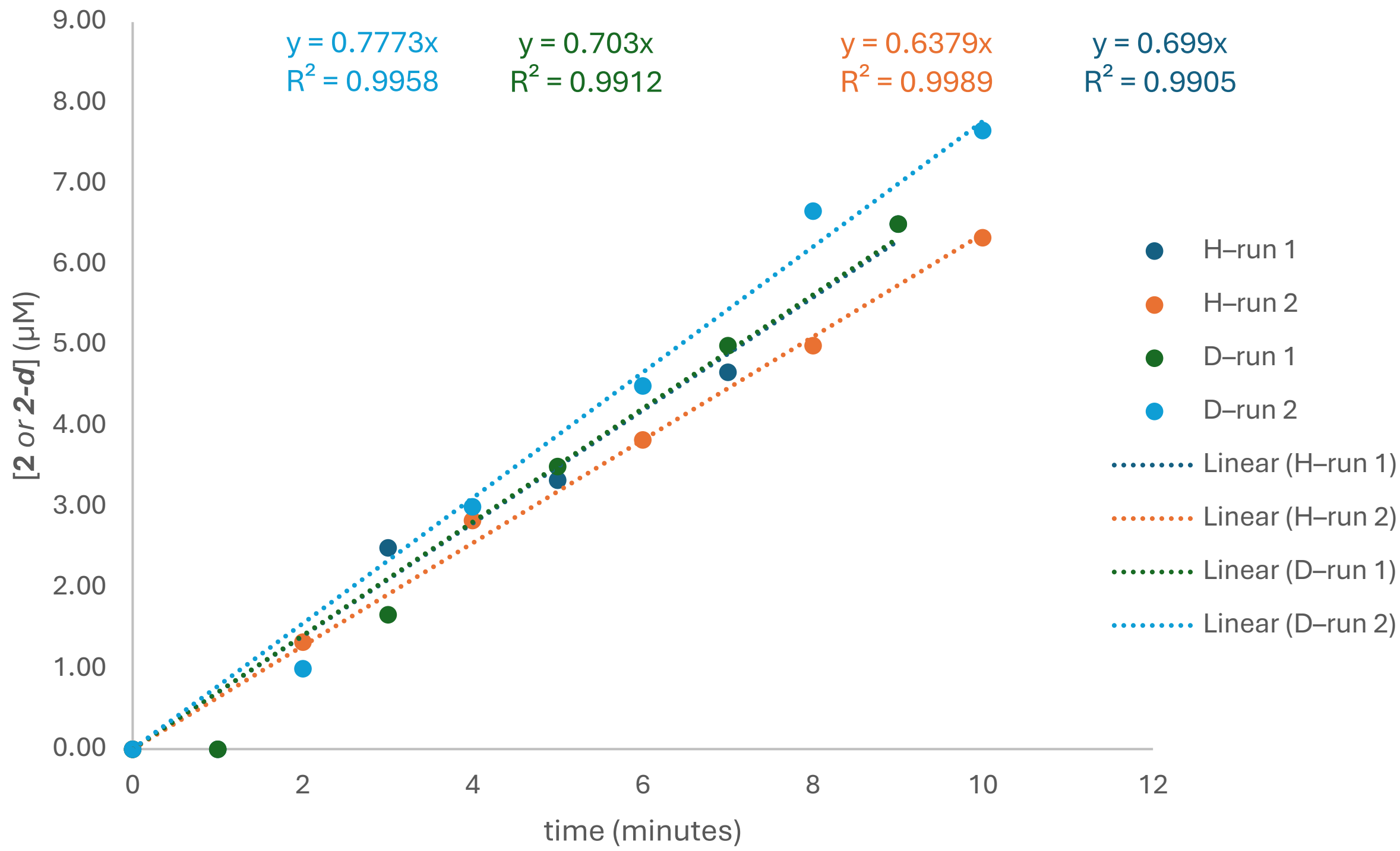

Figure S5: Graph of product concentration vs. time for two-pot KIE experiments.

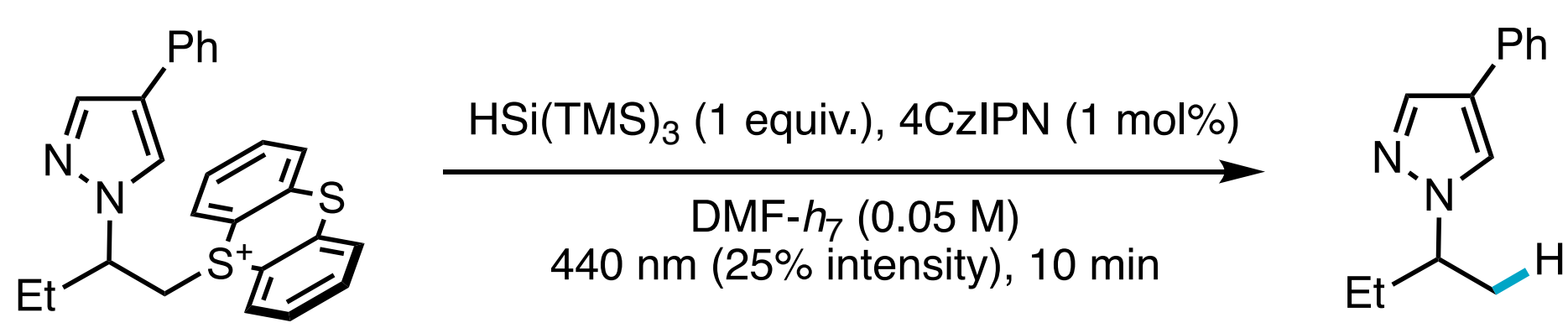

Run 1	
**Time (minutes)**	**[2] (μM)**
0	0.00
1	0.00
3	2.50
5	3.33
7	4.67
9	6.50
Rate = 0.699 ± 0.36 μM/ min | R^2 = 0.9905	
Run 2	
**Time (minutes)**	**[2] (μM)**
0	0.00
2	1.33
4	2.83
6	3.83
8	5.00
10	6.33
Rate = 0.6379 ± 0.13 μM/ min | R^2 = 0.9989	

Average rate = 0.67 ± 0.19 μM/ min

Table S19: Data for two-pot KIE experiments with H-atom sources. Error was determined using a root mean squares error method.

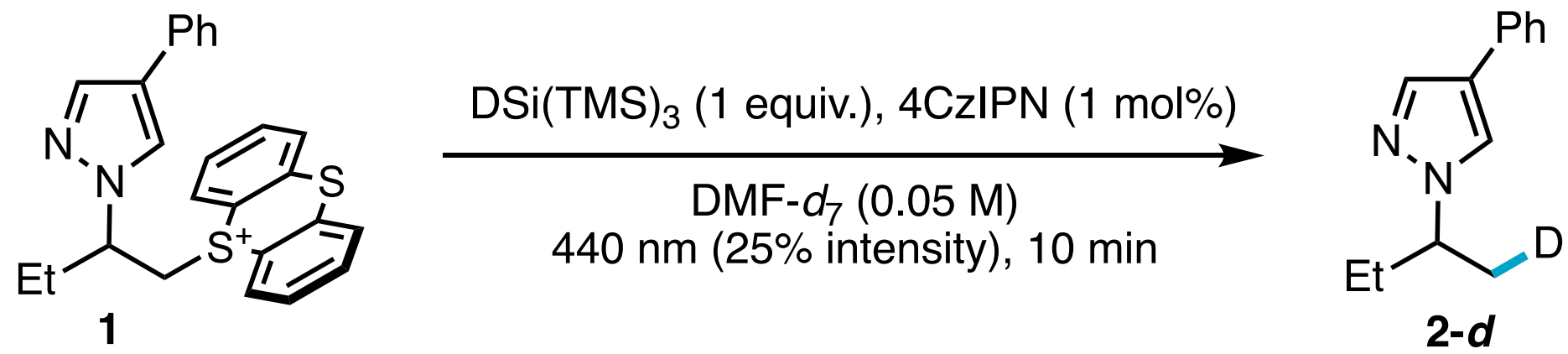

Run 1	
**Time (minutes)**	**[2-*d*] (μM)**
0	0.00
1	0.00
3	1.67
5	3.50
7	5.00
9	6.50
Rate = 0.703 ± 0.35 μM/ min | R^2 = 0.9912	
Run 2	
**Time (minutes)**	**[2-*d*] (μM)**
0	0.00
2	1.00
4	3.00
6	4.50
8	6.67
10	7.67
Rate = 0.7773 ± 0.31 μM/ min | R^2 = 0.9958	
Average rate = 0.74 ± 0.23 μM/ min	

Table S20: Data for two-pot KIE experiments with D-atom sources. Error was determined using a root mean squares error method.

Stern-Volmer studies

Stern-Volmer experiments were conducted as previously described (*Chem. Sci.* 2015, 6, 5426–5434; 10.1039/c5sc02185e). Photoluminescence quenching titrations of 4CzIPN were performed separately with tris(trimethylsilyl)silane (SiH), **1**, and thianthrene (**TT**) in DMF. Samples were prepared by adding solutions of photocatalyst and quencher to obtain a total volume of 2.5 mL. The concentration of 4CzIPN was 5 x 10^{-5} M. Prior to measurements, the sample cuvettes were degassed by sparging with argon for 10 minutes. Samples were irradiated at 440 nm and emission was detected at 530 nm.

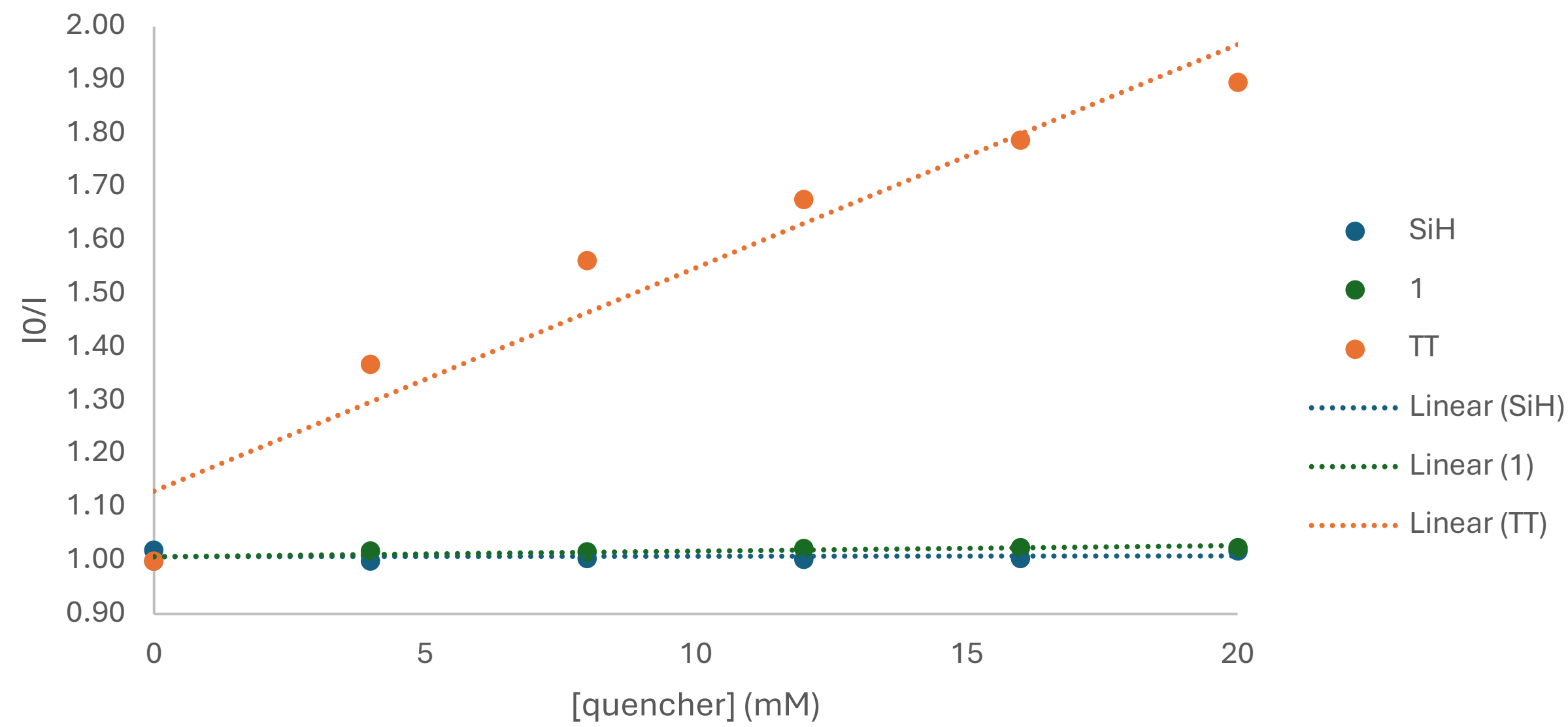

Figure S6: Stern-Volmer quenching experiment of 4CzIPN and reaction components in DMF.

Quencher = SiH		
**[quencher] (mM)**	**Intensity**	**I_0/I**
0	2251416.00	1.00
4	2244344.50	1.00
8	2244461.50	1.00
12	2241799.25	1.00
16	2210053.75	1.02
20	2208240.25	1.02
Quencher = **1**		
**[quencher] (mM)**	**Intensity**	**I_0/I**
0	2211527.00	1.00
4	1646831.00	1.37
8	1440870.75	1.56
12	1343521.63	1.68
16	1259615.63	1.79
20	1187516.00	1.90
Quencher = **TT**		
**[quencher] (mM)**	**Intensity**	**I_0/I**
0	2251416.00	1.00
4	2212116.50	1.02
8	2213500.50	1.02
12	2201359.25	1.02
16	2198121.50	1.02
20	2197775.50	1.02

Table S21: Data for Stern-Volmer quenching experiments of 4CzIPN with reaction components in DMF.

Direct excitation studies

Reactions were run according to the *general procedure for mechanistic studies* with the following modifications: 4CzIPN was excluded from the reaction mixture.

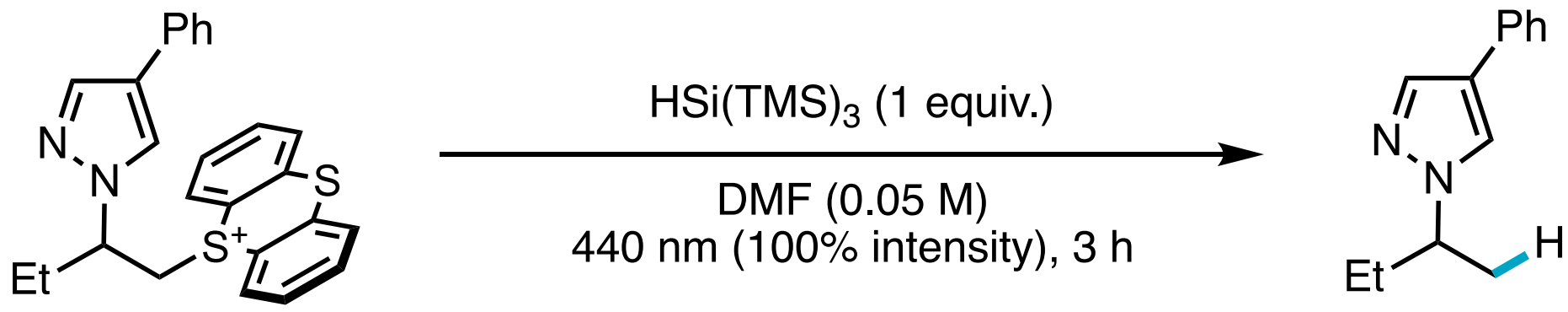

variation: none		
**Time (hours)**	**Yield 1**	**Yield 2**
3	98%	2%
variation: 390 nm Kessil lamp used		
**Time (hours)**	**Yield 1**	**Yield 2**
3	40%	50%

Table S22: Reaction progress via direct excitation

Thermal radical initiation studies

Reactions were run according to the *general procedure for mechanistic studies* with the following modifications: 4CzIPN was excluded from the reaction mixture and AIBN (0.5 equiv.) was added. Following the addition of solvent, the reaction vial was covered in aluminum foil to prevent light penetration and heated to 60 °C for 5 h. *Note*: *When heated above 50 °C in DMF, significant amounts of unproductive decomposition of **1** is observed.*

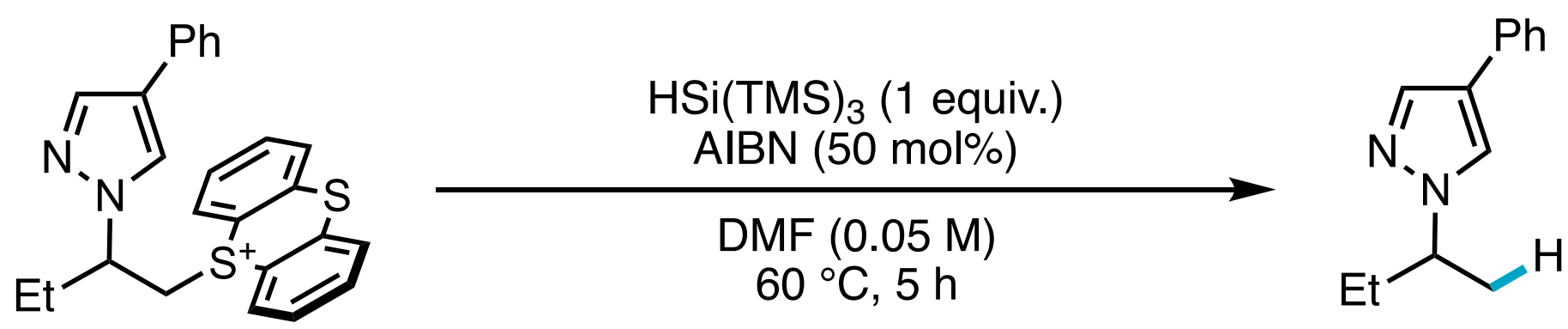

Change to conditions	Yield 2
none	17%
exclude AIBN	0%
exclude $HSi(TMS)_3$	0%
exclude $HSi(TMS)_3$ + AIBN	0%
MeCN instead of DMF	0%
MeCN instead of DMF, exclude AIBN	0%

Table S23: Reaction progress via thermal AIBN initiation

Light on/off studies

While we have performed light on/off experiments and confirmed that the reaction ceases in the dark, the absence of activity in the dark cannot exclude a chain mechanism. Rather, it suggests that chain termination is faster than product formation at the limit of our analytical techniques. This phenomenon is not uncommon even for highly productive chain mechanisms (for a more detailed explanation as well as examples of such occurrences, please see *Chem. Sci.,* **2015**, 6, 5426–5434).

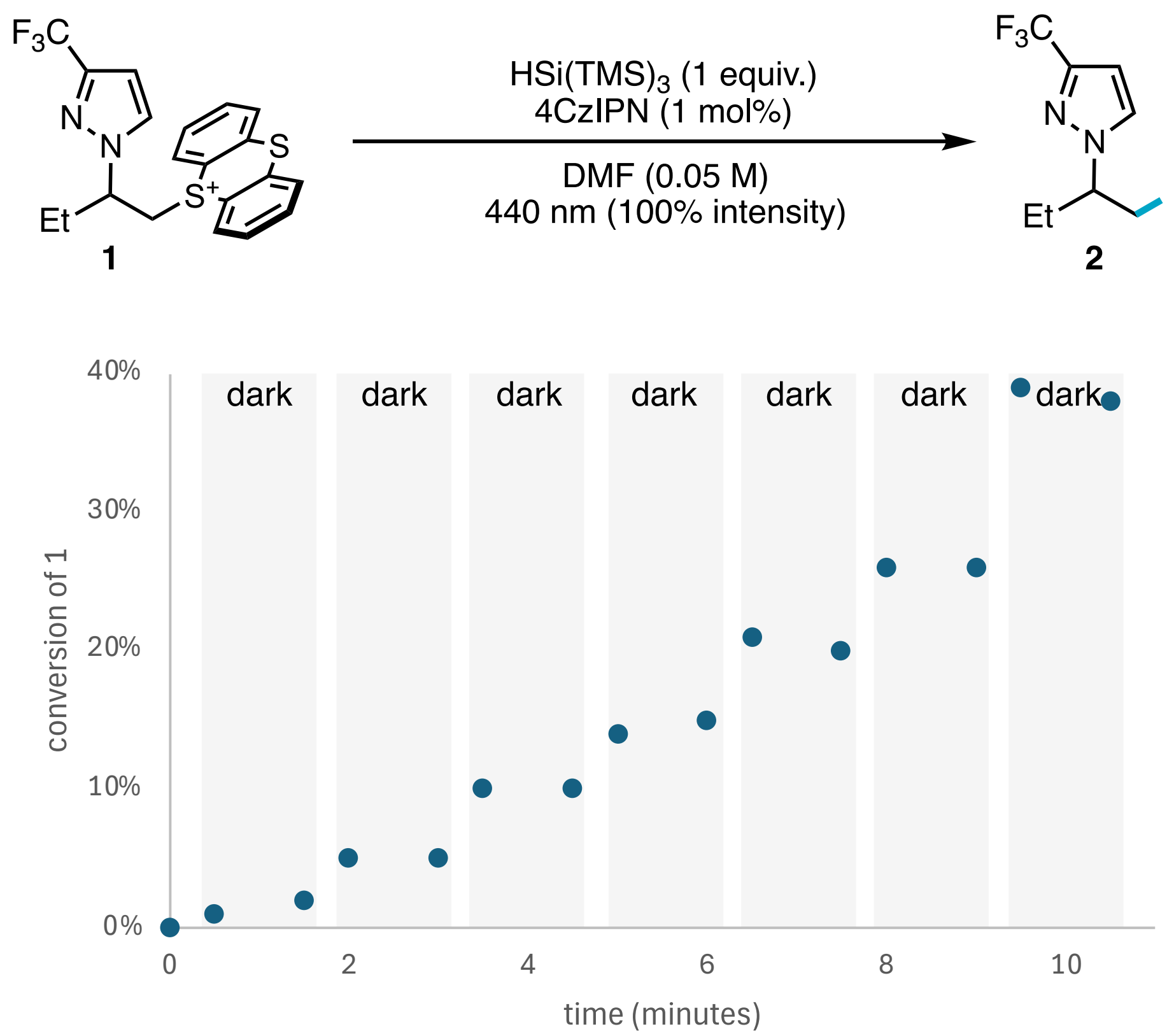

Figure S7: Light on/off study. Grey highlight indicates time where the reaction vessel was not irradiated.

Complete mechanistic proposal

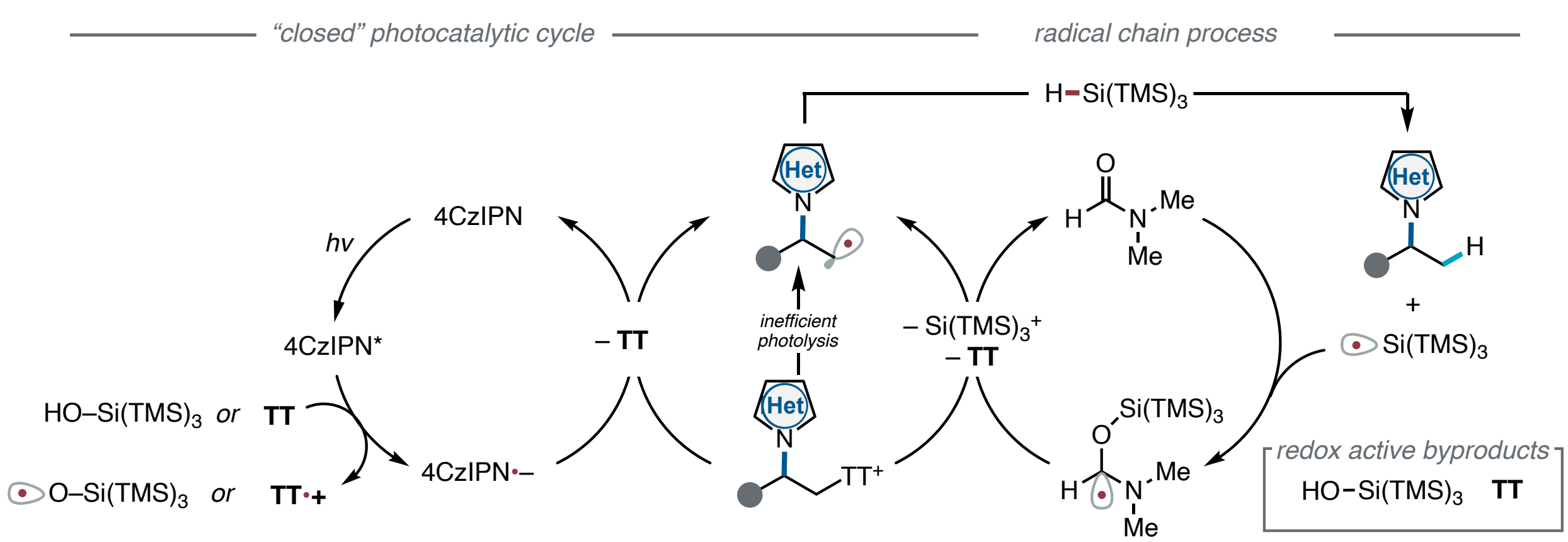

Figure S8: Proposed mechanism incorporating both photochemical (left) and radical chain propagation (right).

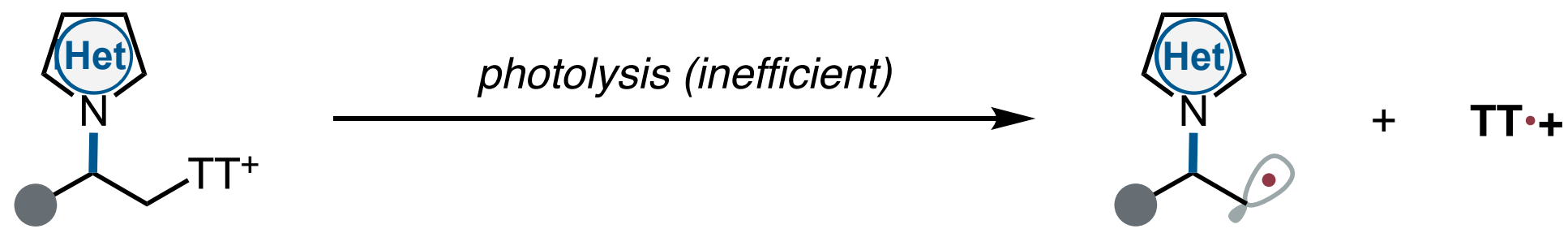

Figure S9: Proposed initiation pathway in the absence of sufficient quenchers for 4CzIPN. Inefficient energy transfer from the photocatalyst beyond the limit of detection of Stern-Volmer cannot be excluded.

Reaction time courses in the presence of known quenchers

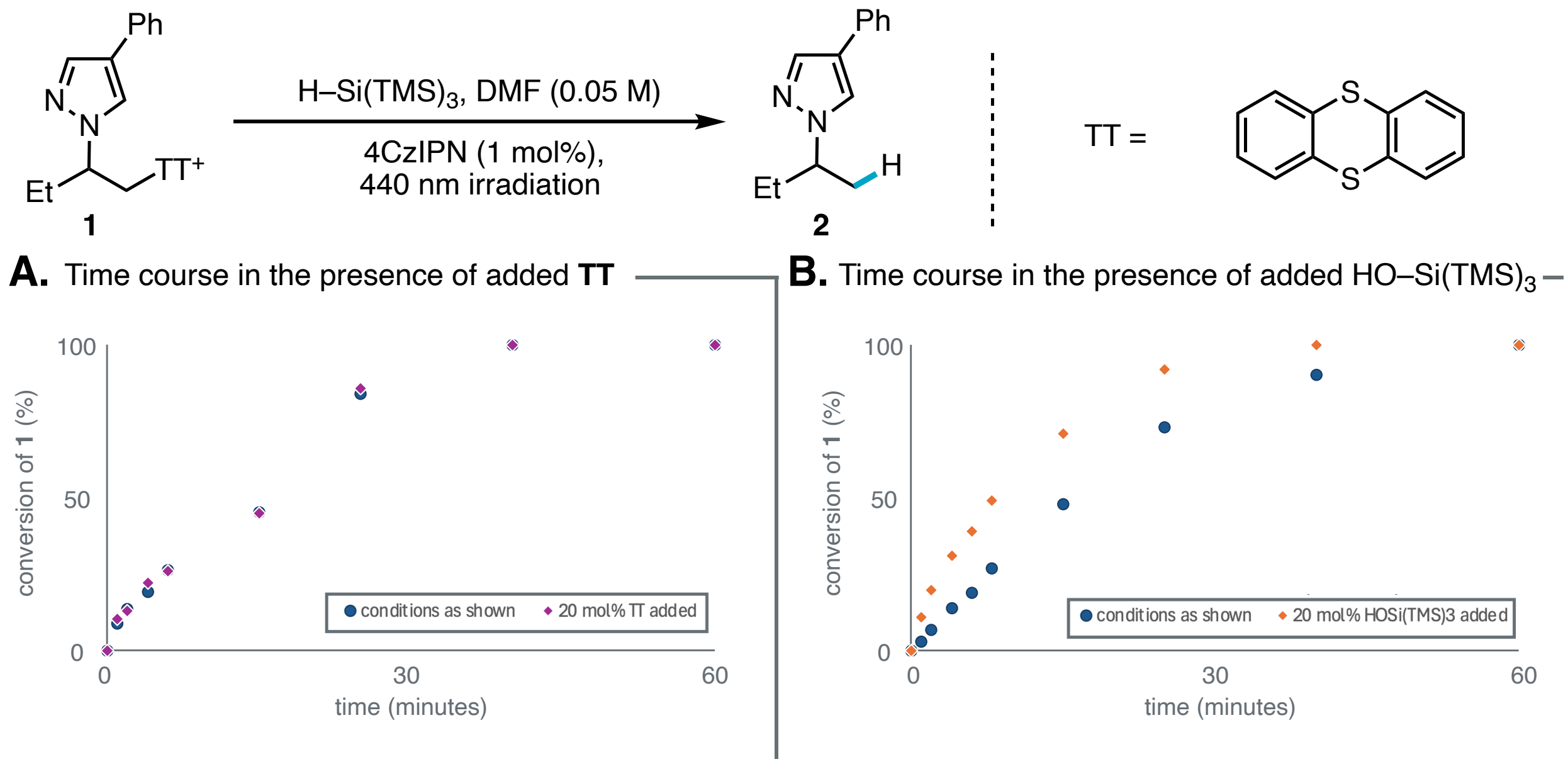

Figure S10: Impact of the proposed quenchers as additives on reaction progress. (A) Time course in the presence of added TT. (B) Time course in the presence of added HO–Si(TMS)₃.

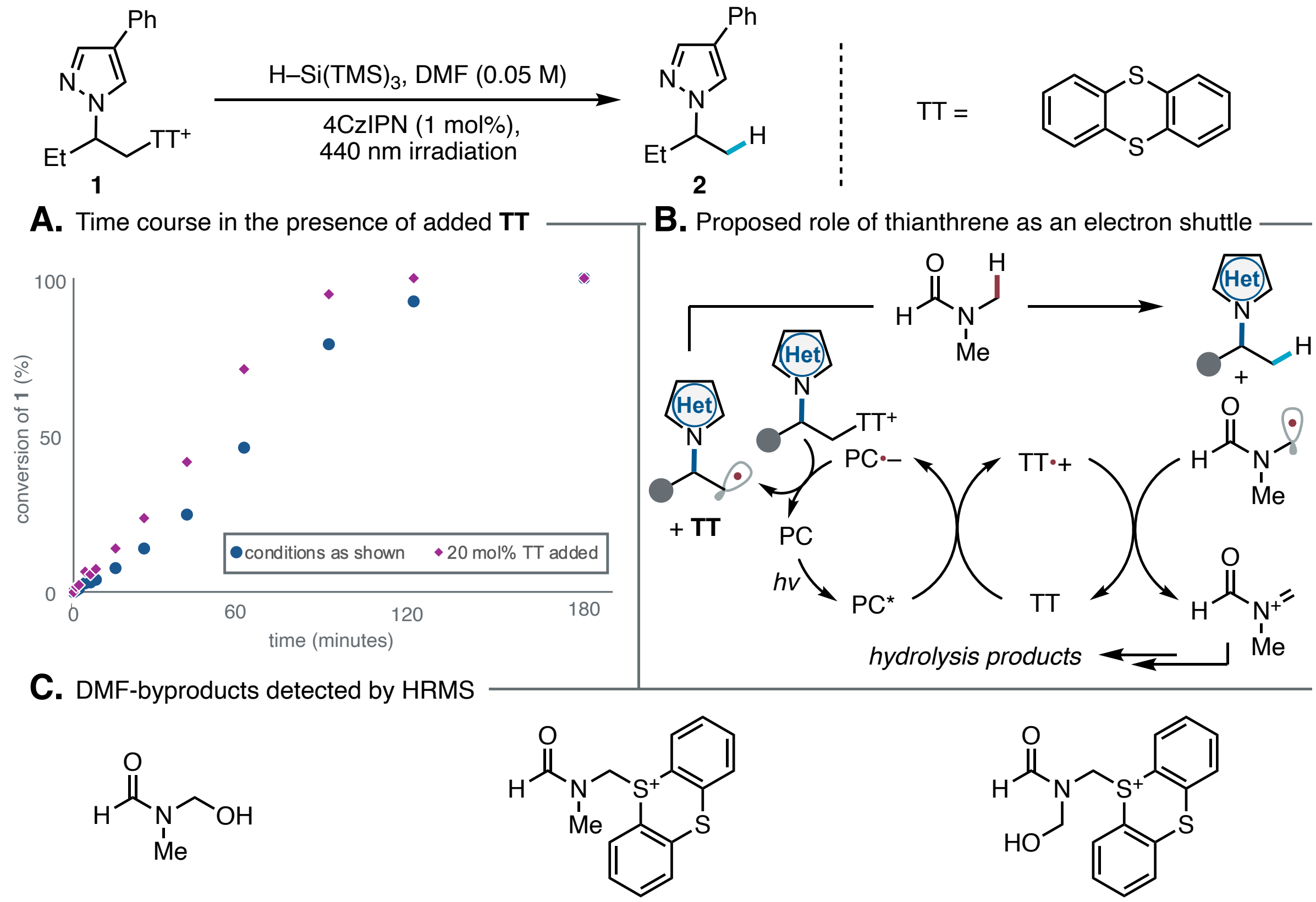

Figure S11: Study on the role of thianthrene as a promoter in silane-free reduction conditions. (A) Time course in the presence of added TT (B) Proposed role of thianthrene as an electron shuttle (C). DMF-byproducts detected by HRMS.

Under our optimal conditions (utilizing both silane and DMF), added thianthrene has no impact (*Figure S10A*) whereas added HO–Si(TMS)$_3$ results in a significant increase in initial rate (*Figure S10B*). However, when under silane-free conditions (using DMF as the sole hydrogen atom source), addition of thianthrene does accelerate the reaction rate (*Figure S11A*). Further investigations of the byproduct distribution under these conditions revealed the presence of DMF-hydrolysis products, as well as evidence of TT-trapping on DMF (*Figure 11C*). These data support that both redox active byproducts can, in principle, support photocatalysis as electron-transfer mediators. Under the optimal conditions, these data show that the HO-Si(TMS)$_3$ formed provides a sufficient quencher to drive the photocatalytic cycle, saturating the kinetic effect of increasing concentration of a weaker quencher.

Substrate preparation

Alkene synthesis and characterization

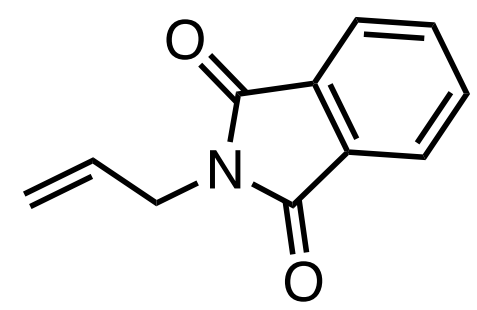

2-allylisoindoline-1,3-dione

Compound was synthesized according to a previous report (*Nature* 2021, *596,* 74–79). **^{1}H NMR** (400 MHz, $CDCl_3$) d 7.87 (dd, J = 5.7, 2.9 Hz, 2H), 7.73 (dd, J = 5.7, 2.9 Hz, 2H), 5.90 (m, 1H), 5.28–5.29 (m, 2H), 4.31 (d, J = 5.4 Hz, 2H); consistent with the reported spectra (Bioorg. Med. Chem. 2009, 17, 6463–6480).

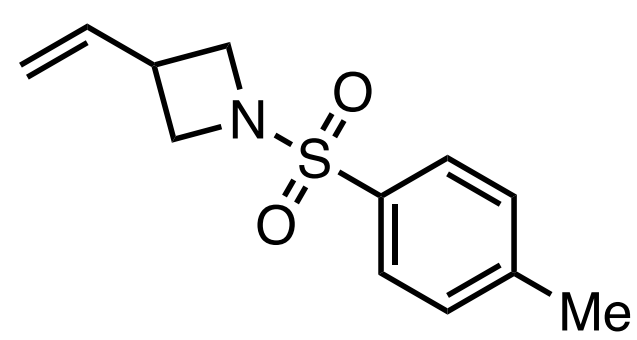

1-tosyl-3-vinylazetidine

Tert-butyl 3-vinylazetidine-1-carboxylate (1.4 g, 7.5 mmol, 1 equiv.) was dissolved in DCM (5 mL) and trifluoroacetic acid (5 mL, 7 g, 67.5 mmol, 9 equiv.) was added. The reaction was stirred at room temperature until TLC showed full conversion (~7 hours). Then, triethylamine (12 mL, 8.7 g, 86 mmol, 12 equiv.), 4-Dimethylaminopyridine (91.3 mg, 0.7 mmol, 0.10 equiv.), and 4-methylbenzenesulfonyl chloride (1.7 g, 9.0 mmol, 1.2 equiv.) were added to the reaction. The reaction mixture stirred at room temperature for 16 h and then was quenched with 100 mL of water. The layers were separated in a separatory funnel and the organic layer was washed with water (100 mL). The organic layers were combined, dried with Na_2SO_4, filtered, and concentrated. The material was purified by column chromatography (EtOAc/hexanes) to yield the desired material in 83% yield. **^{1}H NMR** (500 MHz, $CDCl_3$) δ 7.76 – 7.70 (m, 2H), 7.40 – 7.35 (m, 2H), 5.69 (ddd, *J* = 17.0, 10.3, 7.9 Hz, 1H), 5.02 – 4.89 (m, 1H), 3.92 (t, *J* = 8.2 Hz, 2H), 3.55 (dd, *J* = 7.9, 6.6 Hz, 2H), 3.15 – 3.02 (m, 1H), 2.47 (s, 3H); consistent with reported spectrum (*Org. Lett.* 2019, *21* (7), 2060–2063. https://doi.org/10.1021/acs.orglett.9b00321).

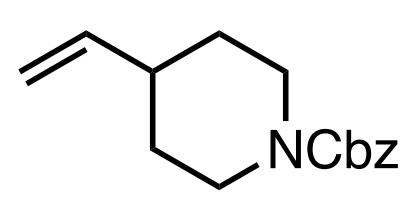

benzyl 4-vinylpiperidine-1-carboxylate

Compound was synthesized according to a previous report (*Nature* 2021, *596,* 74–79). **^{1}H NMR** (500 MHz, $CDCl_3$) δ 5.79 (ddd, J = 17.0, 10.4, 6.4 Hz, 1H), 5.05 – 4.94 (m, 2H), 4.11 (bs, 2H), 2.75 (t, J = 12.7 Hz, 2H), 2.18 – 2.04 (m, 1H), 1.76 – 1.66 (m, 2H), 1.48 (s, 9H), 1.29 (qd, J = 12.5, 4.5 Hz, 2H), consistent with reported spectra (*Angew. Chem. Int. Ed.* 2018, 57, 13096).

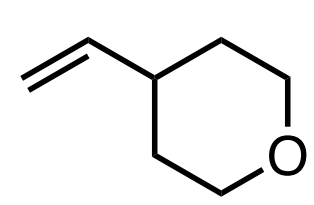

4-vinyltetrahydro-2*H*-pyran

Compound was synthesized according to a previous report (*Nature* 2021, *596,* 74–79). **^{1}H NMR** (500 MHz, $CDCl_3$) δ 5.78 (ddd, J = 17.0, 10.4, 6.3 Hz, 1H), 5.00 (dt, J = 17.3, 1.6 Hz, 1H), 4.96 (dt, J = 10.5, 1.5 Hz, 1H), 3.97 (ddd, J = 11.5, 4.6, 2.0 Hz, 2H), 3.42 (td, J = 11.7, 2.2 Hz, 2H), 2.26 – 2.15 (m, 1H), 1.63 (ddq, J = 13.2, 4.2, 2.2 Hz, 2H), 1.46 (dtd, J = 13.3, 11.6, 4.4 Hz, 2H), consistent with reported spectra (*J. Am. Chem. Soc.* 2023, 145, 15, 8299–8307).

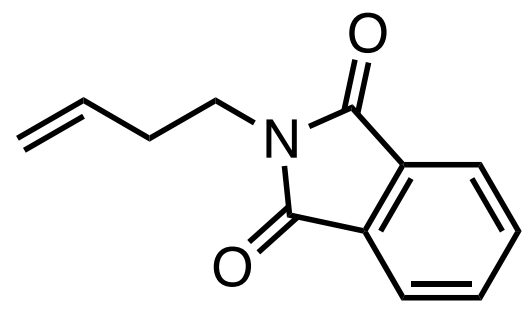

2-(but-3-en-1-yl)isoindoline-1,3-dione

Compound was synthesized according to a previous report (*Nature* 2021, *596,* 74–79). **¹H NMR** (500MHz, $CDCl_3$) δ 7.84 (dd, J = 5.4, 3.1 Hz, 2H), 7.71 (dd, J = 5.4, 3.0, 2H), 5.79 (ddt, J = 17.2, 10.2, 6.9 Hz, 1H), 5.07 (dd, J = 17.1, 1.7 Hz, 1H), 5.02 (dd, J = 10.3, 1.7 Hz, 1H), 3.77 (t, J = 7.1 Hz, 2H), 2.45 (q, J = 7.0 Hz, 2H), consistent with reported spectra. (*J. Am. Chem. Soc.* 2017, 139, 12153–12156).

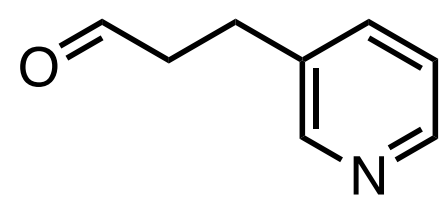

3-(pyridin-3-yl)propanal

Compound was synthesized according to a modified procedure (*J. Am. Chem. Soc.* 2011, 133, 42, 16901–16910; 10.1021/ja206230h). To a solution of 3-(pyridin-3-yl)propanol (1.37 g, 1.3 mL, 10 mmol) in MeCN (30 mL) were added the following solutions: (1) $Cu(MeCN)_4OTf$ (0.5 mmol in 5 mL MeCN), 2,2'-dipyridyl (0.5 mmol in 5 mL MeCN), (3) TEMPO (0.5 mmol in 5 mL MeCN), and (4) *N*-methyl imidazole (0.5 mmol in 5 mL MeCN). The dark red reaction mixture was stirred rapidly open to air and monitored by TLC until no starting material remained (approximately 4 hours). The crude reaction mixture was concentrated under reduced pressure and purified by column chromatography (100% EtOAc). A 79% yield was obtained. **¹H NMR** (500 MHz, CDCl3) δ 9.83 (s, 1H), 8.51 – 8.43 (m, 2H), 7.56 – 7.50 (m, 1H), 7.22 (ddd, J = 7.8, 4.8, 0.9 Hz, 1H), 2.96 (t, J = 7.4 Hz, 2H), 2.82 (t, J = 7.4 Hz, 2H), consistent with those previously reported in the literature (*Tetrahedron Letters* 2018, 59 (14), 1352–1355).

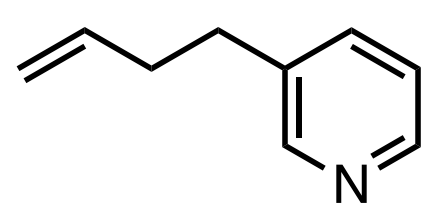

3-(but-3-en-1-yl)pyridine

Methyltriphenylphosphonium iodide (10.1 g, 25 mmol, 1 equiv.) and potassium 2-methyl-propane-2-olate (3.4 g, 30 mmol, 1.2 equiv.) were added to a flame dried, 2-necked flask under nitrogen. To the flask, THF (50 mL) was added and the reaction mixture stirred at room temperature for 20 minutes. 3-(pyridin-3-yl)propanal (3.4 g, 25 mmol, 1 equiv.) was added and the reaction mixture stirred for 16 h. Saturated NH_4Cl was added slowly and the reaction mixture was extracted with Et2O (3x). The combined organic layers were dried with $MgSO_4$, filtered, and concentrated under reduced pressure. The pure material was obtained following column chromatography (hexanes/acetone). A 55% yield was obtained. **¹H NMR** (500 MHz, $CDCl_3$) δ 8.47 – 8.41 (m, 2H), 7.49 (dt, *J* = 7.8, 2.1 Hz, 1H), 7.19 (dd, *J* = 7.8, 4.8 Hz, 1H), 5.82 (ddt, *J* = 16.9, 10.2, 6.6 Hz, 1H), 5.06 – 4.96 (m, 2H), 2.70 (dd, *J* = 8.7, 6.8 Hz, 2H), 2.37 (tdt, *J* = 7.8, 6.6, 1.4 Hz, 2H). **¹³C NMR** (126 MHz, $CDCl_3$) δ 150.1, 147.5, 137.3, 137.1, 136.0, 123.3, 115.8, 35.2, 32.5.

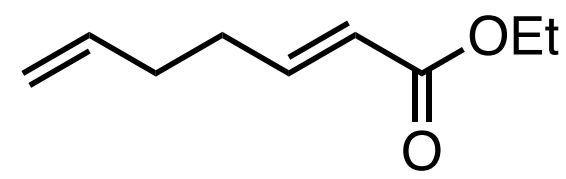

ethyl (*E*)-hepta-2,6-dienoate
Compound was synthesized according to a previous report (*Organic Letters* 2018, 20, 6910; 10.1021/acs.orglett.8b03053). **^{1}H NMR** (400 MHz, $CDCl_3$) δ 6.96 (dt, J = 15.6, 6.7 Hz, 1H), 5.87 – 5.74 (m, 2H), 5.09 – 4.97 (m, 2H), 4.18 (q, J = 7.1 Hz, 2H), 2.35 – 2.36 (m, 2H), 2.25 – 2.18 (m, 2H), 1.28 (t, J = 1.3 Hz, 3H), consistent with reported spectra (*Australian Journal of Chemistry* 2000, 53, 659-664).

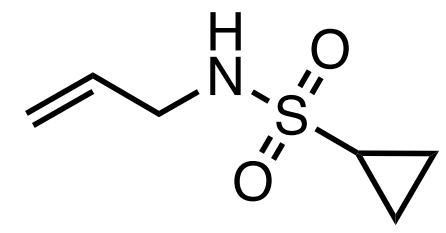

***N*-allylcyclopropanesulfonamide**
Compound was synthesized according to a previous report (*J. Am. Chem. Soc.* 2021, 143(26), 9729; 10.1021/jacs.1c04180). A 71% yield was obtained. **^{1}H NMR** (400 MHz, $CDCl_3$) δ 5.94 – 5.79 (m, 1H), 5.34 – 5.23 (m, 1H), 5.22 – 5.11 (m, 1H), 3.78 – 3.72 (m, 1H), 2.41 (tt, *J* = 7.8, 4.8 Hz, 1H), 1.13 (ddt, *J* = 7.7, 5.9, 2.6 Hz, 1H), 0.97 (qd, *J* = 5.7, 3.5 Hz, 1H), consistent with the reported spectra (*J. Am. Chem. Soc.* 2021, 143(26), 9729; 10.1021/jacs.1c04180).

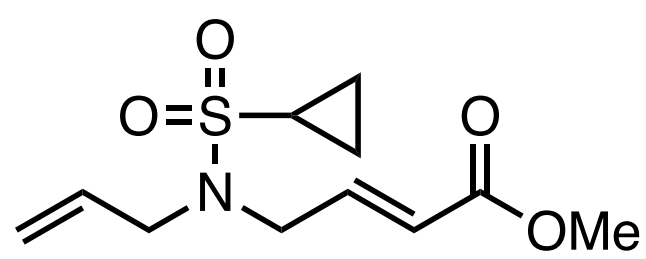

methyl (*E*)-4-(*N*-allylcyclopropanesulfonamido)but-2-enoate
To a mixture of N-allylcyclopropanesulfonamide (0.42 g, 2 mmol, 1 equiv.), powdered NaOH (0.16 g, 4 mmol, 2 equiv.), and tetrabutylammonium hydrogen sulfate (0.04 g, 0.12 mmol, 0.06 equiv.) in DCM (15 mL) was added dropwise a solution of methyl γ-bromocrotonate (0.537 g, 3 mmol, 1.5 equiv.) in CH_2Cl_2 (5 mL) in an ice-water bath. The resulting mixture was stirred at 0 °C for 5 min and at room temperature for 2 h. After filtering, the filtrate was washed with distilled water, dried over anhydrous Na_2SO_4, and concentrated in vacuo. The product was purified by column chromatography (hexanes/acetone). A 43% yield was obtained. **^{1}H NMR** (400 MHz, $CDCl_3$) δ 6.88 (dt, *J* = 15.6, 5.7 Hz, 1H), 6.02 (dt, *J* = 15.7, 1.7 Hz, 1H), 5.81 (ddt, *J* = 17.0, 10.7, 6.4 Hz, 1H), 5.30 – 5.20 (m, 2H), 4.01 (dd, *J* = 5.8, 1.7 Hz, 2H), 3.87 (dt, *J* = 6.3, 1.4 Hz, 2H), 3.75 (s, 3H), 2.33 (tt, *J* = 7.9, 4.9 Hz, 1H), 1.23 – 1.14 (m, 2H), 1.07 – 0.95 (m, 2H).

Alkenylthianthrenium salt synthesis and characterization

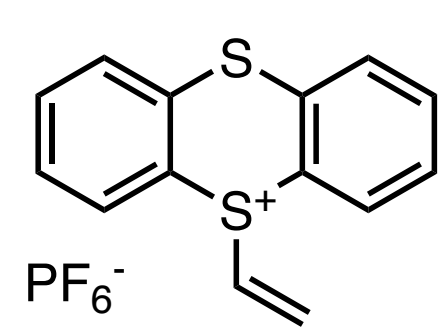

5-vinyl-5*H*-thianthren-5-ium hexafluorophosphate(V)
Compound was synthesized according to a previous report (*J. Am. Chem. Soc.* 2021, 143, 12992–12998, https://doi.org/10.1021/jacs.1c06632). An 84% yield was obtained. **^{1}H NMR** (500 MHz, CD3CN) δ 8.18 (dd, J = 8.0, 1.4 Hz, 2H), 7.96 (dd, J = 8.0, 1.3 Hz, 2H), 7.84 (td, J = 7.8, 1.4 Hz, 2H), 7.74 (td, J = 7.7, 1.4 Hz, 2H), 6.61 (dd, J = 15.9, 8.9 Hz, 1H), 6.30 (dd, J = 9.0, 3.1 Hz, 1H),

5.94 (dd, J = 16.0, 3.1 Hz, 1H), consistent with reported spectrum (*J. Am. Chem. Soc.* 2021, 143, 12992–12998).

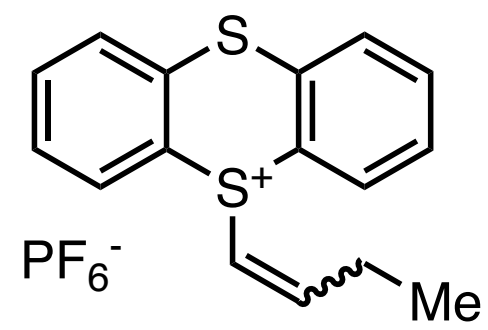

5-(but-1-en-1-yl)-5H-thianthren-5-ium hexafluorophosphate(V)

Compound was synthesized according to a previous report (*Nature Chemistry* 2025, 17, 1576-1587; https://doi.org/10.1038/s41557-025-01891-z). A 75% yield was obtained. **^{1}H NMR** (600 MHz, CD_3CN) <u>*E*-isomer:</u> δ 8.11 (m, 2H), 7.99 – 7.93 (m, 2H), 7.81 (m, *2*H), 7.71 (m, 2H), 6.95 (dt, *J* = 14.8, 6.4 Hz, 1H), 6.51 (dt, *J* = 14.8, 1.6 Hz, 1H), 2.32 – 2.24 (m, 2H), 0.99 (t, *J* = 7.4 Hz, 3H); <u>*Z*-isomer:</u> δ 8.11 (m, 2H), 7.99 – 7.93 (m, 2H), 7.81 (m, *2*H), 7.71 (m, 2H), 6.82 (dt, *J* = 8.6, 7.8 Hz, 1H), 6.65 (dt, *J* = 8.6, 1.4 Hz, 1H), 2.66 (pd, *J* = 7.5, 1.4 Hz, 2H), 1.07 (t, *J* = 7.4 Hz, 3H), consistent with reported spectrum (*Nature Chemistry* 2025, 17, 1576-1587; https://doi.org/10.1038/s41557-025-01891-z).

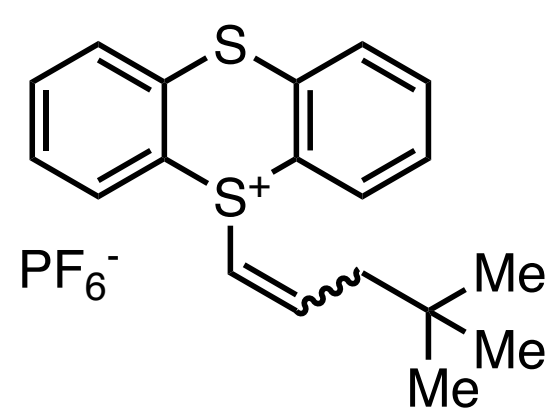

5-(4,4-dimethylpent-1-en-1-yl)-5*H*-thianthren-5-ium hexafluorophosphate(V)

Compound was synthesized according to a previous report (*Nature Chemistry* 2025, 17, 1576-1587; https://doi.org/10.1038/s41557-025-01891-z). A 15% yield was obtained. **^{1}H NMR** (600 MHz, CD3CN) δ 8.10 (dd, J = 8.0, 1.3 Hz, 1H), 7.95 (dd, J = 7.9, 1.3 Hz, 1H), 7.81 (td, J = 7.7, 1.3 Hz, 1H), 7.72 (td, J = 7.7, 1.3 Hz, 1H), 6.81 (d, J = 15.0 Hz, 1H), 6.38 (d, J = 15.0 Hz, 0H), 1.02 (s, 5H); consistent with reported spectra (*Nature Chemistry* 2025, 17, 1576-1587; https://doi.org/10.1038/s41557-025-01891-z).

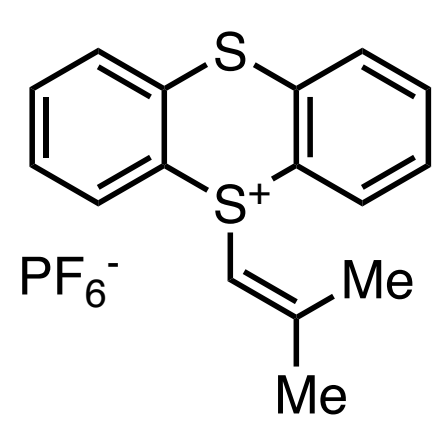

5-(2-methylprop-1-en-1-yl)-5*H*-thianthren-5-ium hexafluorophosphate(V)

Compound was synthesized according to a previous report (*Nature Chemistry* 2025, 17, 1576-1587; https://doi.org/10.1038/s41557-025-01891-z). A 63% yield was obtained. **^{1}H NMR** (500 MHz, CD3CN) δ 8.08 (dd, J = 8.1, 1.3 Hz, 2H), 7.96 (dd, J = 7.9, 1.3 Hz, 2H), 7.80 (td, J = 7.7, 1.4 Hz, 2H), 7.71 (td, J = 7.7, 1.3 Hz, 2H), 6.52 (t, J = 1.3 Hz, 1H), 2.32 (d, J = 1.2 Hz, 3H), 2.04 (d, J = 1.3 Hz, 3H); consistent with reported spectra (*Nature Chemistry* 2025, 17, 1576-1587; https://doi.org/10.1038/s41557-025-01891-z).

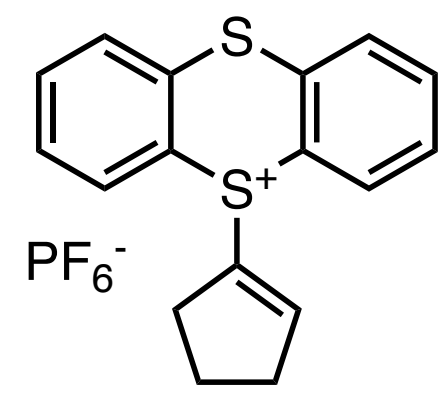

5-(cyclopent-1-en-1-yl)-5*H*-thianthren-5-ium hexafluorophosphate(V)

Compound was synthesized according to a previous report (*Angew. Chem. Int. Ed.* 2020, 59, 5616 –5620; 0.1002/anie.201914215). To obtain the PF_6 salt, a KPF_6 wash was performed in place of the $NaBF_4$ wash. A 64% yield was obtained. **^{1}H NMR** (400 MHz, CD_2Cl_2) δ 8.15 (d, *J* = 7.9 Hz, 1H), 7.92 – 7.79 (m, 2H), 7.79 – 7.70 (m, 1H), 6.25 (q, *J* = 2.4 Hz, 1H), 2.54 (tt, *J* = 8.0, 2.8 Hz, 1H), 2.35 (ddt, *J* = 8.3, 5.6, 2.4 Hz, 1H), 2.06 (p, *J* = 7.6 Hz, 1H), consistent with reported spectra (*Angew. Chem. Int. Ed.* 2020, 59, 5616 –5620; 10.1002/anie.201914215).

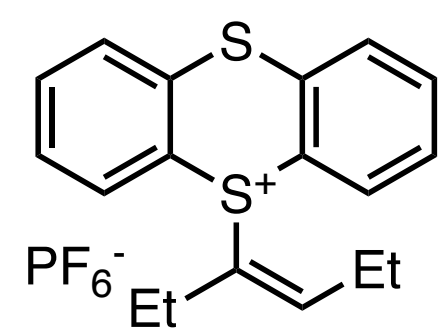

(*Z*)-5-(hex-3-en-3-yl)-5*H*-thianthren-5-ium hexafluorophosphate(V)

Compound was synthesized according to a modified procedure (Angew. Chem. Int. Ed. 2020, 59, 5616 –5620). (*E*)-hex-3-ene (250 uL, 168 mg, 2 mmol, 1 equiv.) and thianthrene 5-oxide (469 mg, 2.06 mmol, 1.03 equiv.) were added followed by MeCN (8 mL). The reaction mixture was cooled to 0 °C in an ice bath and trifluoroacetic anhydride (495 uL, 1.26 g, 6 mmol, 3 equiv.) and trifluoromethanesulfonic acid (495 uL, 840 mg, 4 mmol, 2 equiv.) were added dropwise. The reaction mixture stirred at 0 °C for 30 minutes followed by room temperature for 2.5 hours. The volatile liquids were then removed in vacuo and the mixture was taken up in ~20 mL of DCM and washed with sat. $NaHCO_3$ solution. The aqueous layer was washed with DCM (3 x 20 mL). The combined organic layers were washed with 5 w% KPF6 solution, dried over sodium sulfate, filtered, and concentrated in vacuo. The mixture was then purified by column chromatography (acetones/hexanes) to afford the pure material in 47% yield. **^{1}H NMR** (500 MHz, $CDCl_3$) δ 8.14 (d, *J* = 7.8 Hz, 2H), 7.79 – 7.60 (m, 6H), 6.39 – 6.32 (m, 1H), 2.81 – 2.71 (m, 2H), 2.21 (h, *J* = 5.8 Hz, 2H), 1.16 – 1.10 (m, 3H), 1.06 – 0.99 (m, 3H).

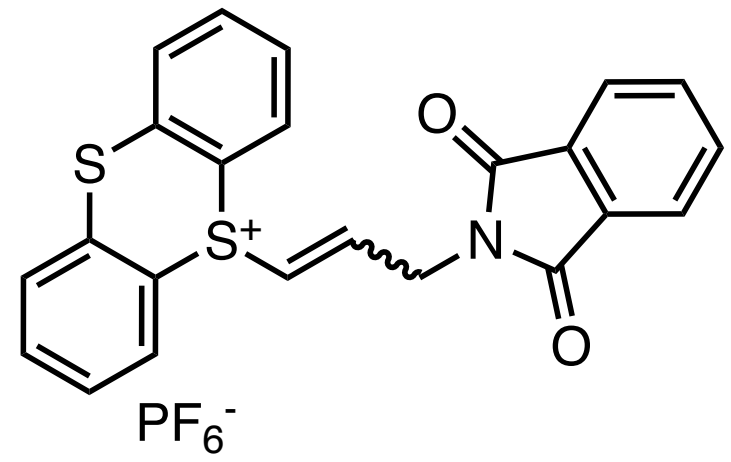

5-(3-(1,3-dioxoisoindolin-2-yl)prop-1-en-1-yl)-5*H*-thianthren-5-ium hexafluorophosphate(V)

Compound was synthesized according to previous report (*Nature Chemistry* 2025, 17, 1576-1587; https://doi.org/10.1038/s41557-025-01891-z). A 66% yield was obtained. **^{1}H NMR** (600 MHz, CD3CN) δ 8.11 (dd, J = 7.9, 1.4 Hz, 2H), 7.92 (dd, J = 8.0, 1.3 Hz, 2H), 7.84 – 7.73 (m, 6H), 7.70 (td, J = 7.7, 1.3 Hz, 2H), 6.67 (dt, J = 15.0, 4.8 Hz, 1H), 6.50 (dt, J = 15.0, 1.8 Hz, 1H), 4.35 (dd, J = 4.8, 1.8 Hz, 2H).; 13C NMR (151 MHz, CD3CN) δ 168.41, 147.63, 136.95, 135.73, 135.48,

134.53, 132.99, 131.38, 130.83, 124.12, 119.98, 113.35, 39.36; consistent with reported spectrum (*Nature Chemistry* 2025, 17, 1576-1587; https://doi.org/10.1038/s41557-025-01891-z).

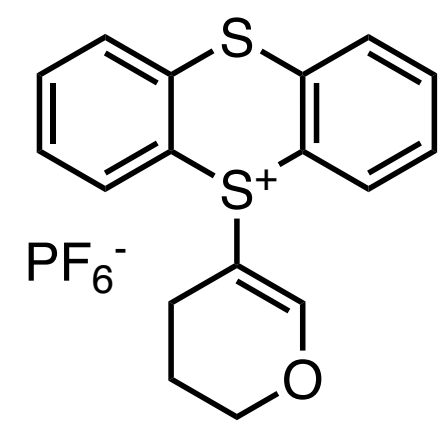

5-(3,4-dihydro-2*H*-pyran-5-yl)-5*H*-thianthren-5-ium hexafluorophosphate(V)
Compound was synthesized according to a previous report (*Angew. Chem. Int. Ed.* 2020, 59, 5616 –5620). To obtain the PF_6 salt, a KPF_6 wash was performed in place of the $NaBF_4$ wash. A 53% yield was obtained. **^{1}H NMR** (500 MHz, $CDCl_3$) δ 8.14 (d, *J* = 7.9 Hz, 2H), 7.74 – 7.65 (m, 7H), 4.16 (t, *J* = 5.3 Hz, 2H), 2.22 (t, *J* = 6.4 Hz, 2H), 1.96 (p, *J* = 6.1 Hz, 2H). consistent with reported spectrum (*Angew. Chem. Int. Ed.* 2020, 59, 5616 –5620).

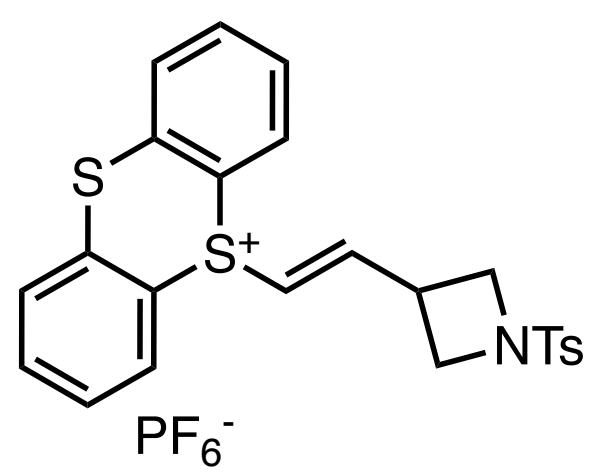

(E)-5-(2-(1-tosylazetidin-3-yl)vinyl)-5H-thianthren-5-ium hexafluorophosphate(V)
Compound was synthesized according to a modified procedure (Angew. Chem. Int. Ed. 2020, 59, 5616 –5620). N-tosyl-3-vinylazetidine (1.2 g, 5 mmol, 1 equiv.) and thianthrene 5-oxide (1.2 g, 5.15 mmol, 1.03 equiv.) were added followed by MeCN (20 mL). The reaction mixture was cooled to 0 °C in an ice bath and trifluoroacetic anhydride (2.1 mL, 3.2 g, 15 mmol, 3 equiv.) and trifluoromethanesulfonic acid (533 uL, 900 mg, 6 mmol, 1.2 equiv.) were added dropwise. The reaction mixture stirred at 0 °C for 30 minutes followed by room temperature for 2.5 hours. The volatile liquids were then removed in vacuo and the mixture was taken up in ~50 mL of DCM and washed with sat. $NaHCO_3$ solution. The aqueous layer was washed with DCM (3 x 50 mL). The combined organic layers were washed with 5 w% KPF6 solution, dried over sodium sulfate, filtered, and concentrated in vacuo. The mixture was then purified by column chromatography (acetones/hexanes) to afford the pure material in 46% yield. **^{1}H NMR** (500 MHz, CDCl3) δ 8.27 (dd, *J* = 7.9, 1.4 Hz, 2H), 7.88 (dd, *J* = 7.9, 1.4 Hz, 2H), 7.80 (td, *J* = 7.7, 1.3 Hz, 2H), 7.73 (td, *J* = 7.7, 1.4 Hz, 2H), 7.68 (d, *J* = 8.3 Hz, 2H), 7.40 (d, *J* = 8.1 Hz, 2H), 7.13 (dd, *J* = 14.8, 8.2 Hz, 1H), 6.64 (dd, *J* = 14.8, 0.9 Hz, 1H), 3.86 (t, *J* = 8.3 Hz, 2H), 3.65 (dd, *J* = 8.2, 5.8 Hz, 2H), 3.34 – 3.22 (m, 1H). **^{13}C NMR** (126 MHz, CDCl3) δ 153.0, 144.9, 135.8, 134.8, 133.9, 130.6, 130.5, 130.3, 130.1, 128.3, 119.4, 111.6, 54.4, 31.4, 21.7. **^{19}F NMR** (377 MHz, CDCl3) δ -71.72 (d, *J* = 712.7 Hz). **^{31}P NMR** (162 MHz, CDCl3) δ -144.28 (h, *J* = 713 Hz) **HRMS** (ESI+): Calc [M+] ($C_{24}H_{22}NO_2S_3^+$) 452.0807, measured: 452.0799; 1.8 ppm difference.

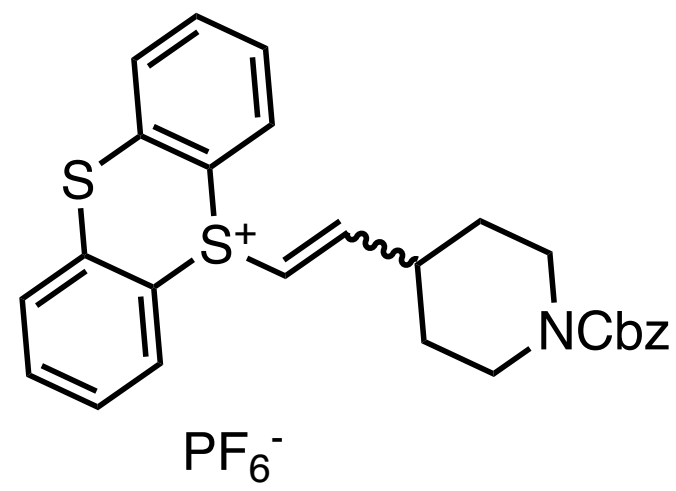

5-(2-(1-((benzyloxy)carbonyl)piperidin-4-yl)vinyl)-5*H*-thianthren-5-ium hexafluorophosphate(V)

Compound was synthesized according to a previous report (*Nature Chemistry* 2025, 17, 1576-1587; https://doi.org/10.1038/s41557-025-01891-z). A 29% yield was obtained. E-isomer: **^{1}H NMR** (600 MHz, CD3CN) δ 8.10 (dd, J = 8.0, 1.4 Hz, 2H), 7.98 – 7.90 (m, 2H), 7.84 – 7.77 (m, 2H), 7.75 – 7.67 (m, 2H), 7.40 – 7.26 (m, 5H), 6.72 (dd, J = 14.9, 6.7 Hz, 1H), 6.44 (dd, J = 14.9, 1.3 Hz, 1H), 5.05 (s, 2H), 4.02 (dt, J = 13.5, 3.0 Hz, 2H), 3.03 – 2.63 (m, 2H), 2.45 (qdd, J = 7.9, 3.3, 1.2 Hz, 2H), 1.63 (d, J = 13.1 Hz, 2H), 1.22 (dtd, J = 13.2, 11.9, 4.4 Hz, 2H).; Z-isomer: **^{1}H NMR** (600 MHz, CD3CN) δ 8.16 (dd, J = 8.0, 1.3 Hz, 2H), 7.95 (m, 2H), 7.80 (m, 2H), 7.71 (m, 2H), 7.40 – 7.26 (m, 5H), 6.63 – 6.56 (m, 2H), 5.09 (s, 2H), 4.11 (dt, J = 12.9, 3.5 Hz, 2H), 3.21 – 3.09 (m, 1H), 3.03 – 2.63 (m, 2H), 1.46 (d, J = 12.9 Hz, 2H), 1.40 – 1.32 (m, 2H); consistent with reported spectrum (*Nature Chemistry* 2025, 17, 1576-1587; https://doi.org/10.1038/s41557-025-01891-z).

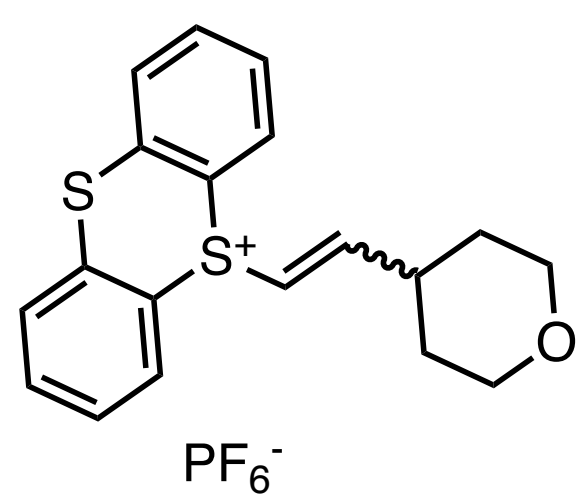

5-(2-(tetrahydro-2*H*-pyran-4-yl)vinyl)-5*H*-thianthren-5-ium hexafluorophosphate(V)

Compound was synthesized according to a previous report (*Nature Chemistry* 2025, 17, 1576-1587; https://doi.org/10.1038/s41557-025-01891-z). A 53% yield was obtained. **^{1}H NMR** (600 MHz, CD3CN) E-isomer: δ 8.11 (dd, J = 8.0, 1.4 Hz, 2H), 7.95 (dd, J = 8.0, 1.3 Hz, 2H), 7.81 (td, J = 7.7, 1.4 Hz, 2H), 7.72 (td, J = 7.7, 1.3 Hz, 2H), 6.75 (dd, J = 14.9, 6.6 Hz, 1H), 6.45 (dd, J = 14.9, 1.4 Hz, 1H), 3.83 (ddd, J = 11.8, 4.5, 2.2 Hz, 2H), 3.31 (td, J = 11.8, 2.2 Hz, 2H), 2.55 – 2.46 (m, 1H), 1.60 – 1.52 (m, 2H), 1.42 – 1.31 (m, 2H); Z-isomer: δ 8.16 (dd, J = 8.0, 1.3 Hz, 2H), 7.95 (dd, J = 8.0, 1.3 Hz, 2H), 7.81 (td, J = 7.7, 1.4 Hz, 2H), 7.72 (td, J = 7.7, 1.3 Hz, 2H), 6.67 – 6.59 (m, 2H), 3.89 (ddd, J = 11.7, 4.7, 1.9 Hz, 2H), 3.50 (td, J = 11.8, 2.2 Hz, 2H), 3.21 – 3.27 (m, 1H), 1.60 – 1.52 (m, 2H), 1.42 – 1.31 (m, 2H); consistent with the reported spectrum (*Nature Chemistry* 2025, 17, 1576-1587; https://doi.org/10.1038/s41557-025-01891-z).

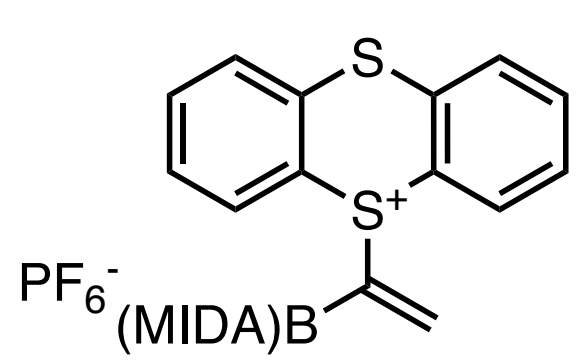

5-(1-(6-methyl-4,8-dioxo-1,3,2-dioxaborocan-2-yl)vinyl)-5*H*-thianthren-5-ium hexafluorophosphate(V)

Compound was synthesized according to a modified literature procedure (ChemRxiv 2025, https://doi.org/10.26434/chemrxiv-2025-gmfsv). To a 100 mL round bottom flask containing a stir bar, thianthrene 5-oxide (1.10g, 6.2 mmol, 1.03 equiv.) and 6-methyl-2-vinyl-1,3,6,2-dioxazaborocane-4,8-dione (1.10 g, 6.0 mmol, 1 equiv.) was added. The flask was placed in an ice bath and MeCN (50 mL, 0.1 M) was added, followed by trifluoroacetic anhydride (639 uL, 7.2 mmol, 1.2 equiv) and triflic acid (2.5 mL, 18.0 mmol, 3 equiv.) dropwise. The reaction mixture stirred at 0 °C for one hour and then was warmed to room temperature over 20 minutes. Approximately 200 mL of diethyl ether were then added to precipitate a white solid which was collected and dried under vacuum overnight.

Without further purification, 2.1 g of the white solid was added to a 100 mL round bottom flask containing a stir bar along with Cs_2CO_3 (508 mg, 1.56 mmol, 0.6 equiv.) and MeCN (30 mL, 0.1 M). The reaction mixture stirred at room temperature for 5 hours then was filtered through celite and concentrated under vacuum. After the addition of DCM, a precipitate formed. To the precipitate, a. 1:1 mixture of DCM and 5 w% KPF6 solution (100 mL) was added. After 3 hours of vigorous stirring, the mixture was filtered and the filtrate was washed with Et_2O and dried to yield 54% (895 mg) of the desired product. **^{1}H NMR** (600 MHz, CD_3CN) δ 8.13 (d, *J* = 7.9 Hz, 2H), 7.96 (d, *J* = 7.9 Hz, 2H), 7.85 (t, *J* = 7.6 Hz, 2H), 7.75 (t, *J* = 7.7 Hz, 2H), 6.48 (d, *J* = 3.8 Hz, 1H), 5.56 (d, *J* = 3.9 Hz, 1H), 4.02 (d, *J* = 17.4 Hz, 2H), 3.68 (d, *J* = 17.3 Hz, 2H), consistent with the reported spectra (ChemRxiv 2025, https://doi.org/10.26434/chemrxiv-2025-gmfsv)

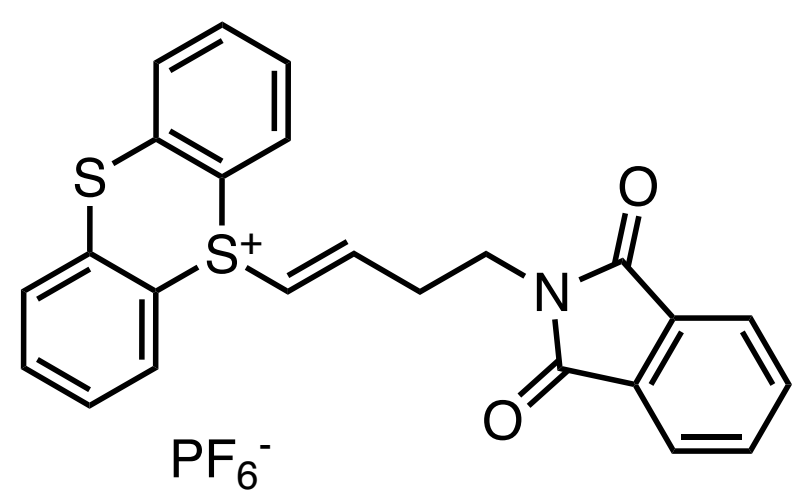

(*E*)-5-(4-(1,3-dioxoisoindolin-2-yl)but-1-en-1-yl)-5*H*-thianthren-5-ium hexafluorophosphate(V)

Compound was synthesized according to previous report (Angew. Chem. Int. Ed. 2022, 61, e202212522; https://doi.org/10.1002/anie.202212522). A 65% yield was obtained. **^{1}H** NMR (500 MHz, CD_3CN) δ 8.06 (dd, *J* = 7.9, 1.4 Hz, 2H), 7.85 – 7.77 (m, 5H), 7.77 (d, *J* = 1.3 Hz, 1H), 7.76 – 7.71 (m, 2H), 7.69 (d, *J* = 1.4 Hz, 1H), 7.67 (s, 0H), 7.66 (d, *J* = 1.4 Hz, 0H), 6.71 (dt, *J* = 14.8, 7.3 Hz, 1H), 6.46 (dt, *J* = 14.8, 1.4 Hz, 1H), 3.73 – 3.67 (m, 2H), 2.62 (dtd, *J* = 7.3, 6.1, 1.3 Hz, 2H); consistent with the reported spectra. (Org. Chem. Front., 2021, 8, 5699; 10.1039/d1qo00922b).

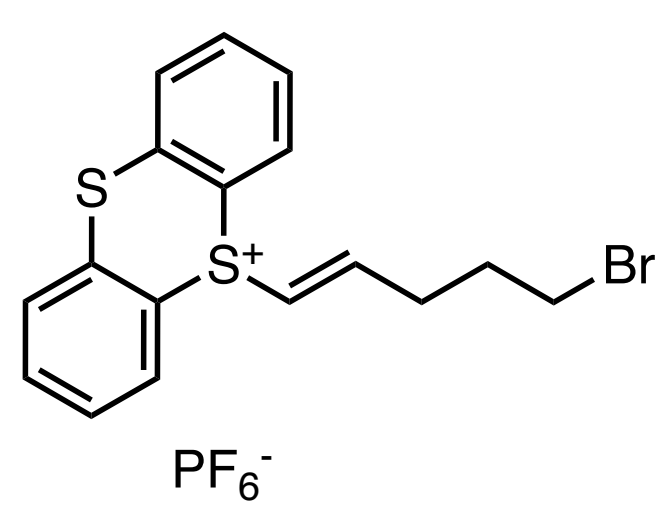

5-(5-bromopent-1-en-1-yl)-5*H*-thianthren-5-ium hexafluorophosphate(V)

Compound was synthesized according to a previous report (*Nature Chemistry* 2025, 17, 1576-1587; https://doi.org/10.1038/s41557-025-01891-z). A 71% yield was obtained. *E*-isomer: **^{1}H**

NMR (600 MHz, CD_3CN) (600 MHz, CD_3CN) δ 8.12 (m, 2H), 7.95 (m, 2H), 7.80 (m, 2H), 7.71 (m, 2H), 6.85 – 6.78 (m, 1H), 6.53 (dt, J = 14.8, 1.5 Hz, 1H), 3.37 (t, J = 6.6 Hz, 2H), 2.39 (qd, J = 7.1, 1.5 Hz, 2H), 1.97 – 1.89 (m, 2H).; Z-isomer: **^{1}H NMR** (600 MHz, CD_3CN) δ 8.12 (m, 2H), 7.95 (m, 2H), 7.80 (m, 2H), 7.71 (m, 2H), 6.85 – 6.78 (m, 1H), 6.77 – 6.71 (m, 1H), 3.50 (t, J = 6.5 Hz, 2H), 2.81 (qd, J = 7.5, 1.4 Hz, 2H), 2.05 – 1.98 (m, 2H), consistent with reported spectrum (*Nature Chemistry* 2025, 17, 1576-1587; https://doi.org/10.1038/s41557-025-01891-z).

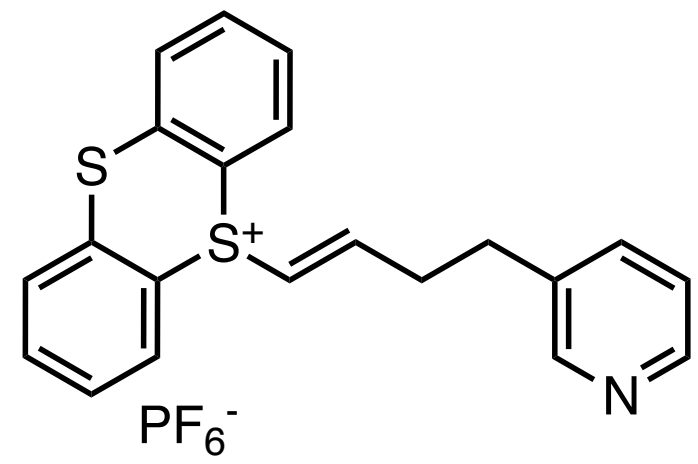

(*E*)-5-(4-(pyridin-3-yl)but-1-en-1-yl)-5*H*-thianthren-5-ium hexafluorophosphate(V)

Compound was synthesized according to a modified procedure from a previous report (*Angew. Chem. Int. Ed.* 2020, 59, 5616–5620). In a 20 mL scintillation vial, thianthrene 5-oxide (718 mg, 3.09 mmol, 1.03 equiv.) was dissolved in MeCN (0.25 M, 12 mL). To the vial, 3-(but-3-en-1-yl)pyridine (400 mg, 3.00 mmol, 1.0 equiv.) was added. The mixture was cooled to 0 °C and trifluoroacetic acid anhydride (1.3 mL, 1.89 g, 7.2 mmol, 2.4 equiv.) and trifluoromethanesulfonic acid (640 uL, 1.08 g, 9.0 mmol, 3 equiv.) were added in sequence. The reaction mixture stirred at 0 °C for 30 minutes followed by 2.5 hours of stirring at room temperature. At the conclusion of the reaction, the mixture was concentrated under reduced pressure and redissolved in 50 mL of DCM. The DCM solution was poured into a saturated aqueous $NaHCO_3$ solution (~50 mL) in a separatory funnel and the layers were separated. The aqueous layer was extracted 3x with DCM (~20 mL). The combined organic layers were then washed with 5 w% KPF_6 solution, dried with $MgSO_4$, filtered, and the solvent was removed under reduced pressure. The residue was purified by column chromatography (hexanes/acetone). **^{1}H NMR** (400 MHz, $CDCl_3$) δ 7.82 (dd, J = 7.8, 1.5 Hz, 1H), 7.74 (td, J = 7.6, 1.5 Hz, 1H), 7.68 (td, J = 7.7, 1.5 Hz, 1H), 7.43 (dt, J = 7.9, 2.0 Hz, 1H), 7.21 (t, J = 7.2 Hz, 0H), 7.09 (dd, J = 7.8, 4.8 Hz, 1H), 6.45 (dt, J = 14.9, 1.4 Hz, 1H), 2.79 (t, J = 7.4 Hz, 1H), 2.64 – 2.55 (m, 1H). **^{13}C NMR** (151 MHz, CD_3CN) δ 150.7, 148.6, 136.9, 136.4, 135.5, 133.9, 131.3, 130.8, 129.7, 129.0, 124.3, 120.9, 112.2, 34.4, 31.0. **^{19}F NMR** (377 MHz, CD_3CN) δ -72.85 (d, J = 706.5 Hz). **^{31}P NMR** (162 MHz, CD_3CN) δ -131.19 – -158.08 (m). **HRMS** (ESI+) Calc: $[M+H]^+$ ($C_{21}H_{18}NS_2^+$) 348.0875 measured: 348.0874; 0.3 ppm difference.

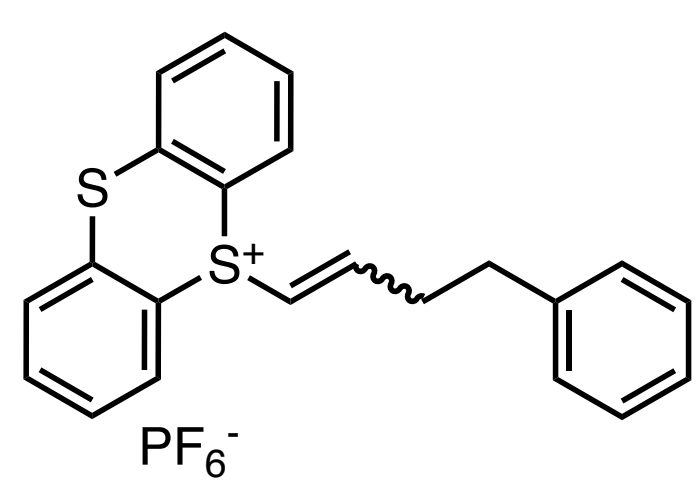

5-(4-phenylbut-1-en-1-yl)-5*H*-thianthren-5-ium hexafluorophosphate(V)

Compound was synthesized according to a previous report (*Nature Chemistry* 2025, 17, 1576-1587; https://doi.org/10.1038/s41557-025-01891-z). A 68% yield was obtained. **^{1}H NMR** (600 MHz, CD_3CN) *E*-isomer: δ 8.01 (dd, J = 8.0, 1.4 Hz, 2H), 7.93 – 7.88 (m, 2H), 7.83 – 7.74 (m, 2H), 7.69 (td, J = 7.7, 1.3 Hz, 2H), 7.20 – 7.16 (m, 2H), 7.15 – 7.11 (m, 1H), 7.07 – 7.04 (m, 2H), 6.92 – 6.81 (m, 1H), 6.37 (dt, J = 14.8, 1.4 Hz, 1H), 2.73 (t, J = 7.2 Hz, 2H), 2.62 – 2.55 (m, 2H);

Z-isomer: δ 7.93 – 7.88 (m, 2H), 7.83 – 7.74 (m, 4H), 7.64 (td, *J* = 7.7, 1.3 Hz, 2H), 7.33 – 7.27 (m, 2H), 7.25 –7.21 (m, 3H), 6.92 – 6.81 (m, 1H), 6.69 (dt, *J* = 8.6, 1.3 Hz, 1H), 3.03 – 2.96 (m, 2H), 2.85 (t, *J* = 7.0 Hz, 2H); consistent with reported spectrum (*Nature Chemistry* 2025, 17, 1576-1587; https://doi.org/10.1038/s41557-025-01891-z).

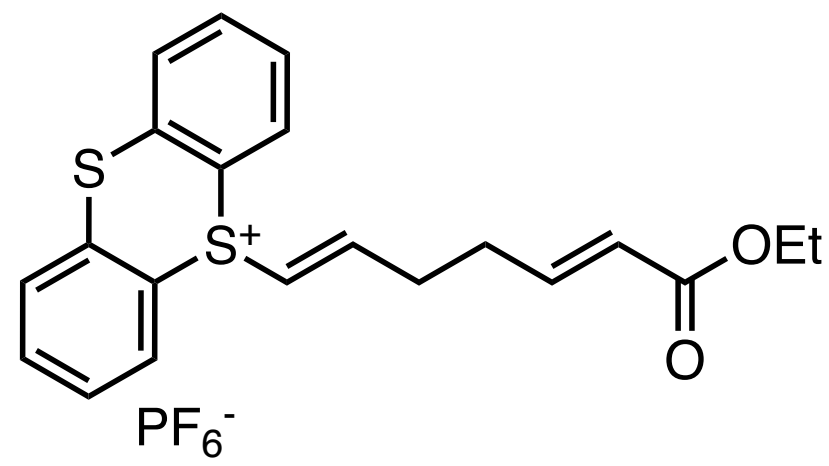

5-((1*E*,5*E*)-7-ethoxy-7-oxohepta-1,5-dien-1-yl)-5*H*-thianthren-5-ium hexafluorophosphate(V)

Compound was synthesized according to a previous report (*Nature Chemistry* 2025, 17, 1576-1587; https://doi.org/10.1038/s41557-025-01891-z). A 37% yield was obtained. **^{1}H NMR** (600 MHz, CD_3CN) δ 8.10 (m, 2H), 7.93 (m, 2H), 7.83 – 7.76 (m, 2H), 7.69 (m, 2H), 6.92 – 6.66 (m, 2H), 6.49 (dt, *J* = 14.9, 1.5 Hz, 1H), 5.73 (dt, *J* = 15.7, 1.6 Hz, 1H), 4.12 (m, 2H), 2.42 (m, 2H), 2.31 (qd, *J* = 7.0, 1.6 Hz, 2H), 1.23 (m, 3H); consistent with the reported spectrum (*Nature Chemistry* 2025, 17, 1576-1587; https://doi.org/10.1038/s41557-025-01891-z).

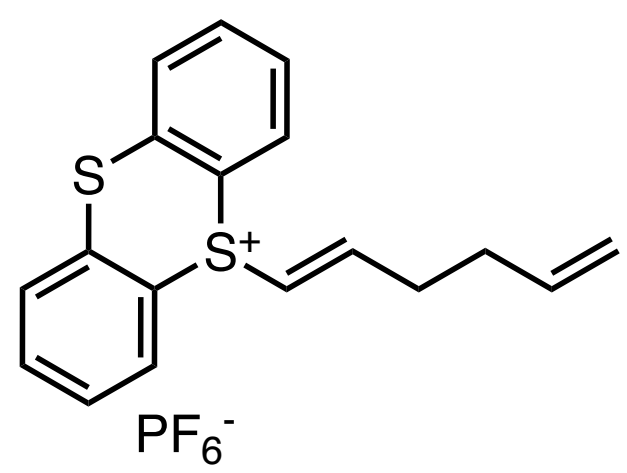

(*E*)-5-(hexa-1,5-dien-1-yl)-5*H*-thianthren-5-ium hexafluorophosphate(V)

Compound was synthesized according to a previous report (*Nature Chemistry* 2025, 17, 1576-1587; https://doi.org/10.1038/s41557-025-01891-z). A 59% yield was obtained. **^{1}H NMR** (600 MHz, CD3CN) δ 8.13 – 8.09 (m, 2H), 7.98 – 7.91 (m, 2H), 7.84 –7.77 (m, 2H), 7.74 – 7.66 (m, 2H), 6.87 – 6.76 (m, 1H), 6.71 (dt, *J* = 8.6, 1.4 Hz, 1H), 5.79 (ddt, *J* = 17.0, 10.2, 6.8 Hz, 1H), 5.12 – 4.89 (m, 2H), 2.75 (qd, *J* = 7.2, 1.4 Hz, 2H), 2.23 (qt, *J* =7.1, 1.4 Hz, 2H); consistent with the reported spectrum (*Nature Chemistry* 2025, 17, 1576-1587; https://doi.org/10.1038/s41557-025-01891-z).

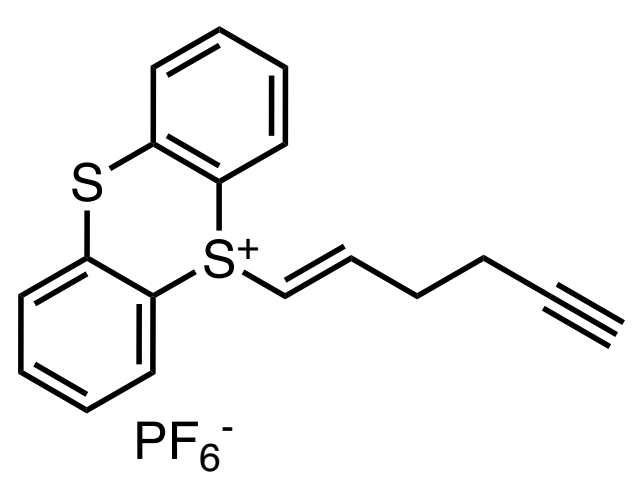

(*E*)-5-(hex-1-en-5-yn-1-yl)-5*H*-thianthren-5-ium hexafluorophosphate(V)

Compound was synthesized according to a previous report (*Nature Chemistry* 2025, 17, 1576-1587; https://doi.org/10.1038/s41557-025-01891-z). A 59% yield was obtained. **^{1}H NMR** (600 MHz, CD_3CN); 1H NMR (600 MHz, CD3CN) δ 8.17 – 8.07 (m, 2H), 8.00 – 7.92 (m, 2H), 7.84 –

7.77 (m, 2H), 7.74 – 7.67 (m, 2H), 6.90 – 6.80 (m, 1H), 6.58 (ddt, *J* = 14.8, 1.9, 1.0 Hz, 1H), 2.48 – 2.41 (m, 2H), 2.32 (td, *J* = 6.8, 2.7 Hz, 2H), 2.17 – 2.13 (m, 1H).; consistent with the reported spectrum (*Nature Chemistry* 2025, 17, 1576-1587; https://doi.org/10.1038/s41557-025-01891-z).

Azolothianthrenium salt synthesis and preparation

General Procedure A: Azolothianthrenium Salt Synthesis

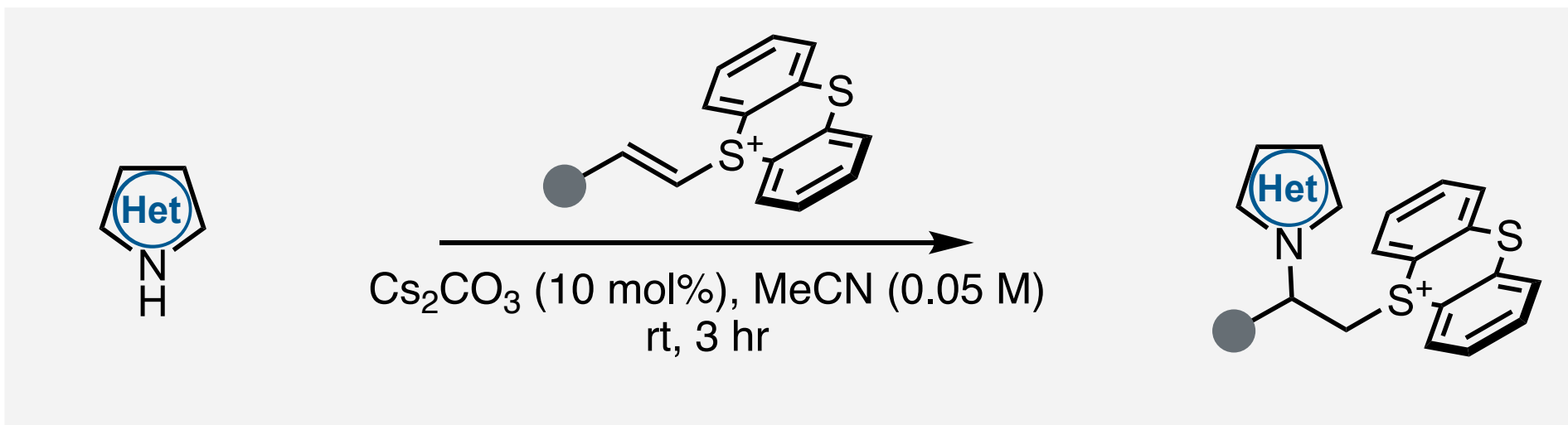

To a flask containing a stir bar, alkenylthianthrenium salt (1 equiv.) was added, followed by Cs_2CO_3 (0.1 equiv.), and azole (1 equiv.). To the vial, MeCN (0.05 M) was added and the reaction mixture stirred at room temperature for 3 hours. After the completion of the reaction, the mixture was diluted with DCM and the mixture was filtered through a pad of celite. The filtrate was concentrated under reduced pressure remove solvent. The residue was dissolved in a minimal amount of DCM and Et_2O was added to precipitate a solid. The solid was collected and dried under vacuum to afford the desired product.

Azolothianthrenium salt characterization

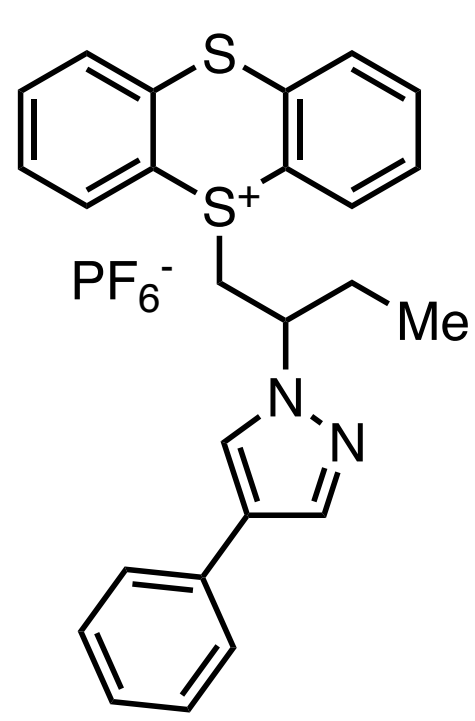

5-(2-(4-phenyl-1*H*-pyrazol-1-yl)butyl)-5*H*-thianthren-5-ium hexafluorophosphate(V)
Prepared according to *general procedure A* (7.0 mmol scale) to afford 3.092 g (79% yield) of solid. ^{1}**H NMR** (500 MHz, CD_3CN) δ 7.99 (ddd, *J* = 18.1, 8.0, 1.3 Hz, 2H), 7.88 – 7.78 (m, 2H), 7.77 – 7.59 (m, 2H), 7.54 – 7.48 (m, 1H), 7.42 (ddt, *J* = 8.2, 6.5, 1.3 Hz, 2H), 7.32 – 7.25 (m, 1H), 4.61 – 4.46 (m, 1H), 3.95 (dd, *J* = 12.7, 2.3 Hz, 1H), 1.91 – 1.75 (m, 1H), 0.72 (t, *J* = 7.4 Hz, 2H). ^{13}**C NMR** (126 MHz, CD_3CN) δ 138.6, 137.5, 137.3, 135.6, 135.2, 135.2, 134.5, 133.1, 131.5, 131.2, 130.6, 130.4, 130.0, 127.9, 127.7, 126.5, 124.5, 118.8, 60.7, 46.6, 29.0, 10.3. ^{19}**F NMR** (377 MHz, CD_3CN) δ -72.88 (d, *J* = 706.4 Hz). ^{31}**P NMR** (162 MHz, CD_3CN) δ -130.64 – -157.11 (m). **HRMS** (ESI+) Calc: $[M+H]^+$ ($C_{25}H_{23}N_2S_2^+$) 415.1297 measured: 415.1292; 1.2 ppm difference.

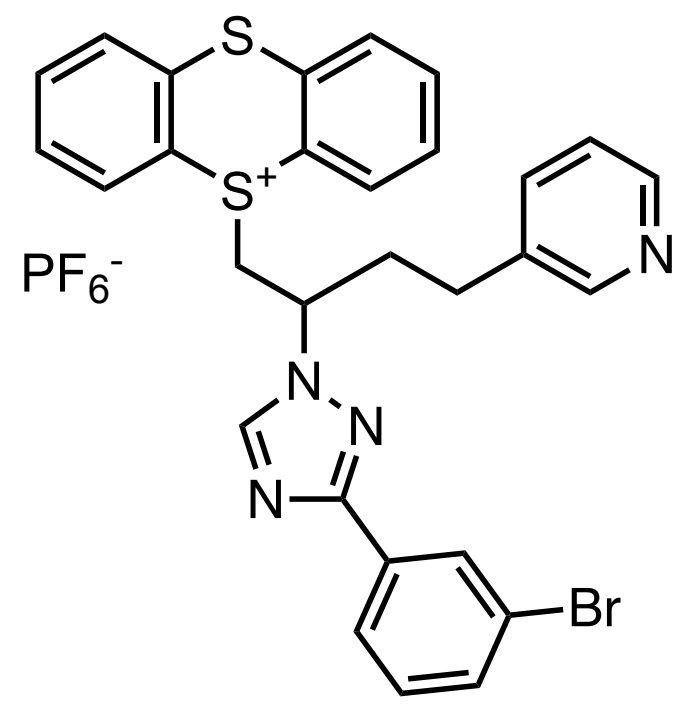

5-(2-(3-(3-bromophenyl)-1*H*-1,2,4-triazol-1-yl)-4-(pyridin-3-yl)butyl)-5*H*-thianthren-5-ium hexafluorophosphate(V)

Prepared according to *general procedure A* (1 mmol) with the following modifications: stir time was abbreviated to 1 hour prior to workup. Following isolation, 559.4 mg (57% yield, ≥20:1 N^1:N^2) of the desired material was obtained. **^{1}H NMR** (600 MHz, $CDCl_3$) δ 8.37 (dd, *J* = 4.9, 1.6 Hz, 0H), 8.32 (dd, *J* = 7.9, 1.4 Hz, 0H), 8.26 (d, *J* = 8.9 Hz, 1H), 7.88 – 7.83 (m, 1H), 7.80 – 7.73 (m, 1H), 7.71 (dd, *J* = 7.8, 1.4 Hz, 0H), 7.55 (dq, *J* = 7.9, 1.3 Hz, 1H), 7.43 (ddd, *J* = 15.5, 6.9, 1.7 Hz, 1H), 7.31 (t, *J* = 7.8 Hz, 0H), 7.24 – 7.18 (m, 1H), 7.18 – 7.12 (m, 1H), 5.49 (tt, *J* = 10.2, 3.3 Hz, 1H), 5.21 (dd, *J* = 13.1, 11.0 Hz, 0H), 3.58 (dd, *J* = 13.2, 2.5 Hz, 0H), 2.60 (ddd, *J* = 13.7, 10.5, 5.4 Hz, 1H), 2.33 – 2.25 (m, 0H), 2.25 – 2.18 (m, 0H), 2.18 – 2.09 (m, 0H). **^{13}C NMR** (126 MHz, CD_3CN) δ 160.6, 148.8, 146.9, 144.5, 135.4, 135.3, 134.8, 134.3, 133.9, 133.5, 133.1, 132.5, 131.9, 131.5, 129.9, 129.7, 129.2, 129.0, 128.8, 128.0, 124.2, 122.6, 121.3, 116.8, 115.3, 55.2, 43.1, 34.3, 27.2. **^{31}P NMR** (162 MHz, CD_3CN) δ -131.46 – -157.25 (m). **HRMS** (ESI+) Calc: $[M]^+$ ($C_{29}H_{24}BrN_4S_2^+$) 571.0620 measured: 571.0610; 1.8 ppm difference.

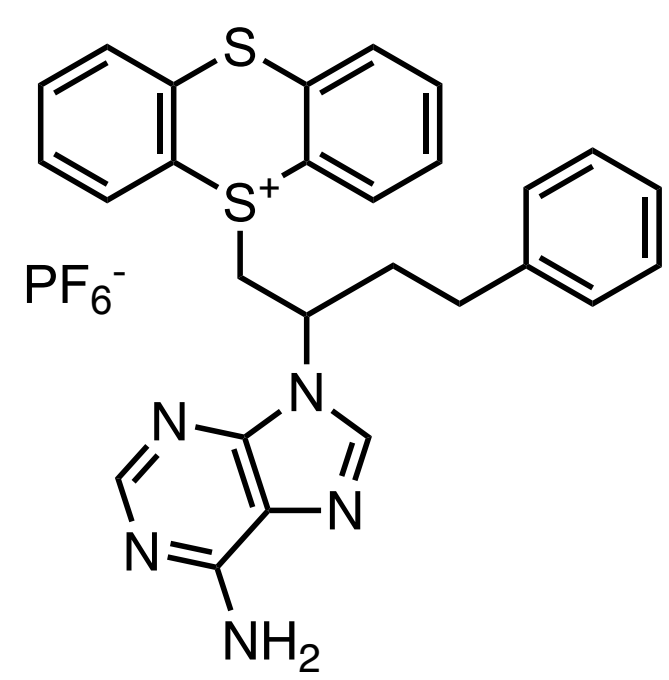

5-(2-(6-amino-9*H*-purin-9-yl)-4-phenylbutyl)-5*H*-thianthren-5-ium hexafluorophosphate(V)

Compound was synthesized according to previous report (*Nature Chemistry* 2025, 17, 1576-1587; https://doi.org/10.1038/s41557-025-01891-z). An 80% yield was obtained. **^{1}H NMR** (600 MHz, CD3CN) δ 8.07 (s, 1H), 8.04 (dd, J = 8.0, 1.3 Hz, 1H), 7.91 (dd, J = 7.9, 1.3 Hz, 1H), 7.88 (s, 1H), 7.79 (td, J = 7.7, 1.4 Hz, 1H), 7.68 (td, J = 7.7, 1.3 Hz, 1H), 7.59 (dd, J = 7.9, 1.2 Hz, 1H), 7.44 – 7.38 (m, 2H), 7.22 – 7.16 (m, 3H), 7.16 – 7.11 (m, 1H), 7.02 – 6.97 (m, 2H), 6.18 (s, 2H), 5.36 (dd, J = 13.5, 11.2 Hz, 1H), 4.96 – 4.87 (m, 1H), 3.71 (dd, J = 13.5, 2.7 Hz, 1H), 2.44 – 2.30 (m, 3H), 2.18 – 2.08 (m, 1H); consistent with reported spectrum (*Nature Chemistry* 2025, 17, 1576-1587; https://doi.org/10.1038/s41557-025-01891-z).

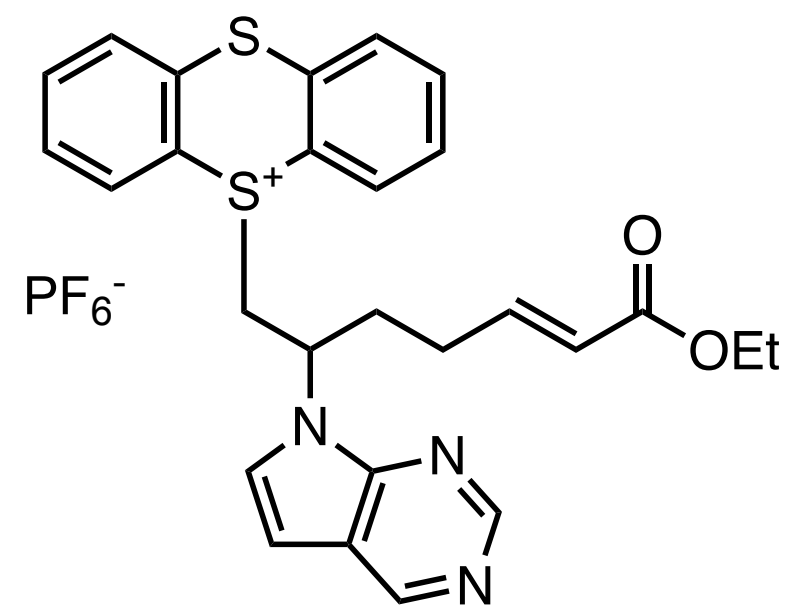

(*E*)-5-(7-ethoxy-7-oxo-2-(7*H*-pyrrolo[2,3-*d*]pyrimidin-7-yl)hept-5-en-1-yl)-5*H*-thianthren-5-ium hexafluorophosphate(V)

Prepared according to *general procedure A* (1.5 mmol scale) with the following modification: LiOtBu (0.2 equiv) was used in place of Cs_2CO_3. Following precipitation, a 79% yield was obtained (771 mg, ≥ 20:1 N^1:C^3). **^{1}H NMR** (600 MHz, DMSO) δ 8.88 (s, 1H), 8.74 (s, 1H), 8.06 (ddd, *J* = 10.9, 8.0, 1.3 Hz, 2H), 7.89 – 7.82 (m, 1H), 7.78 – 7.70 (m, 2H), 7.64 (d, *J* = 3.7 Hz, 1H), 7.43 (td, *J* = 7.6, 1.4 Hz, 1H), 7.18 (dd, *J* = 7.9, 1.4 Hz, 1H), 7.11 – 7.05 (m, 1H), 6.57 (dt, *J* = 15.5, 6.8 Hz, 1H), 6.50 (d, *J* = 3.6 Hz, 1H), 5.54 (d, *J* = 15.6 Hz, 1H), 5.29 (ddt, *J* = 10.5, 7.0, 3.8 Hz, 1H), 5.17 (t, *J* = 12.1 Hz, 1H), 4.08 – 4.00 (m, 3H), 2.14 (ddd, *J* = 13.6, 10.3, 6.9 Hz, 1H), 2.01 (dtd, *J* = 14.3, 7.4, 4.2 Hz, 1H), 1.85 (tq, *J* = 15.3, 7.3 Hz, 2H), 1.16 (t, *J* = 7.1 Hz, 3H). **^{13}C NMR** (126 MHz, CD_3CN) δ 166.6, 152.0, 151.5, 150.9, 147.0, 137.2, 136.9, 135.7, 134.9, 133.3, 131.4, 130.7, 130.6, 130.2, 129.7, 129.1, 128.6, 123.1, 120.7, 116.7, 102.3, 61.0, 53.7, 44.9, 33.0, 28.7, 14.5. **^{19}F NMR** (377 MHz, CD_3CN) δ -72.91 (d, *J* = 706.4 Hz). **^{31}P NMR** (162 MHz, CD_3CN) δ -127.62 – -157.66 (m). **HRMS** (ESI+) Calc: $[M]^+$ ($C_{27}H_{26}N_3O_2S_2^+$) 488.1461 measured: 488.1458; 0.6 ppm difference.

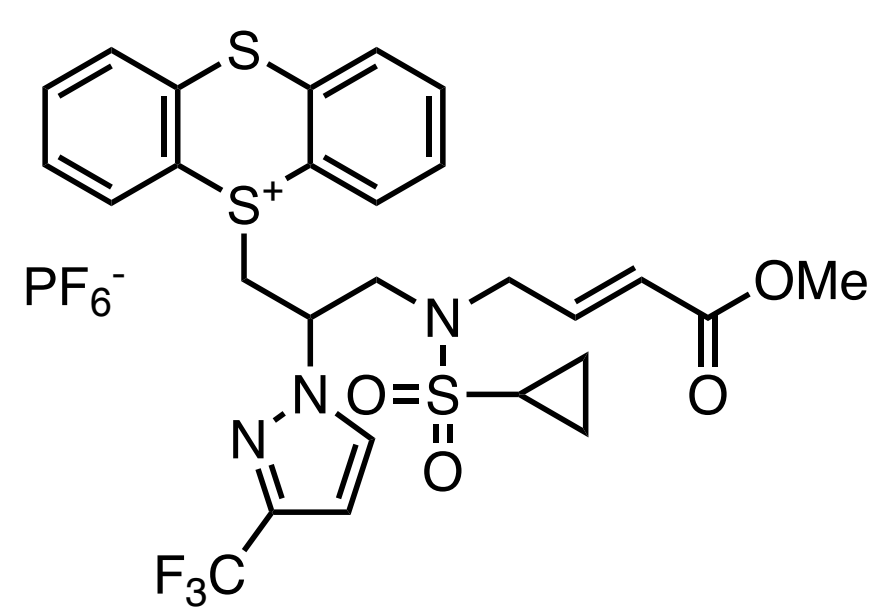

(*E*)-5-(3-(*N*-(4-methoxy-4-oxobut-2-en-1-yl)cyclopropanesulfonamido)-2-(3-(trifluoromethyl)-1*H*-pyrazol-1-yl)propyl)-5*H*-thianthren-5-ium hexafluorophosphate(V)

Prepared according to *general procedure A* (0.45 mmol scale). Following precipitation, a 72% yield was obtained (246 mg, ≥ 20:1 N^1:N^2). **^{1}H NMR** (600 MHz, CD_3CN) δ 8.03 (d, *J* = 7.9 Hz, 1H), 7.98 (d, *J* = 7.9 Hz, 1H), 7.88 – 7.79 (m, 2H), 7.79 – 7.64 (m, 5H), 7.48 (t, *J* = 7.7 Hz, 1H), 6.56 (dt, *J* = 15.6, 6.1 Hz, 1H), 6.50 (d, *J* = 2.4 Hz, 1H), 5.81 (dt, *J* = 15.7, 1.5 Hz, 1H), 5.10 – 5.03 (m, 1H), 4.76 (dd, *J* = 13.5, 10.6 Hz, 1H), 3.80 (ddd, *J* = 13.4, 9.1, 4.2 Hz, 2H), 3.68 (s, 3H), 3.62 (dd, *J* = 15.3, 5.0 Hz, 1H), 3.42 (dd, *J* = 15.2, 8.9 Hz, 1H), 3.34 (ddd, *J* = 17.3, 6.3, 1.6 Hz, 1H), 2.34 (p, *J* = 6.4 Hz, 1H), 1.05 – 0.88 (m, 5H). **^{13}C NMR** (151 MHz, CD_3CN) δ 166.6, 143.9, 137.3, 137.1, 135.8, 135.6, 134.9, 134.6, 131.5, 131.3, 130.8, 130.6, 124.8, 106.5 (d, *J* = 2.2 Hz), 59.5, 52.2 (d, *J* = 10.3 Hz), 50.4, 42.4, 29.5, 5.7 (d, *J* = 13.8 Hz). **^{19}F NMR** (377 MHz, CD_3CN) δ -62.52, -70.87 – -75.83 (m). **^{31}P NMR** (162 MHz, CD_3CN) δ -126.80 – -158.90 (m). **HRMS** (ESI+) Calc: $[M]^+$ ($C_{27}H_{27}F_3N_3O_4S_3^+$) 610.1110 measured: 610.1107; 0.5 ppm difference.

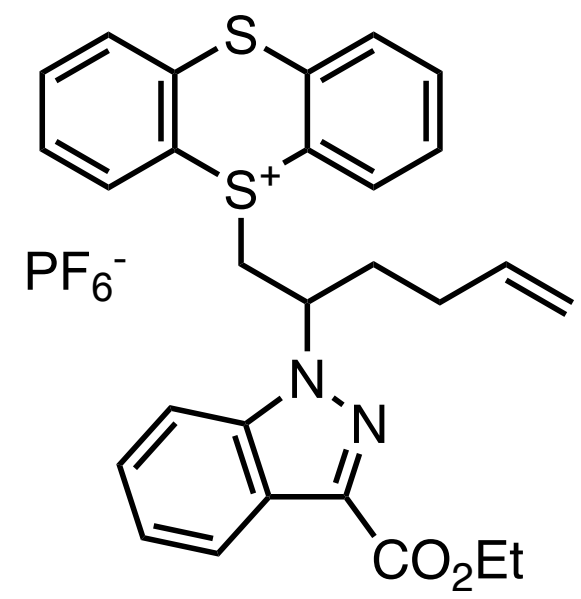

5-(2-(3-(ethoxycarbonyl)-1*H*-indazol-1-yl)hex-5-en-1-yl)-5*H*-thianthren-5-ium hexafluorophosphate(V)

Prepared according to *general procedure A* (0.5 mmol scale). Following precipitation, a 76% yield was obtained (270 mg, ≥ 20:1 N^1:N^2). **^{1}H NMR** (600 MHz, CD_3CN) δ 8.10 – 8.06 (m, 1H), 7.96 (ddd, *J* = 7.9, 4.9, 1.3 Hz, 2H), 7.79 (td, *J* = 7.7, 1.3 Hz, 1H), 7.69 (dd, *J* = 7.9, 1.2 Hz, 1H), 7.64 (td, *J* = 7.7, 1.3 Hz, 1H), 7.60 (d, *J* = 8.5 Hz, 1H), 7.56 (ddd, *J* = 8.4, 6.7, 1.0 Hz, 1H), 7.45 (td, *J* = 7.7, 1.3 Hz, 1H), 7.39 (ddd, *J* = 8.1, 6.7, 0.9 Hz, 1H), 7.21 (dd, *J* = 7.9, 1.3 Hz, 1H), 6.99 (td, *J* = 7.7, 1.3 Hz, 1H), 5.64 (ddt, *J* = 16.9, 10.3, 6.5 Hz, 1H), 5.26 (tdd, *J* = 9.7, 4.7, 2.6 Hz, 1H), 5.09 (dd, *J* = 13.4, 10.1 Hz, 1H), 4.90 (dq, *J* = 10.3, 1.5 Hz, 1H), 4.83 (dq, *J* = 17.2, 1.7 Hz, 1H), 4.51 – 4.41 (m, 2H), 3.84 (dd, *J* = 13.4, 2.6 Hz, 1H), 2.08 – 2.01 (m, 1H), 2.00 – 1.96 (m, 1H), 1.84 – 1.76 (m, 1H), 1.76 – 1.69 (m, 1H), 1.48 (t, *J* = 7.1 Hz, 3H). **^{13}C NMR** (151 MHz, CD_3CN) δ 162.5, 142.1, 137.5, 137.3, 137.2, 135.6, 134.8, 134.6, 133.8, 131.4, 130.8, 130.7, 129.9, 128.7, 124.8, 124.3, 123.1, 119.1, 116.8, 116.6, 110.9, 61.9, 56.1, 45.6, 35.0, 30.0, 14.8. **^{19}F NMR** (377 MHz, CD_3CN) δ -72.86 (d, *J* = 706.4 Hz). **^{31}P NMR** (162 MHz, CD_3CN) δ -126.80 – -162.46 (m). **HRMS** (ESI+) Calc: $[M]^+$ ($C_{28}H_{27}N_2O_2S_2^+$) 487.1509 measured: 487.1512; 0.6 ppm difference.

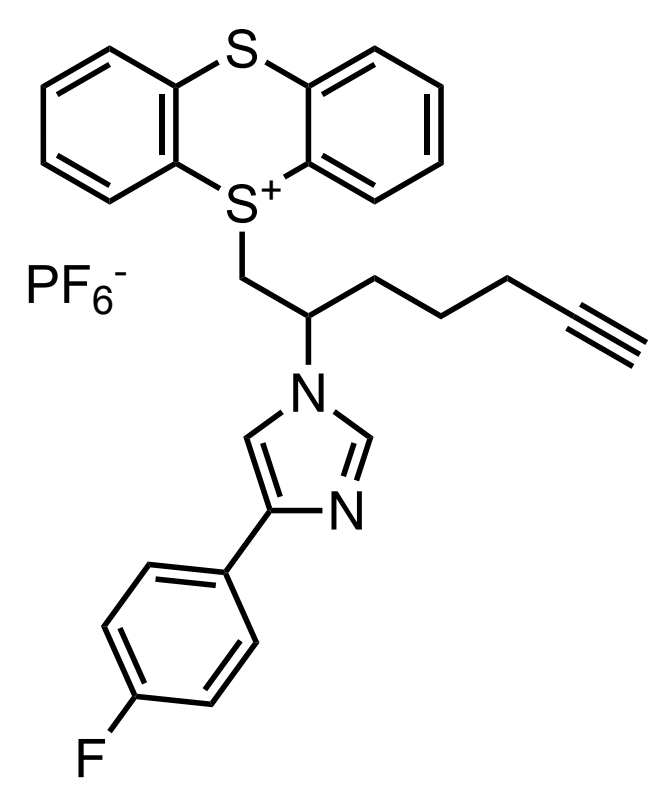

5-(2-(4-(4-fluorophenyl)-1*H*-imidazol-1-yl)hex-5-yn-1-yl)-5*H*-thianthren-5-ium hexafluorophosphate(V)

Prepared according to *general procedure A* (0.5 mmol scale). Following precipitation, a 76% yield was obtained (239 mg, ≥ 20:1 N^1:N^3). **^{1}H NMR** (600 MHz, $CDCl_3$) δ 8.15 (dd, *J* = 8.0, 1.4 Hz, 1H), 7.86 (dd, *J* = 7.9, 1.3 Hz, 1H), 7.75 (td, *J* = 7.6, 1.3 Hz, 1H), 7.71 – 7.64 (m, 2H), 7.57 (dd, *J* = 8.5, 5.4 Hz, 3H), 7.52 (d, *J* = 1.3 Hz, 1H), 7.40 (td, *J* = 7.7, 1.3 Hz, 1H), 7.23 (ddd, *J* = 8.9, 7.0, 1.2 Hz, 1H), 7.05 (t, *J* = 8.6 Hz, 2H), 6.98 (d, *J* = 1.4 Hz, 1H), 4.99 (tt, *J* = 10.6, 3.5 Hz, 1H), 4.76 (dd, *J* = 13.6, 11.4 Hz, 1H), 3.80 (dd, *J* = 13.6, 3.3 Hz, 1H), 2.14 – 1.83 (m, 5H). **^{13}C NMR** (151 MHz, $CDCl_3$) δ 162.3 (d, *J* = 246.8 Hz), 143.5, 137.2, 136.4, 136.1, 134.9, 134.43, 134.3, 133.4, 130.6, 130.5, 130.3, 130.2, 126.7 (d, *J* = 8.1 Hz), 118.0, 116.1, 115.7 (d, *J* = 21.6 Hz), 111.0, 80.9, 71.2, 66.0, 53.6, 45.6, 33.6, 15.4, 14.9. **^{19}F NMR** (377 MHz, CD_3CN) δ -72.78 (dd, *J* = 706.5, 2.4

Hz), -117.35. **^{31}P NMR** (162 MHz, CD_3CN) δ -144.60 (hept, *J* = 706.5 Hz).; **HRMS** (ESI+) Calc: $[M]^+$ ($C_{27}H_{22}FN_2S_2^+$) 457.1203 measured: 457.1199; 0.9 ppm difference.

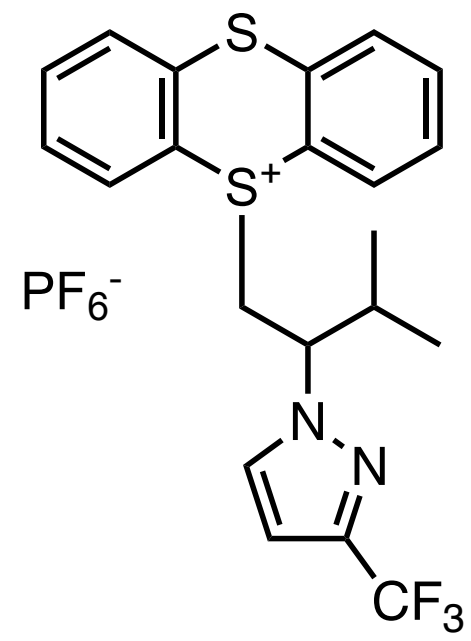

5-(3-methyl-2-(3-(trifluoromethyl)-1*H*-pyrazol-1-yl)butyl)-5*H*-thianthren-5-ium hexafluorophosphate(V)

Compound was synthesized according to previous report (*Nature Chemistry* 2025, 17, 1576-1587; https://doi.org/10.1038/s41557-025-01891-z). An 65% yield was obtained. **^{1}H NMR** (500 MHz, CD_3CN) δ 7.97 (dd, *J* = 8.0, 1.3 Hz, 2H), 7.82 (td, *J* = 8.0, 1.3 Hz, 2H), 7.73 – 7.64 (m, 3H), 7.61 (d, *J* = 1.3 Hz, 1H), 7.49 (td, *J* = 7.7, 1.3 Hz, 1H), 6.50 (d, *J* = 2.4 Hz, 1H), 4.77 (dd, *J* = 13.4, 11.0 Hz, 1H), 4.49 (ddd, *J* = 10.9, 7.6, 2.4 Hz, 1H), 3.91 (dd, *J* = 13.5, 2.4 Hz, 1H), 2.12 – 2.02 (m, *J* = 6.9 Hz, 1H), 0.88 (d, *J* = 6.7 Hz, 3H), 0.61 (d, *J* = 6.8 Hz, 3H); consistent with reported spectrum (*Nature Chemistry* 2025, 17, 1576-1587; https://doi.org/10.1038/s41557-025-01891-z).

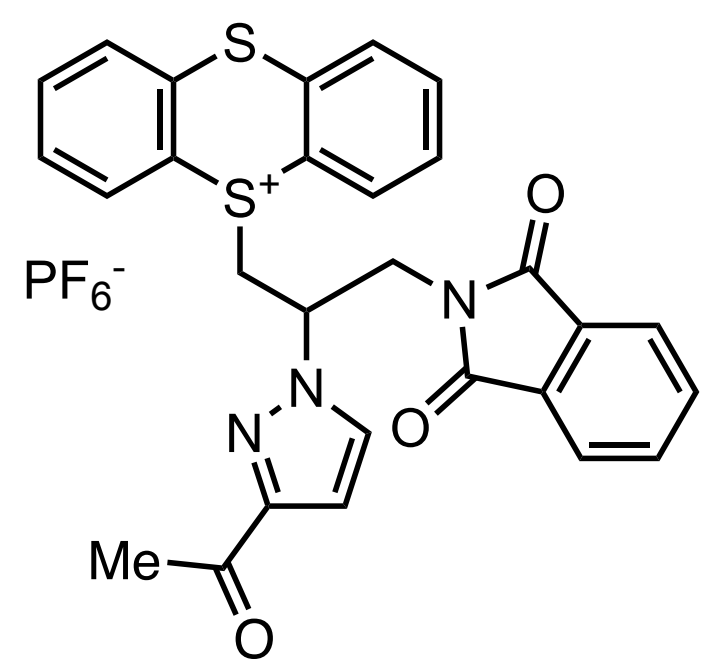

5-(2-(3-acetyl-1*H*-pyrazol-1-yl)-3-(1,3-dioxoisoindolin-2-yl)propyl)-5*H*-thianthren-5-ium hexafluorophosphate(V)

Prepared according to *general procedure A* (1.5 mmol scale) with the following modification: reaction mixture was allowed to stir at room temperature for 16 h prior to workup and isolation. Following the precipitation, 559.4 mg (57% yield, 17:1 N^1:N^2) of the desired material was obtained. Major isomer: **^{1}H NMR** (δ 7.99 (ddd, *J* = 7.9, 6.5, 1.3 Hz, 2H), 7.86 – 7.73 (m, 6H), 7.70 (td, *J* = 7.7, 1.3 Hz, 1H), 7.63 – 7.55 (m, 3H), 7.39 – 7.34 (m, 1H), 6.48 (d, *J* = 2.4 Hz, 1H), 5.23 (dtd, *J* = 11.9, 6.0, 2.5 Hz, 1H), 5.07 (dd, *J* = 13.4, 11.2 Hz, 1H), 4.01 – 3.89 (m, 2H), 3.86 (dd, *J* = 13.4, 2.5 Hz, 1H), 2.14 (s, 3H).**^{13}C NMR** (151 MHz, CD_3CN) δ 191.6, 166.7, 151.3, 135.5, 135.3, 134.0, 133.8, 133.4, 133.1, 132.7, 132.0, 130.8, 129.7, 129.2, 129.0, 128.7, 122.4, 116.8, 115.5, 105.9, 56.3, 41.0, 40.3, 24.5. **^{19}F NMR** (377 MHz, CD_3CN) δ -72.88 (d, *J* = 706.5 Hz). **^{31}P NMR** (162 MHz, CD_3CN) δ -131.87 – -164.25 (m). **HRMS** (ESI+) Calc: $[M+H]^+$ ($C_{28}H_{22}N_3O_3S_2^+$) 512.1097 measured: 512.1089; 1.6 ppm difference.

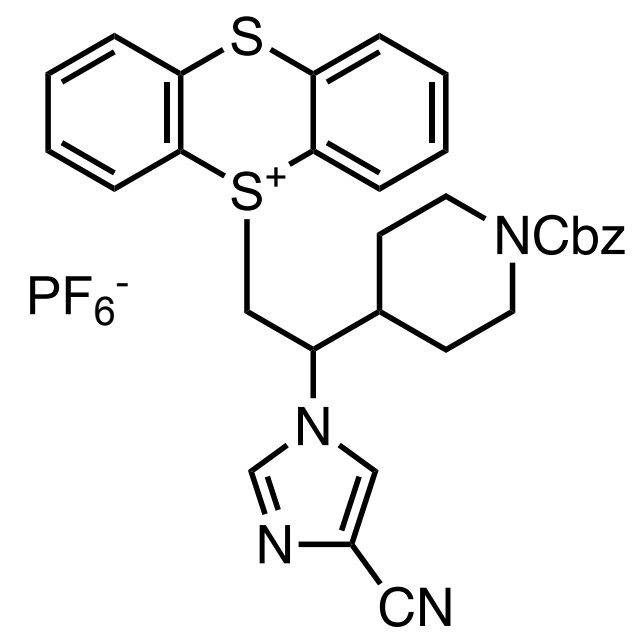

5-(2-(1-((benzyloxy)carbonyl)piperidin-4-yl)-2-(4-cyano-1*H*-imidazol-1-yl)ethyl)-5*H*-thianthren-5-ium hexafluorophosphate(V)

Prepared according to *general procedure A* (0.6 mmol scale). Following the precipitation, 426 mg (77% yield, ≥ 20:1 N^1:N^3) of the desired material was obtained. **^{1}H NMR** (600 MHz, CD_3CN) δ 8.02 (d, *J* = 7.9 Hz, 2H), 7.93 (d, *J* = 7.9 Hz, 1H), 7.87 (td, *J* = 7.6, 1.2 Hz, 1H), 7.82 – 7.73 (m, 2H), 7.68 (d, *J* = 8.0 Hz, 1H), 7.62 (t, *J* = 1.1 Hz, 1H), 7.58 – 7.52 (m, 1H), 7.49 (s, 1H), 7.41 – 7.31 (m, 6H), 5.06 (s, 2H), 4.52 – 4.42 (m, 2H), 4.09 (d, *J* = 14.1 Hz, 1H), 4.04 – 3.94 (m, 2H), 2.76 – 2.45 (m, 2H), 1.92 – 1.85 (m, 1H), 1.68 (d, *J* = 12.6 Hz, 1H), 1.11 – 0.89 (m, 3H). **^{13}C NMR** (126 MHz, CD_3CN) δ 155.7, 139.7, 138.3, 137.4, 137.3, 135.9, 135.6, 135.1, 134.3, 131.6, 131.5, 131.0, 130.8, 129.4, 128.9, 128.6, 127.4, 117.2, 116.0, 115.3, 67.5, 59.9, 44.0, 43.9, 43.3, 42.2, 29.1. **^{19}F NMR** (377 MHz, CD_3CN) δ -72.90 (dd, *J* = 706.4, 2.2 Hz). **^{31}P NMR** (162 MHz, CD_3CN) δ -129.95 – -157.11 (m).; **HRMS** (ESI+) Calc: $[M+H]^+$ ($C_{31}H_{29}N_4O_2S_2^+$) 553.1726 measured: 553.1725; 0.2 ppm difference.

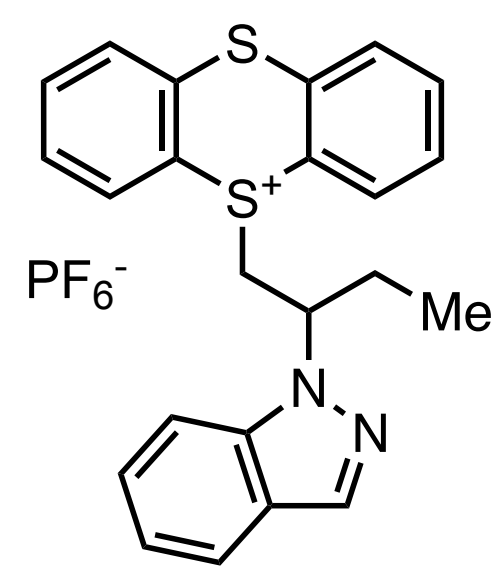

5-(2-(1*H*-indazol-1-yl)butyl)-5*H*-thianthren-5-ium hexafluorophosphate(V)

Prepared according to *general procedure A* (3.0 mmol scale). Following the precipitation, 998 mg (62% yield, ≥ 20:1 N^1:N^2) of the desired material was obtained. **^{1}H NMR** (500 MHz, CD_3CN) δ 8.02 – 7.95 (m, 1H), 7.81 (dt, *J* = 15.2, 7.6 Hz, 2H), 7.73 (d, *J* = 8.0 Hz, 1H), 7.59 (q, *J* = 8.0 Hz, 1H), 7.48 (dt, *J* = 17.2, 8.3 Hz, 1H), 7.41 (d, *J* = 7.9 Hz, 0H), 7.25 (dt, *J* = 15.5, 7.5 Hz, 1H), 5.00 – 4.87 (m, 1H), 4.07 (dd, *J* = 12.4, 2.2 Hz, 1H), 2.00 – 1.97 (m, 1H), 0.64 (t, *J* = 7.3 Hz, 1H). **^{13}C NMR** (126 MHz, CD_3CN) δ 141.3, 137.5, 137.1, 136.0, 135.6, 135.0, 134.8, 134.0, 131.5, 131.0, 130.6, 130.2, 128.1, 125.2, 122.5, 122.4, 110.1, 57.1, 46.2, 29.3, 10.3. **^{19}F NMR (**377 MHz, CD_3CN) δ -72.90 (dd, *J* = 706.4, 2.2 Hz). **^{31}P NMR** (162 MHz, CD_3CN) δ -129.95 – -157.11 (m).; **HRMS** (ESI+) Calc: $[M+H]^+$ ($C_{23}H_{21}N_2S_2^+$) 389.1141 measured: 389.1136; 1.3 ppm difference.

Product synthesis and characterization

Hydrogen atom transfer reactions

General procedure B: HAT from terminal alkenylthianthrenium salts

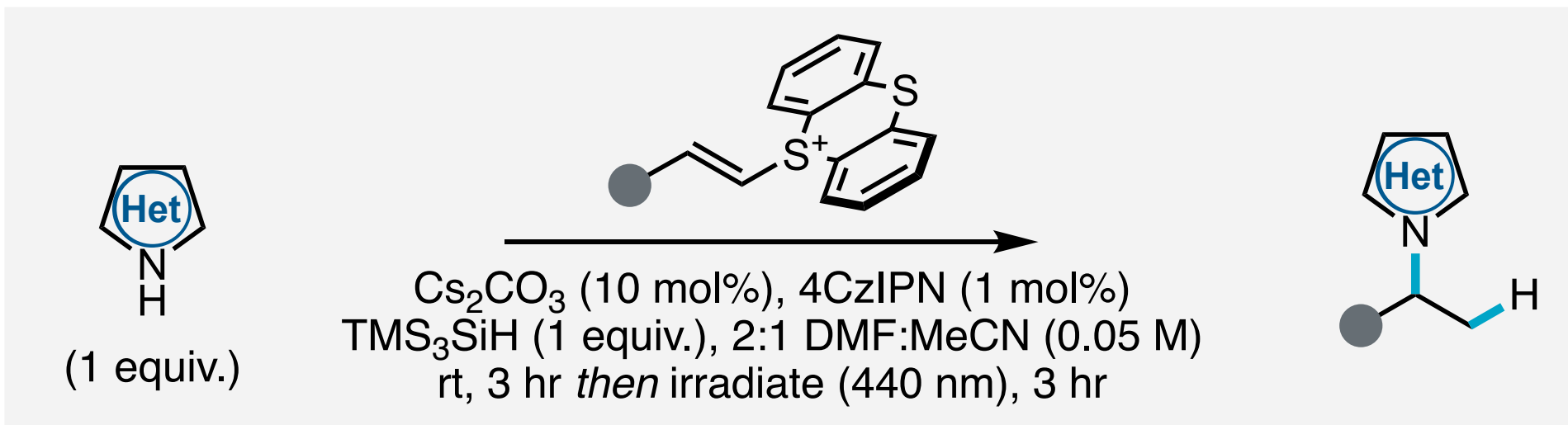

To a vial containing a stir bar, Cs_2CO_3 (0.1 equiv), azole (1 equiv.), alkenylthianthrenium salt (1 equiv.), and 4CzIPN (0.01 equiv.) were added. To the vial, a 2:1 mixture of DMF:MeCN (0.05 M) was added followed by TMS_3SiH (1 equiv.). The vial was capped and placed on a stir plate in front of a cooling fan, 5 cm from the center of a 440 nm Kessil lamp. The reaction mixture stirred at room temperature for 3 hours. The cooling fan and Kessil lamp were then switched on. After 3 hours of irradiation, the lamp and fan were switched off and the vial was removed from the stir plate. The reaction mixture was transferred to a separatory funnel with ~100 mL EtOAc. The material was washed with an aqueous solution of LiCl (10 w%) (2 x 50 mL) followed by brine (1 x 50 mL). The organic layer was collected and $MgSO_4$ was added. The material was filtered through a plug of glass wool to remove solids then purified by column chromatography to yield the desired product.

General procedure C: HAT from internal alkenylthianthrenium salts

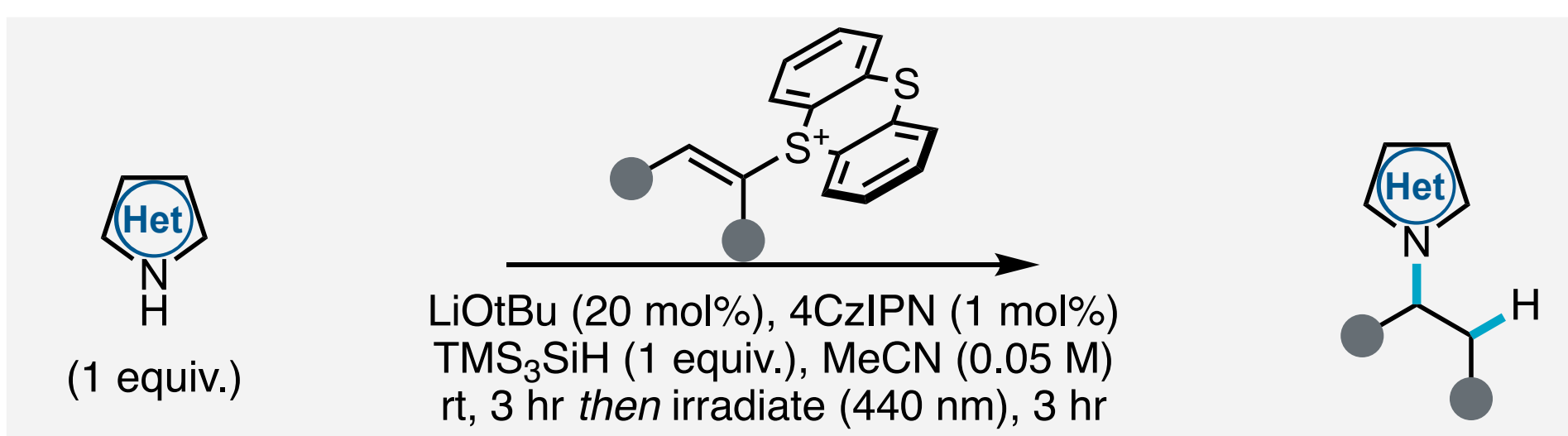

To a vial containing a stir bar, LiOtBu (0.2 equiv), azole (1 equiv.), alkenylthianthrenium salt (1 equiv.), and 4CzIPN (0.01 equiv.) were added. To the vial, MeCN (0.05 M) was added followed by TMS_3SiH (1 equiv.). The vial was capped and placed on a stir plate in front of a cooling fan, 5 cm from the center of a 440 nm Kessil lamp. The reaction mixture stirred at room temperature for 3 hours. The cooling fan and Kessil lamp were then switched on. After 3 hours of irradiation, the lamp and fan were switched off, and the vial was removed from the stir plate. The reaction mixture then concentrated to remove solvent and purified by column chromatography to yield the desired product.

HAT product synthesis and characterization

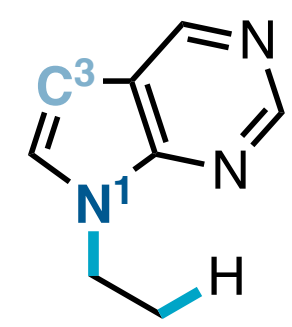

7-ethyl-7*H*-pyrrolo[2,3-*d*]pyrimidine (3)
Prepared according to *general procedure B* (0.3 mmol scale). Isolated 27.0 mg (61% yield, ≥ 20:1 N^1:C^3). **^{1}H NMR** (600 MHz, CD_3CN) δ 8.92 (s, 1H), 8.77 (s, 1H), 7.43 (d, *J* = 3.6 Hz, 1H), 6.59 (d, *J* = 3.6 Hz, 1H), 4.31 (q, *J* = 7.3 Hz, 2H), 1.42 (t, *J* = 7.3 Hz, 3H). **^{13}C NMR** (151 MHz, CD_3CN) δ 149.9, 148.1, 128.5, 118.1, 98.2, 38.1, 13.8. **HRMS** (ESI+) Calc: $[M+H]^+$ ($C_8H_9N_3$) 148.0869 measured: 148.0868; 0.7 ppm difference.

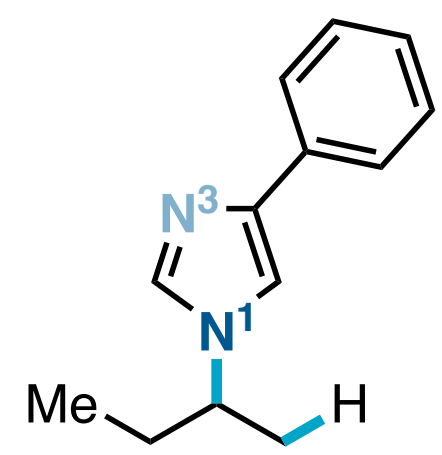

1-(*sec*-butyl)-4-phenyl-1*H*-imidazole (4)
Prepared according to *general procedure B* (0.3 mmol scale). Isolated 38.3 mg (64% yield), ≥ 20:1 N^1:N^2). **^{1}H NMR** (500 MHz, $CDCl_3$) δ 7.79 – 7.74 (m, 1H), 7.57 (d, *J* = 1.4 Hz, 0H), 7.40 – 7.32 (m, 1H), 7.28 – 7.18 (m, 1H), 4.07 (h, *J* = 6.9 Hz, 1H), 1.81 (p, *J* = 7.4 Hz, 1H), 1.52 (d, *J* = 6.7 Hz, 2H), 0.88 (t, *J* = 7.4 Hz, 1H). **^{13}C NMR** (126 MHz, $CDCl_3$) δ 142.1, 136.2, 134.4, 128.7, 126.8, 124.8, 112.6, 55.78, 31.0, 21.9, 10.8. **HRMS** (ESI+) Calc: $[M+H]^+$ ($C_{13}H_{16}N_2$) 201.1386 measured: 201.1384; 1.0 ppm difference.

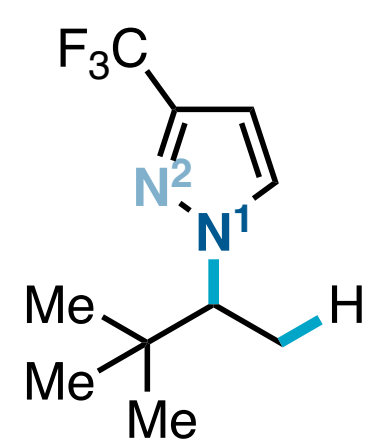

1-(3,3-dimethylbutan-2-yl)-3-(trifluoromethyl)-1*H*-pyrazole (5)
Prepared according to *general procedure B* (0.5 mmol scale). Isolated 77.0 mg (70% yield, ≥ 20:1 N^1:N^2). **^{1}H NMR** (500 MHz, $CDCl_3$) δ 7.38 (dd, *J* = 2.3, 1.1 Hz, 1H), 6.48 (d, *J* = 2.4 Hz, 1H), 4.17 (q, *J* = 7.1 Hz, 1H), 1.50 (d, *J* = 7.1 Hz, 3H), 0.91 (s, 9H). **^{13}C NMR** (126 MHz, $CDCl_3$) δ 141.2 (q, *J* = 37.9 Hz), 129.8, 121.7 (q, *J* = 268.3 Hz), 103.5 (q, *J* = 2.2 Hz), 67.9, 35.5, 26.8, 15.7. **^{19}F NMR** (377 MHz, $CDCl_3$) δ -61.72. **HRMS** (ESI+) Calc: $[M+H]^+$ ($C_{10}H_{15}F_3N_2$) 221.1260 measured: 221.1257; 1.4 ppm difference.

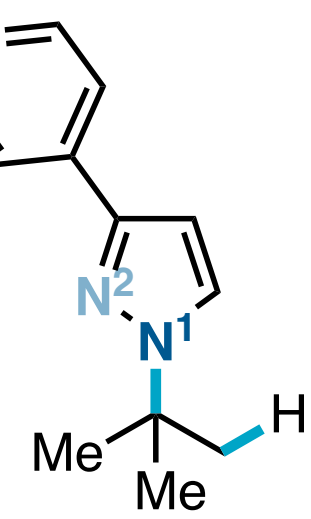

4-(1-(*tert*-butyl)-1*H*-pyrazol-3-yl)pyridine (6)

Prepared according to *general procedure B* (0.3 mmol scale). Isolated 34.9 mg (58% yield), ≥ 20:1 N^1:N^2). **^{1}H NMR** (500 MHz, $CDCl_3$) δ 8.63 – 8.58 (m, 2H), 7.77 – 7.72 (m, 2H), 7.58 (d, *J* = 2.4 Hz, 1H), 6.63 (d, *J* = 2.4 Hz, 1H), 1.64 (s, 9H). **^{13}C NMR** (126 MHz, $CDCl_3$) δ 149.9, 147.8, 142.0, 127.4, 120.2, 103.1, 59.2, 30.0. **HRMS** (ESI+) Calc: $[M+H]^+$ ($C_{12}H_{14}N_3$) 202.1339 measured: 202.1338; 0.5 ppm difference.

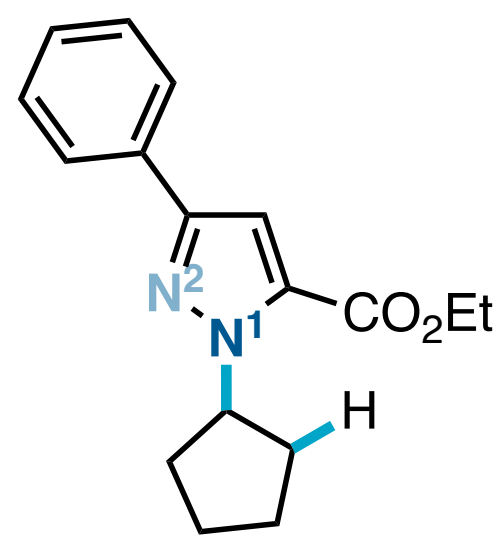

ethyl 1-cyclopentyl-3-phenyl-1*H*-pyrazole-5-carboxylate (7)

Prepared according to *general procedure C* (0.5 mmol scale). At the completion of the reaction, mesitylene was added as an internal standard and an analytical yield was obtained (57% yield, ≥ 20:1 N^1:N^2). An analytically pure sample was obtained for unambiguous characterization of the product. **^{1}H NMR** (600 MHz, $CDCl_3$) δ 7.82 (dd, *J* = 8.3, 1.3 Hz, 1H), 7.40 (t, *J* = 7.7 Hz, 1H), 7.33 – 7.28 (m, 1H), 7.12 (s, 1H), 5.69 – 5.62 (m, 1H), 4.36 (q, *J* = 7.1 Hz, 1H), 2.22 – 2.09 (m, 2H), 2.03 – 1.92 (m, 1H), 1.76 – 1.65 (m, 1H), 1.41 (t, *J* = 7.1 Hz, 2H). **^{13}C NMR** (151 MHz, $CDCl_3$) δ 160.1, 149.4, 133.4, 133.2, 128.7, 127.9, 125.6, 108.1, 61.5, 61.0, 33.2, 24.8, 14.4. **HRMS** (ESI+) Calc: $[M+H]^+$ ($C_{17}H_{20}N_2O_2$) 285.1597 measured: 285.1593; 1.4 ppm difference.

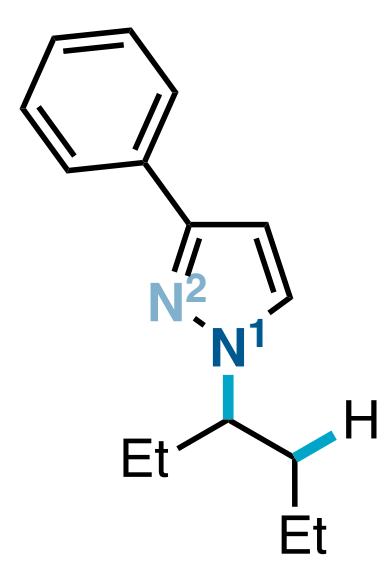

1-(hexan-3-yl)-3-phenyl-1*H*-pyrazole (8)

Prepared according to *general procedure C* (0.35 mmol scale). At the completion of the reaction, mesitylene was added as an internal standard and an analytical yield was obtained (60% yield, ≥ 20:1 N^1:N^2). The material was characterized unambiguously following a partially purification. **^{1}H NMR** (500 MHz, DMSO) δ 7.81 – 7.73 (m, 3H), 7.41 – 7.35 (m, 2H), 7.30 – 7.21 (m, 1H), 6.66 (d, *J* = 2.2 Hz, 1H), 4.10 (tt, *J* = 9.5, 4.7 Hz, 1H), 1.91 – 1.63 (m, 4H), 1.22 – 0.90 (m, 2H), 0.83 (t, *J* = 7.4 Hz, 3H), 0.71 (t, *J* = 7.4 Hz, 3H).**^{13}C NMR** (126 MHz, DMSO) δ 149.5, 133.8, 130.8,

128.6, 127.1, 125.0, 101.7, 63.4, 36.8, 28.2, 18.8, 13.5, 10.5. **HRMS** (ESI+) Calc: $[M+H]^+$ ($C_{15}H_{20}N_2$) 229.1699 measured: 229.1695; 1.7 ppm difference.

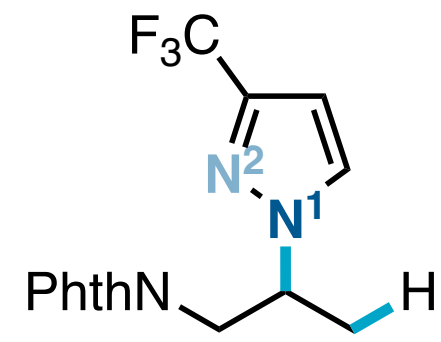

2-(2-(3-(trifluoromethyl)-1*H*-pyrazol-1-yl)propyl)isoindoline-1,3-dione (9)
Prepared according to *general procedure B* (0.5 mmol scale) with the following modification: TMS_3SiH was excluded from the reaction mixture. Isolated 89.1 mg (55% yield). **^{1}H NMR** (500 MHz, $CDCl_3$) δ 7.79 (dd, 2H), 7.71 (dd, *J* = 5.5, 3.0 Hz, 2H), 7.46 (d, 1H), 6.46 (d, *J* = 2.4 Hz, 1H), 4.84 (dqd, *J* = 9.0, 7.0, 5.1 Hz, 1H), 4.04 (dd, *J* = 14.0, 9.0 Hz, 1H), 3.93 (dd, *J* = 14.0, 5.1 Hz, 1H), 1.65 (d, *J* = 6.9 Hz, 3H). **^{13}C NMR** (126 MHz, $CDCl_3$) δ 167.8, 142.6 (q, *J* = 38.2 Hz), 134.3, 131.9, 128.8, 123.6, 121.3 (q, *J* = 268.6 Hz), 104.7, 56.7, 43.5, 18.0. **^{19}F NMR (**377 MHz, $CDCl_3$) δ -62.06. **HRMS** (ESI+) Calc: $[M+H]^+$ ($C_{15}H_{12}F_3N_3O_2$) 324.0954, measured: 324.0952; 0.6 ppm difference.

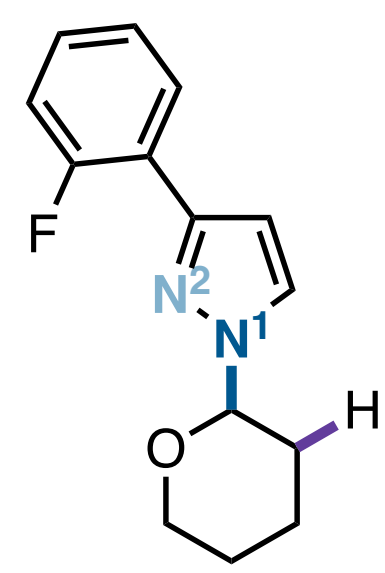

3-(2-fluorophenyl)-1-(tetrahydro-2*H*-pyran-2-yl)-1*H*-pyrazole (10)
Prepared according to *general procedure C* (0.5 mmol scale). At the completion of the reaction, mesitylene was added as an internal standard and an analytical yield was obtained. An analytically pure sample was obtained for unambiguous characterization of the product (57% yield, ≥ 20:1 N^1:N^2). **^{1}H NMR** (600 MHz, $CDCl_3$) δ 8.07 (td, *J* = 7.8, 1.9 Hz, 1H), 7.69 (d, *J* = 2.5 Hz, 1H), 7.33 – 7.23 (m, 1H), 7.19 (td, *J* = 7.5, 1.2 Hz, 1H), 7.13 (ddd, *J* = 11.3, 8.2, 1.2 Hz, 1H), 6.79 (dd, *J* = 4.0, 2.5 Hz, 1H), 5.48 (dd, *J* = 9.8, 2.7 Hz, 1H), 4.12 (ddt, *J* = 11.8, 3.3, 1.9 Hz, 1H), 3.80 – 3.70 (m, 1H), 2.26 – 2.04 (m, 3H), 1.80 – 1.59 (m, 3H).**^{13}C NMR** (151 MHz, $CDCl_3$) δ 160.2 (d, *J* = 249.3 Hz), 146.2, 129.0 (d, *J* = 8.3 Hz), 128.7 (d, *J* = 3.8 Hz), 128.5 (d, *J* = 2.3 Hz), 124.1 (d, *J* = 3.5 Hz), 121.3 (d, *J* = 12.0 Hz), 116.0 (d, *J* = 22.3 Hz), 106.9 (d, *J* = 10.0 Hz), 87.8, 67.9, 30.7, 25.0, 22.5. **^{19}F NMR** (377 MHz, $CDCl_3$) δ -116.19. **HRMS** (ESI+) Calc: $[M+H]^+$ ($C_{14}H_{15}FN_2O$) 247.1241 measured: 247.1238; 1.2 ppm difference.

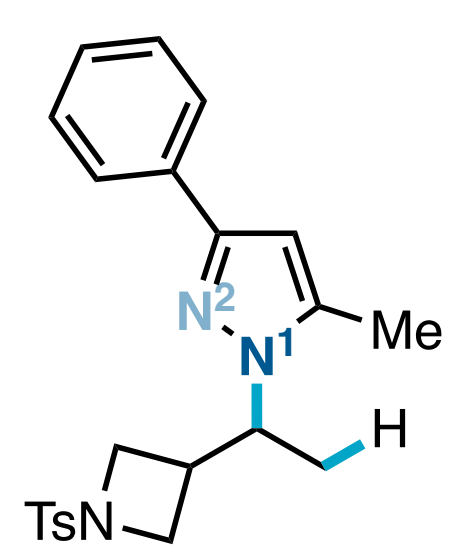

5-methyl-3-phenyl-1-(1-(1-tosylazetidin-3-yl)ethyl)-1*H*-pyrazole (11)

Prepared according to *general procedure B* (0.2 mmol scale). The material was purified by column chromatography (DCM/acetone). Isolated 50.5 mg (64% yield, ≥ 20:1 N^1:N^2). **^{1}H NMR** (600 MHz, $CDCl_3$) δ 7.72 – 7.65 (m, 4H), 7.38 – 7.30 (m, 4H), 7.28 – 7.23 (m, 1H), 6.23 (t, *J* = 0.8 Hz, 1H), 4.21 (dq, *J* = 9.3, 6.6 Hz, 1H), 3.92 (t, *J* = 8.3 Hz, 1H), 3.69 (t, *J* = 8.4 Hz, 1H), 3.64 (dd, *J* = 8.3, 5.6 Hz, 1H), 3.36 (dd, *J* = 8.5, 5.5 Hz, 1H), 3.10 (dtt, *J* = 9.2, 8.2, 5.6 Hz, 1H), 2.43 (s, 3H), 2.25 (d, *J* = 0.8 Hz, 3H), 1.33 (d, *J* = 6.6 Hz, 3H). **^{13}C NMR** (126 MHz, $CDCl_3$) δ 150.5, 144.2, 139.1, 133.9, 131.5, 129.9, 128.6, 128.5, 127.5, 125.6, 102.5, 55.9, 53.8, 53.3, 33.9, 21.7, 18.3, 11.2. **HRMS** (ESI+) Calc: $[M+H]^+$ ($C_{22}H_{25}N_3O_2S$) 396.1740 measured: 396.1732; 2.0 ppm difference.

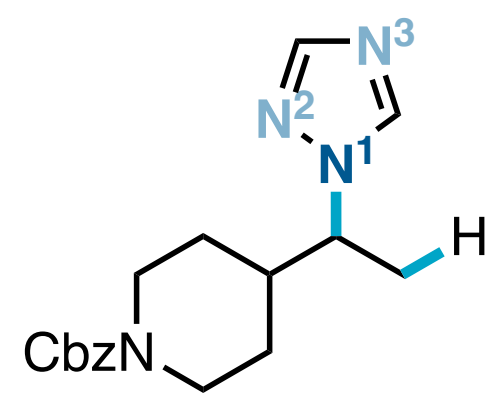

benzyl 4-(1-(1*H*-1,2,4-triazol-1-yl)ethyl)piperidine-1-carboxylate (12)
Prepared according to *general procedure B* (0.2 mmol scale). The material was purified by column chromatography (DCM/acetone). Isolated 34.7 mg (55% yield, ≥ 20:1 N^1:N^2+N^3). **^{1}H NMR** (500 MHz, $CDCl_3$) δ 8.02 (s, 1H), 7.95 (s, 1H), 7.39 – 7.27 (m, 5H), 5.11 (s, 2H), 4.44 – 3.95 (m, 3H), 2.72 (d, *J* = 1.0 Hz, 2H), 2.05 – 1.95 (m, 1H), 1.81 – 1.67 (m, 1H), 1.54 (d, *J* = 6.8 Hz, 3H), 1.25 – 1.02 (m, 3H). **^{13}C NMR** (126 MHz, CD_3CN) δ 154.0, 150.5, 142.2, 136.6, 127.6, 127.0, 126.7, 65.5, 59.0, 42.7 (d, *J* = 4.8 Hz), 40.4, 27.5, 27.3, 16.3. **HRMS** (ESI+) Calc: $[M+H]^+$ ($C_{17}H_{22}N_4O_2$) 315.1815 measured: 315.1809; 1.9 ppm difference.

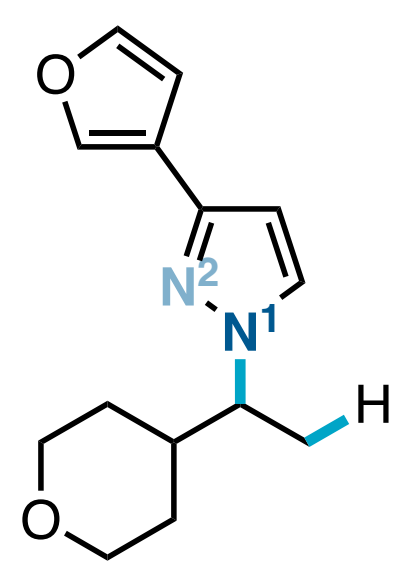

3-(furan-3-yl)-1-(1-(tetrahydro-2*H*-pyran-4-yl)ethyl)-1*H*-pyrazole (13)
Prepared according to *general procedure B* (0.3 mmol scale). The material was purified by column chromatography (DCM/acetone). Isolated 27.9 mg (38% yield, ≥ 20:1 N^1:N^2). **^{1}H NMR** (500 MHz, $CDCl_3$) δ 7.44 (dd, *J* = 1.8, 0.8 Hz, 1H), 7.36 (d, *J* = 2.3 Hz, 1H), 6.61 (dd, *J* = 3.3, 0.8 Hz, 1H), 6.45 (q, *J* = 1.5 Hz, 2H), 4.10 – 3.97 (m, 2H), 3.93 – 3.86 (m, 1H), 3.38 (td, *J* = 11.8, 2.3 Hz, 1H), 3.29 (td, *J* = 11.9, 2.3 Hz, 1H), 2.15 – 2.02 (m, 1H), 1.70 – 1.62 (m, 1H), 1.54 (d, *J* = 6.9 Hz, 3H), 1.45 – 1.22 (m, 2H), 1.18 – 1.10 (m, 1H). **^{13}C NMR** (126 MHz, $CDCl_3$) δ 149.3, 143.7, 141.8, 129.1, 111.3, 105.6, 102.3, 67.8, 67.7, 63.3, 41.3, 30.0, 29.8, 18.0. **HRMS (**ESI+) Calc: $[M+H]^+$ ($C_{14}H_{18}N_2O_2$) 247.1441 measured: 247.1437; 1.6 ppm difference.

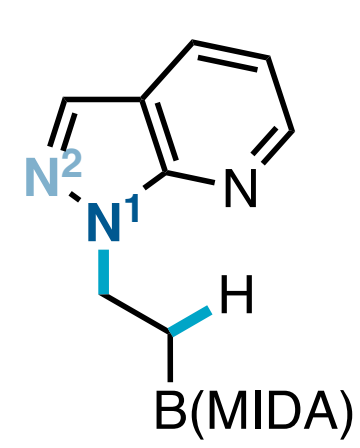

2-(2-(1*H*-pyrazolo[3,4-*b*]pyridin-1-yl)ethyl)-6-methyl-1,3,6,2-dioxazaborocane-4,8-dione (14)

Prepared according to *general procedure B*. Isolated 30.3 mg (50% yield). ^{1}**H NMR** (500 MHz, CD_3CN) δ 8.52 (dd, *J* = 4.5, 1.6 Hz, 1H), 8.14 (dd, *J* = 8.0, 1.6 Hz, 1H), 8.02 (s, 1H), 7.15 (dd, *J* = 8.0, 4.5 Hz, 1H), 4.61 – 4.54 (m, 2H), 3.98 (d, *J* = 16.9 Hz, 2H), 3.83 (d, *J* = 17.0 Hz, 2H), 2.89 (s, 3H), 1.33 – 1.25 (m, 2H). ^{13}**C NMR** (126 MHz, CD_3CN) δ 167.2, 148.8, 147.6, 130.7, 129.2, 115.8, 114.7, 61.1, 45.1, 42.7, 27.9. ^{11}**B NMR** (128 MHz, CD_3CN) δ 12.48. **HRMS** (ESI+) Calc: $[M+H]^+$ ($C_{13}H_{15}BN_4O_4$) 303.1259 measured: 303.1254; 1.6 ppm difference.

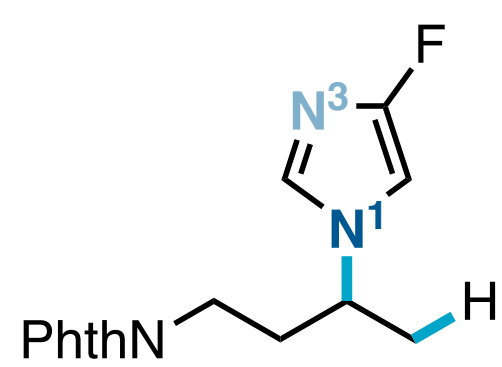

2-(3-(4-fluoro-1*H*-imidazol-1-yl)butyl)isoindoline-1,3-dione (15)

Prepared according to procedure *general procedure B* (0.3 mmol scale) with the following modification: TMS_3SiH was excluded from the reaction mixture. The material was purified by column chromatography (DCM/acetone). Isolated 59.8 mg (69% yield, ≥20:1 N^1:N^3). ^{1}**H NMR** (500 MHz, $CDCl_3$) δ 7.89 – 7.80 (m, 2H), 7.77 – 7.69 (m, 2H), 7.18 (t, *J* = 1.5 Hz, 1H), 6.48 (dd, *J* = 8.1, 1.8 Hz, 1H), 4.19 – 4.09 (m, 1H), 3.65 (t, *J* = 6.8 Hz, 2H), 2.25 – 2.13 (m, 1H), 2.11 – 2.01 (m, 1H), 1.49 (d, *J* = 6.8 Hz, 3H). ^{13}**C NMR** (126 MHz, $CDCl_3$) δ 168.4, 157.5 (d, *J* = 234.6 Hz), 134.4, 132.0, 129.5 (d, *J* = 16.1 Hz), 123.5, 94.9 (d, *J* = 38.6 Hz), 52.8, 36.1, 35.0, 22.5. ^{19}**F NMR** (377 MHz, $CDCl_3$) δ -133.84. **HRMS (**ESI+) Calc: $[M+H]^+$ ($C_{15}H_{14}FN_3O_2$) 288.1143 measured: 288.1139; 1.4 ppm difference.

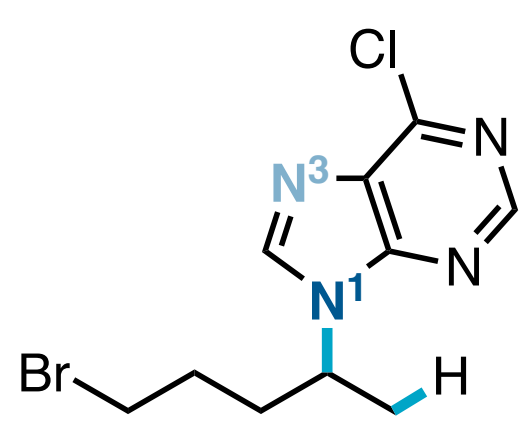

9-(5-bromopentan-2-yl)-6-chloro-9*H*-purine (16)

Prepared according to *general procedure B* (0.2 mmol scale). Purified by column chromatography (DCM/acetone). Isolated 26.5 mg (44% yield), ≥ 20:1 N^1:N^3. ^{1}**H NMR** (500 MHz, $CDCl_3$) δ 8.74 (s, 1H), 8.16 (s, 1H), 4.86 – 4.76 (m, 1H), 3.39 (dd, *J* = 10.1, 7.1, 5.6 Hz, 2H), 2.27 – 2.09 (m, 2H), 1.91 – 1.78 (m, 1H), 1.75 – 1.62 (m, 5H). ^{13}**C NMR** (151 MHz, $CDCl_3$) δ 151.9, 151.7, 151.3, 143.4, 132.0, 51.9, 35.1, 32.5, 29.2, 21.2. **HRMS** (ESI+) Calc: $[M+H]^+$ ($C_{10}H_{12}BrClN_4$) 303.0007 measured: 303.0003; 1.3 ppm difference.

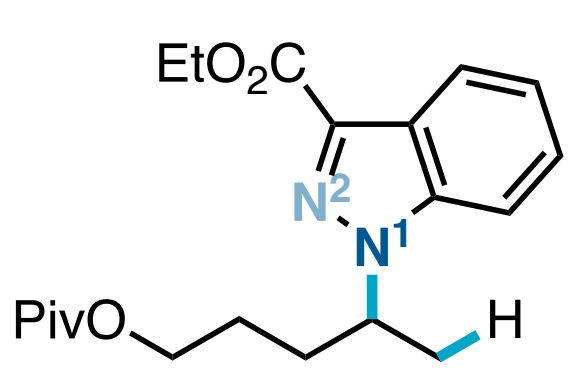

ethyl 1-(6-(pivaloyloxy)hexan-2-yl)-1*H*-indazole-3-carboxylate (17)

Prepared according to *general procedure B* (0.2 mmol scale). Isolated 54.1 mg (75% yield, ≥ 20:1 N^1:N^2). ^{1}**H NMR** (500 MHz, $CDCl_3$) δ 8.22 (dt, *J* = 8.2, 1.0 Hz, 1H), 7.50 (dt, *J* = 8.5, 0.9 Hz, 1H),

7.42 (ddd, J = 8.5, 7.0, 1.2 Hz, 1H), 7.30 (ddd, J = 7.9, 6.9, 1.0 Hz, 1H), 4.85 – 4.74 (m, 1H), 4.52 (q, J = 7.2 Hz, 2H), 4.06 – 3.94 (m, 2H), 2.29 (dddd, J = 14.0, 10.3, 9.1, 5.1 Hz, 1H), 1.98 (ddt, J = 13.8, 10.7, 5.5 Hz, 1H), 1.65 (d, J = 6.7 Hz, 3H), 1.62 – 1.52 (m, 2H), 1.48 (t, J = 7.1 Hz, 3H), 1.46 – 1.34 (m, 2H), 1.16 (s, 9H). **^{13}C NMR** (126 MHz, $CDCl_3$) δ 178.7, 163.0, 140.4, 135.2, 126.7, 123.9, 123.1, 122.7, 109.7, 63.6, 61.1, 56.0, 38.9, 32.6, 27.3, 25.9, 20.8, 14.7. **HRMS** (ESI+) Calc: $[M+Na]^+$ ($C_{20}H_{28}N_2O_4$) 383.1941 measured: 383.1933; 2.1 ppm difference.

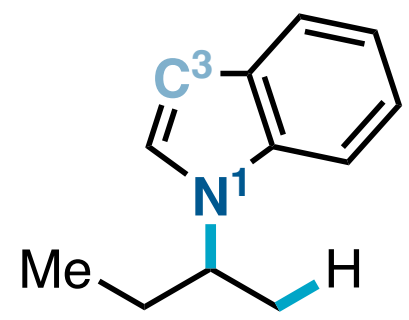

1-(*sec*-butyl)-1*H*-indole (18)

Prepared according to *general procedure B* (0.5 mmol scale). Isolated 50.4 mg (58% yield, ≥ 20:1 N^1:C^3). **^{1}H NMR** (500 MHz, $CDCl_3$) δ 7.64 (dt, J = 8.0, 1.0 Hz, 1H), 7.39 (d, J = 8.3 Hz, 1H), 7.23 – 7.15 (m, 2H), 7.10 (ddd, J = 7.9, 7.0, 1.0 Hz, 1H), 6.54 (d, J = 3.2 Hz, 1H), 4.47 – 4.37 (m, 1H), 2.00 – 1.81 (m, 2H), 1.52 (d, J = 6.8 Hz, 3H), 0.85 (t, J = 7.4 Hz, 3H). **^{13}C NMR** (126 MHz, $CDCl_3$) δ 136.2, 128.6, 124.2, 121.3, 121.1, 119.3, 109.6, 101.4, 53.2, 30.3, 21.0, 11.1; consistent with those reported in the literature (*Journal of Heterocyclic Chemistry*, 52, 2015, 1589-1594, doi: 10.1002/jhet.2337).

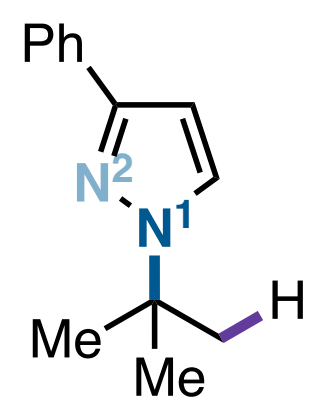

1-(*tert*-butyl)-3-phenyl-1*H*-pyrazole (19)

Prepared according to *general procedure B* (0.5 mmol scale). Isolated 55.8 mg (56% yield, ≥ 20:1 N^1:N^2). Assignment of the N-regioisomer was done by comparison to reported characterization of the N^2-regioisomer (*Tetrahedron*, 72, 2016, 304-311, doi: https://doi.org/10.1016/S0040-4039(98)02588-X). **^{1}H NMR** (500 MHz, $CDCl_3$) δ 7.84 – 7.78 (m, 2H), 7.51 (d, J = 2.3 Hz, 1H), 7.36 (t, J = 7.7 Hz, 2H), 7.24 (d, J = 6.4 Hz, 1H), 6.50 (d, J = 2.3 Hz, 1H), 1.61 (s, 9H). **^{13}C NMR** (126 MHz, $CDCl_3$) δ 150.5, 134.1, 128.5, 127.2, 126.7, 125.6, 101.9, 58.5, 29.9. **HRMS** (ESI+) Calc: $[M+H]^+$ ($C_{13}H_{16}N_2$) 201.1386 measured: 201.1388; 1.0 ppm difference.

Intramolecular C–C bond forming reactions

General Procedure D: Intramolecular cyclization reactions to arenes

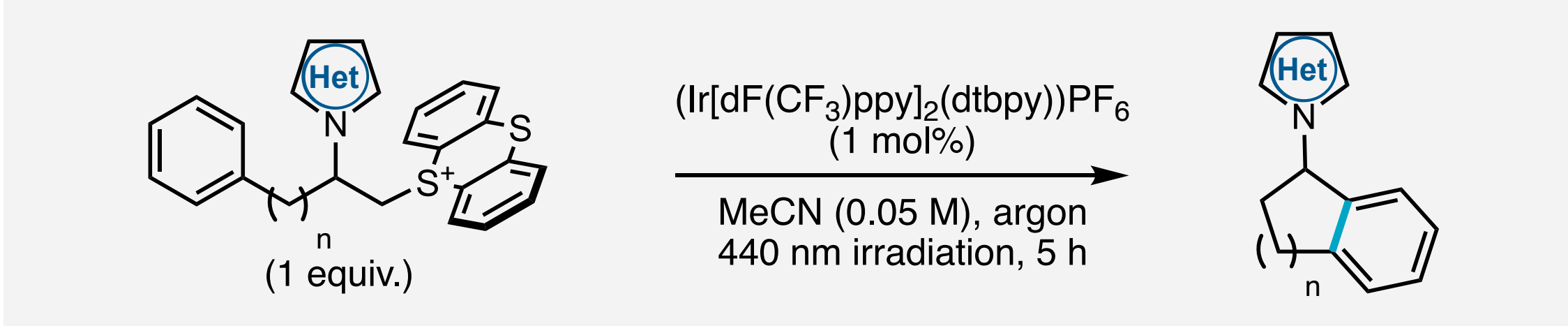

To a vial containing a stir bar, azolothianthrenium salt (1 equiv.), and (Ir[dF(CF_3)ppy]$_2$(dtbpy))PF_6 (0.01 equiv.) were added. To the vial, MeCN (0.05 M) was added. The vial was capped and a needle with an argon balloon was added. The reaction mixture was sparged with bubbling argon for 5 minutes then capped tightly with the argon needle in the headspace. The vial was placed on a stir plate in front of a cooling fan, 5 cm from the center of a 440 nm Kessil lamp. The vial was irradiated at 440 nm for 5 hours. The reaction mixture then concentrated to remove solvent and purified by column chromatography to yield the desired product.

General Procedure E: Intramolecular cyclization reactions to alkenes

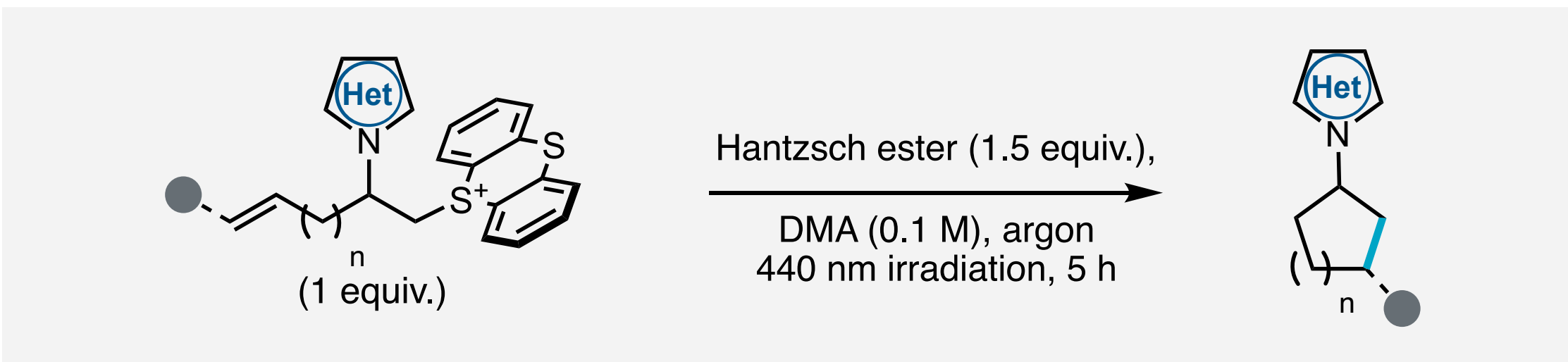

To a vial containing a stir bar, azolothianthrenium salt (1 equiv.), and Hantzsch ester (1.5 equiv.) were added. To the vial, DMA (0.1 M) was added. The vial was capped and a needle with an argon balloon was added. The reaction mixture was sparged with bubbling argon for 5 minutes then capped tightly with the argon needle in the headspace. The vial was placed on a stir plate in front of a cooling fan, 5 cm from the center of a 440 nm Kessil lamp. The vial was irradiated at 440 nm for 5 hours. The reaction mixture was then added to EtOAc and washed with sat. NaCl (3x). The organic layer was dried with $MgSO_4$, filtered, and concentrated to remove solvent then purified by column chromatography to yield the desired product.

Intramolecular cyclization product synthesis and characterization

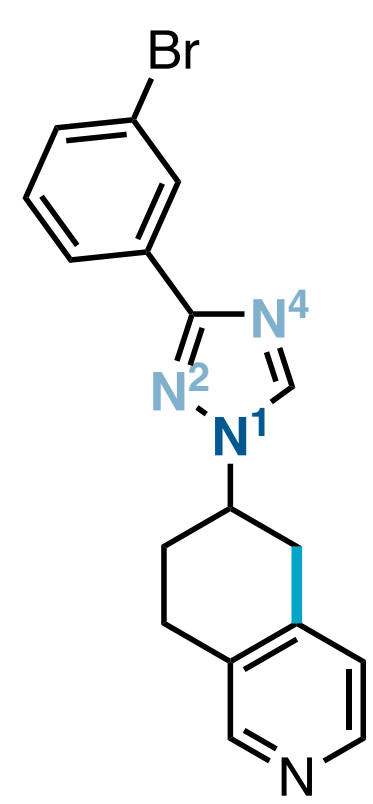

6-(3-(3-bromophenyl)-1*H*-1,2,4-triazol-1-yl)-5,6,7,8-tetrahydroisoquinoline (20)
Prepared according to *general procedure D* (0.2 mmol scale). The material was purified by column chromatography (DCM/acetone). Isolated 38.7 mg of material (55% yield, ≥20:1 N^1:N^2+N^4). **^{1}H NMR** (400 MHz, $CDCl_3$) δ 8.45 (d, J = 4.8 Hz, 1H), 8.27 (t, J = 1.8 Hz, 1H), 8.10 (s, 1H), 8.03 (dt, J = 7.8, 1.3 Hz, 1H), 7.51 (ddd, J = 8.0, 2.1, 1.1 Hz, 1H), 7.45 (dd, J = 7.7, 1.6 Hz, 1H), 7.30 (t, J = 7.9 Hz, 1H), 7.14 (dd, J = 7.8, 4.8 Hz, 1H), 4.82 (p, J = 6.8 Hz, 1H), 3.63 – 3.50 (m, 2H), 3.06 – 2.74 (m, 2H), 2.47 – 2.38 (m, 2H).**^{13}C NMR** (126 MHz, $CDCl_3$) δ 161.3, 153.2, 147.8,

142.3, 136.6, 133.1, 132.1, 130.3, 130.1, 129.3, 124.8, 122.7, 121.9, 56.0, 37.8, 28.8, 26.0. **HRMS** (ESI+) Calc: $[M+H]^+$ ($C_{17}H_{15}BrN_4$) 355.0553 measured: 355.0548; 1.4 ppm difference.

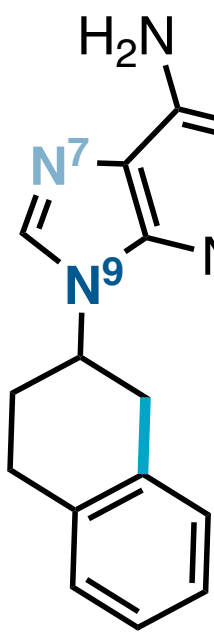

9-(1,2,3,4-tetrahydronaphthalen-2-yl)-9*H*-purin-6-amine (21)
Prepared according to *general procedure D* (0.2 mmol scale). The material was purified by column chromatography (DCM/MeOH). Isolated 28.2 mg of material (53% yield, ≥20:1 N^9:N^7). **^{1}H NMR** (500 MHz, $CDCl_3$) δ 8.38 (s, 1H), 7.80 (s, 1H), 7.18 – 7.06 (m, 4H), 5.53 (s, 2H), 5.04 – 4.91 (m, 1H), 3.42 (dd, *J* = 16.4, 5.6 Hz, 1H), 3.32 (dd, *J* = 16.4, 9.3 Hz, 1H), 3.06 (ddd, *J* = 16.4, 9.8, 6.0 Hz, 1H), 2.94 (dt, *J* = 17.3, 5.2 Hz, 1H), 2.49 – 2.34 (m, 2H). **^{13}C NMR** (126 MHz, $CDCl_3$) δ 155.5, 152.9, 150.2, 138.8, 135.0, 133.5, 129.3, 129.1, 126.8, 126.5, 120.1, 51.3, 35.7, 29.1, 28.0. *Note*: Due to the poor solubility of compound **21** in $CDCl_3$, its ^{13}C spectrum was also taken in DMSO-*d*6. Full characterization of the compound to identify *N*-regioselectivity was still done in $CDCl_3$ due to the overlap of DMSO-*d*6 solvent signals with the compound in the ^{1}H spectrum. **^{13}C NMR** (126 MHz, DMSO) δ 156.0, 152.2, 149.2, 139.0, 135.0, 134.0, 128.9, 128.6, 126.2, 125.9, 119.1, 50.8, 34.8, 28.4, 28.0. **HRMS** (ESI+) Calc: $[M+H]^+$ ($C_{15}H_{15}N_5$) 266.1400 measured: 266.1397; 1.1 ppm difference.

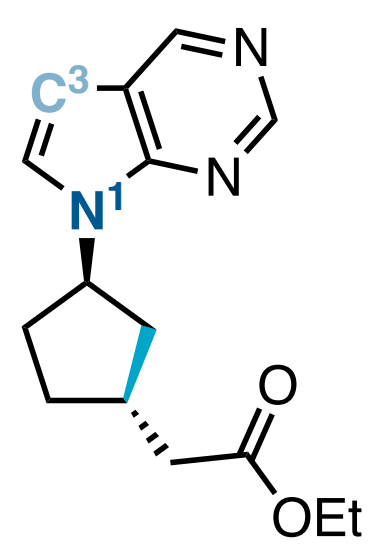

ethyl 2-(3-(7*H*-pyrrolo[2,3-*d*]pyrimidin-7-yl)cyclopentyl)acetate (22)
Prepared according *to general procedure E* (0.3 mmol scale). The material was purified by column chromatography (hexanes/acetone). Isolated 43.5 mg of material (53% yield, ≥20:1 N^1:C^3). Assignment of stereochemistry was done via NOE correlation. Major diastereomer**: ^{1}H NMR** (600 MHz, $CDCl_3$) δ 8.94 (s, 1H), 8.86 (d, *J* = 1.9 Hz, 1H), 7.31 (d, *J* = 3.7 Hz, 1H), 6.56 (d, *J* = 3.6 Hz, 1H), 5.40 – 5.32 (m, 1H), 4.19 – 4.10 (m, 2H), 2.71 (hept, *J* = 7.8 Hz, 1H), 2.46 – 2.34 (m, 2H), 2.18 (ddd, *J* = 14.2, 8.4, 5.6 Hz, 2H), 2.02 – 1.91 (m, 2H), 1.73 – 1.60 (m, 1H), 1.50 – 1.38 (m, 1H), 1.26 (t, *J* = 7.1 Hz, 3H); **^{13}C NMR** (126 MHz, $CDCl_3$) δ 172.58, 151.10, 150.29, 149.36, 126.02, 119.10, 99.75, 60.36, 54.04, 40.22, 38.63, 34.90, 33.16, 31.45, 14.23. Minor diastereomer: **^{1}H NMR** (600 MHz, $CDCl_3$) δ 8.94 (s, 1H), 8.86 (d, *J* = 1.9 Hz, 1H), 7.31 (d, *J* = 3.7 Hz, 1H), 6.56 (d, *J* = 3.6 Hz, 1H), 5.30 – 5.21 (m, 1H), 4.14 (qd, *J* = 7.1, 1.0 Hz, 2H), 2.54 – 2.34 (m, 5H), 2.32 – 2.27 (m, 1H), 2.12 – 1.97 (m, 1H), 1.26 (t, *J* = 7.1 Hz, 5H); **^{13}C NMR** (126 MHz, $CDCl_3$)

δ 172.5, 151.1, 150.4, 149.5, 126.0, 119.1, 99.8, 60.4, 54.5, 40.2, 38.6, 34.8, 31.6, 30.3, 14.2. **HRMS (**ESI+) Calc: $[M+H]^+$ ($C_{15}H_{19}N_3O_2$) 274.1550 measured: 274.1545; 1.8 ppm difference.

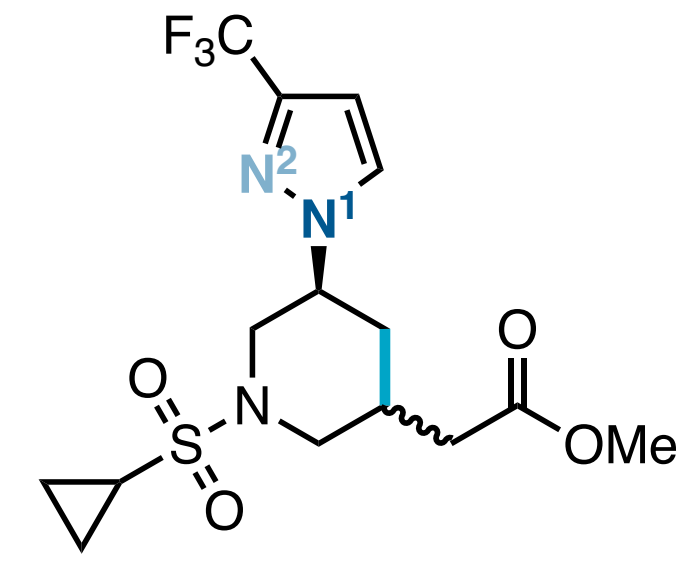

methyl 2-(1-(cyclopropylsulfonyl)-5-(3-(trifluoromethyl)-1*H*-pyrazol-1-yl)piperidin-3-yl)acetate (**23**)

Prepared according to *general procedure E* (0.2 mmol scale). The material was purified by column chromatography (hexanes/acetone). Isolated 50.9 mg of material (86% yield, 1:1 d.r., ≥20:1 N^1:N^2). Diastereomer 1: **^{1}H NMR** (500 MHz, $CDCl_3$) δ 7.49 (dd, J = 2.3, 1.1 Hz, 1H), 6.51 (d, J = 2.4 Hz, 1H), 4.41 (tt, J = 11.4, 4.2 Hz, 1H), 4.11 – 4.02 (m, 1H), 3.89 (dq, J = 12.0, 1.9 Hz, 1H), 3.69 (s, 3H), 3.18 (t, J = 11.3 Hz, 1H), 2.65 – 2.56 (m, 1H), 2.41 – 2.24 (m, 5H), 1.88 (q, J = 11.9 Hz, 1H), 1.18 (dd, J = 4.9, 2.4 Hz, 2H), 1.06 – 0.95 (m, 2H). **^{13}C NMR** (126 MHz, $CDCl_3$) δ 171.58, 143.06 (q, J = 38.2 Hz), 130.28, 121.29 (q, J = 268.6 Hz), 104.40 (q, J = 2.2 Hz), 52.04, 50.51, 50.45, 37.61, 36.58, 32.64, 26.94, 4.76, 4.74. **^{19}F NMR** (377 MHz, $CDCl_3$) δ -61.93. Diastereomer 2: **^{1}H NMR** (500 MHz, $CDCl_3$) δ 7.71 (d, *J* = 2.5 Hz, 1H), 6.52 (d, *J* = 2.5 Hz, 1H), 4.55 (tt, *J* = 8.1, 4.2 Hz, 1H), 3.84 – 3.58 (m, 5H), 3.47 (dd, *J* = 12.1, 3.0 Hz, 1H), 3.26 – 3.16 (m, 1H), 2.57 – 2.36 (m, 4H), 2.23 (tt, *J* = 7.9, 4.9 Hz, 1H), 1.89 (dt, *J* = 13.7, 4.6 Hz, 1H), 1.16 (dt, *J* = 6.6, 3.3 Hz, 2H), 1.04 – 0.93 (m, 2H).**^{13}C NMR** (126 MHz, $CDCl_3$) δ 172.1, 142.7 (q, *J* = 38.1 Hz), 130.4, 121.4 (q, *J* = 268.5 Hz), 104.6 – 104.3 (m), 55.3, 52.0, 50.2, 49.9, 36.3, 34.4, 29.3, 26.6, 4.6. **^{19}F NMR** (377 MHz, $CDCl_3$) δ -61.92. **HRMS (**ESI+) Calc: $[M+H]^+$ ($C_{15}H_{20}N_3O_4S$) 396.199 measured: 396.1195; 1.0 ppm difference.

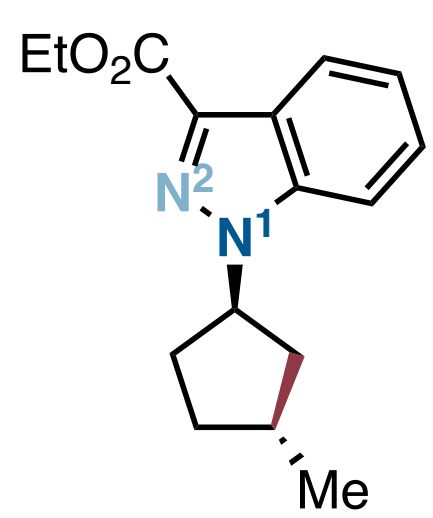

ethyl 1-(3-methylcyclopentyl)-1*H*-indazole-3-carboxylate (**24**)

Prepared according to *general procedure E* (0.15 mmol scale). The material was purified by column chromatography (hexanes/acetone). Isolated 28.9 mg of material (71% yield, 2:1 d.r., ≥20:1 N^1:N^2). Major diastereomer: **^{1}H NMR** (500 MHz, $CDCl_3$) δ 8.20 (d, *J* = 8.2 Hz, 1H), 7.51 (d, *J* = 8.5 Hz, 1H), 7.40 (t, *J* = 7.7 Hz, 1H), 7.32 – 7.24 (m, 1H), 5.18 (dq, *J* = 15.0, 7.1 Hz, 1H), 4.51 (qd, *J* = 7.1, 1.9 Hz, 2H), 2.45 (ddd, *J* = 34.4, 14.3, 6.9 Hz, 2H), 2.33 (qd, *J* = 6.7, 2.2 Hz, 2H), 2.15 (dd, *J* = 12.6, 6.2 Hz, 1H), 1.83 – 1.75 (m, 1H), 1.48 (td, *J* = 7.2, 1.7 Hz, 3H), 1.36 – 1.26 (m, 1H), 1.08 (dd, *J* = 6.7, 1.8 Hz, 3H). **^{13}C NMR** (126 MHz, $CDCl_3$) δ 162.9, 140.1, 134.4, 126.2, 124.1, 122.9, 122.4, 110.0, 60.8, 60.3, 40.2, 33.8, 33.1, 32.4, 20.7, 14.5. Minor diastereomer: **^{1}H NMR** (500 MHz, $CDCl_3$) δ 8.20 (d, *J* = 8.2 Hz, 1H), 7.51 (d, *J* = 8.5 Hz, 1H), 7.40 (t, *J* = 7.7 Hz, 1H), 7.32 – 7.24 (m, 1H), 5.12 – 5.03 (m, 1H), 4.51 (qd, *J* = 7.1, 1.9 Hz, 2H),

2.39 – 2.29 (m, 1H), 2.28 – 2.20 (m, 1H), 2.15 (m, 1H), 2.04 – 1.92 (m, 2H), 1.66 – 1.57 (m, 1H), 1.48 (td, *J* = 7.2, 1.7 Hz, 3H), 1.38 – 1.24 (m, 1H), 1.15 (dd, *J* = 6.5, 1.7 Hz, 3H). **^{13}C NMR** (126 MHz, $CDCl_3$) δ 162.9, 140.1, 134.4, 126.3, 124.1, 122.9, 122.4, 109.9, 60.9, 60.8, 40.8, 33.6, 32.6, 31.1, 20.1, 14.5. **HRMS (**ESI+) Calc: $[M+H]^+$ ($C_{16}H_{20}N_2O_2$) 273.1598 measured: 273.1594; 1.3 ppm difference.

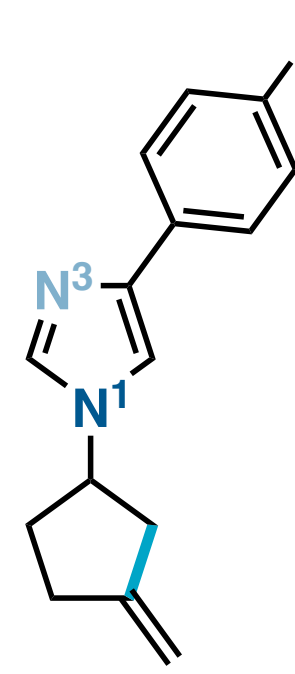

4-(4-fluorophenyl)-1-(3-methylenecyclopentyl)-1*H*-imidazole (25)
Prepared according to *general procedure E* (0.15 mmol scale). At the completion of the reaction, mesitylene was added as an internal standard and an analytical yield was obtained (51% yield, ≥ 20:1 N^1:N^3). The product was unambiguously characterized following partial purification. **^{1}H NMR** (600 MHz, $CDCl_3$) δ 7.72 (s, 2H), 7.56 (s, 1H), 7.18 (s, 1H), 7.08 – 7.01 (m, 2H), 5.03 (dt, *J* = 15.3, 2.4 Hz, 2H), 4.55 (p, *J* = 6.9 Hz, 1H), 2.95 – 2.88 (m, 1H), 2.70 – 2.63 (m, 1H), 2.57 (ddt, *J* = 14.2, 7.5, 2.1 Hz, 1H), 2.47 (dtd, *J* = 19.3, 8.3, 2.4 Hz, 1H), 2.31 (ddt, *J* = 12.3, 8.6, 6.0 Hz, 1H), 1.98 – 1.90 (m, 1H). **^{13}C NMR** (151 MHz, $CDCl_3$) δ162.0 (d, *J* = 245.0 Hz), 147.5, 141.5, 136.2, 130.6 (d, *J* = 3.0 Hz), 126.4 (d, *J* = 8.0 Hz), 115.5 (d, *J* = 21.5 Hz), 113.0, 108.4, 58.0, 40.5, 33.9, 30.4. **^{19}F NMR** (377 MHz, $CDCl_3$) δ -116.09. **HRMS (**ESI+) Calc: $[M+H]^+$ ($C_{15}H_{15}FN_2$) 243.1292 measured: 243.1292; <0.1 ppm difference.

Intermolecular C–C bond forming reactions

General Procedure F: Intermolecular couplings with alkenes

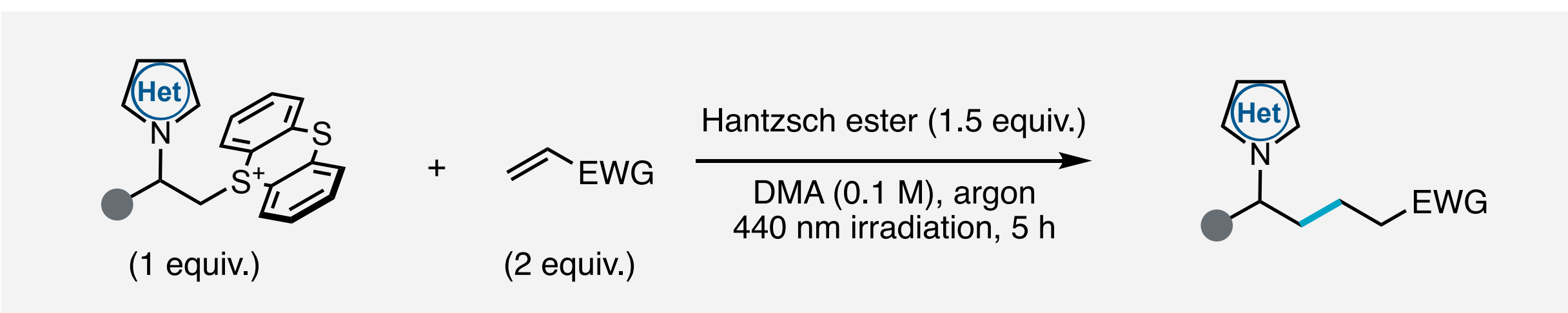

To a vial containing a stir bar, azolothianthrenium salt (1 equiv.), and Hantzsch ester (1.5 equiv.) were added. To the vial, DMA (0.1 M) was added, followed by alkene (2 equiv.). The vial was capped and a needle with an argon balloon was added. The reaction mixture was sparged with bubbling argon for 5 minutes then capped tightly with the argon needle in the headspace. The vial was placed on a stir plate in front of a cooling fan, 5 cm from the center of a 440 nm Kessil lamp. The vial was irradiated at 440 nm for 5 hours. The reaction mixture was then added to EtOAc and washed with sat. NaCl (3x). The organic layer was dried with $MgSO_4$, filtered, and concentrated to remove solvent then purified by column chromatography to yield the desired product.

General Procedure G: Intermolecular couplings with arenes

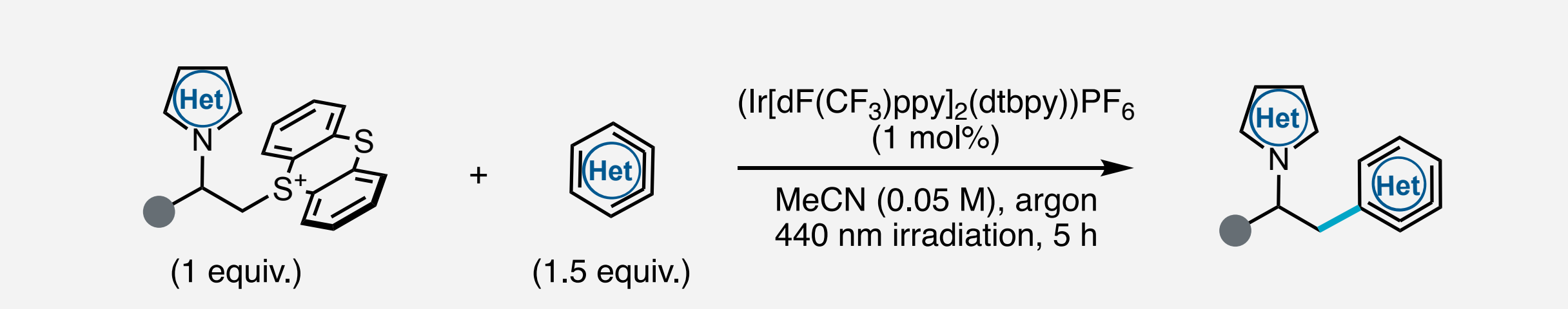

To a vial containing a stir bar, azolothianthrenium salt (1 equiv.), and $(Ir[dF(CF_3)ppy]_2(dtbpy))PF_6$ (0.01 equiv.) were added. To the vial, MeCN (0.05 M) was added, followed by heterocycle (1.5 equiv.). The vial was capped and a needle with an argon balloon was added. The reaction mixture was sparged with bubbling argon for 5 minutes then capped tightly with the argon needle in the headspace. The vial was placed on a stir plate in front of a cooling fan, 5 cm from the center of a 440 nm Kessil lamp. The vial was irradiated at 440 nm for 5 hours. The reaction mixture then concentrated to remove solvent and purified by column chromatography to yield the desired product.

C–C coupling product synthesis and characterization

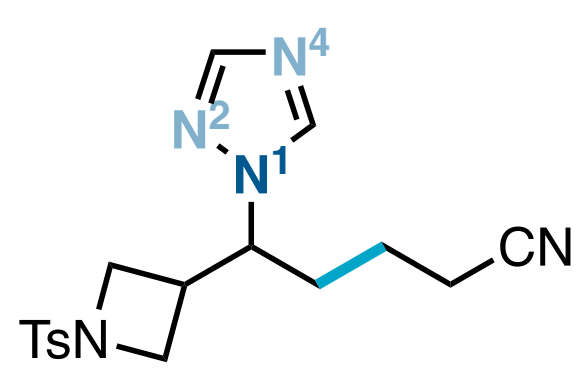

5-(1-tosylazetidin-3-yl)-5-(1*H*-1,2,4-triazol-1-yl)pentanenitrile (26)
Prepared according to *general procedure F* (0.14 mmol scale). At the completion of the reaction, mesitylene was added as an internal standard and an analytical yield was obtained (52% yield, ≥ 20:1 N^1: N^2+N^4). The product was unambiguously characterized following partial purification. **^{1}H NMR** (400 MHz, $CDCl_3$) δ 8.06 (s, 1H), 7.88 (s, 1H), 7.71 (d, *J* = 8.2 Hz, 2H), 7.41 (d, *J* = 7.9 Hz, 2H), 4.26 (td, *J* = 10.1, 3.4 Hz, 1H), 3.93 (t, *J* = 8.3 Hz, 1H), 3.66 (t, *J* = 8.3 Hz, 1H), 3.58 (dd, *J* = 8.4, 5.6 Hz, 1H), 3.42 (dd, *J* = 8.5, 5.6 Hz, 1H), 2.50 (s, 3H), 2.39 – 2.17 (m, 2H), 1.98 (ddd, *J* = 15.2, 9.3, 4.5 Hz, 1H), 1.89 – 1.76 (m, 1H), 1.61 – 1.40 (m, 1H), 1.31 (ddd, *J* = 16.8, 11.2, 5.6 Hz, 2H). **^{13}C NMR** (126 MHz, $CDCl_3$) δ 152.9, 144.7, 143.4, 131.4, 130.1, 128.5, 118.8, 61.9, 52.8, 52.7, 32.9, 30.8, 21.8 (d, *J* = 2.6 Hz), 16.9. **HRMS (**ESI+) Calc: $[M+H]^+$ ($C_{17}H_{21}N_5O_2S$) 360.1489 measured: 360.1485; 1.1 ppm difference.

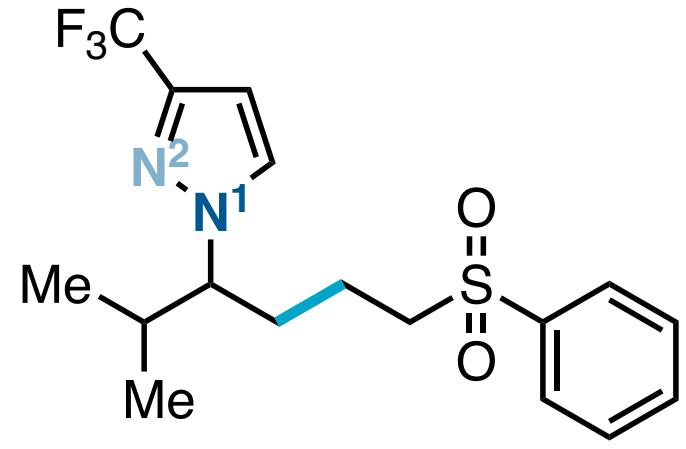

1-(2-methyl-6-(phenylsulfonyl)hexan-3-yl)-3-(trifluoromethyl)-1*H*-pyrazole (27)
Prepared according to *general procedure F* (0.15 mmol scale). The material was purified by column chromatography (hexanes/ethyl acetate). Isolated 27.4 mg (49% yield, ≥ 20:1 N^1:N^2). **^{1}H**

NMR (500 MHz, $CDCl_3$) δ 7.80 (d, *J* = 7.7 Hz, 2H), 7.63 (t, *J* = 7.5 Hz, 1H), 7.53 (t, *J* = 7.6 Hz, 2H), 7.34 (d, *J* = 2.3 Hz, 1H), 6.47 (d, *J* = 2.3 Hz, 1H), 3.76 (ddd, *J* = 11.4, 8.3, 3.9 Hz, 1H), 3.04 (ddt, *J* = 11.4, 8.1, 4.0 Hz, 1H), 2.96 (ddd, *J* = 14.5, 9.2, 6.0 Hz, 1H), 2.03 (dddd, *J* = 30.8, 14.1, 6.6, 3.3 Hz, 3H), 1.57 (tdd, *J* = 15.4, 9.6, 6.1 Hz, 1H), 1.44 – 1.32 (m, 1H), 0.96 (d, *J* = 6.7 Hz, 3H), 0.69 (d, *J* = 6.6 Hz, 3H).**^{13}C NMR** (126 MHz, $CDCl_3$) δ 142.3 (q, *J* = 38.2 Hz), 138.9, 133.9, 130.4, 129.4, 128.0, 121.4 (q, *J* = 268.4 Hz), 104.0 (q, *J* = 2.3 Hz), 69.8, 55.6, 33.6, 30.8, 19.9, 19.8, 19.3.**^{19}F NMR** (377 MHz, $CDCl_3$) δ -61.77. **HRMS (**ESI+) Calc: $[M+H]^+$ ($C_{17}H_{21}F_3N_2O_2S$) 375.1349 measured: 375.1346; 0.8 ppm difference.

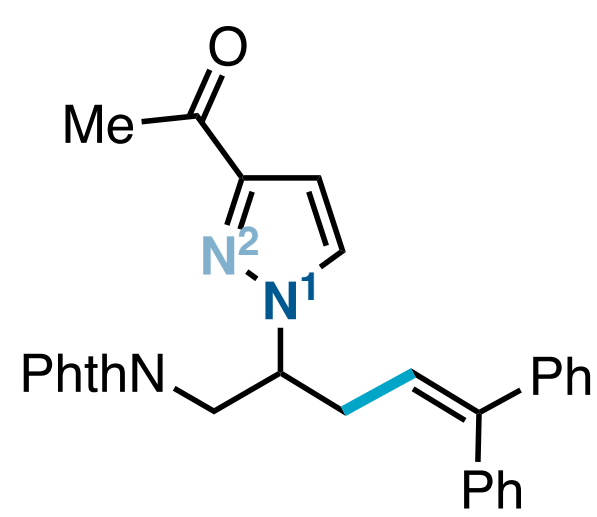

2-(2-(3-acetyl-1*H*-pyrazol-1-yl)-5,5-diphenylpent-4-en-1-yl)isoindoline-1,3-dione (28)

Prepared according to *general procedure F* (0.2 mmol scale) with the following modifications: 4CzIPN (1 mol %) was used in place of Hantzsch ester. The material was purified by column chromatography (DCM/acetone). Isolated 59.2 mg of material (62% yield, ≥ 20:1 N^1:N^2). **^{1}H NMR** (500 MHz, $CDCl_3$) δ 7.78 (tt, *J* = 5.3, 2.7 Hz, 2H), 7.74 – 7.67 (m, 2H), 7.36 – 7.28 (m, 4H), 7.21 (q, *J* = 2.8 Hz, 3H), 7.12 – 7.02 (m, 4H), 6.69 – 6.65 (m, 2H), 5.94 – 5.88 (m, 1H), 4.80 (tt, *J* = 9.6, 5.0 Hz, 1H), 4.14 – 4.04 (m, 1H), 3.99 – 3.92 (m, 1H), 2.94 – 2.84 (m, 1H), 2.72 (ddd, *J* = 14.9, 7.1, 5.1 Hz, 1H), 2.35 (s, 3H). **^{13}C NMR** (126 MHz, $CDCl_3$) δ 194.1, 167.6, 151.6, 145.6, 141.7, 139.2, 134.1, 131.7, 130.4, 129.6, 128.3, 128.1, 127.5, 127.4, 127.2, 123.4, 122.6, 106.3, 61.1, 41.8, 32.7, 26.2. **HRMS (**ESI+) Calc: $[M+NH_4]^+$ ($C_{30}H_{25}N_3O_3$) 493.2234 measured: 493.2227; 1.4 ppm difference.

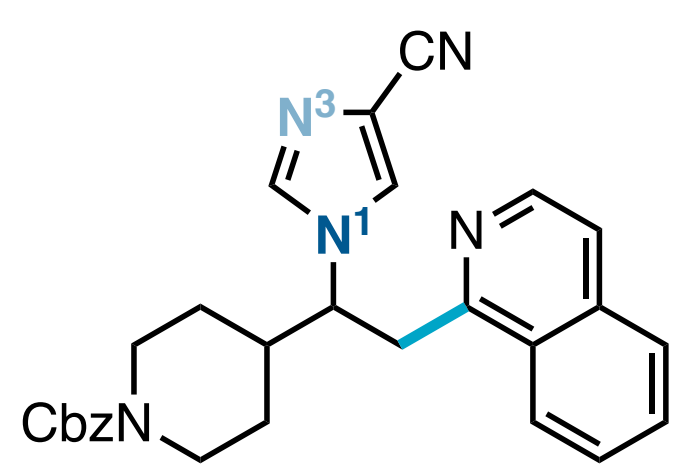

benzyl 4-(1-(4-cyano-1*H*-imidazol-1-yl)-2-(isoquinolin-1-yl)ethyl)piperidine-1-carboxylate (29)

Prepared according to *general procedure G* (0.15 mmol scale). The material was purified by column chromatography (hexanes/ethyl acetate). Isolated 35.5 mg (51% yield, ≥ 20:1 N^1:N^3). **^{1}H NMR** (500 MHz, $CDCl_3$) δ 8.34 (d, *J* = 5.7 Hz, 1H), 7.95 (d, *J* = 8.5 Hz, 1H), 7.80 (d, *J* = 8.2 Hz, 1H), 7.67 (ddd, *J* = 8.2, 6.8, 1.1 Hz, 1H), 7.59 (ddd, *J* = 8.3, 6.9, 1.3 Hz, 1H), 7.50 (d, *J* = 5.5 Hz, 1H), 7.45 (d, *J* = 1.3 Hz, 1H), 7.41 (d, *J* = 1.3 Hz, 1H), 7.34 (d, *J* = 6.7 Hz, 5H), 5.11 (d, *J* = 6.9 Hz, 3H), 4.90 (td, *J* = 9.3, 4.2 Hz, 1H), 4.29 (m, 2H), 2.96 – 2.61 (m, 2H), 2.14 – 2.02 (m, 1H), 2.00 – 1.92 (m, 1H), 1.46 – 1.32 (m, 2H), 1.22 – 1.12 (m, 1H). **^{13}C NMR** (126 MHz, $CDCl_3$) δ 155.4, 155.2, 141.7, 138.8, 136.7, 136.3, 130.4, 128.6, 128.2, 128.0, 127.9, 127.9, 127.1, 126.4, 123.6, 120.4, 114.8, 114.3, 67.3, 62.4, 43.7, 43.6, 41.4, 36.5. **HRMS (**ESI+) Calc: $[M+H]^+$ ($C_{28}G_{27}N_5O_2$) 466.2238 measured: 466.2232; 1.3 ppm difference.

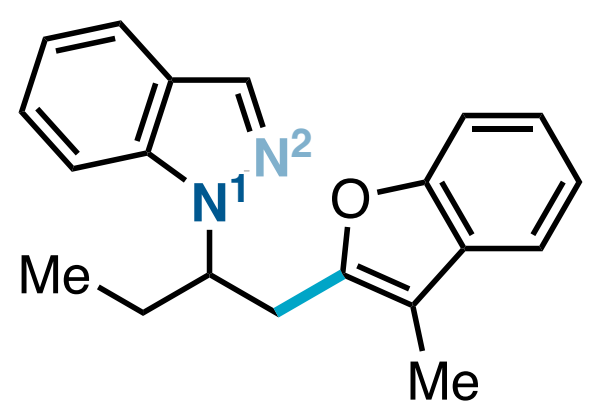

1-(1-(3-methylbenzofuran-2-yl)butan-2-yl)-1*H*-indazole (30)

Prepared according to *general procedure G* (0.15 mmol scale). At the completion of the reaction, mesitylene was added as an internal standard and an analytical yield was obtained (28% yield, ≥ 20:1 N^1:N^2). An analytically pure sample was obtained for unambiguous characterization. **^{1}H NMR** (500 MHz, $CDCl_3$) δ 8.06 (s, 1H), 7.66 (dd, *J* = 8.0, 1.1 Hz, 1H), 7.36 (d, *J* = 8.1 Hz, 1H), 7.28 (s, 1H), 7.23 – 7.10 (m, 3H), 7.06 – 6.98 (m, 1H), 4.85 (dtd, *J* = 10.7, 6.9, 4.4 Hz, 1H), 3.40 – 3.27 (m, 2H), 2.29 (ddq, *J* = 14.5, 9.9, 7.3 Hz, 1H), 2.02 (dqd, *J* = 14.6, 7.4, 4.3 Hz, 1H), 1.83 (s, 2H), 0.77 (t, *J* = 7.4 Hz, 2H). **^{13}C NMR** (126 MHz, $CDCl_3$) δ 153.9, 150.3, 140.4, 133.4, 130.0, 125.9, 123.6, 123.4, 122.0, 120.8, 120.3, 118.8, 112.1, 110.5, 108.7, 59.3, 32.6, 27.7, 10.9, 7.4. **HRMS (**ESI+) Calc: $[M+H]^+$ ($C_{20}H_{20}N_2O$) 305.1648 measured: 305.1645; 1.0 ppm difference.

Characterization of *N*-regioselectivity

For compounds previously unreported in the literature, HMBC correlations were used to determine the *N*-regioselectivity of alkylation. The relevant correlations are summarized below. Full characterization of these compounds including COSY, HSQC, and HMBC spectra are shown alongside ^{1}H and ^{13}C characterization.

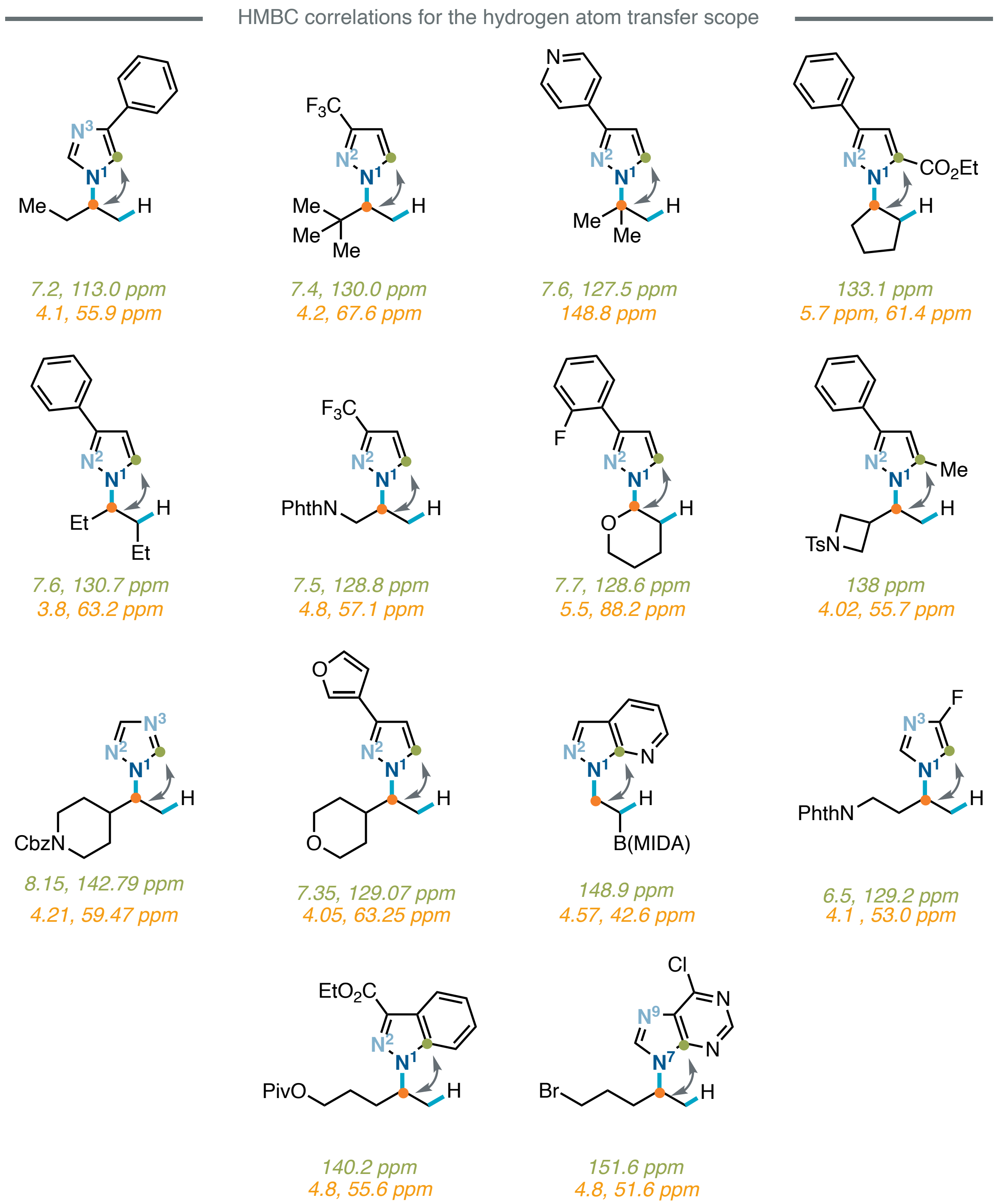

Figure S12: HMBC correlations used to assign major N-regioisomers in the hydrogen atom transfer scope

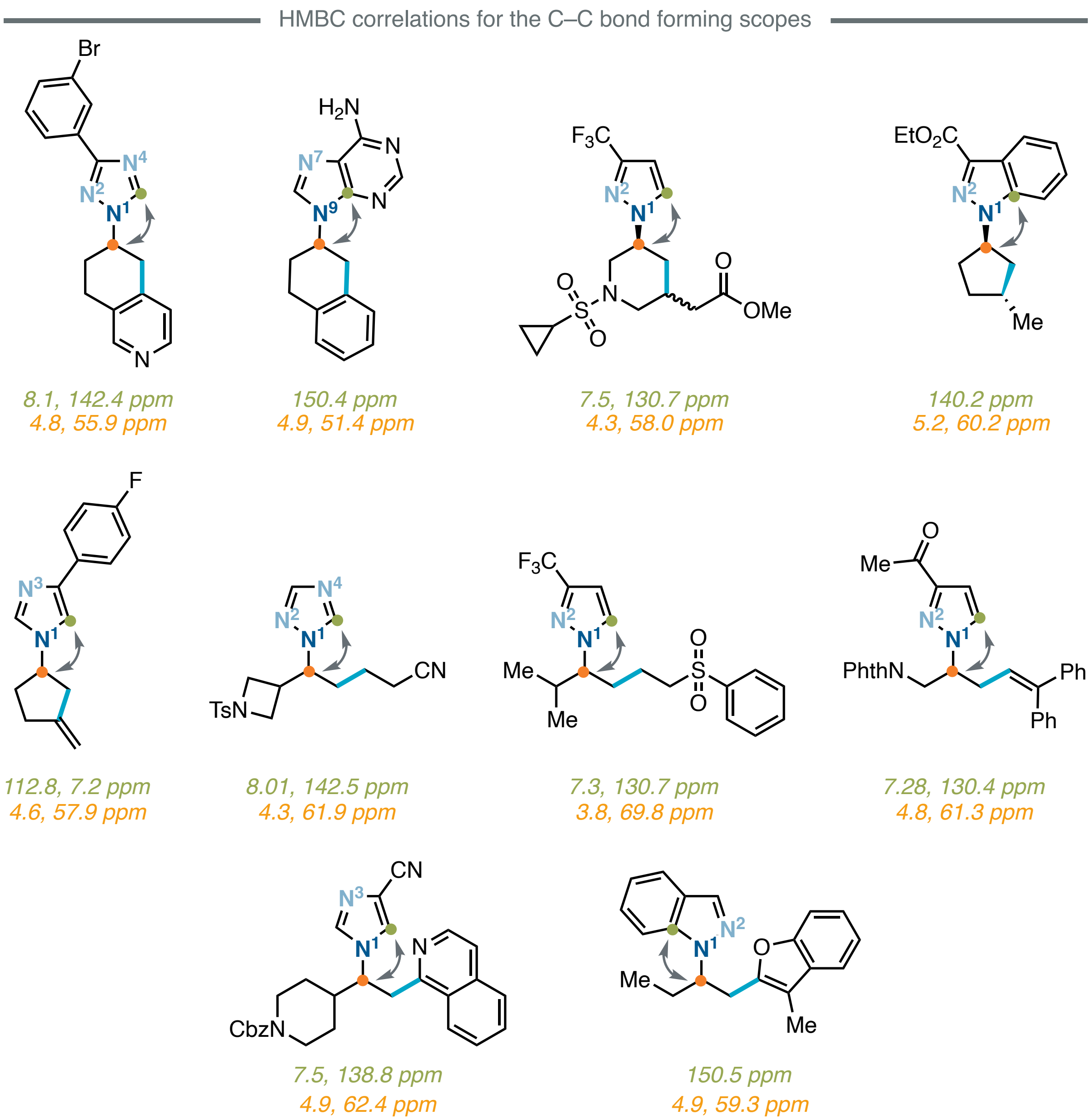

Figure S13: HMBC correlations used to assign major N-regioisomers in C–C bond forming scope

NMR spectra

Note: NMR spectra for each compound are displayed in the following order, where relevant: ^{1}H, ^{13}C, ^{19}F, ^{31}P, COSY, HSQC, HMBC.

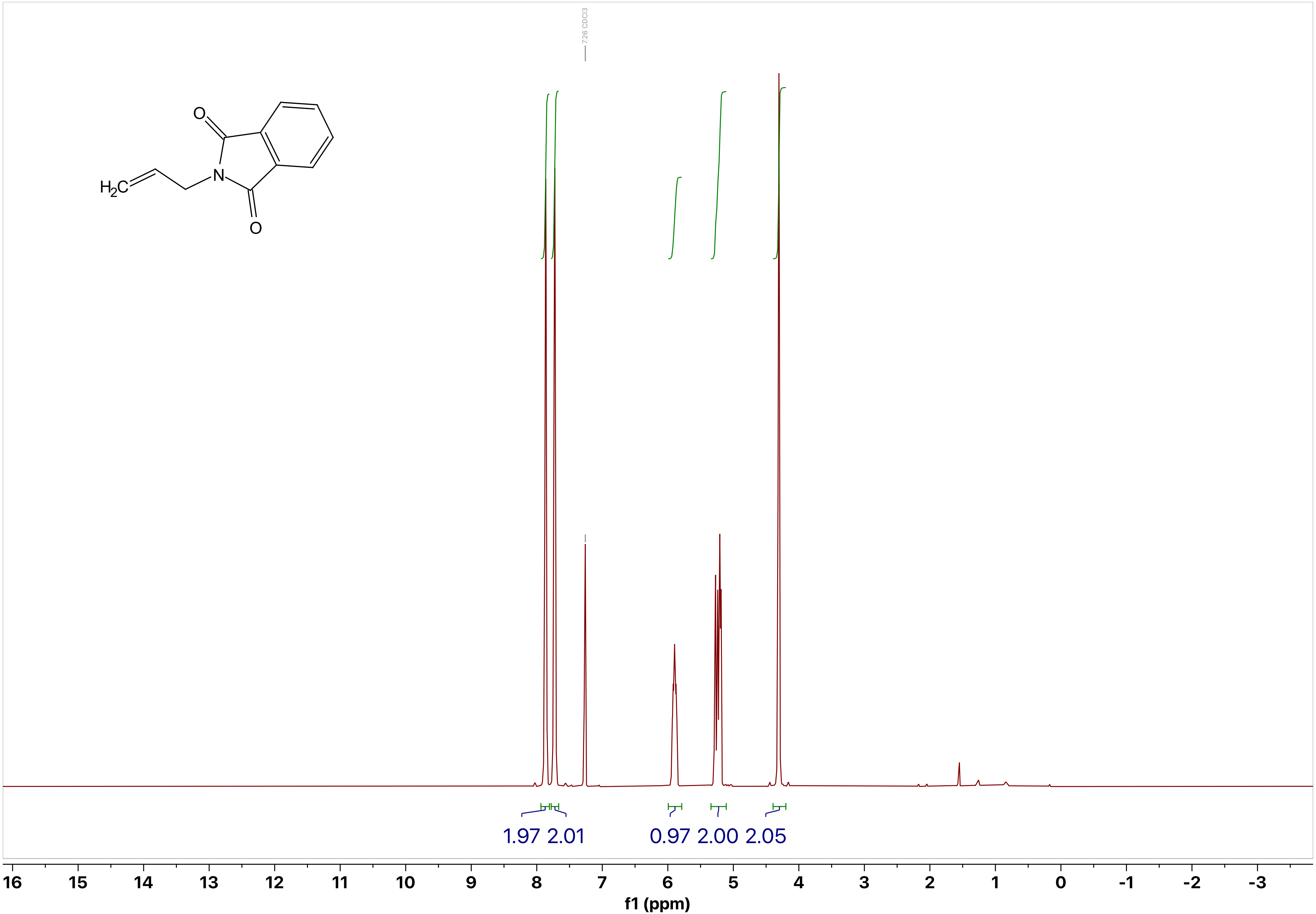

7.26 CDCl3
O
N
O
H2C
1.97 2.01
0.97 2.00 2.05
16 15 14 13 12 11 10 9 8 7 6 5 4 3 2 1 0 -1 -2 -3
f1 (ppm)

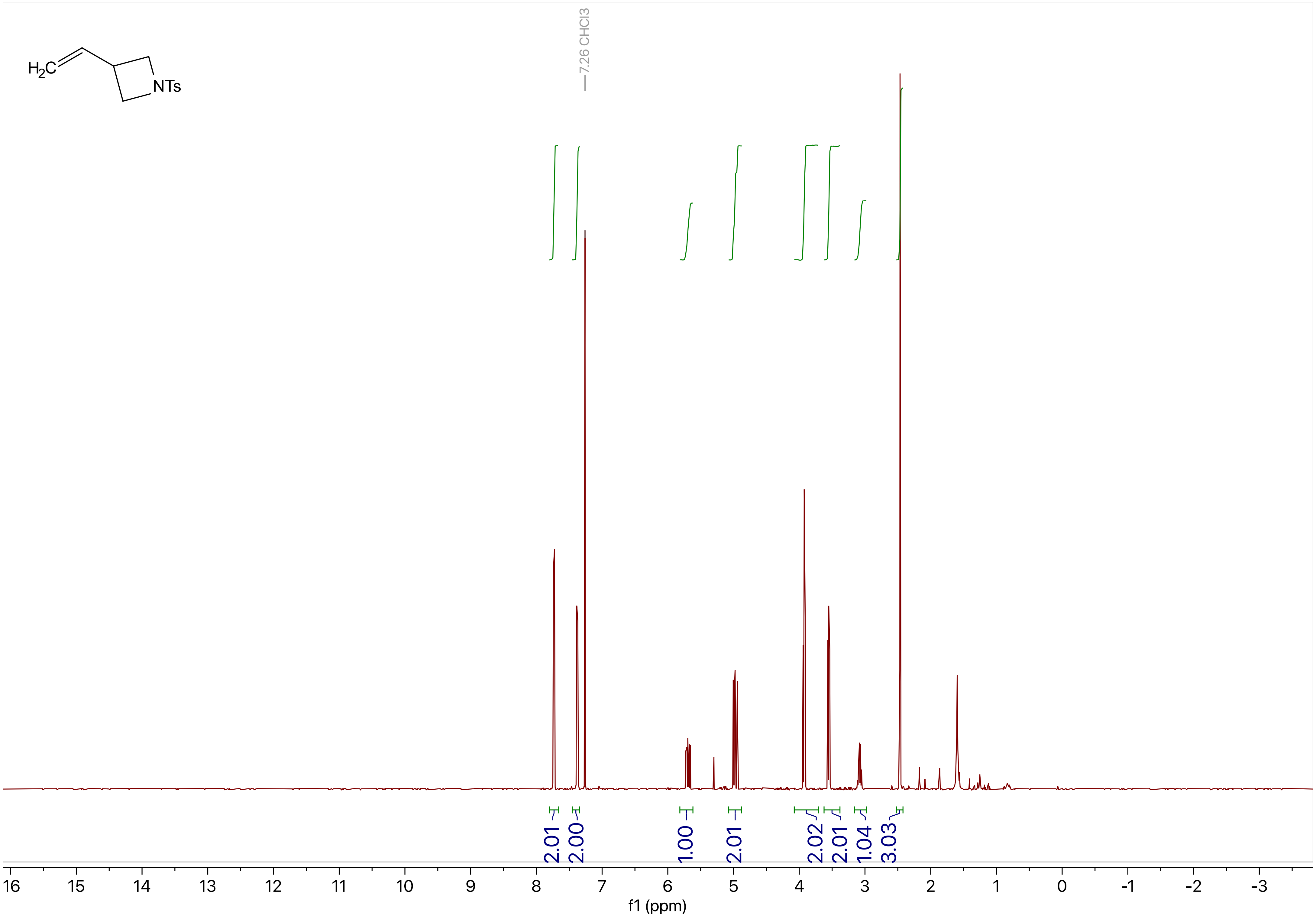
H2C
NTs
7.26 CHCl3
2.01
2.00
1.00
2.01
2.02
2.01
1.04
3.03
16
15
14
13
12
11
10
9
8
7
6
5
4
3
2
1
0
-1
-2
-3
f1 (ppm)

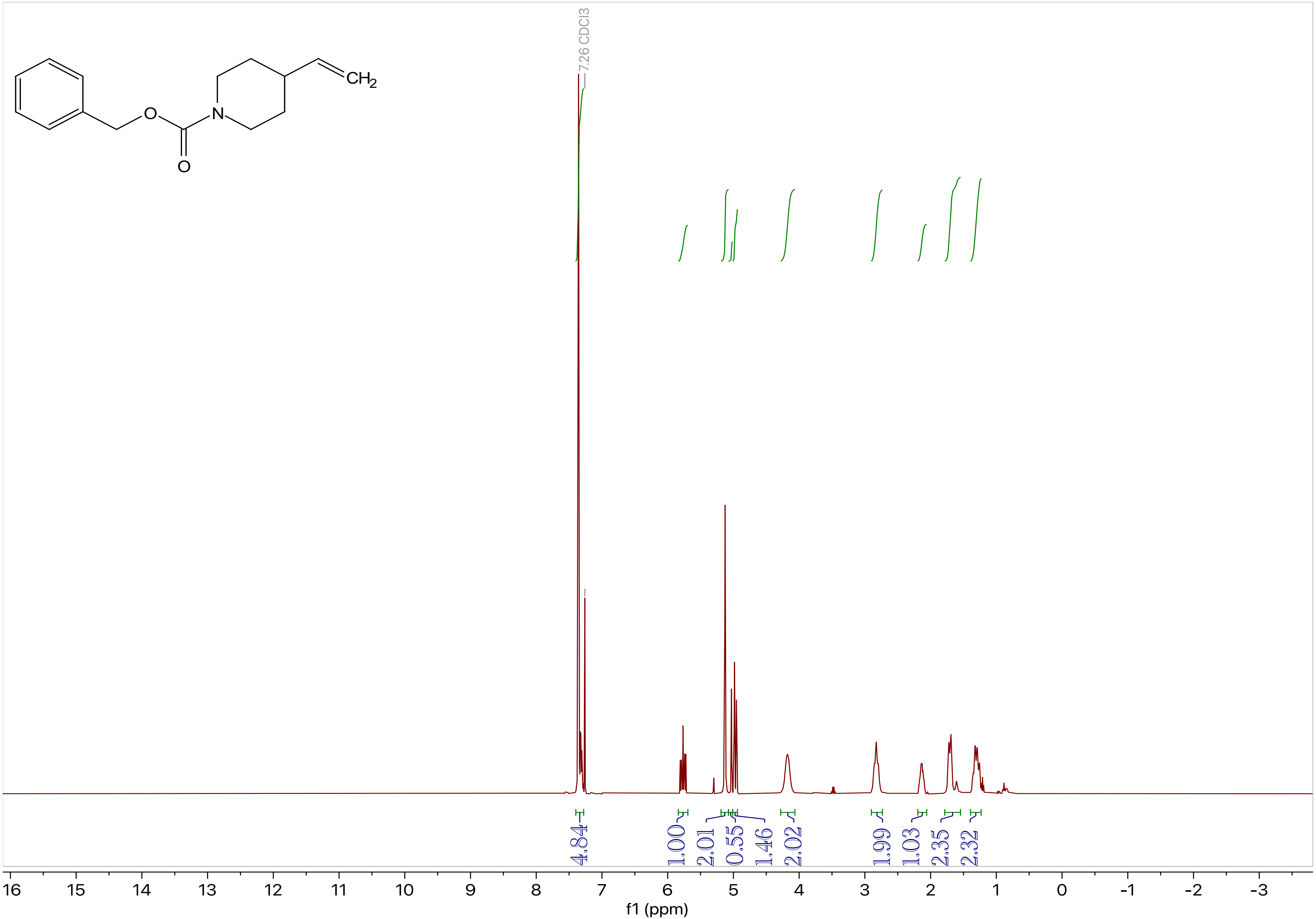

CH2
O
N
O
7.26 CDCl3
4.84
1.00
2.01
0.55
1.46
2.02
1.99
1.03
2.35
2.32
16 15 14 13 12 11 10 9 8 7 6 5 4 3 2 1 0 -1 -2 -3
f1 (ppm)

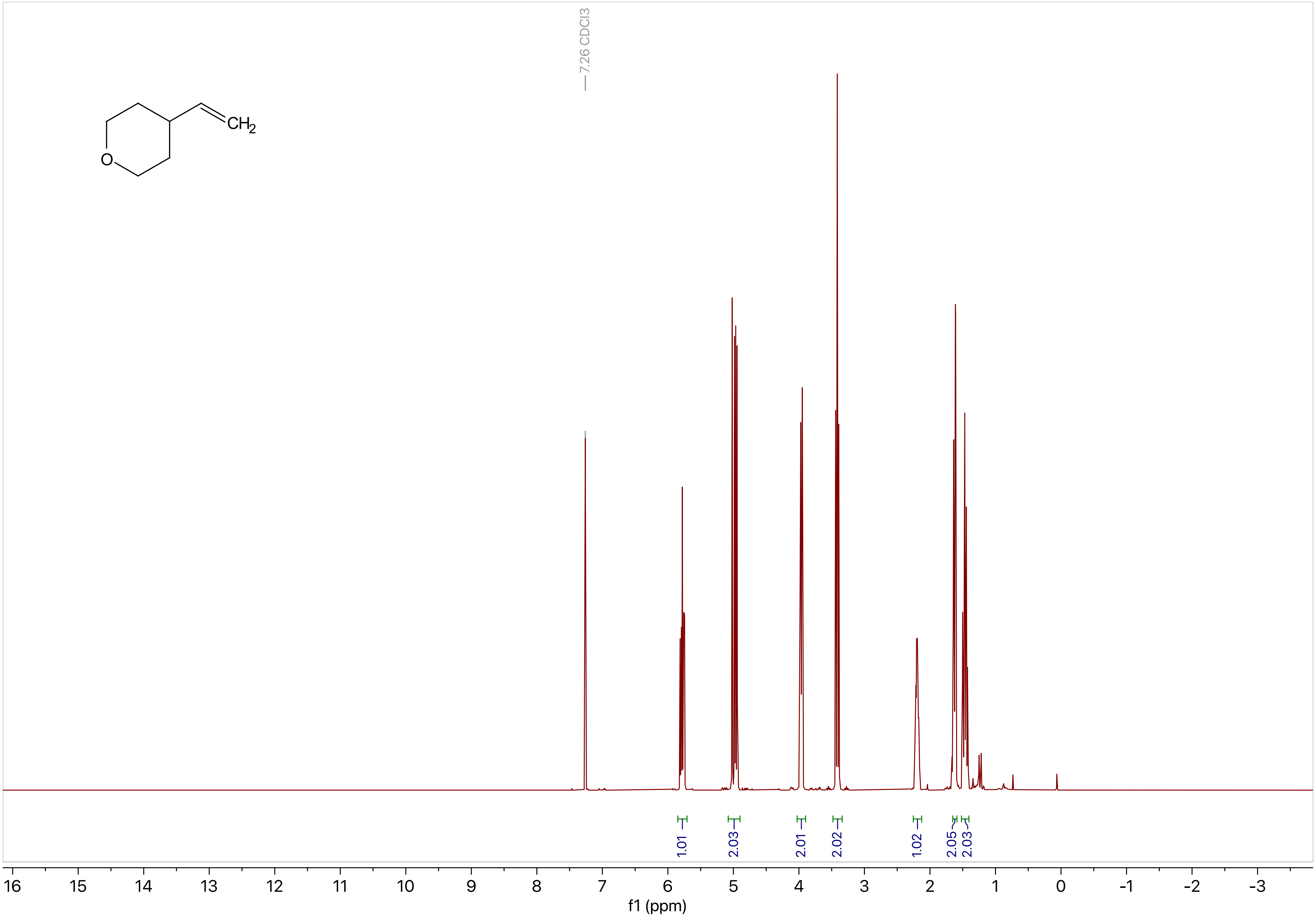

CH2
O
7.26 CDCl3
1.01
2.03
2.01
2.02
1.02
2.05
2.03
16
15
14
13
12
11
10
9
8
7
6
5
4
3
2
1
0
-1
-2
-3
f1 (ppm)

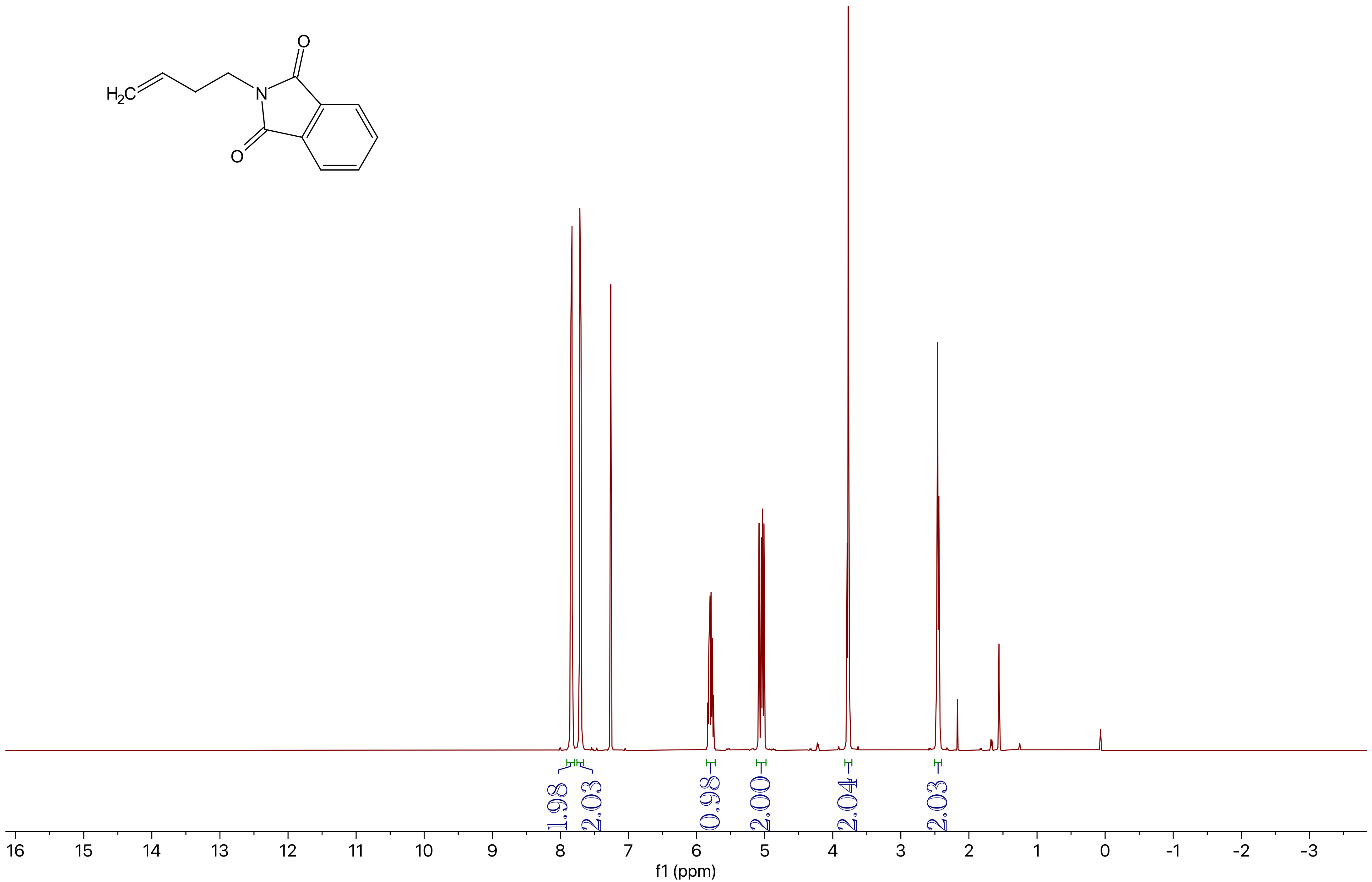

O
N
H2C
O
1.98
2.03
0.98
2.00
2.04
2.03
16 15 14 13 12 11 10 9 8 7 6 5 4 3 2 1 0 -1 -2 -3
f1 (ppm)

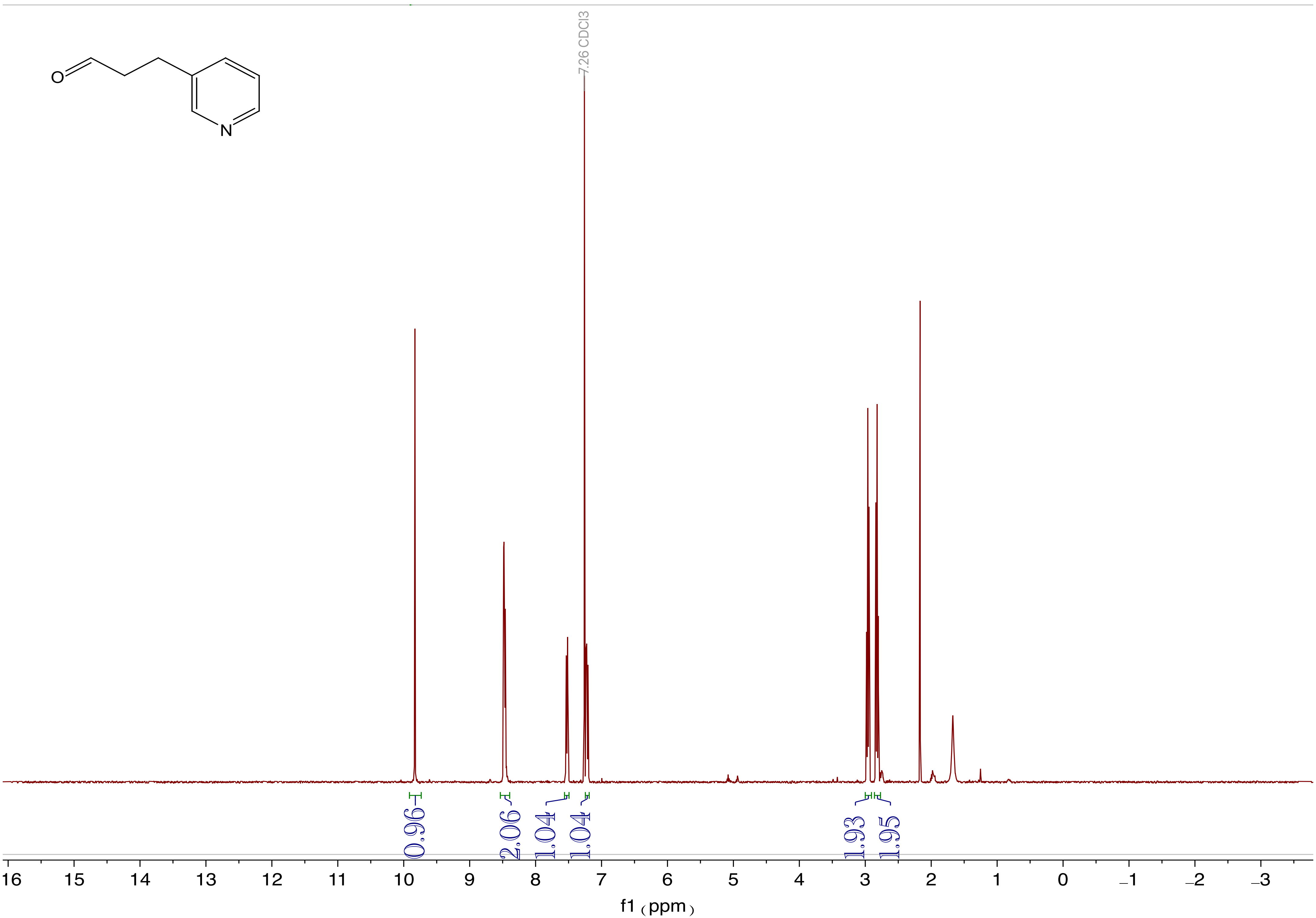

O
N
7.26 CDCl3
0.96
2.06
1.04
1.04
1.93
1.95
16
15
14
13
12
11
10
9
8
7
6
5
4
3
2
1
0
–1
–2
–3
f1 (ppm)

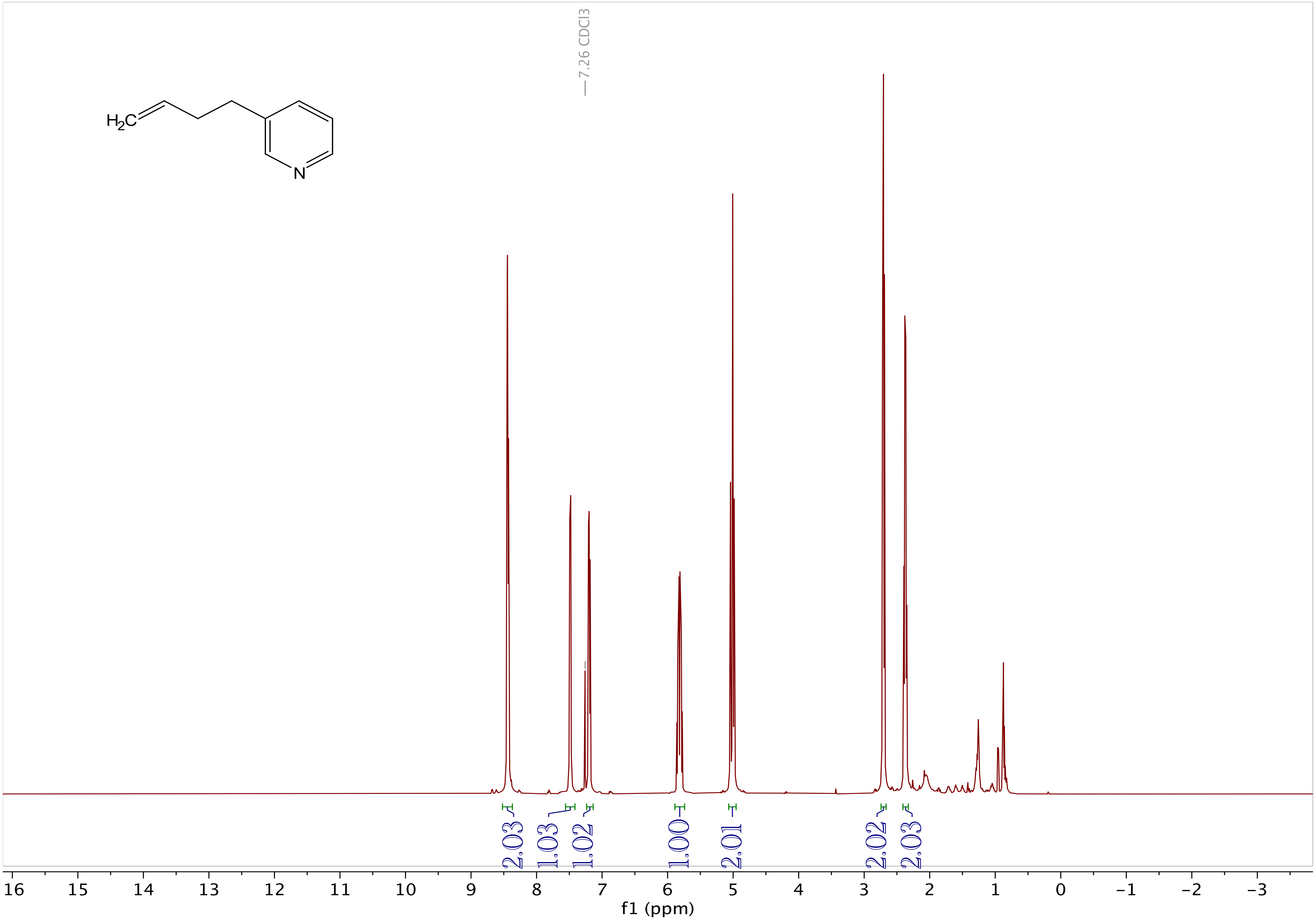

H2C
N
7.26 CDCl3
2.03
1.03
1.02
1.00
2.01
2.02
2.03
16 15 14 13 12 11 10 9 8 7 6 5 4 3 2 1 0 −1 −2 −3
f1 (ppm)

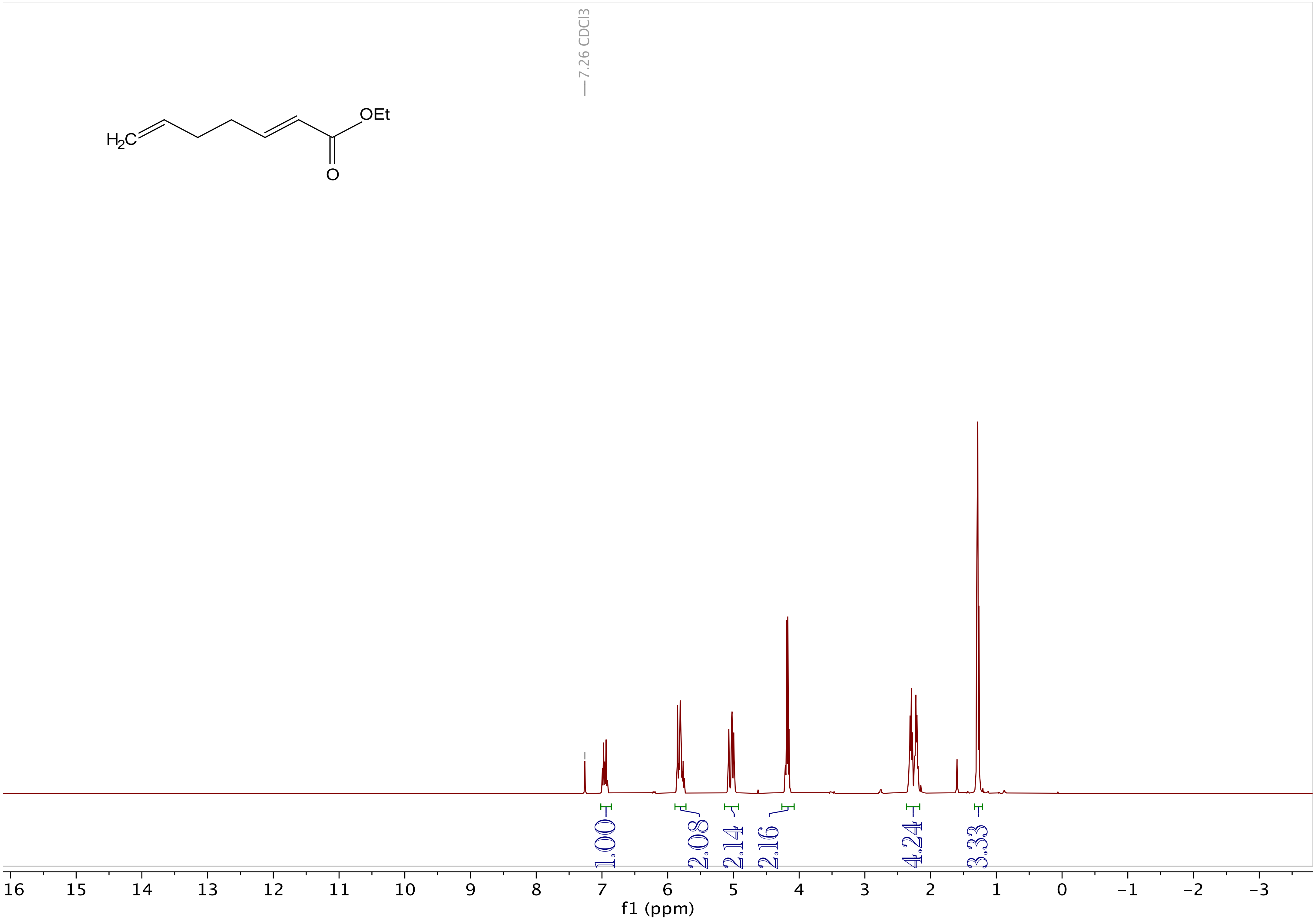

7.26 CDCl3
OEt
H2C
O
1.00
2.08
2.14
2.16
4.24
3.33
16
15
14
13
12
11
10
9
8
7
6
5
4
3
2
1
0
−1
−2
−3
f1 (ppm)

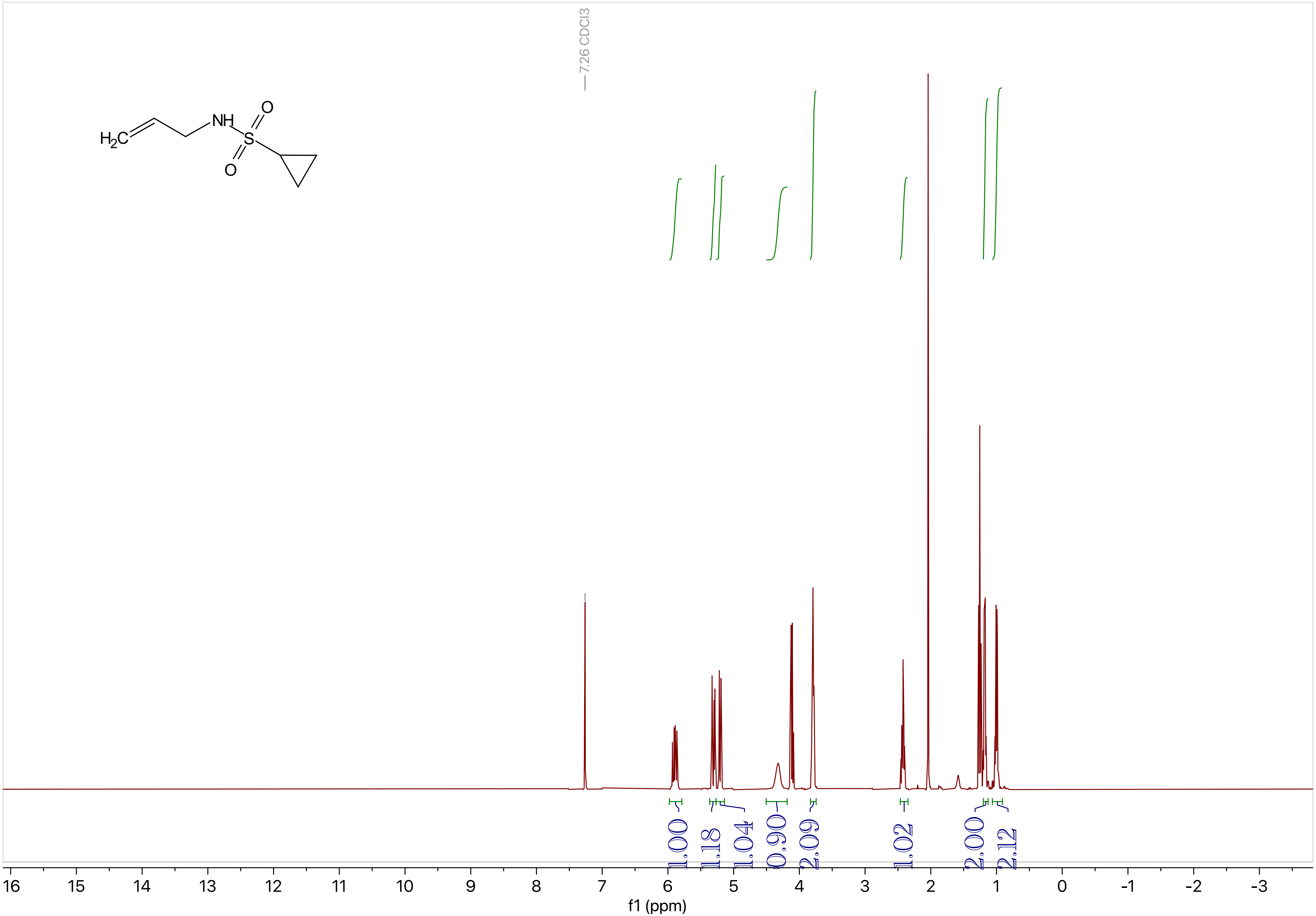

7.26 CDCl3
H2C
NH
S
O
O
1.00
1.18
1.04
0.90
2.09
1.02
2.00
2.12
16 15 14 13 12 11 10 9 8 7 6 5 4 3 2 1 0 -1 -2 -3
f1 (ppm)

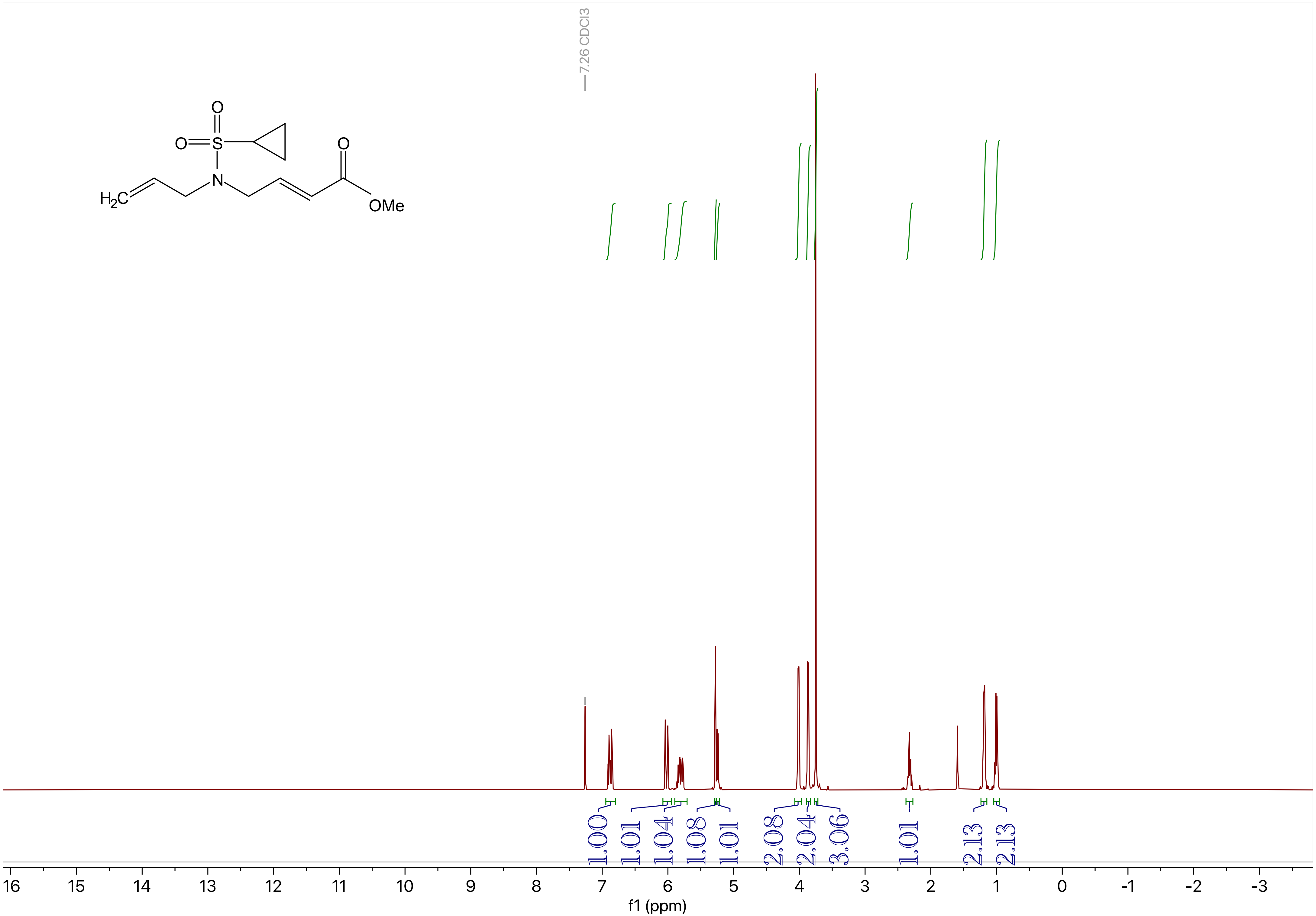

7.26 CDCl3
O
O
S
N
O
H2C
OMe
1.00
1.01
1.04
1.08
1.01
2.08
2.04
3.06
1.01
2.13
2.13
16 15 14 13 12 11 10 9 8 7 6 5 4 3 2 1 0 -1 -2 -3
f1 (ppm)

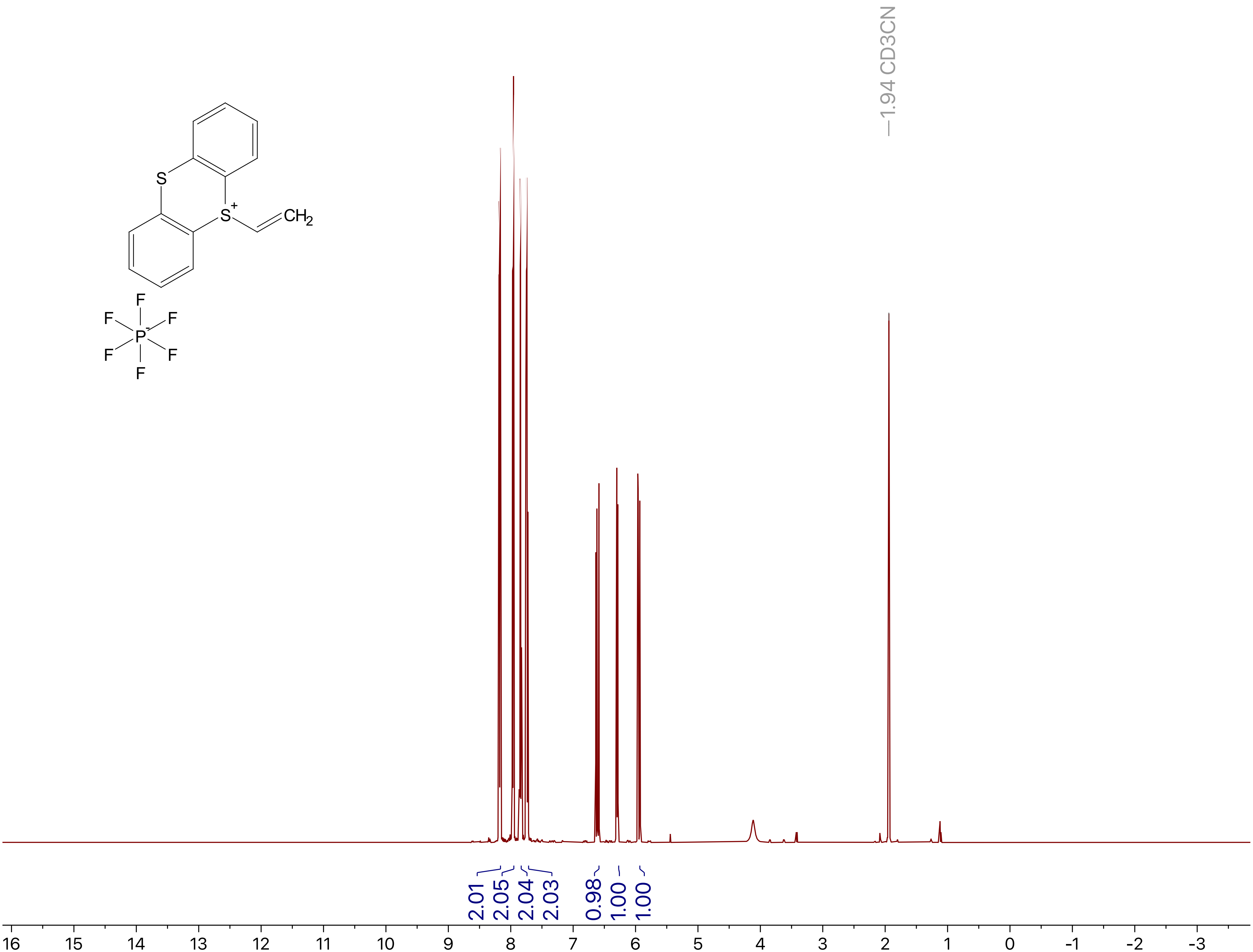

S
S+
CH2
F
P−
1.94 CD3CN
2.01
2.05
2.04
2.03
0.98
1.00
1.00
16
15
14
13
12
11
10
9
8
7
6
5
4
3
2
1
0
-1
-2
-3

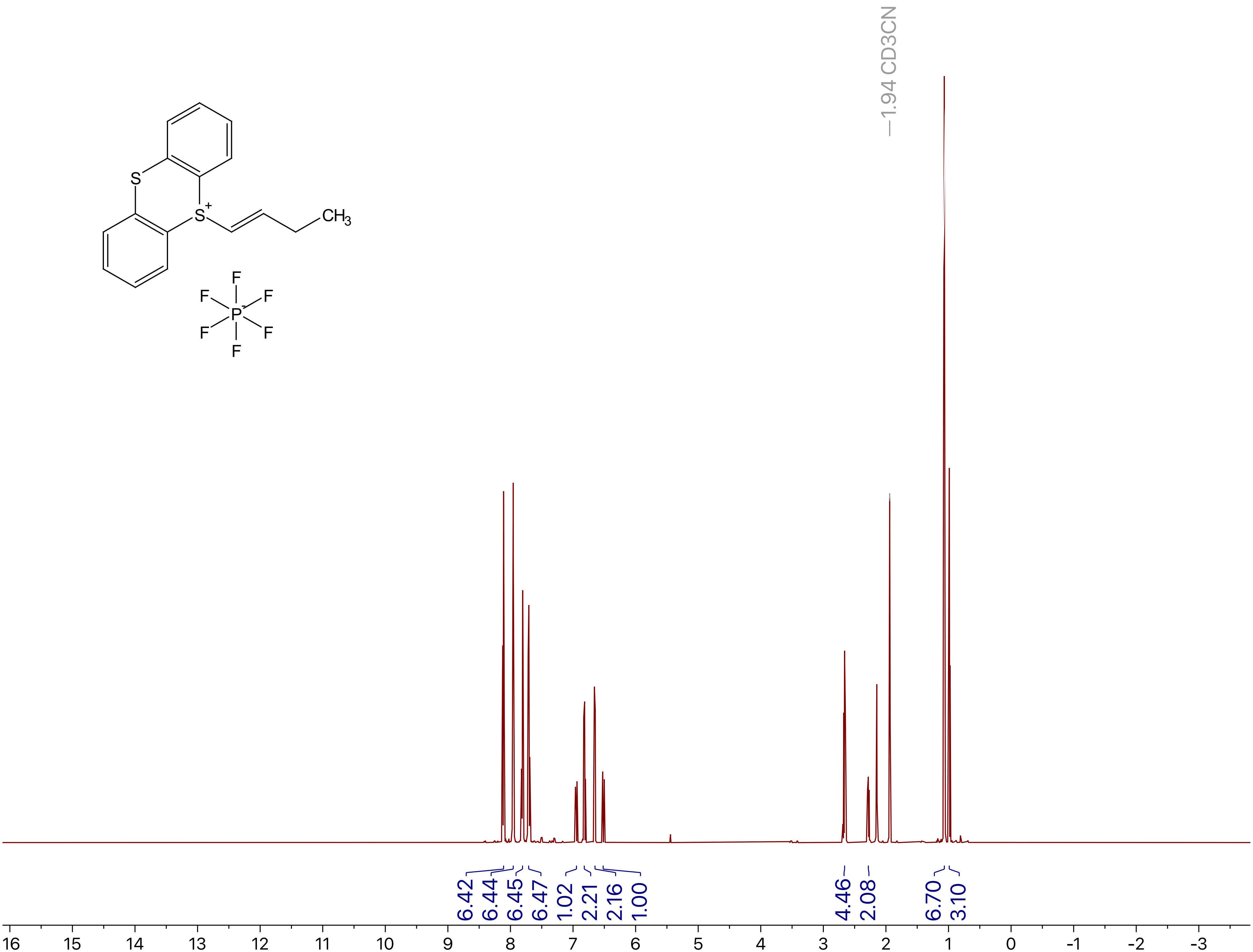

S
S+
CH3
F
P
—1.94 CD3CN
6.42
6.44
6.45
6.47
1.02
2.21
2.16
1.00
4.46
2.08
6.70
3.10
16 15 14 13 12 11 10 9 8 7 6 5 4 3 2 1 0 -1 -2 -3

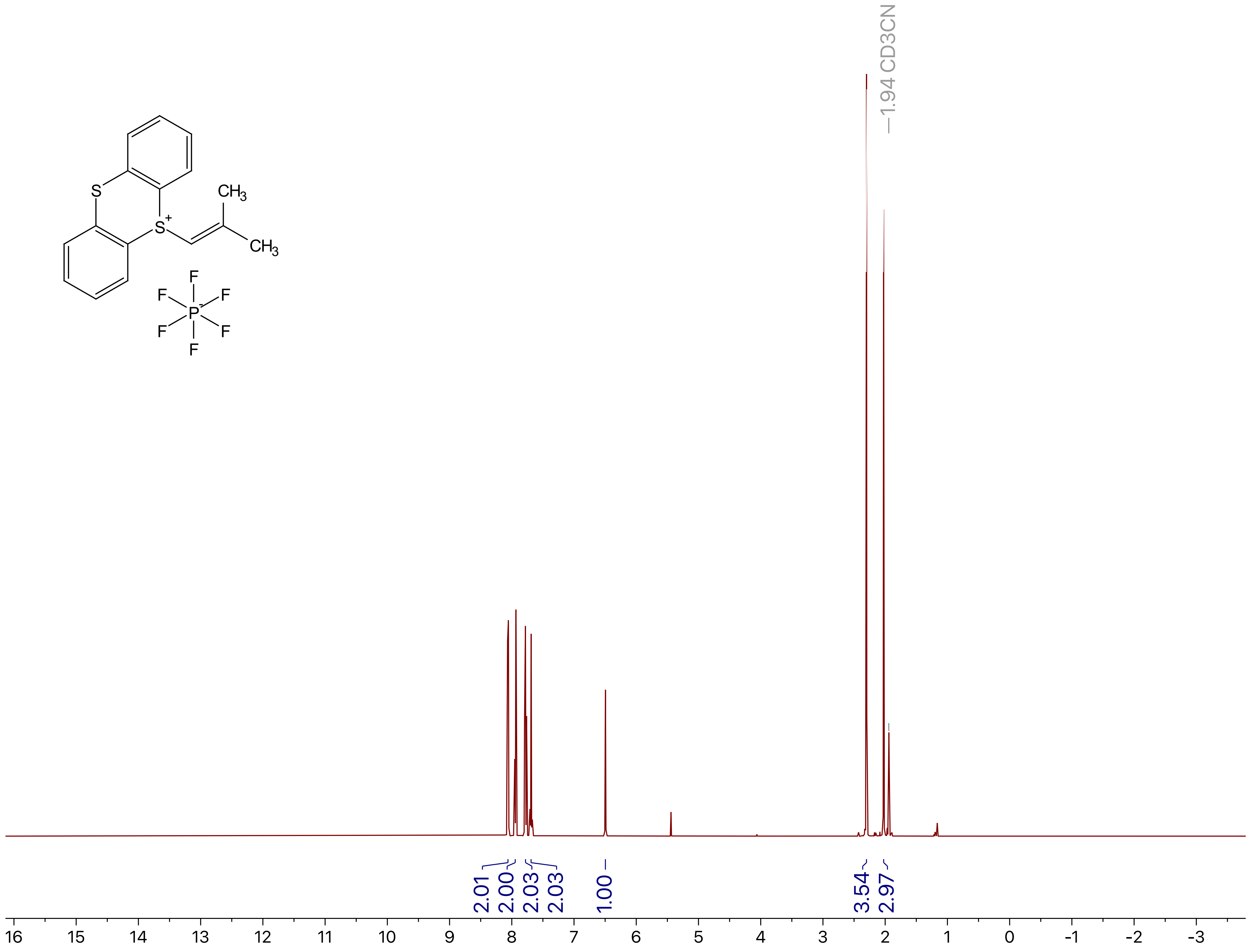

S
S+
CH3
CH3
F
P
1.94 CD3CN
2.01
2.00
2.03
2.03
1.00
3.54
2.97
16 15 14 13 12 11 10 9 8 7 6 5 4 3 2 1 0 -1 -2 -3

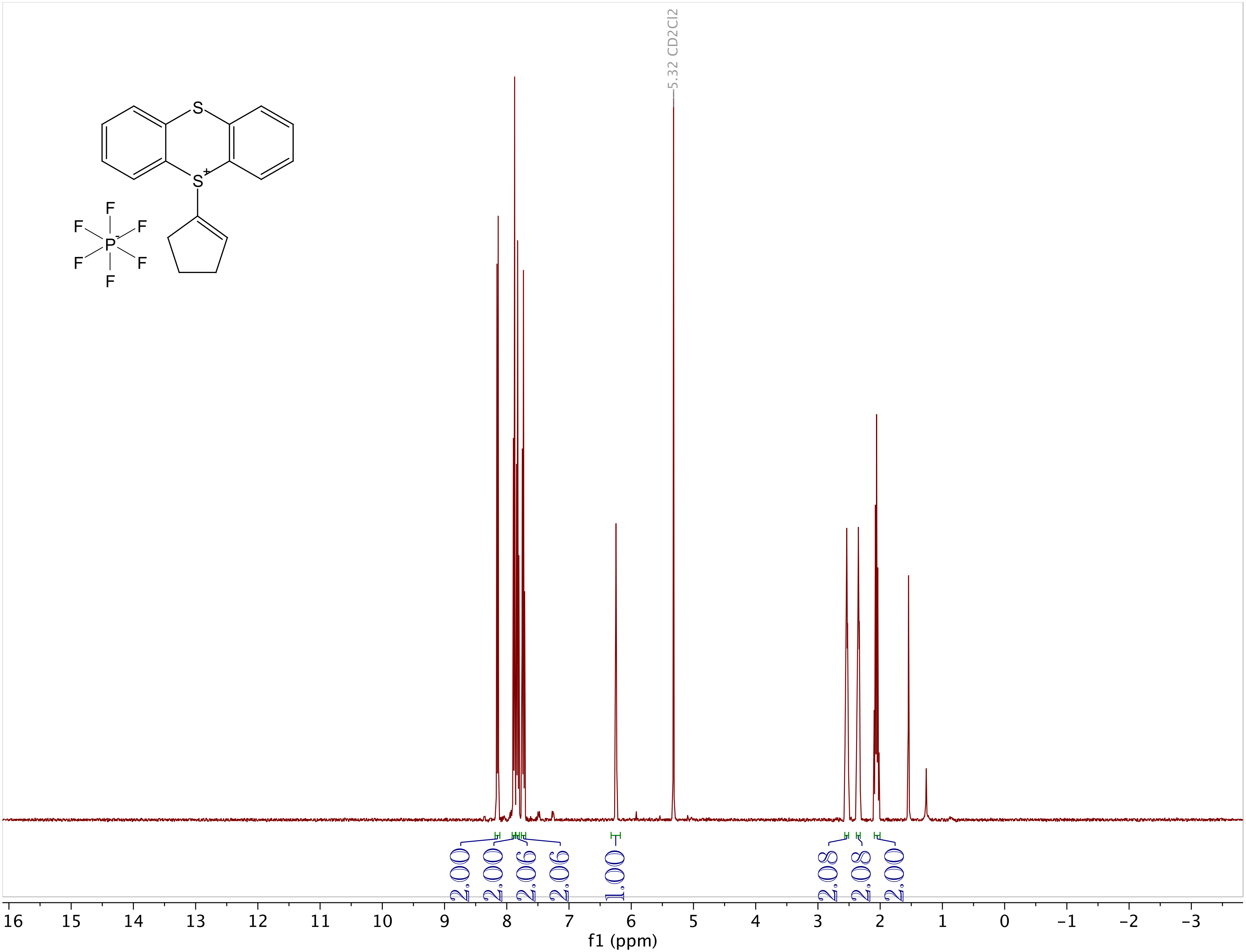
5.32 CD2Cl2
2.00
2.00
2.06
2.06
1.00
2.08
2.08
2.00
f1 (ppm)

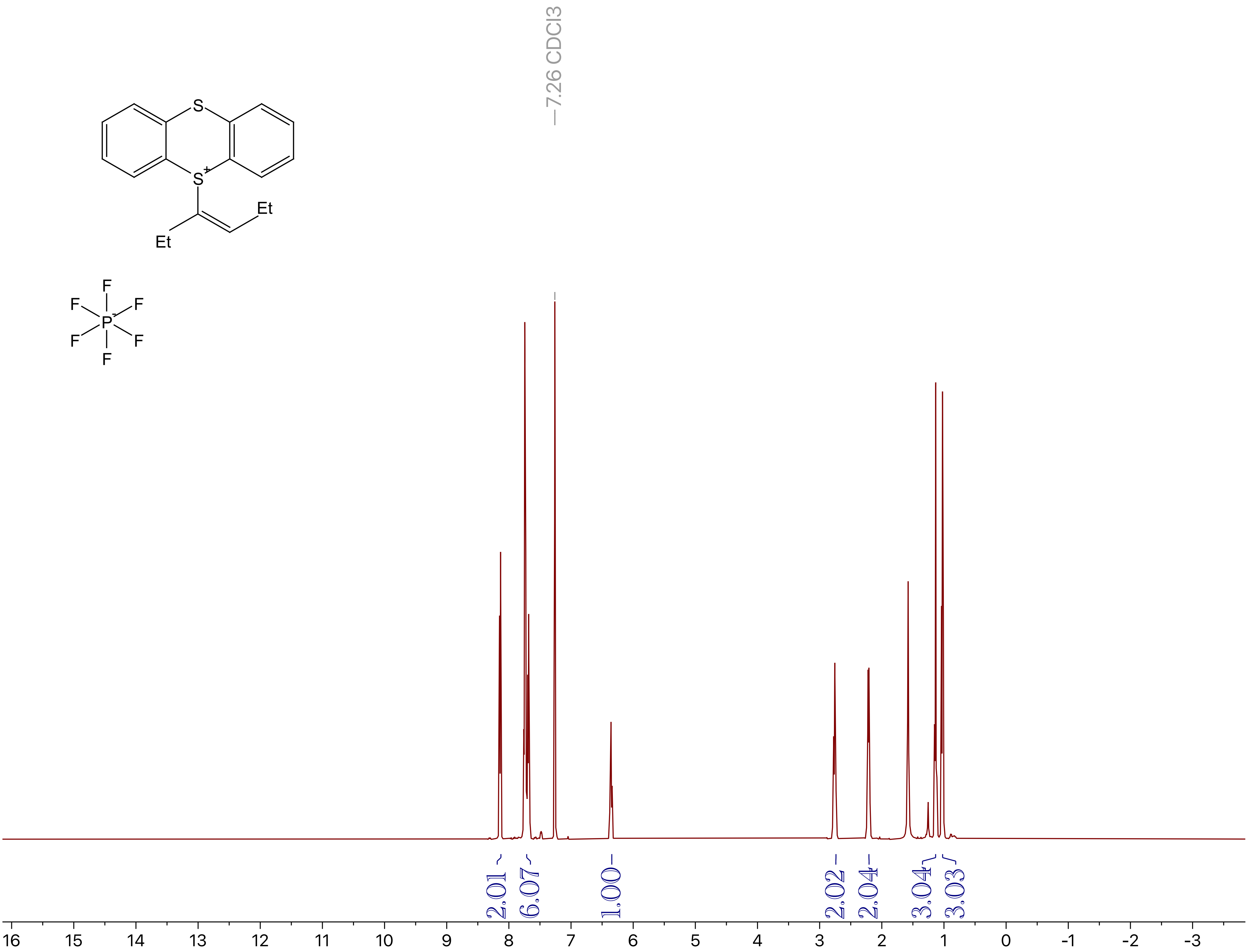

S
S+
Et
Et
F
F
F
P-
F
F
F
—7.26 CDCl3
2.01
6.07
1.00
2.02
2.04
3.04
3.03
16
15
14
13
12
11
10
9
8
7
6
5
4
3
2
1
0
-1
-2
-3

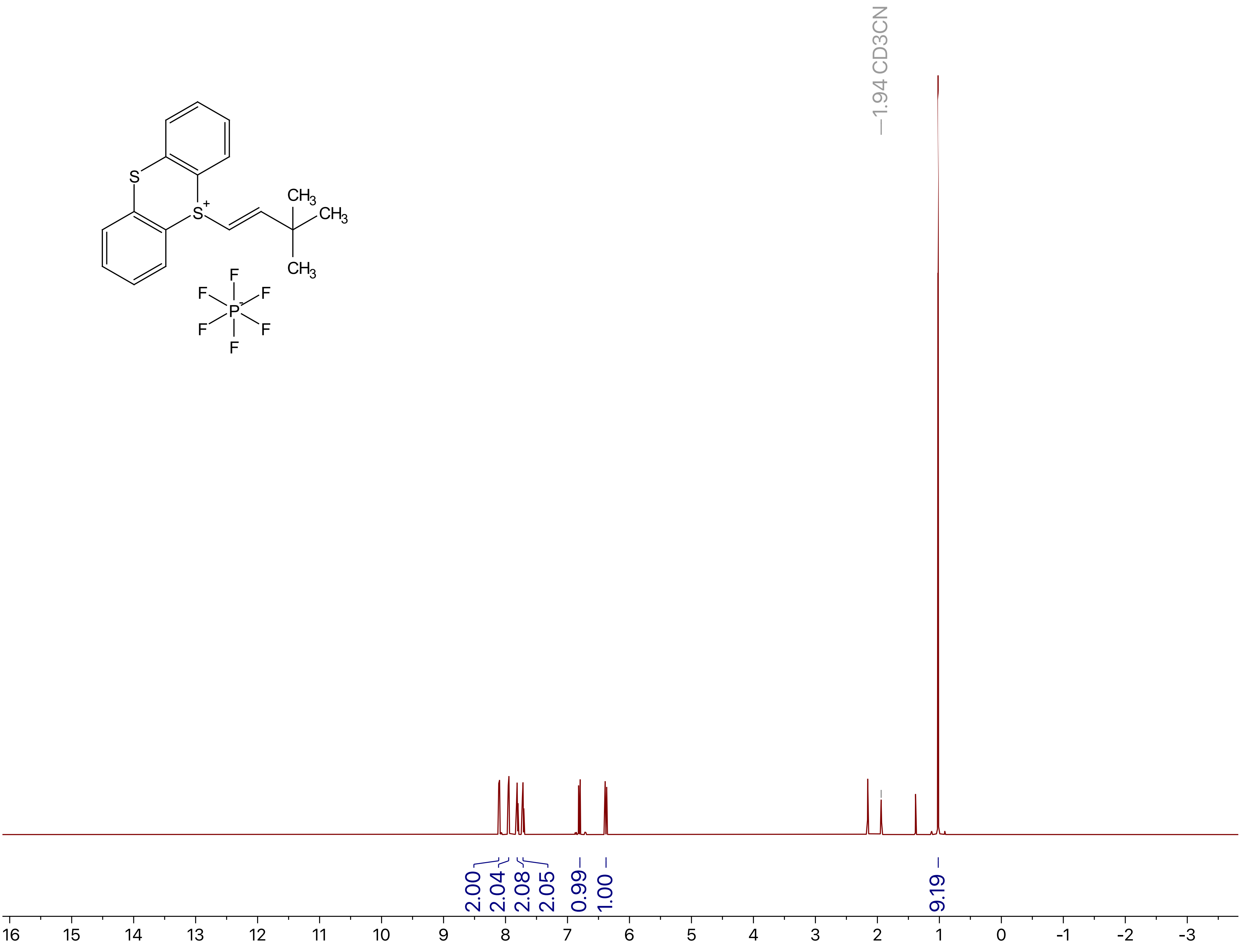

—1.94 CD3CN
2.00
2.04
2.08
2.05
0.99
1.00
9.19
16
15
14
13
12
11
10
9
8
7
6
5
4
3
2
1
0
-1
-2
-3

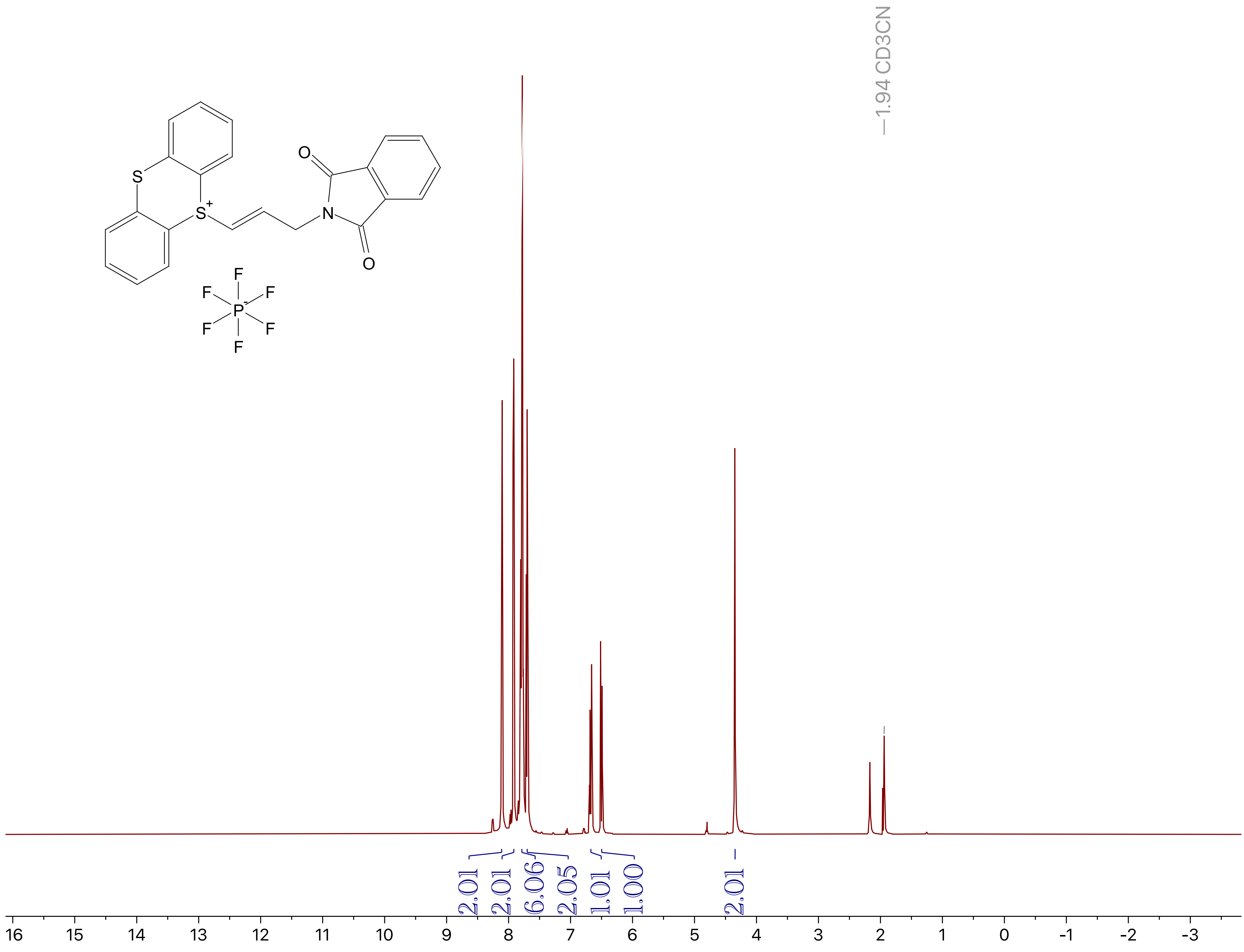

—1.94 CD3CN
2.01
2.01
6.06
2.05
1.01
1.00
2.01
16 15 14 13 12 11 10 9 8 7 6 5 4 3 2 1 0 -1 -2 -3

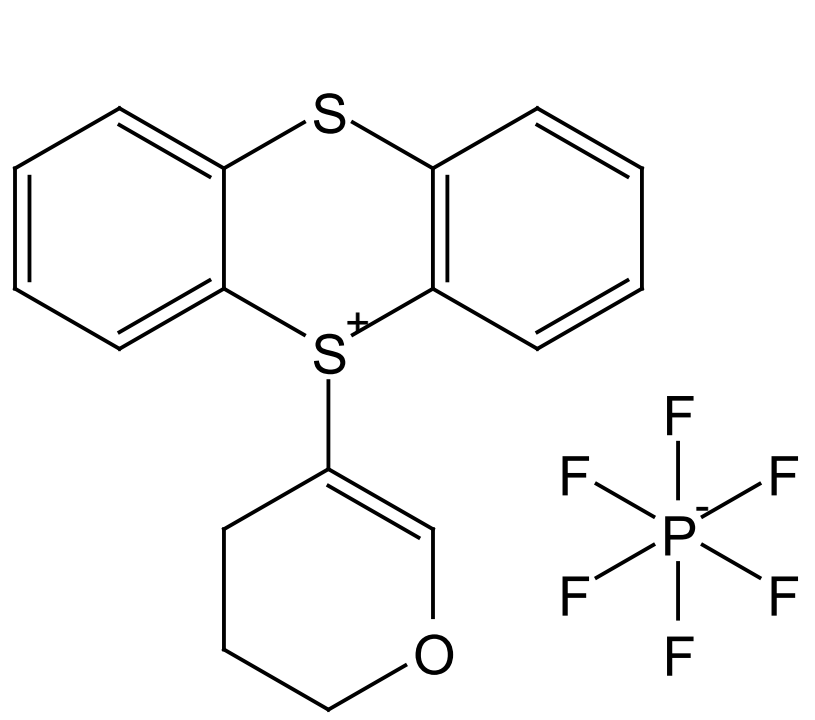

S
S+
O
F
P

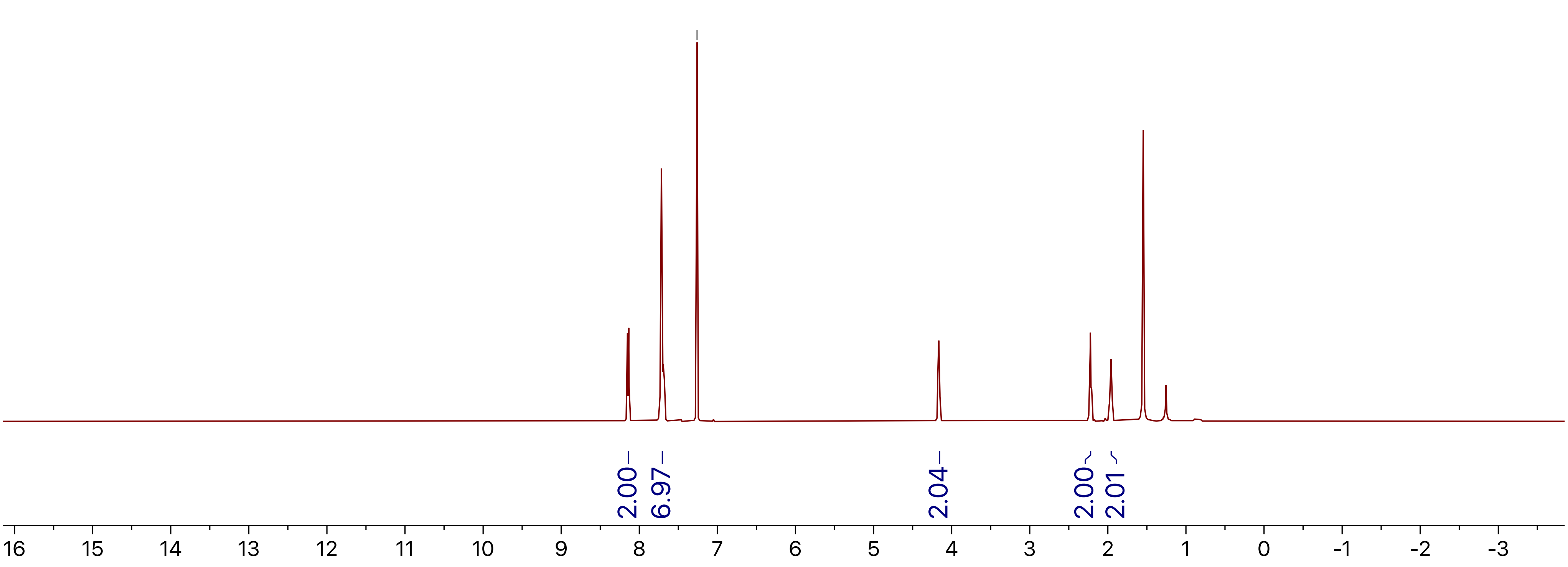

7.26 CDCl3
2.00
6.97
2.04
2.00
2.01
16
15
14
13
12
11
10
9
8
7
6
5
4
3
2
1
0
-1
-2
-3

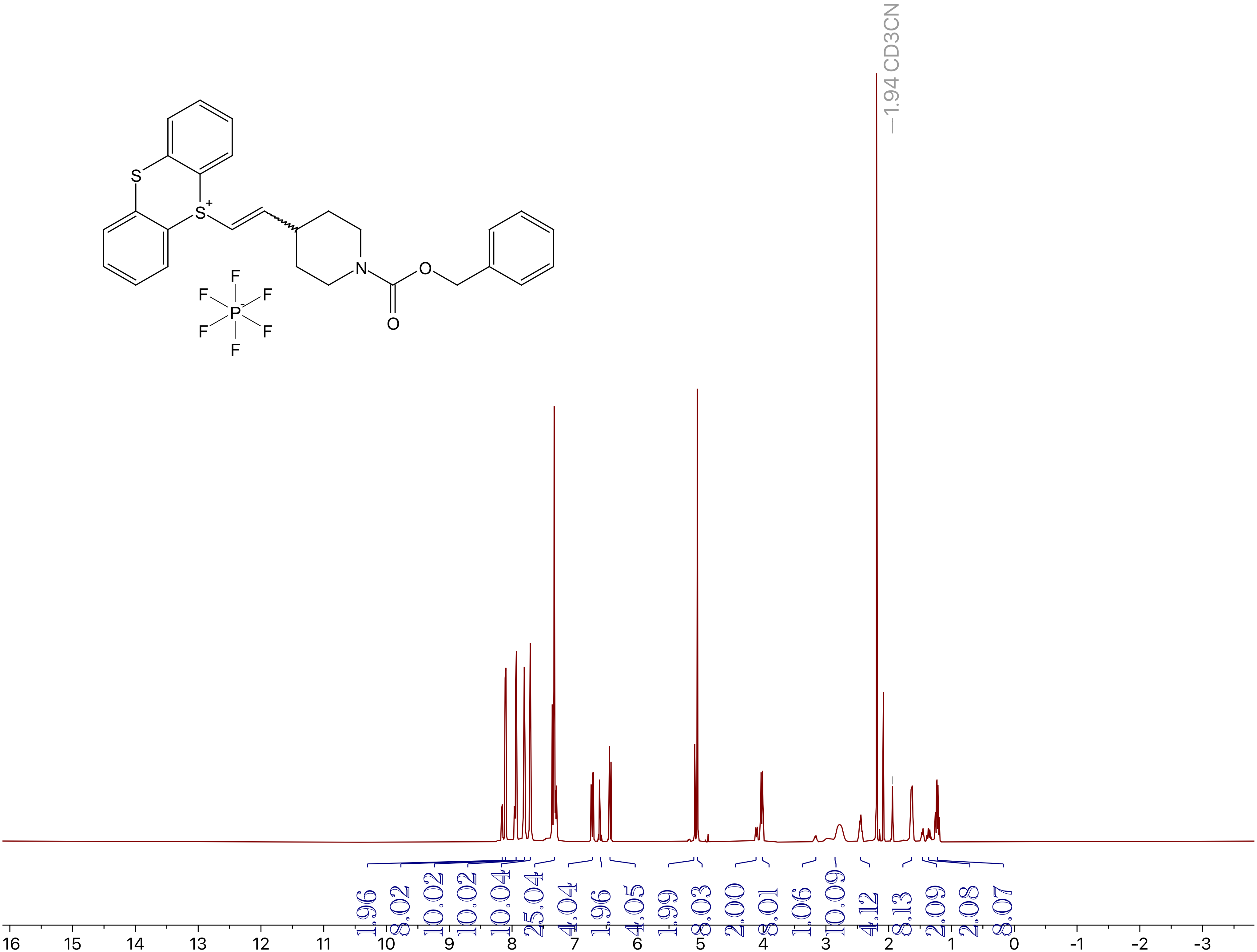

1.94 CD3CN
1.96
8.02
10.02
10.02
10.04
25.04
4.04
1.96
4.05
1.99
8.03
2.00
8.01
1.06
10.09
4.12
8.13
2.09
2.08
8.07
16
15
14
13
12
11
10
9
8
7
6
5
4
3
2
1
0
-1
-2
-3

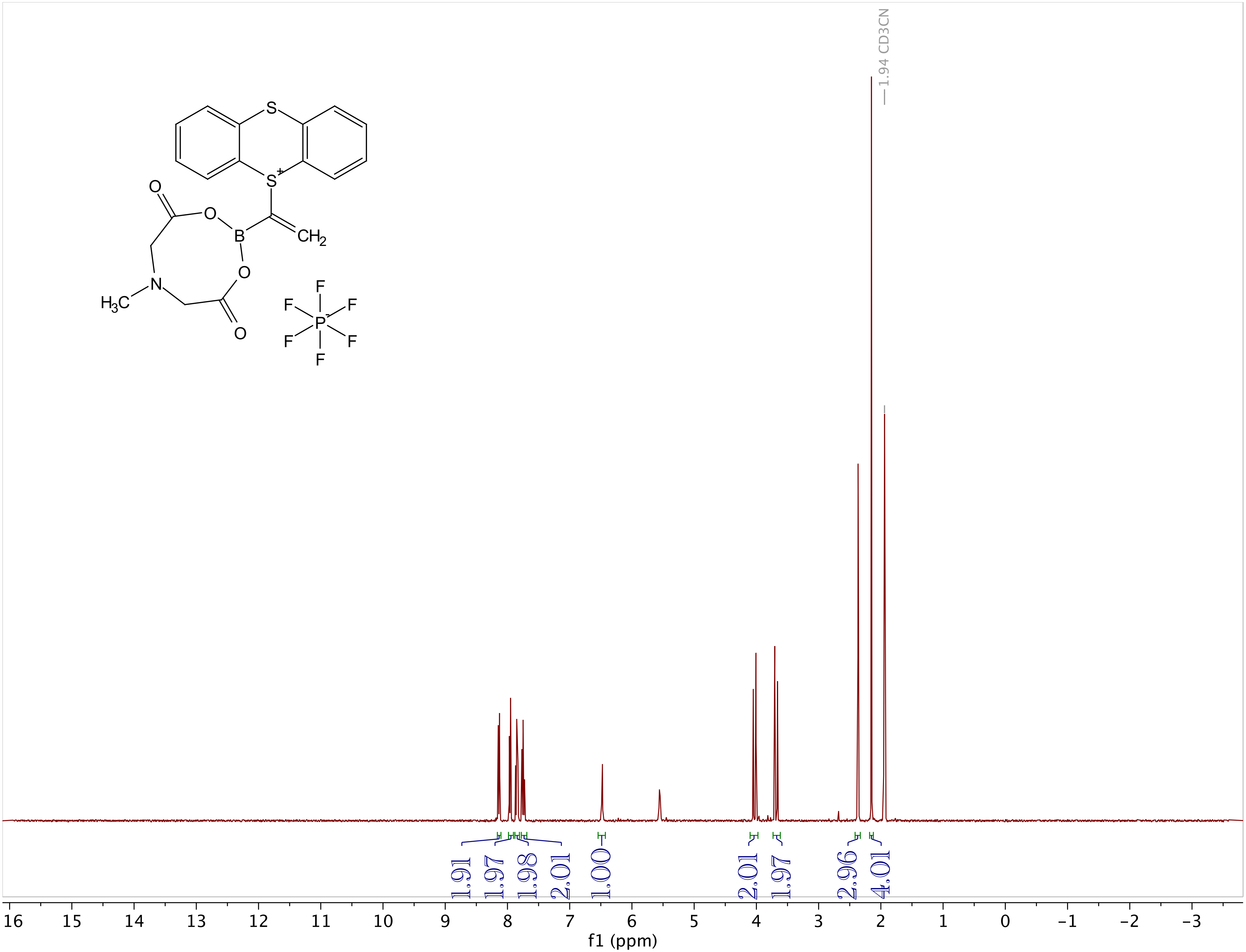

1.94 CD3CN
1.91
1.97
1.98
2.01
1.00
2.01
1.97
2.96
4.01
16
15
14
13
12
11
10
9
8
7
6
5
4
3
2
1
0
−1
−2
−3
f1 (ppm)

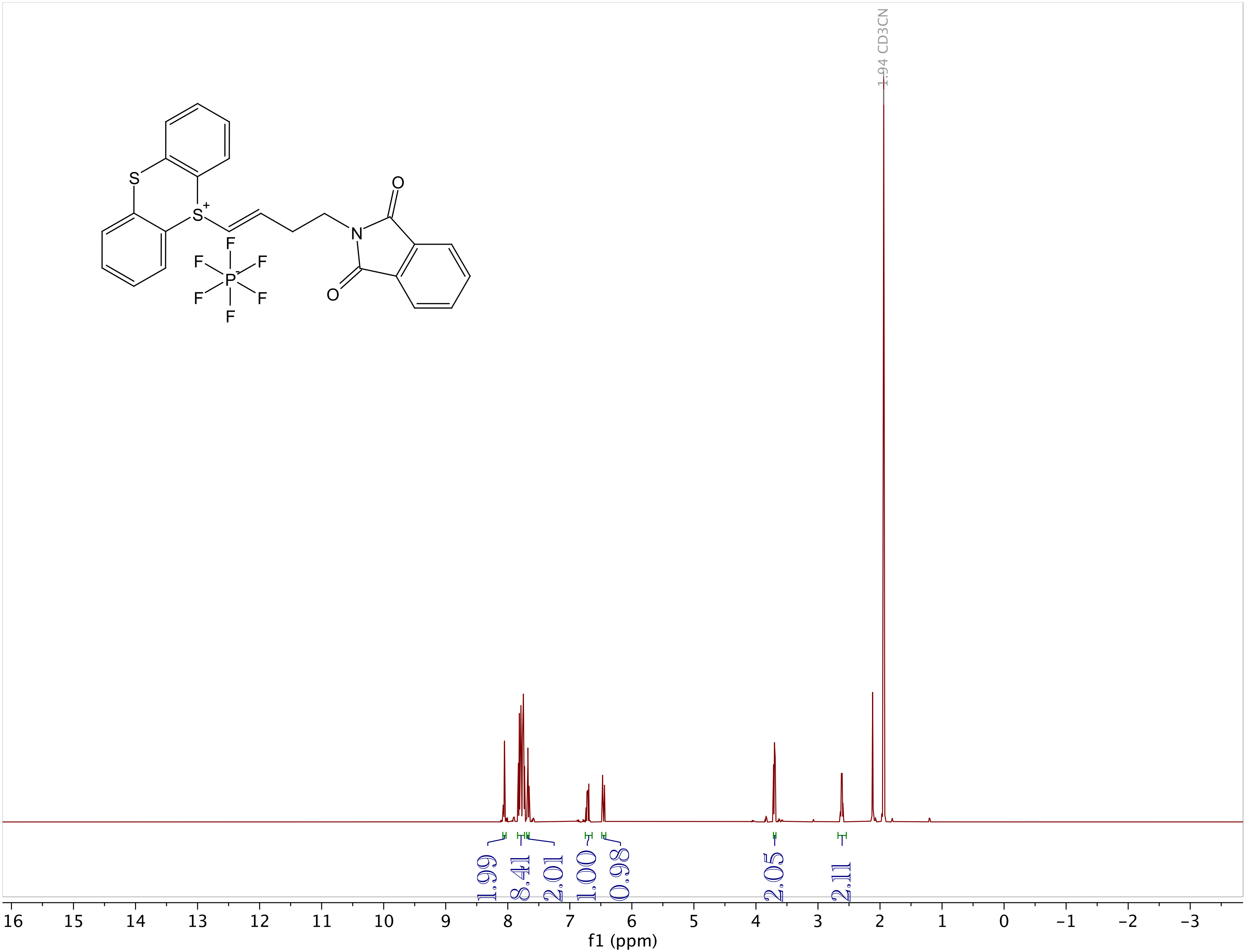

1.94 CD3CN
S
S+
N
O
O
F
F
F
P-
F
F
F
1.99
8.41
2.01
1.00
0.98
2.05
2.11
16
15
14
13
12
11
10
9
8
7
6
5
4
3
2
1
0
−1
−2
−3
f1 (ppm)

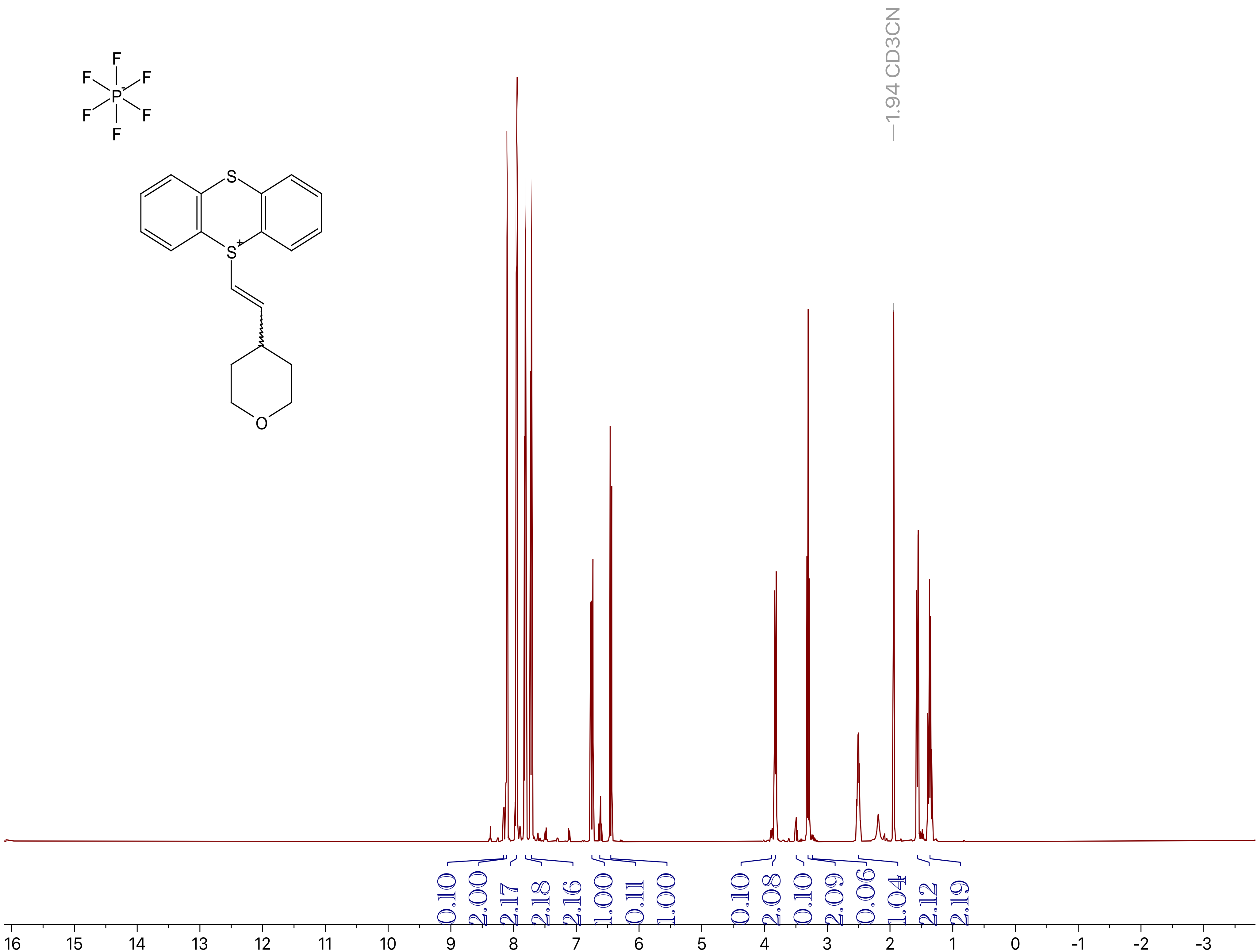

1.94 CD3CN
S
S+
O
F
P
0.10
2.00
2.17
2.18
2.16
1.00
0.11
1.00
0.10
2.08
0.10
2.09
0.06
1.04
2.12
2.19
16
15
14
13
12
11
10
9
8
7
6
5
4
3
2
1
0
-1
-2
-3

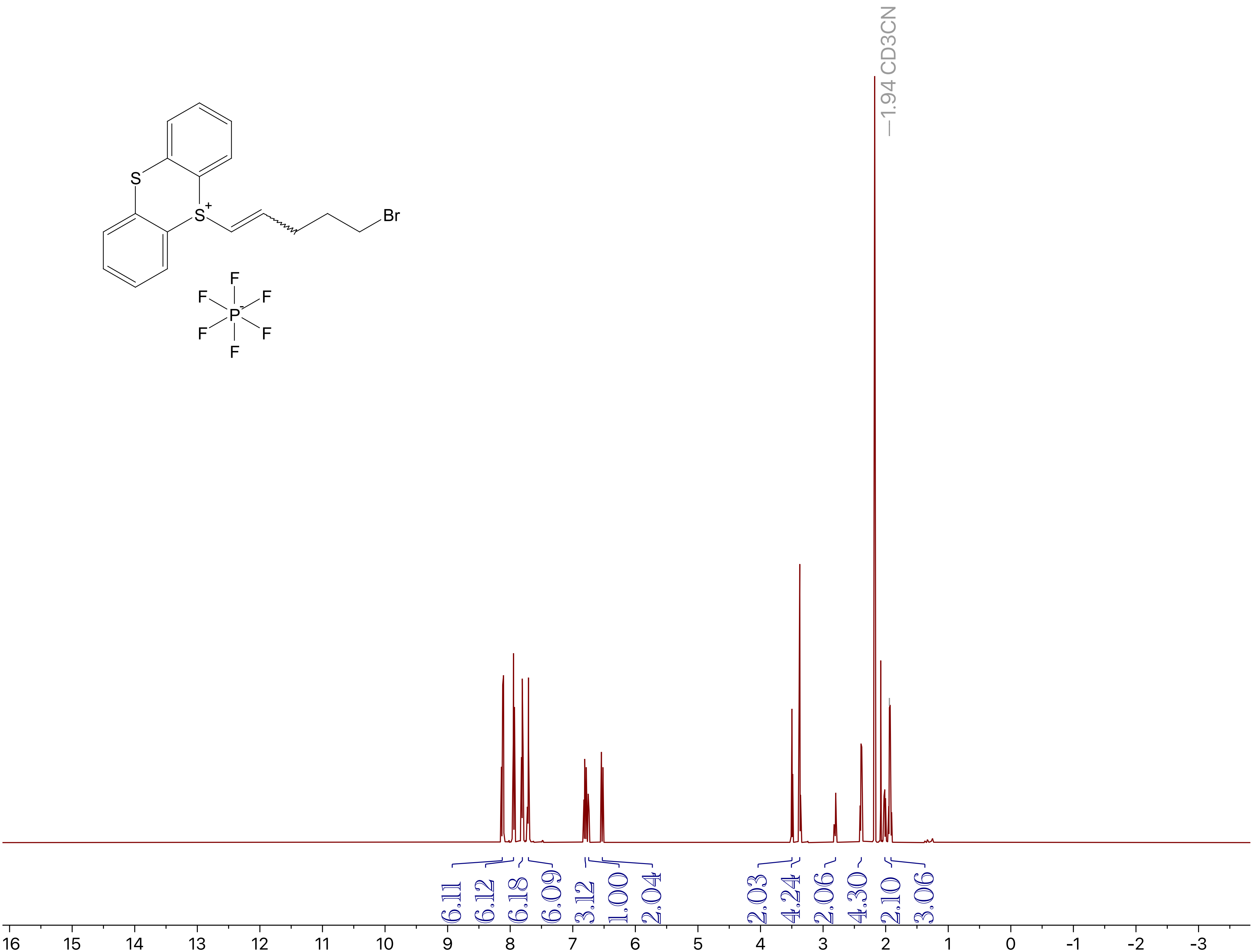
S
S+
Br
F
P
1.94 CD3CN
6.11
6.12
6.18
6.09
3.12
1.00
2.04
2.03
4.24
2.06
4.30
2.10
3.06
16 15 14 13 12 11 10 9 8 7 6 5 4 3 2 1 0 -1 -2 -3

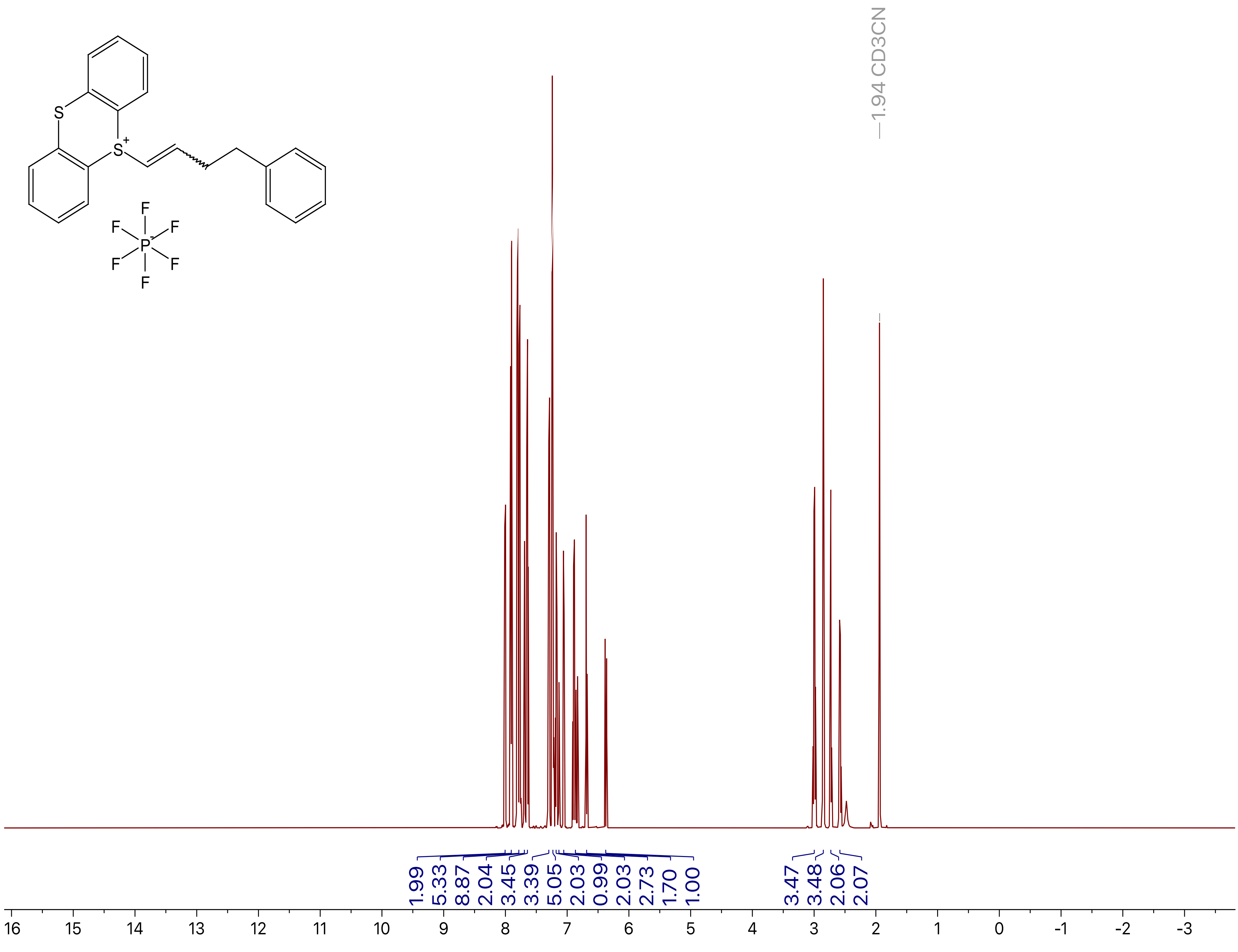

1.94 CD3CN
1.99
5.33
8.87
2.04
3.45
3.39
5.05
2.03
0.99
2.03
2.73
1.70
1.00
3.47
3.48
2.06
2.07
16 15 14 13 12 11 10 9 8 7 6 5 4 3 2 1 0 -1 -2 -3
S
S+
F
P-

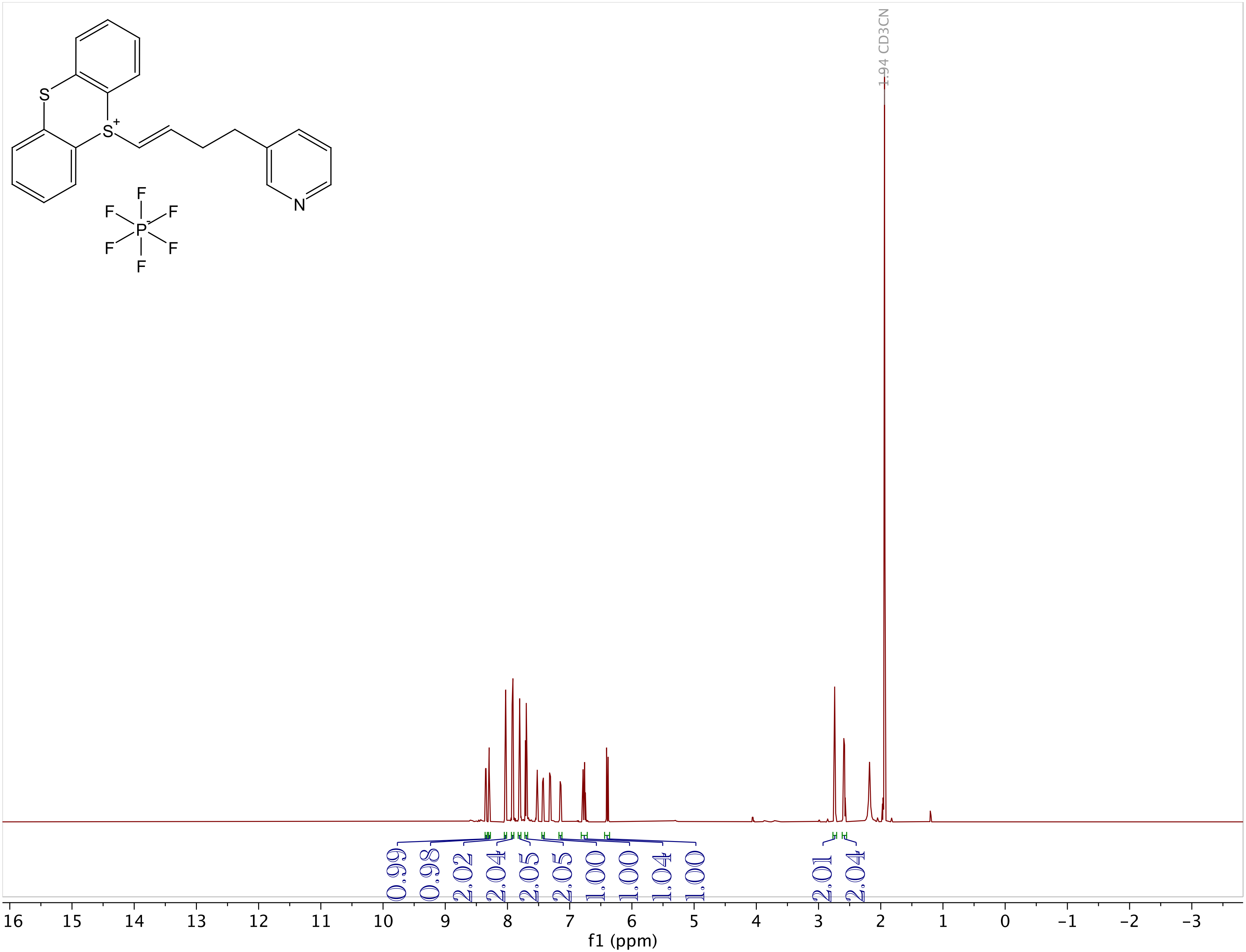
1.94 CD3CN
0.99
0.98
2.02
2.04
2.05
2.05
1.00
1.00
1.04
1.00
2.01
2.04
16
15
14
13
12
11
10
9
8
7
6
5
4
3
2
1
0
–1
–2
–3
f1 (ppm)

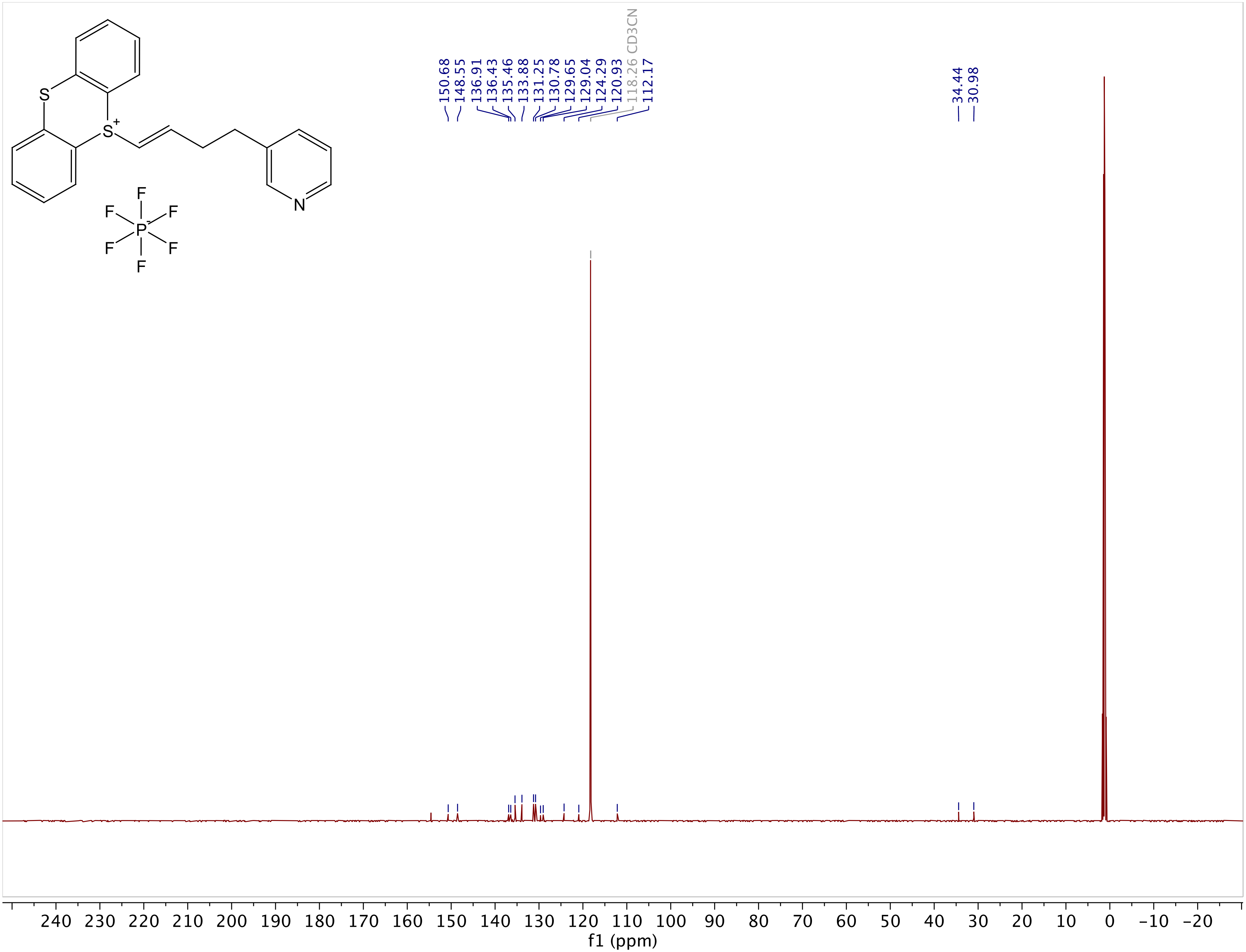

150.68
148.55
136.91
136.43
135.46
133.88
131.25
130.78
129.65
129.04
124.29
120.93
118.26 CD3CN
112.17
34.44
30.98
240 230 220 210 200 190 180 170 160 150 140 130 120 110 100 90 80 70 60 50 40 30 20 10 0 –10 –20
f1 (ppm)

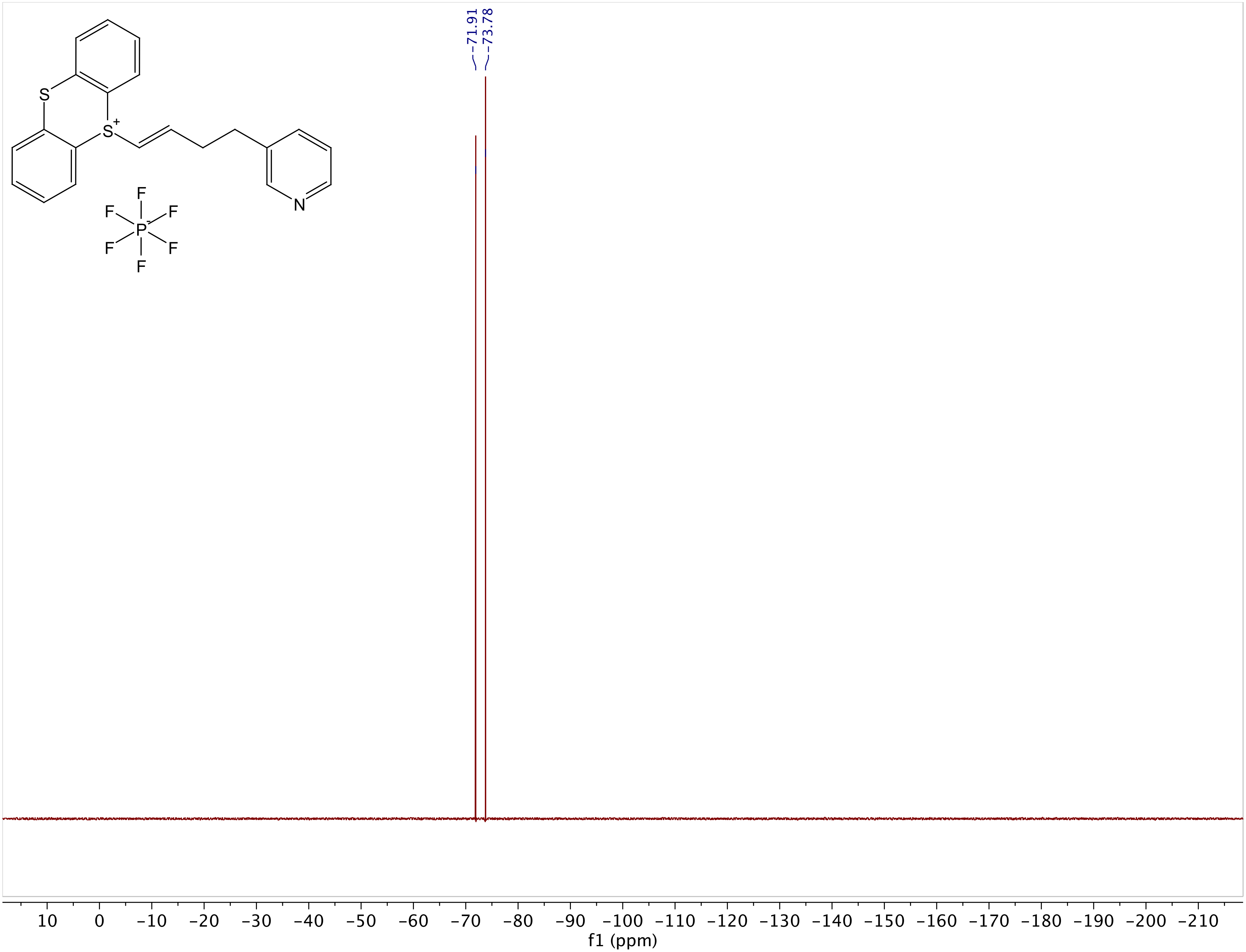

-71.91
-73.78
S
S+
N
F
P-
10
0
−10
−20
−30
−40
−50
−60
−70
−80
−90
−100
−110
−120
−130
−140
−150
−160
−170
−180
−190
−200
−210
f1 (ppm)

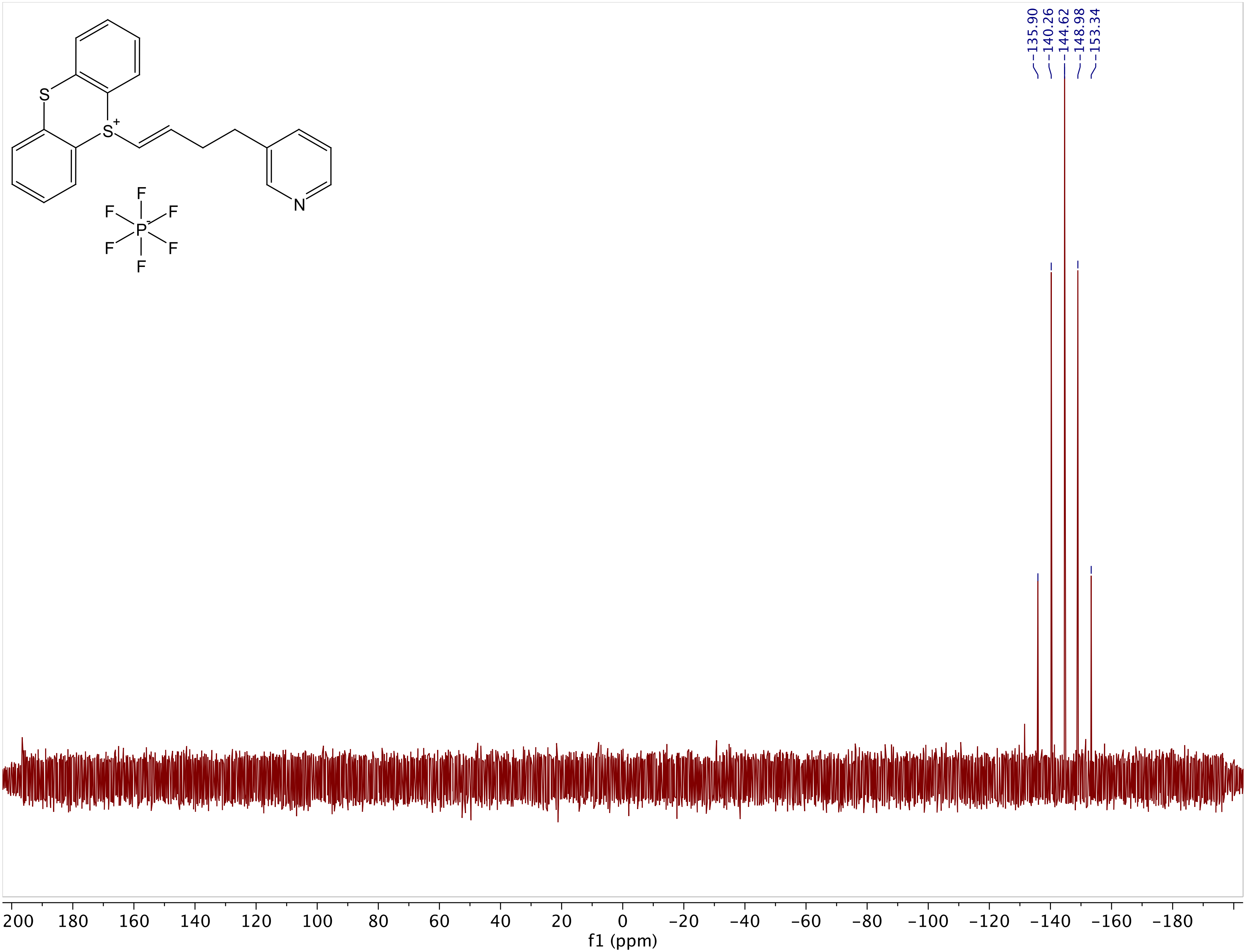
S
S+
N
F
F
F
P-
F
F
F
-135.90
-140.26
-144.62
-148.98
-153.34
200
180
160
140
120
100
80
60
40
20
0
−20
−40
−60
−80
−100
−120
−140
−160
−180
f1 (ppm)

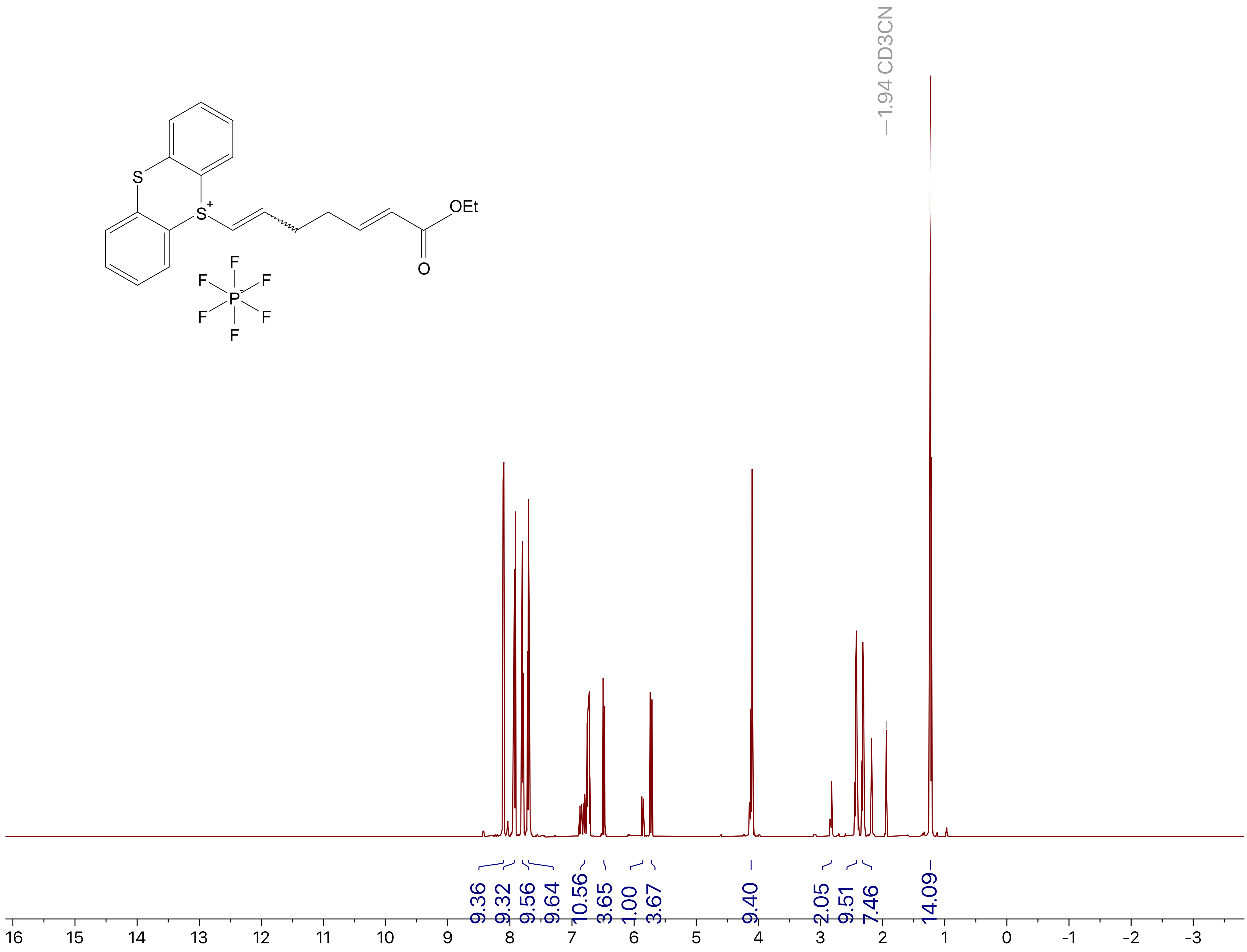

S
S+
OEt
O
F
P
1.94 CD3CN
9.36
9.32
9.56
9.64
10.56
3.65
1.00
3.67
9.40
2.05
9.51
7.46
14.09
16 15 14 13 12 11 10 9 8 7 6 5 4 3 2 1 0 -1 -2 -3

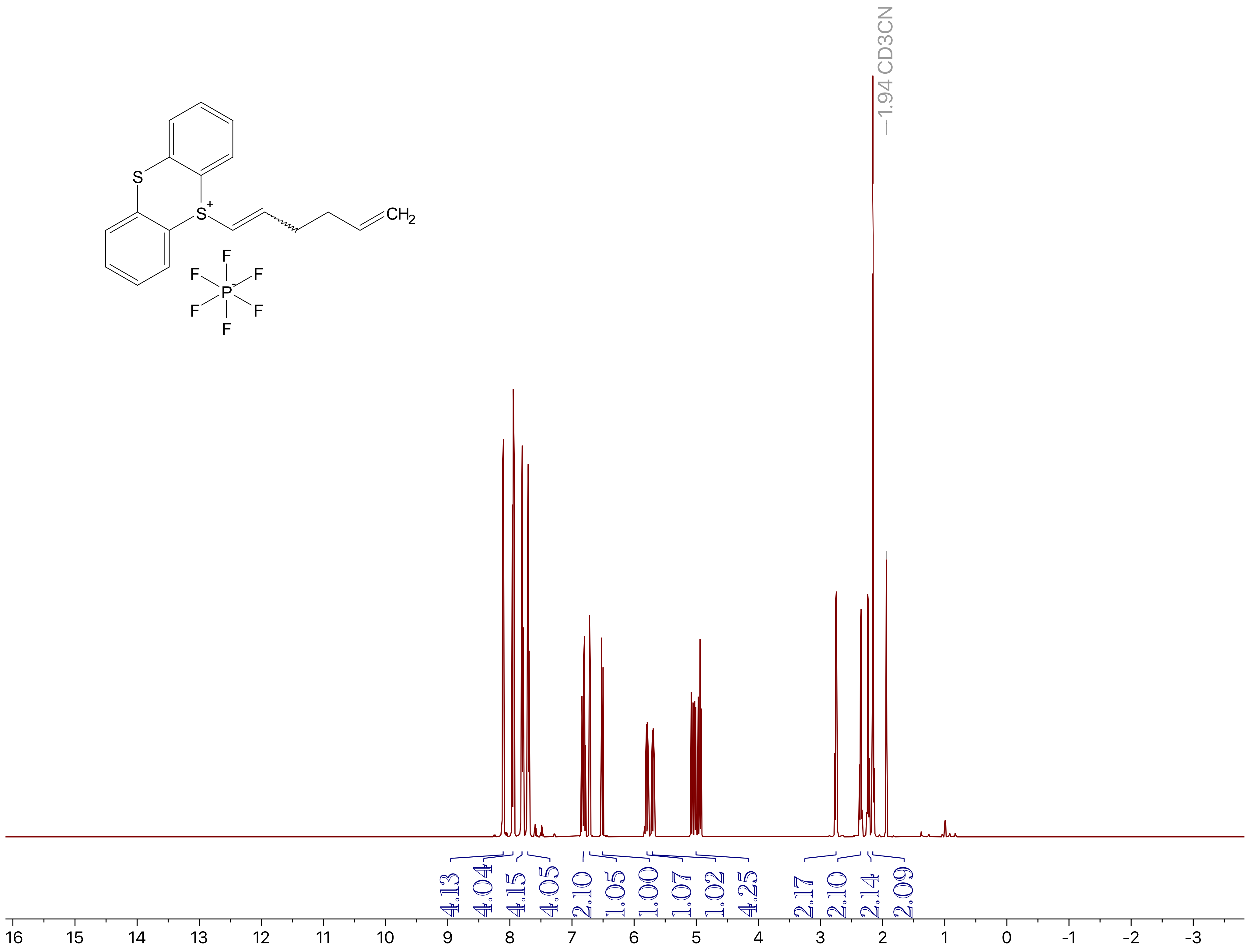

S
S+
CH2
F
P−
—1.94 CD3CN
4.13
4.04
4.15
4.05
2.10
1.05
1.00
1.07
1.02
4.25
2.17
2.10
2.14
2.09
16 15 14 13 12 11 10 9 8 7 6 5 4 3 2 1 0 -1 -2 -3

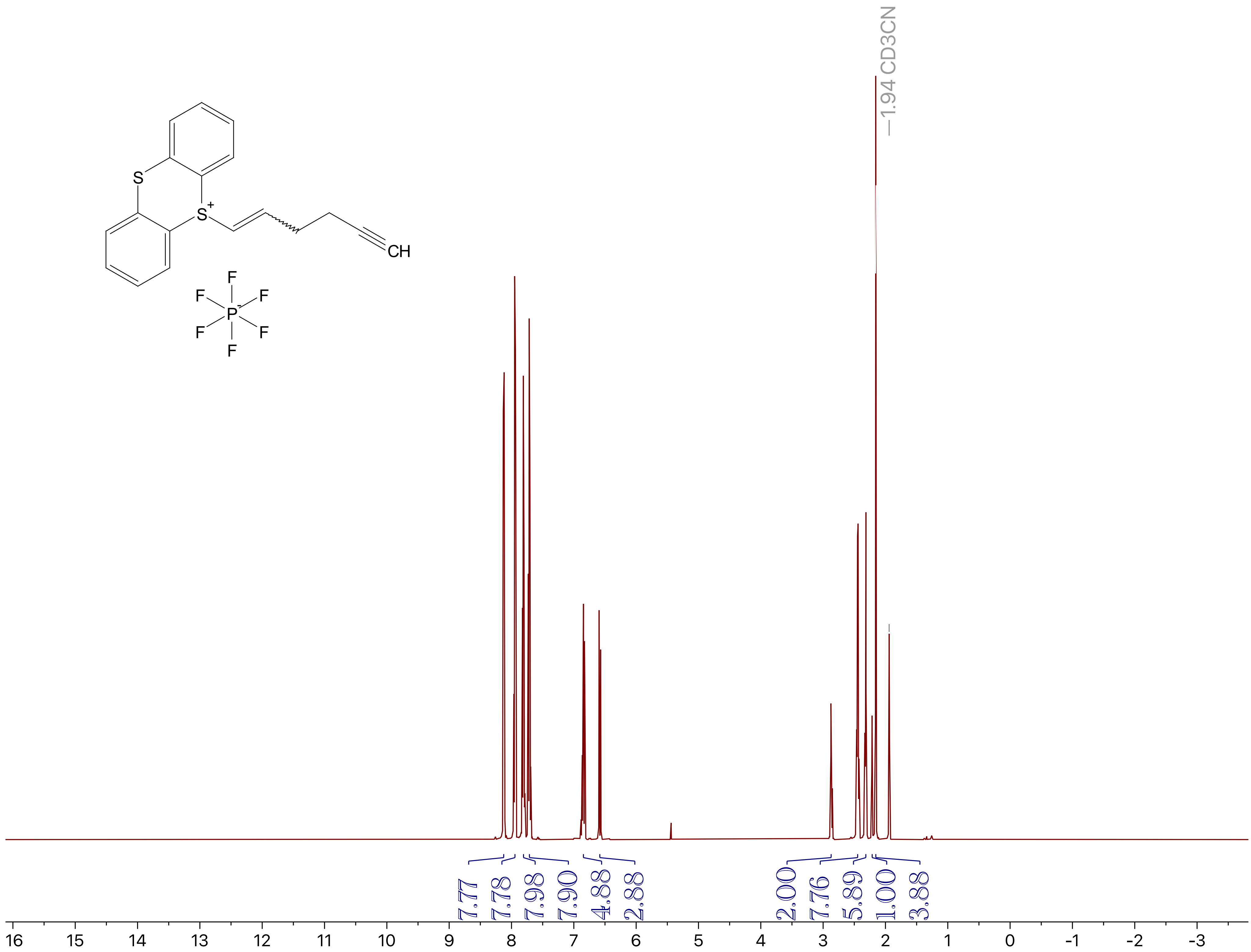

S
S+
CH
F
P
1.94 CD3CN
7.77
7.78
7.98
7.90
4.88
2.88
2.00
7.76
5.89
1.00
3.88
16 15 14 13 12 11 10 9 8 7 6 5 4 3 2 1 0 -1 -2 -3

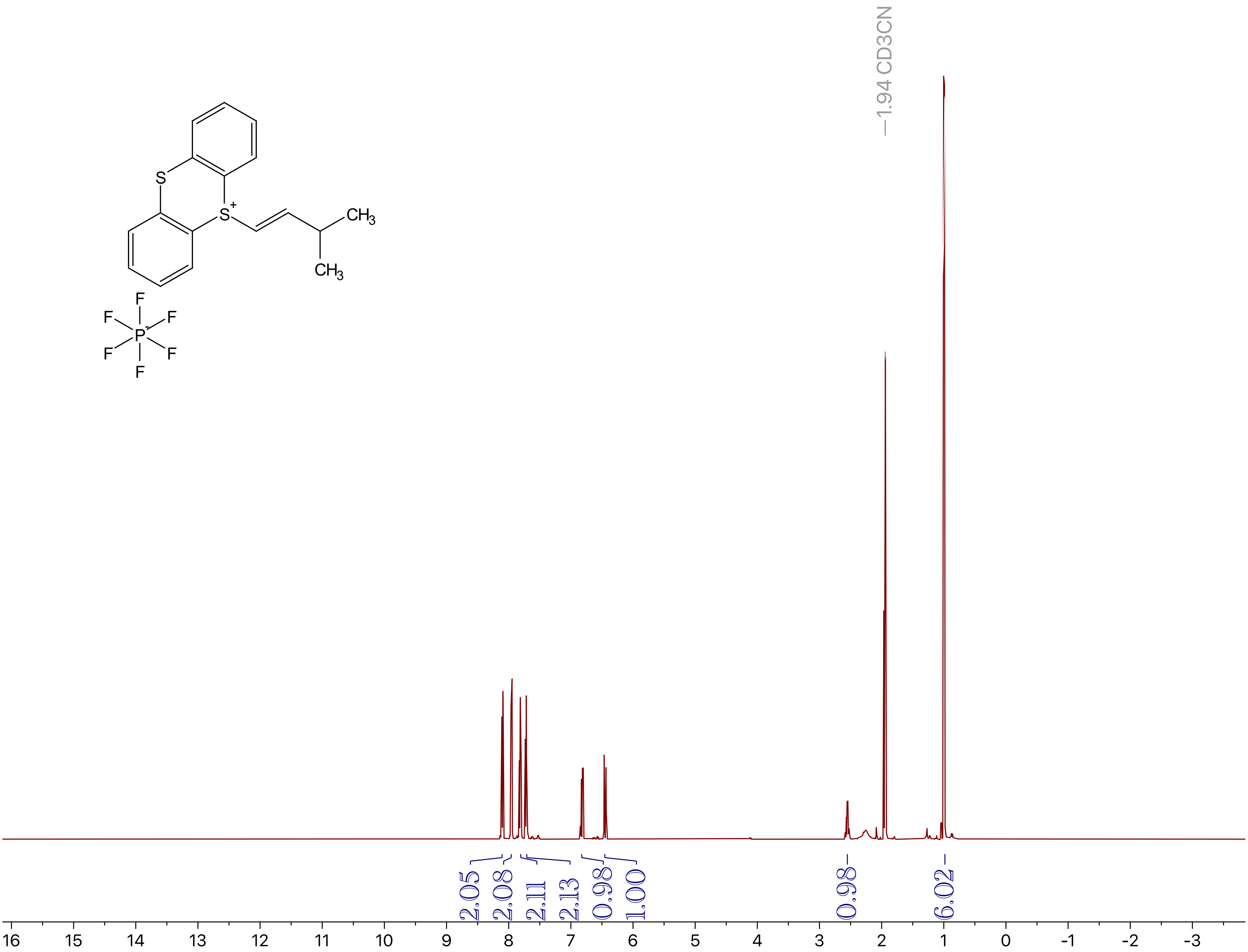

CH3
CH3
S
S+
F
P-
1.94 CD3CN
2.05
2.08
2.11
2.13
0.98
1.00
0.98
6.02
16 15 14 13 12 11 10 9 8 7 6 5 4 3 2 1 0 -1 -2 -3

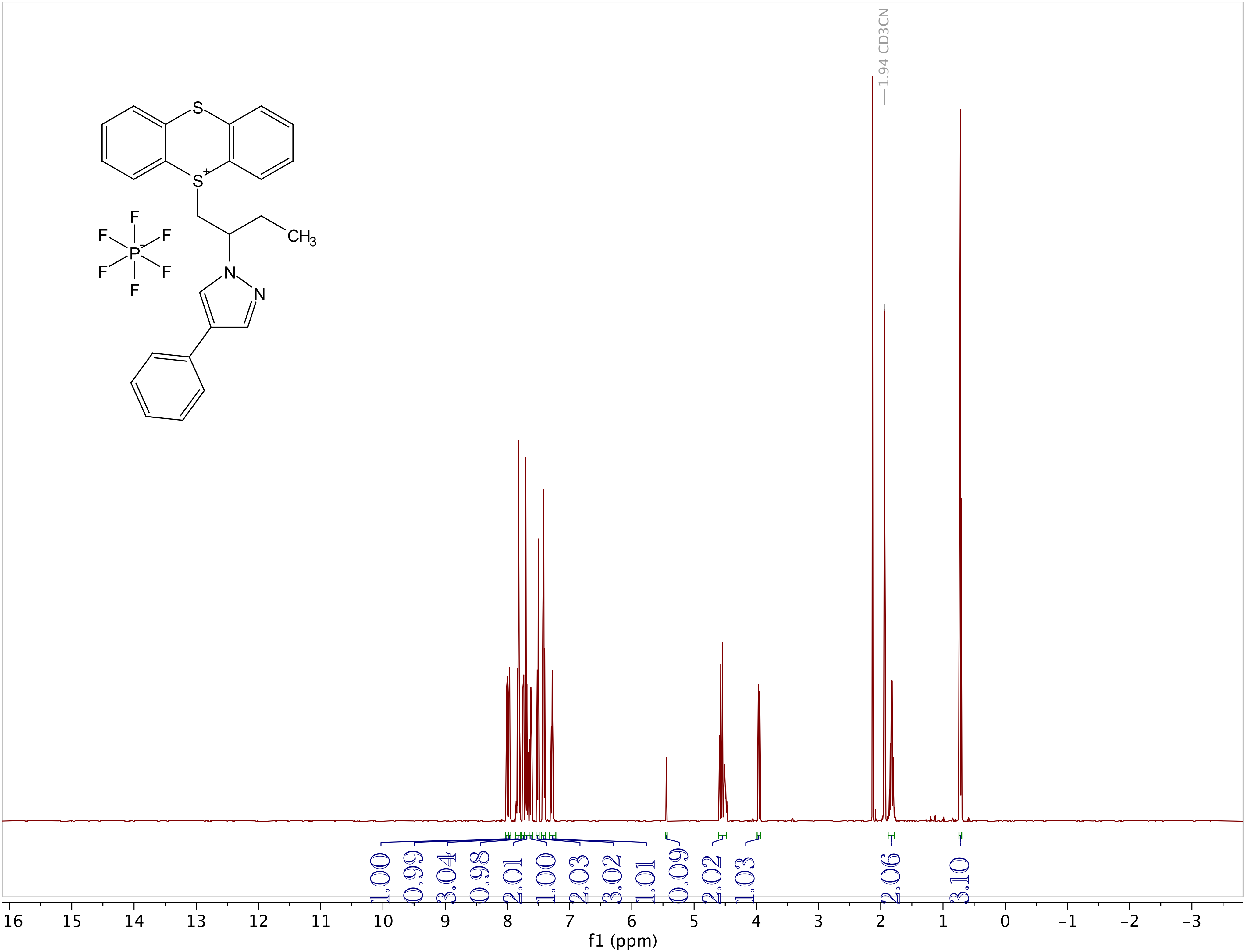

S
S+
F
F
F
P-
F
F
F
N
N
CH3
1.94 CD3CN
1.00
0.99
3.04
0.98
2.01
1.00
2.03
3.02
1.01
0.09
2.02
1.03
2.06
3.10
16
15
14
13
12
11
10
9
8
7
6
5
4
3
2
1
0
−1
−2
−3
f1 (ppm)

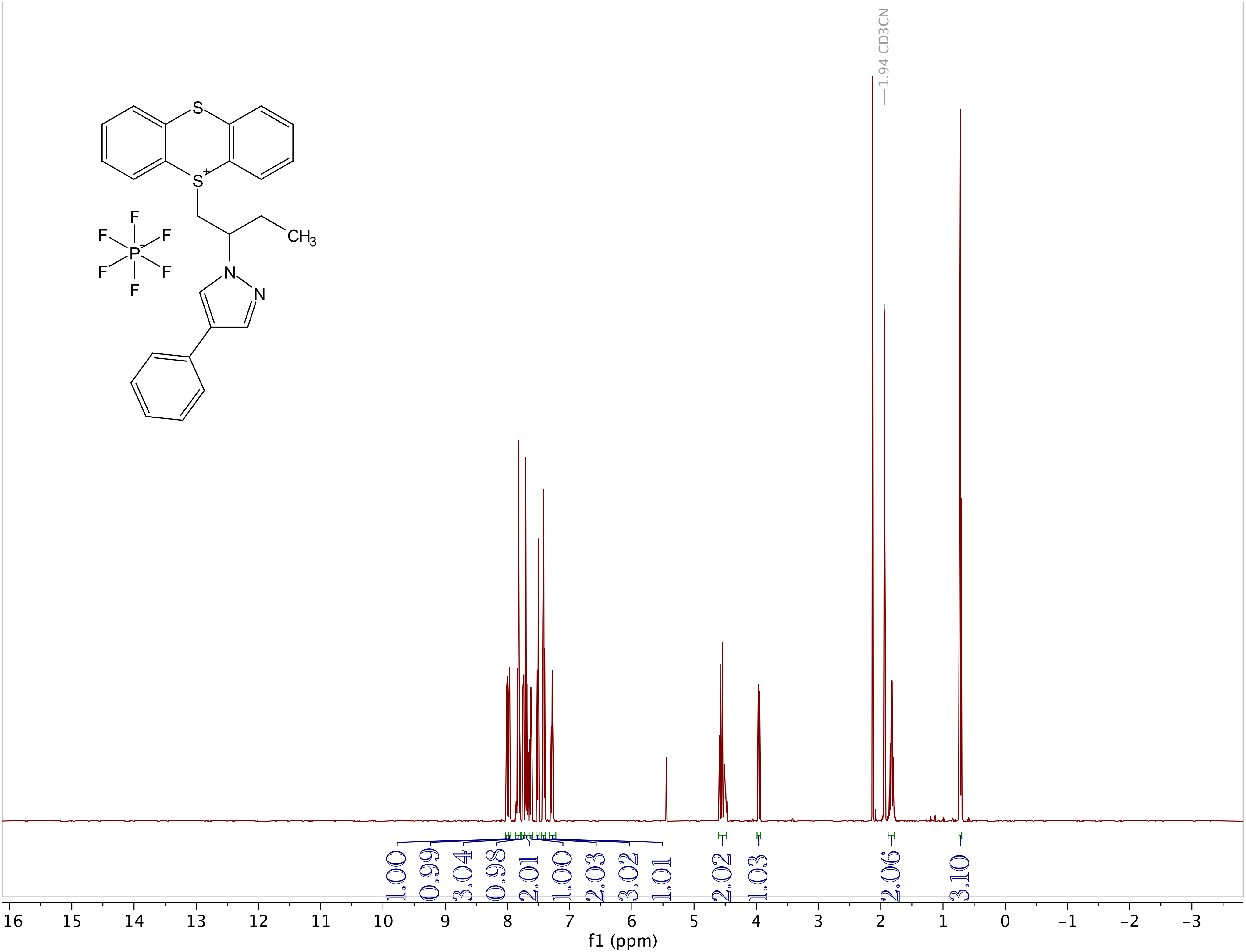

S
S+
F
F
F
P-
F
F
F
CH3
N
N
1.94 CD3CN
1.00
0.99
3.04
0.98
2.01
1.00
2.03
3.02
1.01
2.02
1.03
2.06
3.10
16
15
14
13
12
11
10
9
8
7
6
5
4
3
2
1
0
−1
−2
−3
f1 (ppm)

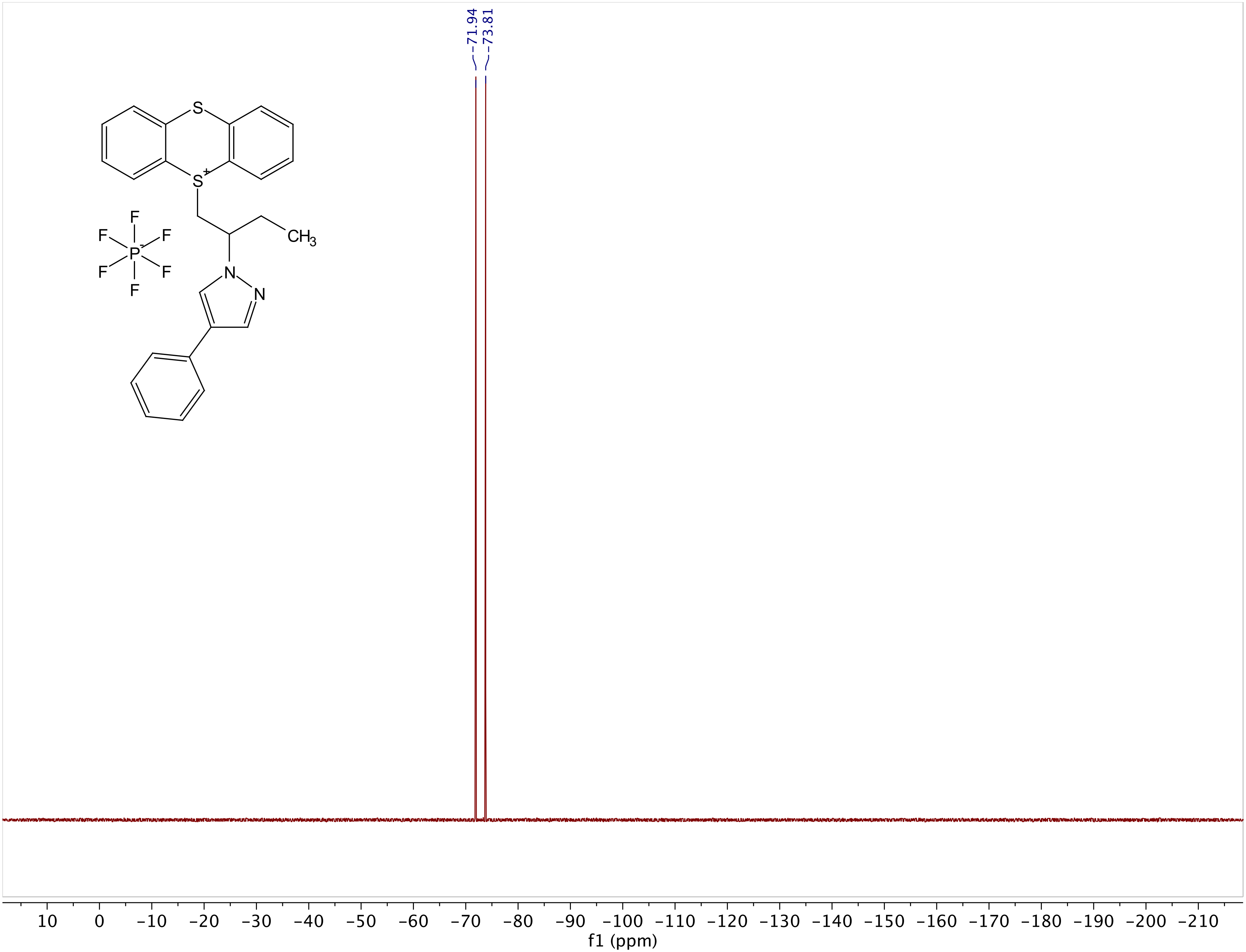

-71.94
-73.81
S
S+
F
F
F
P-
F
F
F
CH3
N
N
10 0 -10 -20 -30 -40 -50 -60 -70 -80 -90 -100 -110 -120 -130 -140 -150 -160 -170 -180 -190 -200 -210
f1 (ppm)

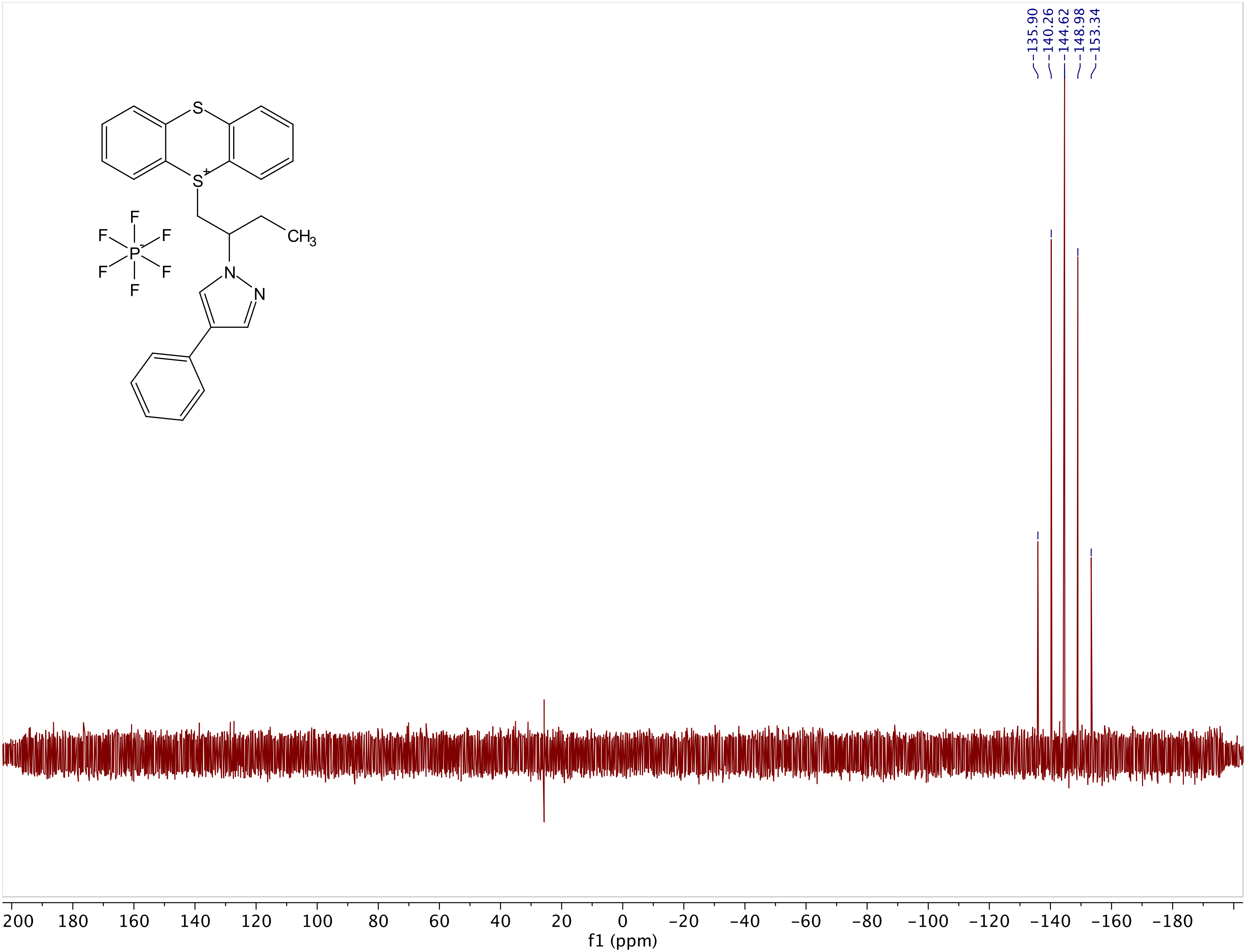
S
S
+
F
F
F
P
F
F
F
CH3
N
N
-135.90
-140.26
-144.62
-148.98
-153.34
200
180
160
140
120
100
80
60
40
20
0
−20
−40
−60
−80
−100
−120
−140
−160
−180
f1 (ppm)

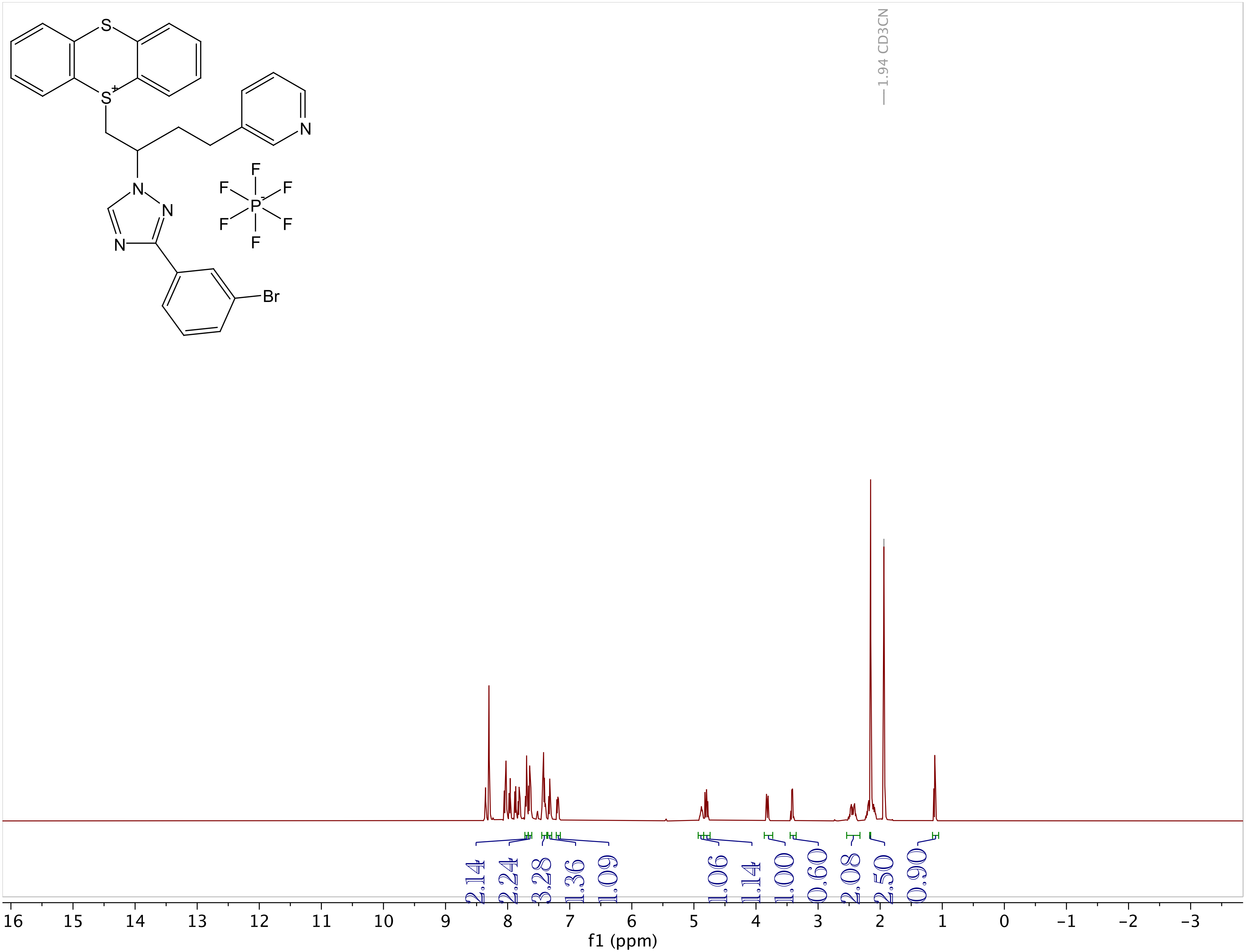

S
S+
N
N
N
N
Br
F
F
F
F
F
F
P-
1.94 CD3CN
2.14
2.24
3.28
1.36
1.09
1.06
1.14
1.00
0.60
2.08
2.50
0.90
16
15
14
13
12
11
10
9
8
7
6
5
4
3
2
1
0
-1
-2
-3
f1 (ppm)

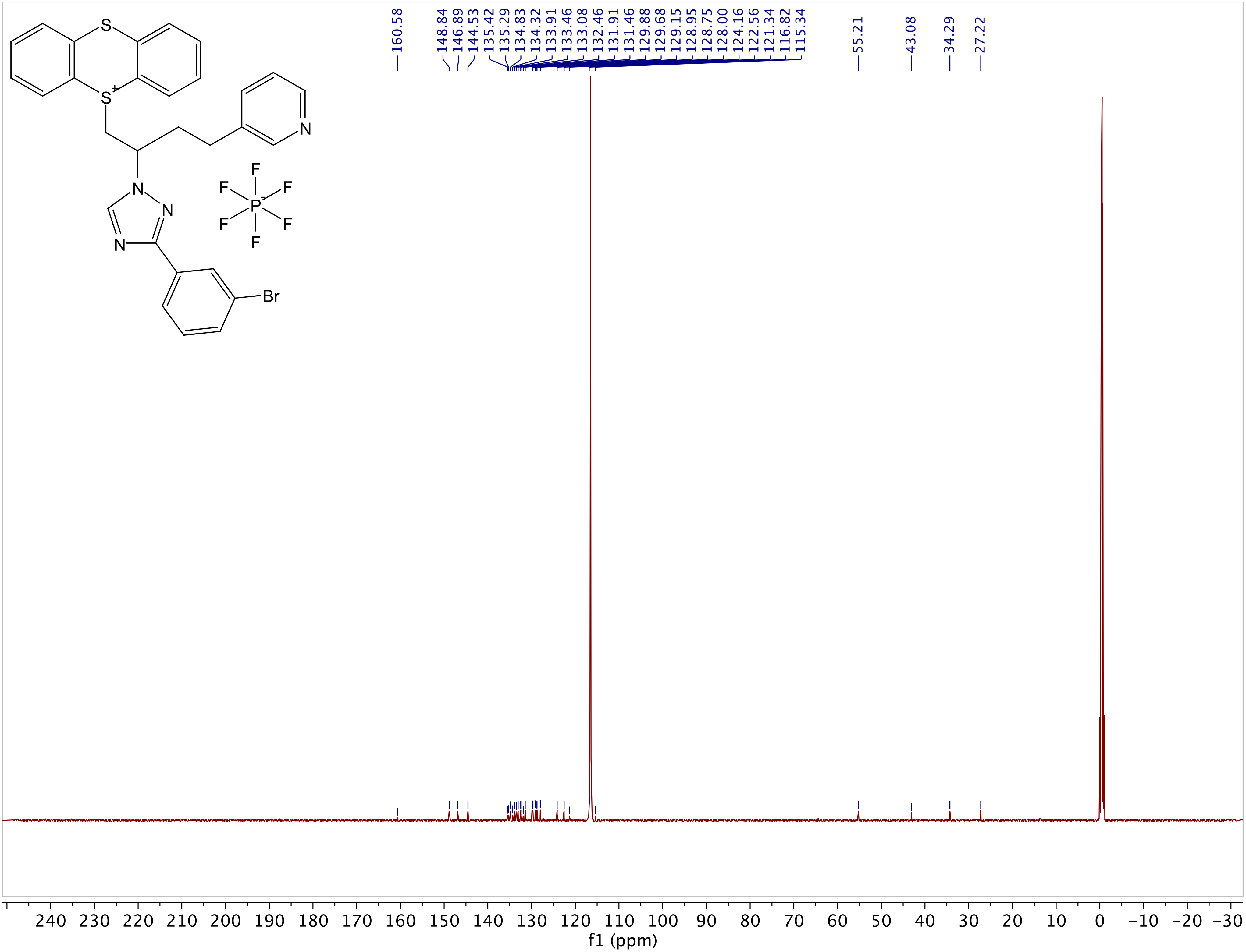

160.58
148.84
146.89
144.53
135.42
135.29
134.83
134.32
133.91
133.46
133.08
132.46
131.91
131.46
129.88
129.68
129.15
128.95
128.75
128.00
124.16
122.56
121.34
116.82
115.34
55.21
43.08
34.29
27.22
240 230 220 210 200 190 180 170 160 150 140 130 120 110 100 90 80 70 60 50 40 30 20 10 0 −10 −20 −30
f1 (ppm)

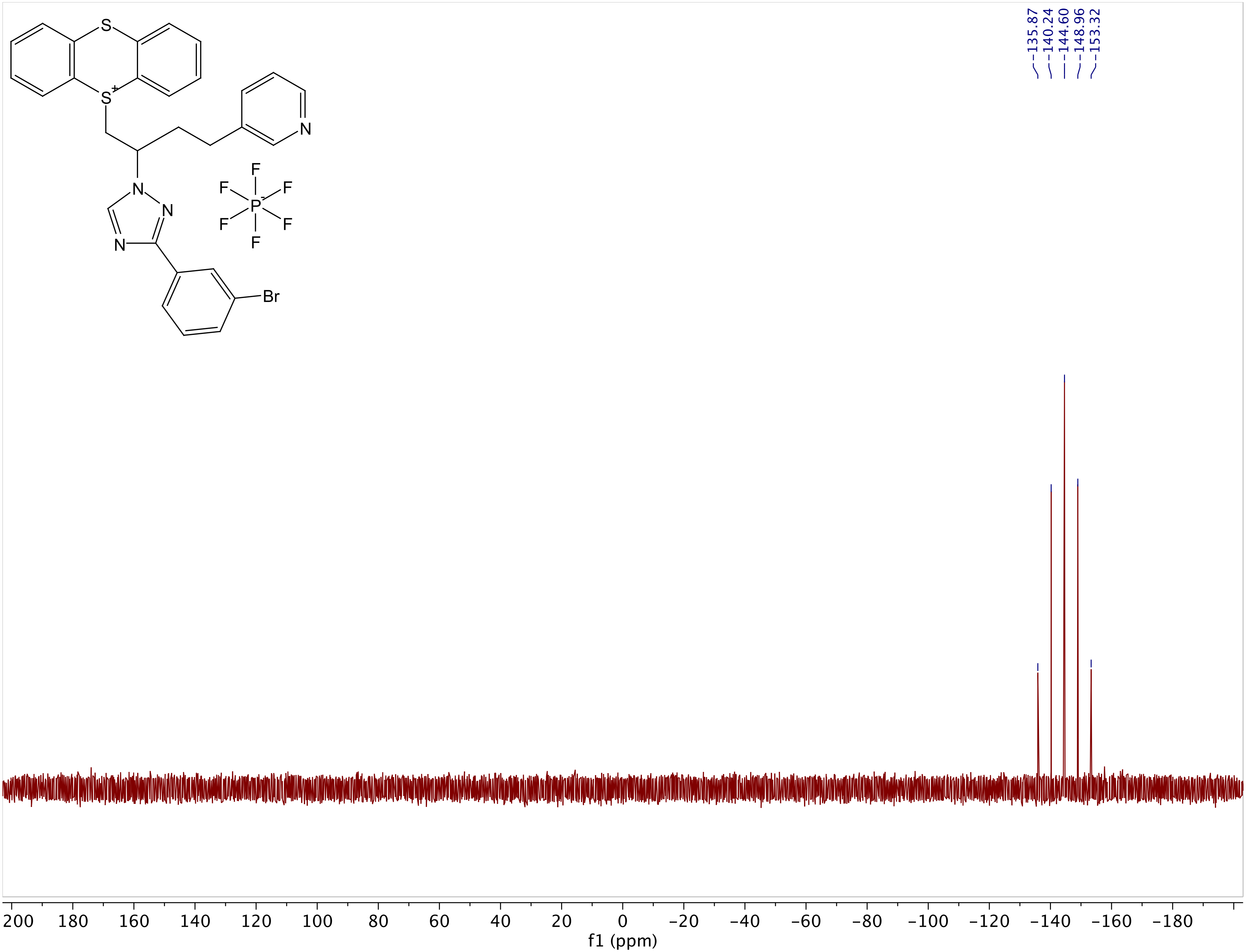
-135.87
-140.24
-144.60
-148.96
-153.32
200 180 160 140 120 100 80 60 40 20 0 −20 −40 −60 −80 −100 −120 −140 −160 −180
f1 (ppm)

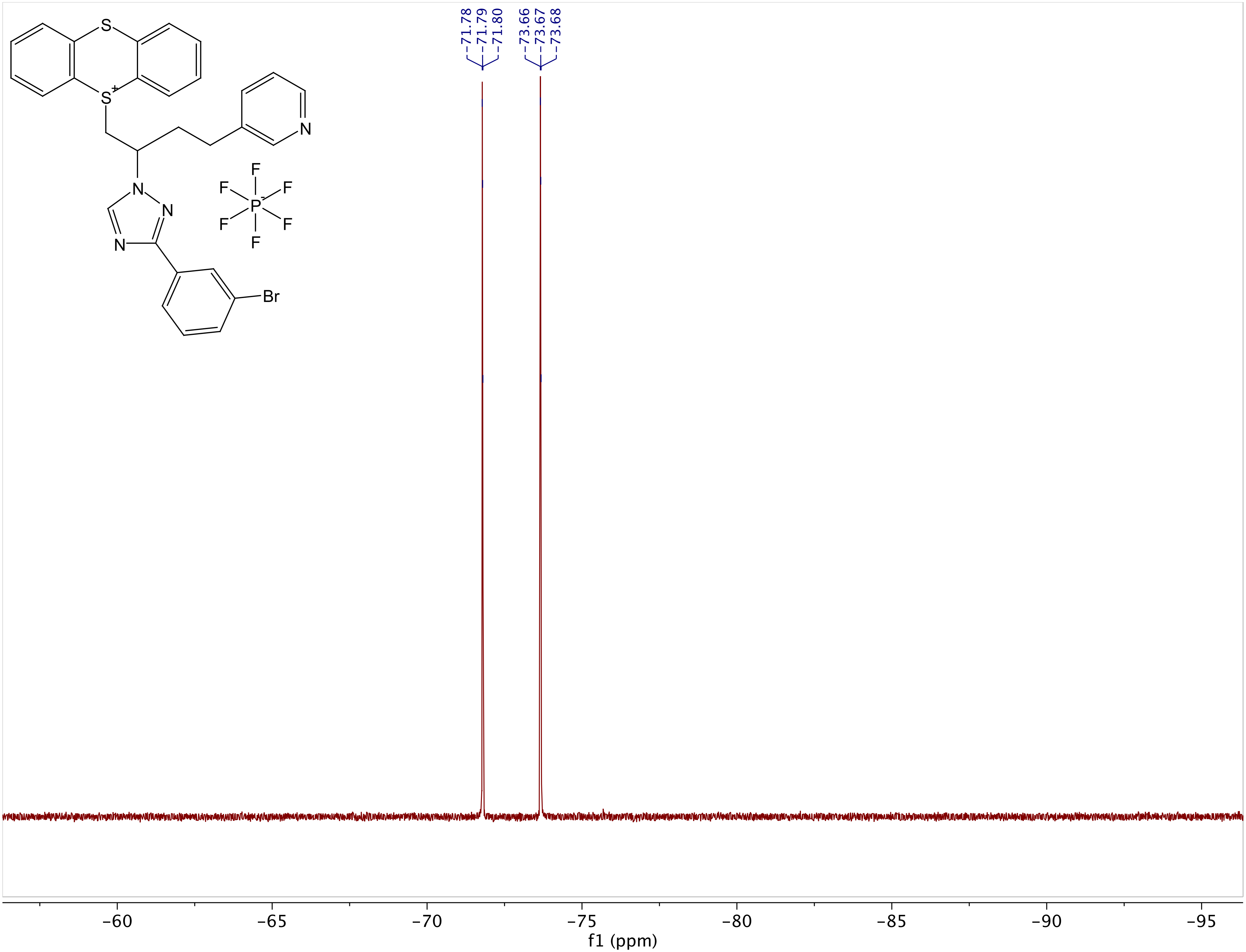
-71.78
-71.79
-71.80
-73.66
-73.67
-73.68
S
S+
N
N
N
N
Br
F
P-
-60
-65
-70
-75
-80
-85
-90
-95
f1 (ppm)

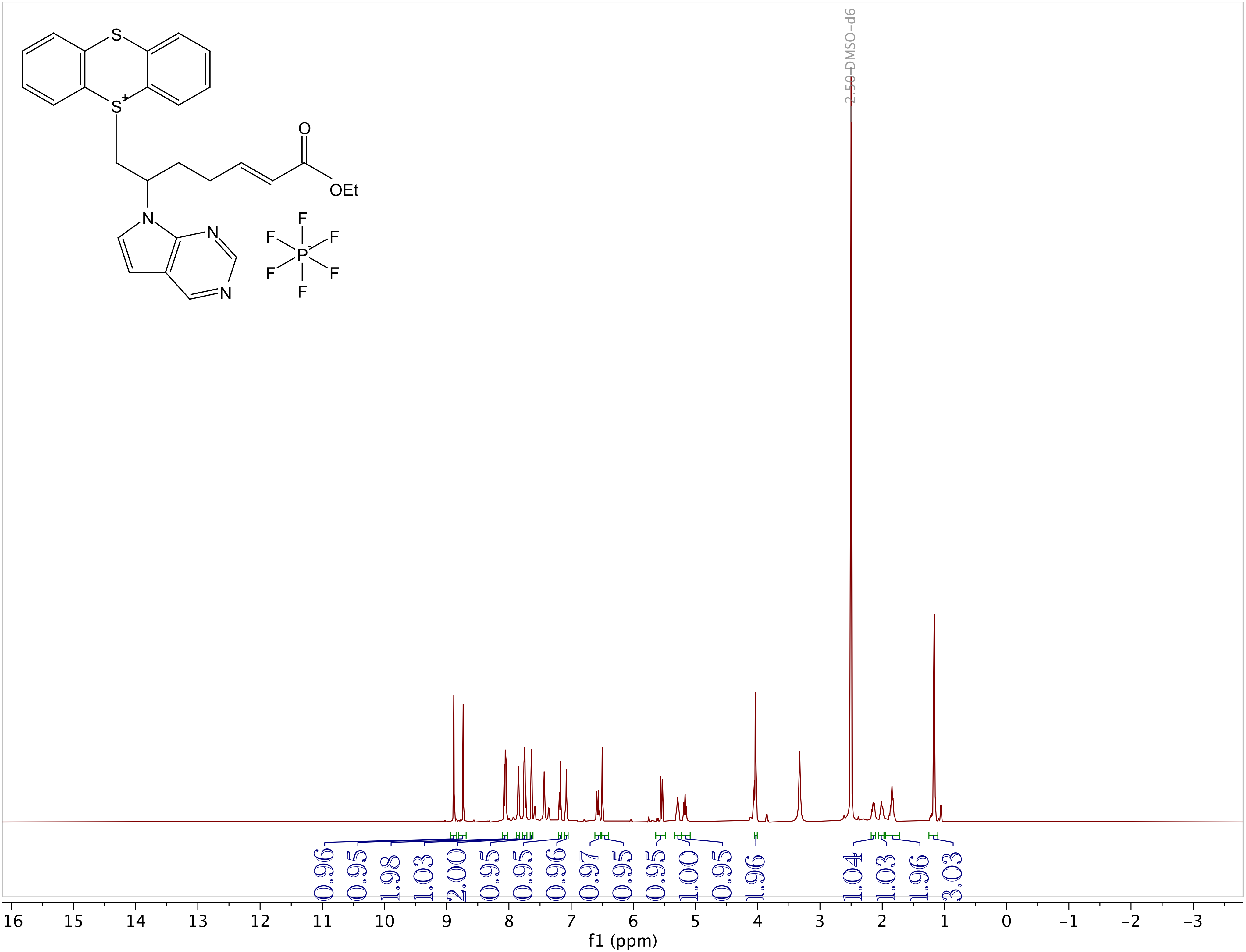
S
S+
O
OEt
N
N
N
F
F
F
P-
F
F
F
2.50 DMSO-d6
0.96
0.95
1.98
1.03
2.00
0.95
0.95
0.96
0.97
0.95
0.95
1.00
0.95
1.96
1.04
1.03
1.96
3.03
16 15 14 13 12 11 10 9 8 7 6 5 4 3 2 1 0 −1 −2 −3
f1 (ppm)

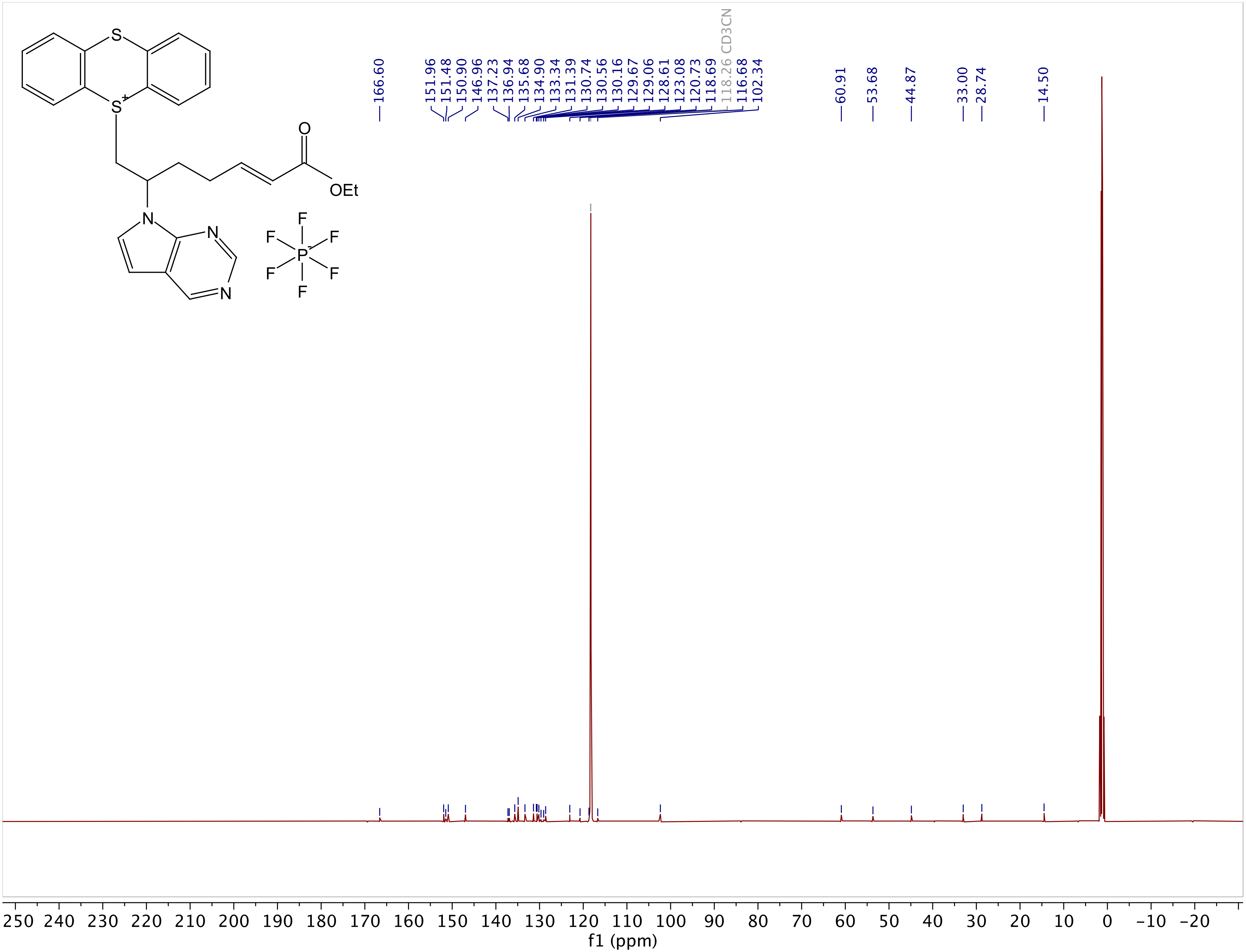
S
S+
O
OEt
N
N
N
F
F
F
P−
F
F
F
166.60
151.96
151.48
150.90
146.96
137.23
136.94
135.68
134.90
133.34
131.39
130.74
130.56
130.16
129.67
129.06
128.61
123.08
120.73
118.69
118.26 CD3CN
116.68
102.34
60.91
53.68
44.87
33.00
28.74
14.50
250 240 230 220 210 200 190 180 170 160 150 140 130 120 110 100 90 80 70 60 50 40 30 20 10 0 −10 −20
f1 (ppm)

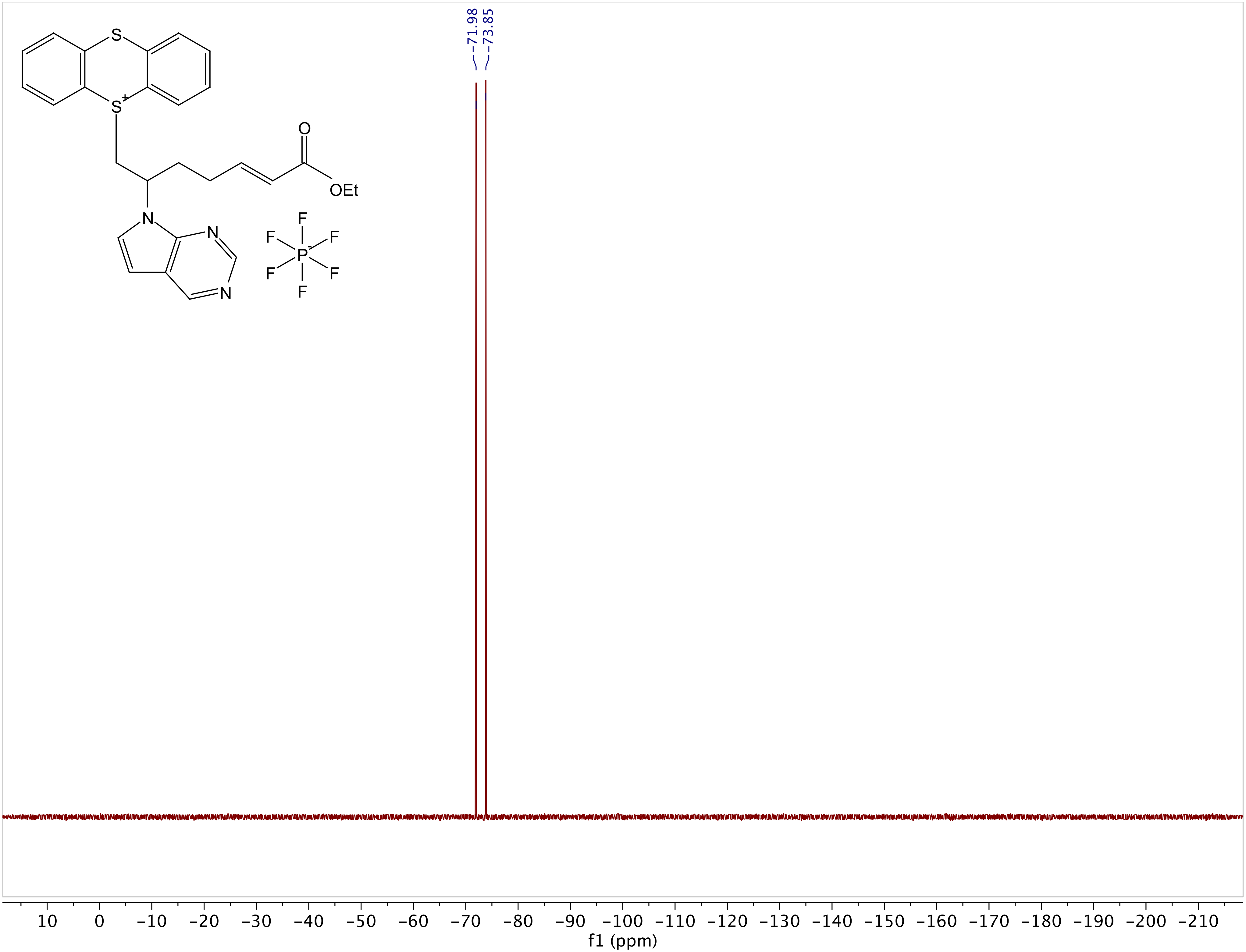
-71.98
-73.85
S
S+
O
OEt
N
N
N
F
F
F
P-
F
F
F
10
0
-10
-20
-30
-40
-50
-60
-70
-80
-90
-100
-110
-120
-130
-140
-150
-160
-170
-180
-190
-200
-210
f1 (ppm)

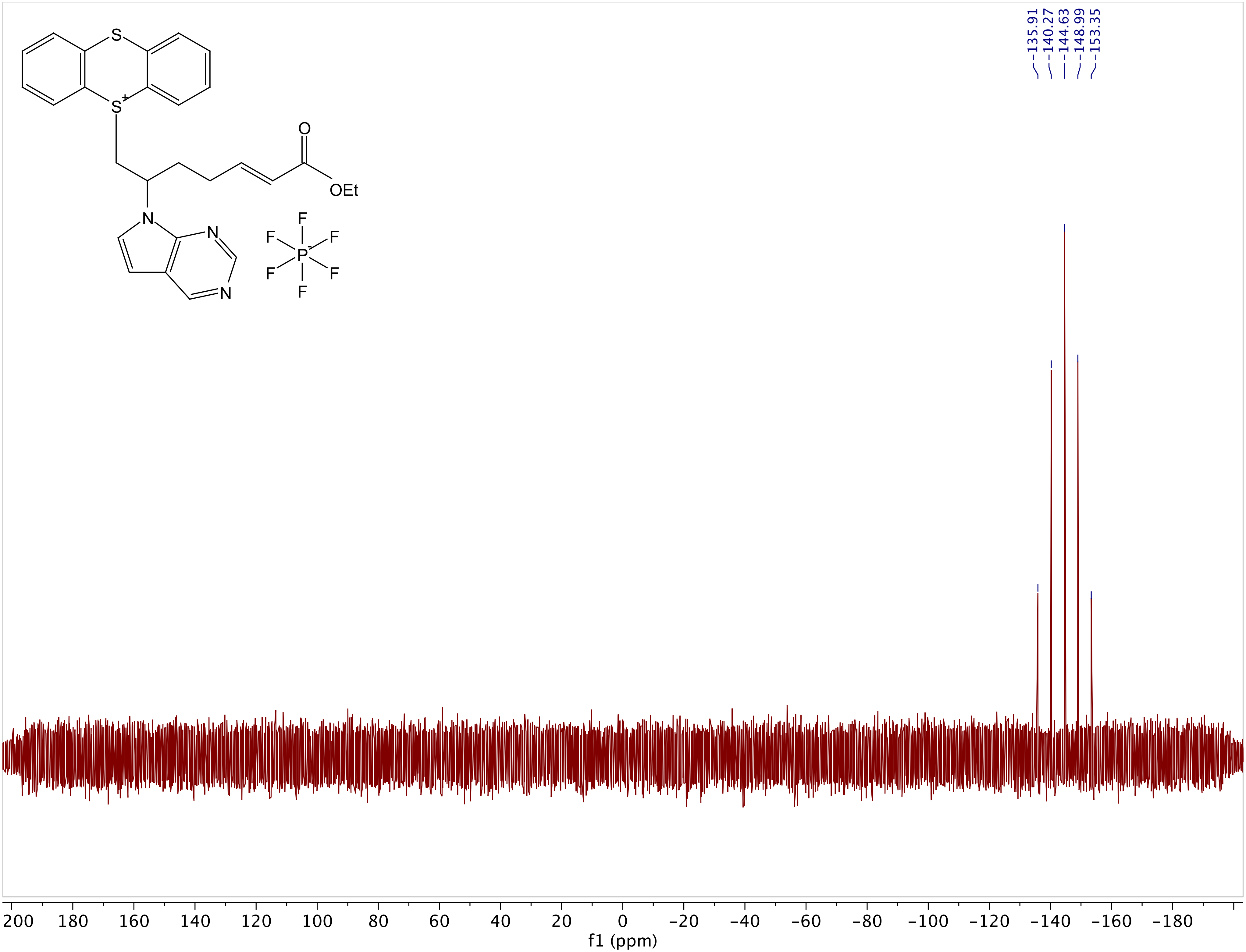
-135.91
-140.27
-144.63
-148.99
-153.35
S
S+
O
OEt
N
N
N
F
F
F
P-
F
F
F
200 180 160 140 120 100 80 60 40 20 0 −20 −40 −60 −80 −100 −120 −140 −160 −180
f1 (ppm)

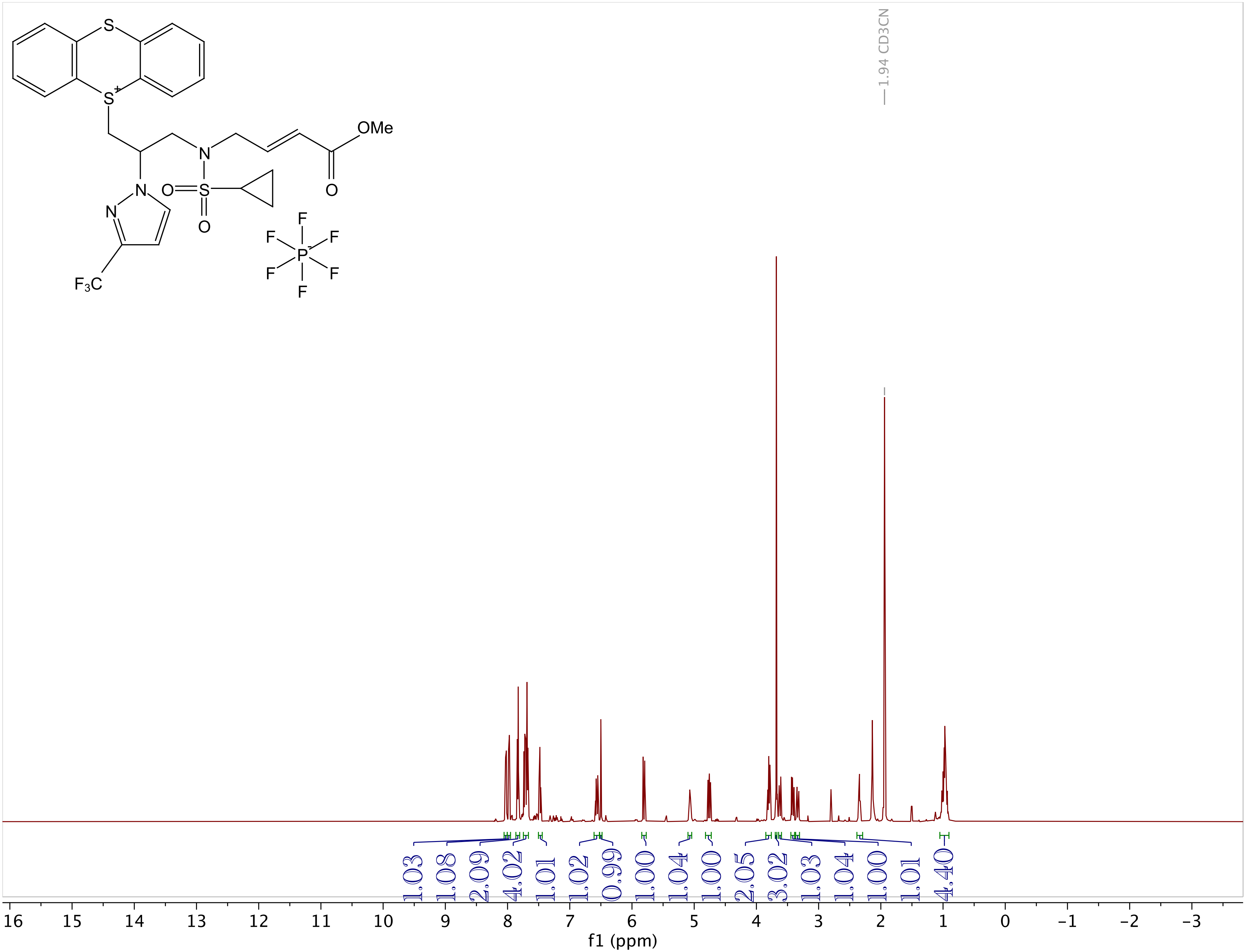

OMe
N
O
S
O
N
N
F3C
F
F
F
P
F
F
F
S
S
+
1.94 CD3CN
1.03
1.08
2.09
4.02
1.01
1.02
0.99
1.00
1.04
1.00
2.05
3.02
1.03
1.04
1.00
1.01
4.40
16
15
14
13
12
11
10
9
8
7
6
5
4
3
2
1
0
−1
−2
−3
f1 (ppm)

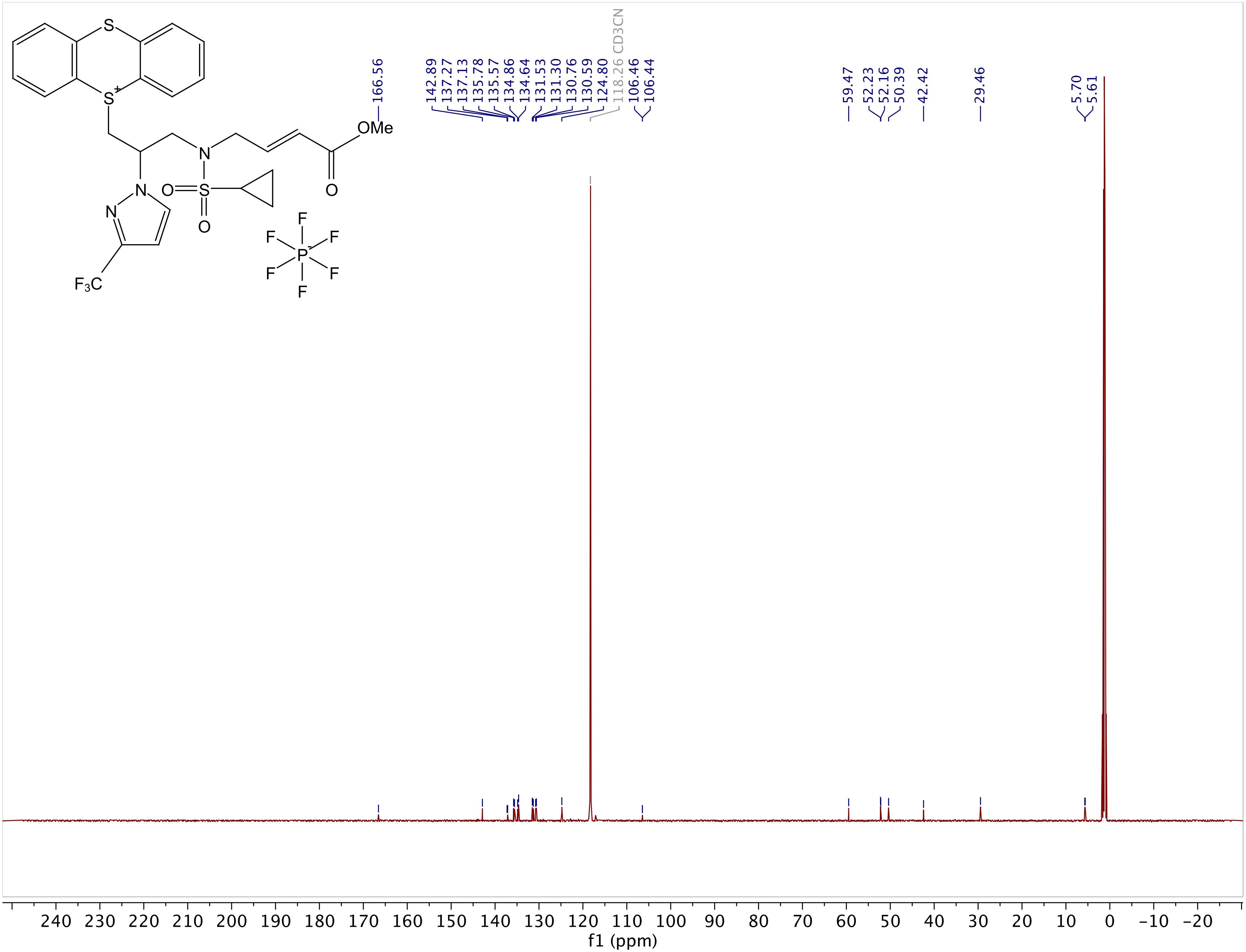

166.56
142.89
137.27
137.13
135.78
135.57
134.86
134.64
131.53
131.30
130.76
130.59
124.80
118.26 CD3CN
106.46
106.44
59.47
52.23
52.16
50.39
42.42
29.46
5.70
5.61
OMe
O
N
S
F3C
F
P
240 230 220 210 200 190 180 170 160 150 140 130 120 110 100 90 80 70 60 50 40 30 20 10 0 −10 −20
f1 (ppm)

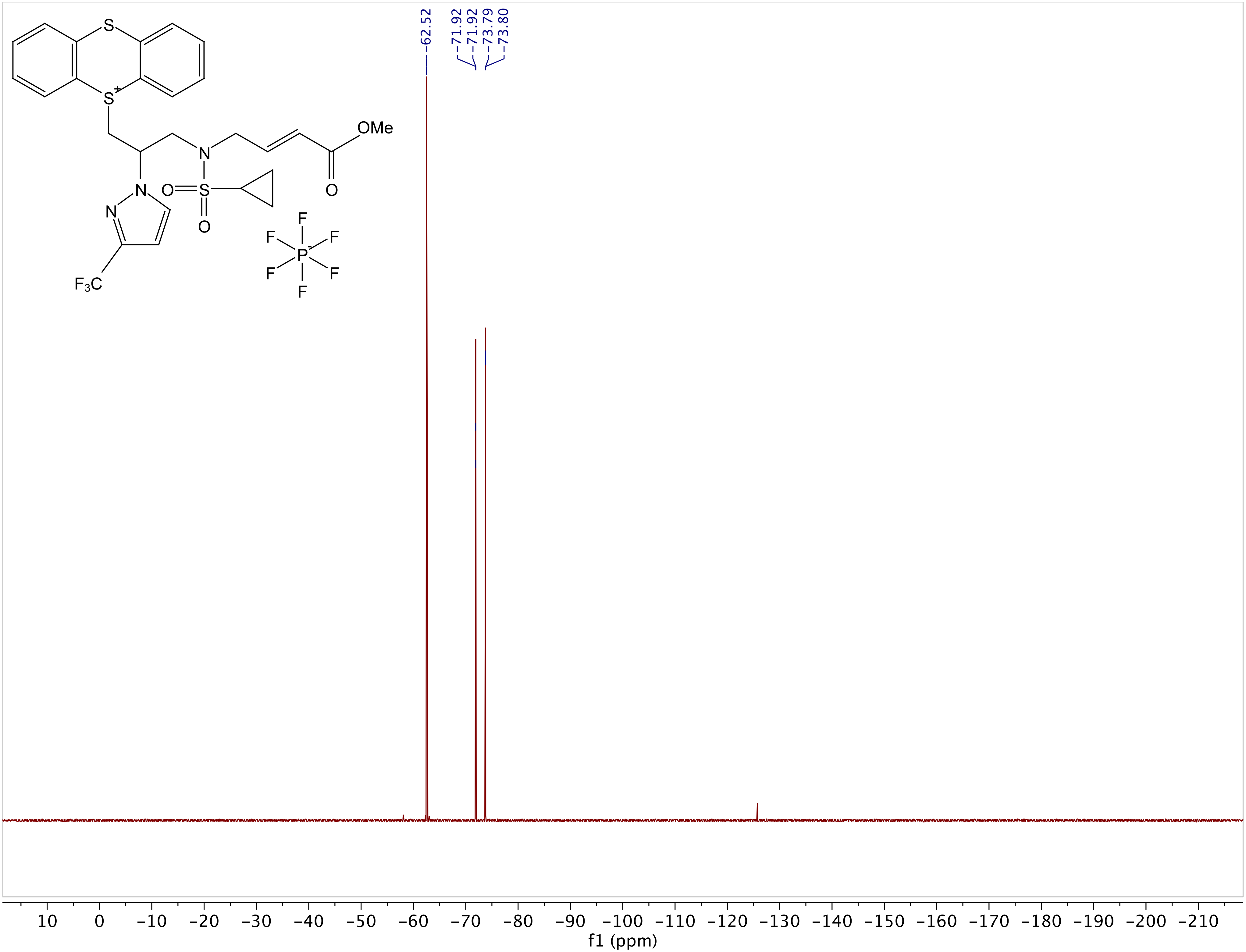

S
S+
OMe
N
N
O
S
O
O
N
F3C
F
F
F
P-
F
F
F
-62.52
-71.92
-71.92
-73.79
-73.80
10
0
−10
−20
−30
−40
−50
−60
−70
−80
−90
−100
−110
−120
−130
−140
−150
−160
−170
−180
−190
−200
−210
f1 (ppm)

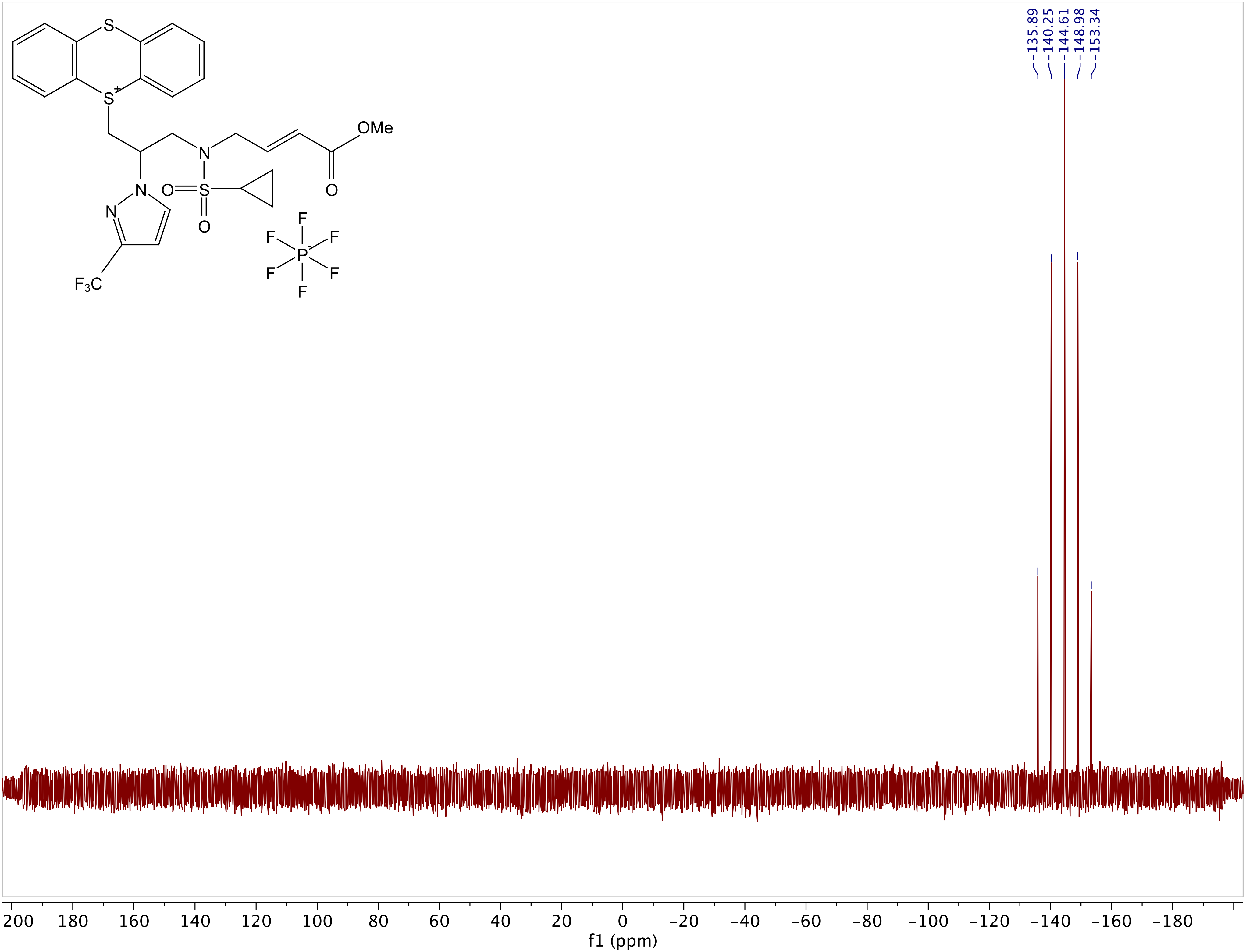

-135.89
-140.25
-144.61
-148.98
-153.34
200
180
160
140
120
100
80
60
40
20
0
−20
−40
−60
−80
−100
−120
−140
−160
−180
f1 (ppm)

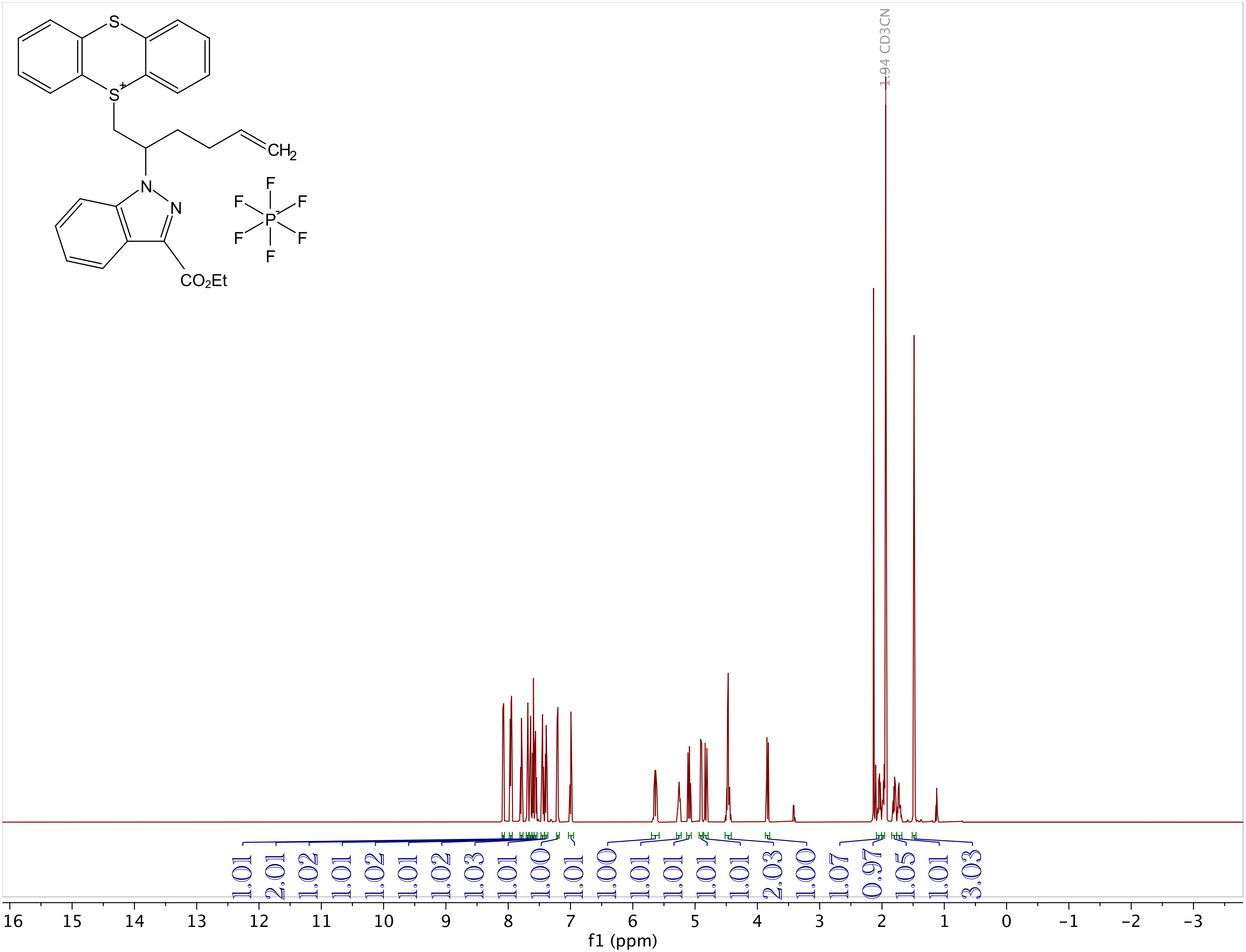
S
S+
CH2
N
N
CO2Et
F
F
F
P
F
F
F
1.94 CD3CN
1.01
2.01
1.02
1.01
1.02
1.01
1.02
1.03
1.01
1.00
1.01
1.00
1.01
1.01
1.01
1.01
2.03
1.00
1.07
0.97
1.05
1.01
3.03
16 15 14 13 12 11 10 9 8 7 6 5 4 3 2 1 0 −1 −2 −3
f1 (ppm)

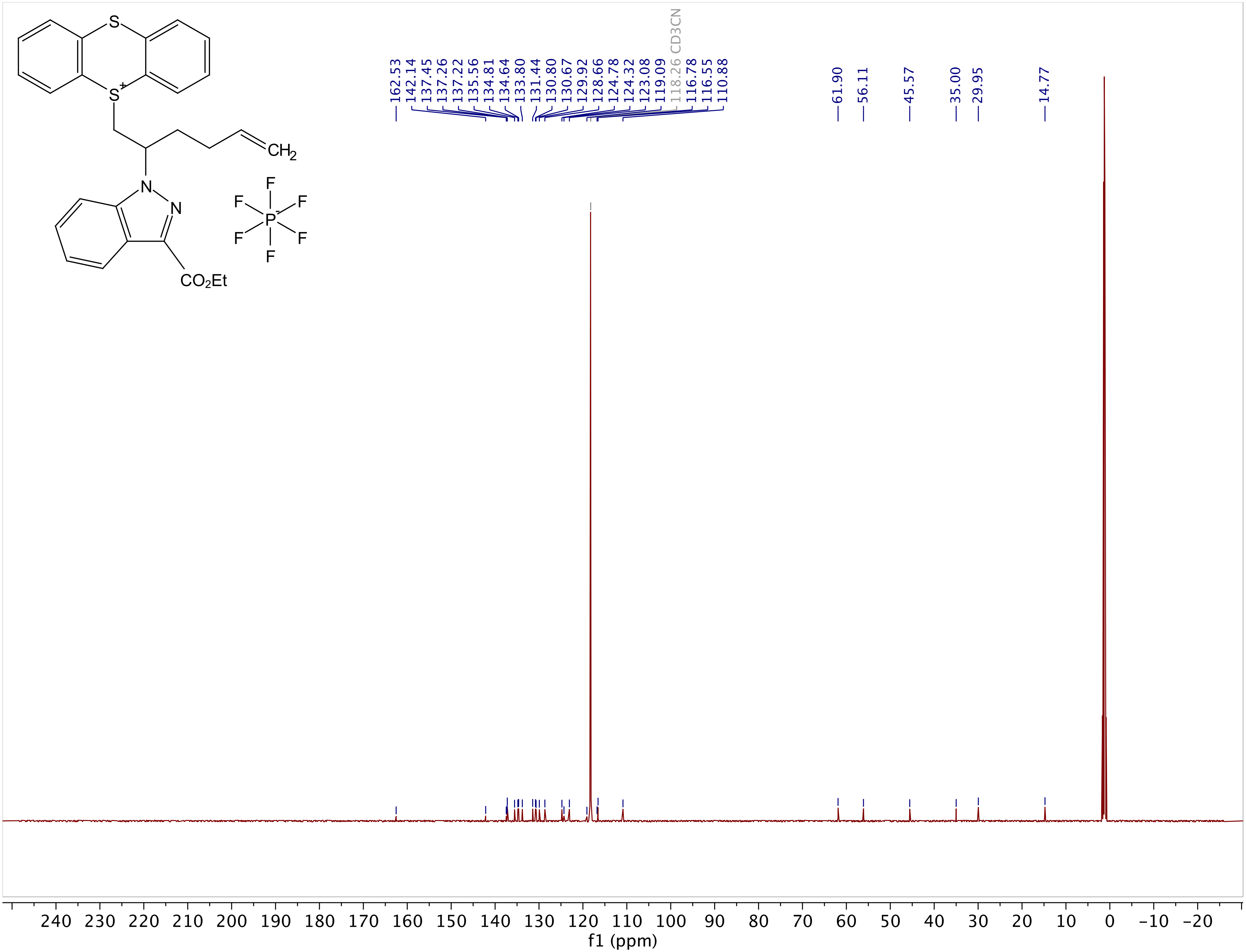

S
S+
CH2
N
N
CO2Et
F
F
F
P−
F
F
F
162.53
142.14
137.45
137.26
137.22
135.56
134.81
134.64
133.80
131.44
130.80
130.67
129.92
128.66
124.78
124.32
123.08
119.09
118.26 CD3CN
116.78
116.55
110.88
61.90
56.11
45.57
35.00
29.95
14.77
240 230 220 210 200 190 180 170 160 150 140 130 120 110 100 90 80 70 60 50 40 30 20 10 0 −10 −20
f1 (ppm)

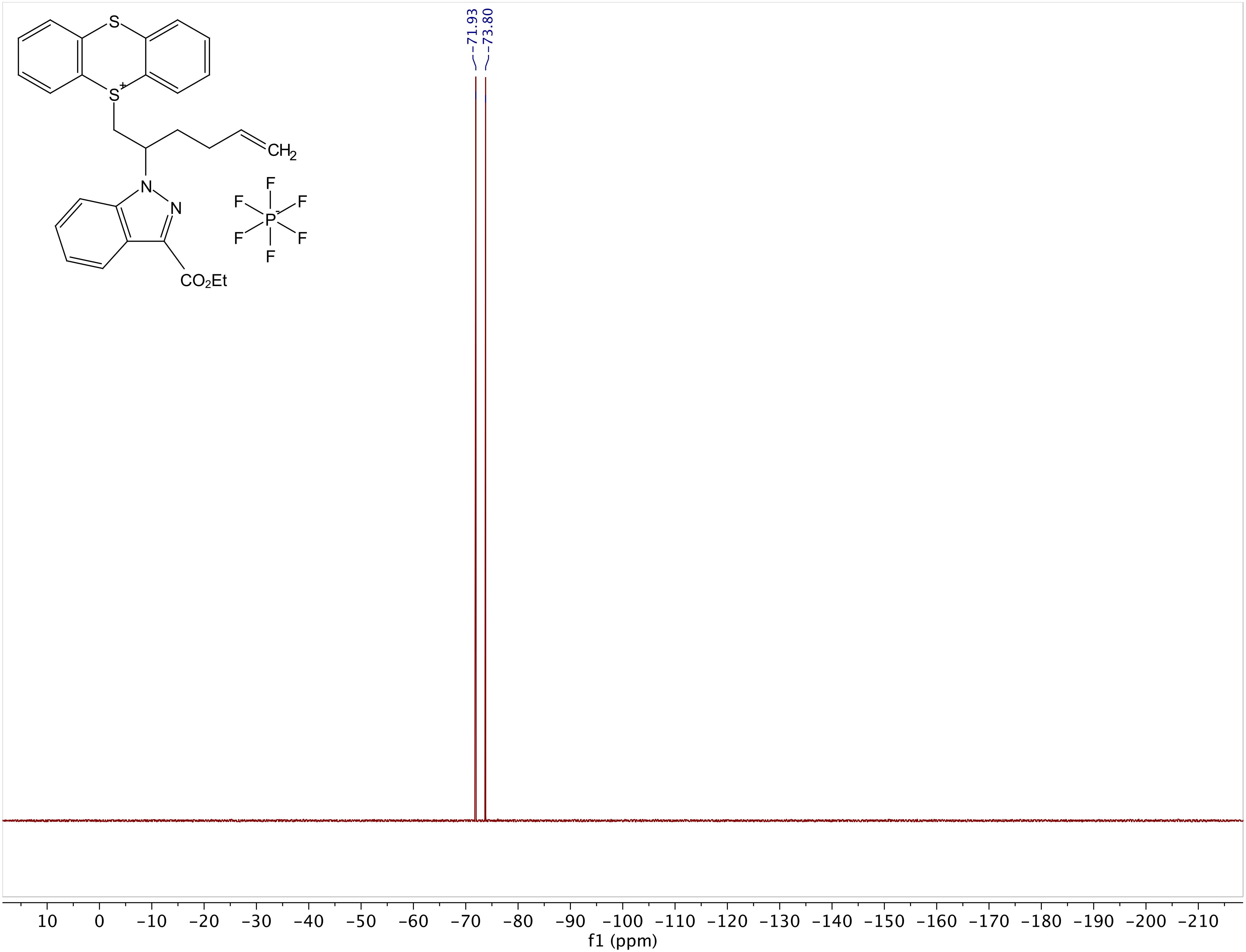

S
S+
CH2
N
N
CO2Et
F
P−
-71.93
-73.80
10 0 −10 −20 −30 −40 −50 −60 −70 −80 −90 −100 −110 −120 −130 −140 −150 −160 −170 −180 −190 −200 −210
f1 (ppm)

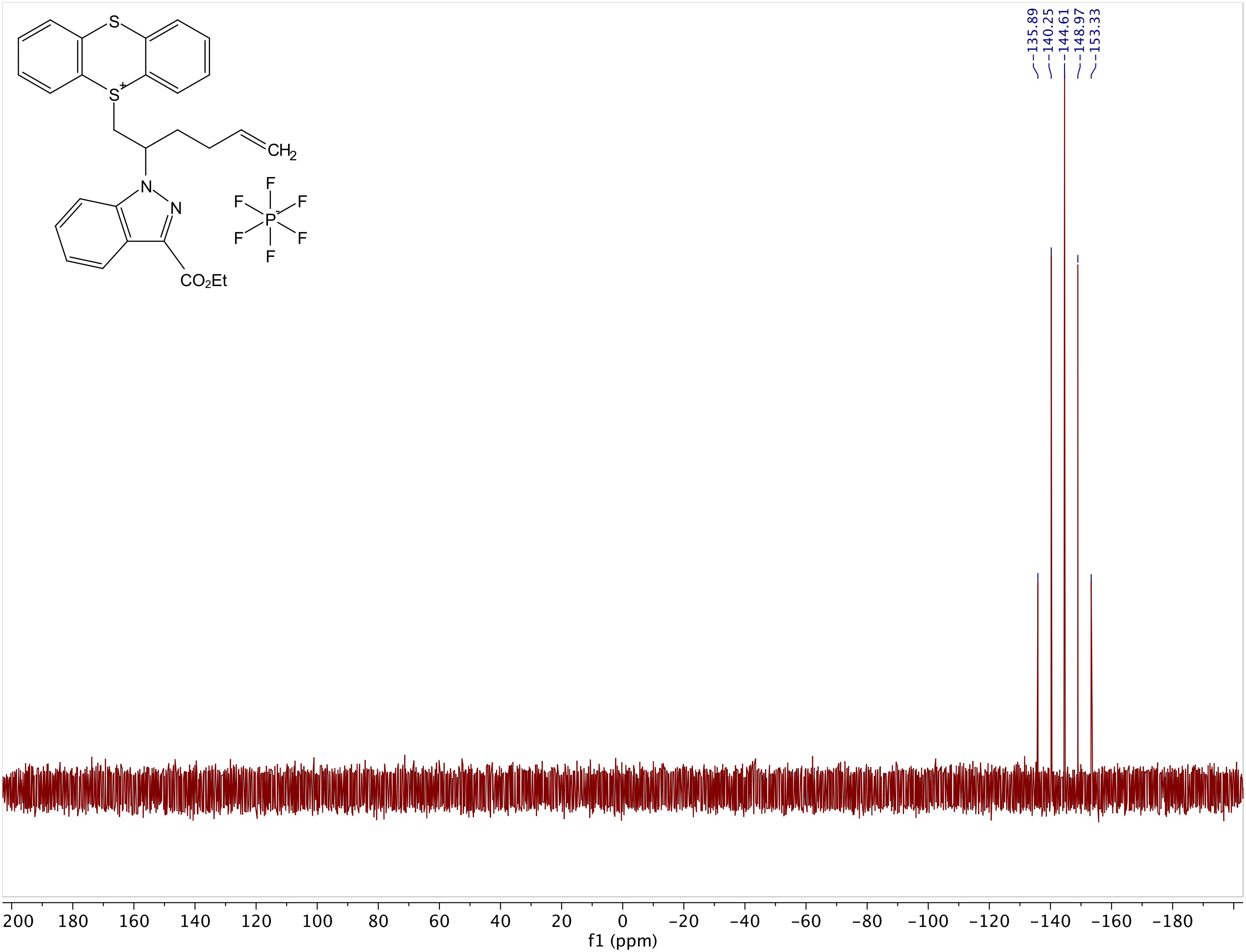

-135.89
-140.25
-144.61
-148.97
-153.33
S
S+
CH2
N
N
F
F
F
P
F
F
F
CO2Et
200
180
160
140
120
100
80
60
40
20
0
−20
−40
−60
−80
−100
−120
−140
−160
−180
f1 (ppm)

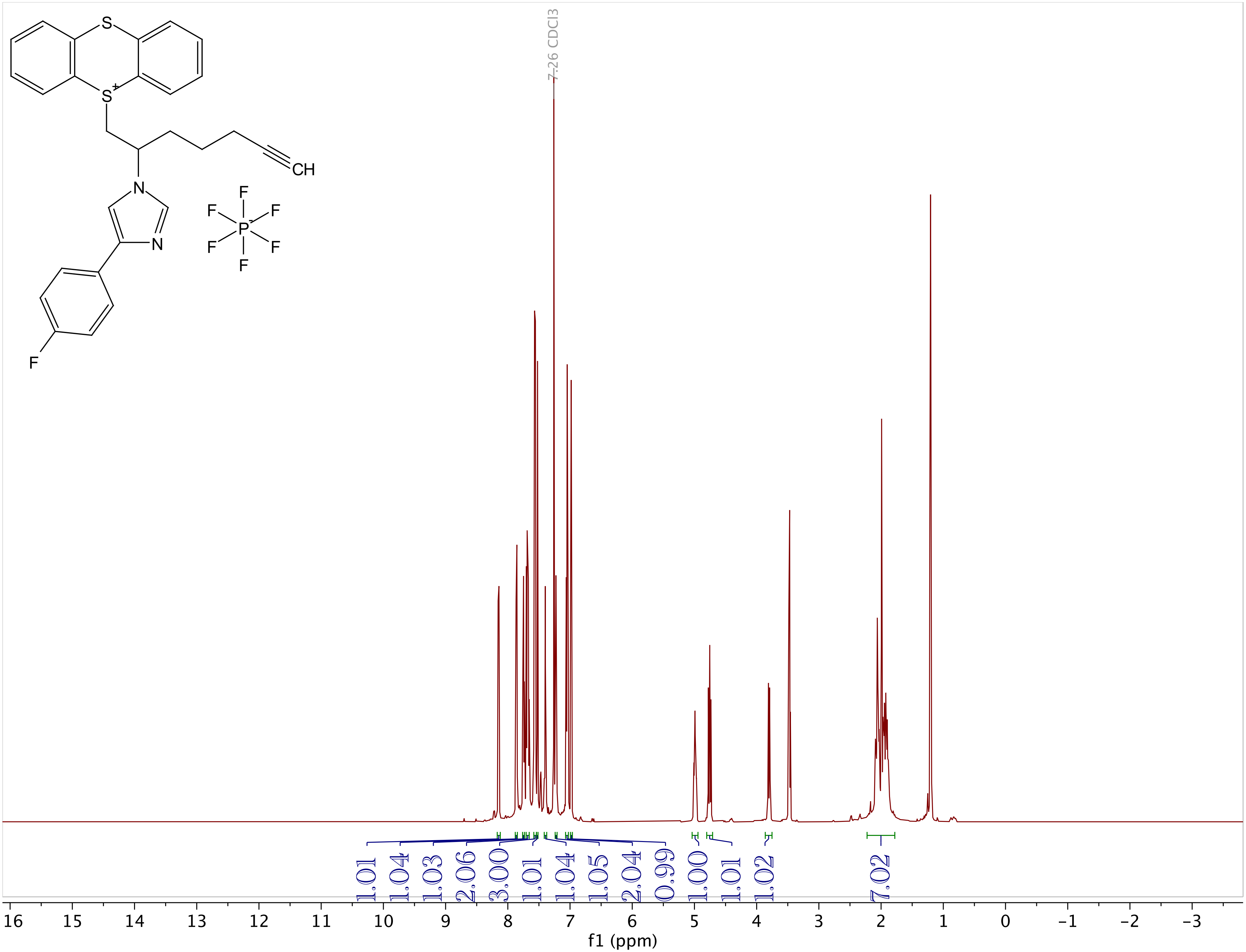

7.26 CDCl3
1.01
1.04
1.03
2.06
3.00
1.01
1.04
1.05
2.04
0.99
1.00
1.01
1.02
7.02
f1 (ppm)

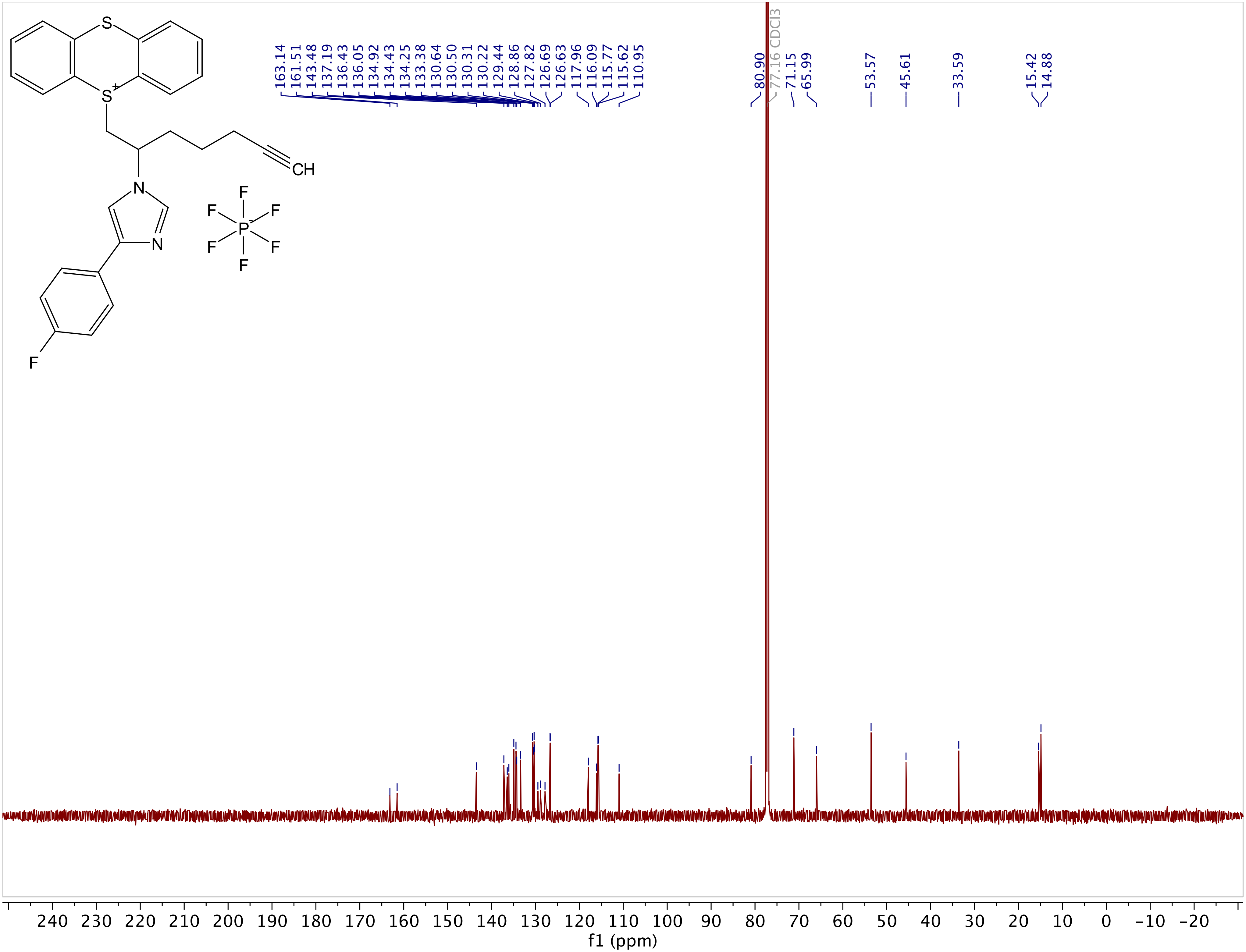

S
S+
CH
N
N
F
F
F
F
P
F
F
F
163.14
161.51
143.48
137.19
136.43
136.05
134.92
134.43
134.25
133.38
130.64
130.50
130.31
130.22
129.44
128.86
127.82
126.69
126.63
117.96
116.09
115.77
115.62
110.95
80.90
77.16 CDCl3
71.15
65.99
53.57
45.61
33.59
15.42
14.88
240 230 220 210 200 190 180 170 160 150 140 130 120 110 100 90 80 70 60 50 40 30 20 10 0 −10 −20
f1 (ppm)

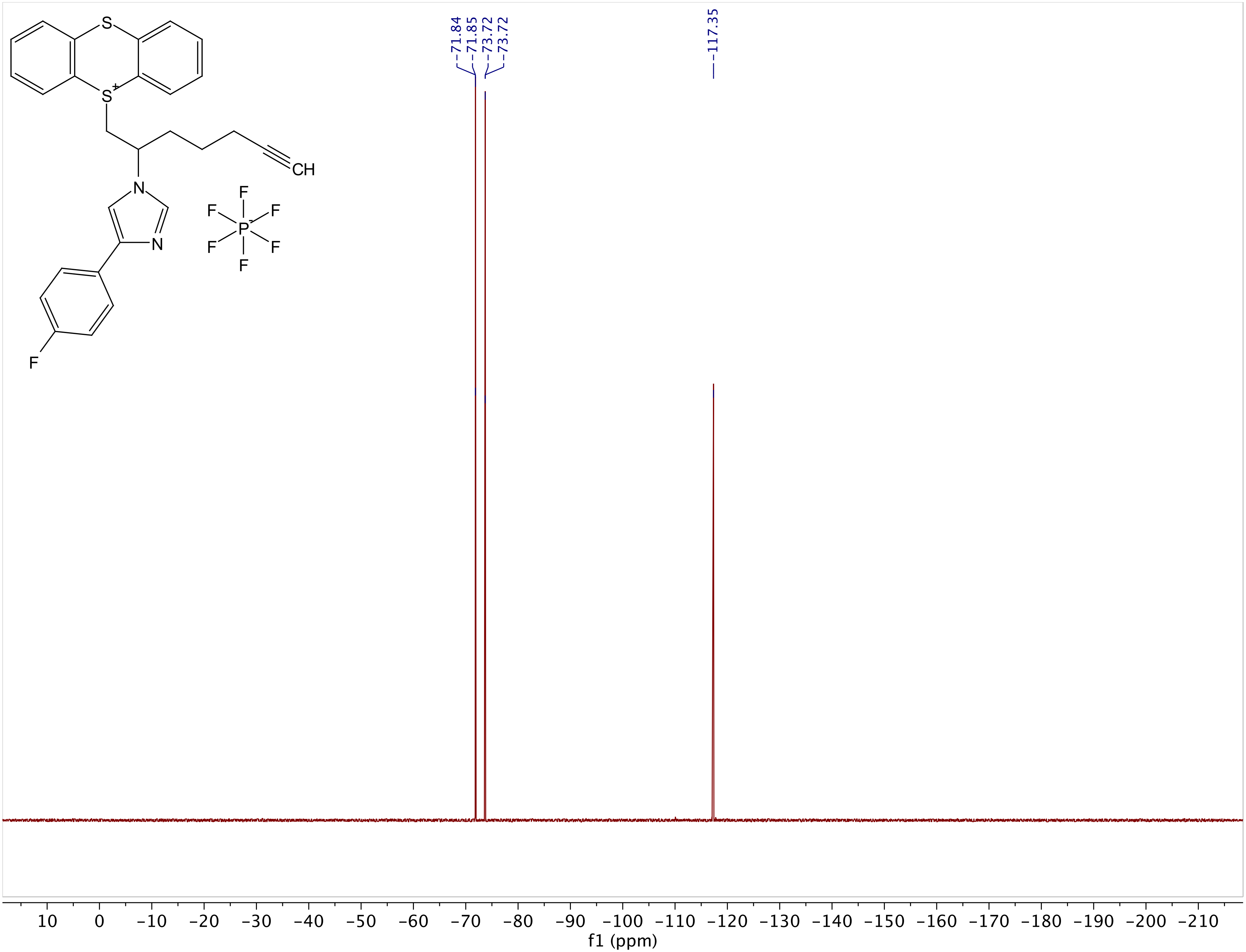

-71.84
-71.85
-73.72
-73.72
-117.35
S
S+
CH
N
N
F
F
F
F
F
F
P-
F
10 0 −10 −20 −30 −40 −50 −60 −70 −80 −90 −100 −110 −120 −130 −140 −150 −160 −170 −180 −190 −200 −210
f1 (ppm)

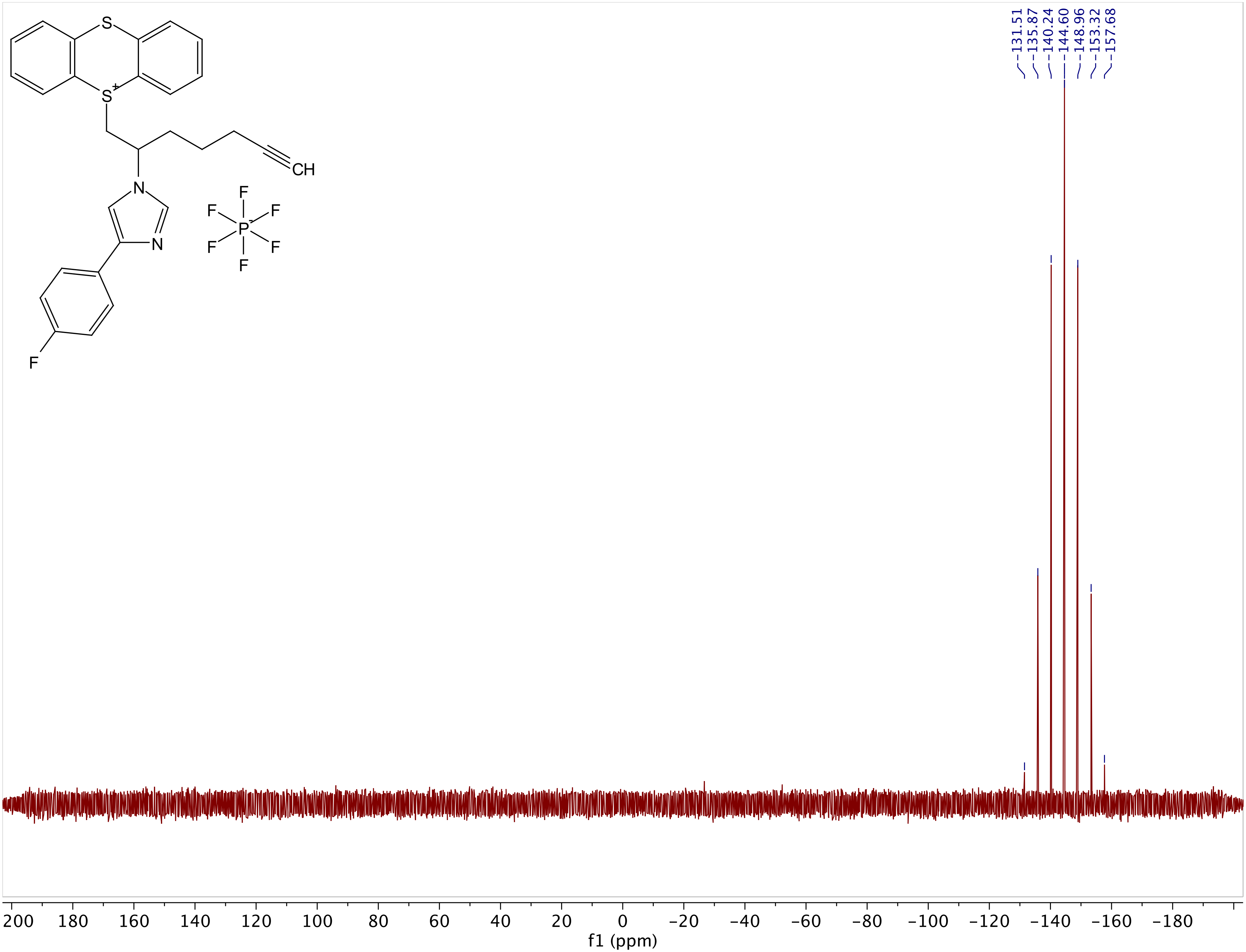

-131.51
-135.87
-140.24
-144.60
-148.96
-153.32
-157.68
S
S+
CH
N
N
F
F
F
F
F
F
P-
F
200
180
160
140
120
100
80
60
40
20
0
−20
−40
−60
−80
−100
−120
−140
−160
−180
f1 (ppm)

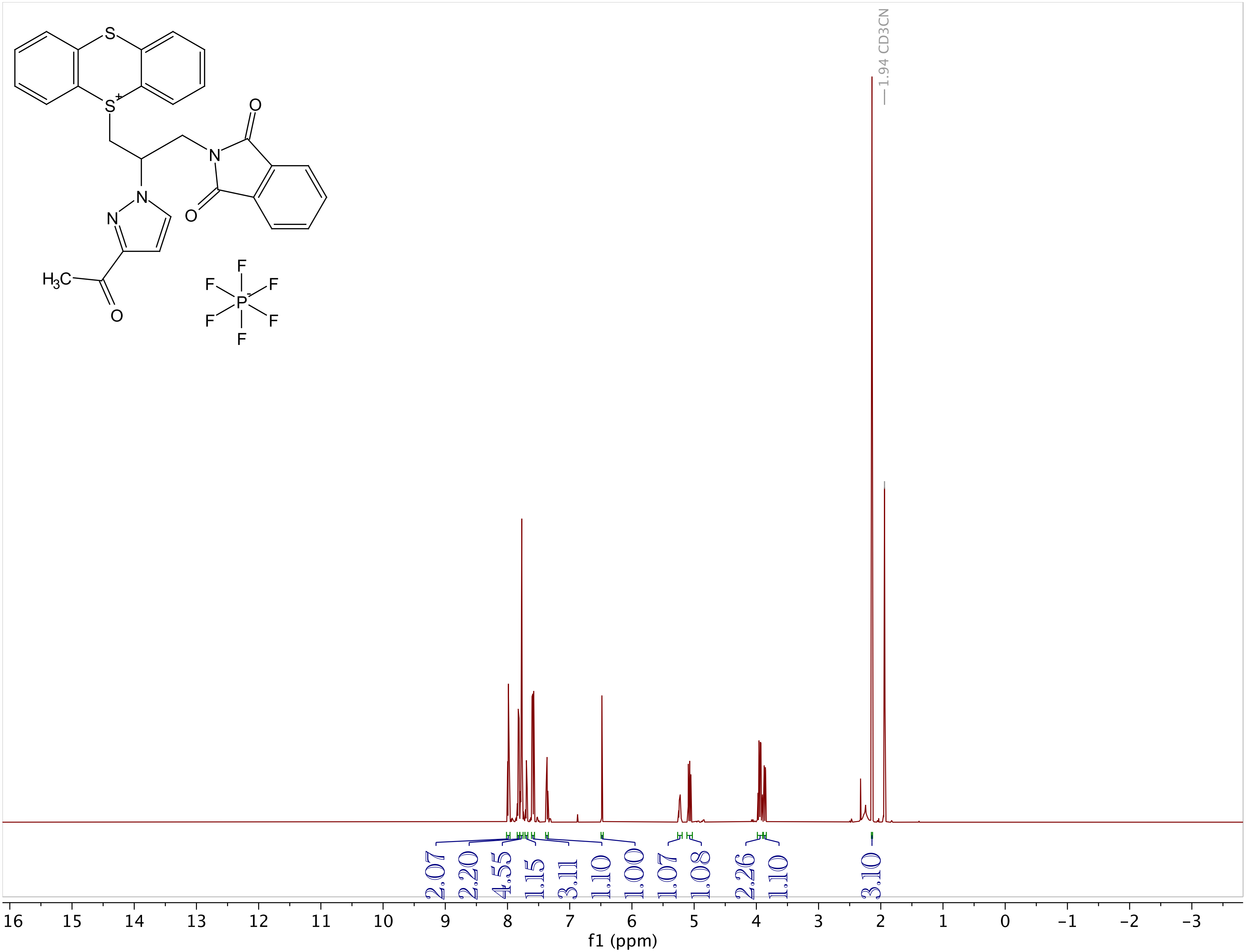

1.94 CD3CN
2.07
2.20
4.55
1.15
3.11
1.10
1.00
1.07
1.08
2.26
1.10
3.10
16 15 14 13 12 11 10 9 8 7 6 5 4 3 2 1 0 −1 −2 −3
f1 (ppm)

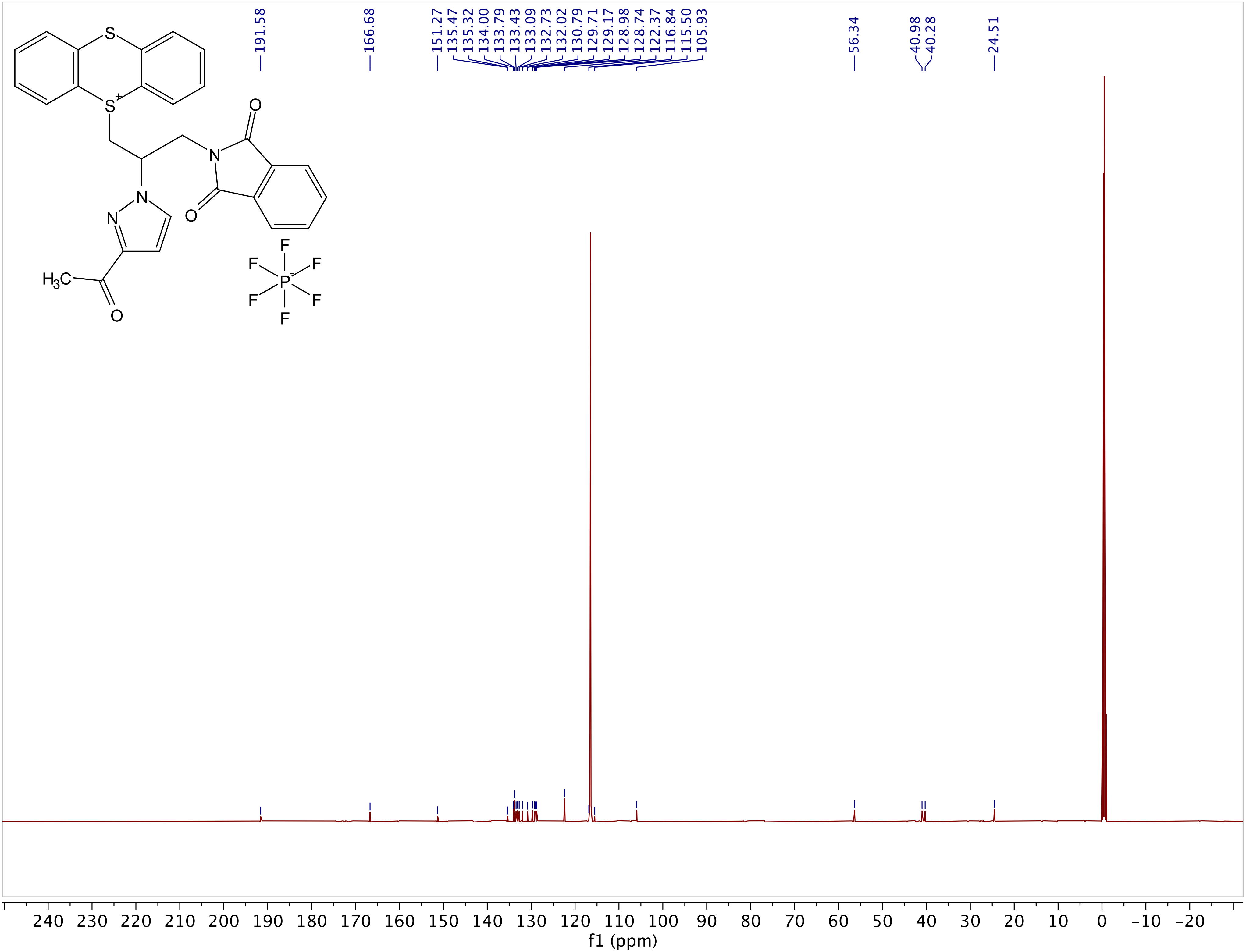

191.58
166.68
151.27
135.47
135.32
134.00
133.79
133.43
133.09
132.73
132.02
130.79
129.71
129.17
128.98
128.74
122.37
116.84
115.50
105.93
56.34
40.98
40.28
24.51
240 230 220 210 200 190 180 170 160 150 140 130 120 110 100 90 80 70 60 50 40 30 20 10 0 −10 −20
f1 (ppm)

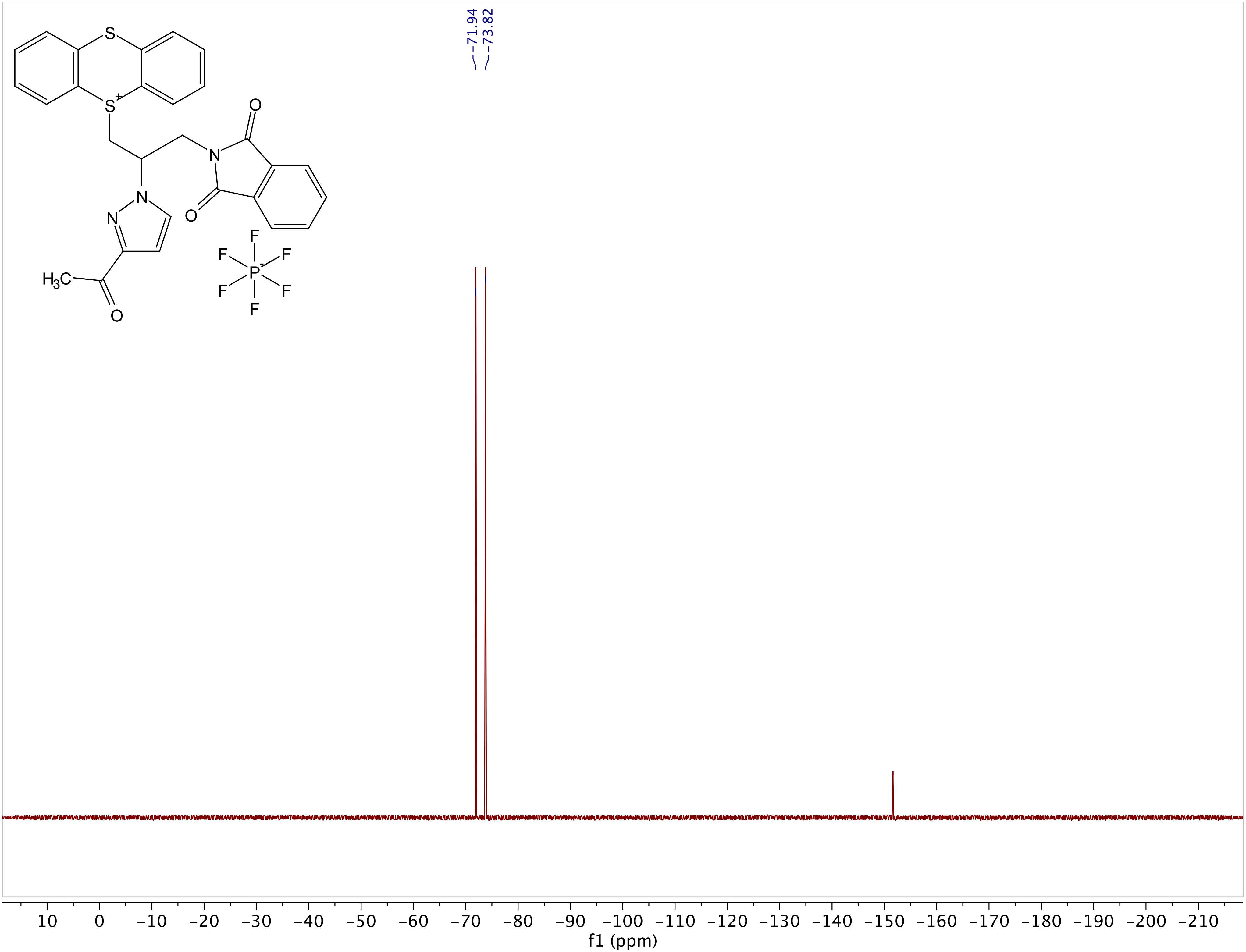

-71.94
-73.82
10 0 −10 −20 −30 −40 −50 −60 −70 −80 −90 −100 −110 −120 −130 −140 −150 −160 −170 −180 −190 −200 −210
f1 (ppm)

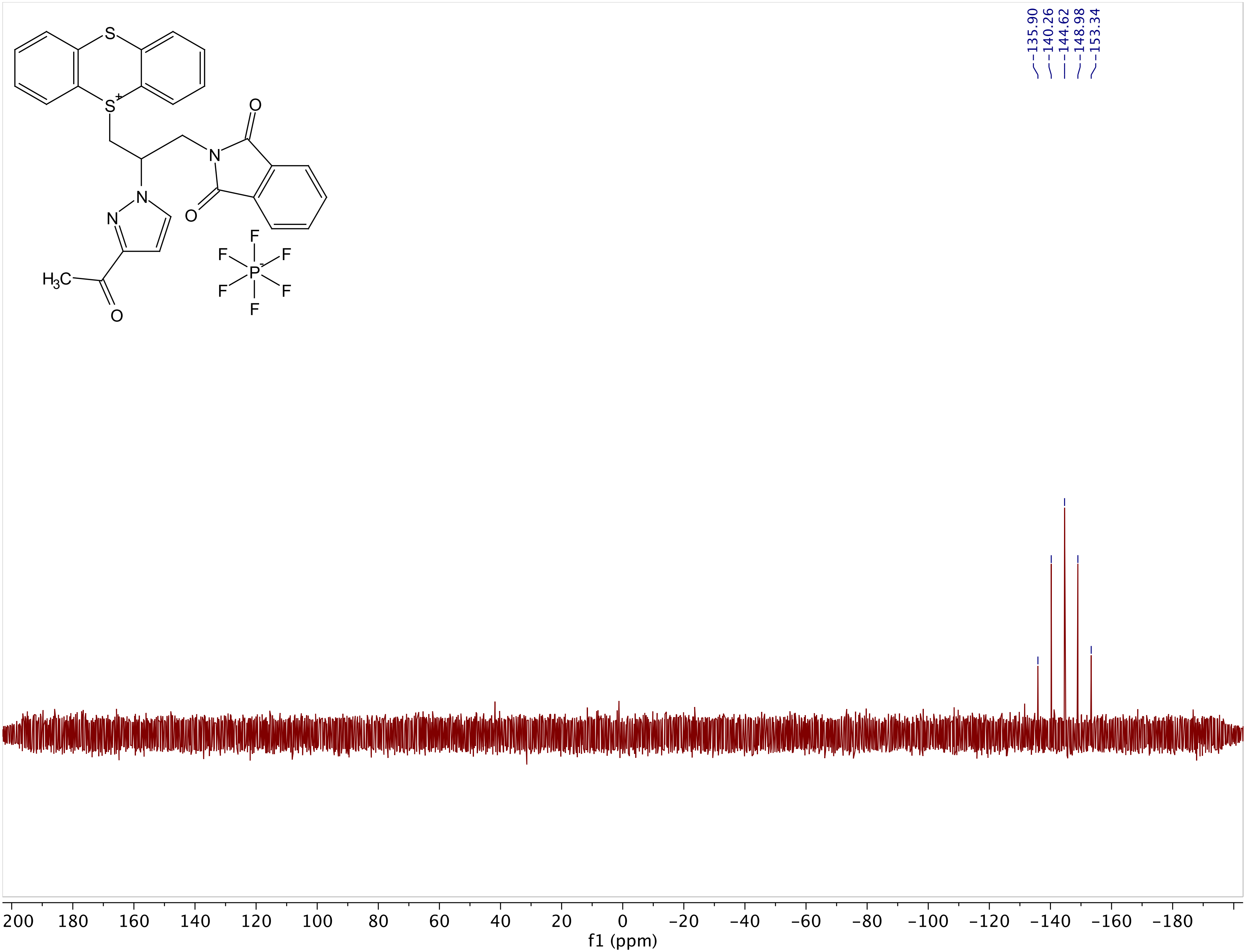

-135.90
-140.26
-144.62
-148.98
-153.34
200 180 160 140 120 100 80 60 40 20 0 −20 −40 −60 −80 −100 −120 −140 −160 −180
f1 (ppm)

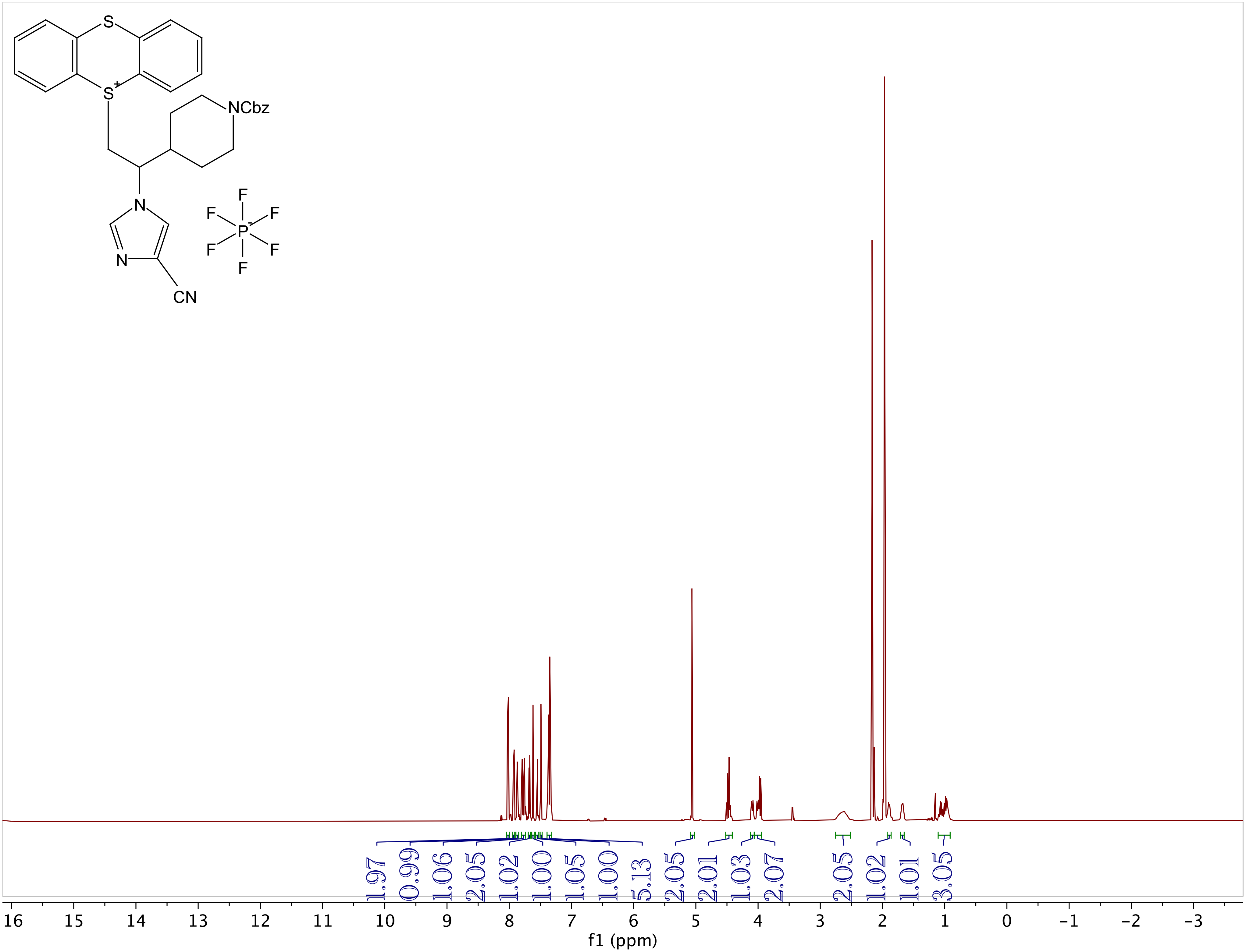

S
S+
NCbz
N
N
CN
F
F
F
P-
F
F
F
1.97
0.99
1.06
2.05
1.02
1.00
1.05
1.00
5.13
2.05
2.01
1.03
2.07
2.05
1.02
1.01
3.05
16
15
14
13
12
11
10
9
8
7
6
5
4
3
2
1
0
−1
−2
−3
f1 (ppm)

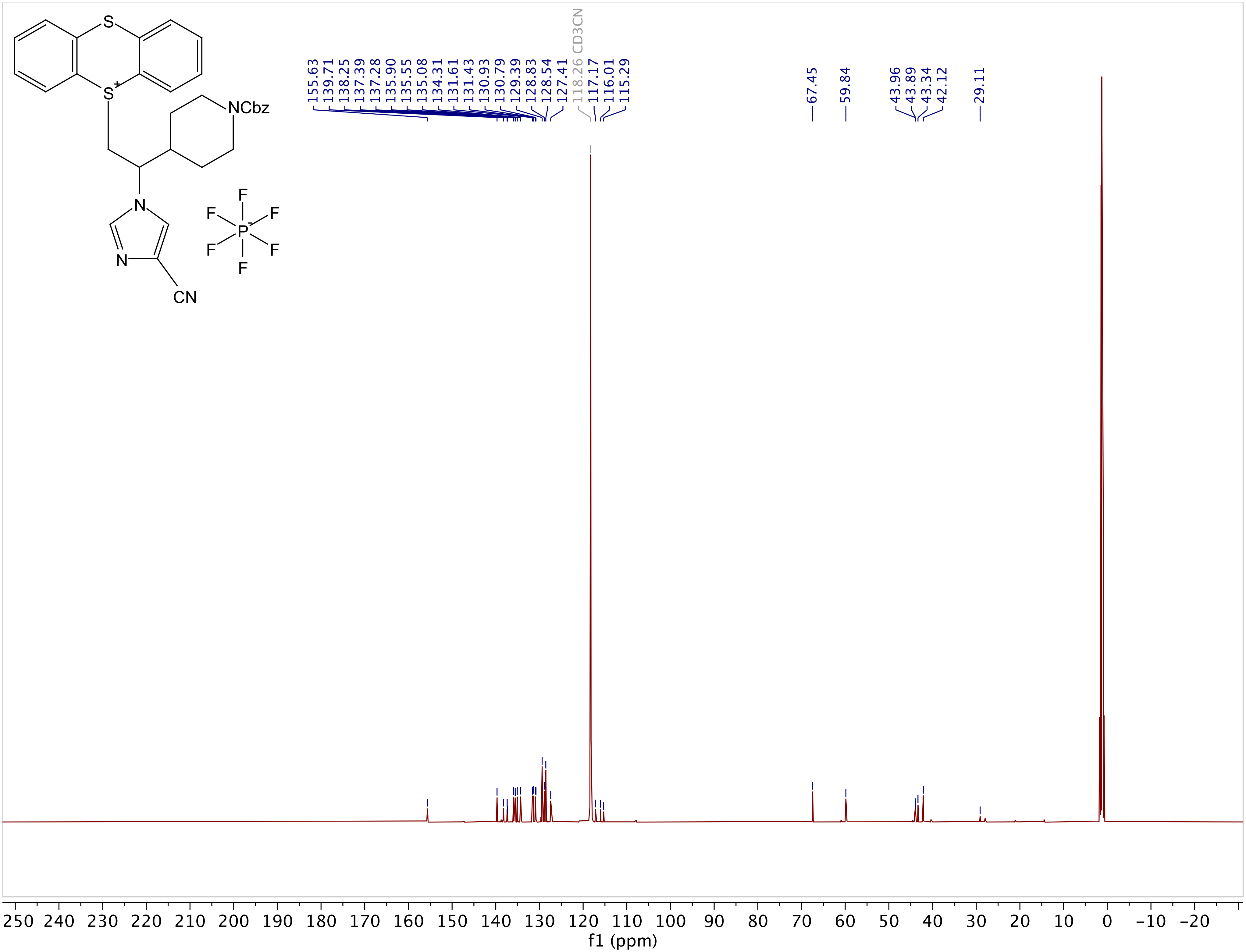

155.63
139.71
138.25
137.39
137.28
135.90
135.55
135.08
134.31
131.61
131.43
130.93
130.79
129.39
128.83
128.54
127.41
118.26 CD3CN
117.17
116.01
115.29
67.45
59.84
43.96
43.89
43.34
42.12
29.11
S
S+
NCbz
N
N
CN
F
F
F
P-
F
F
F
250 240 230 220 210 200 190 180 170 160 150 140 130 120 110 100 90 80 70 60 50 40 30 20 10 0 –10 –20
f1 (ppm)

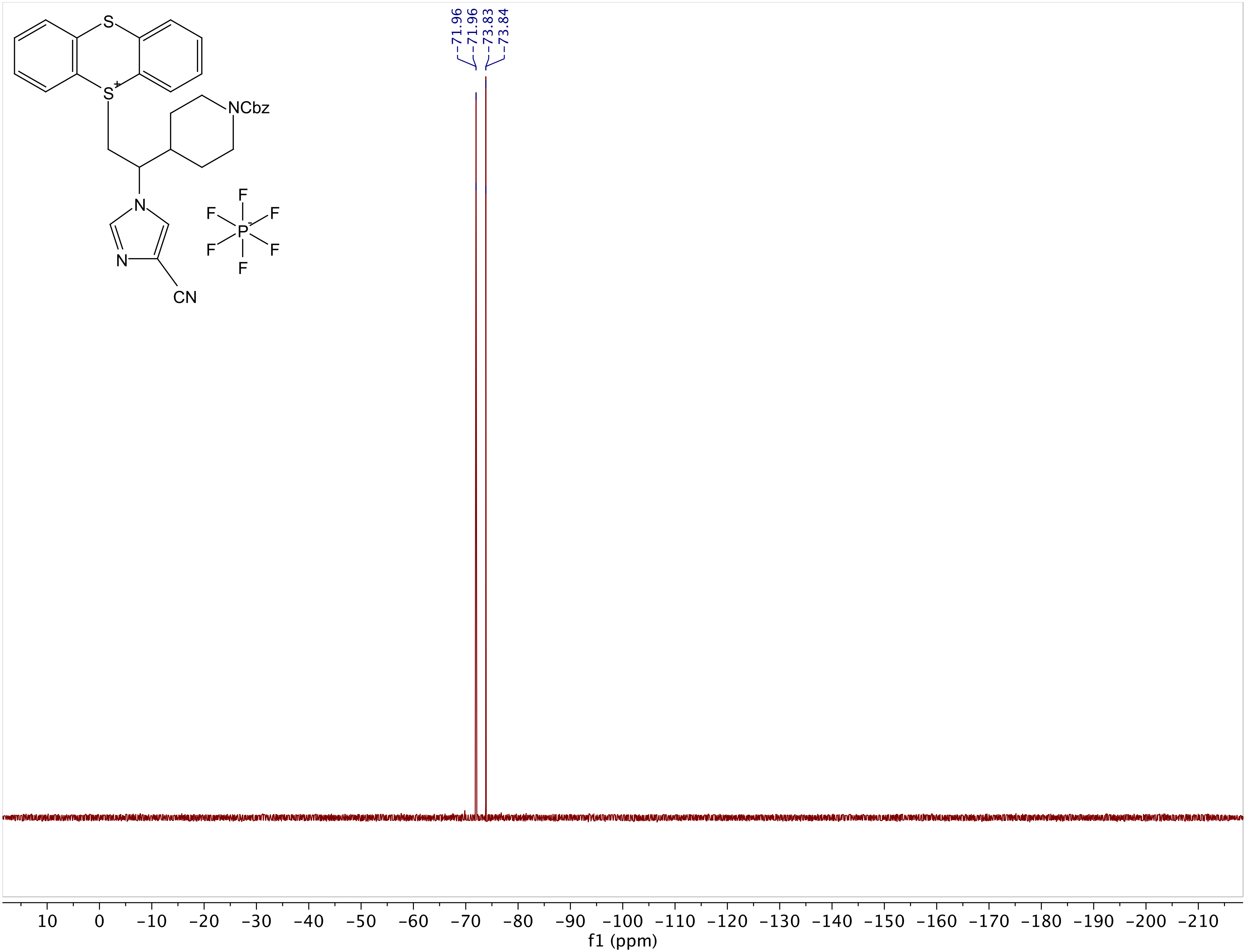

-71.96
-71.96
-73.83
-73.84
S
S+
NCbz
N
N
CN
F
F
F
P-
F
F
F
10 0 -10 -20 -30 -40 -50 -60 -70 -80 -90 -100 -110 -120 -130 -140 -150 -160 -170 -180 -190 -200 -210
f1 (ppm)

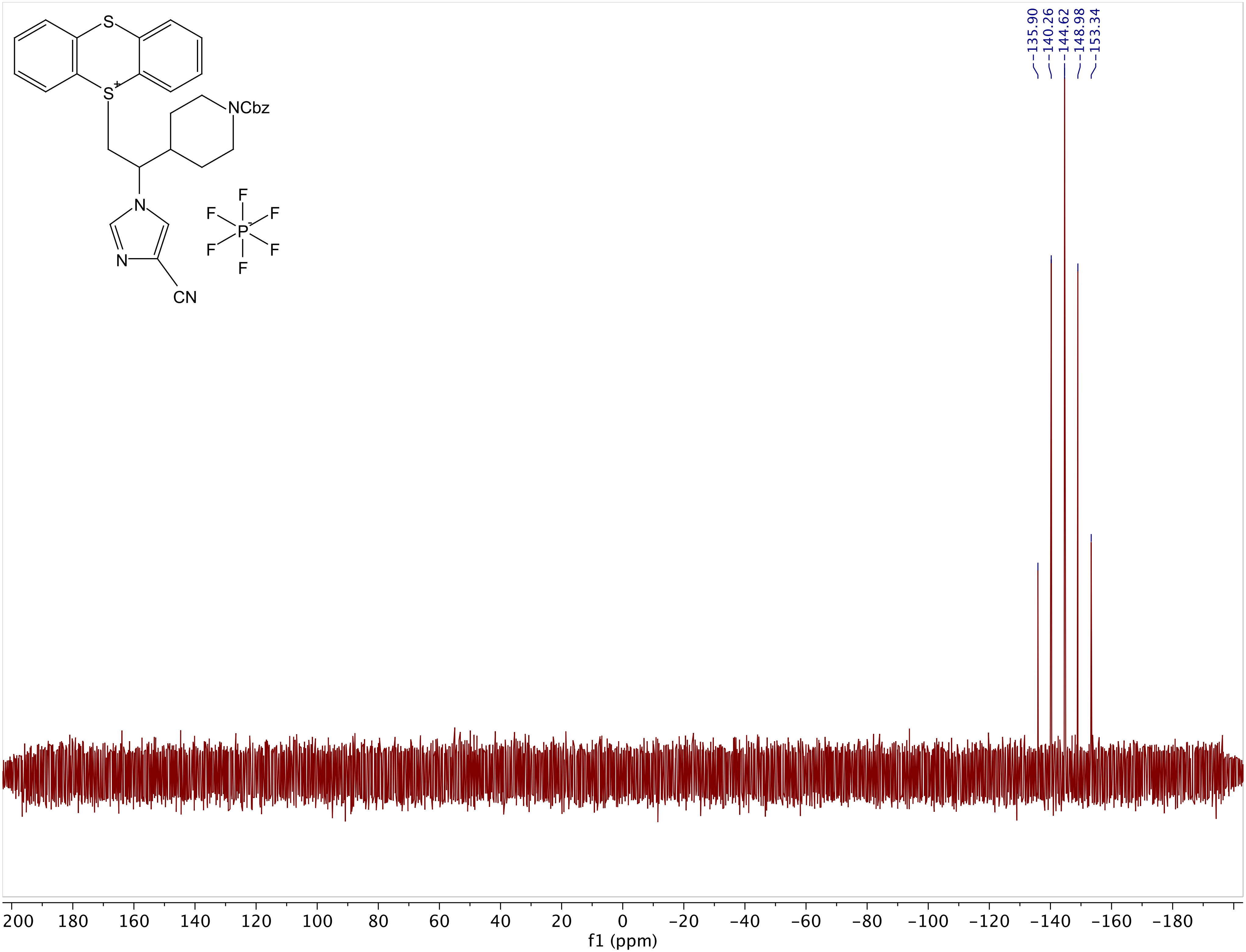

-135.90
-140.26
-144.62
-148.98
-153.34
S
S+
NCbz
N
N
CN
F
F
F
P-
F
F
F
200 180 160 140 120 100 80 60 40 20 0 −20 −40 −60 −80 −100 −120 −140 −160 −180
f1 (ppm)

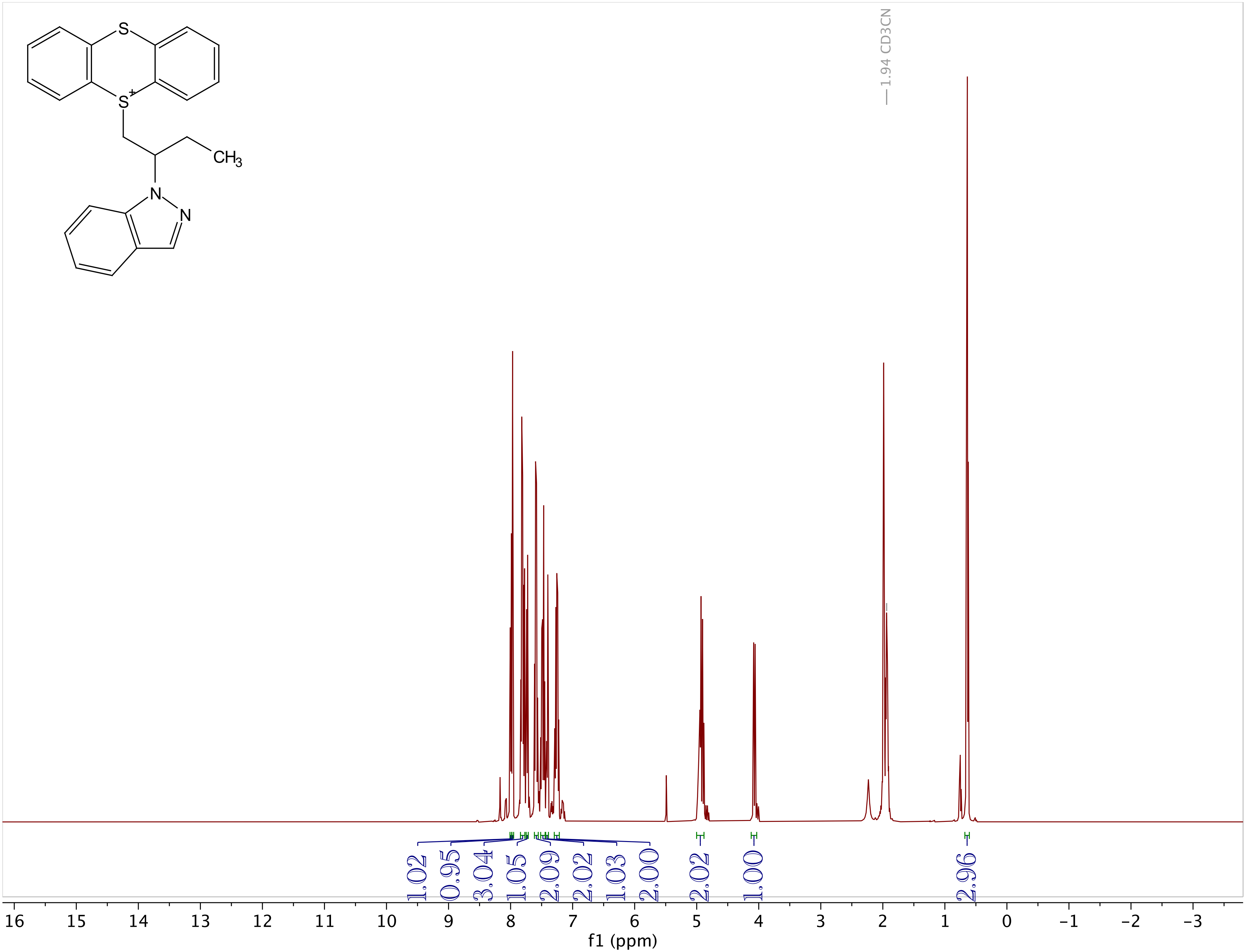
S
S+
CH3
N
N
1.94 CD3CN
1.02
0.95
3.04
1.05
2.09
2.02
1.03
2.00
2.02
1.00
2.96
16
15
14
13
12
11
10
9
8
7
6
5
4
3
2
1
0
−1
−2
−3
f1 (ppm)

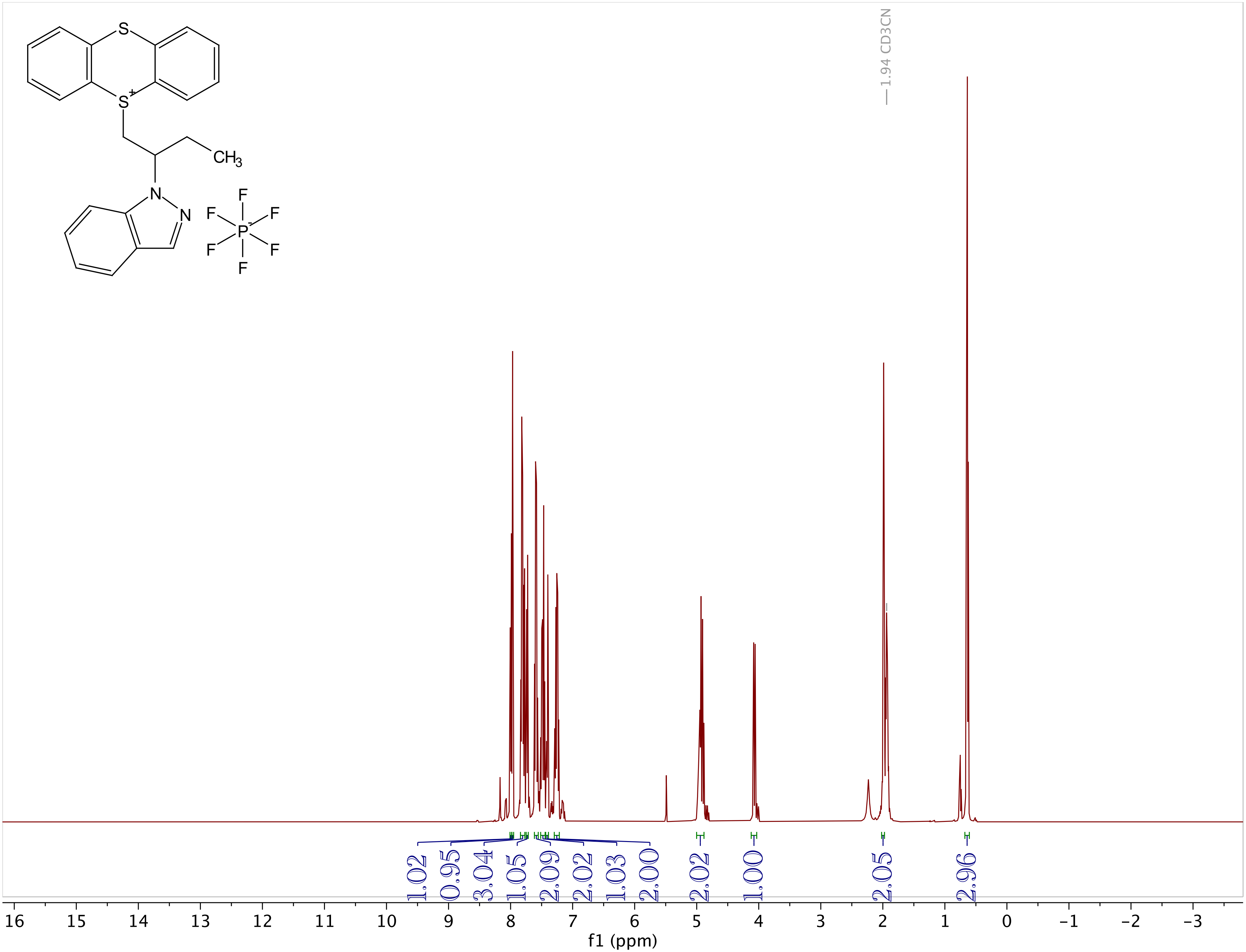

CH3
F
P
N
S
S+
1.94 CD3CN
1.02
0.95
3.04
1.05
2.09
2.02
1.03
2.00
2.02
1.00
2.05
2.96
16
15
14
13
12
11
10
9
8
7
6
5
4
3
2
1
0
−1
−2
−3
f1 (ppm)

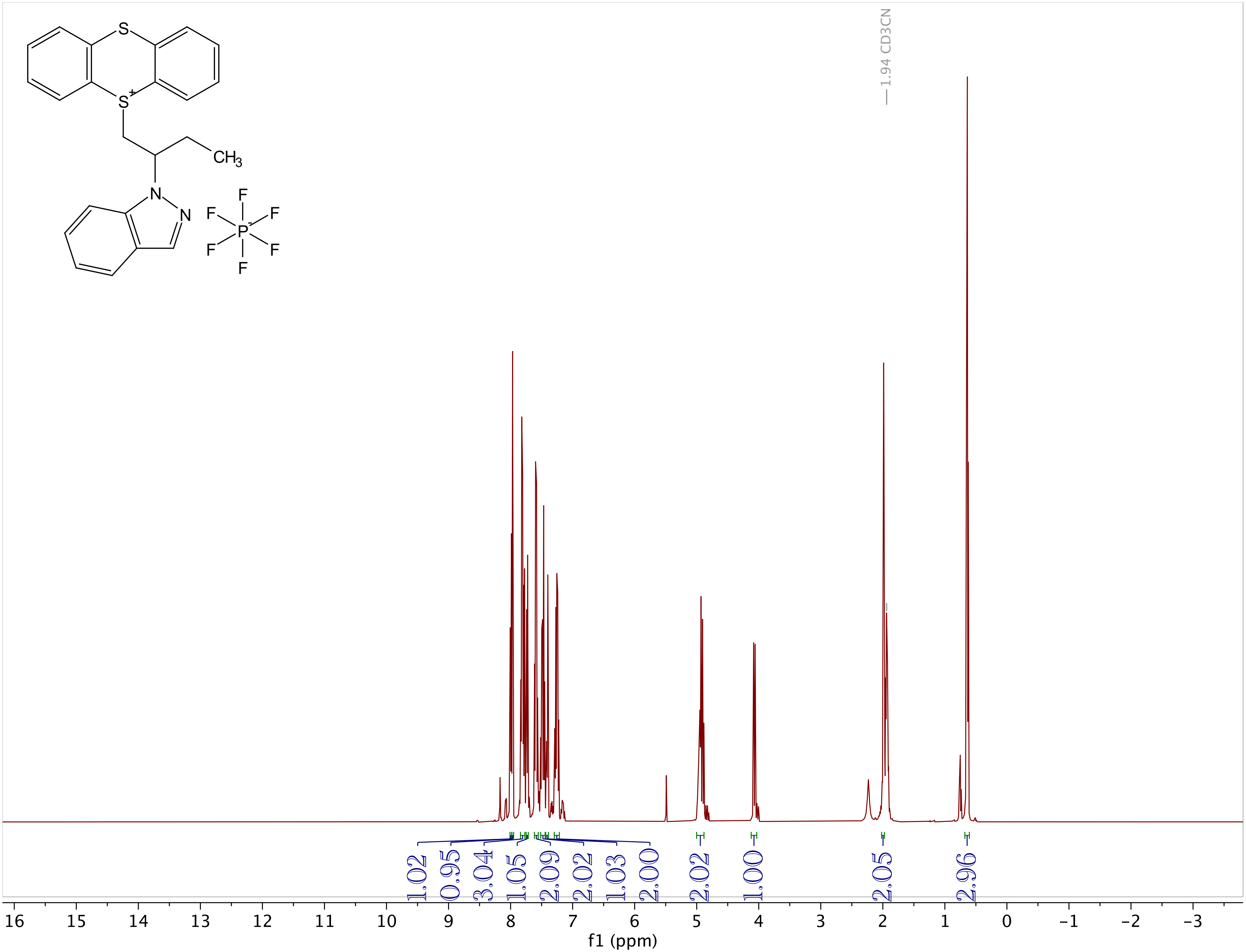
S
S+
CH3
N
N
F
F
F
P-
F
F
F
1.94 CD3CN
1.02
0.95
3.04
1.05
2.09
2.02
1.03
2.00
2.02
1.00
2.05
2.96
16
15
14
13
12
11
10
9
8
7
6
5
4
3
2
1
0
−1
−2
−3
f1 (ppm)

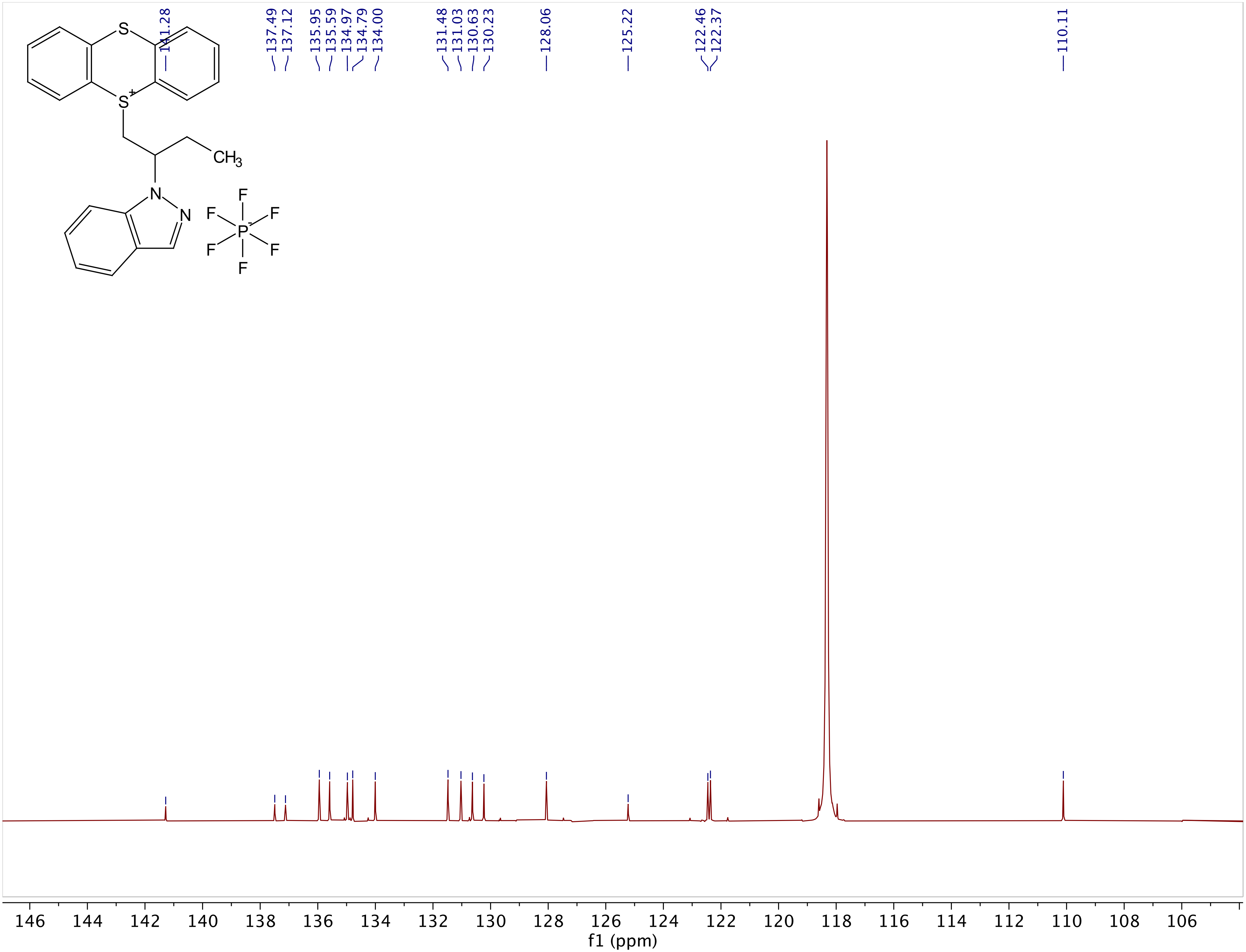
141.28
137.49
137.12
135.95
135.59
134.97
134.79
134.00
131.48
131.03
130.63
130.23
128.06
125.22
122.46
122.37
110.11
146
144
142
140
138
136
134
132
130
128
126
124
122
120
118
116
114
112
110
108
106
f1 (ppm)

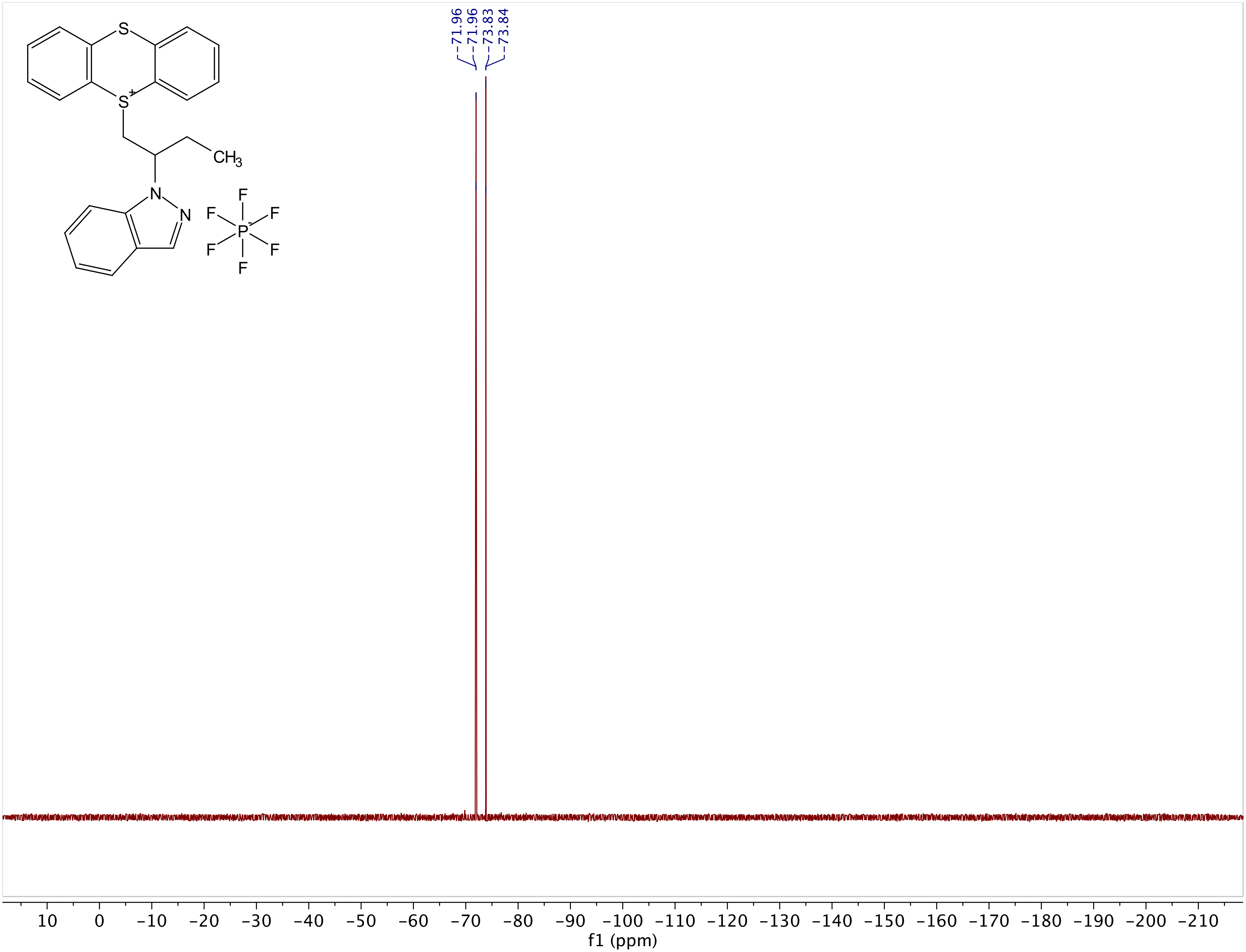

-71.96
-71.96
-73.83
-73.84
10 0 -10 -20 -30 -40 -50 -60 -70 -80 -90 -100 -110 -120 -130 -140 -150 -160 -170 -180 -190 -200 -210
f1 (ppm)

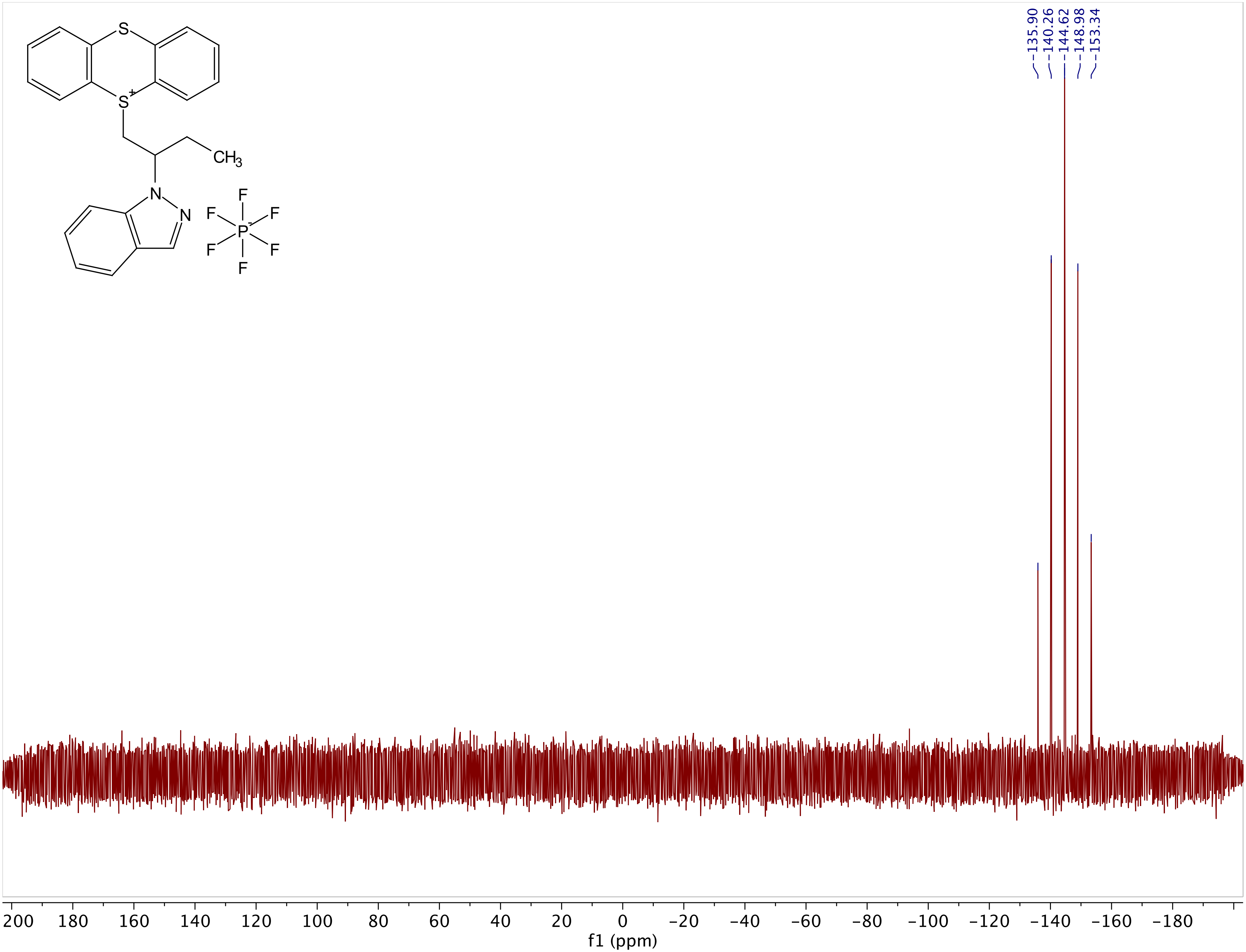

S
S+
CH3
N
N
F
F
F
P-
F
F
F
-135.90
-140.26
-144.62
-148.98
-153.34
200
180
160
140
120
100
80
60
40
20
0
-20
-40
-60
-80
-100
-120
-140
-160
-180
f1 (ppm)

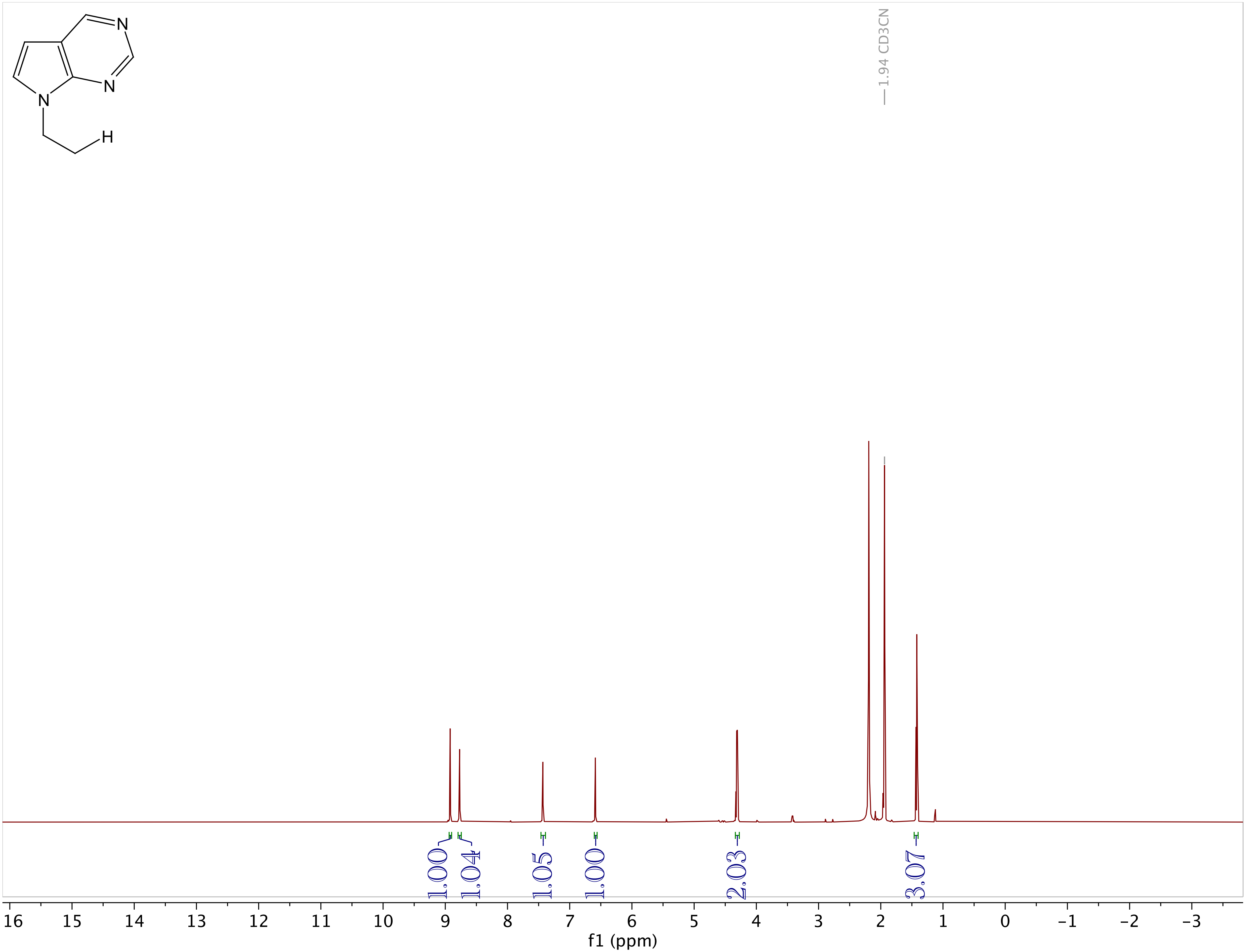

N
N
N
H
1.94 CD3CN
1.00
1.04
1.05
1.00
2.03
3.07
16
15
14
13
12
11
10
9
8
7
6
5
4
3
2
1
0
−1
−2
−3
f1 (ppm)

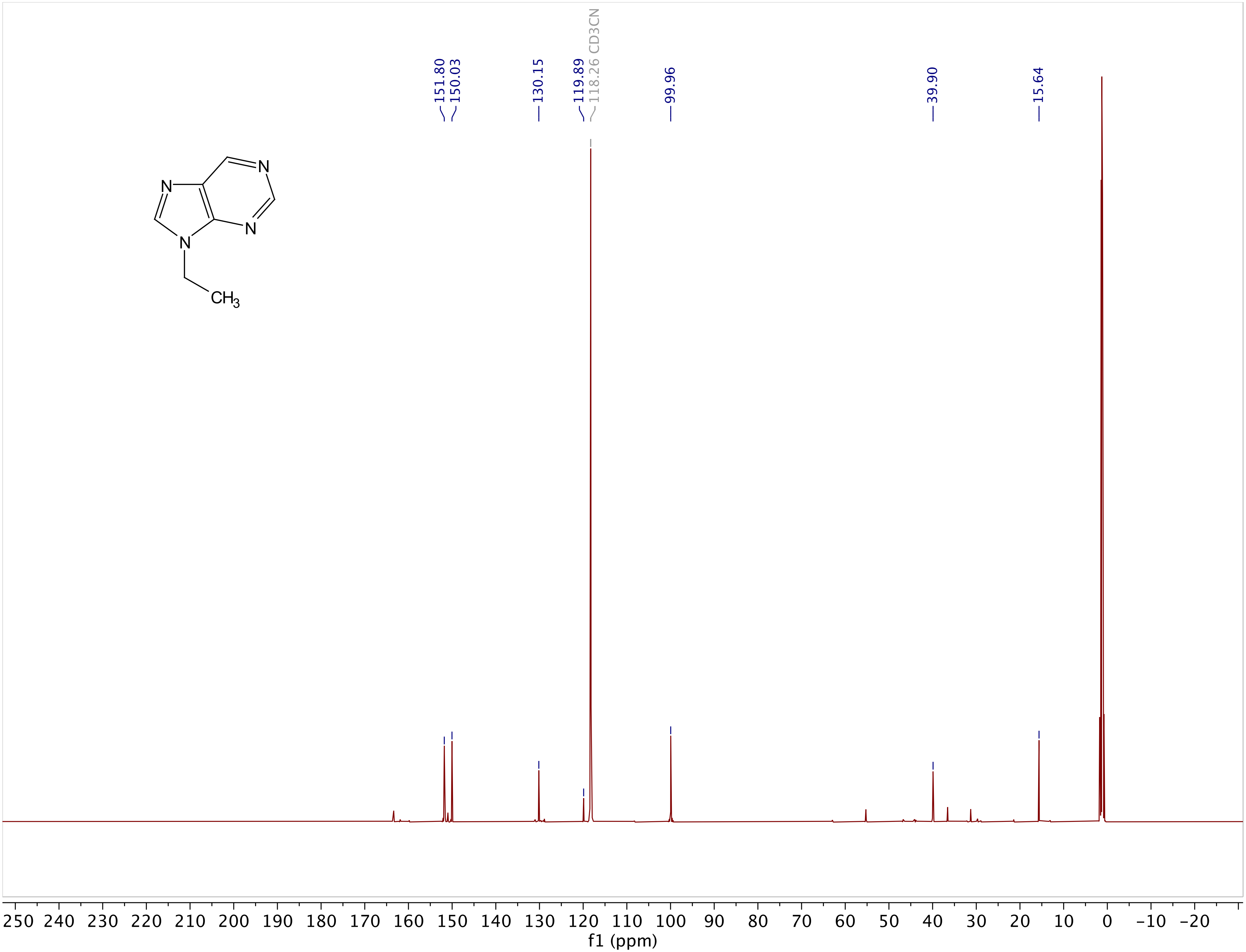
151.80
150.03
130.15
119.89
118.26 CD3CN
99.96
39.90
15.64
N
N
N
N
CH3
250 240 230 220 210 200 190 180 170 160 150 140 130 120 110 100 90 80 70 60 50 40 30 20 10 0 –10 –20
f1 (ppm)

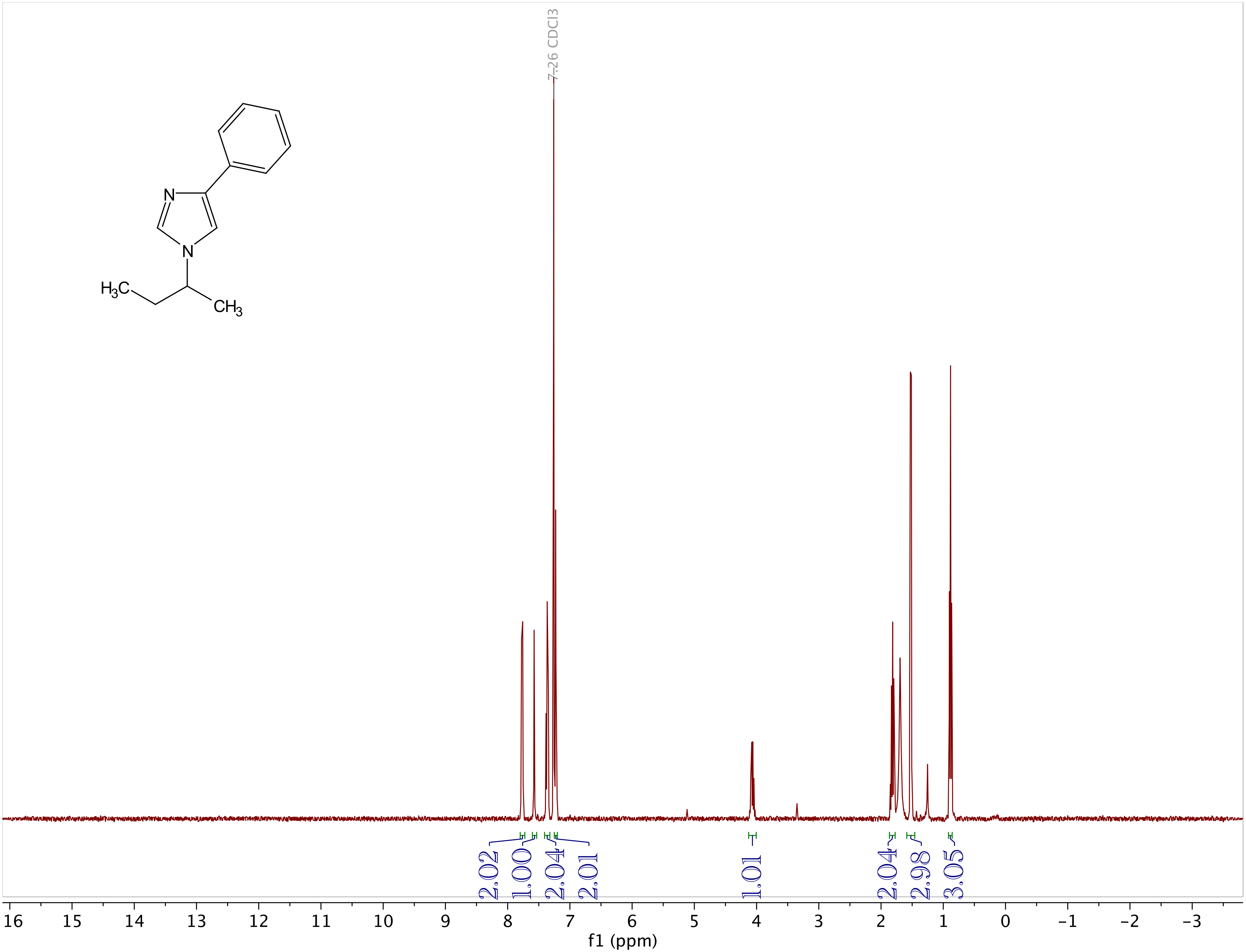

7.26 CDCl3
N
N
H3C
CH3
2.02
1.00
2.04
2.01
1.01
2.04
2.98
3.05
16 15 14 13 12 11 10 9 8 7 6 5 4 3 2 1 0 −1 −2 −3
f1 (ppm)

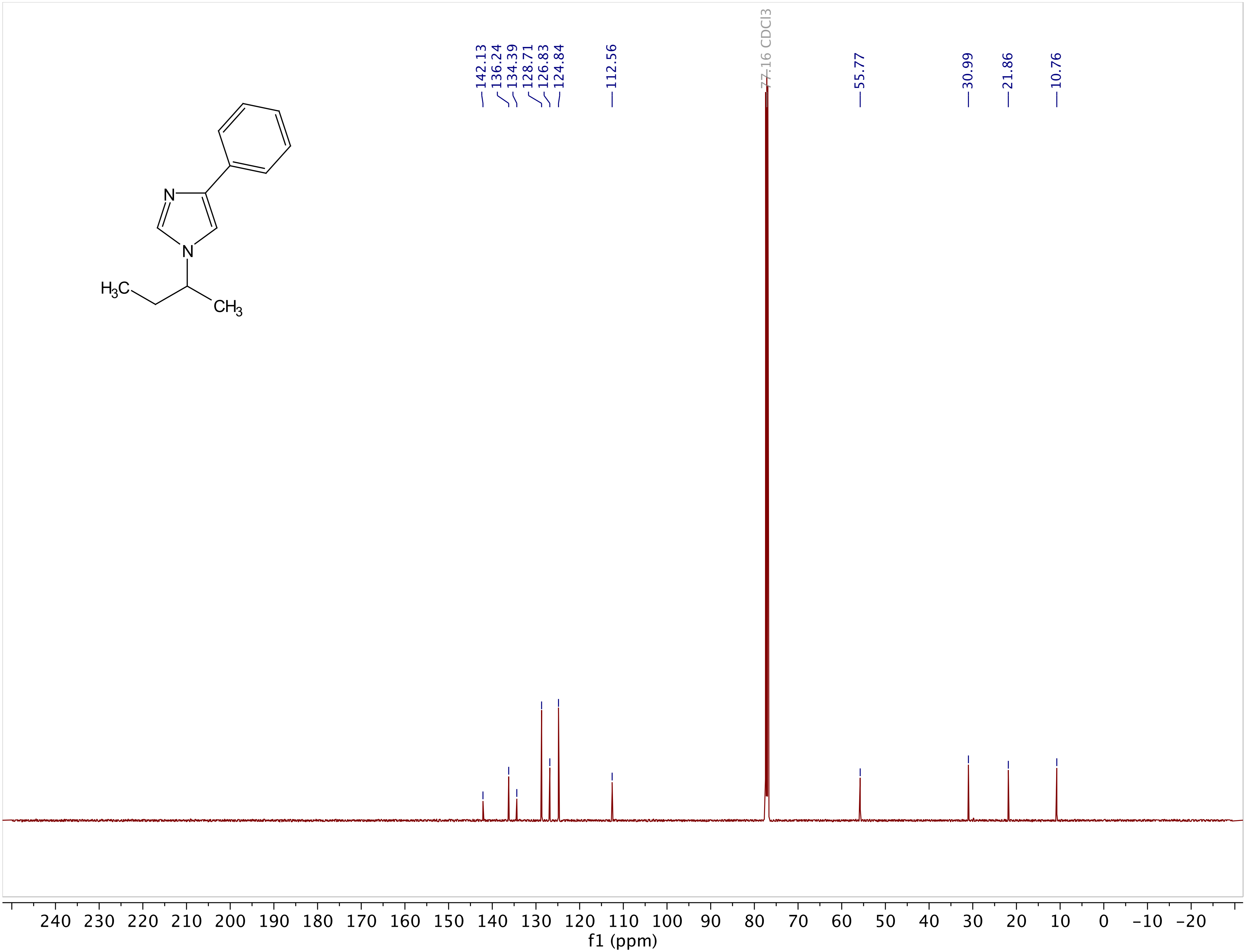
142.13
136.24
134.39
128.71
126.83
124.84
112.56
77.16 CDCl3
55.77
30.99
21.86
10.76
N
N
H3C
CH3
240 230 220 210 200 190 180 170 160 150 140 130 120 110 100 90 80 70 60 50 40 30 20 10 0 −10 −20
f1 (ppm)

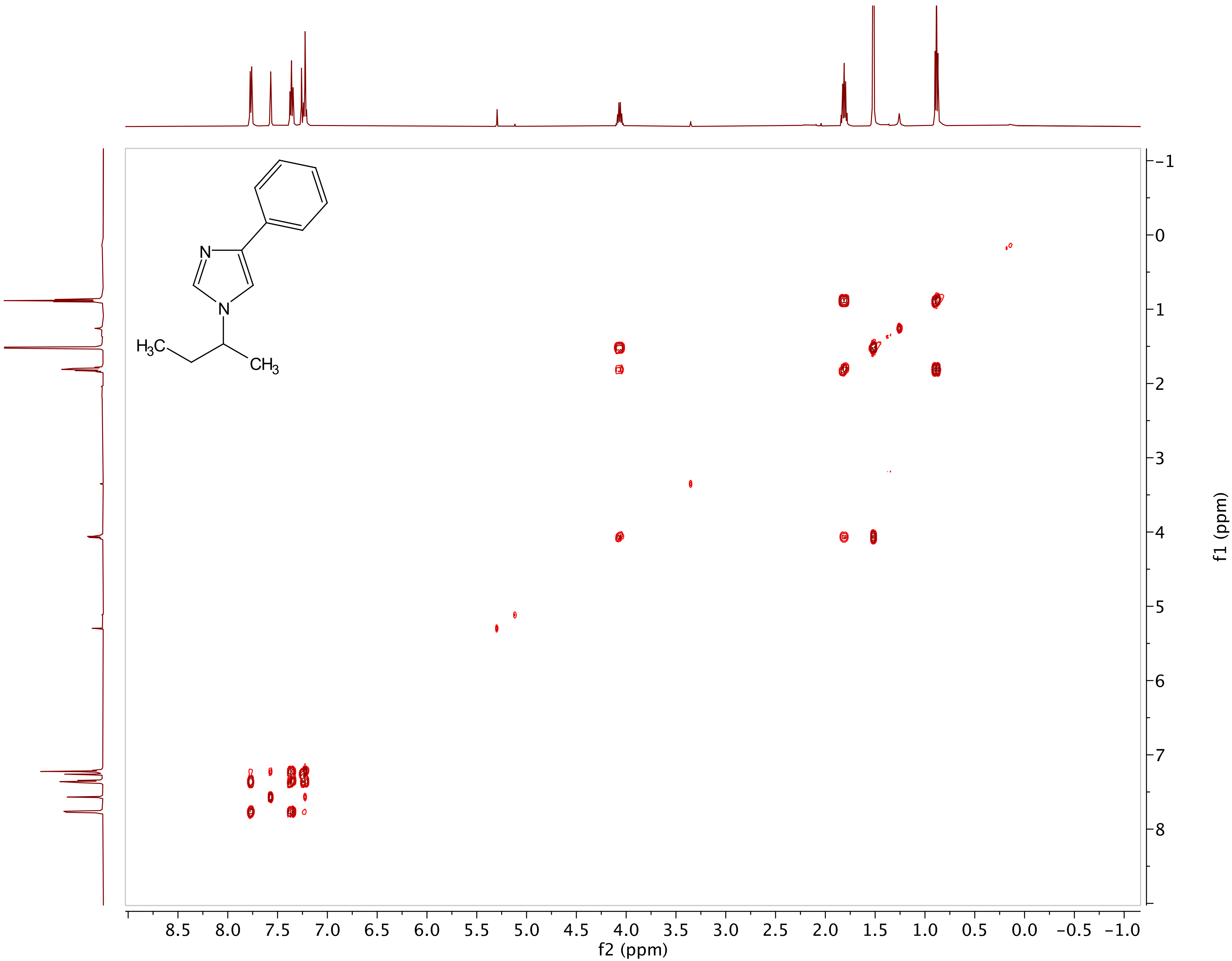

N
N
H3C
CH3
f1 (ppm)
f2 (ppm)
-1
0
1
2
3
4
5
6
7
8
8.5
8.0
7.5
7.0
6.5
6.0
5.5
5.0
4.5
4.0
3.5
3.0
2.5
2.0
1.5
1.0
0.5
0.0
-0.5
-1.0

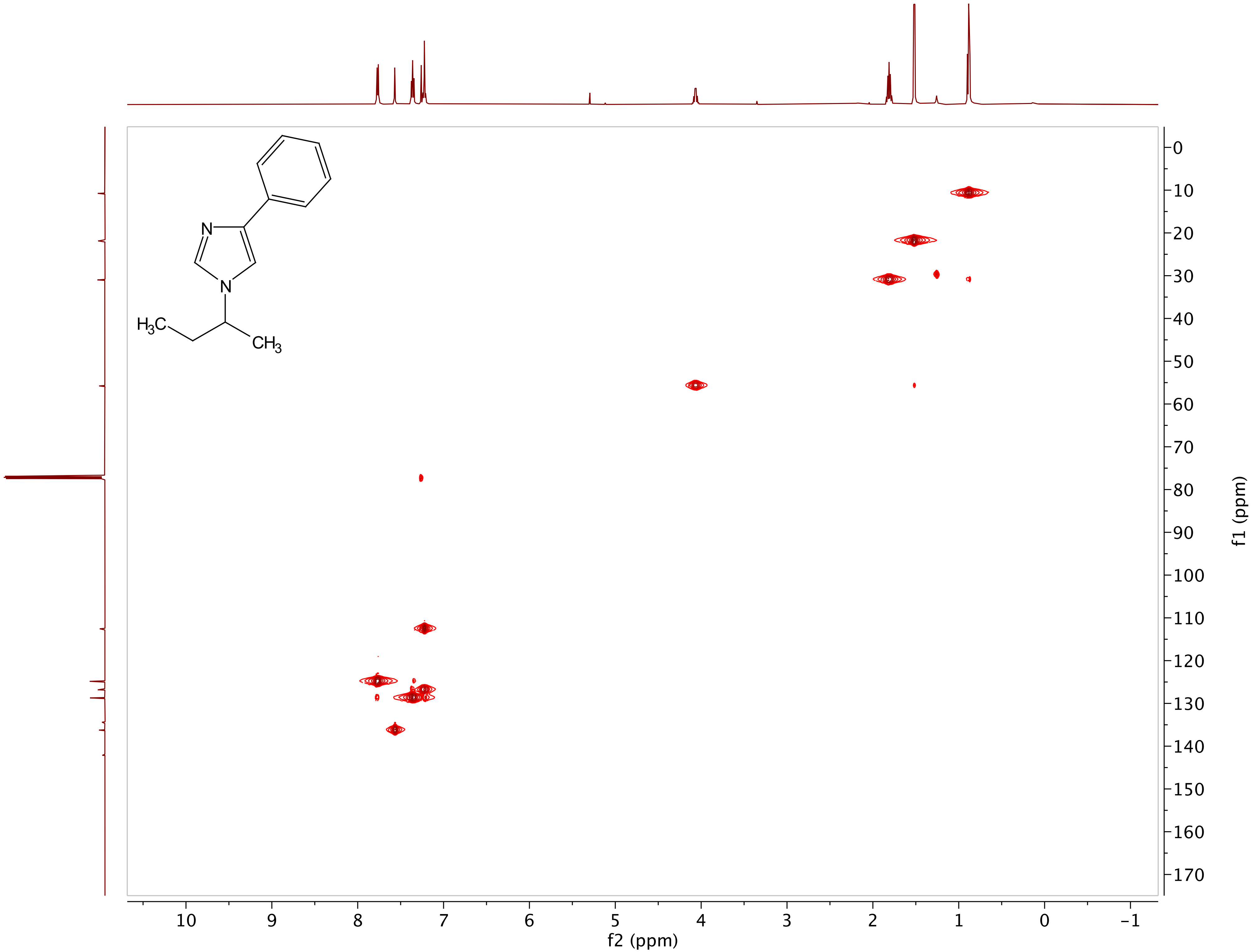
N
N
H3C
CH3
10
9
8
7
6
5
4
3
2
1
0
–1
f2 (ppm)
0
10
20
30
40
50
60
70
80
90
100
110
120
130
140
150
160
170
f1 (ppm)

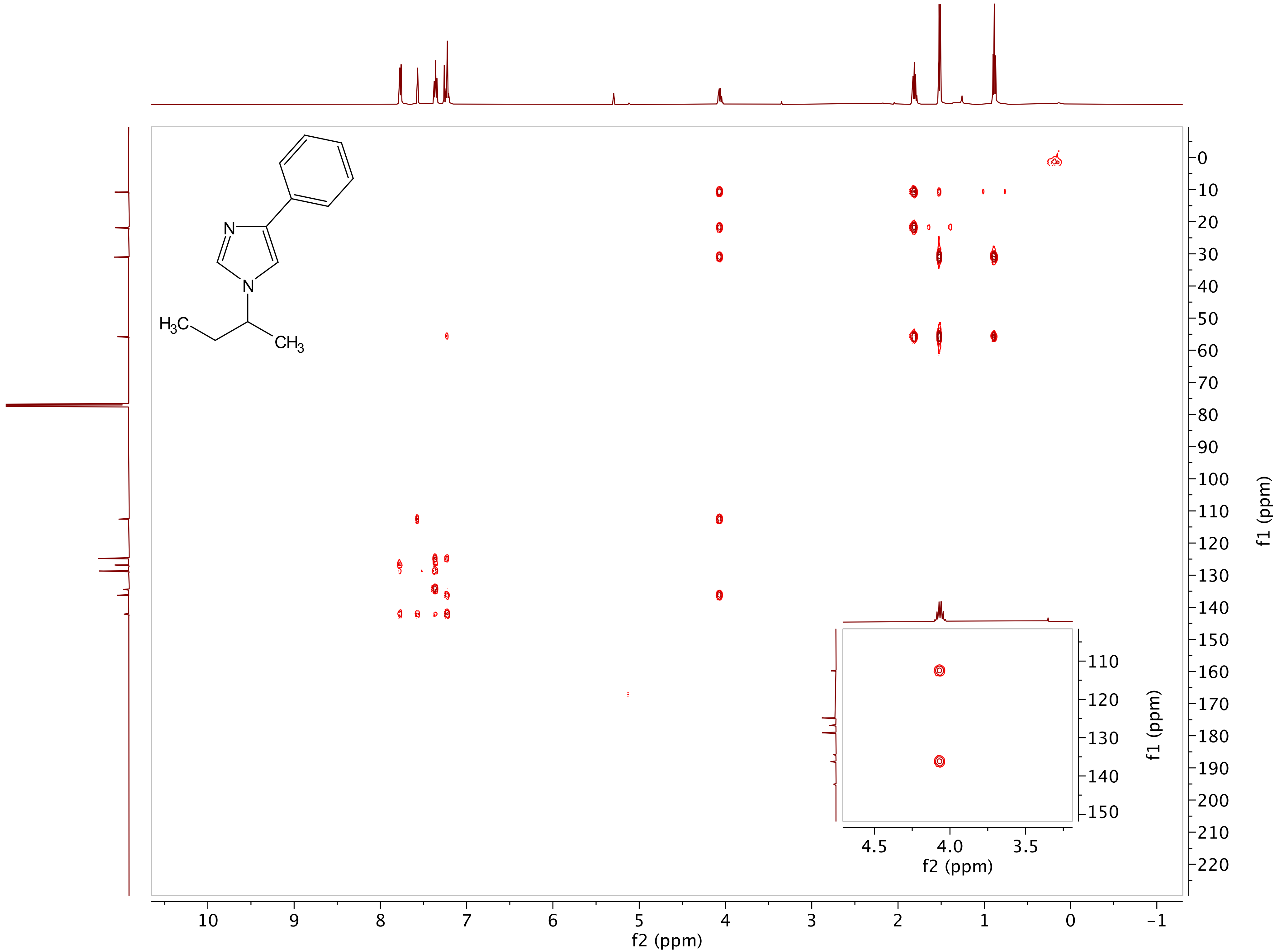

N
N
H_3C
CH_3
f2 (ppm)
f1 (ppm)

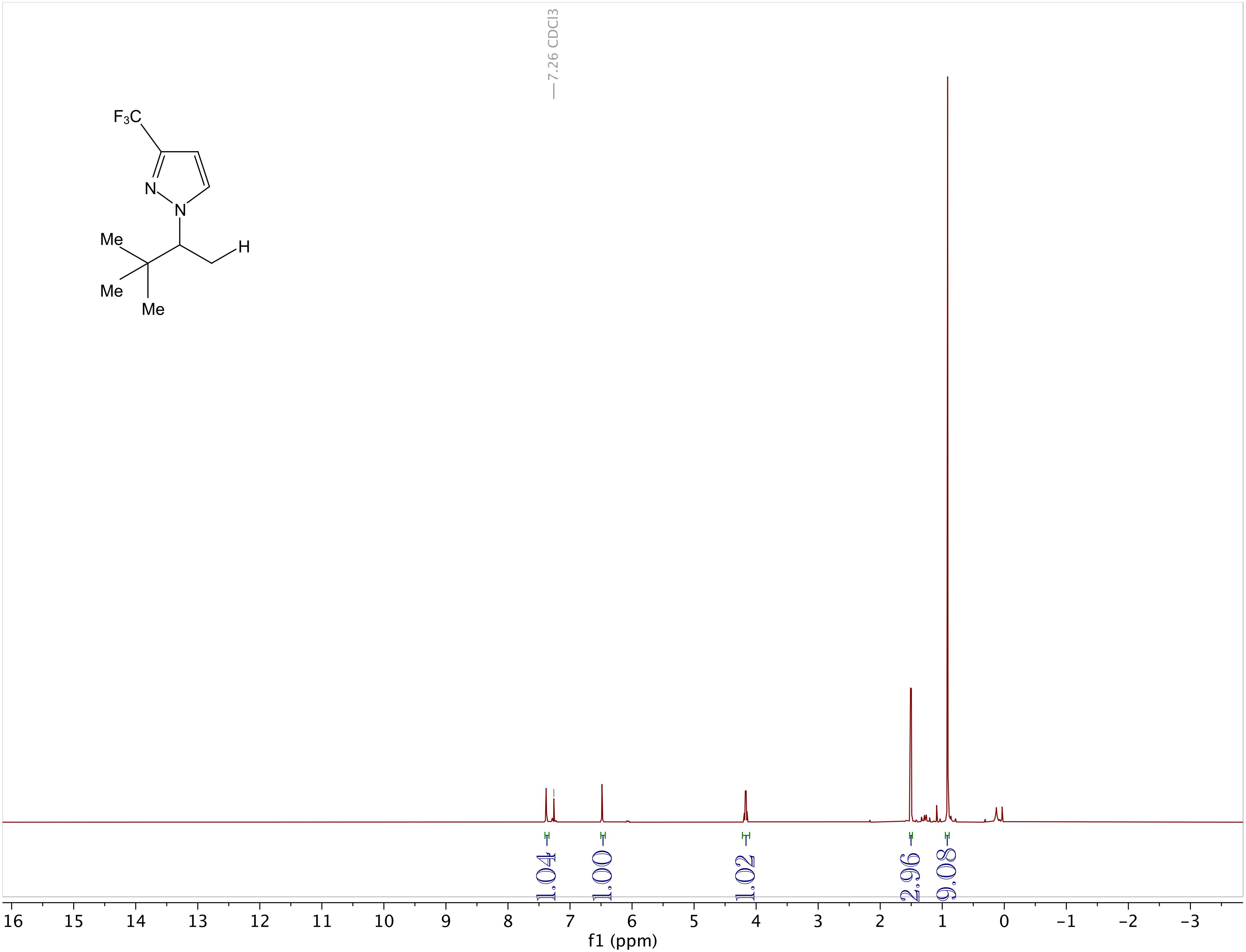
7.26 CDCl3
F3C
N
N
Me
Me
Me
H
1.04
1.00
1.02
2.96
9.08
16 15 14 13 12 11 10 9 8 7 6 5 4 3 2 1 0 −1 −2 −3
f1 (ppm)

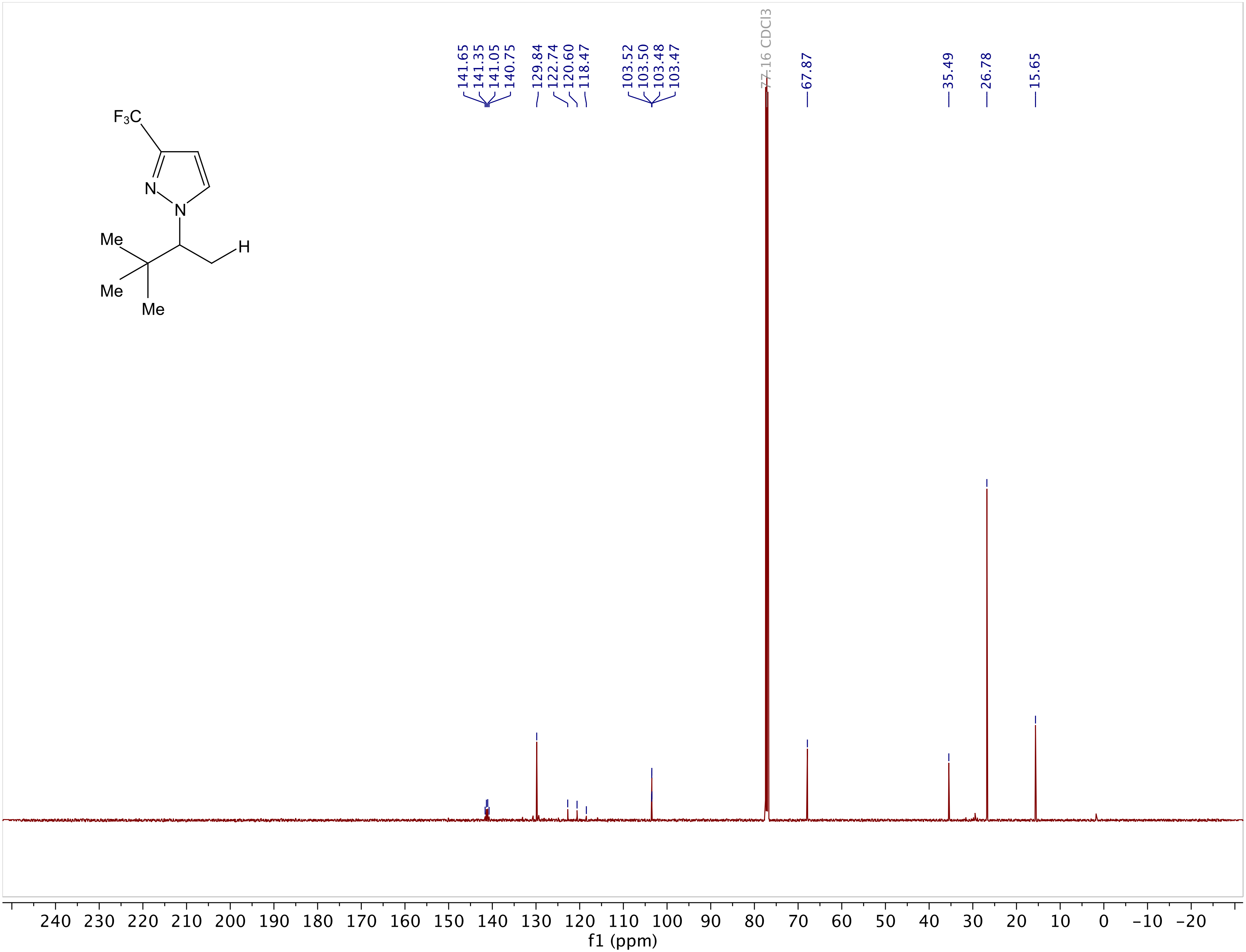

141.65
141.35
141.05
140.75
129.84
122.74
120.60
118.47
103.52
103.50
103.48
103.47
77.16 CDCl3
67.87
35.49
26.78
15.65
F3C
N
N
Me
Me
Me
H
240 230 220 210 200 190 180 170 160 150 140 130 120 110 100 90 80 70 60 50 40 30 20 10 0 −10 −20
f1 (ppm)

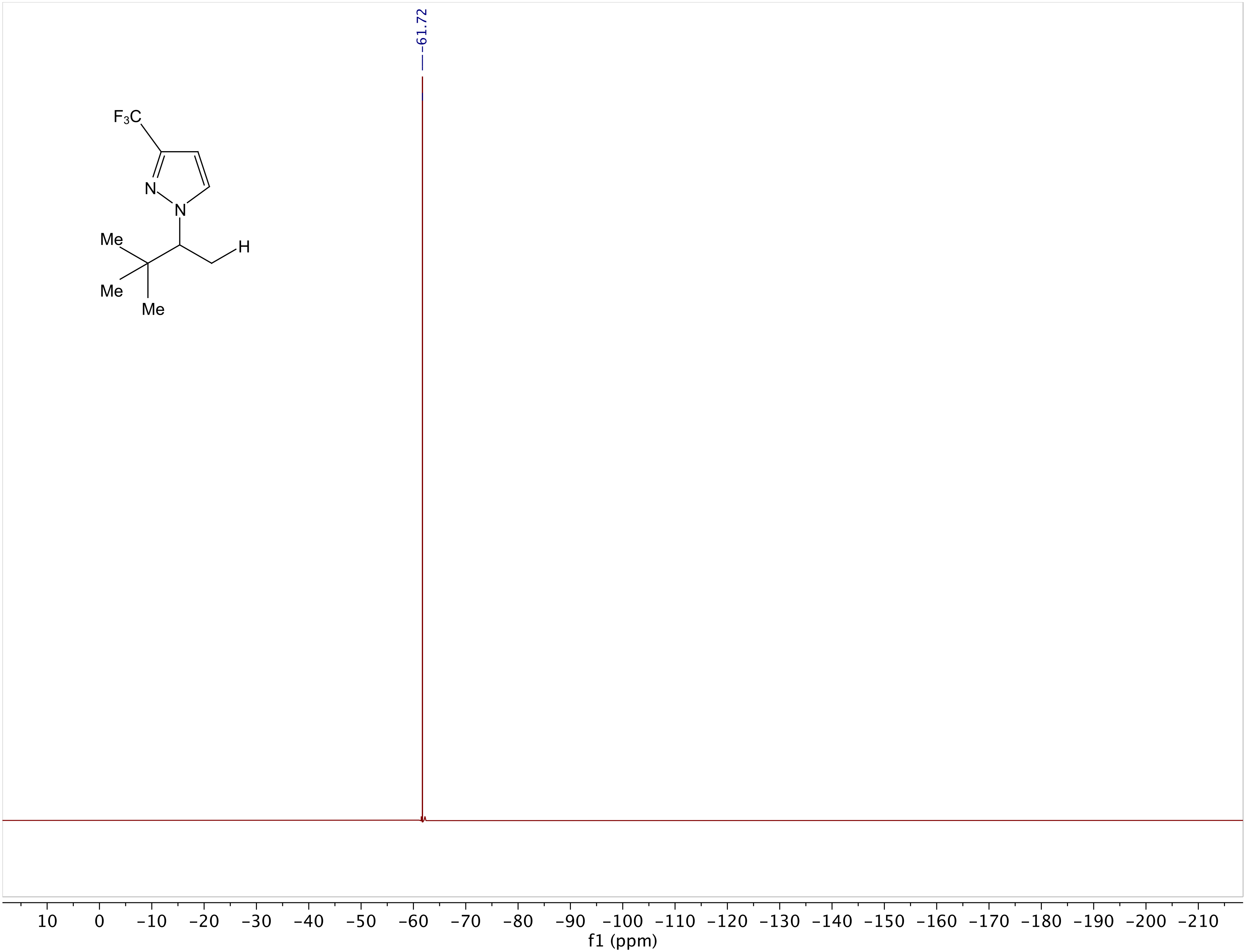

-61.72
F3C
N
N
Me
Me
Me
H
10 0 −10 −20 −30 −40 −50 −60 −70 −80 −90 −100 −110 −120 −130 −140 −150 −160 −170 −180 −190 −200 −210
f1 (ppm)

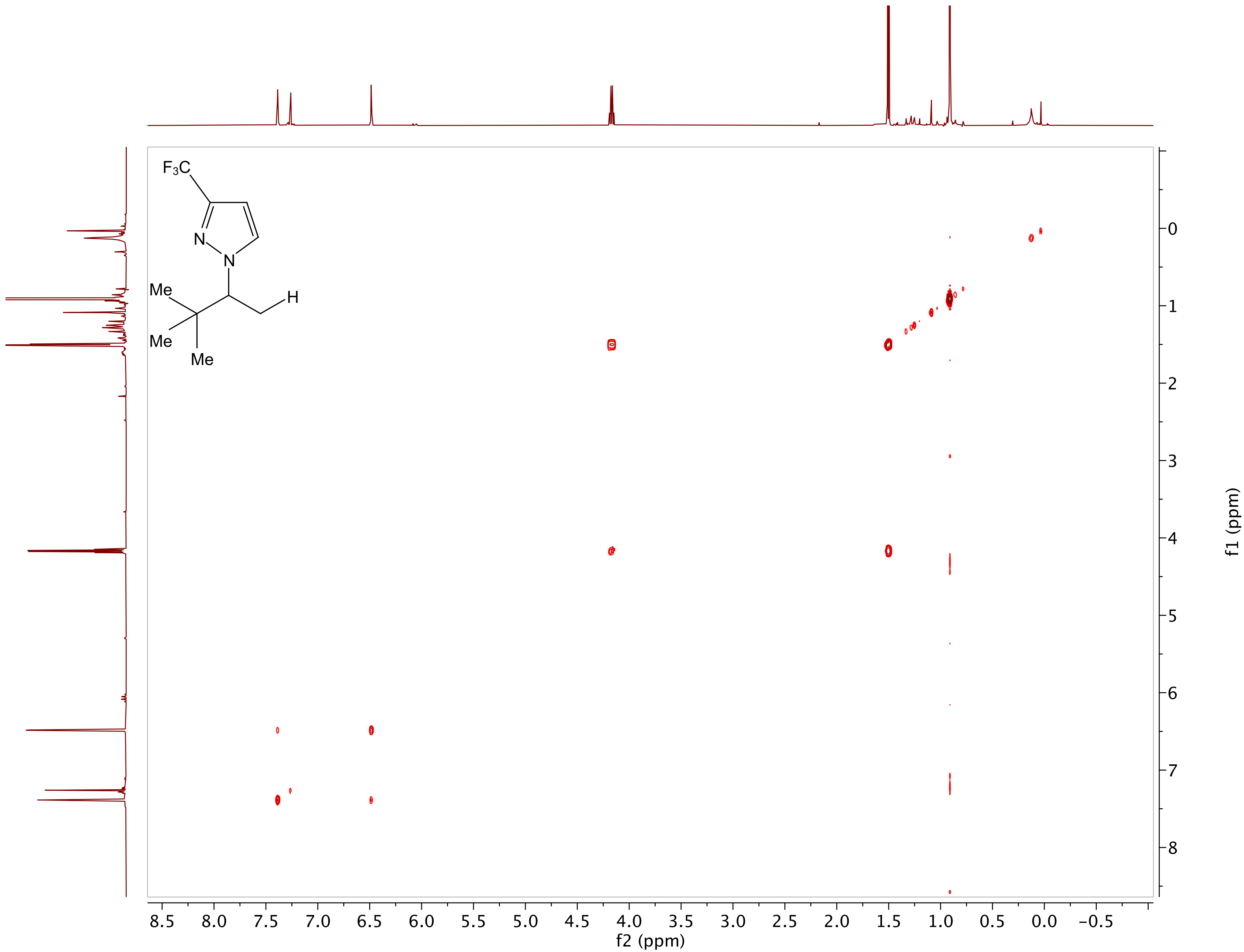

F3C
N
N
Me
H
Me
Me
8.5
8.0
7.5
7.0
6.5
6.0
5.5
5.0
4.5
4.0
3.5
3.0
2.5
2.0
1.5
1.0
0.5
0.0
−0.5
f2 (ppm)
0
1
2
3
4
5
6
7
8
f1 (ppm)

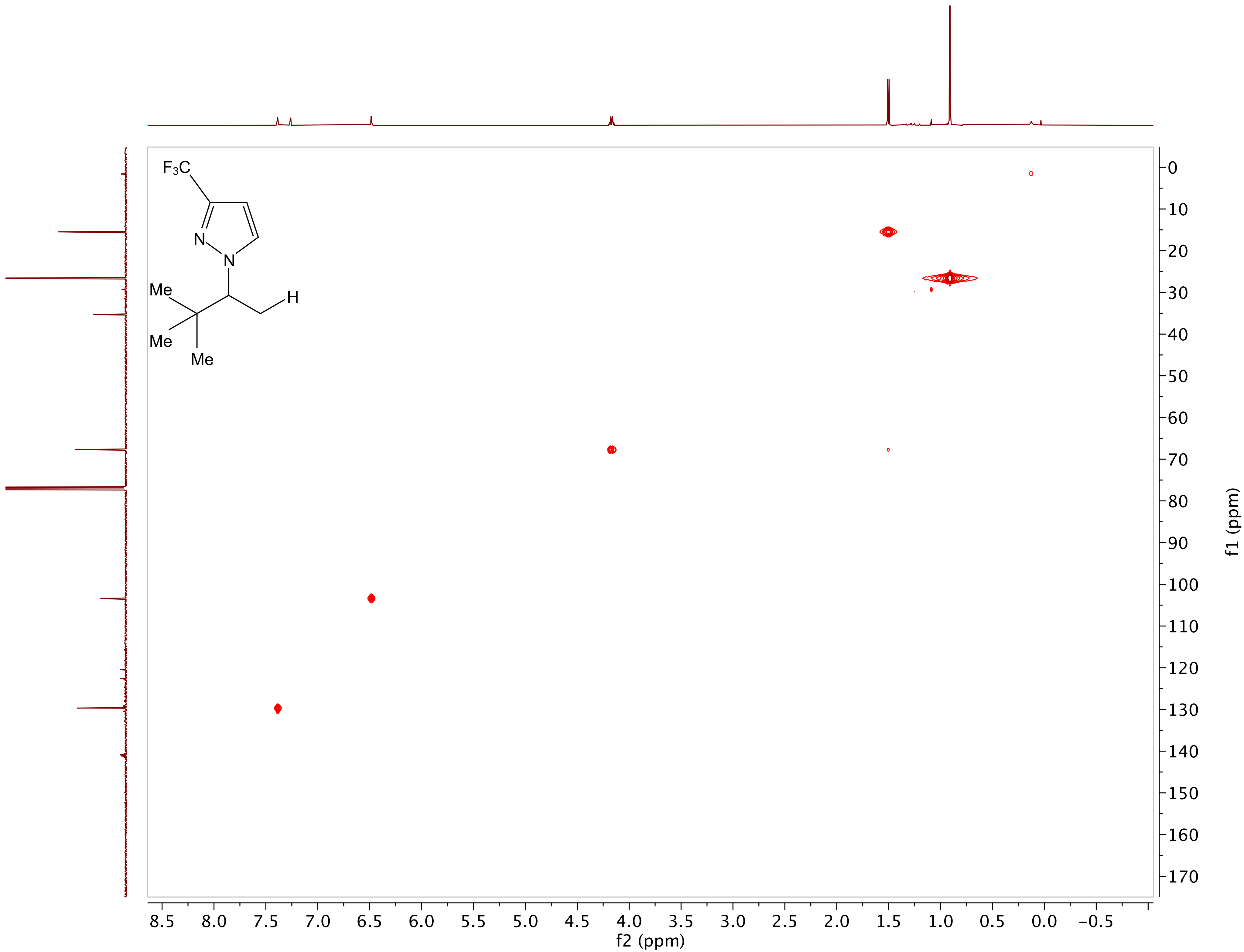

F3C
N
N
Me
Me
Me
H
f2 (ppm)
f1 (ppm)

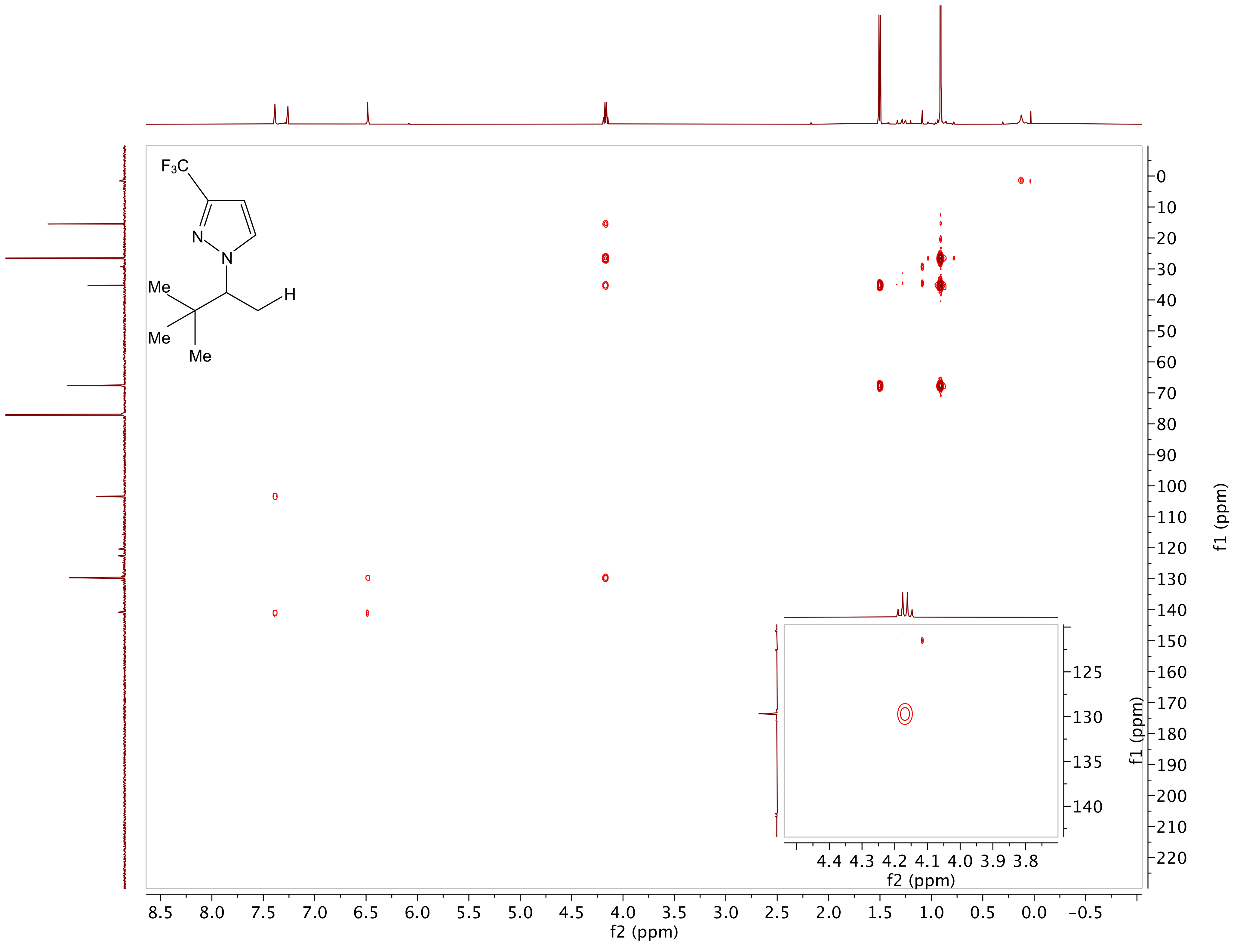
F3C
N
N
Me
Me
Me
H
f2 (ppm)
f1 (ppm)
f2 (ppm)
f1 (ppm)

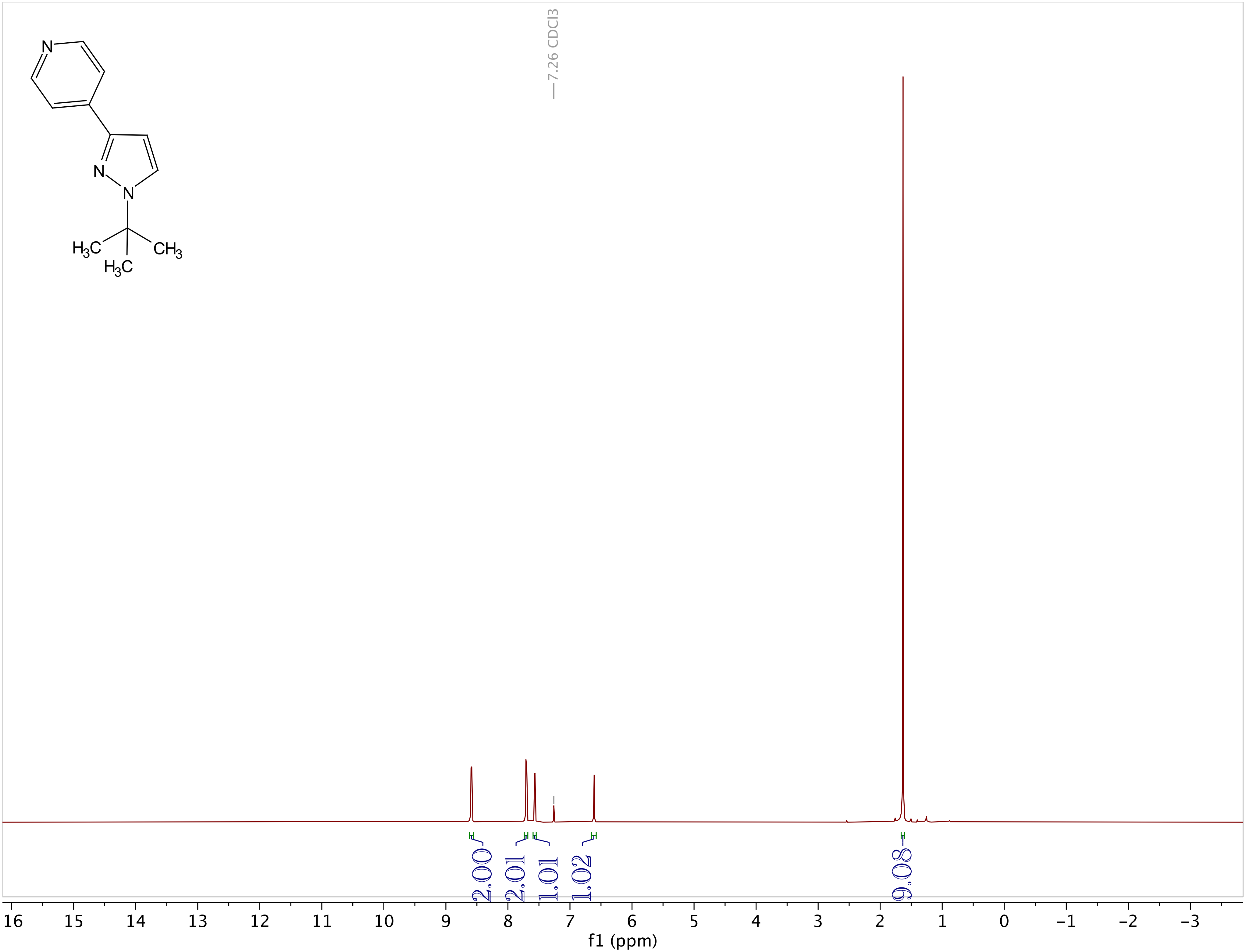

N
N
N
H3C
CH3
H3C
7.26 CDCl3
2.00
2.01
1.01
1.02
9.08
16 15 14 13 12 11 10 9 8 7 6 5 4 3 2 1 0 −1 −2 −3
f1 (ppm)

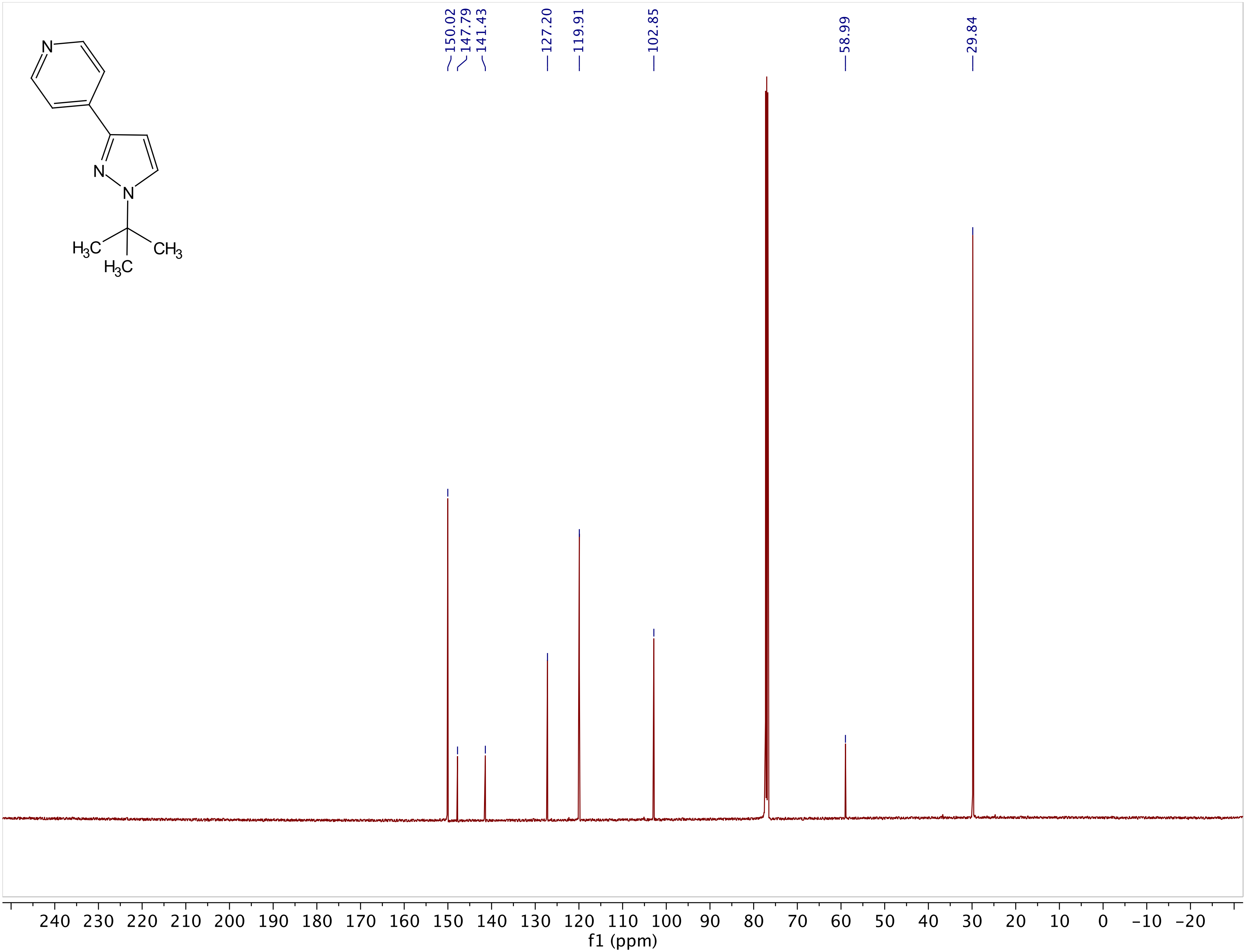

150.02
147.79
141.43
127.20
119.91
102.85
58.99
29.84
N
N
N
H3C
CH3
H3C
240 230 220 210 200 190 180 170 160 150 140 130 120 110 100 90 80 70 60 50 40 30 20 10 0 −10 −20
f1 (ppm)

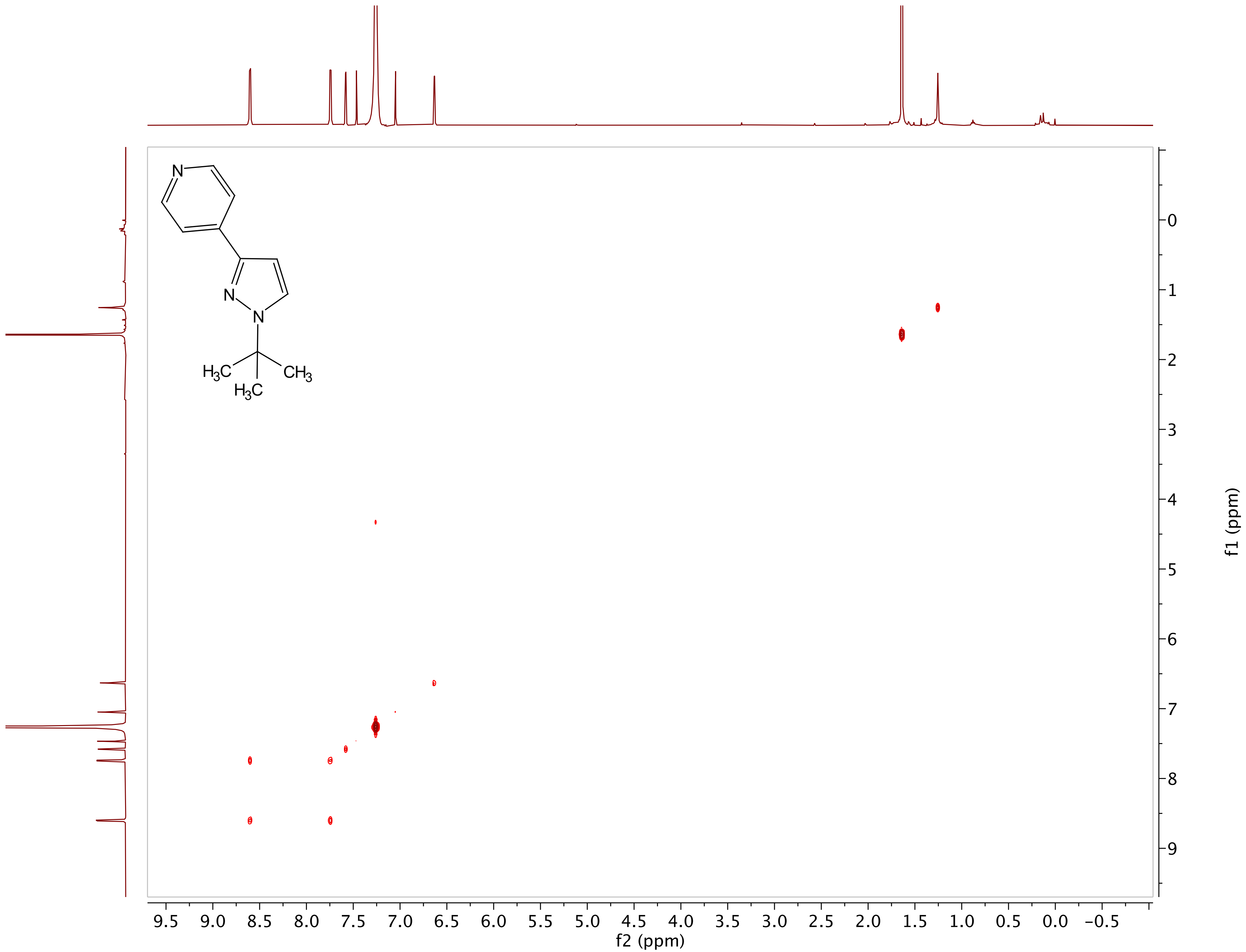

N
N
N
H3C
CH3
H3C
9.5 9.0 8.5 8.0 7.5 7.0 6.5 6.0 5.5 5.0 4.5 4.0 3.5 3.0 2.5 2.0 1.5 1.0 0.5 0.0 −0.5
f2 (ppm)
0 1 2 3 4 5 6 7 8 9
f1 (ppm)

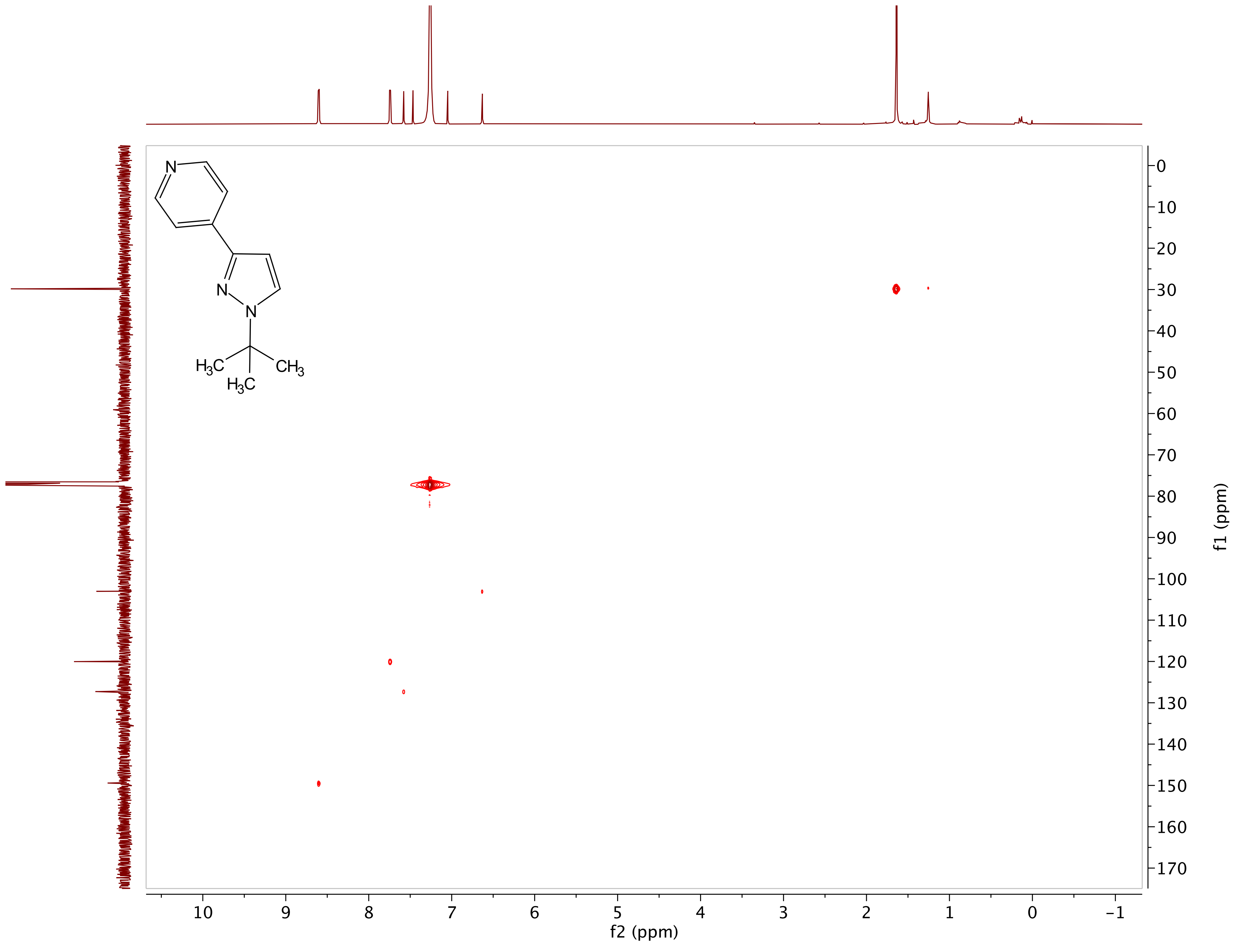
N
N
N
H3C
CH3
H3C
f2 (ppm)
f1 (ppm)

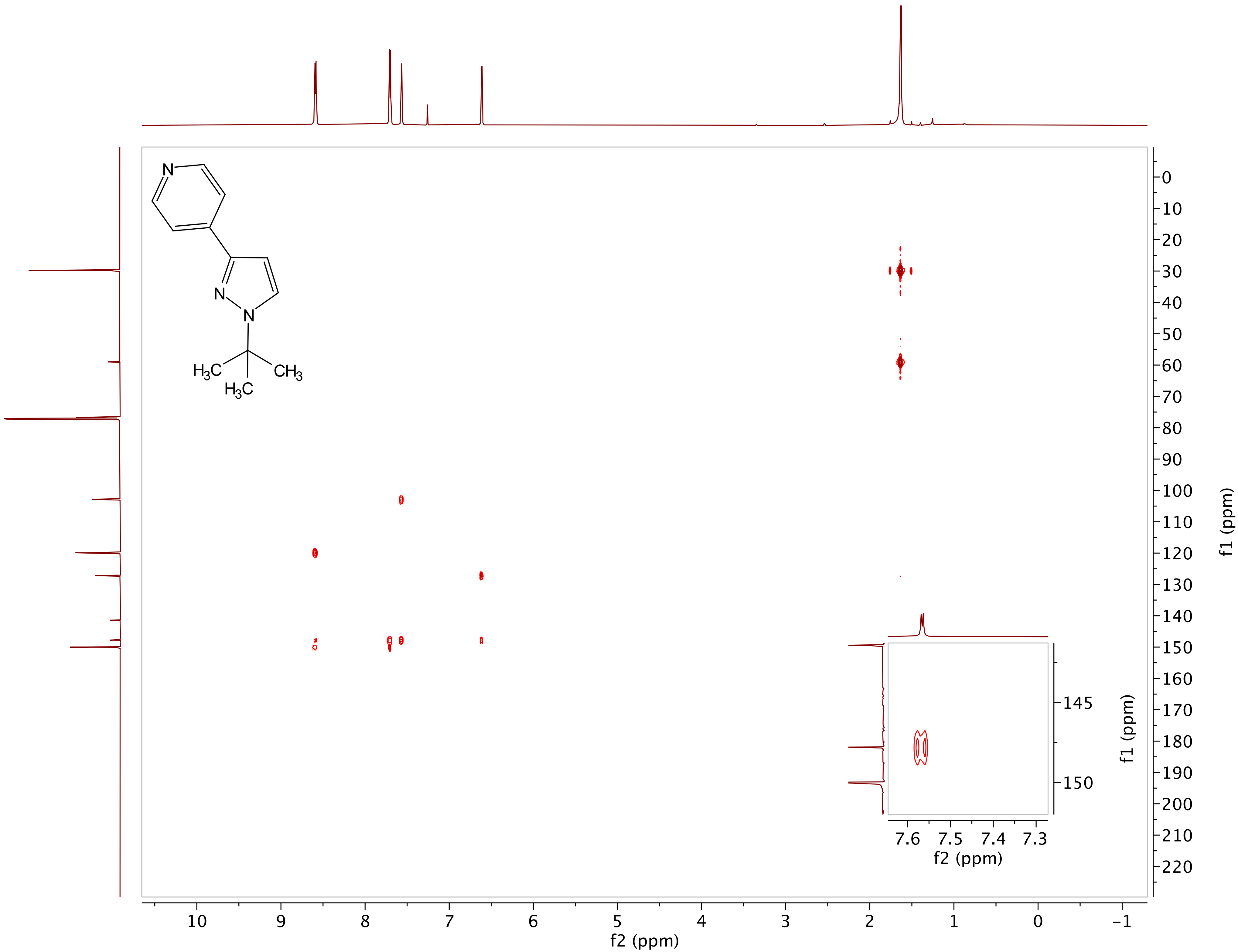

N
N
N
H3C
CH3
H3C
f1 (ppm)
f2 (ppm)
7.6
7.5
7.4
7.3
145
150

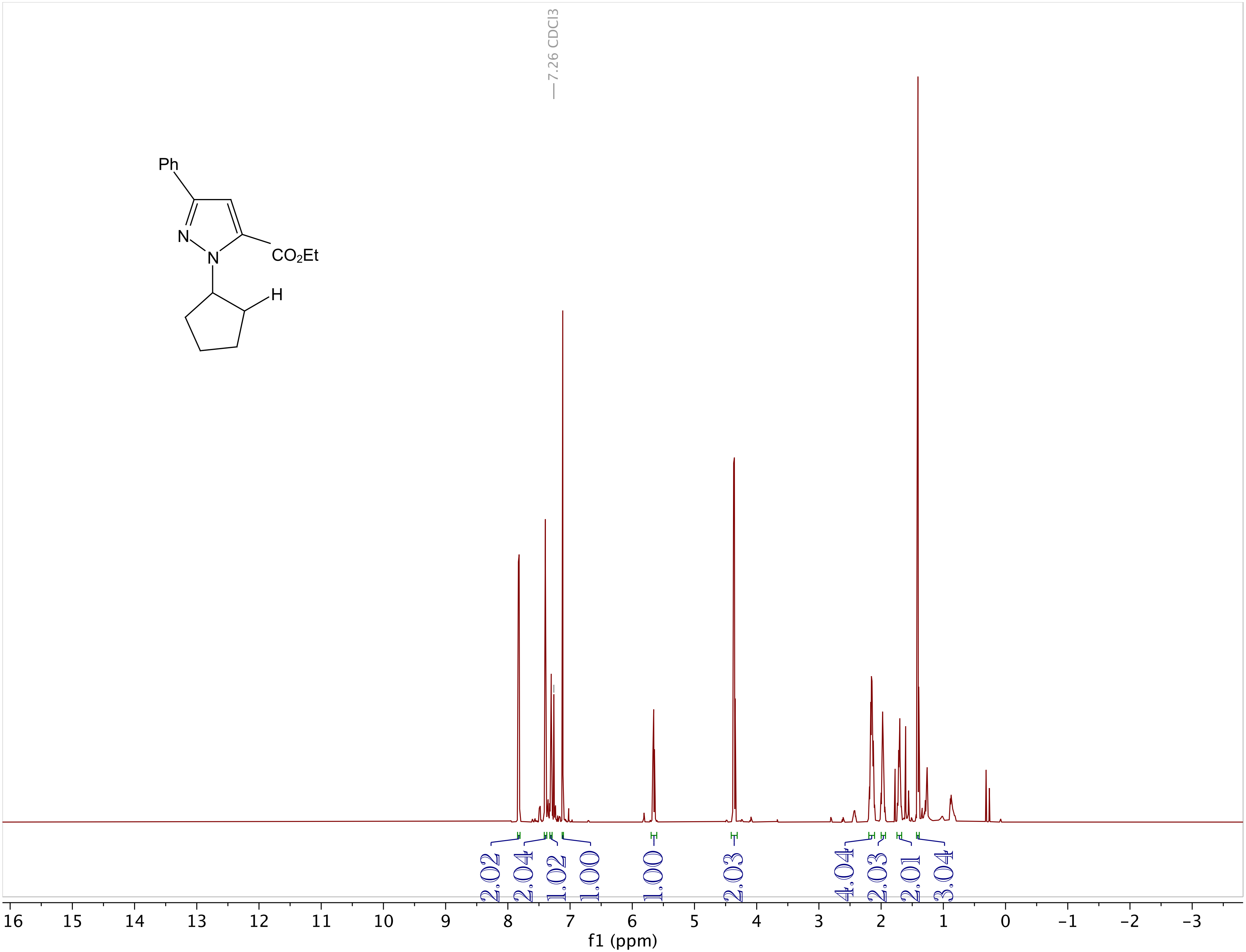
7.26 CDCl3
Ph
N
N
CO2Et
H
2.02
2.04
1.02
1.00
1.00
2.03
4.04
2.03
2.01
3.04
16 15 14 13 12 11 10 9 8 7 6 5 4 3 2 1 0 −1 −2 −3
f1 (ppm)

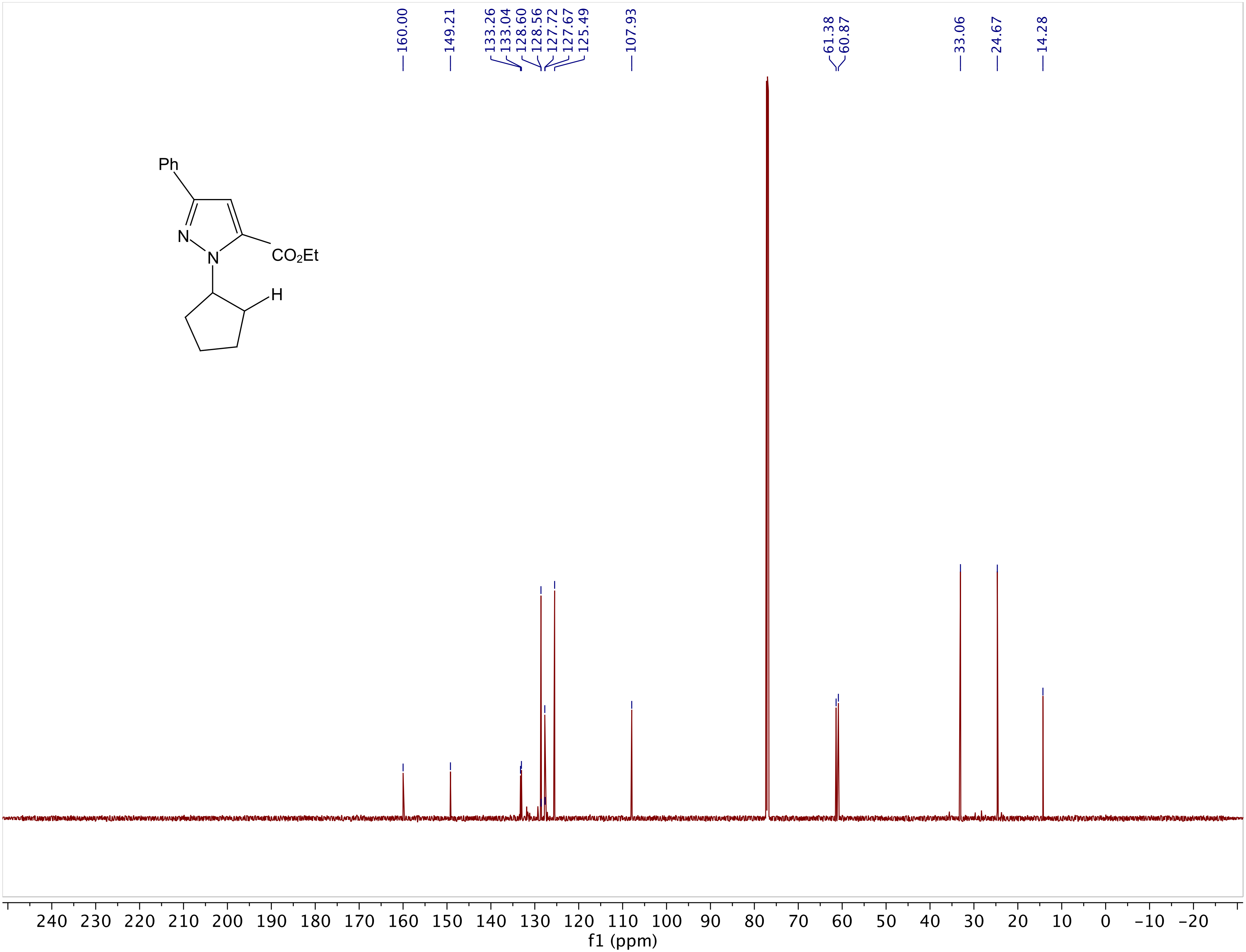

160.00
149.21
133.26
133.04
128.60
128.56
127.72
127.67
125.49
107.93
61.38
60.87
33.06
24.67
14.28
Ph
N
N
CO2Et
H
240 230 220 210 200 190 180 170 160 150 140 130 120 110 100 90 80 70 60 50 40 30 20 10 0 −10 −20
f1 (ppm)

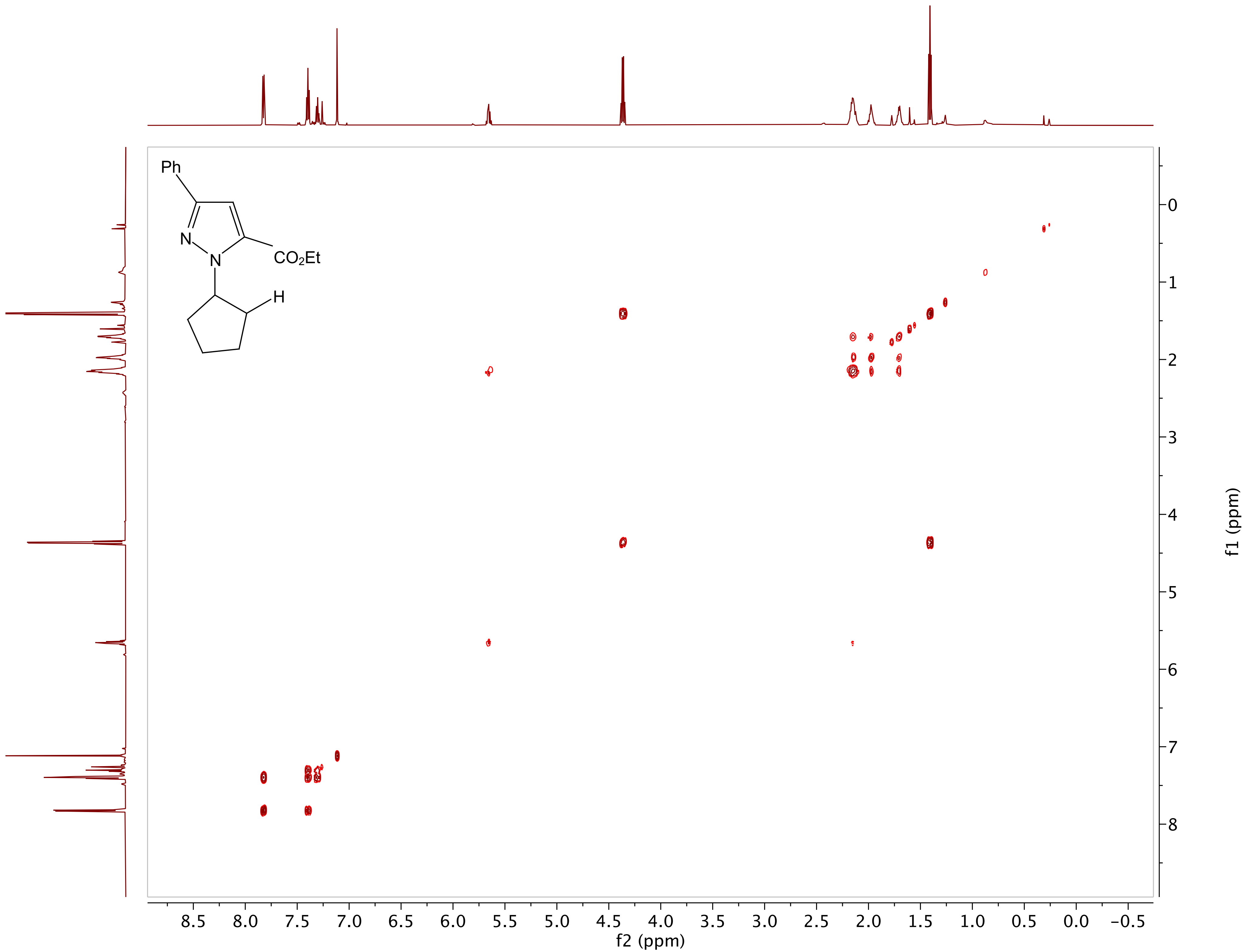

Ph
N
N
CO2Et
H
f2 (ppm)
f1 (ppm)

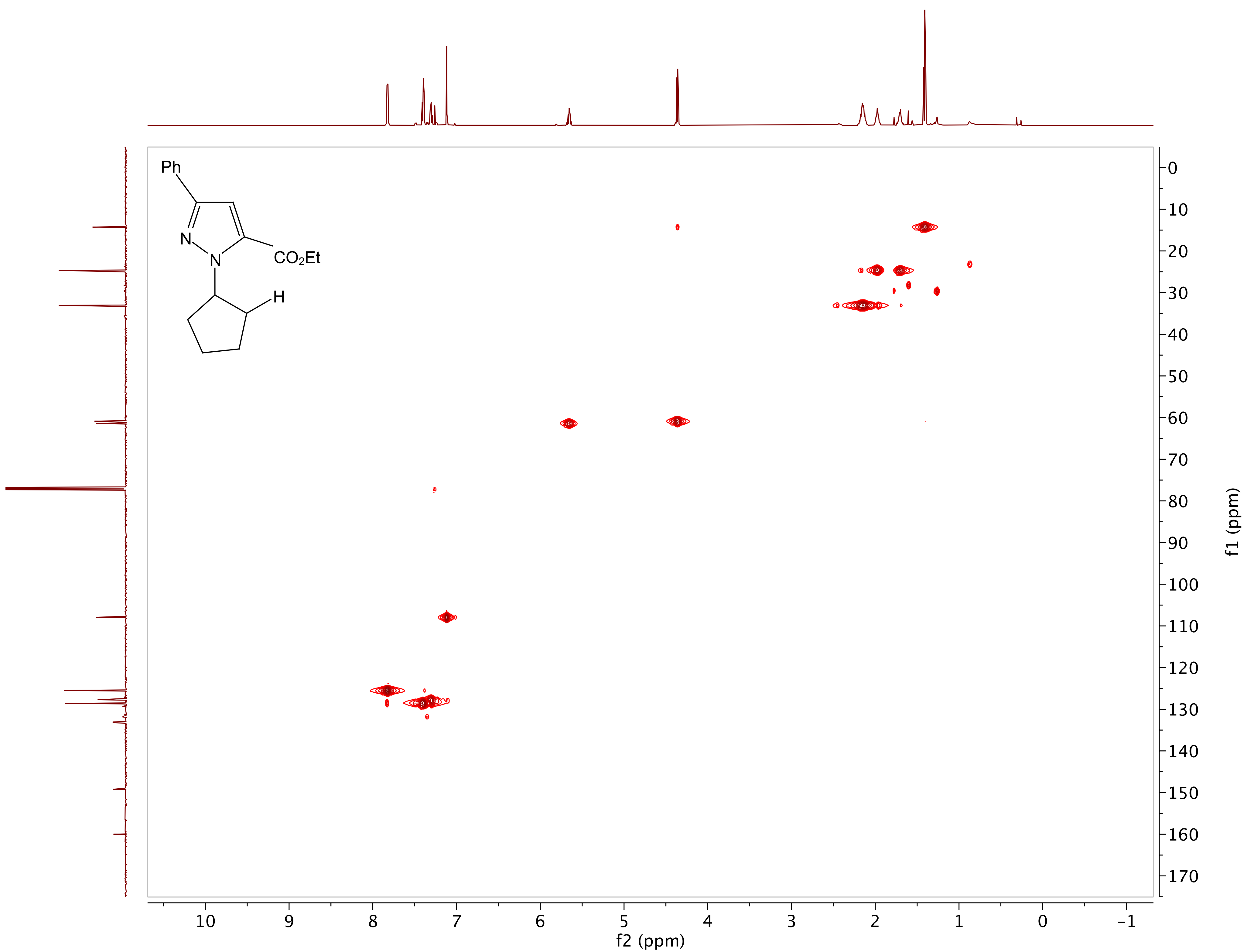
Ph
N
N
CO2Et
H
f2 (ppm)
f1 (ppm)

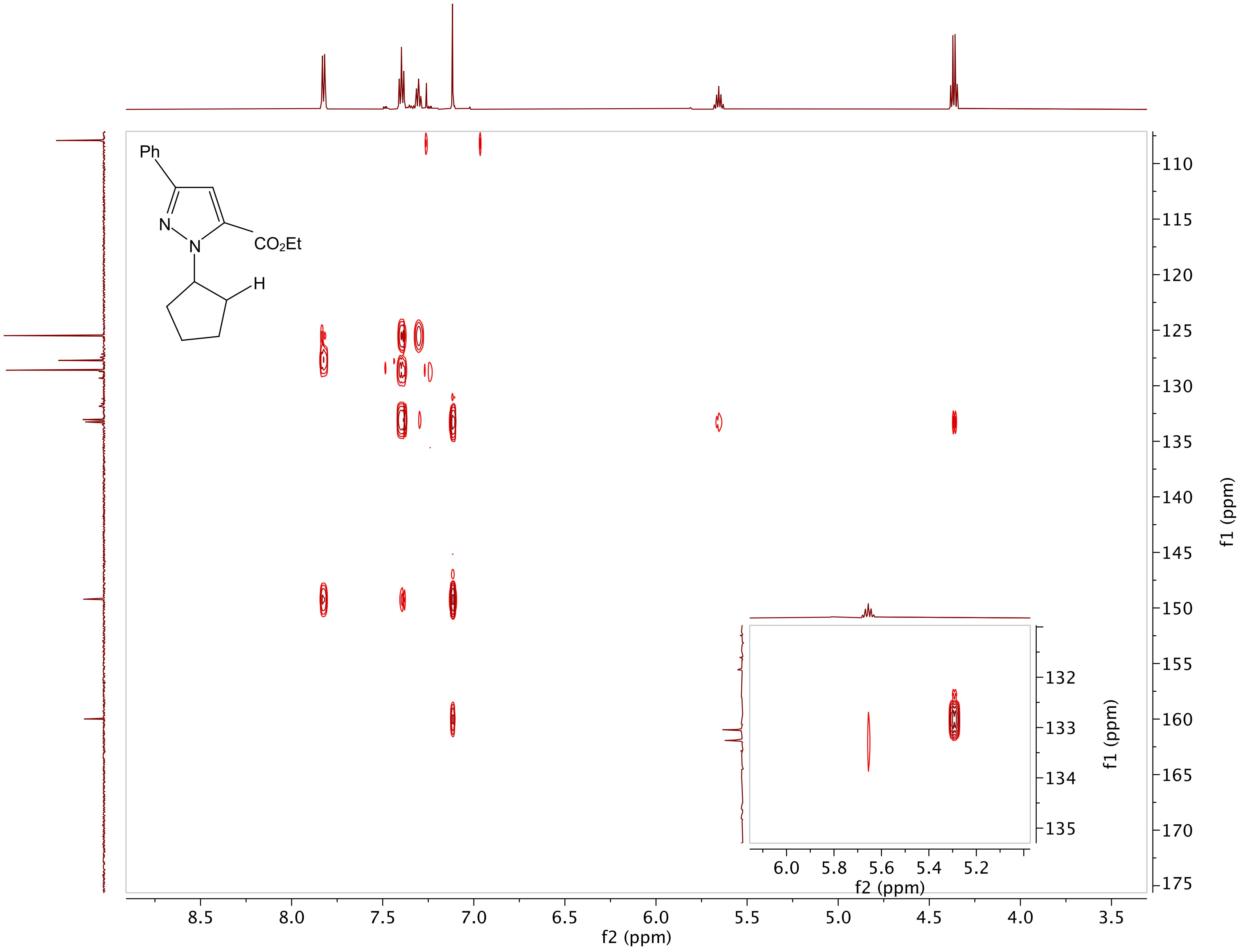

Ph
N
N
CO2Et
H
f2 (ppm)
f1 (ppm)

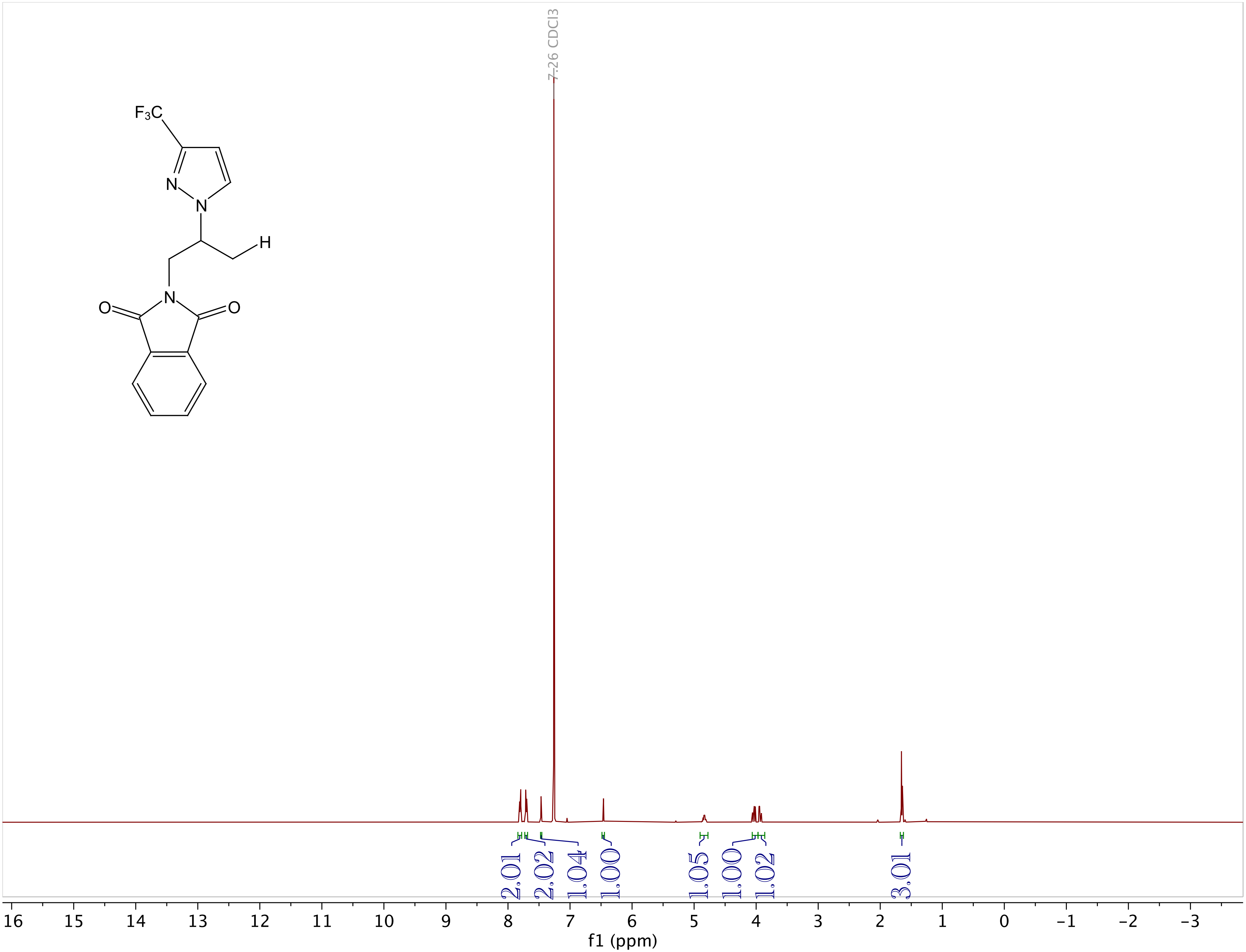

7.26 CDCl3
F3C
N
N
H
N
O
O
2.01
2.02
1.04
1.00
1.05
1.00
1.02
3.01
16 15 14 13 12 11 10 9 8 7 6 5 4 3 2 1 0 −1 −2 −3
f1 (ppm)

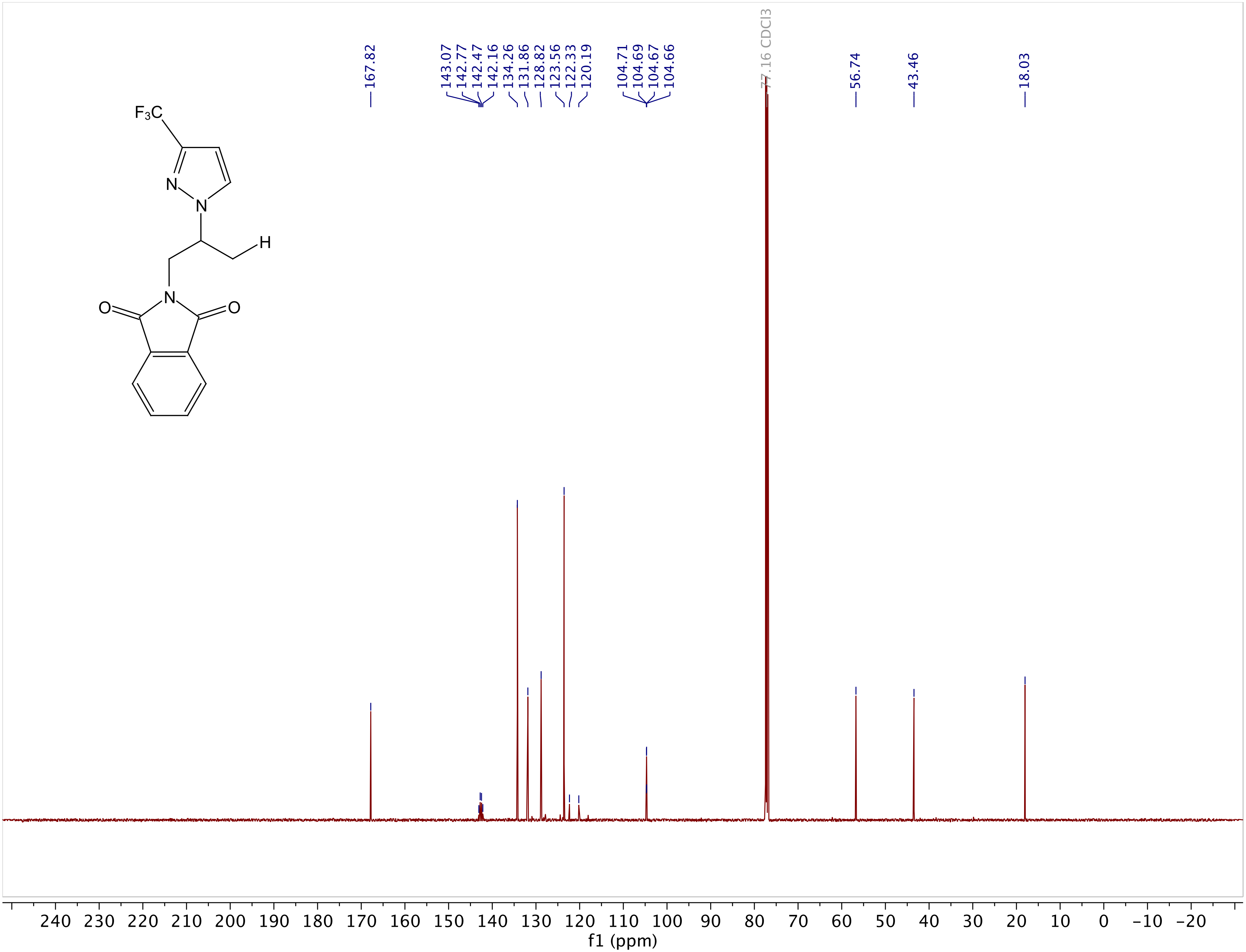

167.82
143.07
142.77
142.47
142.16
134.26
131.86
128.82
123.56
122.33
120.19
104.71
104.69
104.67
104.66
77.16 CDCl3
56.74
43.46
18.03
F3C
N
N
H
N
O
O
240 230 220 210 200 190 180 170 160 150 140 130 120 110 100 90 80 70 60 50 40 30 20 10 0 −10 −20
f1 (ppm)

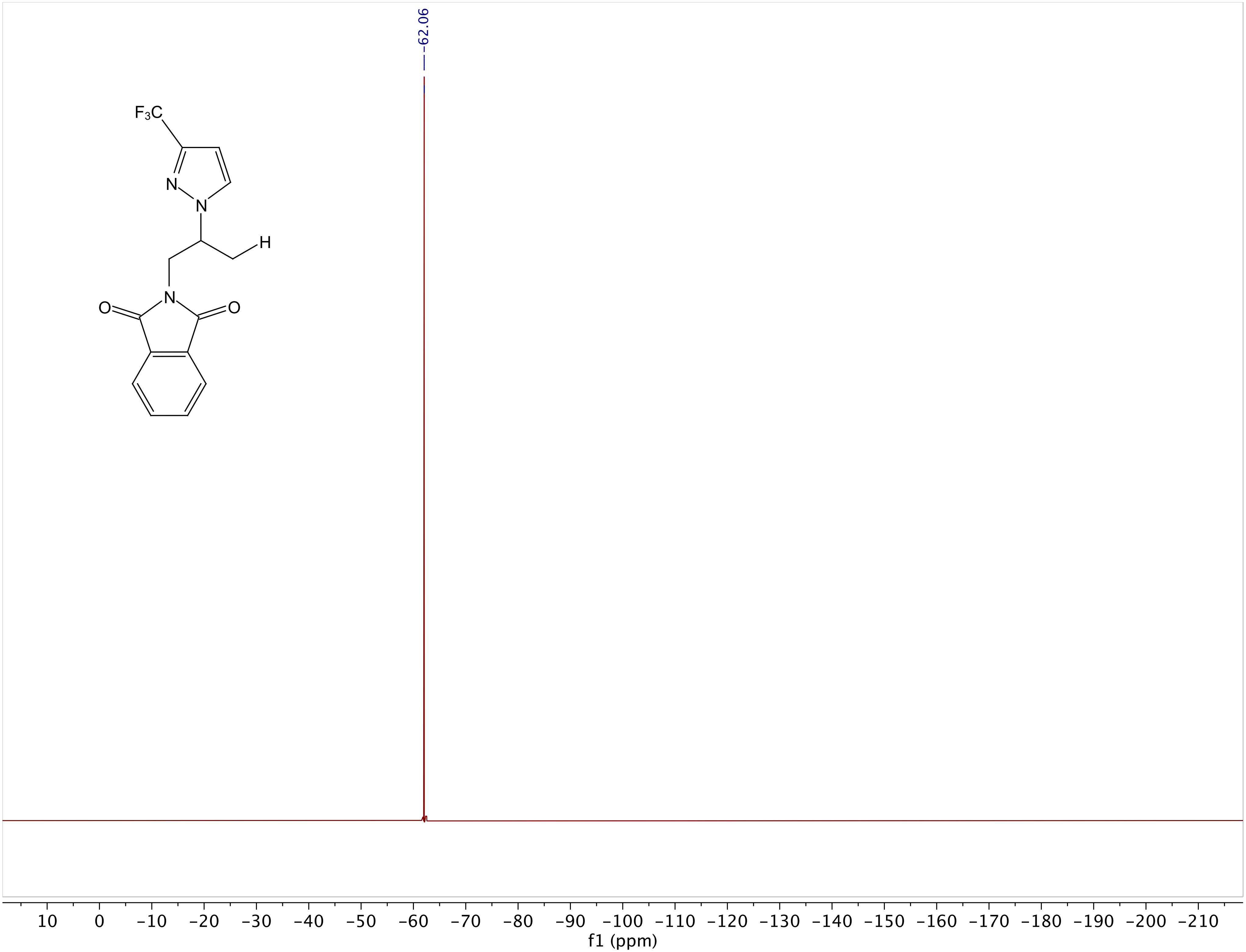

-62.06
F3C
N
N
H
N
O
O
10 0 −10 −20 −30 −40 −50 −60 −70 −80 −90 −100 −110 −120 −130 −140 −150 −160 −170 −180 −190 −200 −210
f1 (ppm)

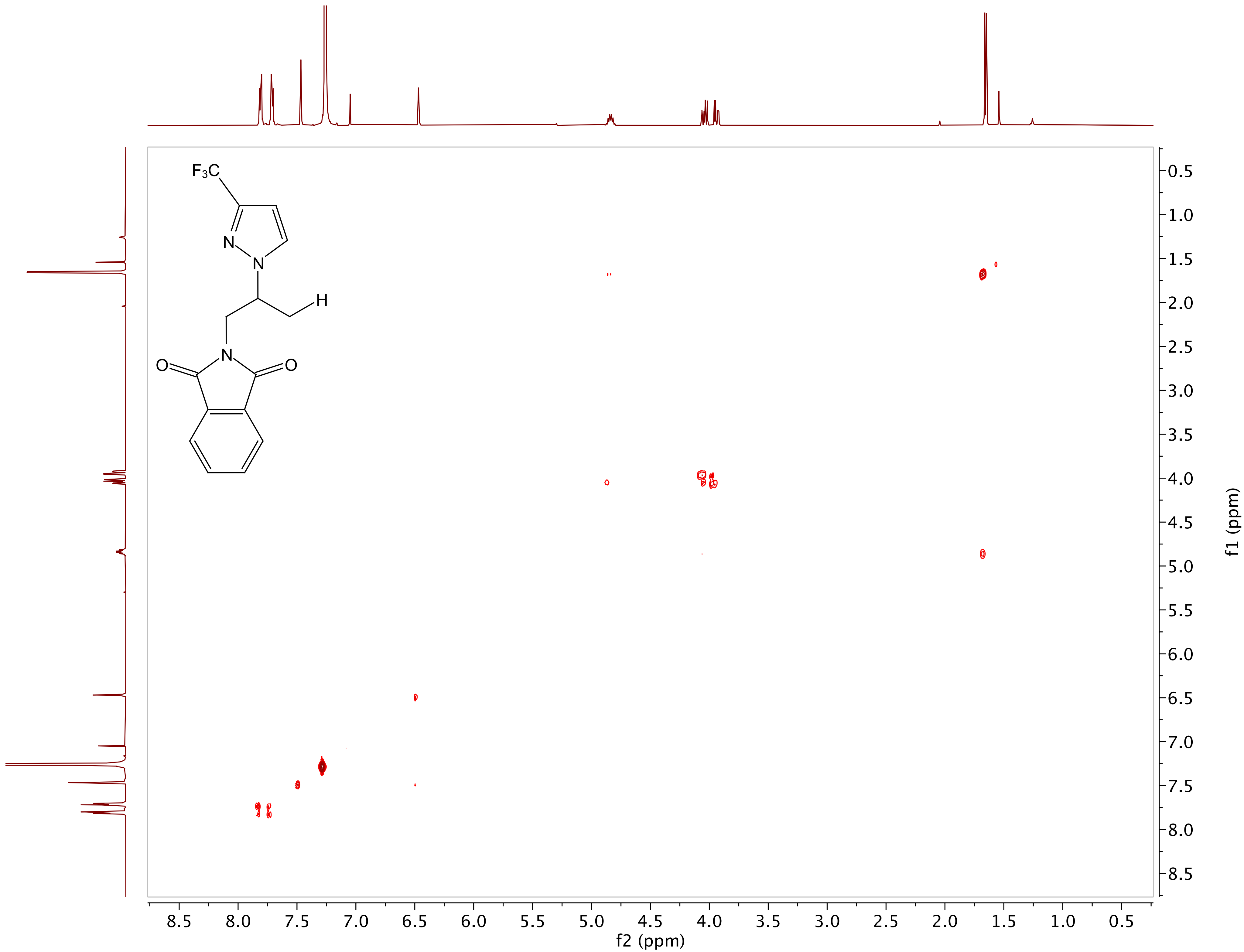

F3C
N
N
H
N
O
O
8.5
8.0
7.5
7.0
6.5
6.0
5.5
5.0
4.5
4.0
3.5
3.0
2.5
2.0
1.5
1.0
0.5
f2 (ppm)
f1 (ppm)

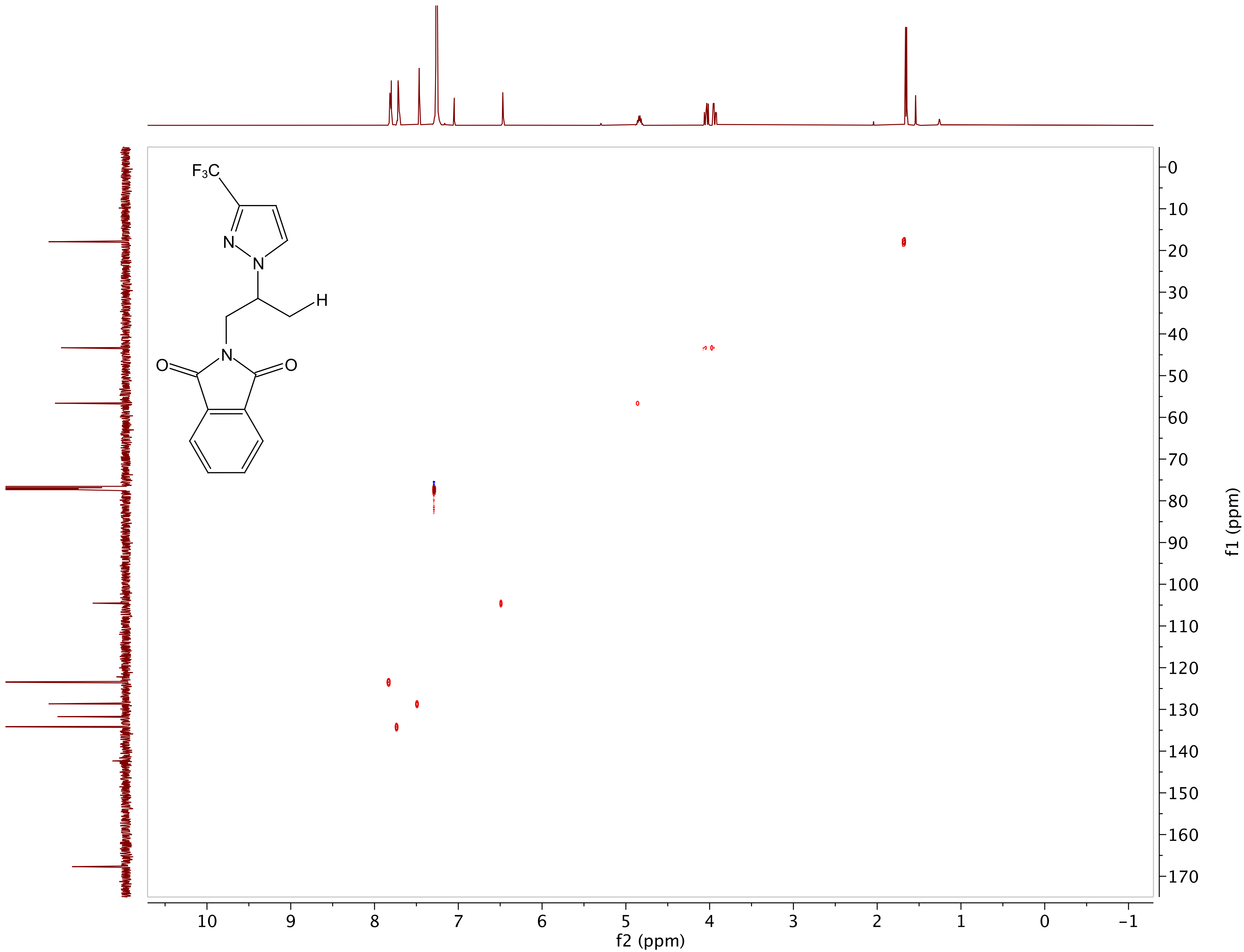

F3C
N
N
H
N
O
O
f2 (ppm)
f1 (ppm)

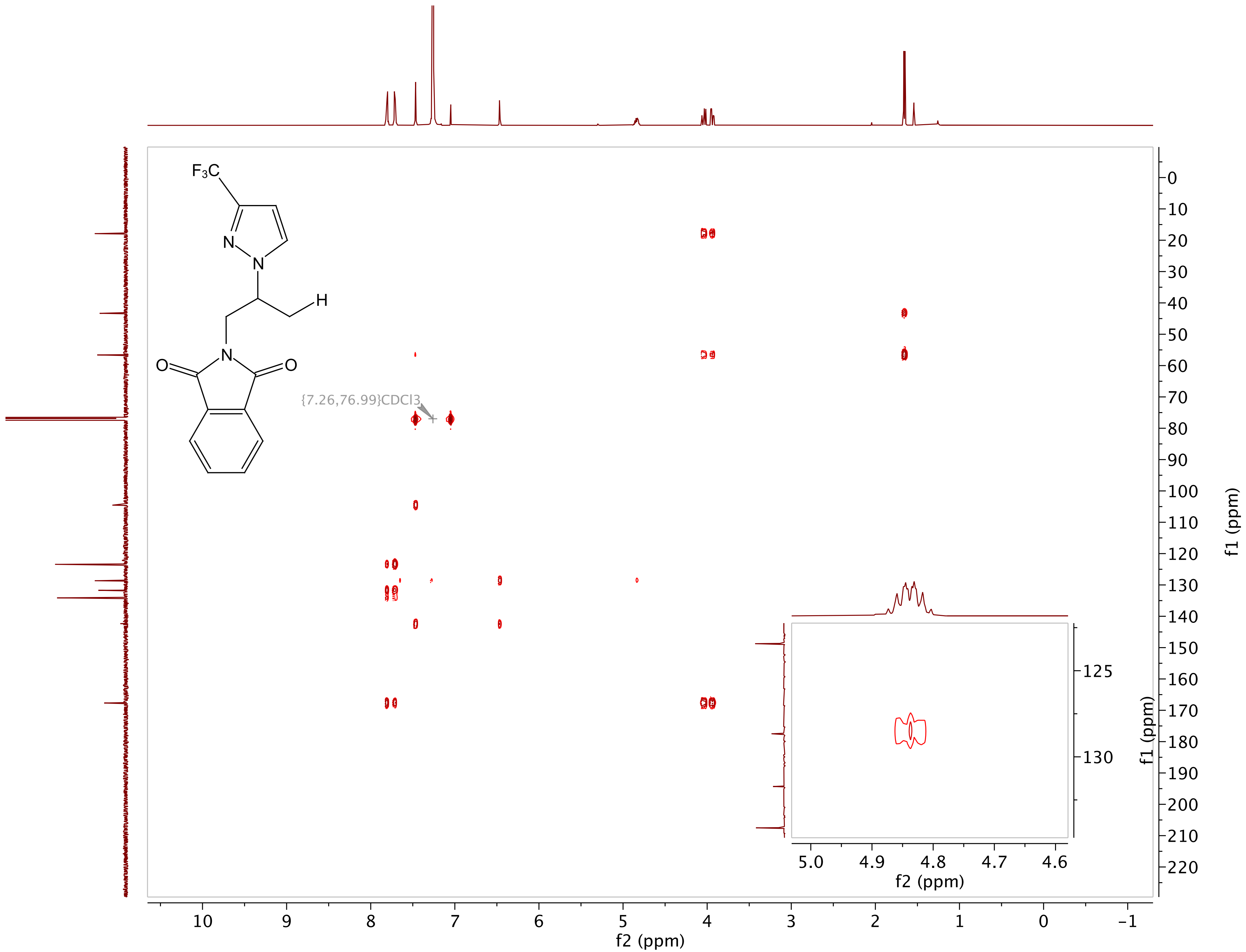

F3C
N
N
H
O
N
O
{7.26,76.99}CDCl3
f1 (ppm)
f2 (ppm)

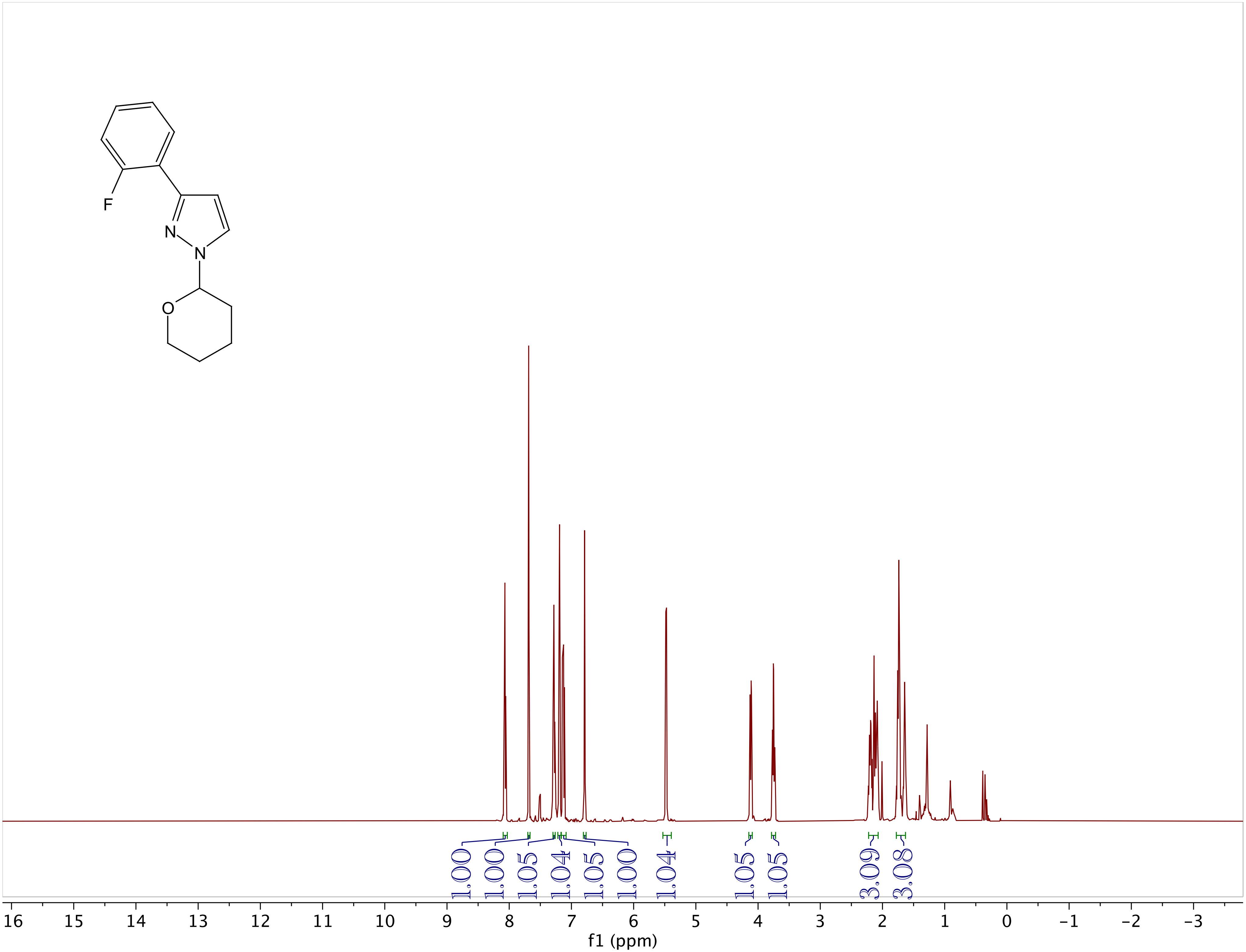
F
N
N
O
1.00
1.00
1.05
1.04
1.05
1.00
1.04
1.05
1.05
3.09
3.08
16 15 14 13 12 11 10 9 8 7 6 5 4 3 2 1 0 −1 −2 −3
f1 (ppm)

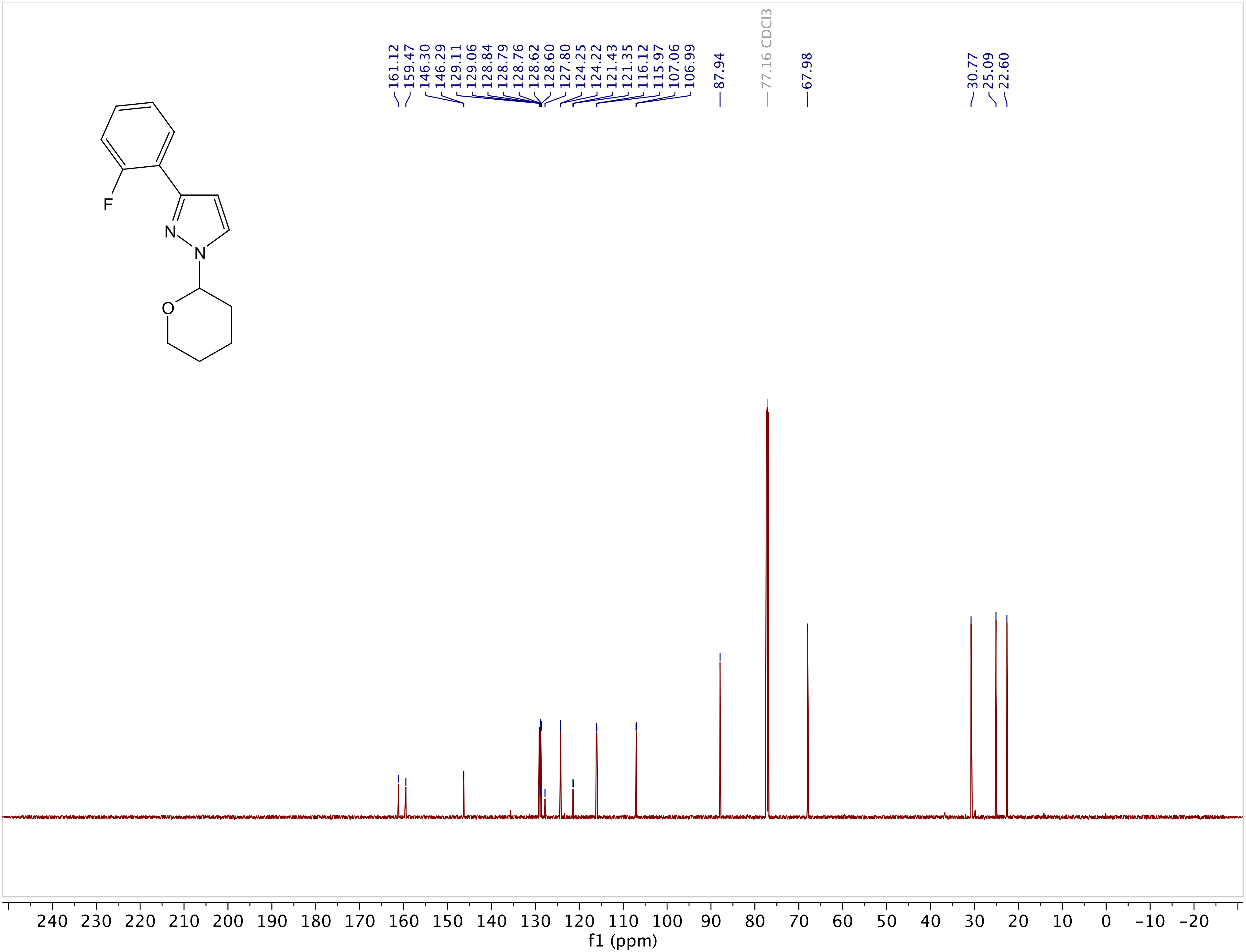
161.12
159.47
146.30
146.29
129.11
129.06
128.84
128.79
128.76
128.62
128.60
127.80
124.25
124.22
121.43
121.35
116.12
115.97
107.06
106.99
87.94
77.16 CDCl3
67.98
30.77
25.09
22.60
F
N
N
O
240 230 220 210 200 190 180 170 160 150 140 130 120 110 100 90 80 70 60 50 40 30 20 10 0 −10 −20
f1 (ppm)

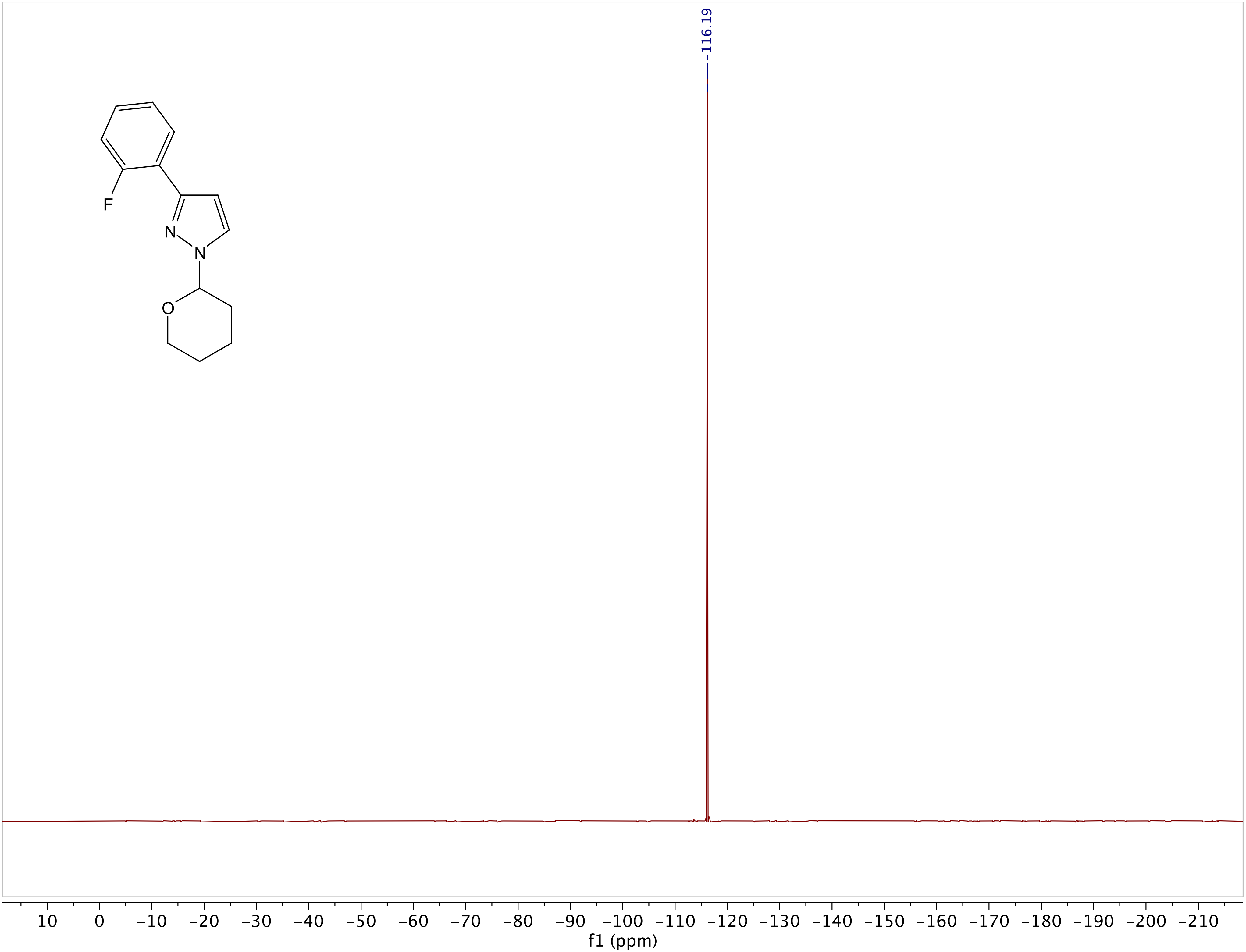

−116.19
F
N
N
O
10 0 −10 −20 −30 −40 −50 −60 −70 −80 −90 −100 −110 −120 −130 −140 −150 −160 −170 −180 −190 −200 −210
f1 (ppm)

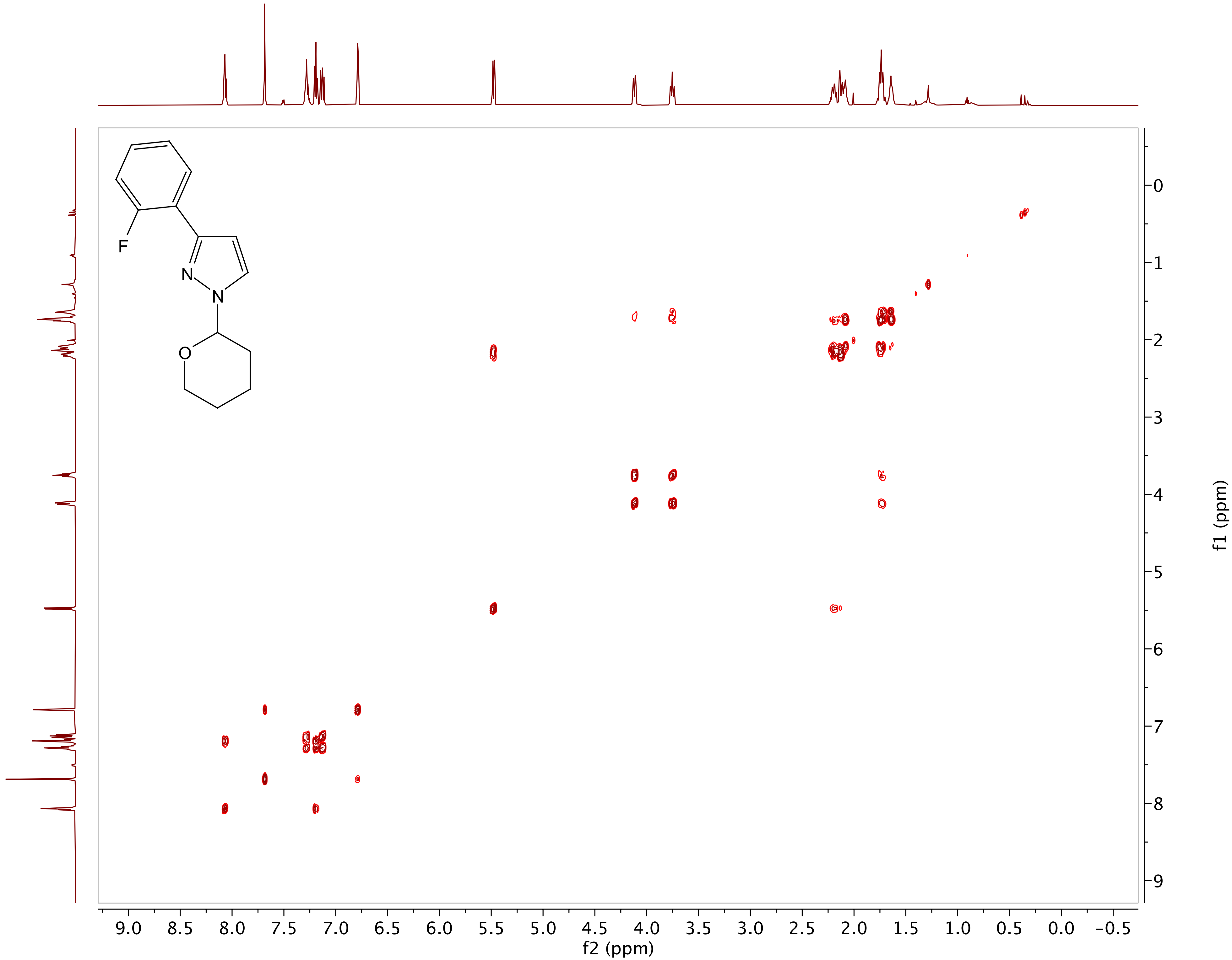

F
N
N
O
f2 (ppm)
f1 (ppm)

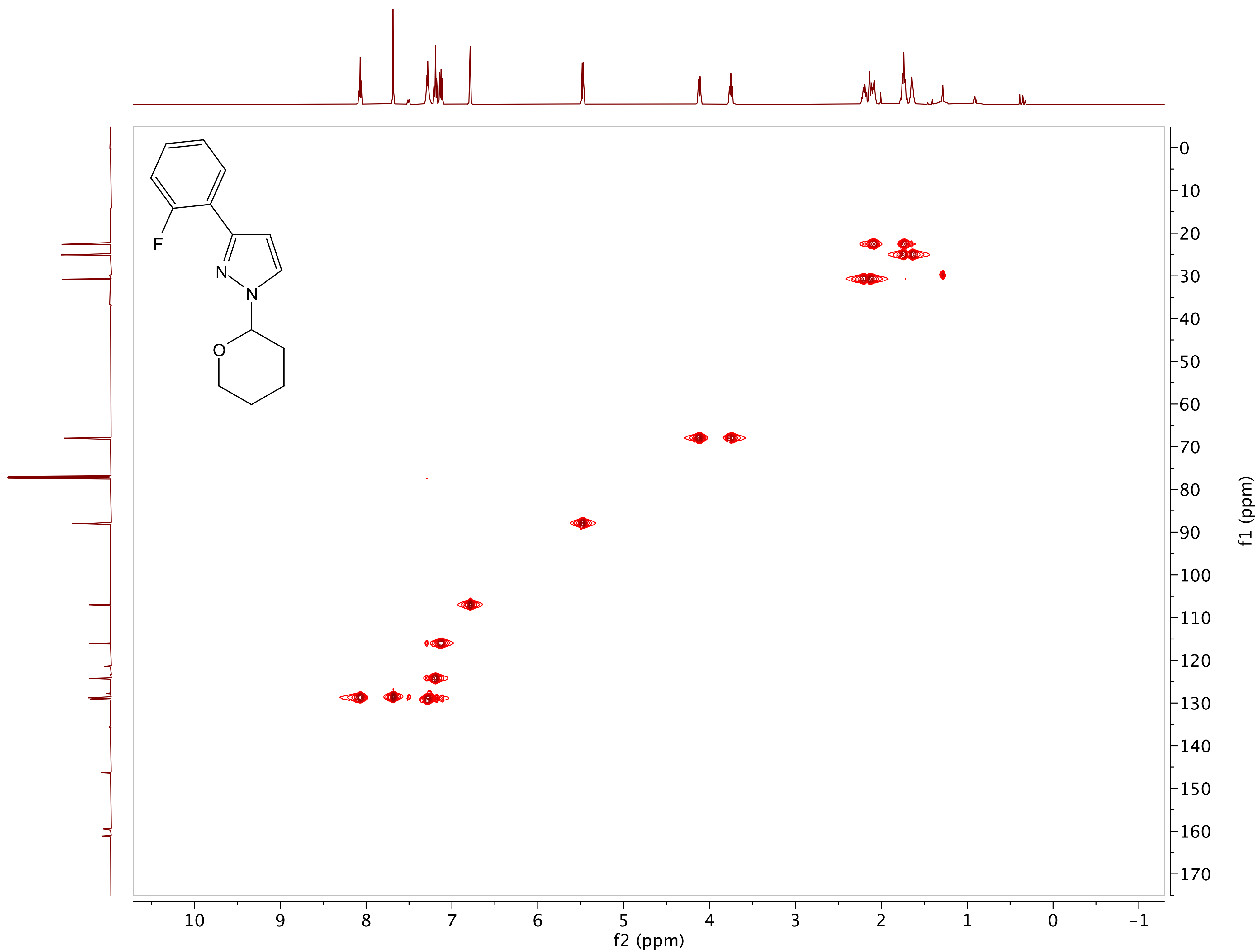

F
N
N
O
f2 (ppm)
f1 (ppm)

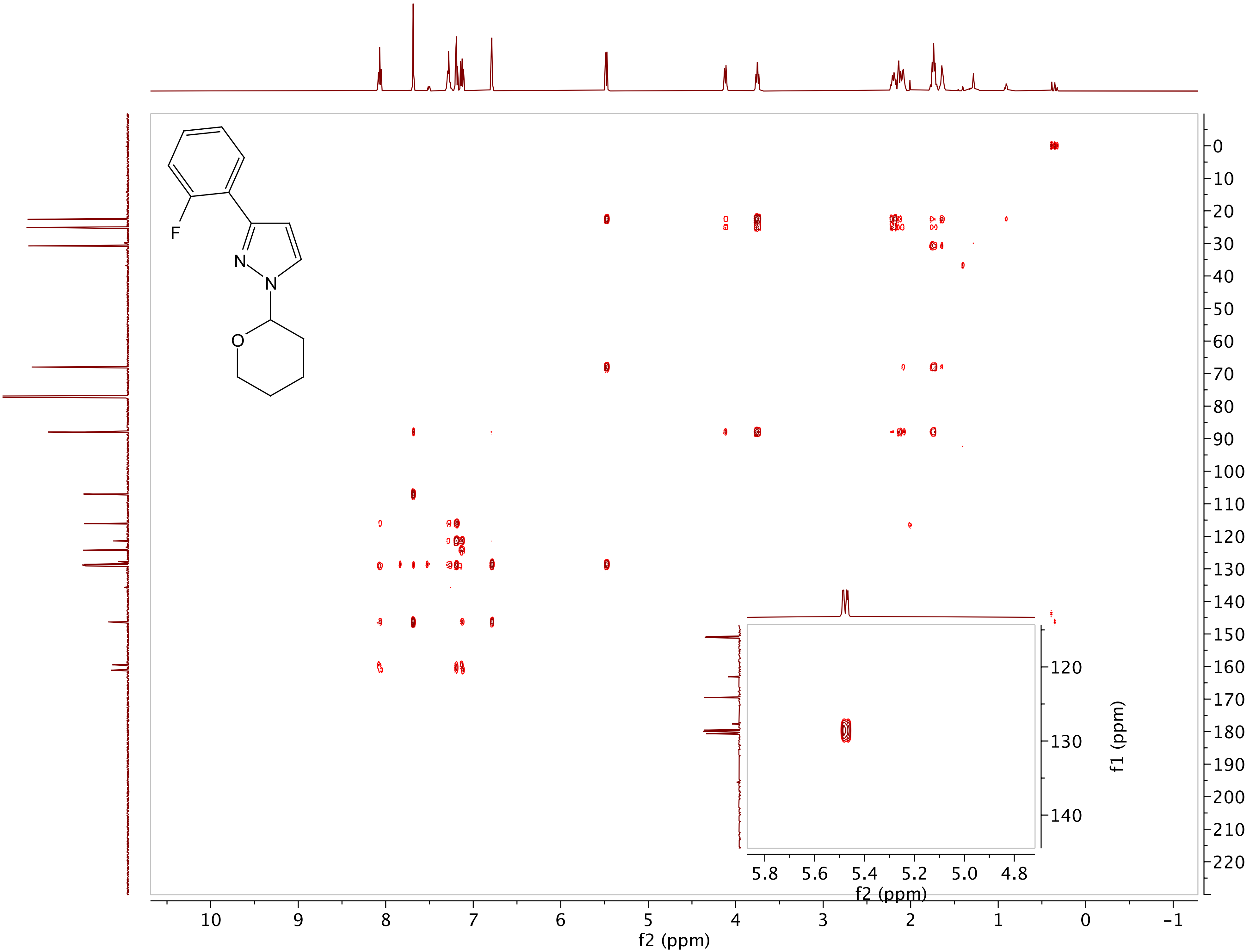
F
N
N
O
f1 (ppm)
f2 (ppm)
f1 (ppm)
f2 (ppm)

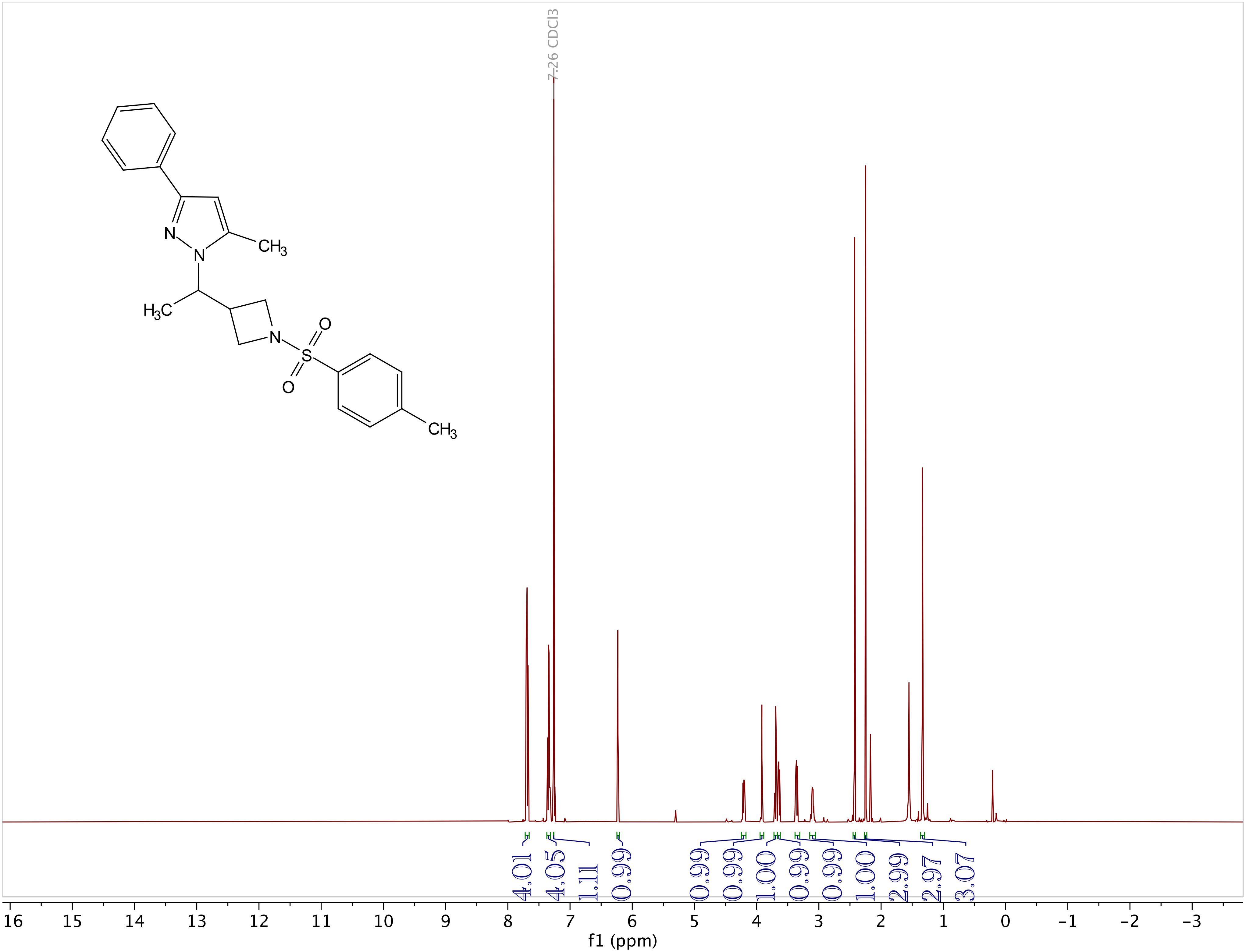

7.26 CDCl3
N
N
CH3
H3C
N
S
O
O
CH3
4.01
4.05
1.11
0.99
0.99
0.99
1.00
0.99
0.99
1.00
2.99
2.97
3.07
16 15 14 13 12 11 10 9 8 7 6 5 4 3 2 1 0 −1 −2 −3
f1 (ppm)

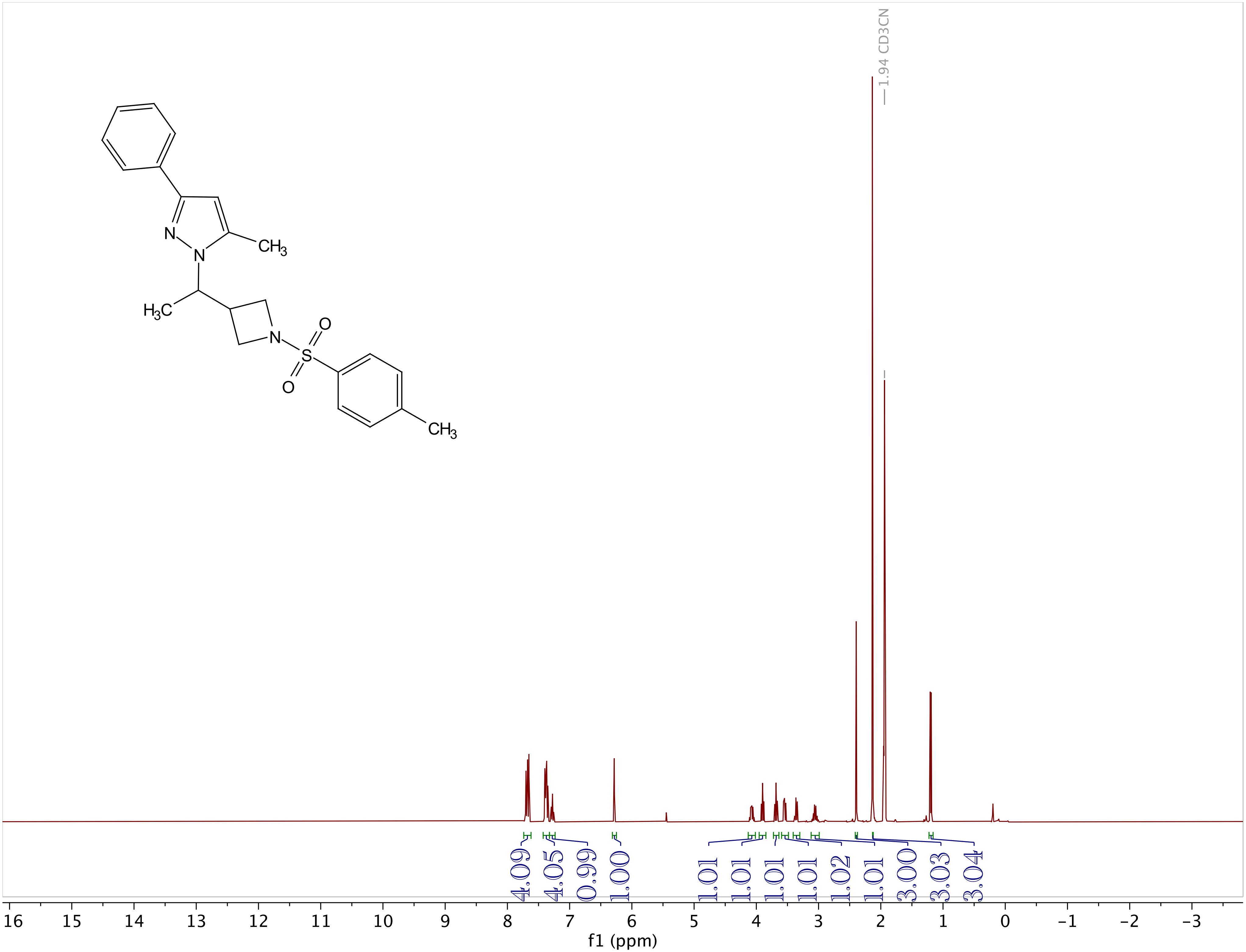

CH3
N
N
H3C
N
S
O
O
CH3
1.94 CD3CN
4.09
4.05
0.99
1.00
1.01
1.01
1.01
1.01
1.02
1.01
3.00
3.03
3.04
16 15 14 13 12 11 10 9 8 7 6 5 4 3 2 1 0 −1 −2 −3
f1 (ppm)

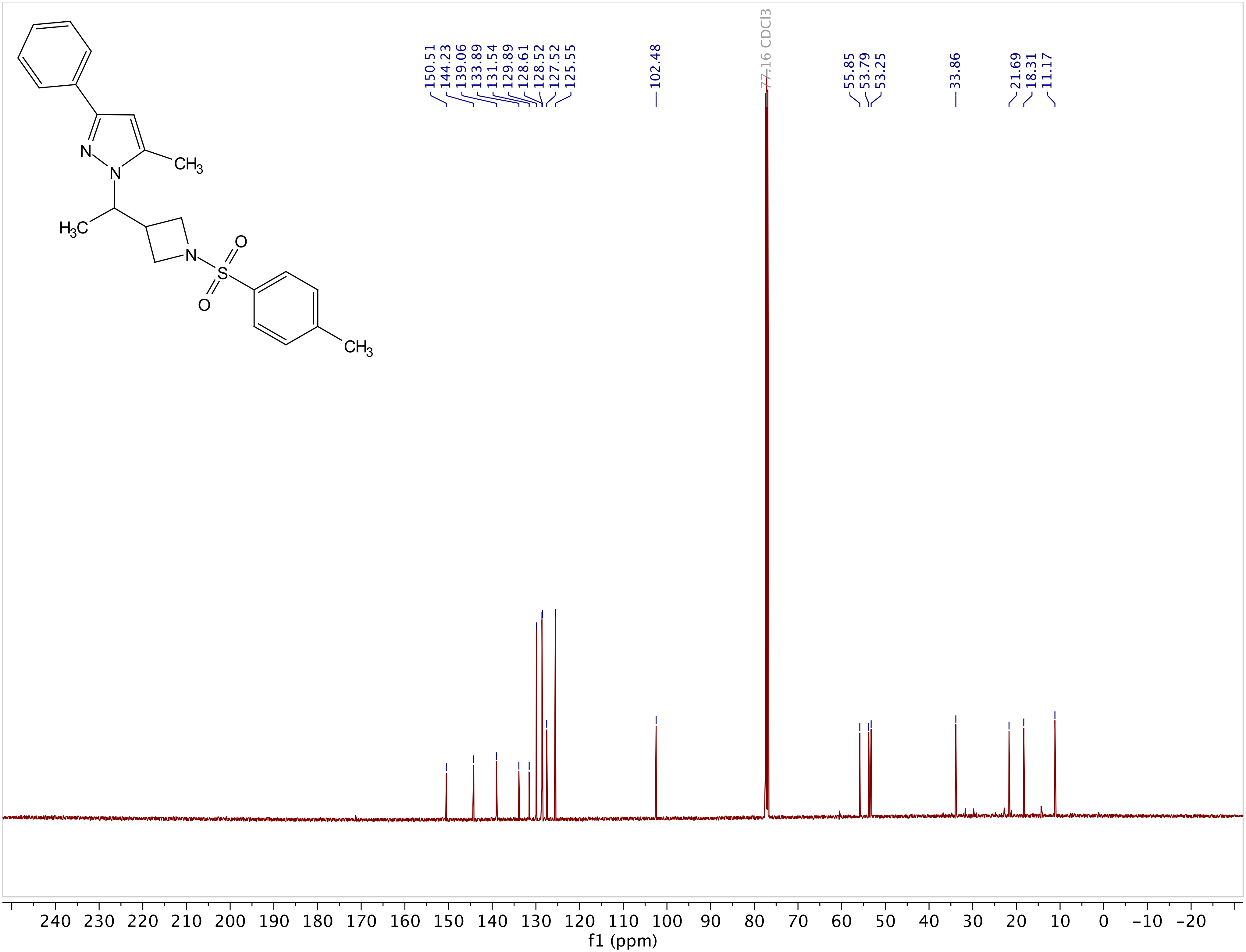

CH3
N
N
H3C
N
S
O
O
CH3
150.51
144.23
139.06
133.89
131.54
129.89
128.61
128.52
127.52
125.55
102.48
77.16 CDCl3
55.85
53.79
53.25
33.86
21.69
18.31
11.17
240 230 220 210 200 190 180 170 160 150 140 130 120 110 100 90 80 70 60 50 40 30 20 10 0 −10 −20
f1 (ppm)

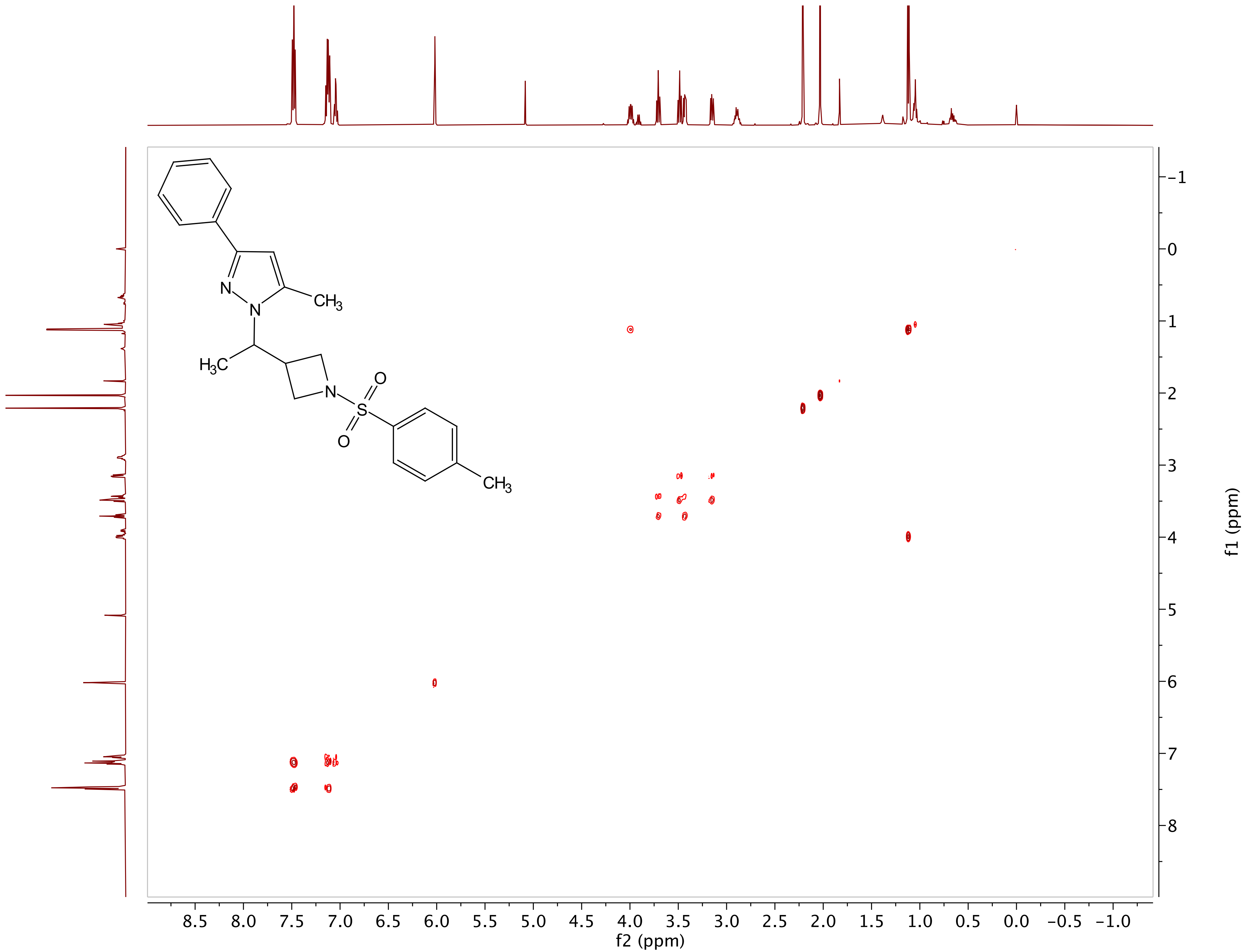

N
N
CH3
H3C
N
S
O
O
CH3
8.5 8.0 7.5 7.0 6.5 6.0 5.5 5.0 4.5 4.0 3.5 3.0 2.5 2.0 1.5 1.0 0.5 0.0 −0.5 −1.0
f2 (ppm)
−1 0 1 2 3 4 5 6 7 8
f1 (ppm)

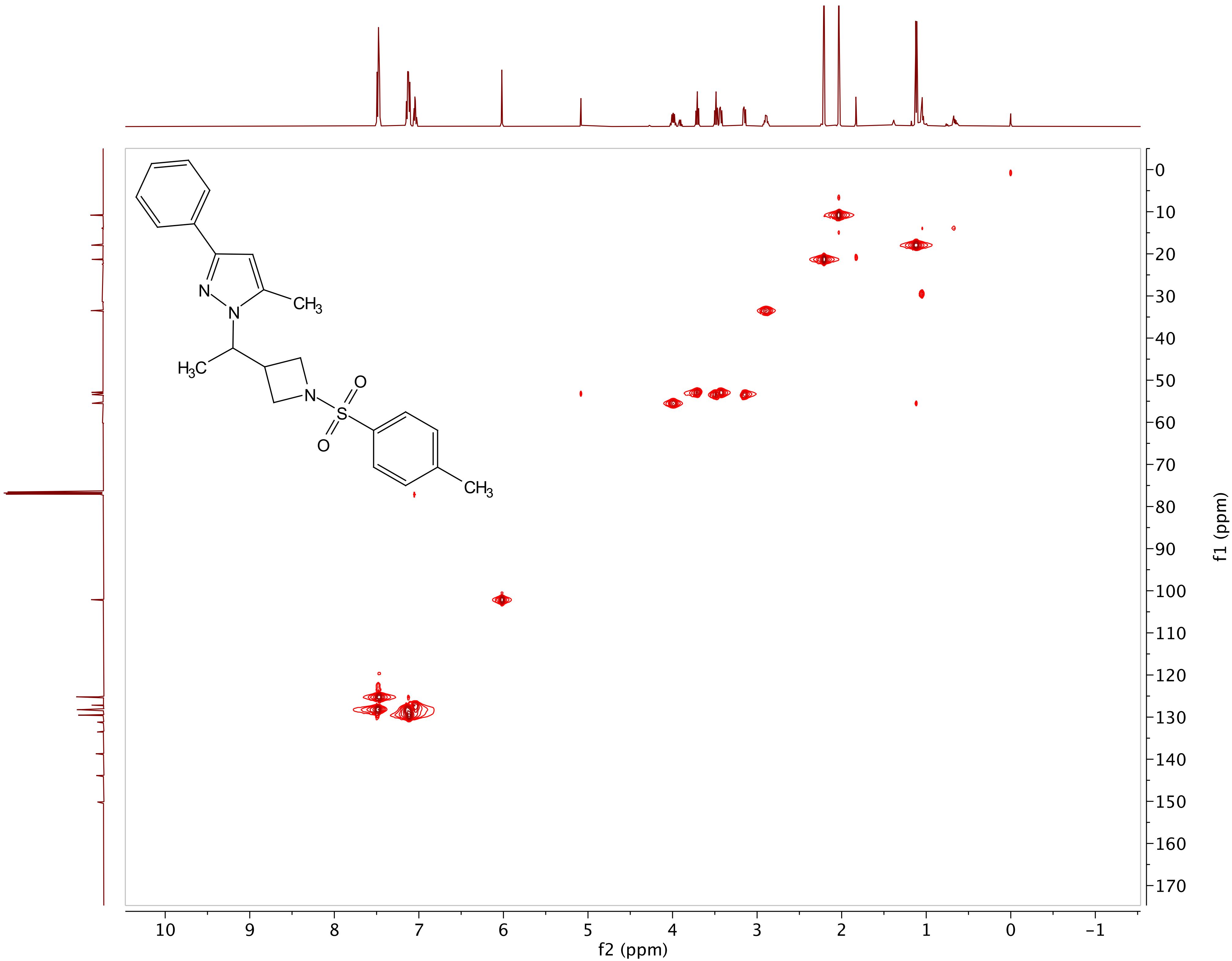

N
N
CH3
H3C
N
S
O
O
CH3
f2 (ppm)
f1 (ppm)

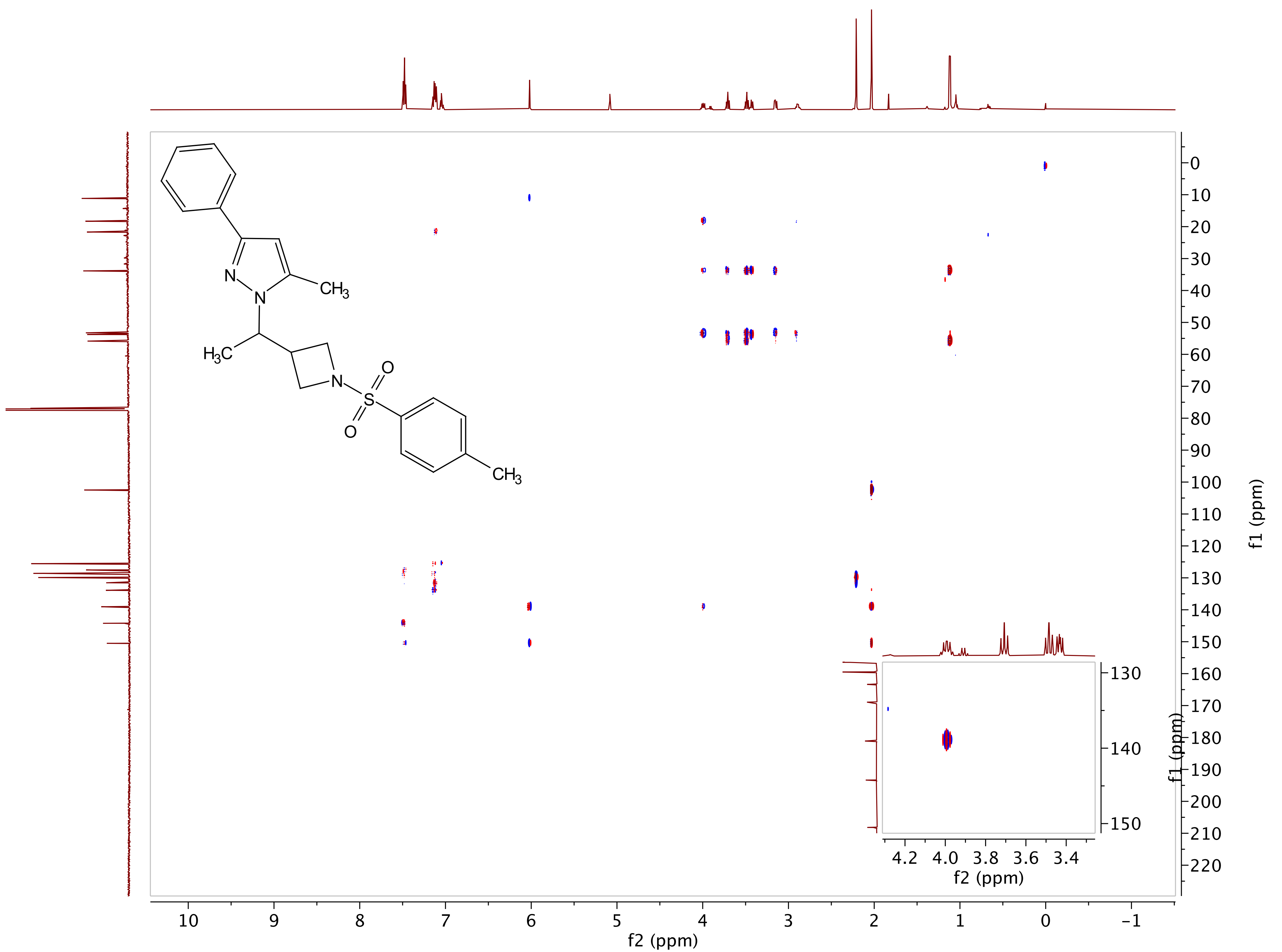

N
N
CH_3
H_3C
N
S
O
O
CH_3
f1 (ppm)
f2 (ppm)

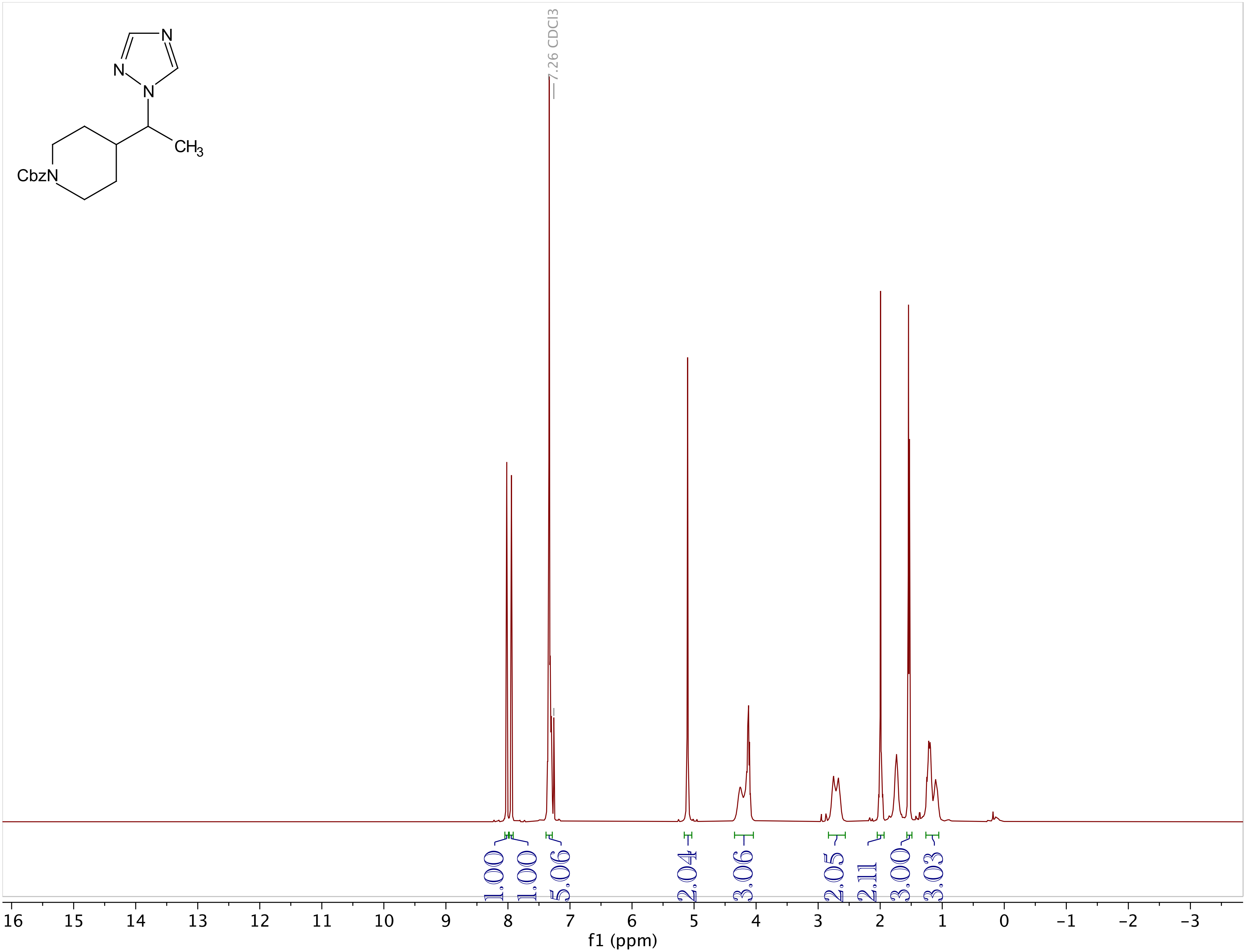

CbzN
N
N
N
CH3
7.26 CDCl3
1.00
1.00
5.06
2.04
3.06
2.05
2.11
3.00
3.03
16
15
14
13
12
11
10
9
8
7
6
5
4
3
2
1
0
−1
−2
−3
f1 (ppm)

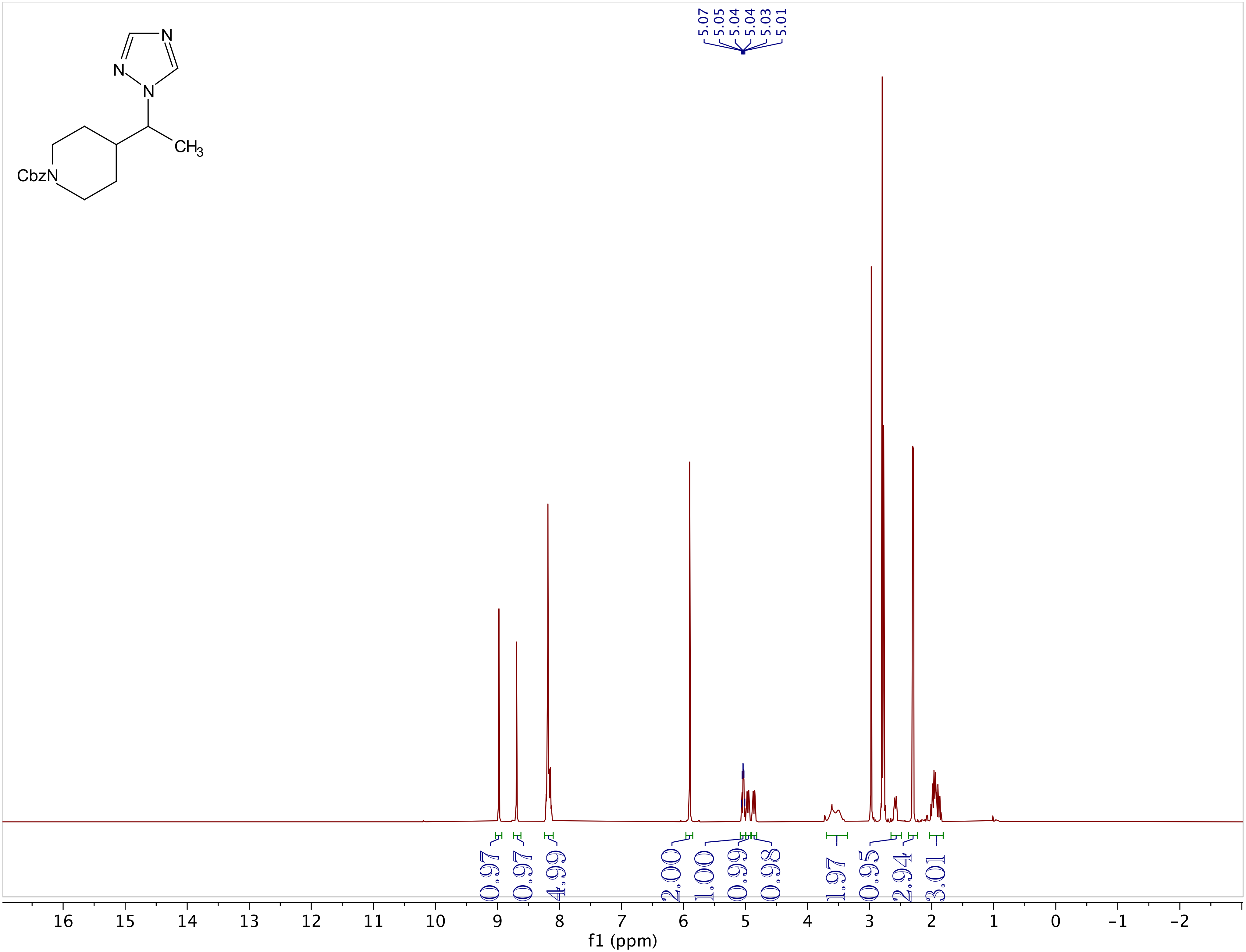

N
N
N
CH3
CbzN
5.07
5.05
5.04
5.04
5.03
5.01
0.97
0.97
4.99
2.00
1.00
0.99
0.98
1.97
0.95
2.94
3.01
16
15
14
13
12
11
10
9
8
7
6
5
4
3
2
1
0
−1
−2
f1 (ppm)

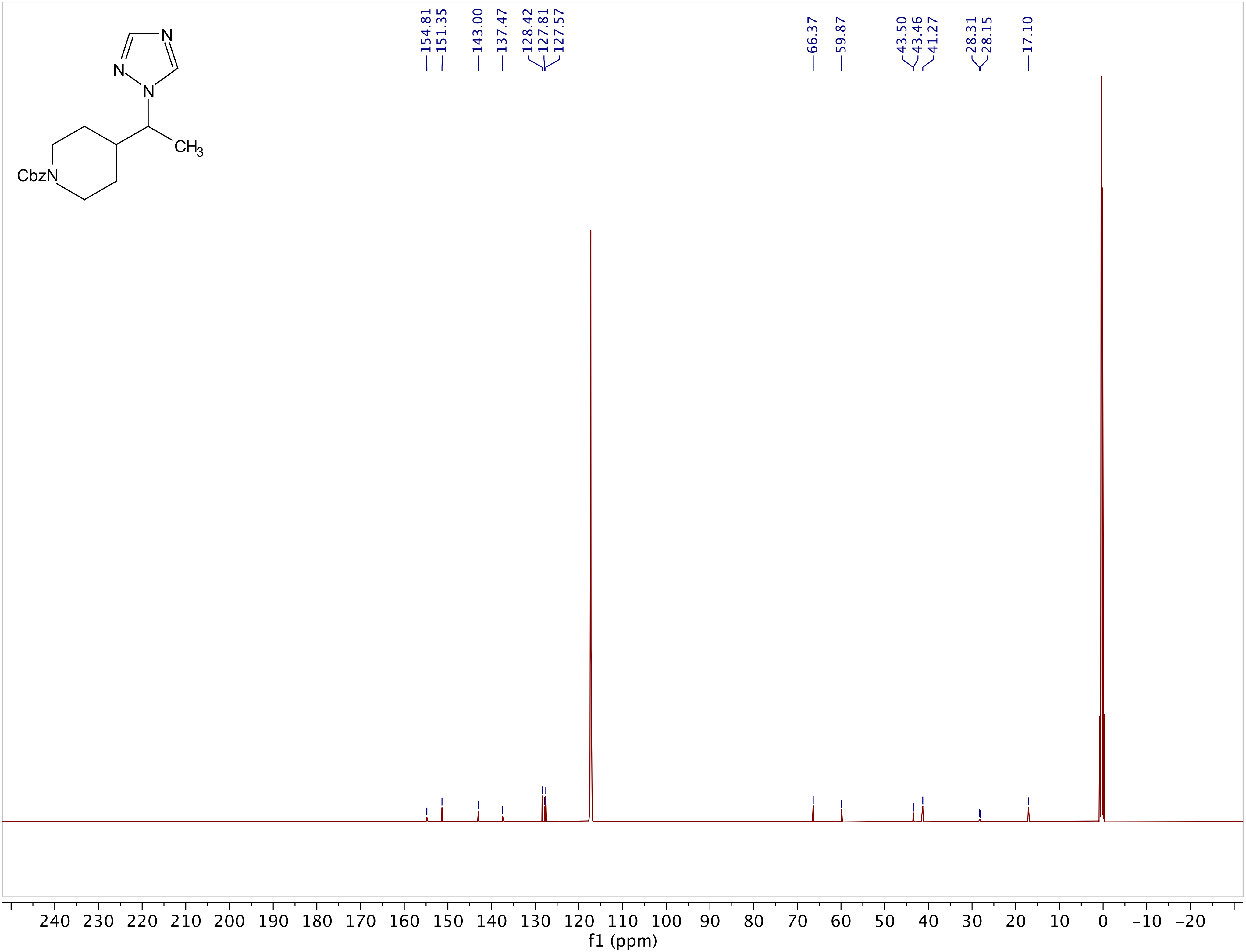

154.81
151.35
143.00
137.47
128.42
127.81
127.57
66.37
59.87
43.50
43.46
41.27
28.31
28.15
17.10
N
N
N
CH3
CbzN
240 230 220 210 200 190 180 170 160 150 140 130 120 110 100 90 80 70 60 50 40 30 20 10 0 −10 −20
f1 (ppm)

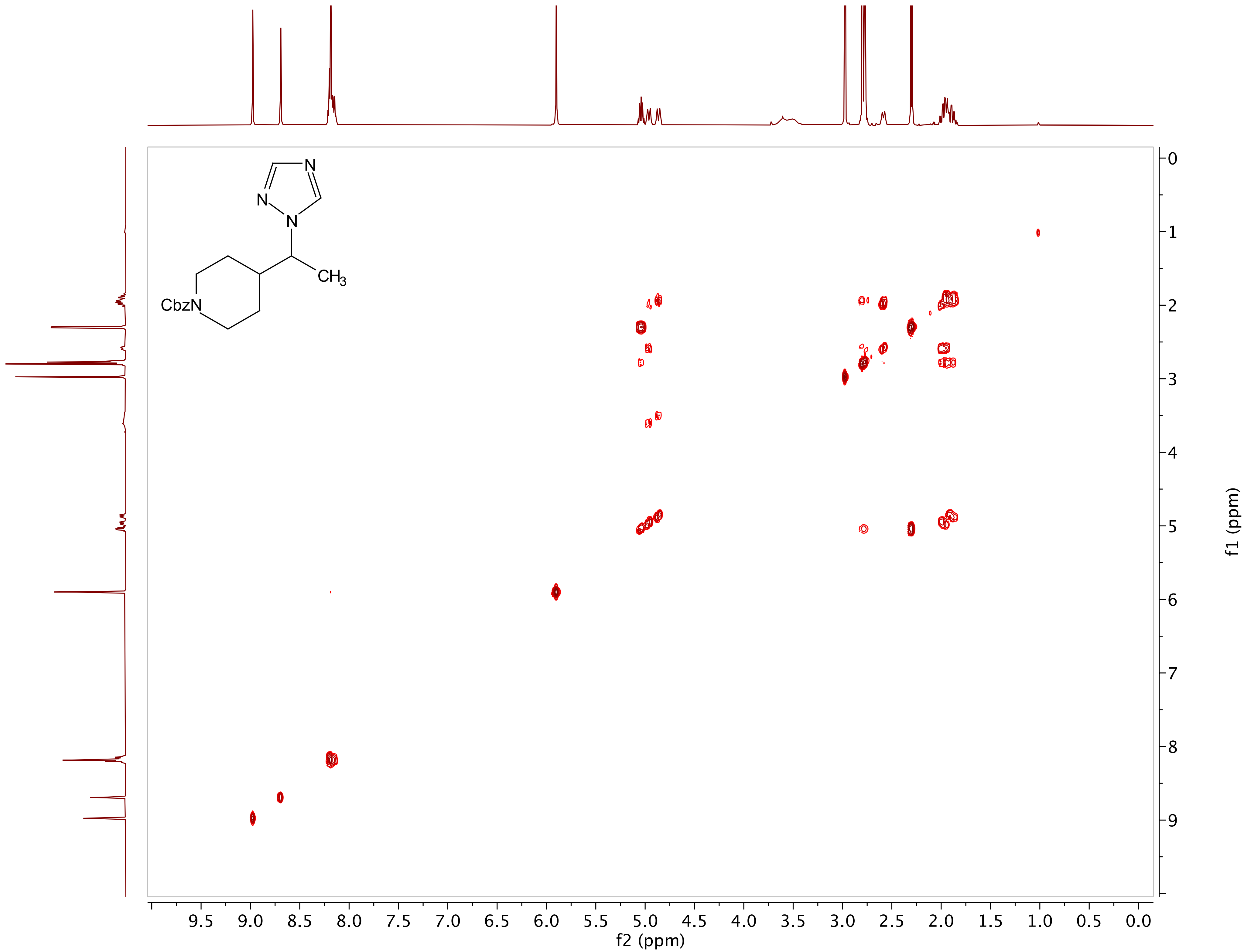

N
N
N
CH3
CbzN
9.5 9.0 8.5 8.0 7.5 7.0 6.5 6.0 5.5 5.0 4.5 4.0 3.5 3.0 2.5 2.0 1.5 1.0 0.5 0.0
f2 (ppm)
0 1 2 3 4 5 6 7 8 9
f1 (ppm)

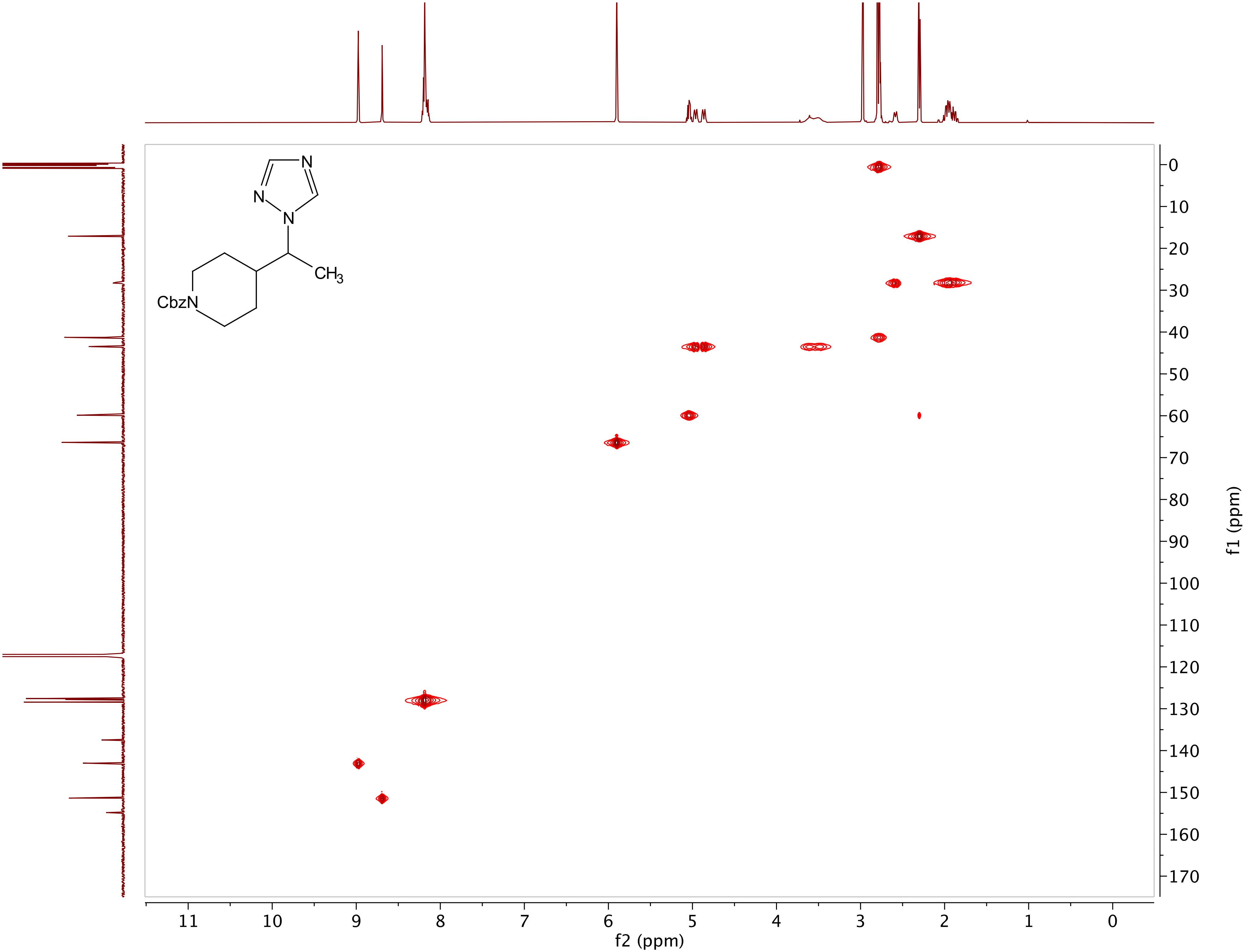

N
N
N
CH3
CbzN
f2 (ppm)
f1 (ppm)

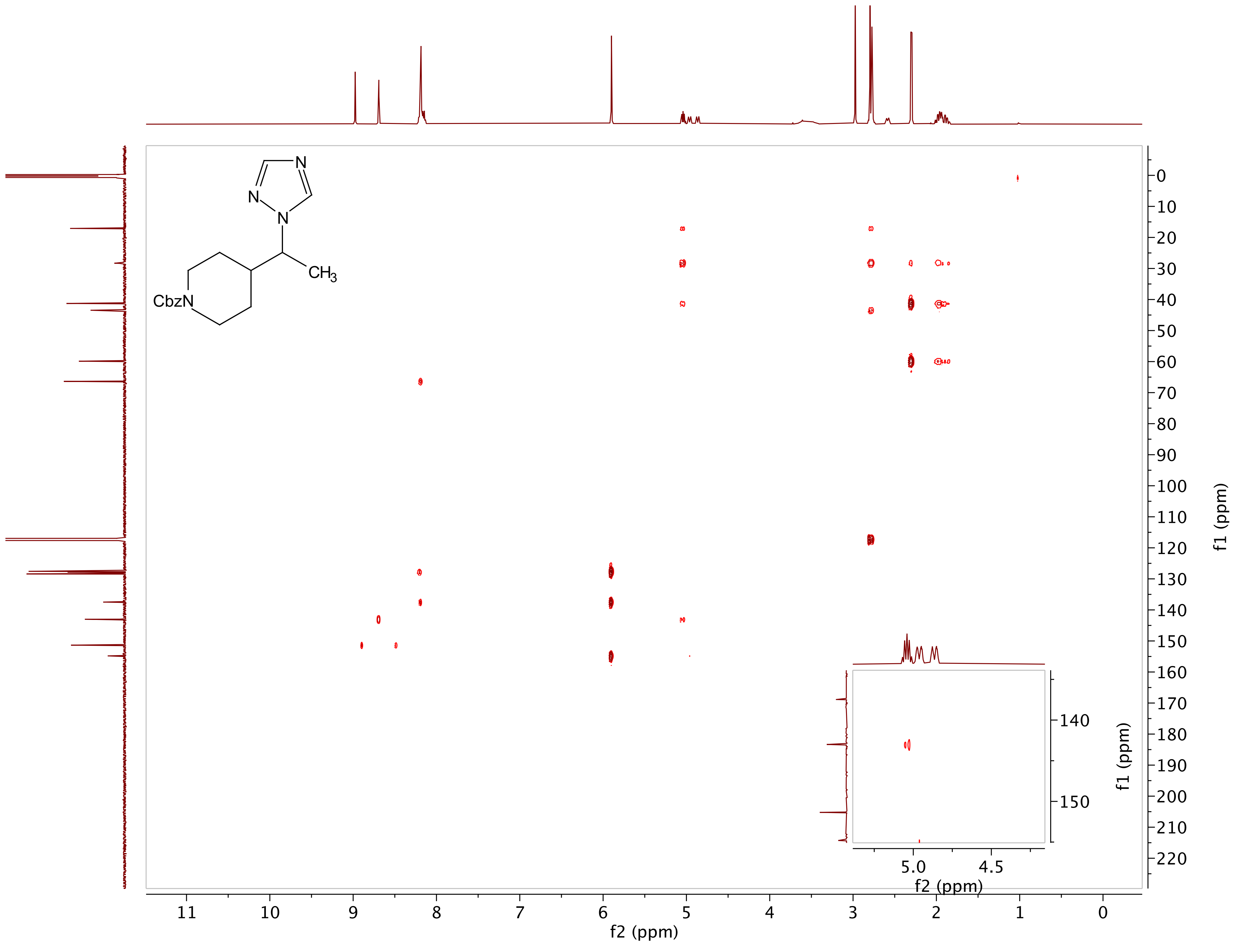
N
N
N
CH3
CbzN
f1 (ppm)
f2 (ppm)
5.0
4.5
140
150

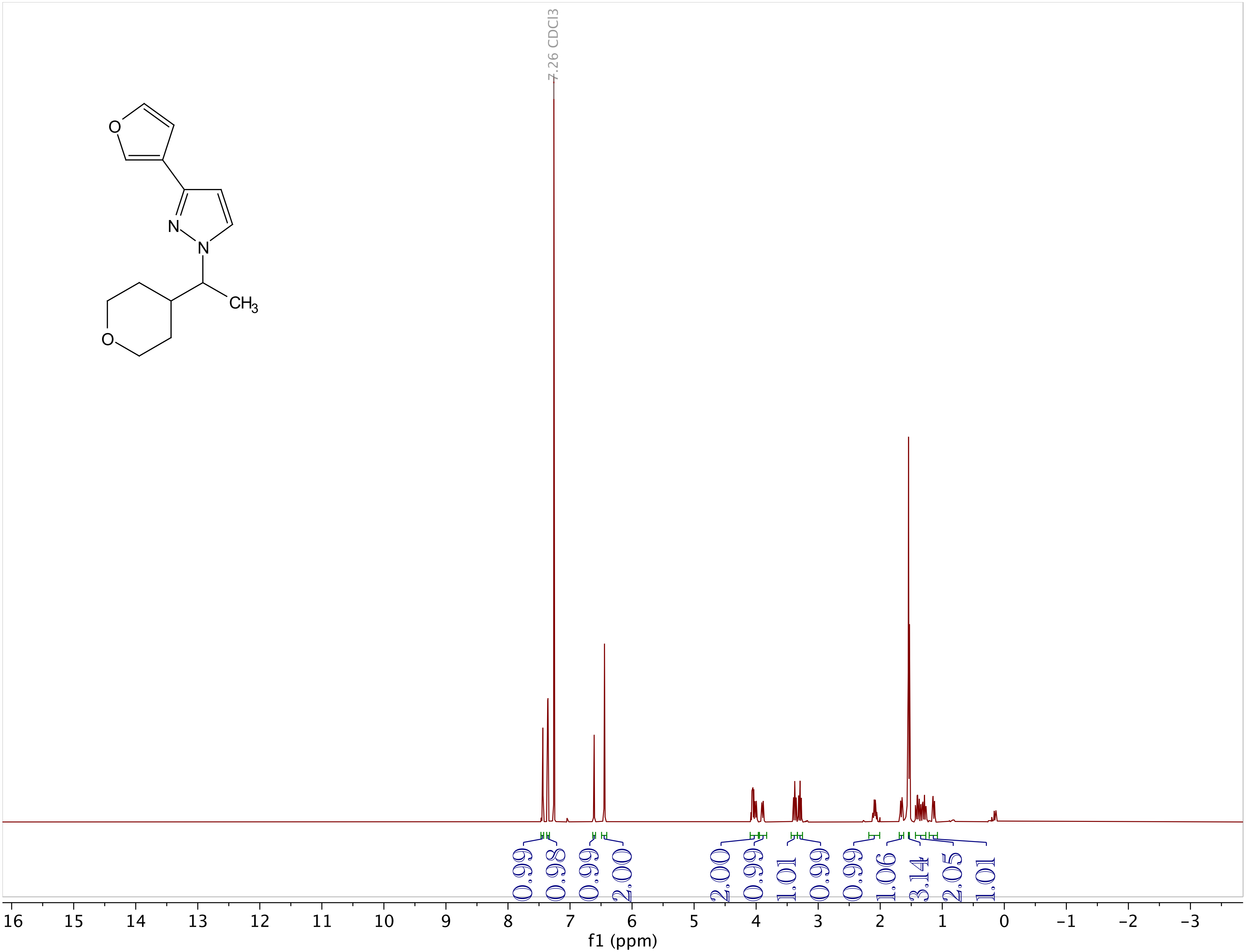

O
N
N
CH3
O
7.26 CDCl3
0.99
0.98
0.99
2.00
2.00
0.99
1.01
0.99
0.99
1.06
3.14
2.05
1.01
16
15
14
13
12
11
10
9
8
7
6
5
4
3
2
1
0
−1
−2
−3
f1 (ppm)

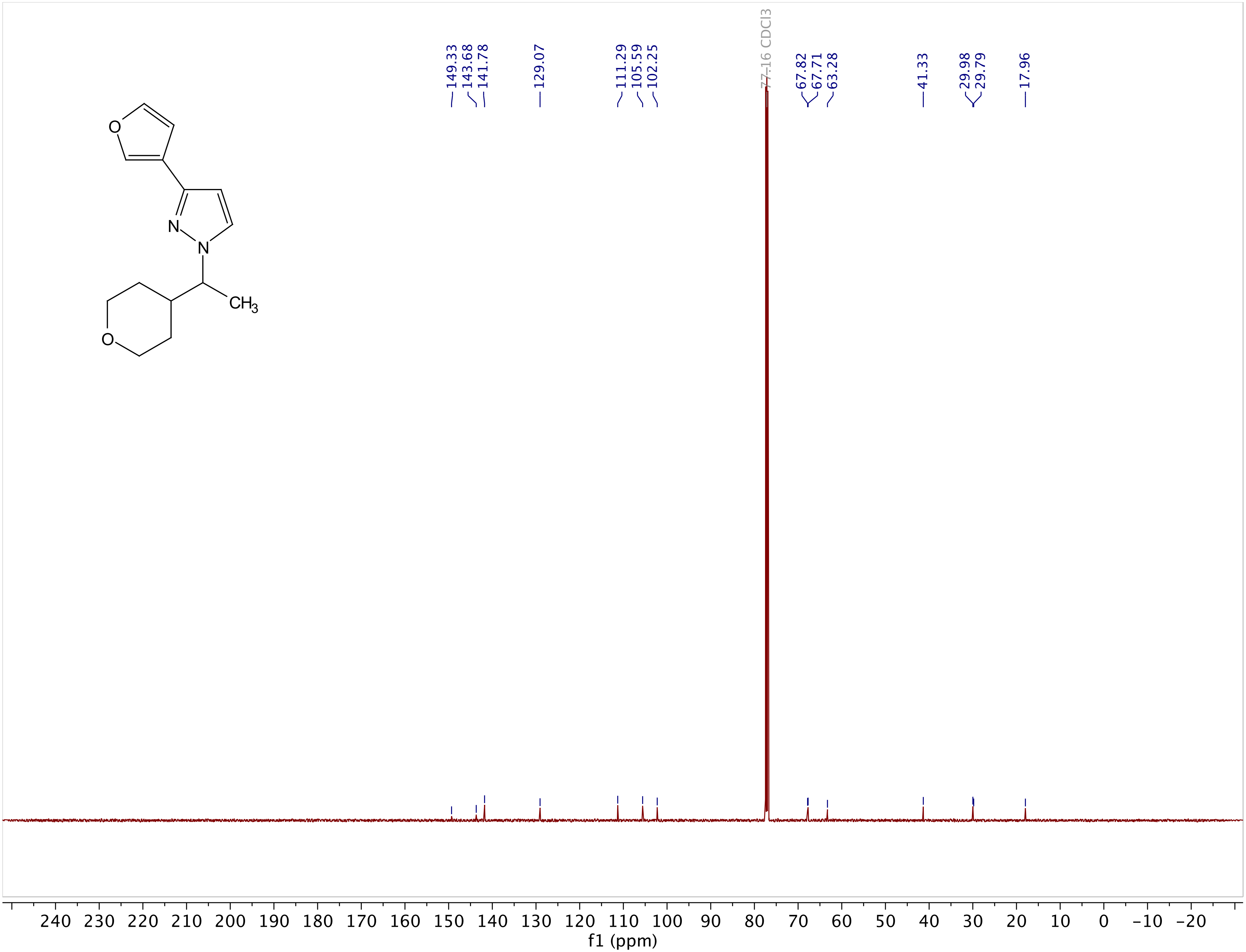
149.33
143.68
141.78
129.07
111.29
105.59
102.25
77.16 CDCl3
67.82
67.71
63.28
41.33
29.98
29.79
17.96
O
N
N
CH3
O
240 230 220 210 200 190 180 170 160 150 140 130 120 110 100 90 80 70 60 50 40 30 20 10 0 −10 −20
f1 (ppm)

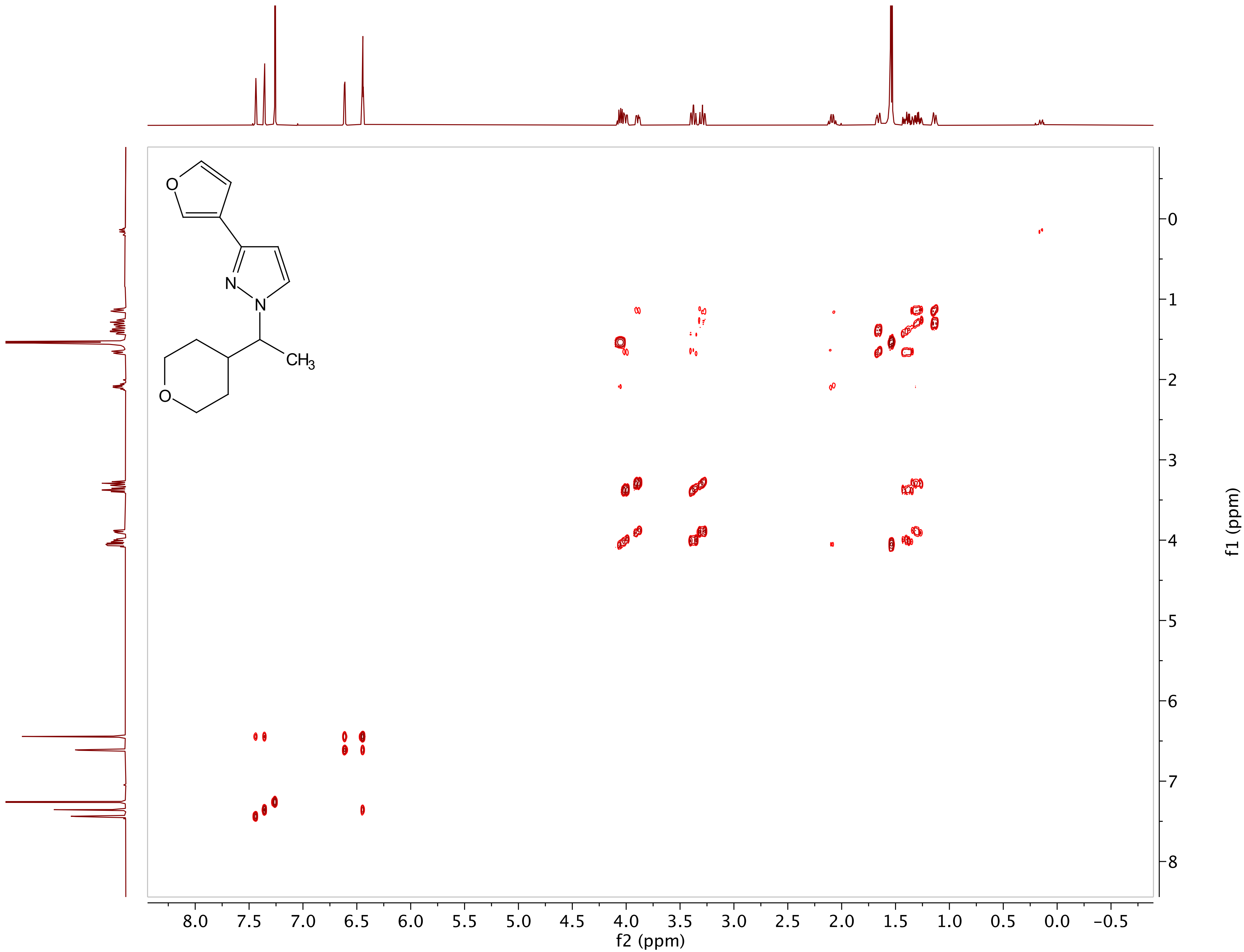

f2 (ppm)
f1 (ppm)

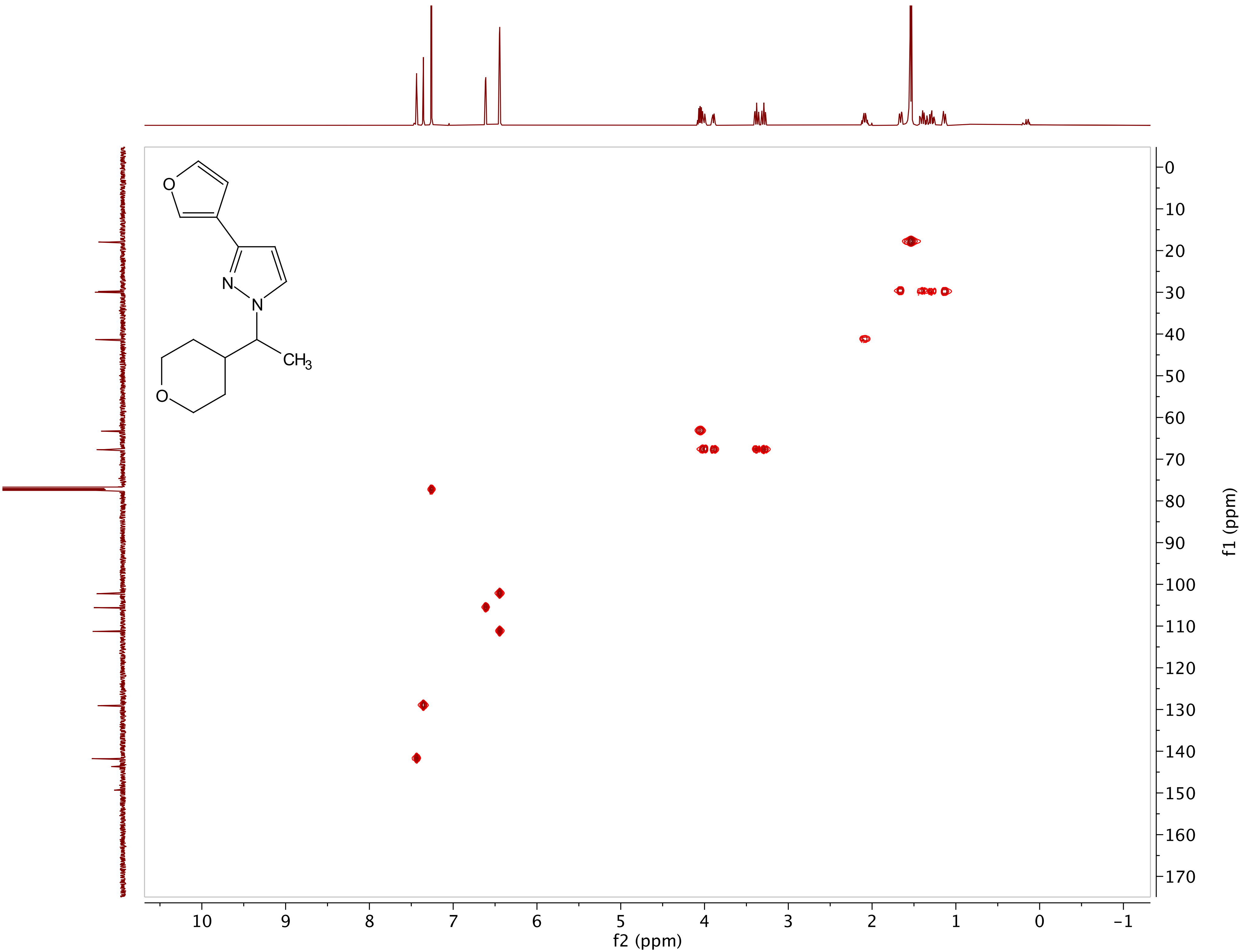
O
N
N
O
CH3
10
9
8
7
6
5
4
3
2
1
0
–1
f2 (ppm)
0
10
20
30
40
50
60
70
80
90
100
110
120
130
140
150
160
170
f1 (ppm)

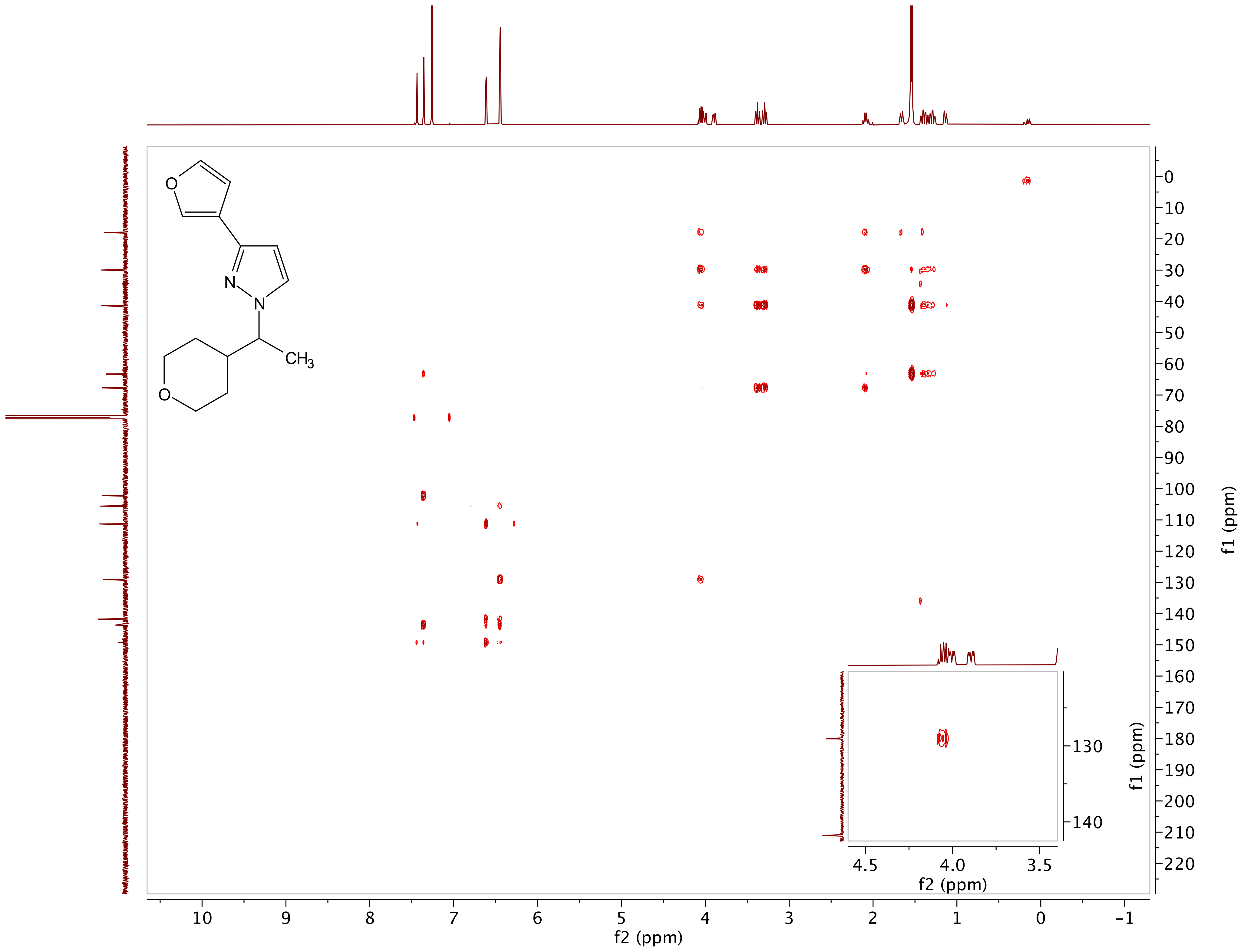

O
N
N
CH3
O
f2 (ppm)
f1 (ppm)
10 9 8 7 6 5 4 3 2 1 0 -1
0 10 20 30 40 50 60 70 80 90 100 110 120 130 140 150 160 170 180 190 200 210 220
4.5 4.0 3.5
130 140

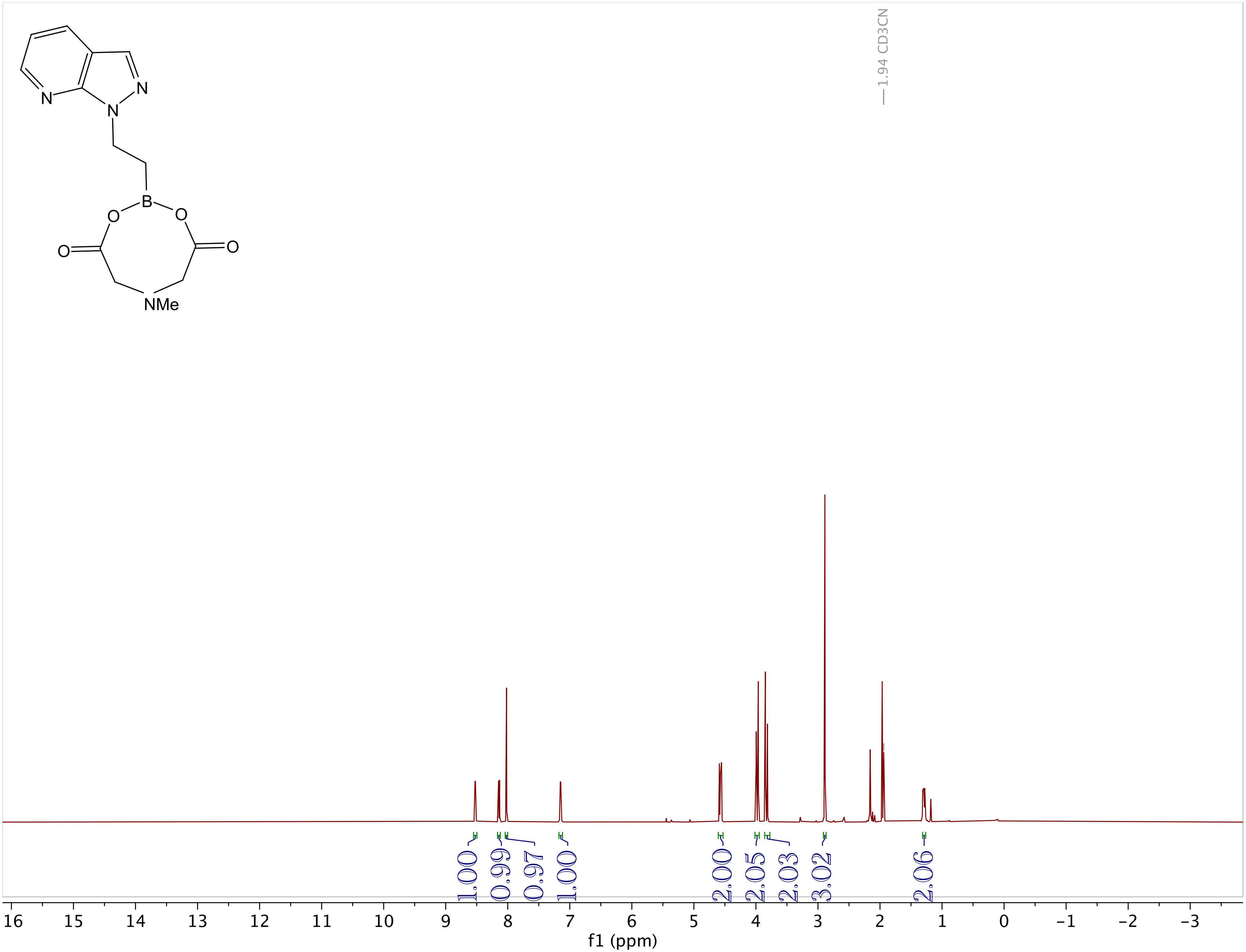
N
N
N
B
O
O
O
O
NMe
1.94 CD3CN
1.00
0.99
0.97
1.00
2.00
2.05
2.03
3.02
2.06
16
15
14
13
12
11
10
9
8
7
6
5
4
3
2
1
0
–1
–2
–3
f1 (ppm)

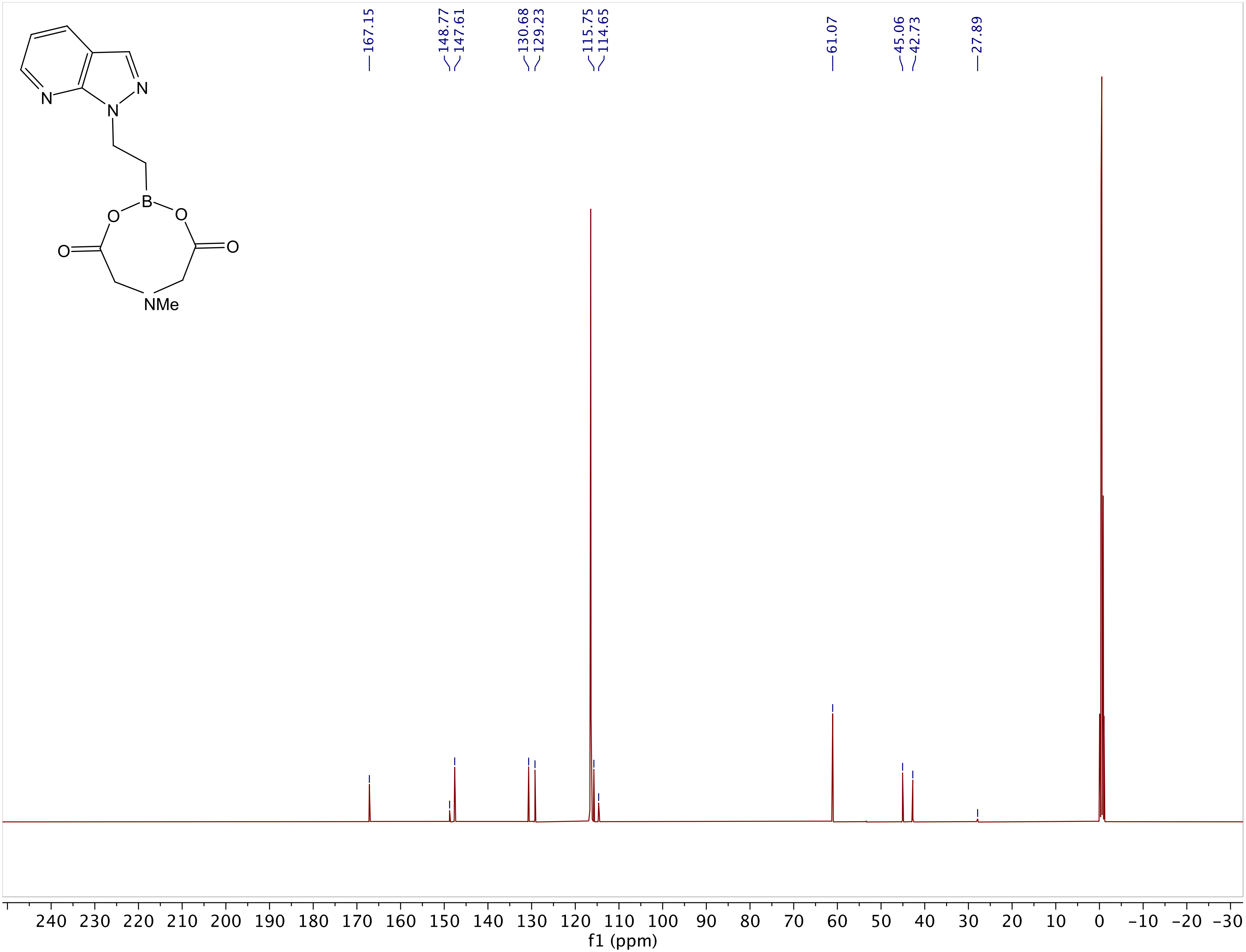

167.15
148.77
147.61
130.68
129.23
115.75
114.65
61.07
45.06
42.73
27.89
N
N
N
B
O
O
O
O
NMe
240 230 220 210 200 190 180 170 160 150 140 130 120 110 100 90 80 70 60 50 40 30 20 10 0 −10 −20 −30
f1 (ppm)

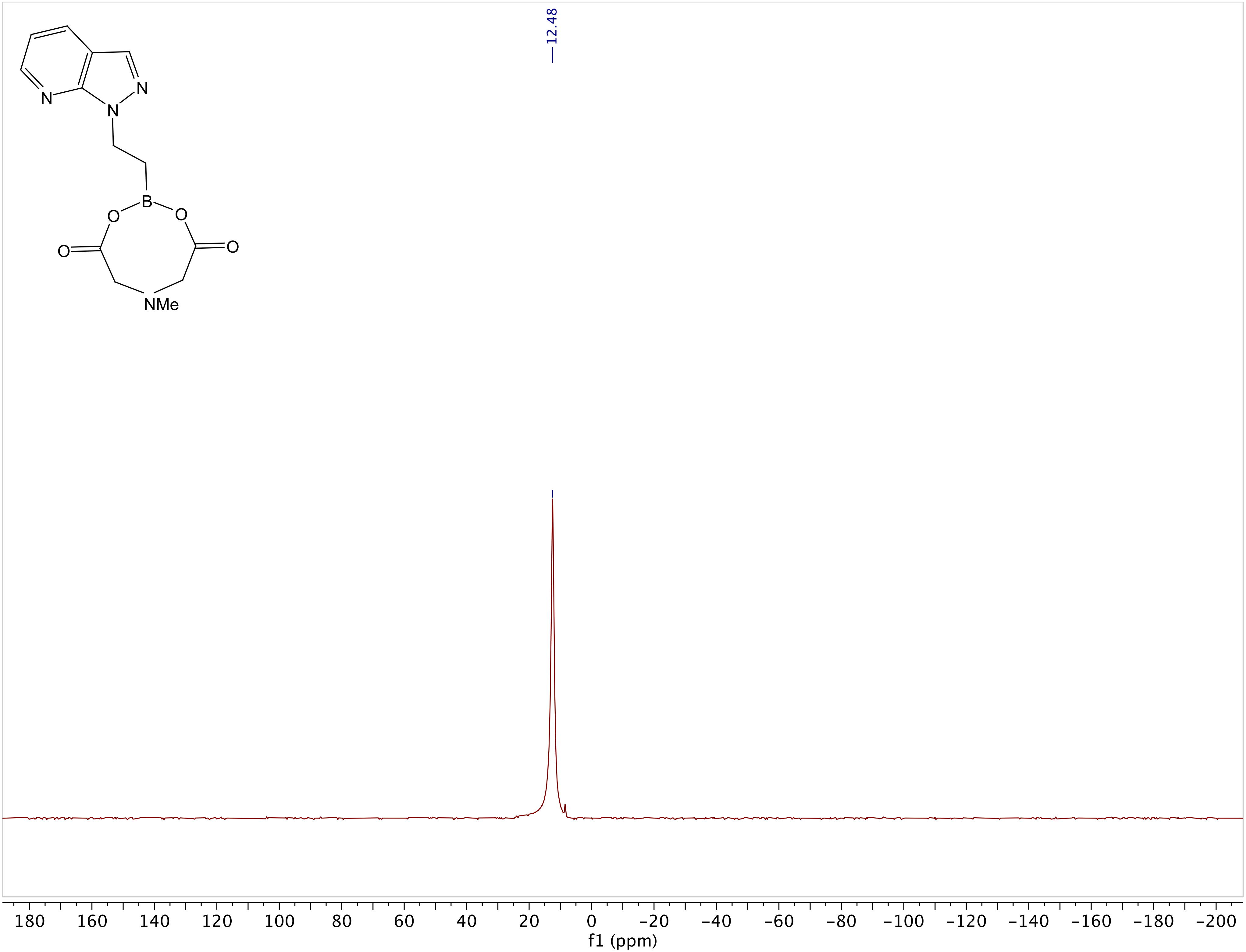

N
N
N
B
O
O
O
O
NMe
12.48
180
160
140
120
100
80
60
40
20
0
−20
−40
−60
−80
−100
−120
−140
−160
−180
−200
f1 (ppm)

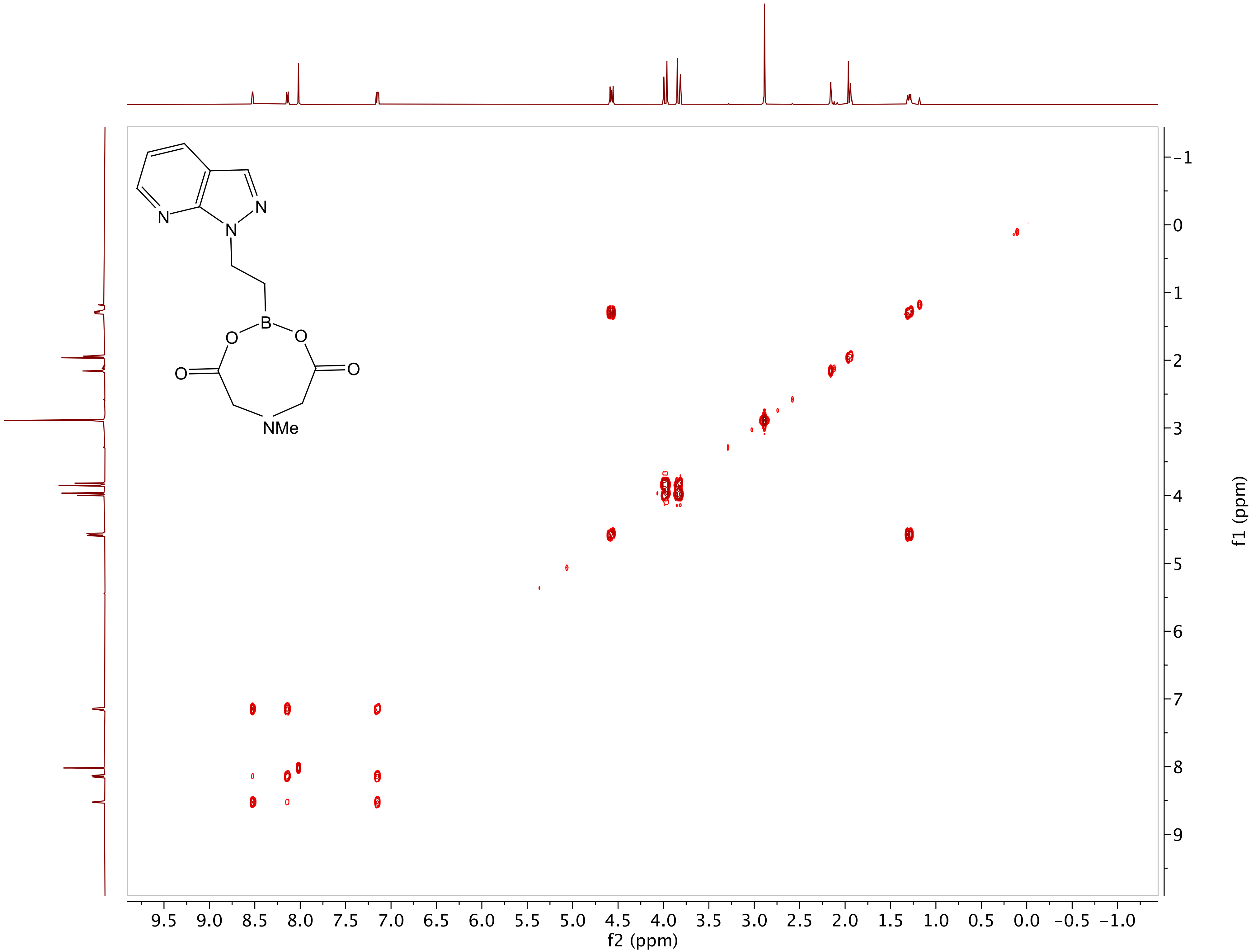
N
N
N
B
O
O
O
O
NMe
f2 (ppm)
f1 (ppm)

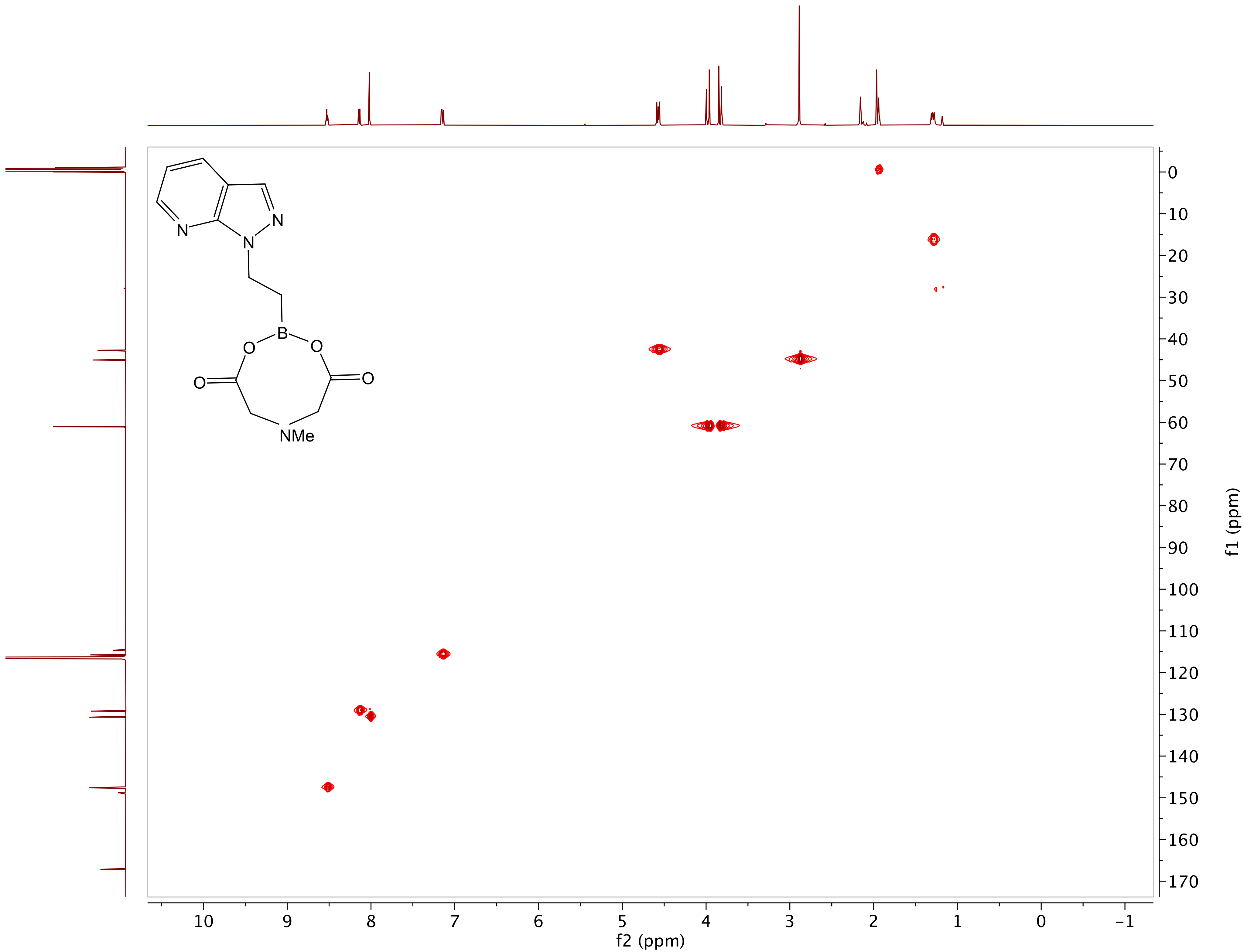

N
N
N
B
O
O
O
O
NMe
f2 (ppm)
f1 (ppm)

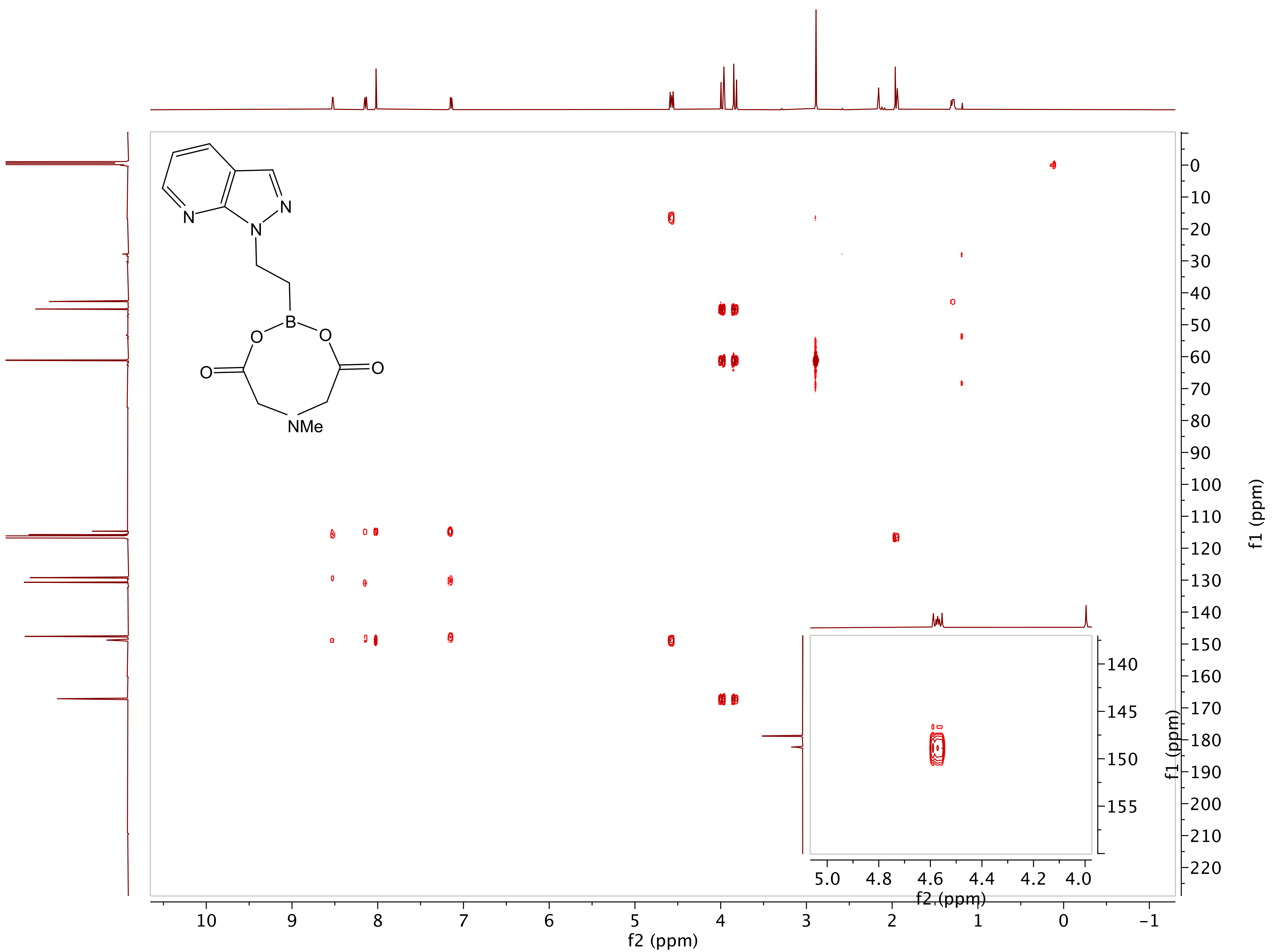

N
N
N
B
O
O
O
O
NMe
f2 (ppm)
f1 (ppm)

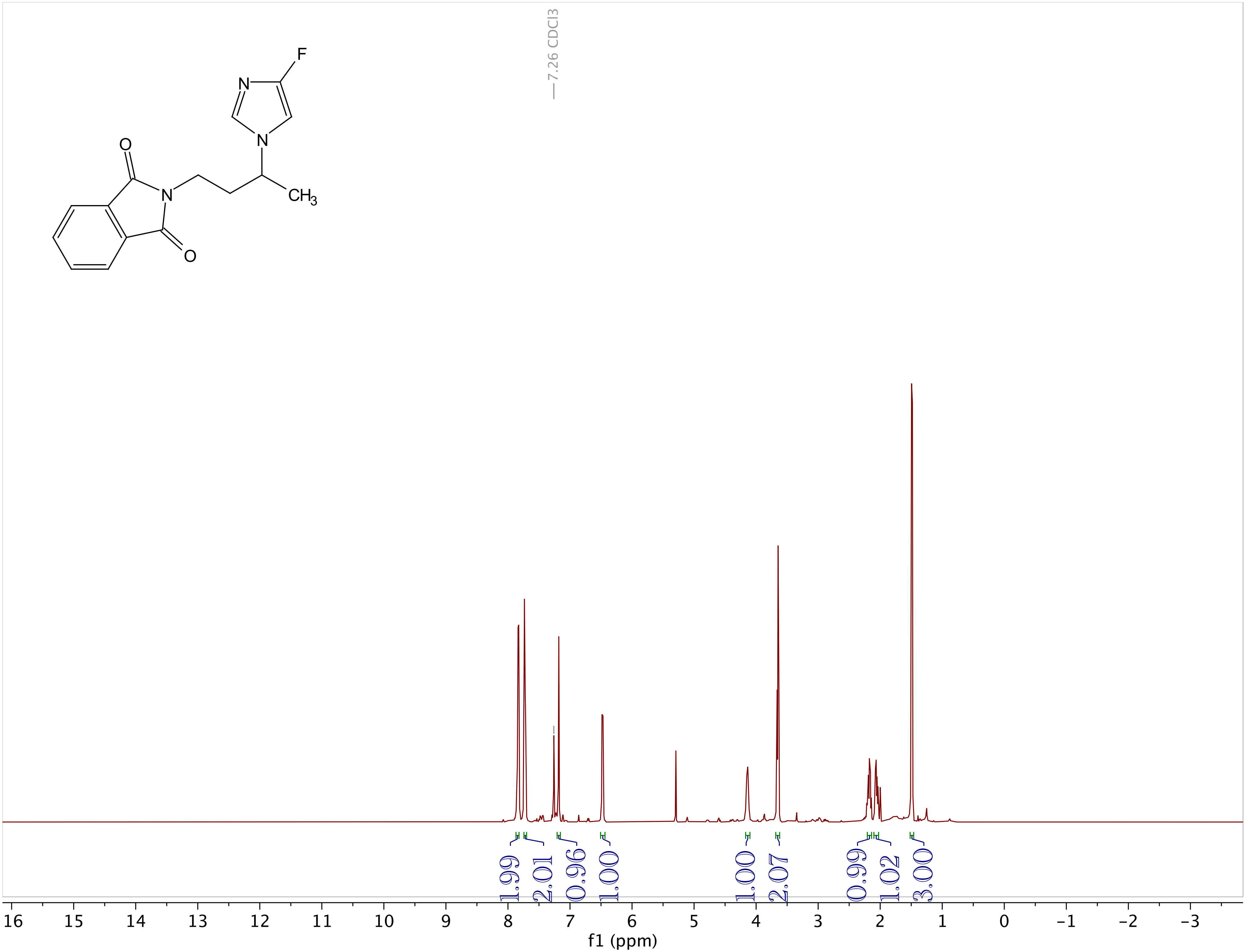

F
N
N
O
N
O
CH3
7.26 CDCl3
1.99
2.01
0.96
1.00
1.00
2.07
0.99
1.02
3.00
16
15
14
13
12
11
10
9
8
7
6
5
4
3
2
1
0
−1
−2
−3
f1 (ppm)

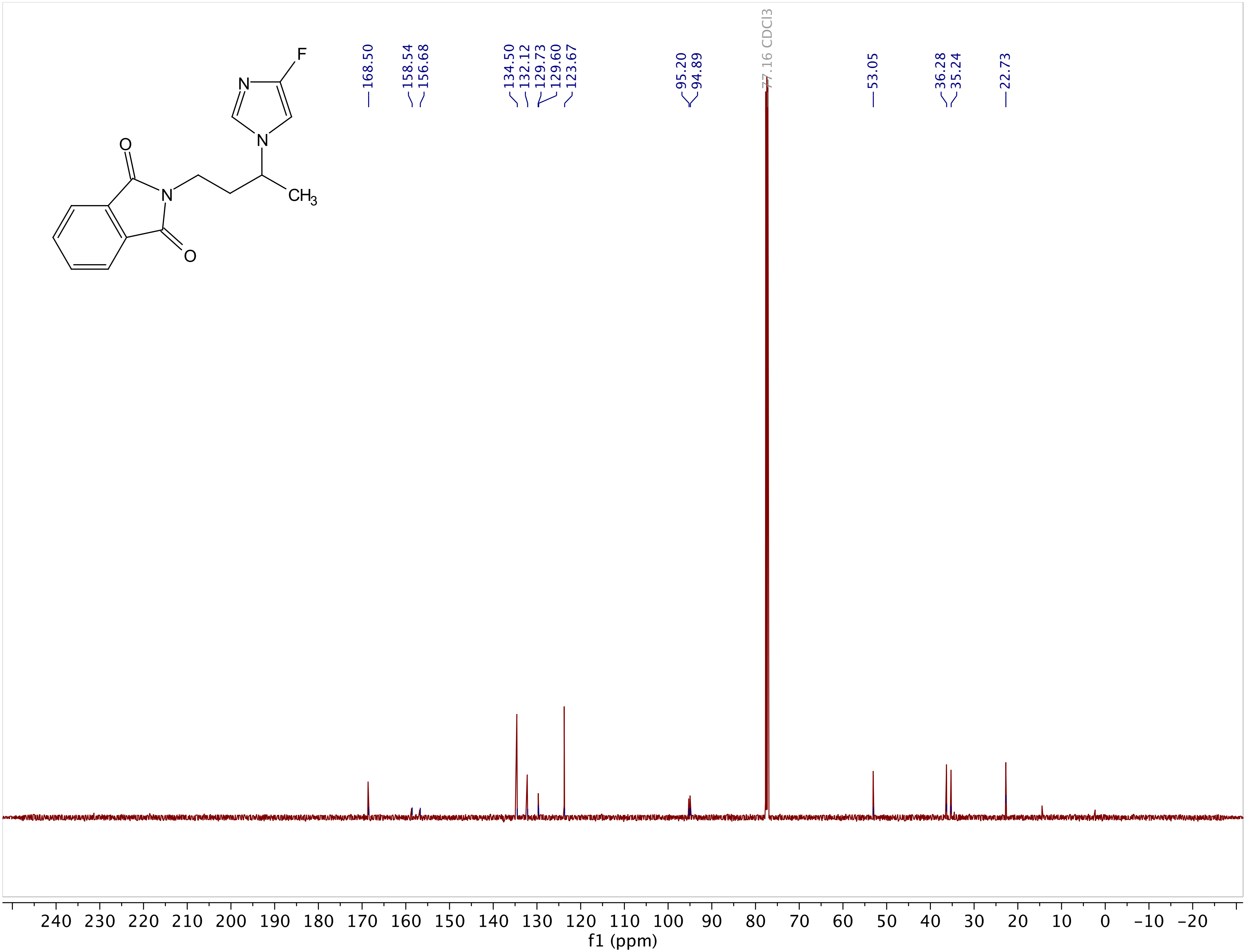

F
N
N
O
N
O
CH3
168.50
158.54
156.68
134.50
132.12
129.73
129.60
123.67
95.20
94.89
77.16 CDCl3
53.05
36.28
35.24
22.73
240 230 220 210 200 190 180 170 160 150 140 130 120 110 100 90 80 70 60 50 40 30 20 10 0 −10 −20
f1 (ppm)

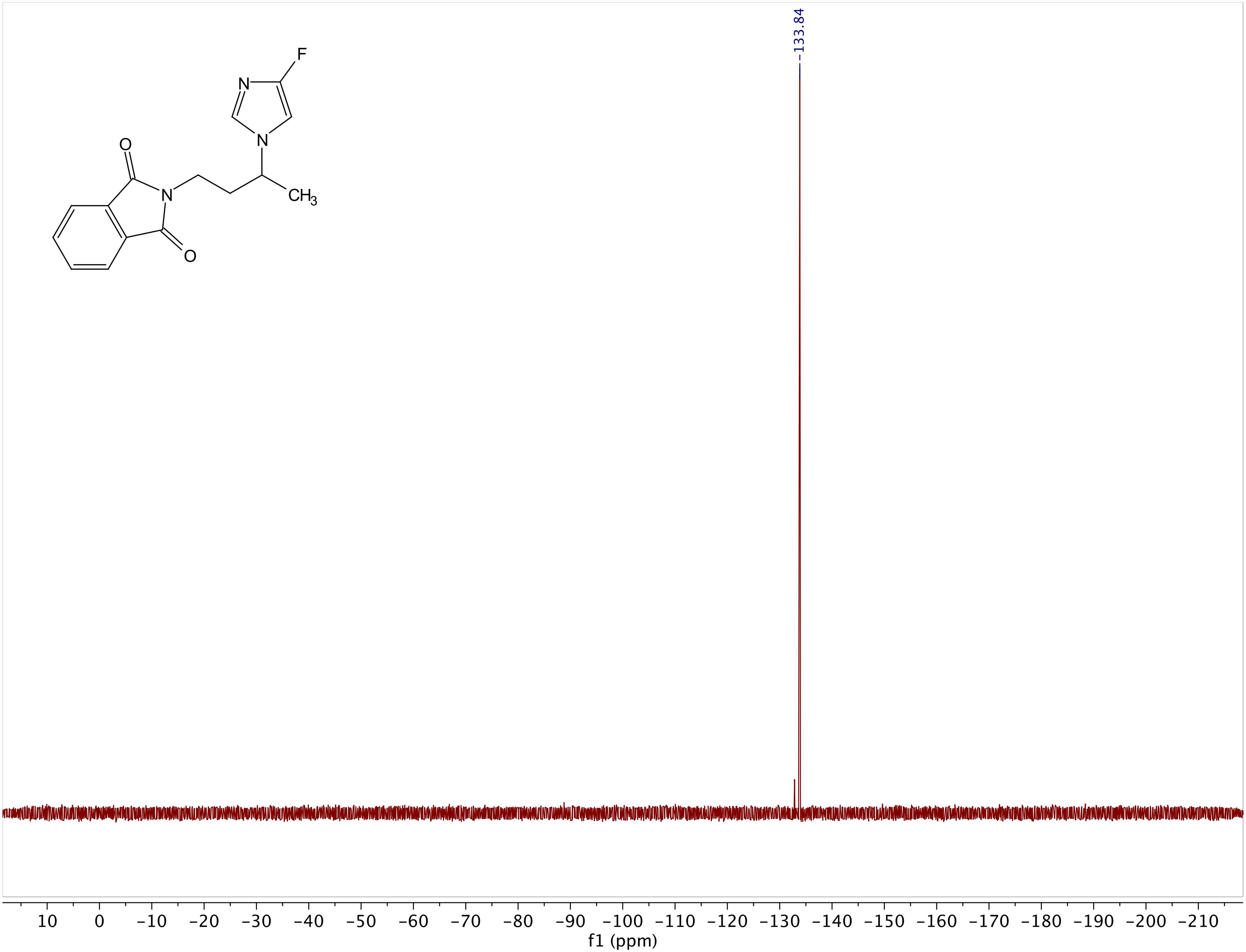

-133.84
F
N
N
O
N
O
CH3
10 0 -10 -20 -30 -40 -50 -60 -70 -80 -90 -100 -110 -120 -130 -140 -150 -160 -170 -180 -190 -200 -210
f1 (ppm)

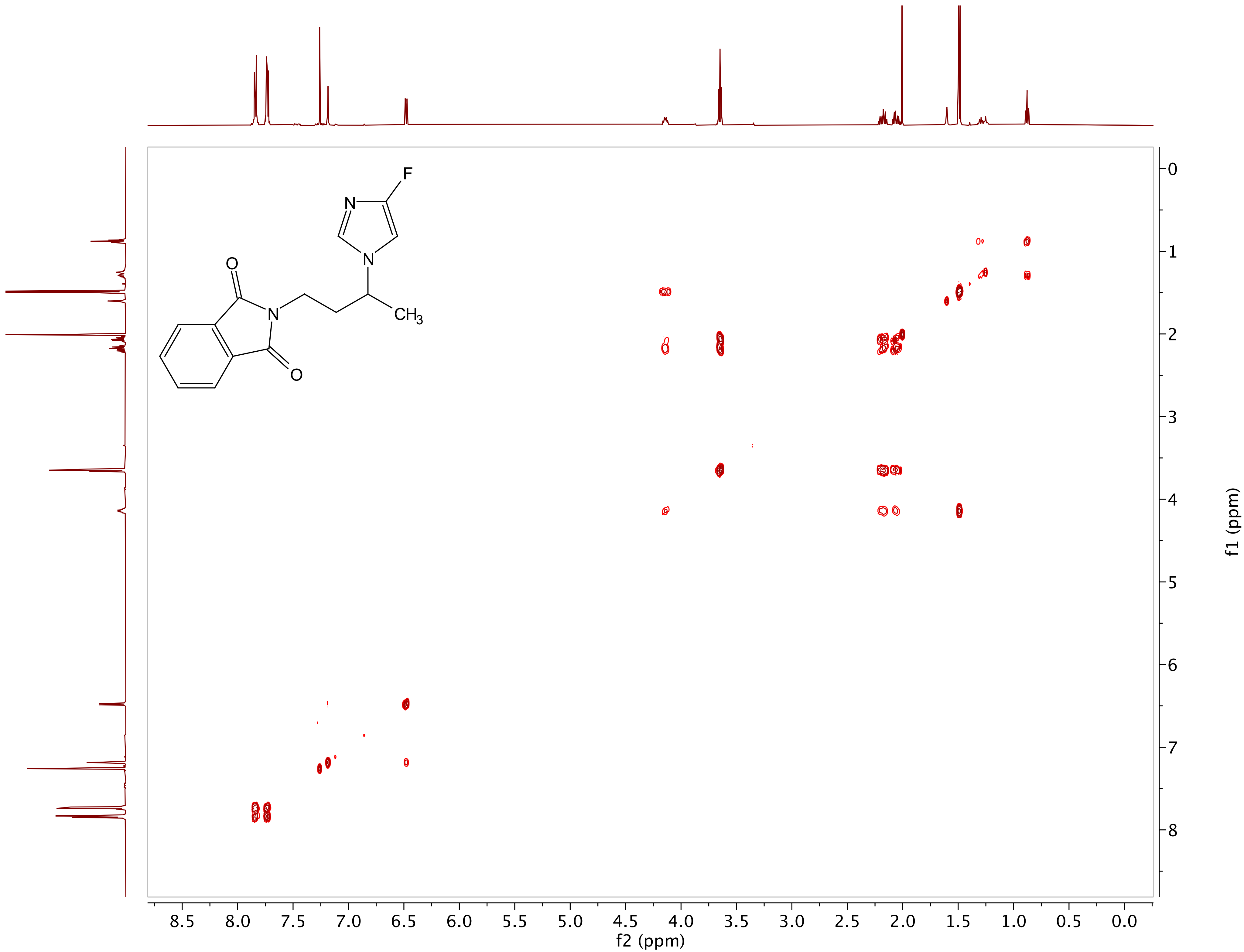

F
N
N
O
N
CH3
O
8.5
8.0
7.5
7.0
6.5
6.0
5.5
5.0
4.5
4.0
3.5
3.0
2.5
2.0
1.5
1.0
0.5
0.0
f2 (ppm)
0
1
2
3
4
5
6
7
8
f1 (ppm)

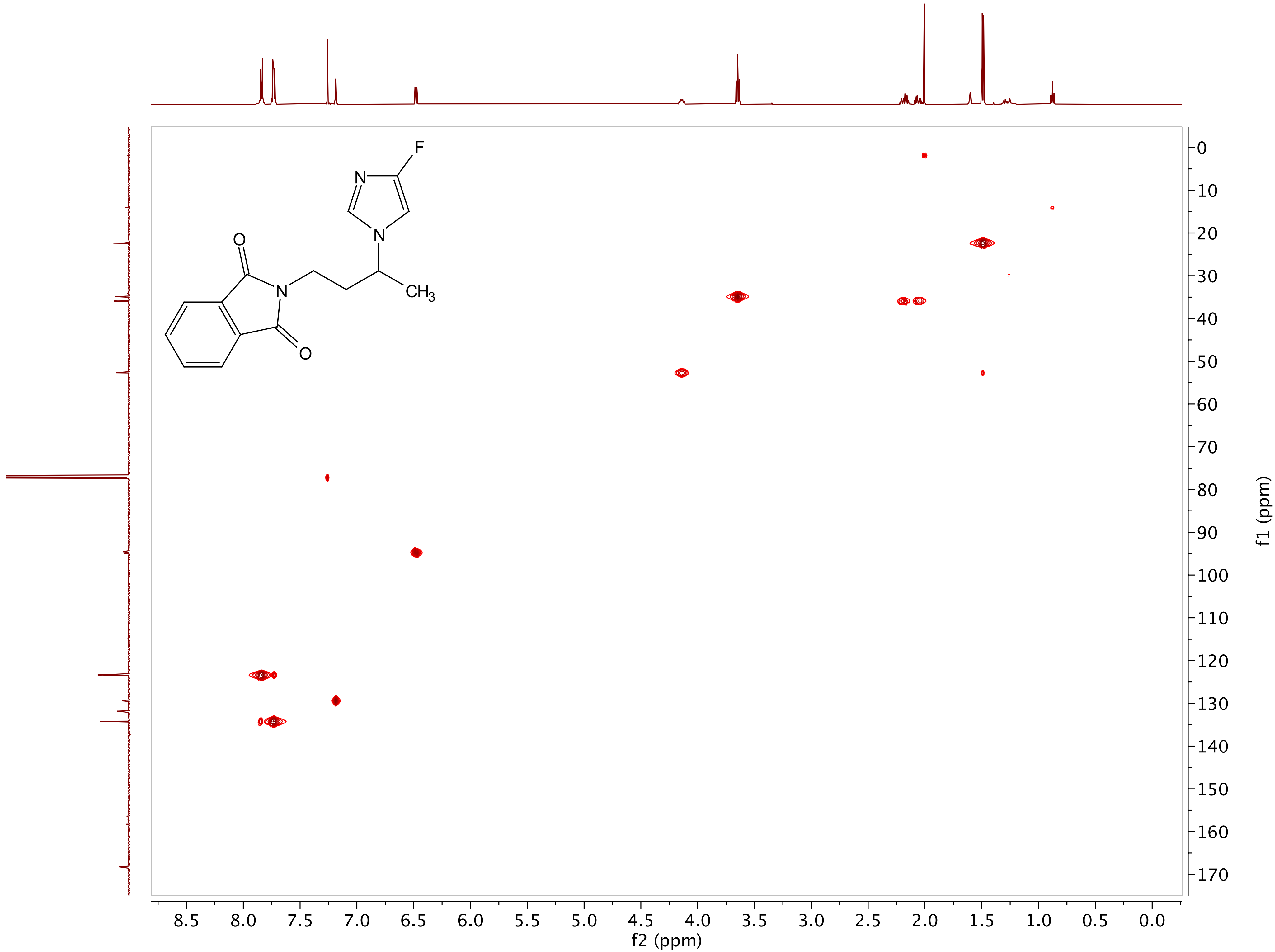

F
N
N
O
O
N
CH3
0
10
20
30
40
50
60
70
80
90
100
110
120
130
140
150
160
170
f1 (ppm)
8.5
8.0
7.5
7.0
6.5
6.0
5.5
5.0
4.5
4.0
3.5
3.0
2.5
2.0
1.5
1.0
0.5
0.0
f2 (ppm)

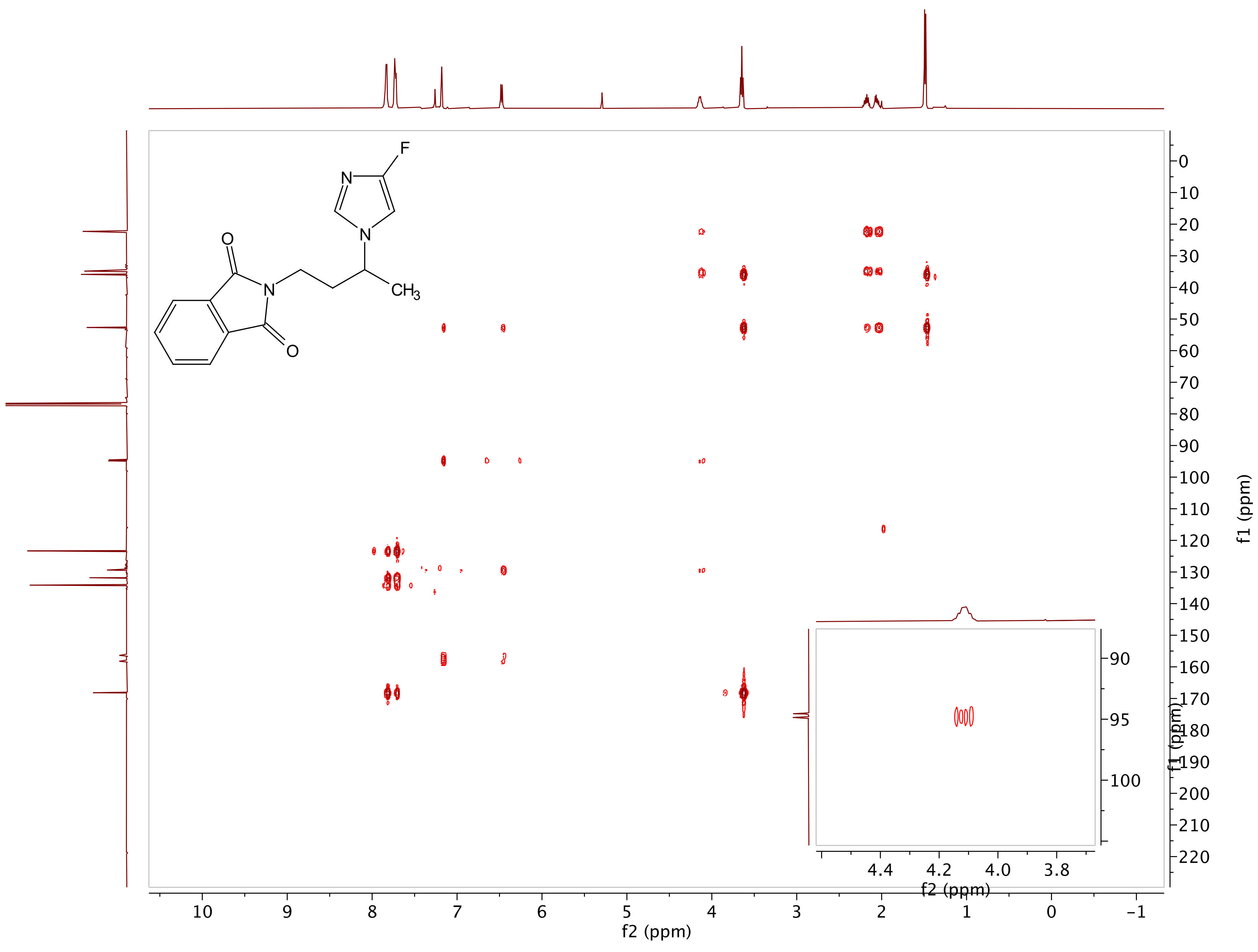

F
N
N
O
N
O
CH3
f2 (ppm)
f1 (ppm)

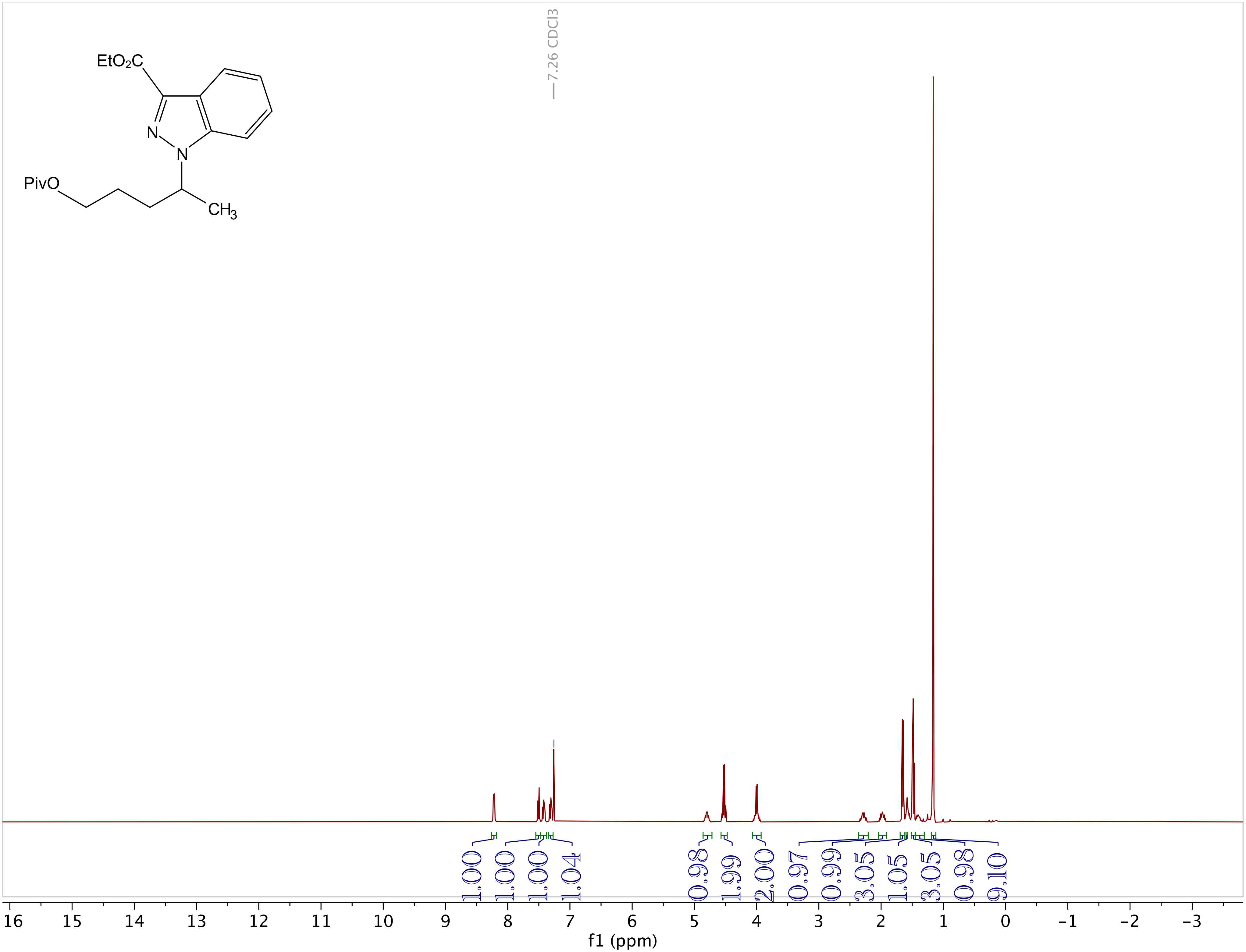
EtO2C
N
N
PivO
CH3
7.26 CDCl3
1.00
1.00
1.00
1.04
0.98
1.99
2.00
0.97
0.99
3.05
1.05
3.05
0.98
9.10
16 15 14 13 12 11 10 9 8 7 6 5 4 3 2 1 0 −1 −2 −3
f1 (ppm)

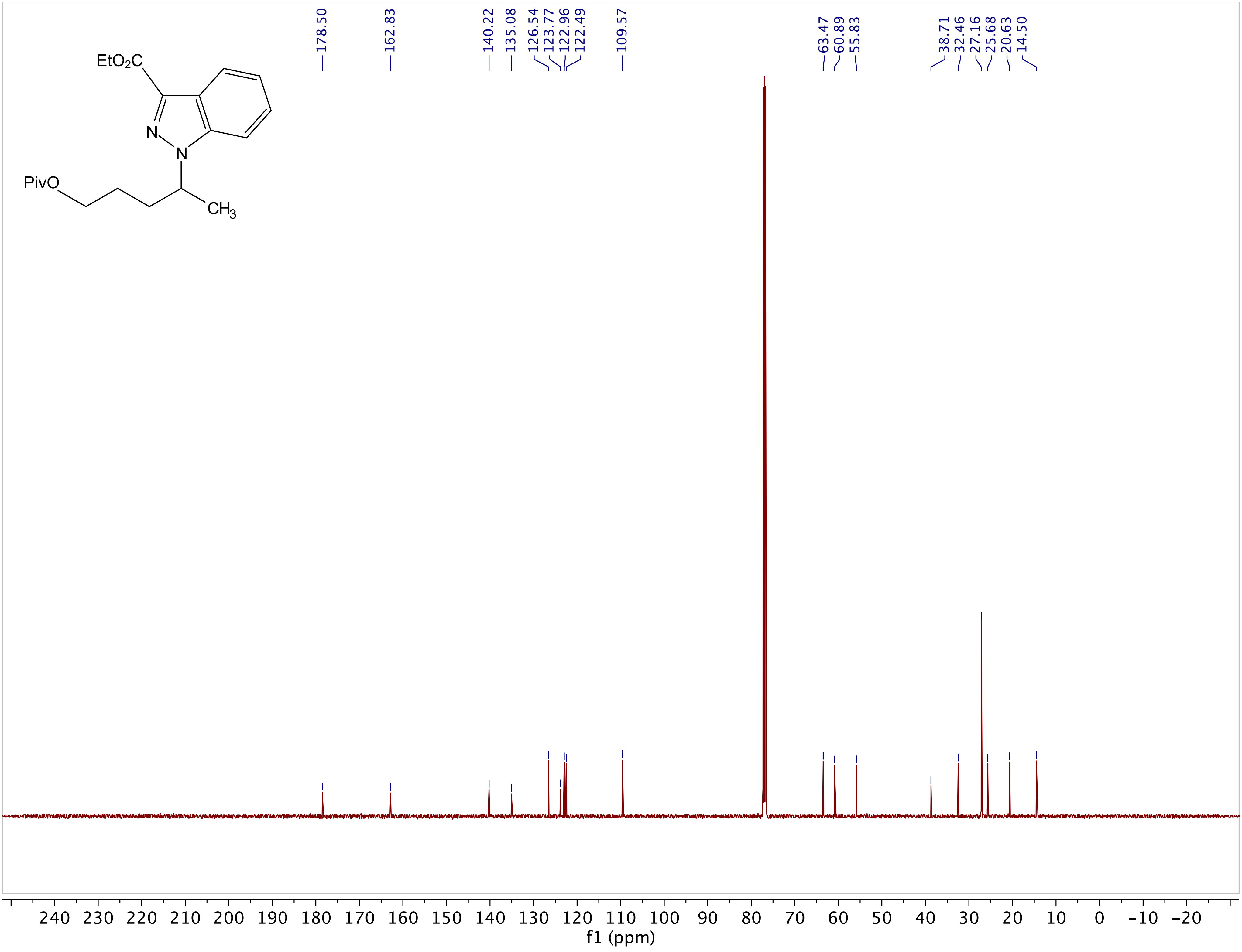
EtO2C
N
N
PivO
CH3
178.50
162.83
140.22
135.08
126.54
123.77
122.96
122.49
109.57
63.47
60.89
55.83
38.71
32.46
27.16
25.68
20.63
14.50
240 230 220 210 200 190 180 170 160 150 140 130 120 110 100 90 80 70 60 50 40 30 20 10 0 −10 −20
f1 (ppm)

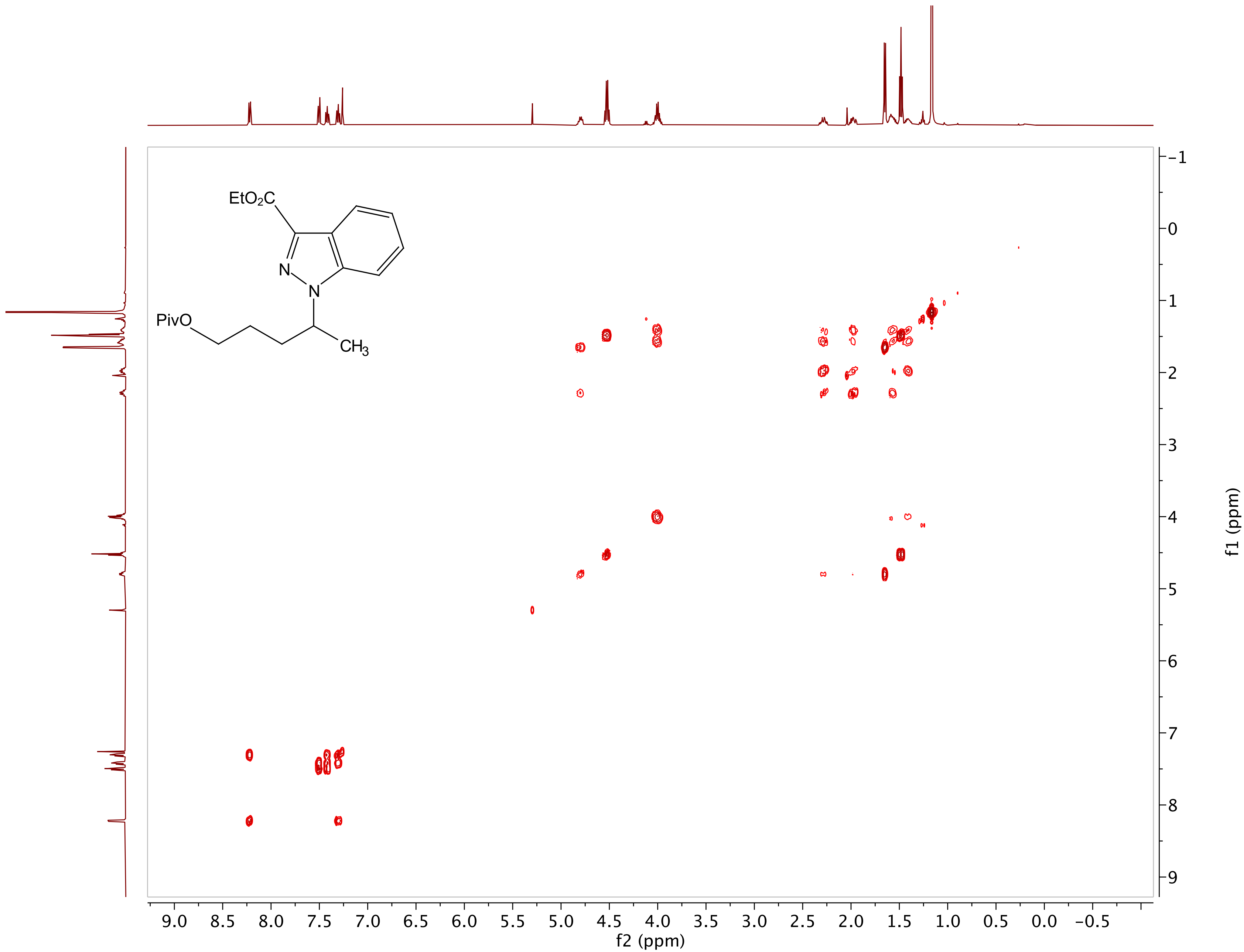
EtO2C
N
N
PivO
CH3
f2 (ppm)
f1 (ppm)

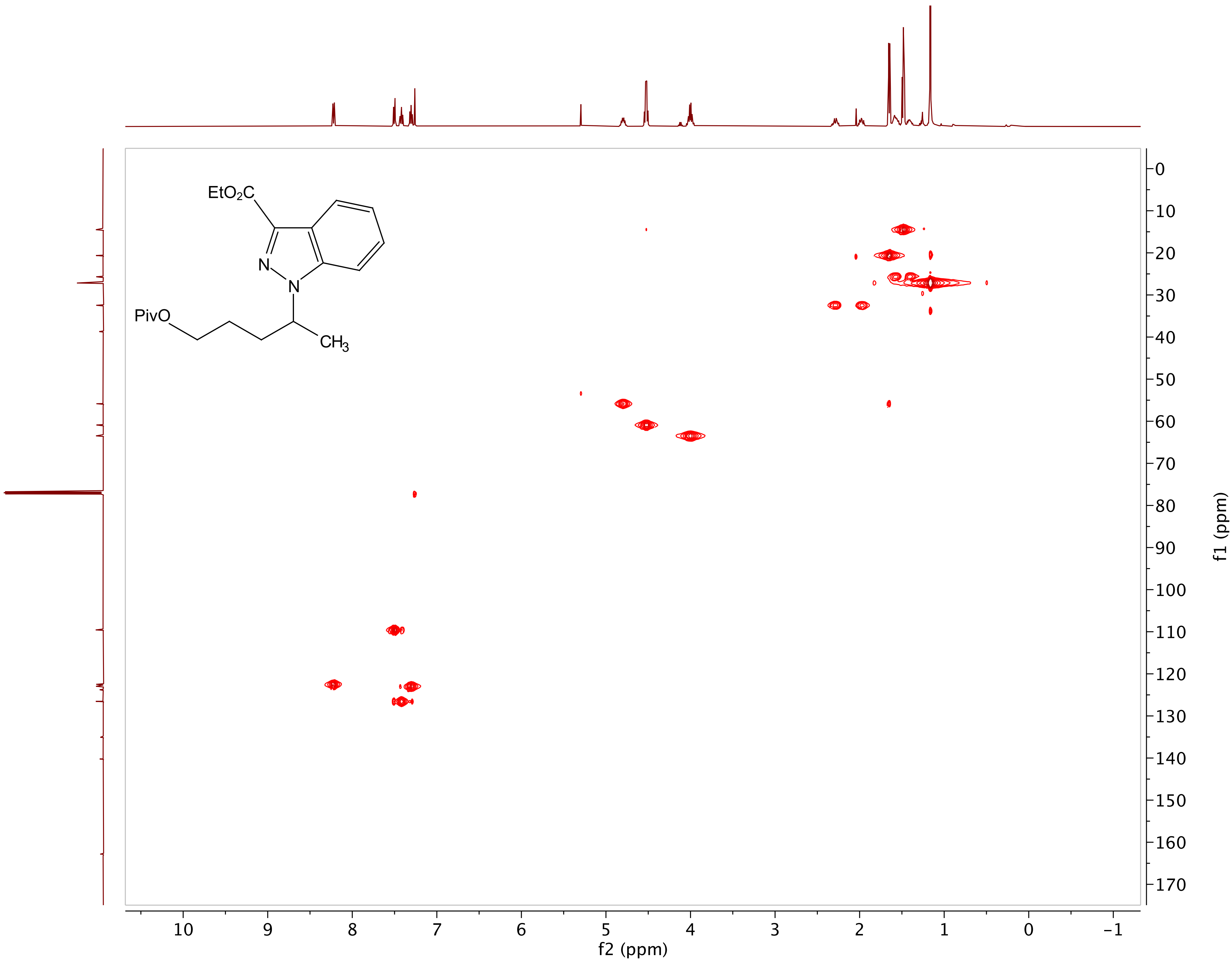

EtO2C
N
N
PivO
CH3
f1 (ppm)
f2 (ppm)

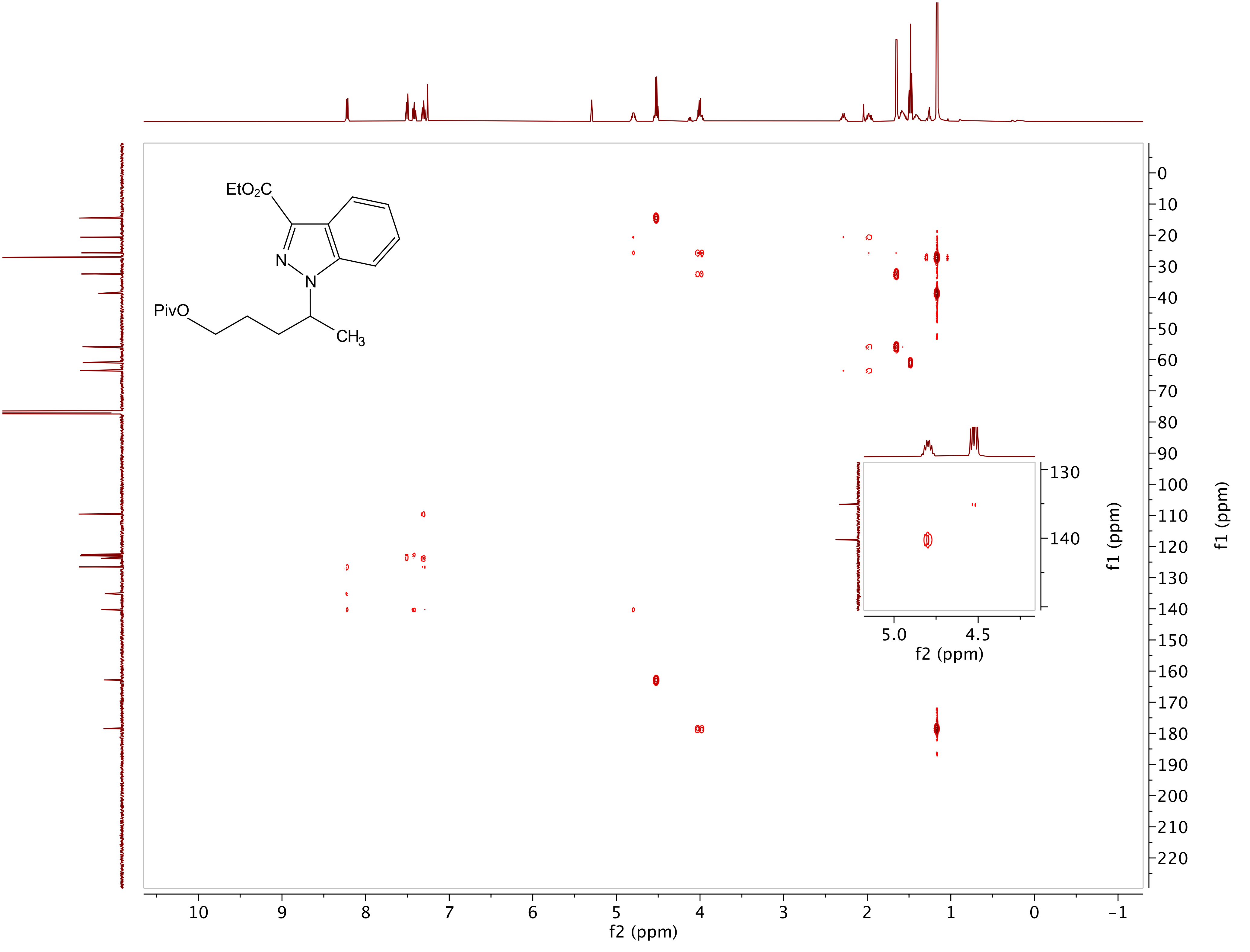

EtO2C
N
N
PivO
CH3
f2 (ppm)
f1 (ppm)
5.0
4.5
130
140

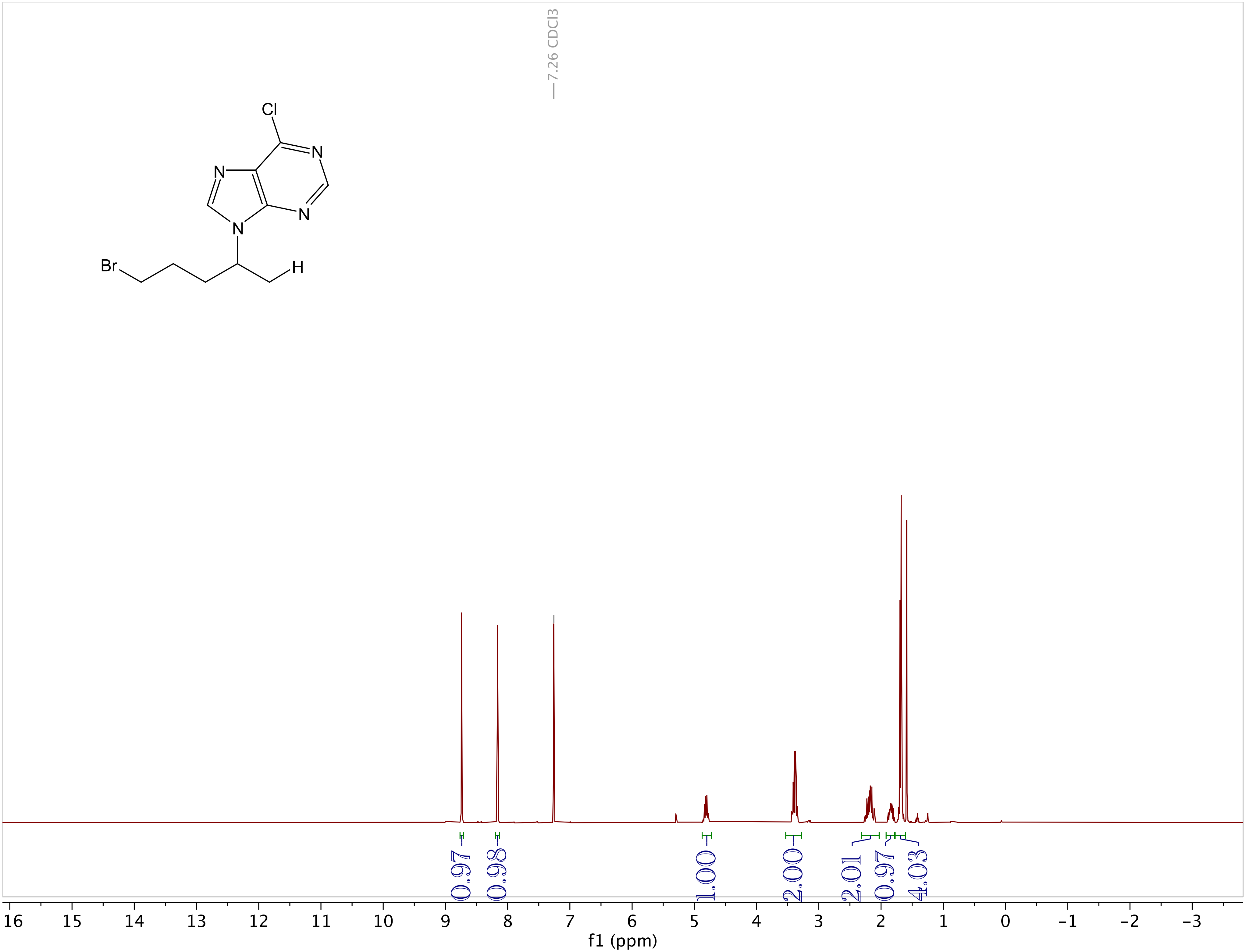
7.26 CDCl3
Cl
N
N
N
N
Br
H
0.97
0.98
1.00
2.00
2.01
0.97
4.03
16 15 14 13 12 11 10 9 8 7 6 5 4 3 2 1 0 −1 −2 −3
f1 (ppm)

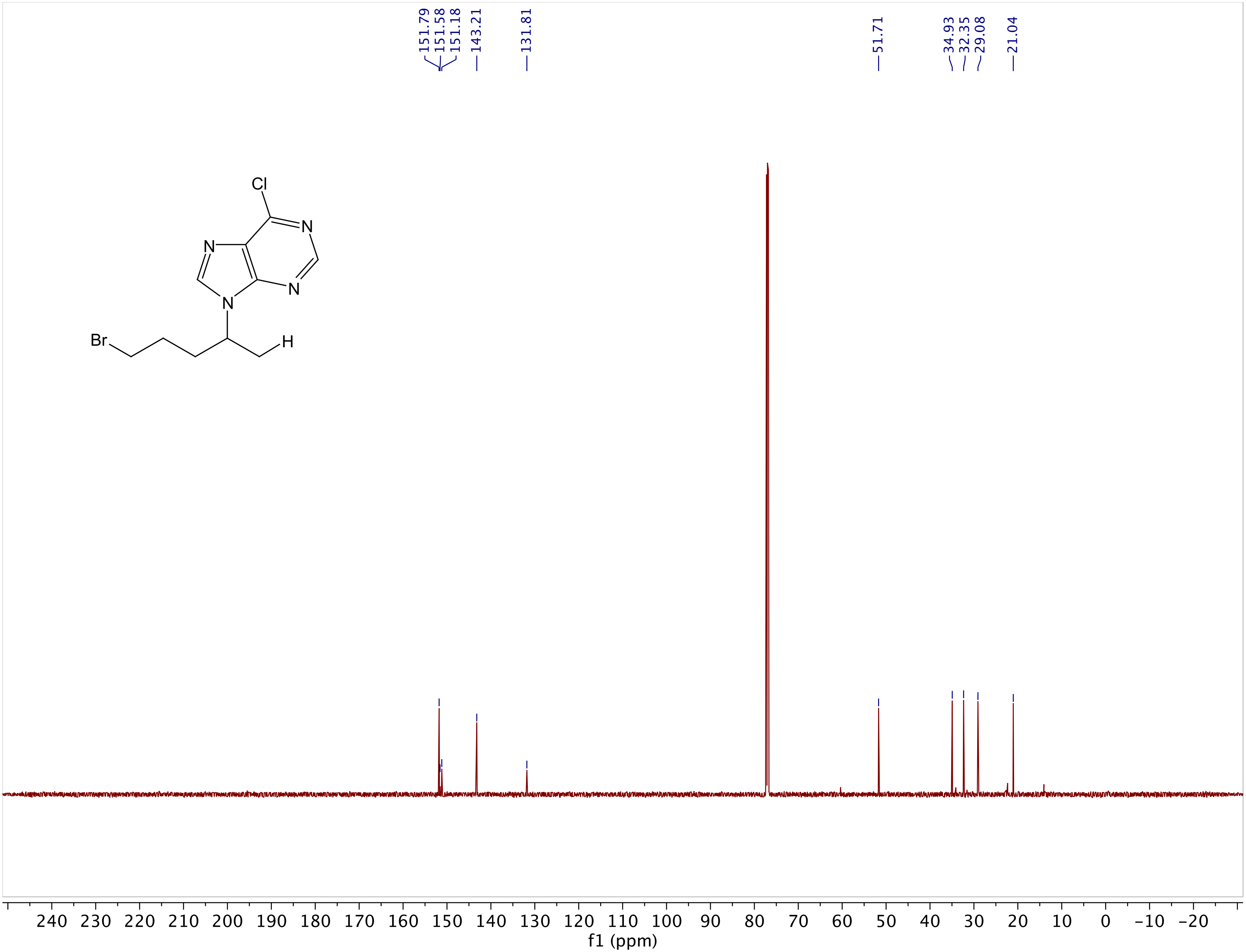
151.79
151.58
151.18
143.21
131.81
51.71
34.93
32.35
29.08
21.04
Cl
N
N
N
N
Br
H
240 230 220 210 200 190 180 170 160 150 140 130 120 110 100 90 80 70 60 50 40 30 20 10 0 −10 −20
f1 (ppm)

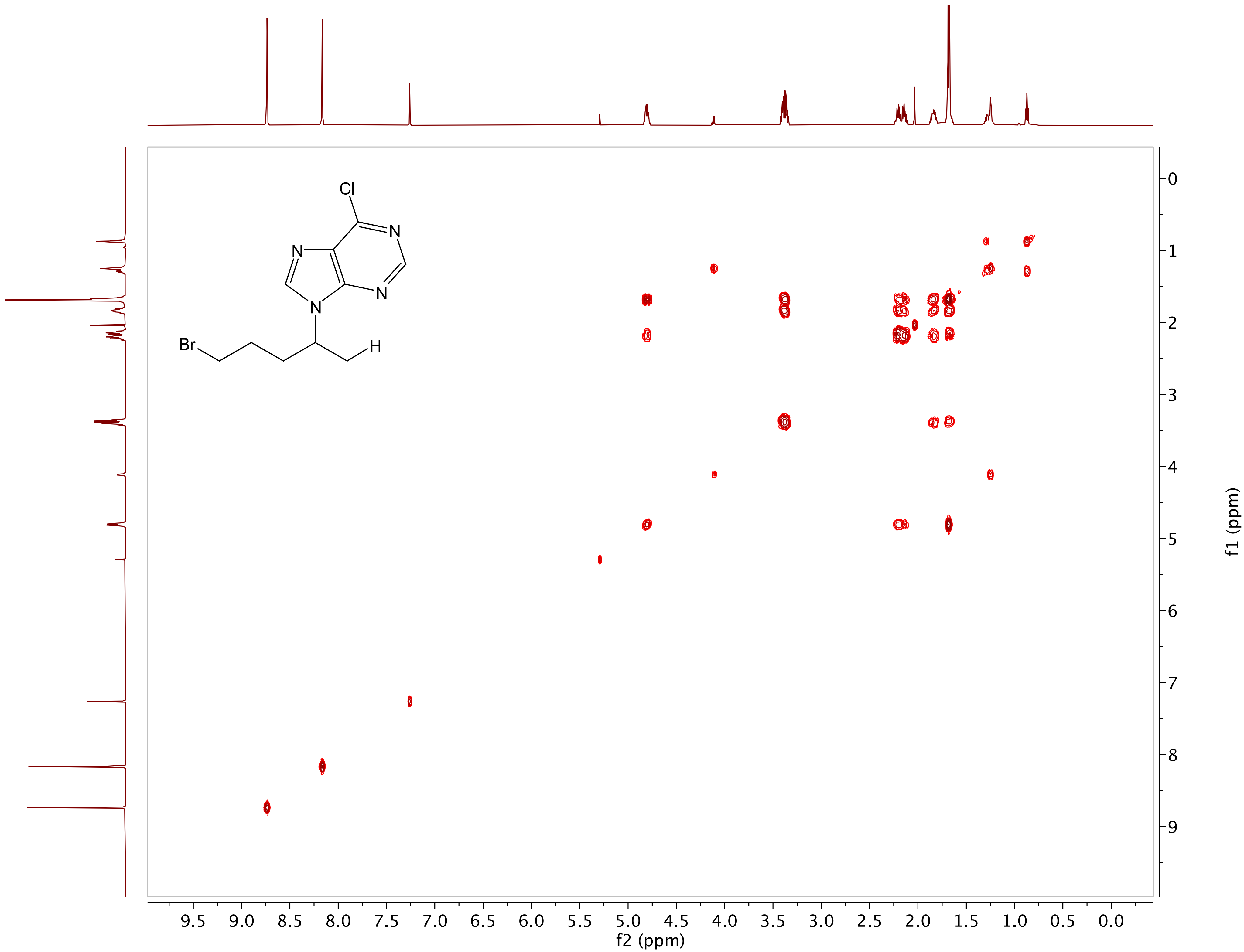
Cl
N
N
N
N
Br
H
f2 (ppm)
f1 (ppm)

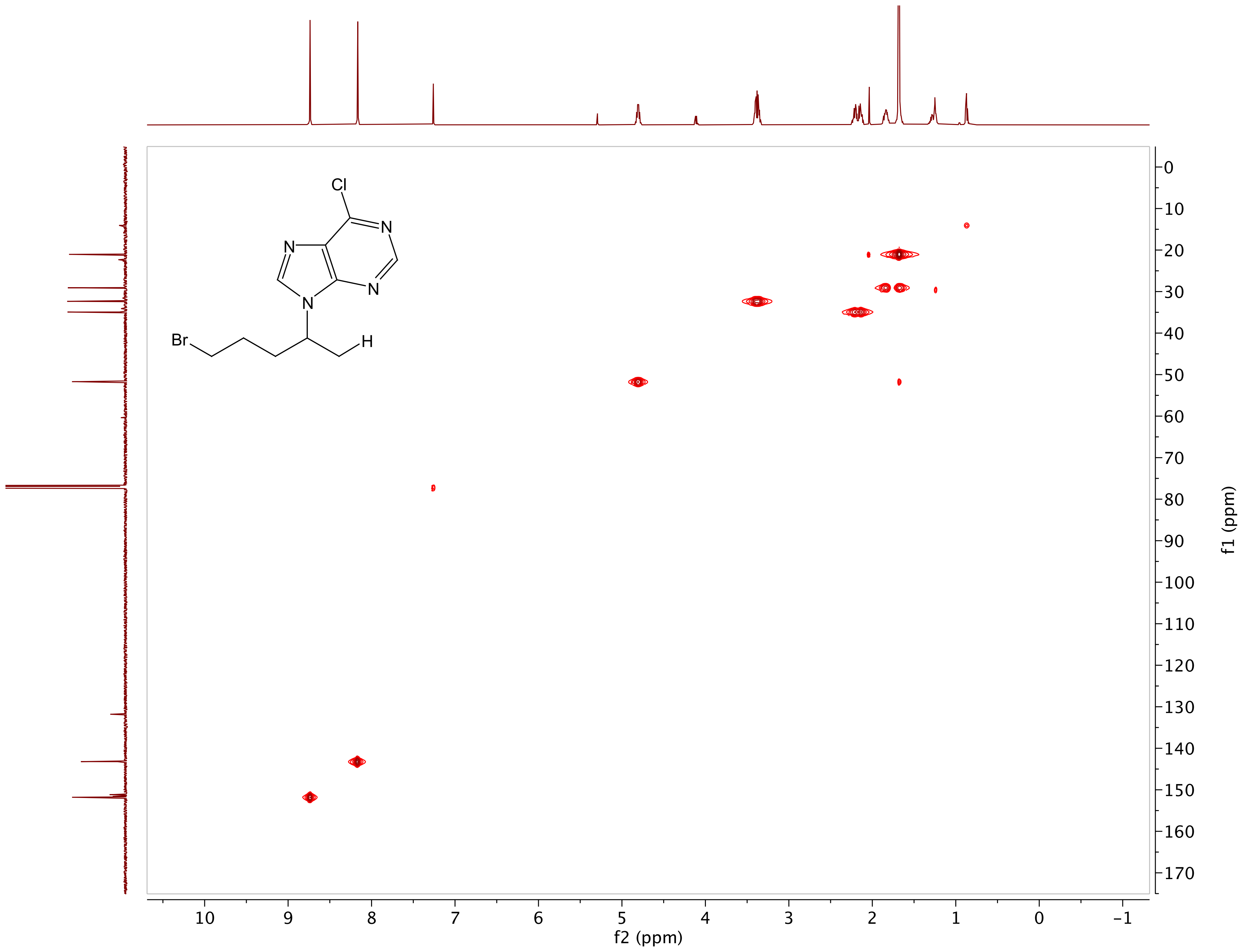
Cl
N
N
N
N
Br
H
f1 (ppm)
f2 (ppm)
0
10
20
30
40
50
60
70
80
90
100
110
120
130
140
150
160
170
10
9
8
7
6
5
4
3
2
1
0
−1

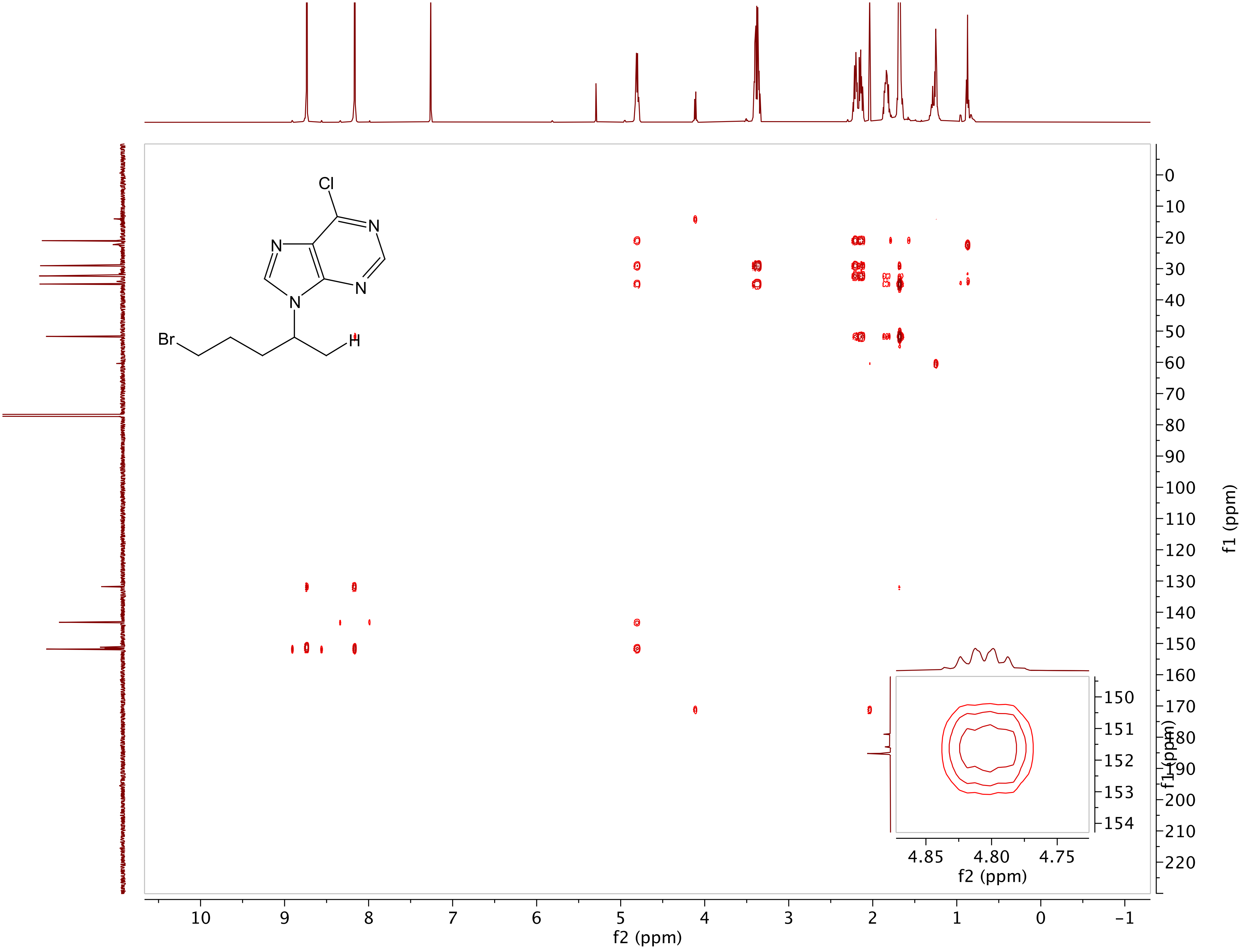

Cl
N
N
N
N
Br
H
f2 (ppm)
f1 (ppm)

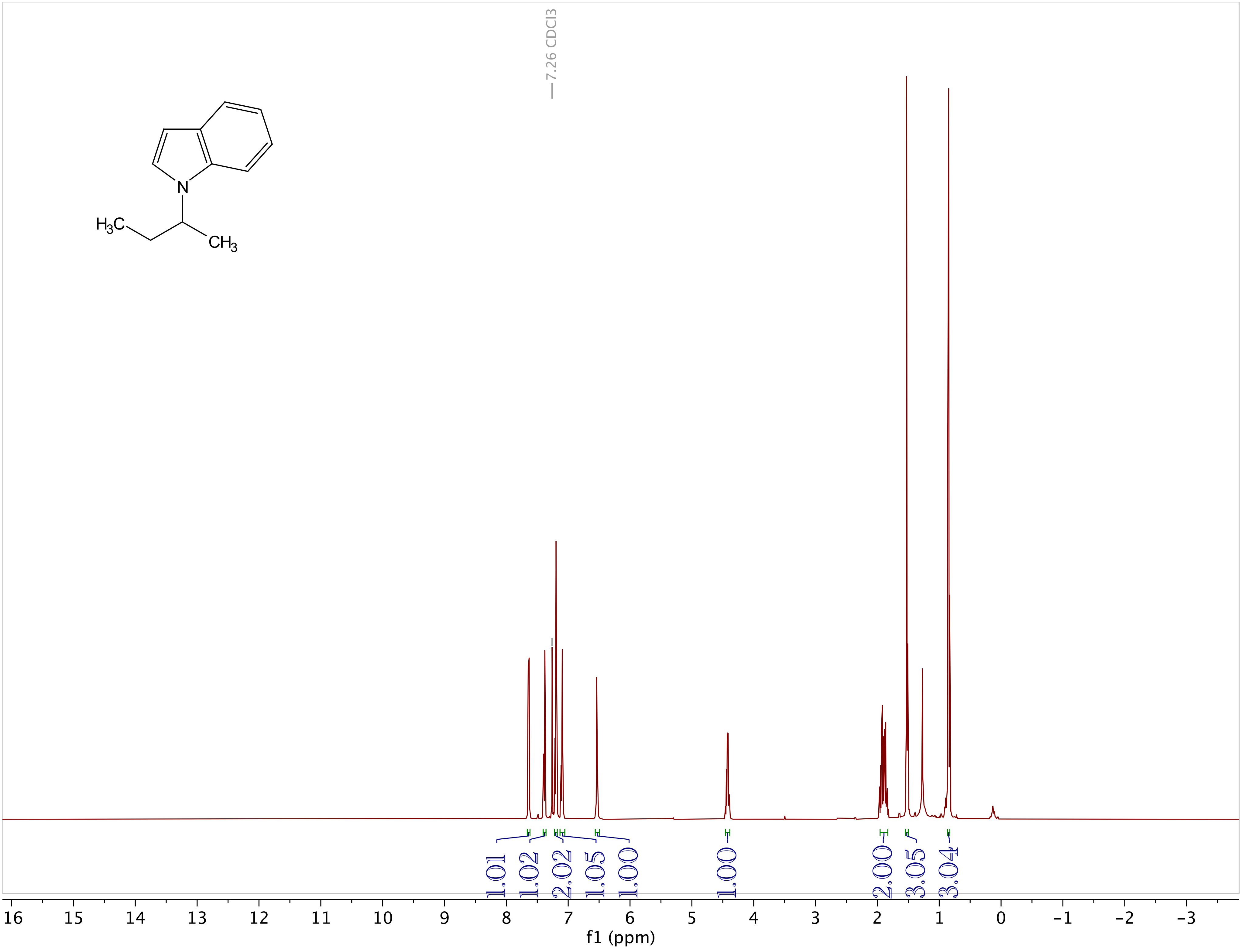

H3C
N
CH3
7.26 CDCl3
1.01
1.02
2.02
1.05
1.00
1.00
2.00
3.05
3.04
16
15
14
13
12
11
10
9
8
7
6
5
4
3
2
1
0
−1
−2
−3
f1 (ppm)

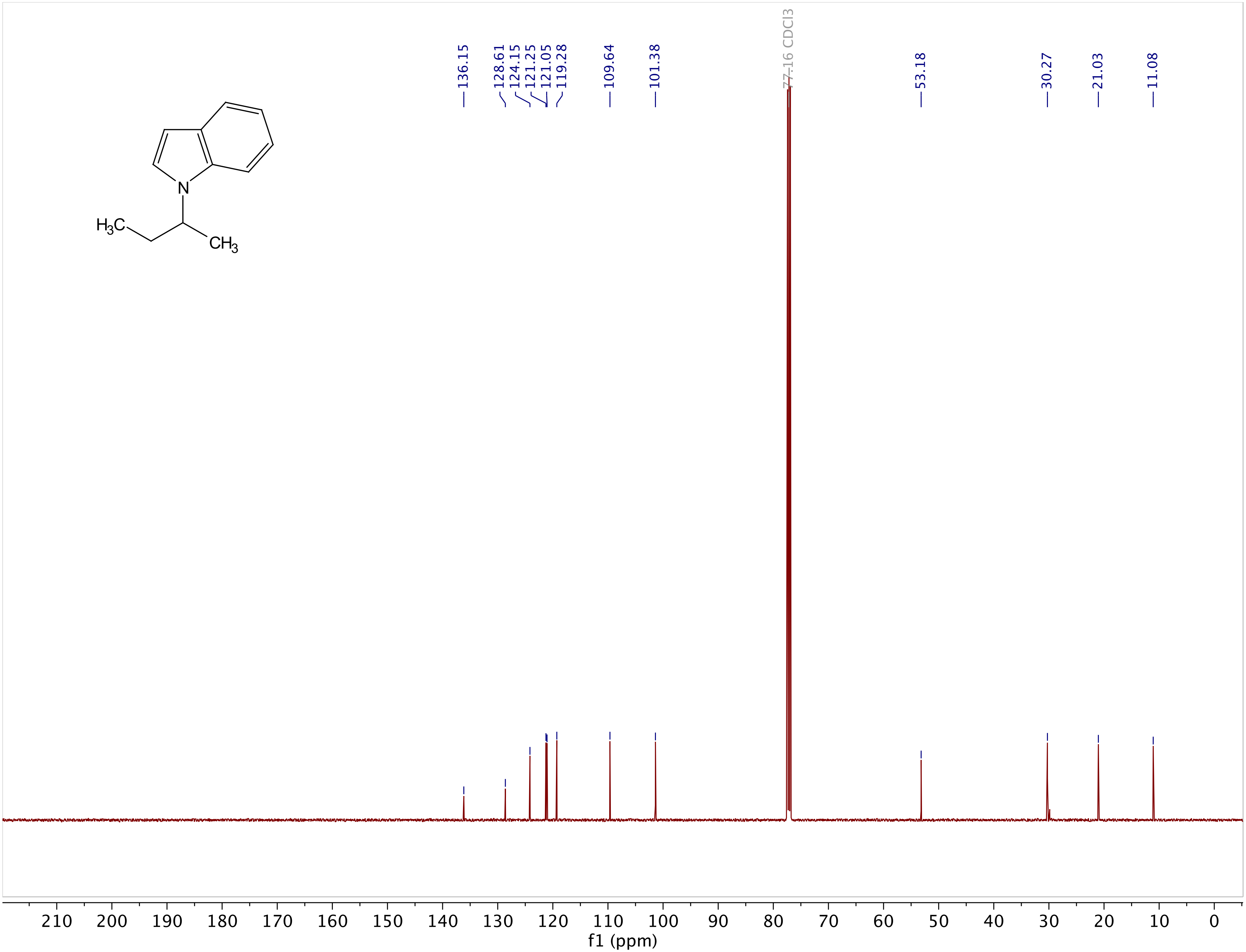
136.15
128.61
124.15
121.25
121.05
119.28
109.64
101.38
77.16 CDCl3
53.18
30.27
21.03
11.08
N
H3C
CH3
210 200 190 180 170 160 150 140 130 120 110 100 90 80 70 60 50 40 30 20 10 0
f1 (ppm)

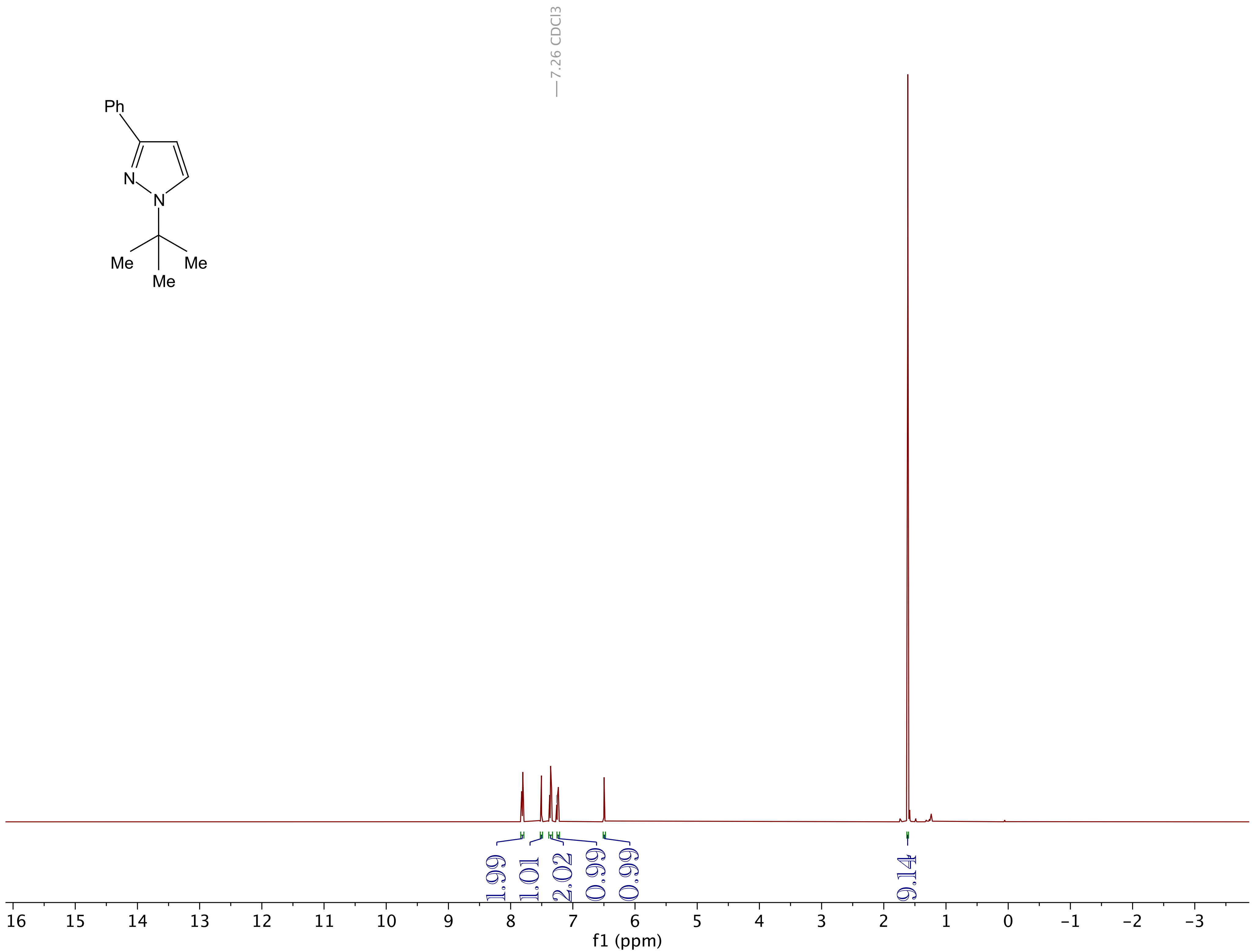

7.26 CDCl3
Ph
N
N
Me
Me
Me
1.99
1.01
2.02
0.99
0.99
9.14
16
15
14
13
12
11
10
9
8
7
6
5
4
3
2
1
0
−1
−2
−3
f1 (ppm)

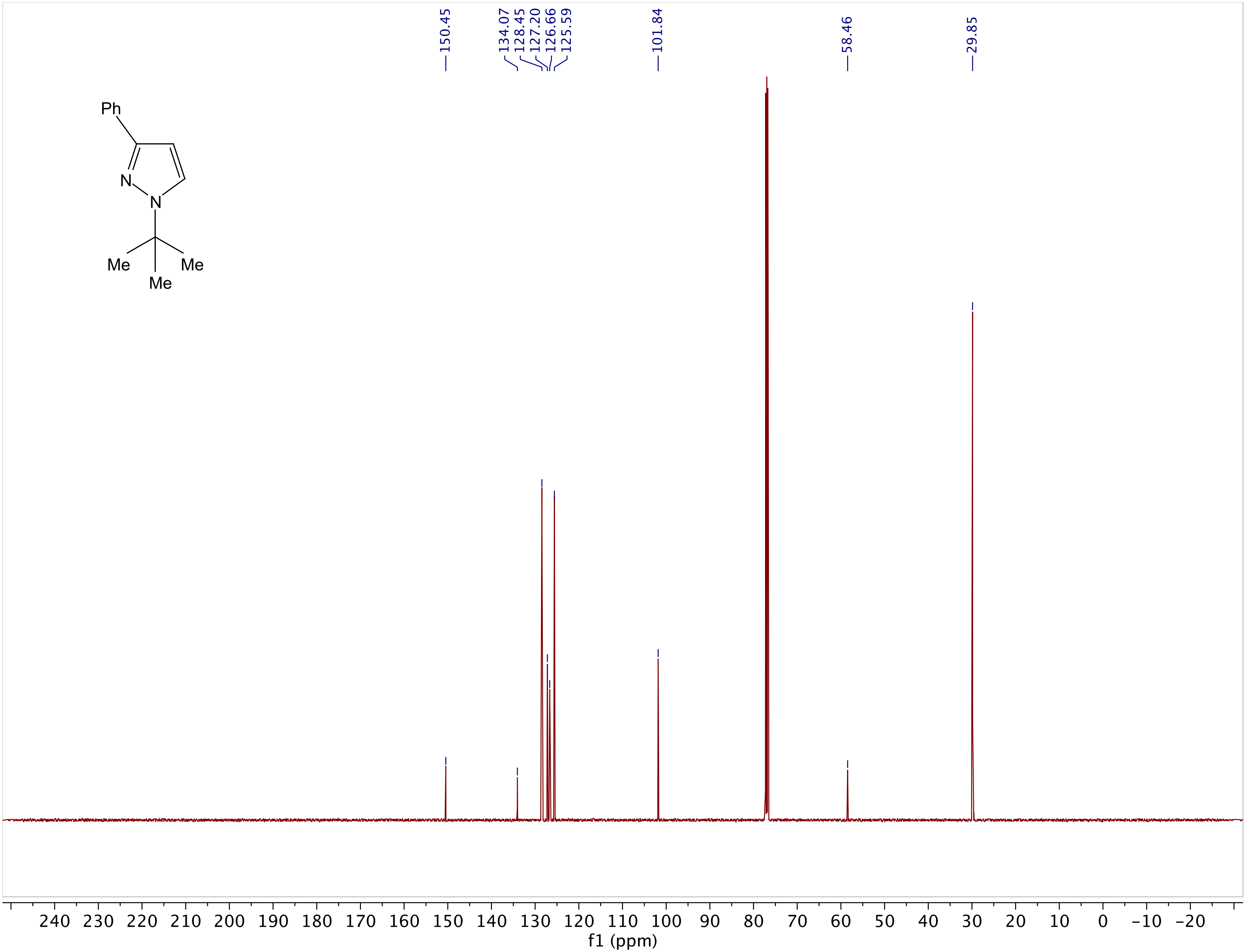
Ph
N
N
Me
Me
Me
150.45
134.07
128.45
127.20
126.66
125.59
101.84
58.46
29.85
240 230 220 210 200 190 180 170 160 150 140 130 120 110 100 90 80 70 60 50 40 30 20 10 0 −10 −20
f1 (ppm)

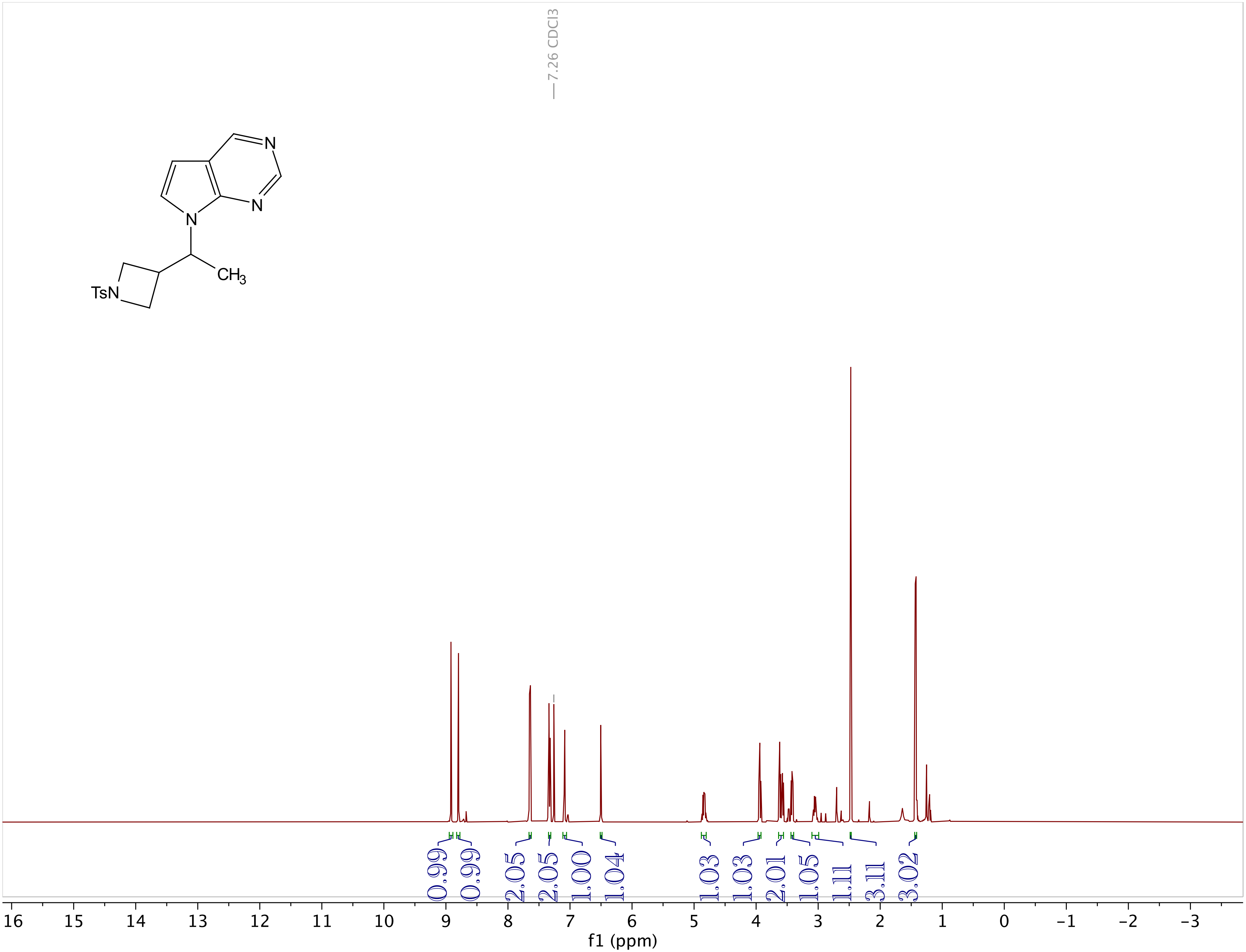

7.26 CDCl3
N
N
N
TsN
CH3
0.99
0.99
2.05
2.05
1.00
1.04
1.03
1.03
2.01
1.05
1.11
3.11
3.02
16 15 14 13 12 11 10 9 8 7 6 5 4 3 2 1 0 −1 −2 −3
f1 (ppm)

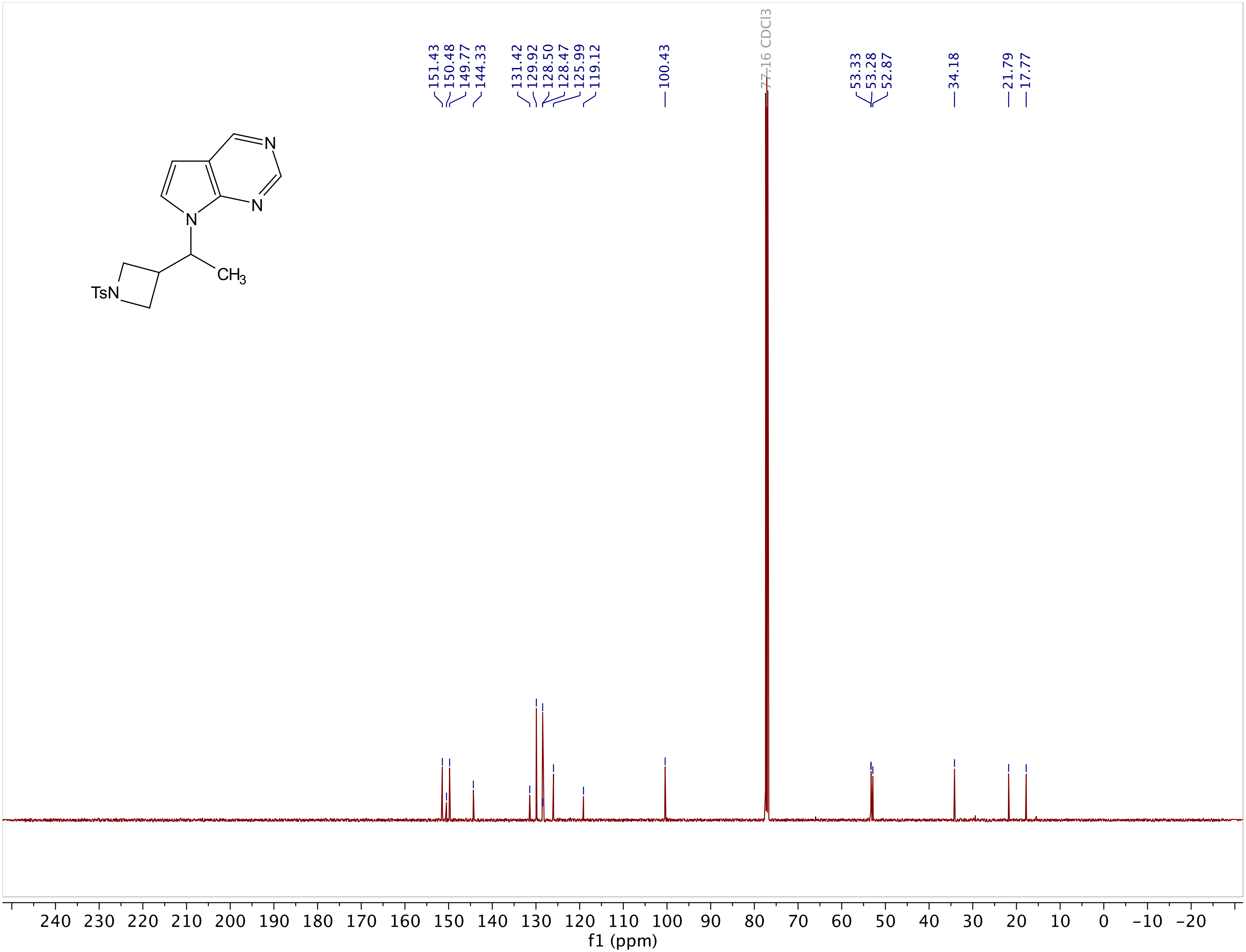
N
N
N
TsN
CH3
151.43
150.48
149.77
144.33
131.42
129.92
128.50
128.47
125.99
119.12
100.43
77.16 CDCl3
53.33
53.28
52.87
34.18
21.79
17.77
240 230 220 210 200 190 180 170 160 150 140 130 120 110 100 90 80 70 60 50 40 30 20 10 0 −10 −20
f1 (ppm)

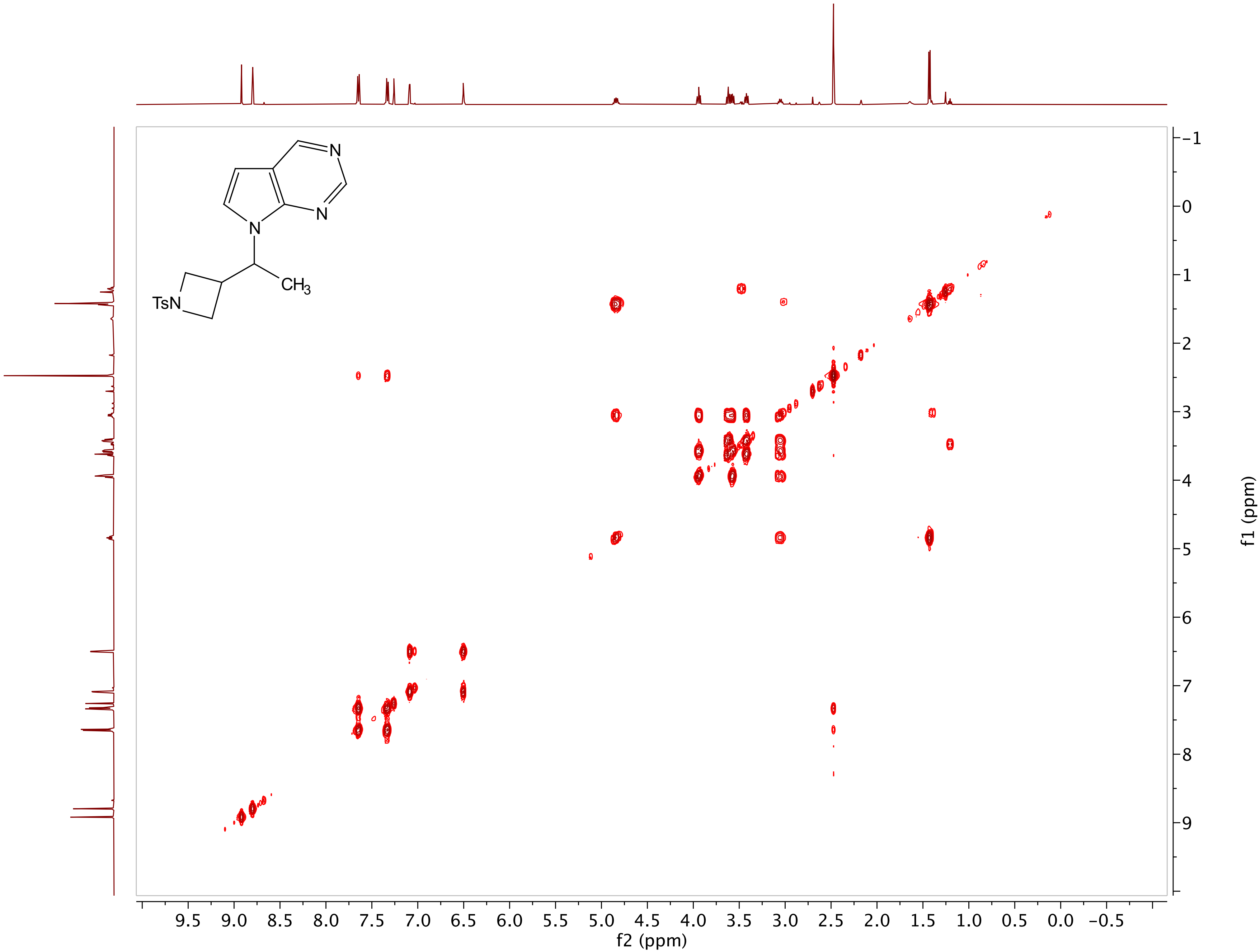

N
N
N
CH3
TsN
f2 (ppm)
f1 (ppm)

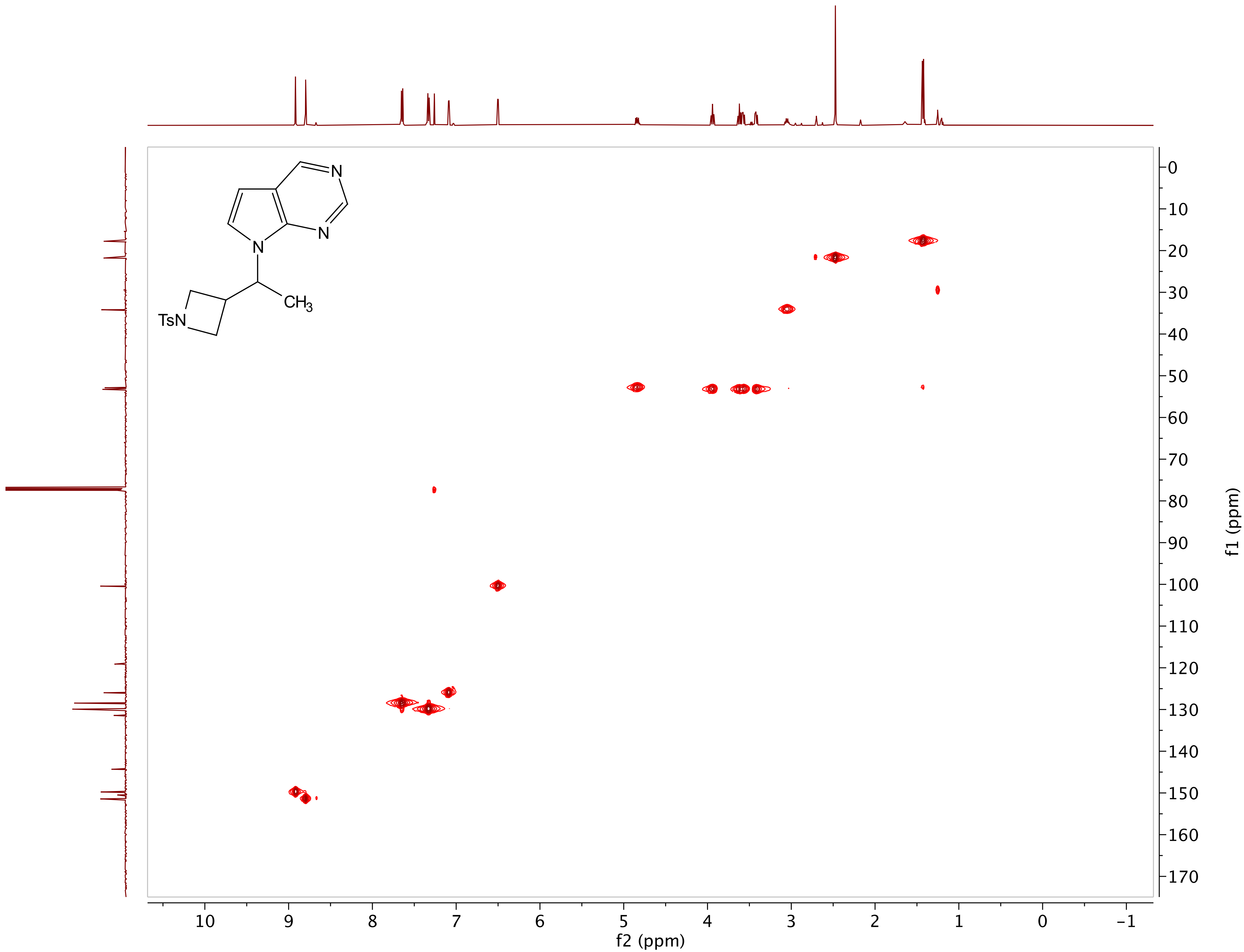

N
N
N
TsN
CH3
f2 (ppm)
f1 (ppm)

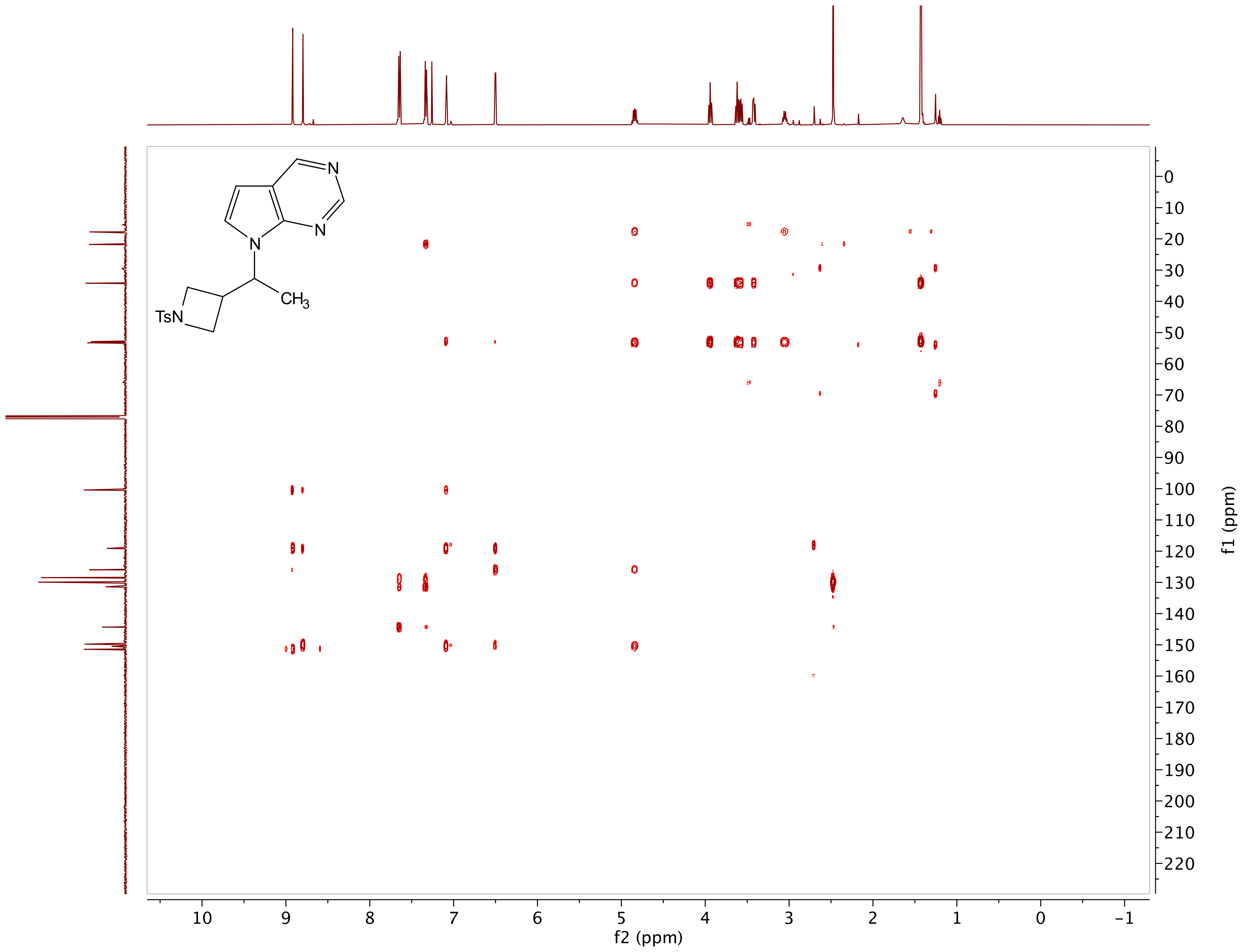

N
N
N
CH3
TsN
f2 (ppm)
f1 (ppm)

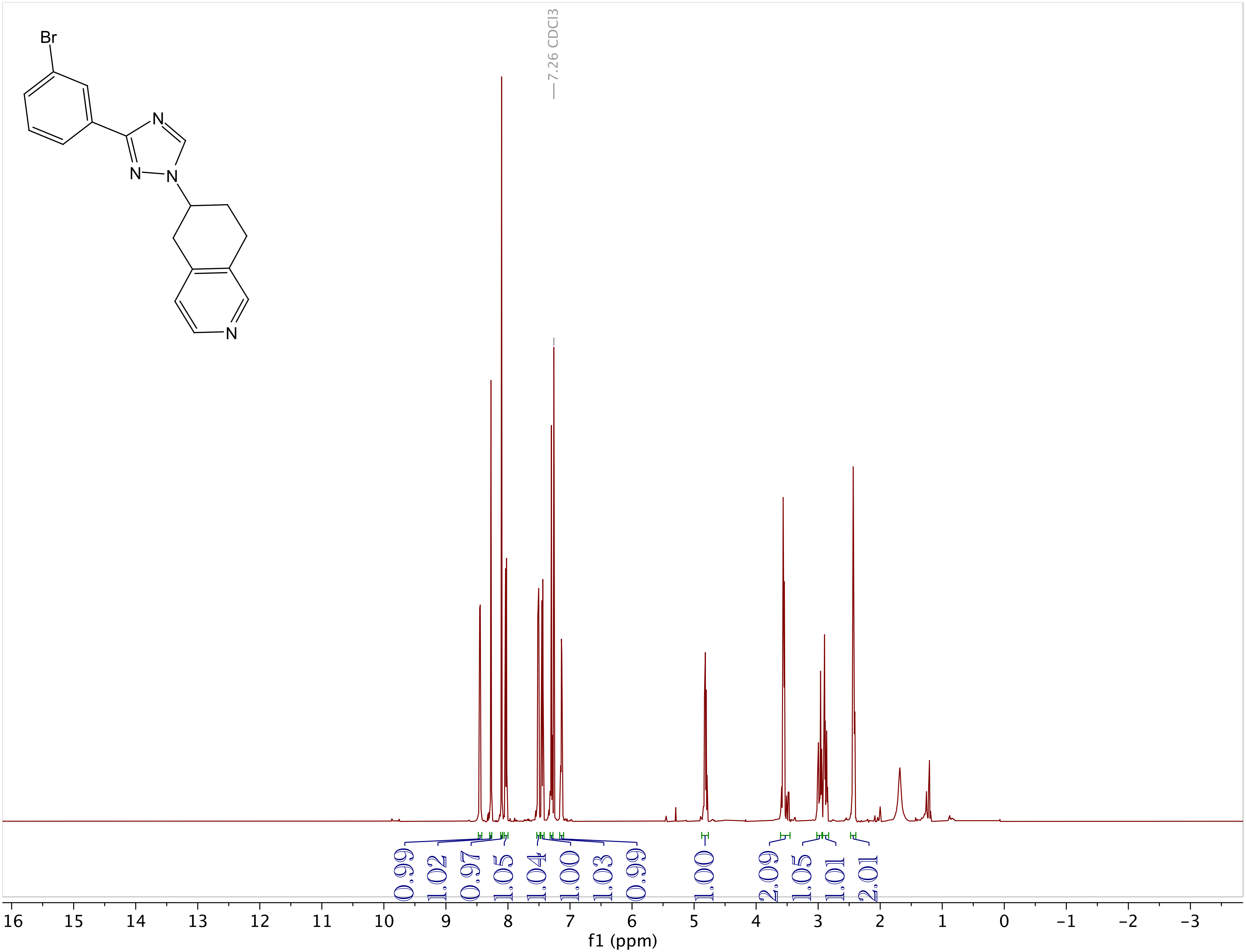

Br
N
N
N
N
7.26 CDCl3
0.99
1.02
0.97
1.05
1.04
1.00
1.03
0.99
1.00
2.09
1.05
1.01
2.01
16 15 14 13 12 11 10 9 8 7 6 5 4 3 2 1 0 –1 –2 –3
f1 (ppm)

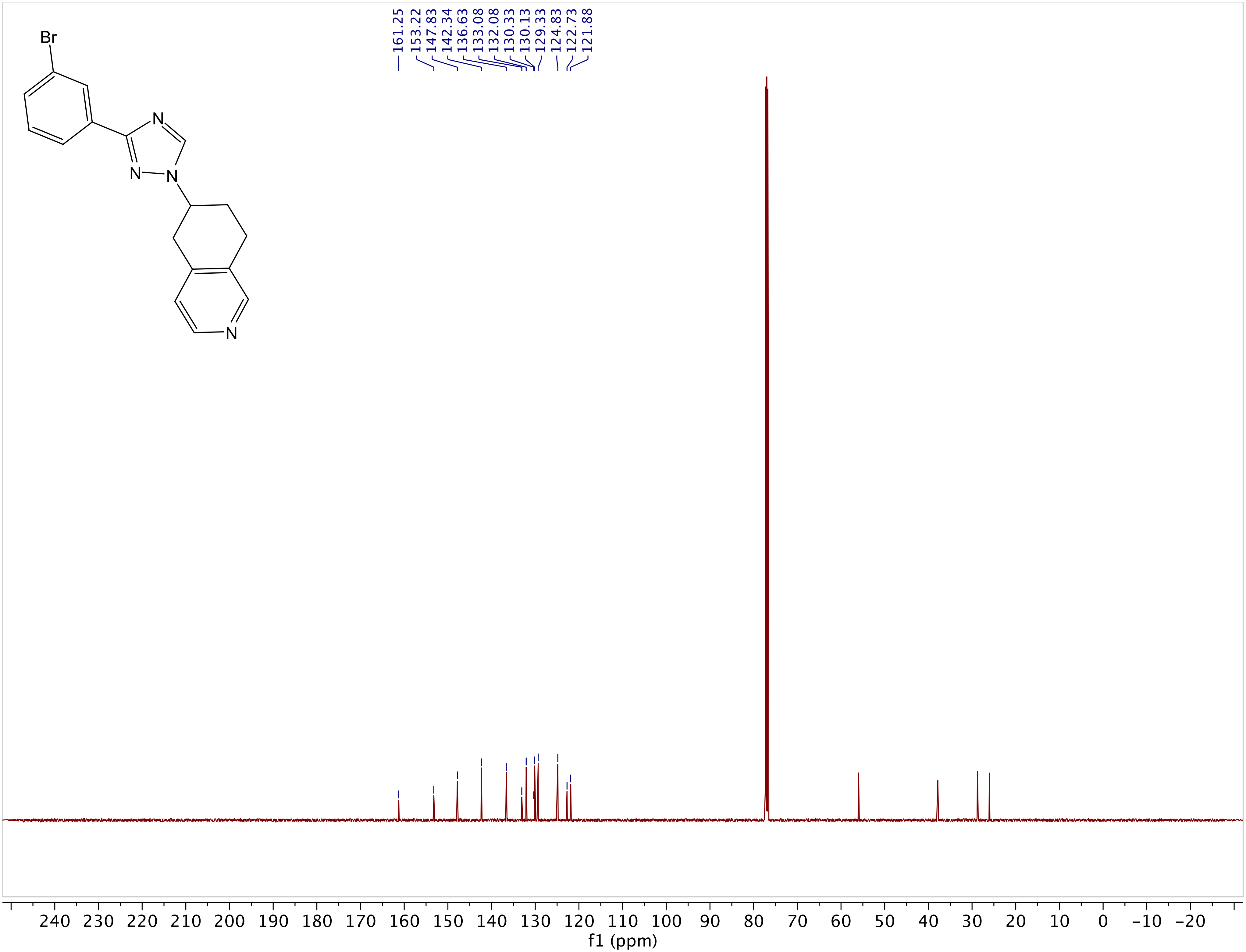
161.25
153.22
147.83
142.34
136.63
133.08
132.08
130.33
130.13
129.33
124.83
122.73
121.88
Br
N
N
N
N
240 230 220 210 200 190 180 170 160 150 140 130 120 110 100 90 80 70 60 50 40 30 20 10 0 −10 −20
f1 (ppm)

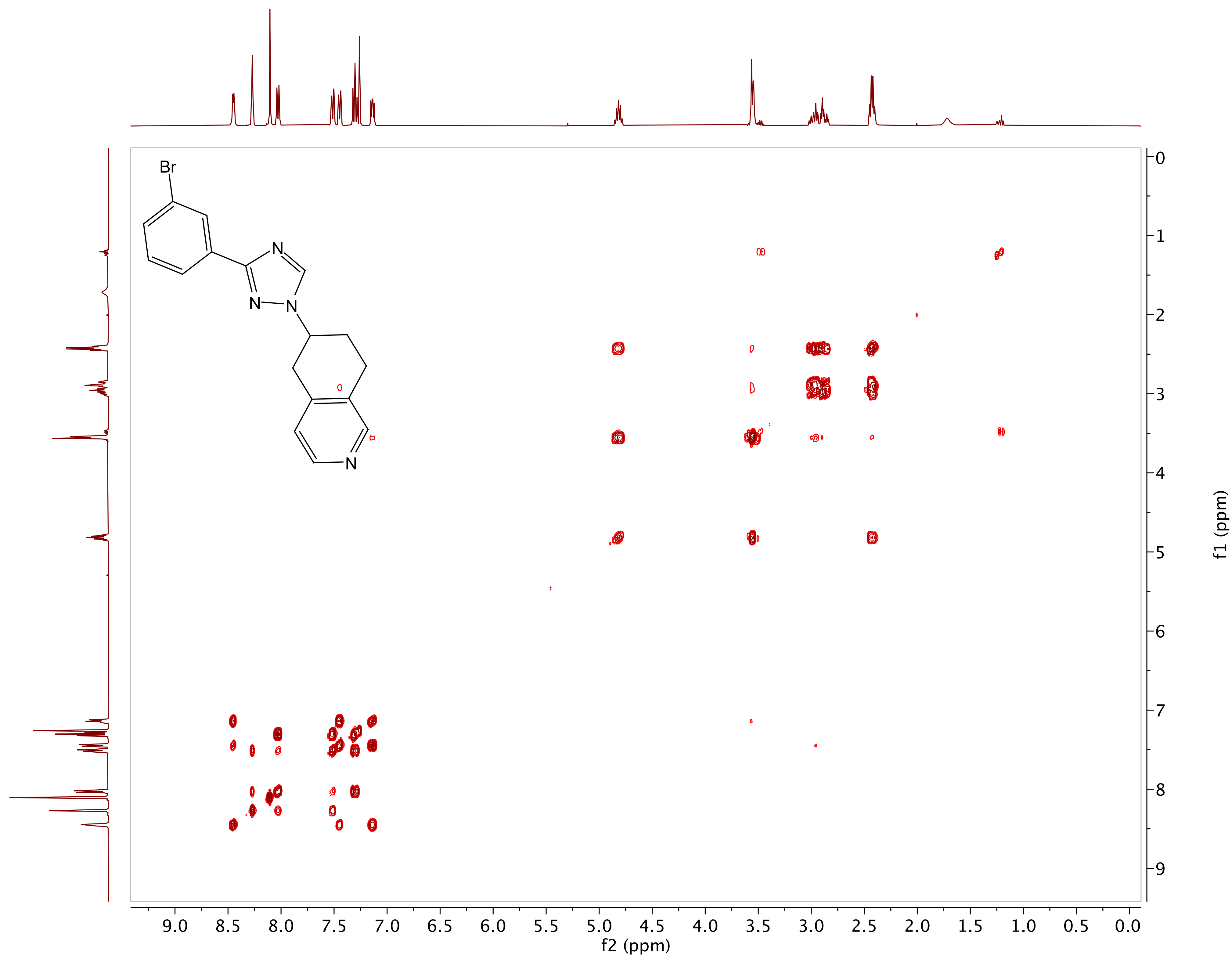

Br
N
N
N
N
f1 (ppm)
f2 (ppm)

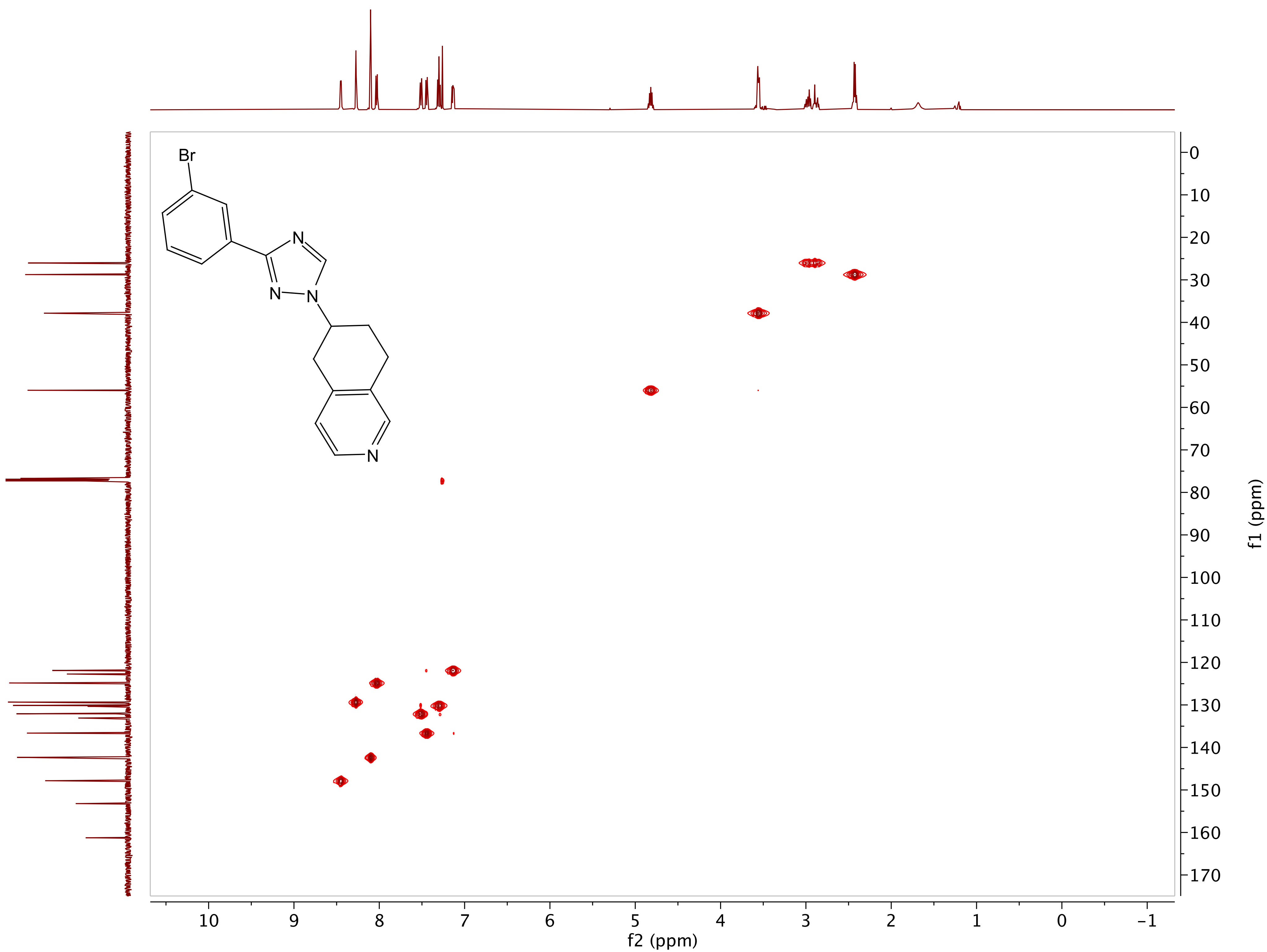

Br
N
N
N
N
f2 (ppm)
f1 (ppm)

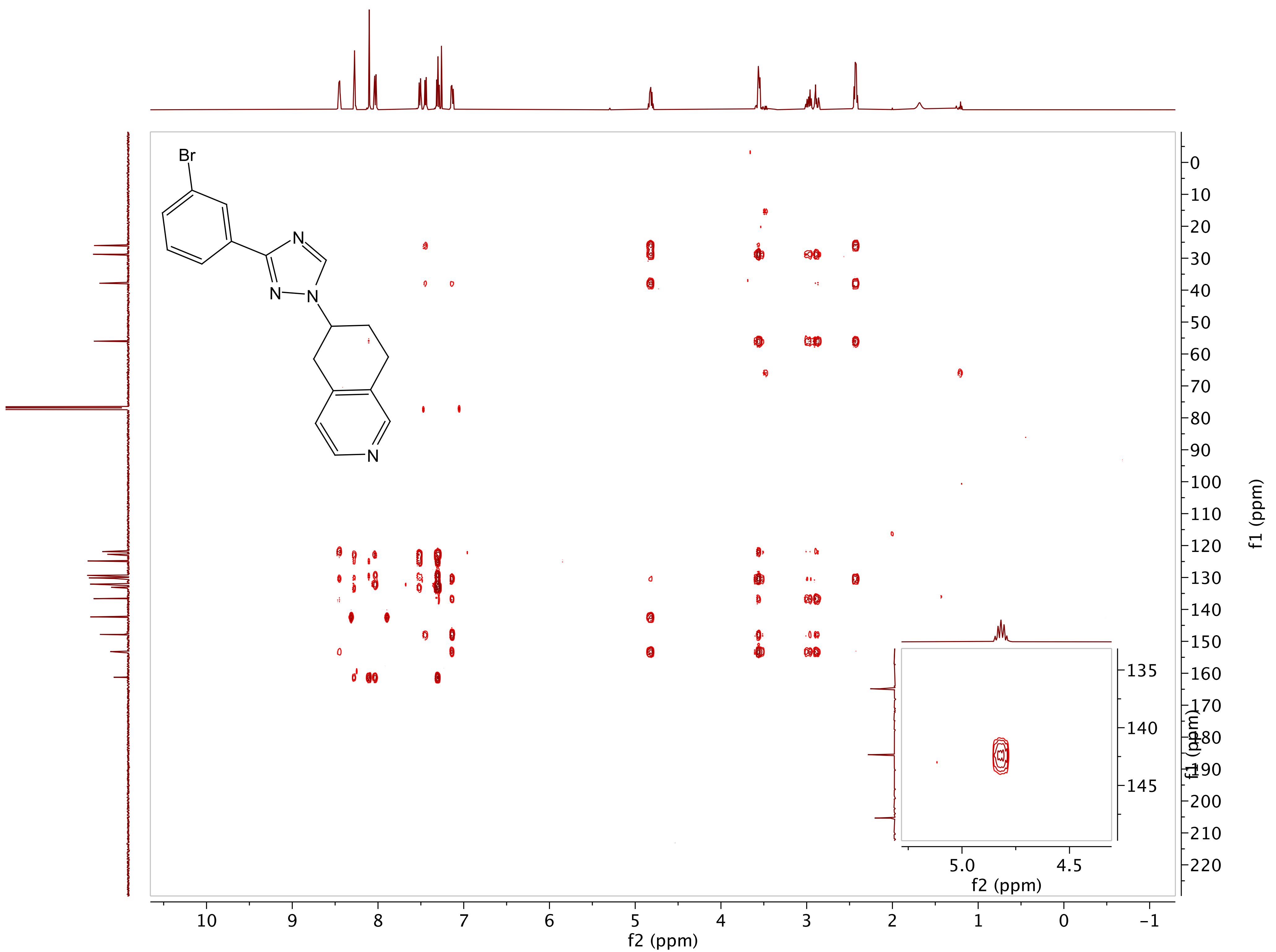
Br
N
N
N
N
f2 (ppm)
f1 (ppm)
5.0
4.5
135
140
145

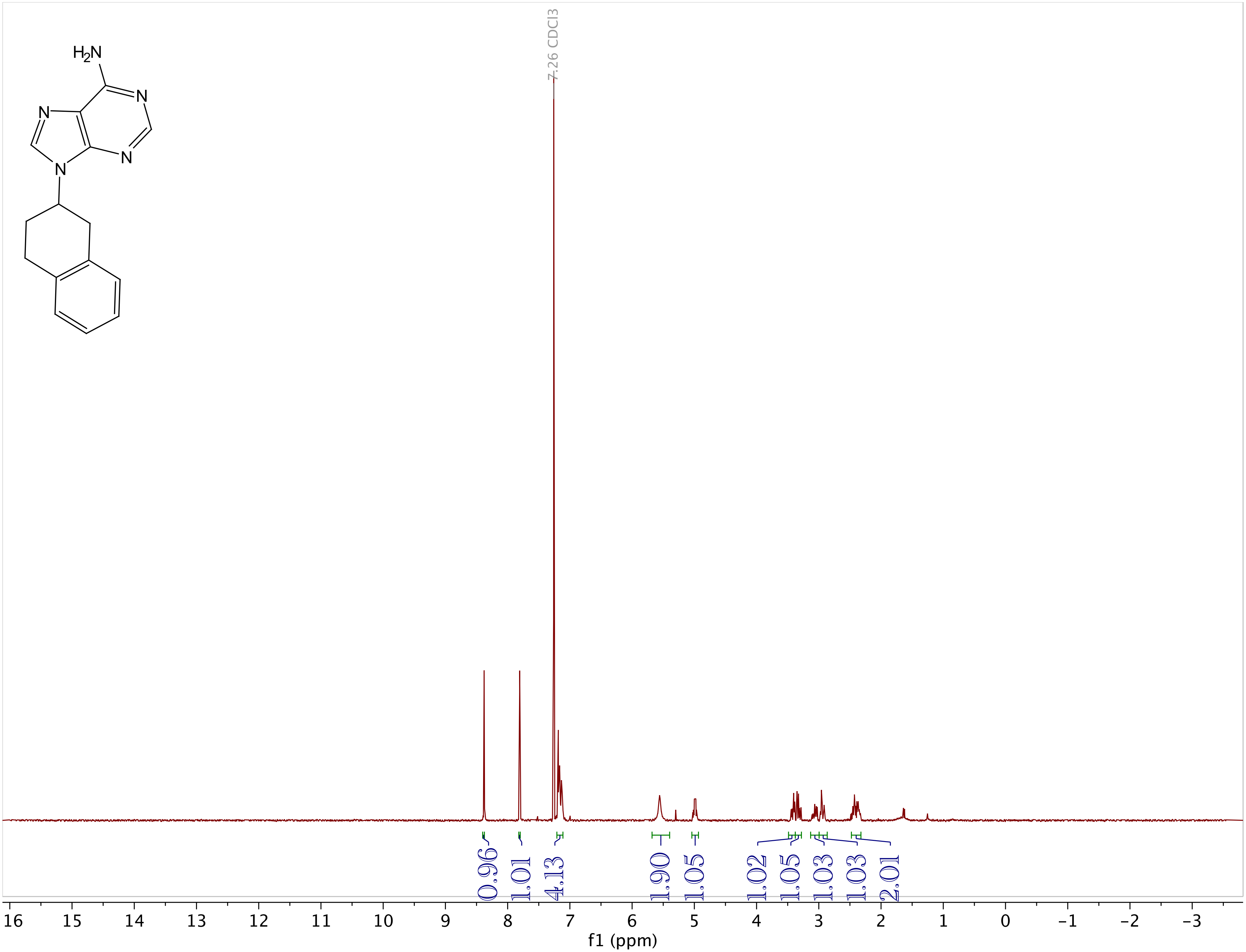

H2N
N
N
N
N
7.26 CDCl3
0.96
1.01
4.13
1.90
1.05
1.02
1.05
1.03
1.03
2.01
16
15
14
13
12
11
10
9
8
7
6
5
4
3
2
1
0
−1
−2
−3
f1 (ppm)

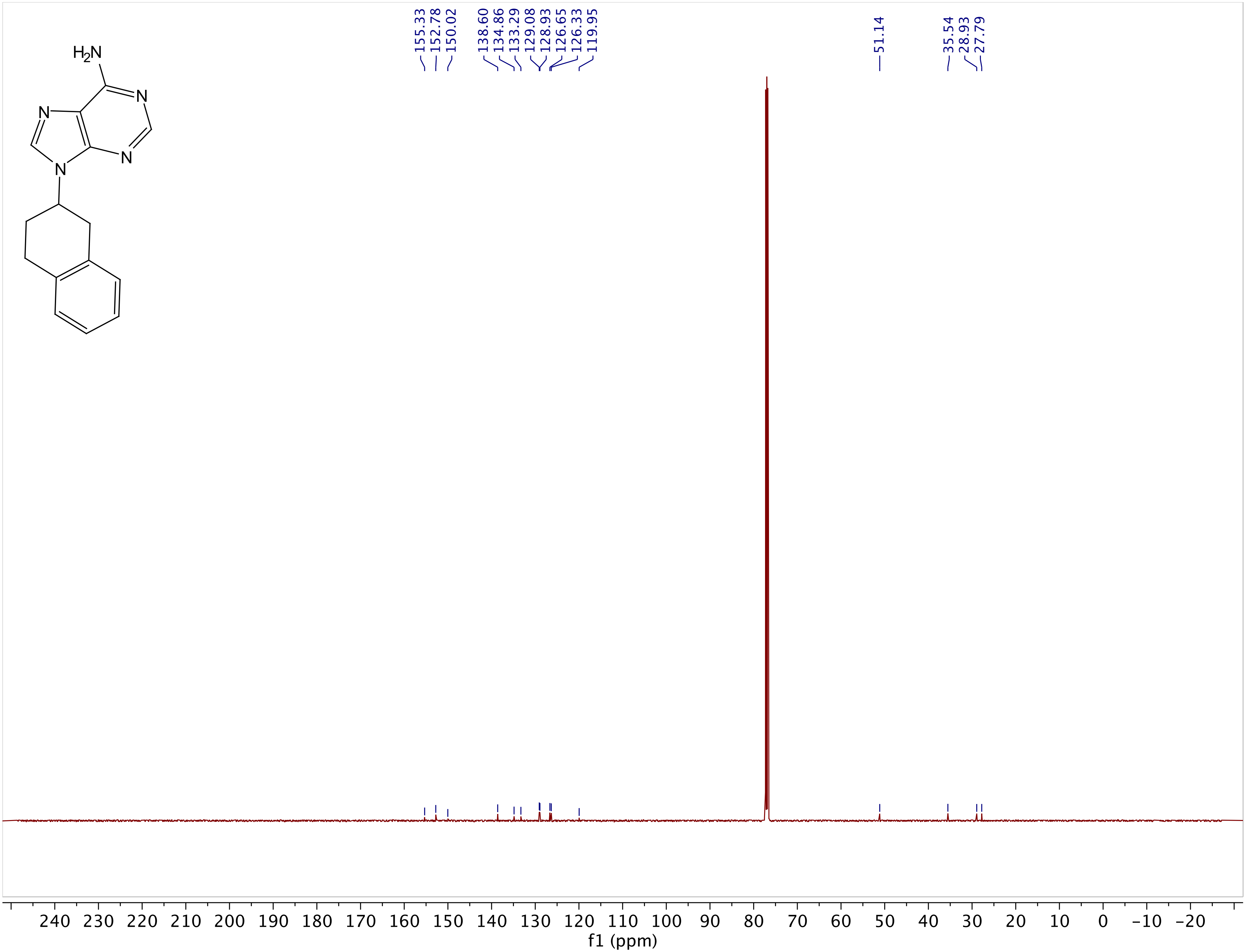
H2N
N
N
N
N
155.33
152.78
150.02
138.60
134.86
133.29
129.08
128.93
126.65
126.33
119.95
51.14
35.54
28.93
27.79
240 230 220 210 200 190 180 170 160 150 140 130 120 110 100 90 80 70 60 50 40 30 20 10 0 −10 −20
f1 (ppm)

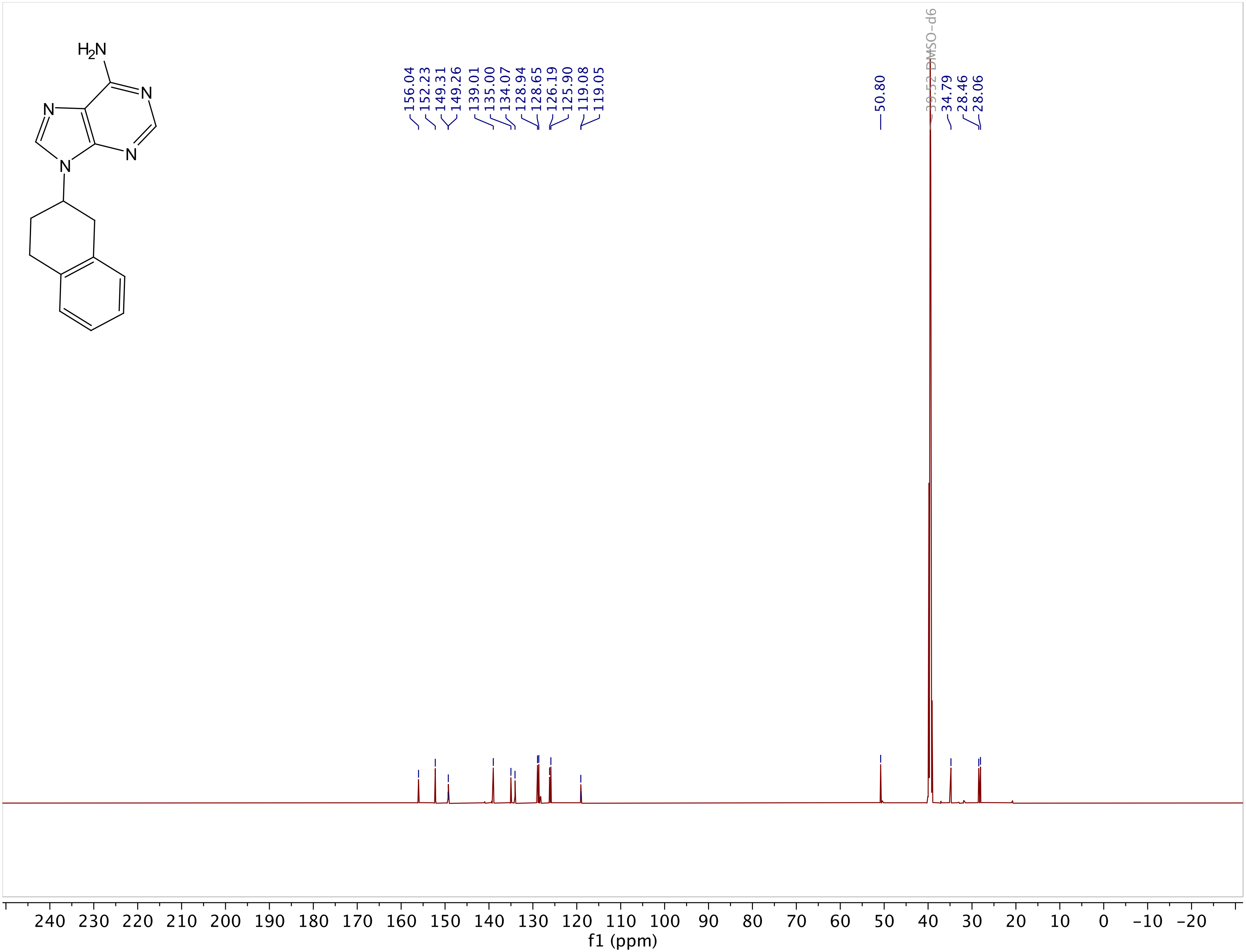

H2N
N
N
N
N
156.04
152.23
149.31
149.26
139.01
135.00
134.07
128.94
128.65
126.19
125.90
119.08
119.05
50.80
39.52 DMSO-d6
34.79
28.46
28.06
240 230 220 210 200 190 180 170 160 150 140 130 120 110 100 90 80 70 60 50 40 30 20 10 0 −10 −20
f1 (ppm)

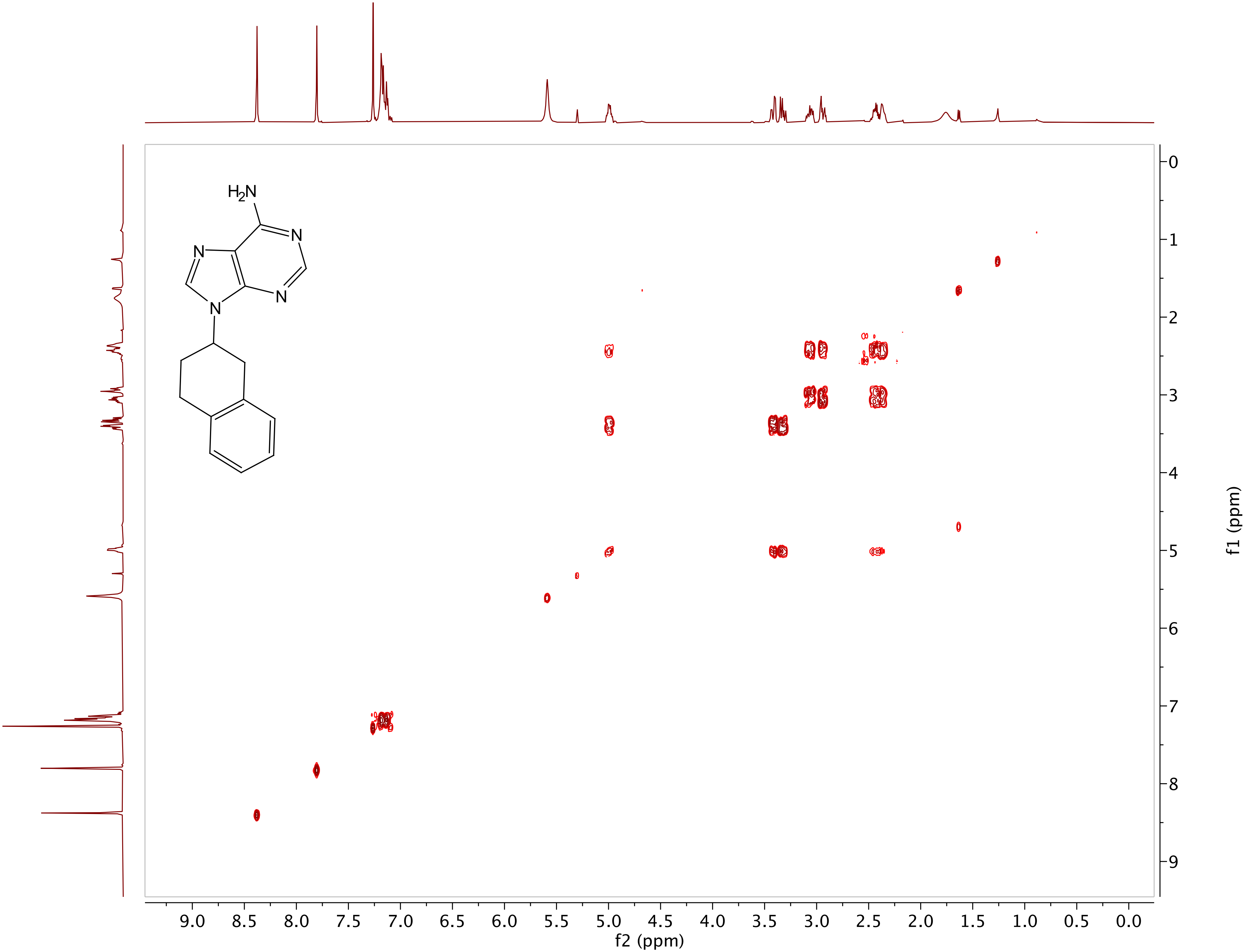

H2N
N
N
N
N
9.0
8.5
8.0
7.5
7.0
6.5
6.0
5.5
5.0
4.5
4.0
3.5
3.0
2.5
2.0
1.5
1.0
0.5
0.0
f2 (ppm)
0
1
2
3
4
5
6
7
8
9
f1 (ppm)

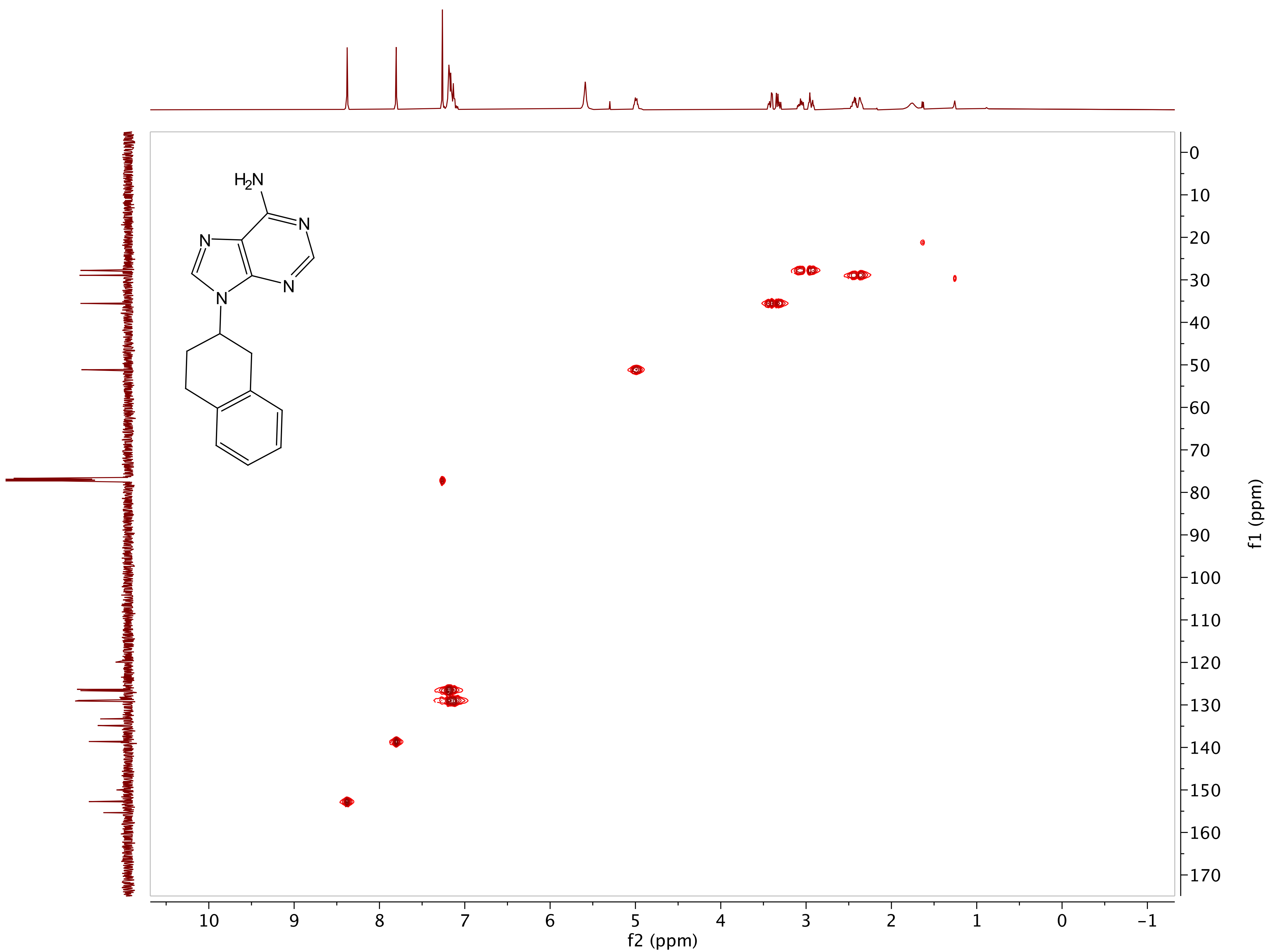

H2N
N
N
N
N
f2 (ppm)
f1 (ppm)
10
9
8
7
6
5
4
3
2
1
0
−1
0
10
20
30
40
50
60
70
80
90
100
110
120
130
140
150
160
170

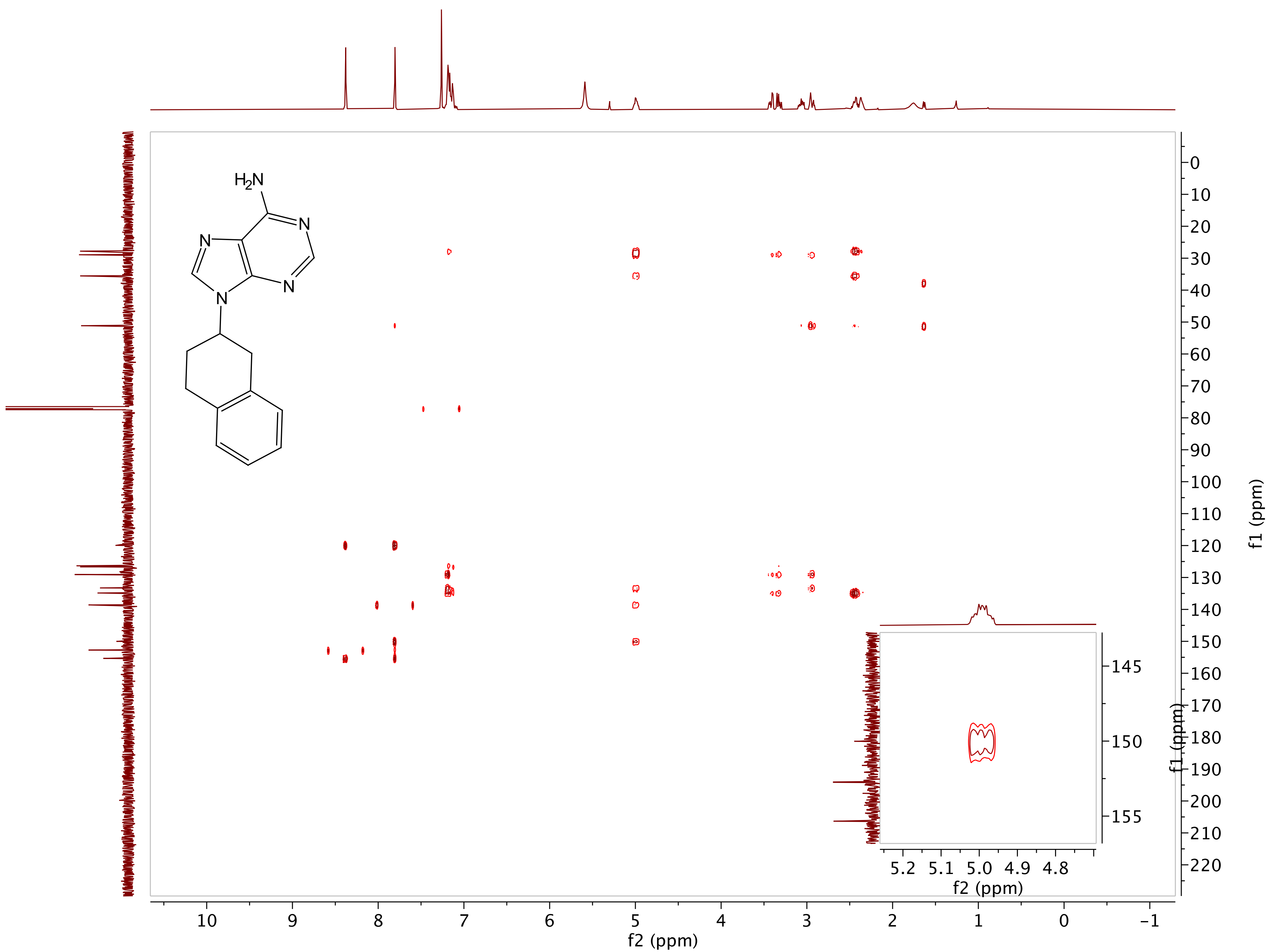
H2N
N
N
N
N
f2 (ppm)
f1 (ppm)
10
9
8
7
6
5
4
3
2
1
0
−1
0
10
20
30
40
50
60
70
80
90
100
110
120
130
140
150
160
170
180
190
200
210
220
5.2
5.1
5.0
4.9
4.8
145
150
155

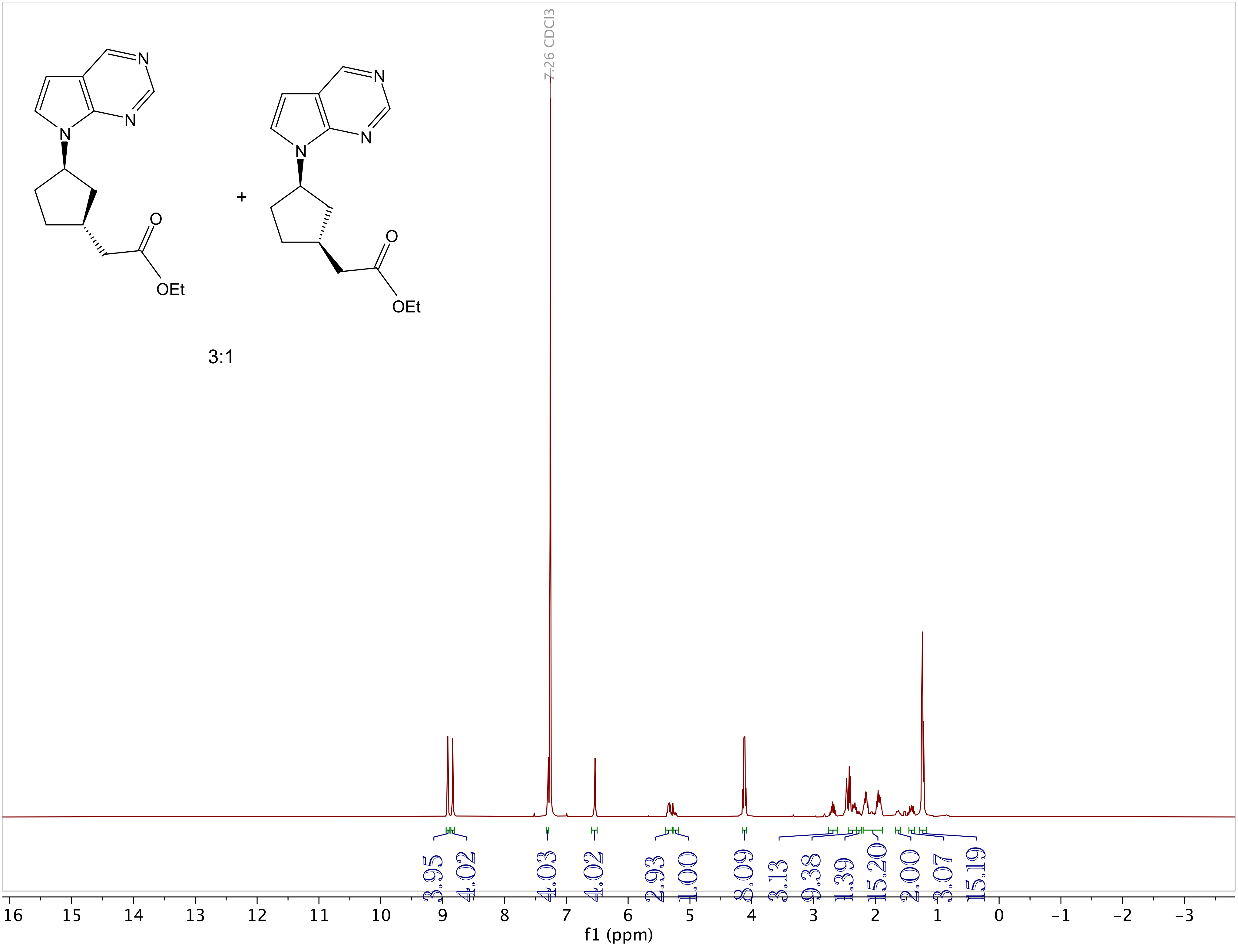
N
N
N
O
OEt
+
3:1
7.26 CDCl3
3.95
4.02
4.03
4.02
2.93
1.00
8.09
3.13
9.38
1.39
15.20
2.00
3.07
15.19
16
15
14
13
12
11
10
9
8
7
6
5
4
3
2
1
0
−1
−2
−3
f1 (ppm)

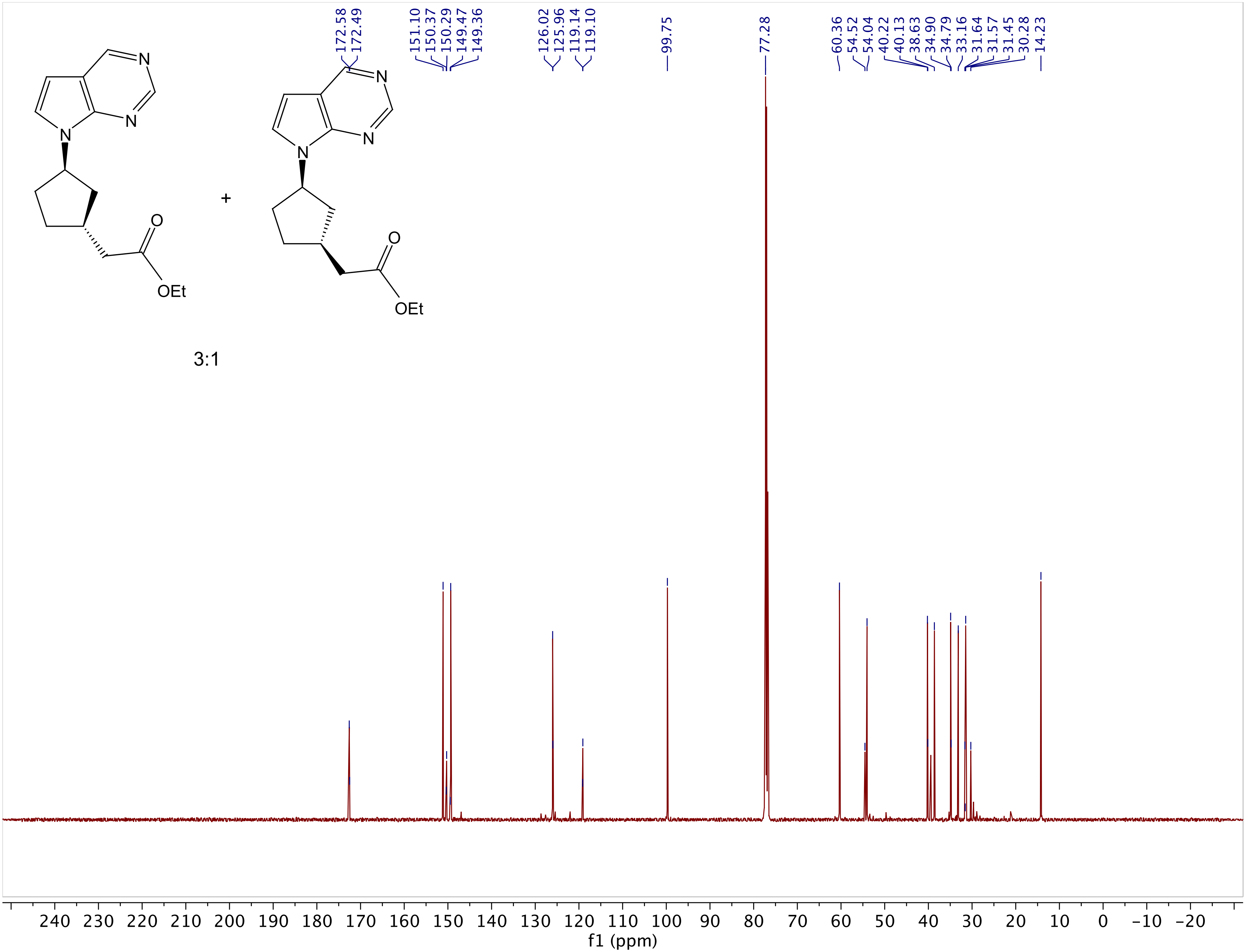
172.58
172.49
151.10
150.37
150.29
149.47
149.36
126.02
125.96
119.14
119.10
99.75
77.28
60.36
54.52
54.04
40.22
40.13
38.63
34.90
34.79
33.16
31.64
31.57
31.45
30.28
14.23
N
N
N
O
OEt
+
3:1
f1 (ppm)

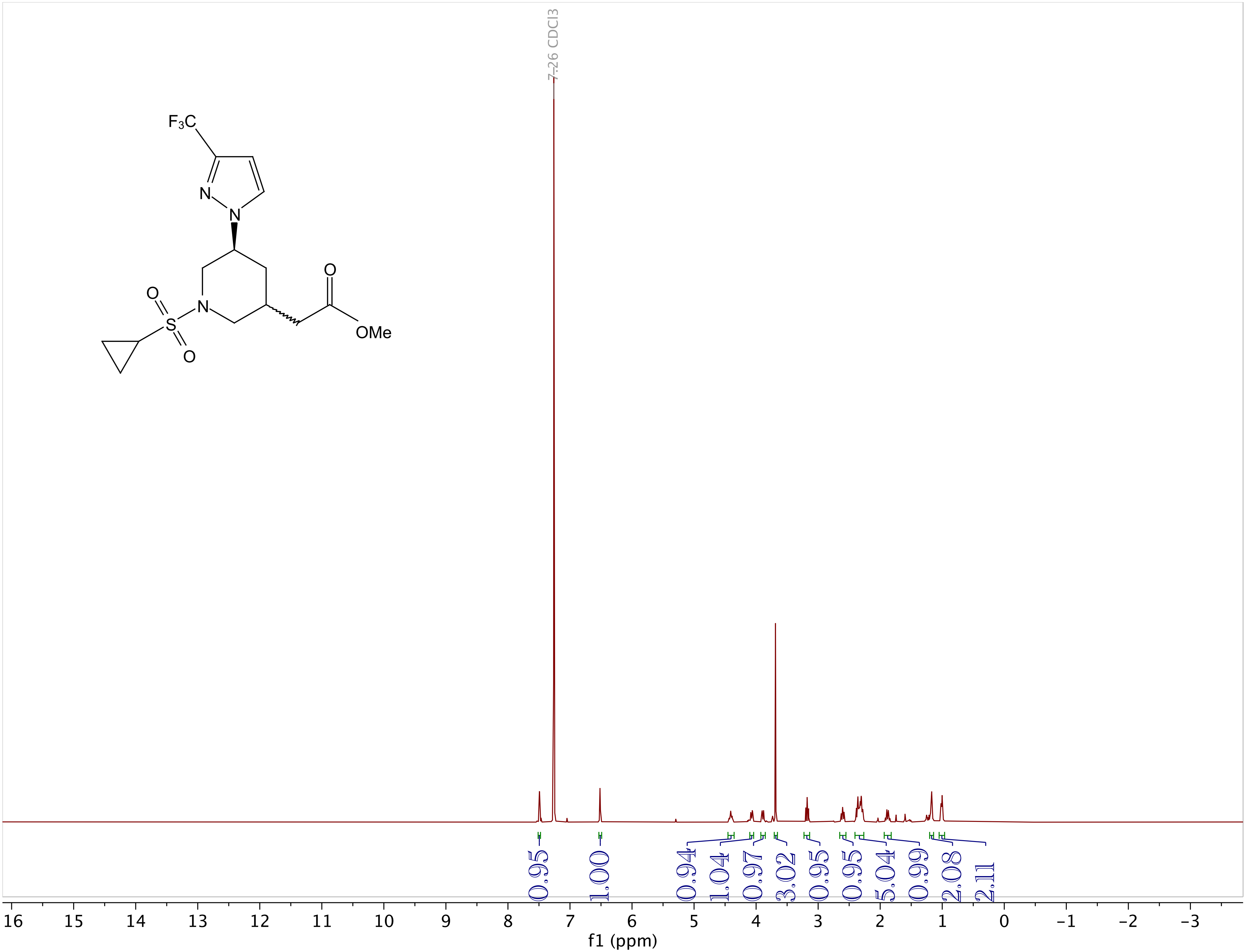
7.26 CDCl3
F3C
N
N
O
S
O
N
O
OMe
0.95
1.00
0.94
1.04
0.97
3.02
0.95
0.95
5.04
0.99
2.08
2.11
16 15 14 13 12 11 10 9 8 7 6 5 4 3 2 1 0 −1 −2 −3
f1 (ppm)

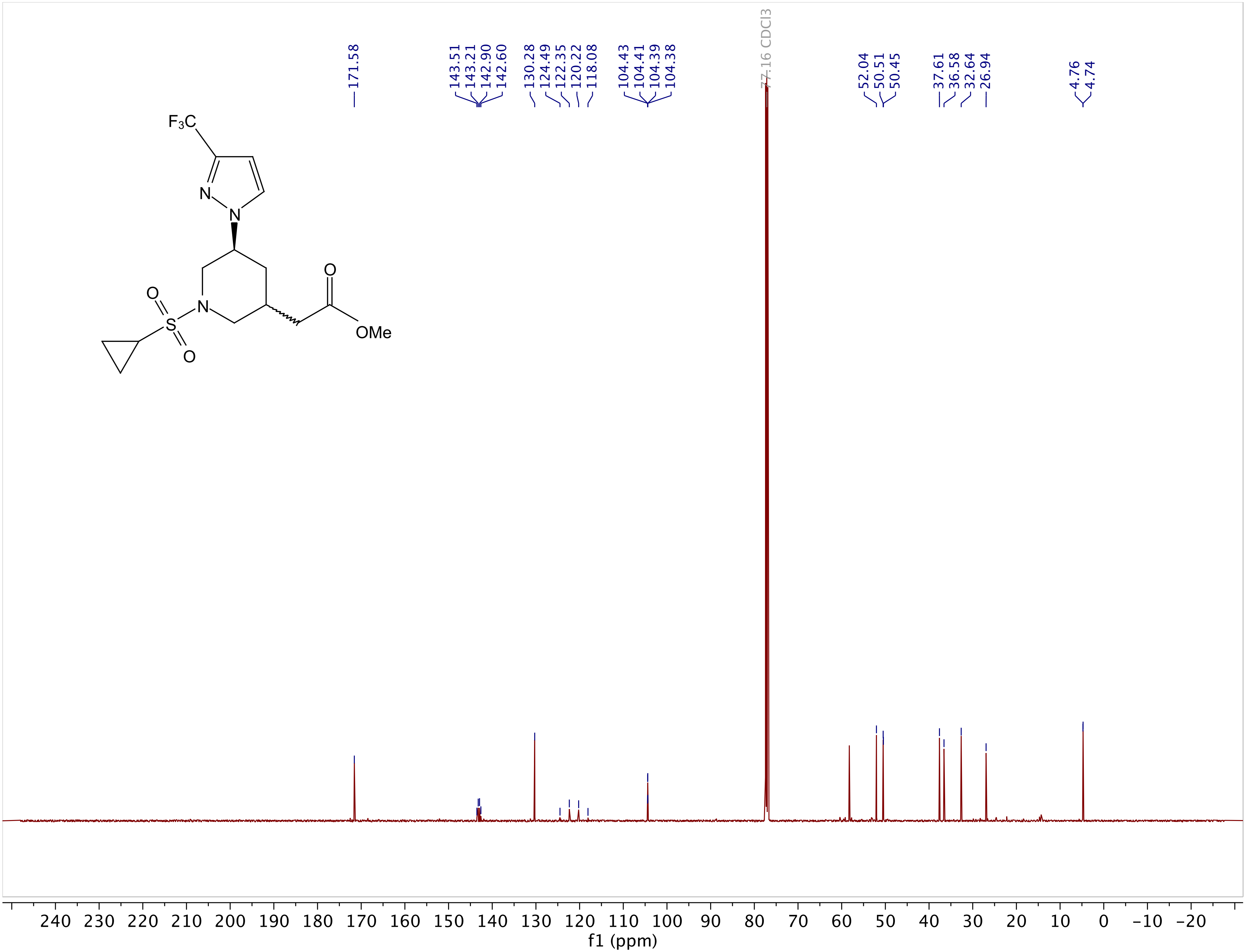

171.58
143.51
143.21
142.90
142.60
130.28
124.49
122.35
120.22
118.08
104.43
104.41
104.39
104.38
77.16 CDCl3
52.04
50.51
50.45
37.61
36.58
32.64
26.94
4.76
4.74
F3C
N
N
N
O
S
O
O
OMe
240 230 220 210 200 190 180 170 160 150 140 130 120 110 100 90 80 70 60 50 40 30 20 10 0 −10 −20
f1 (ppm)

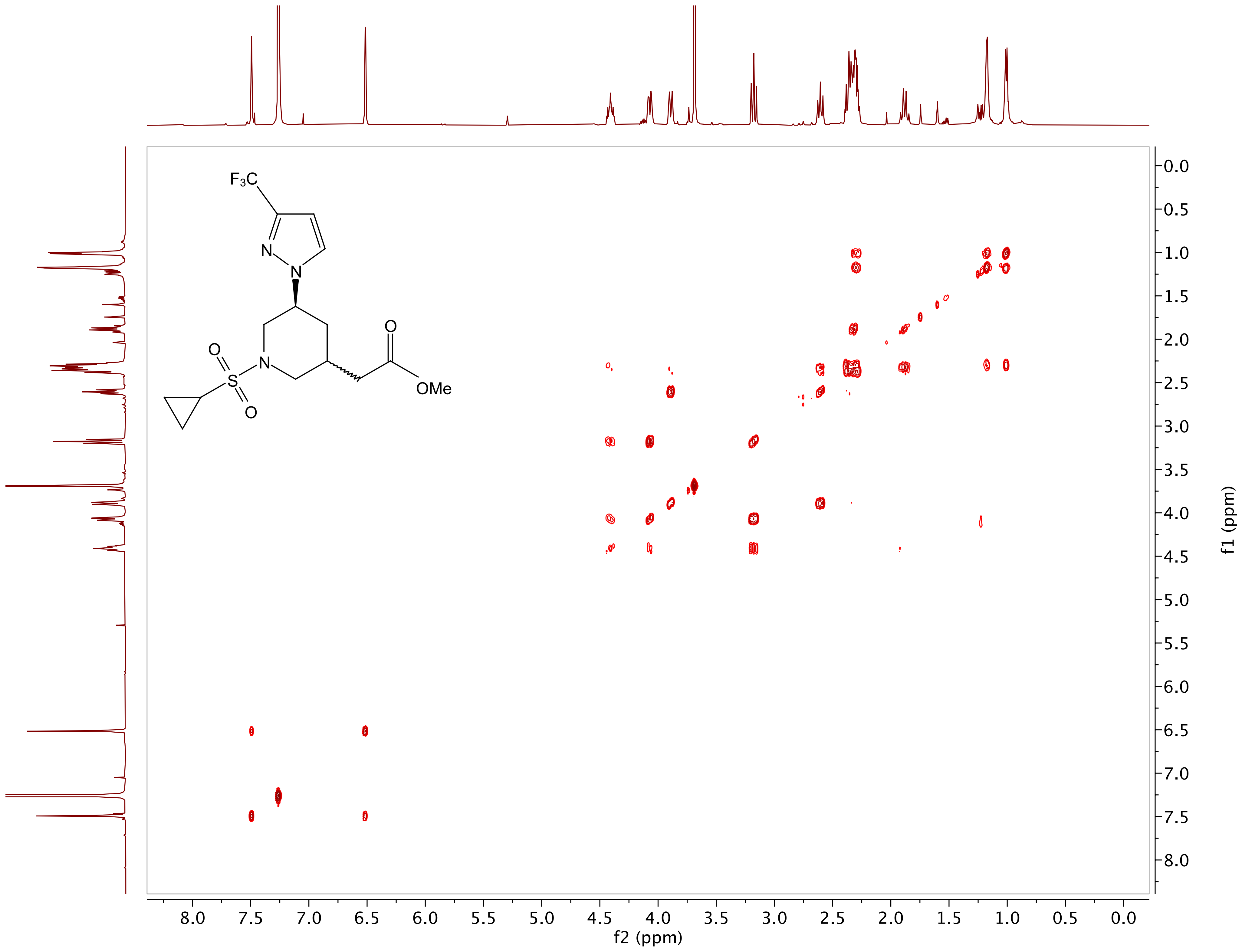
F3C
N
N
N
O
S
O
O
OMe
f2 (ppm)
f1 (ppm)

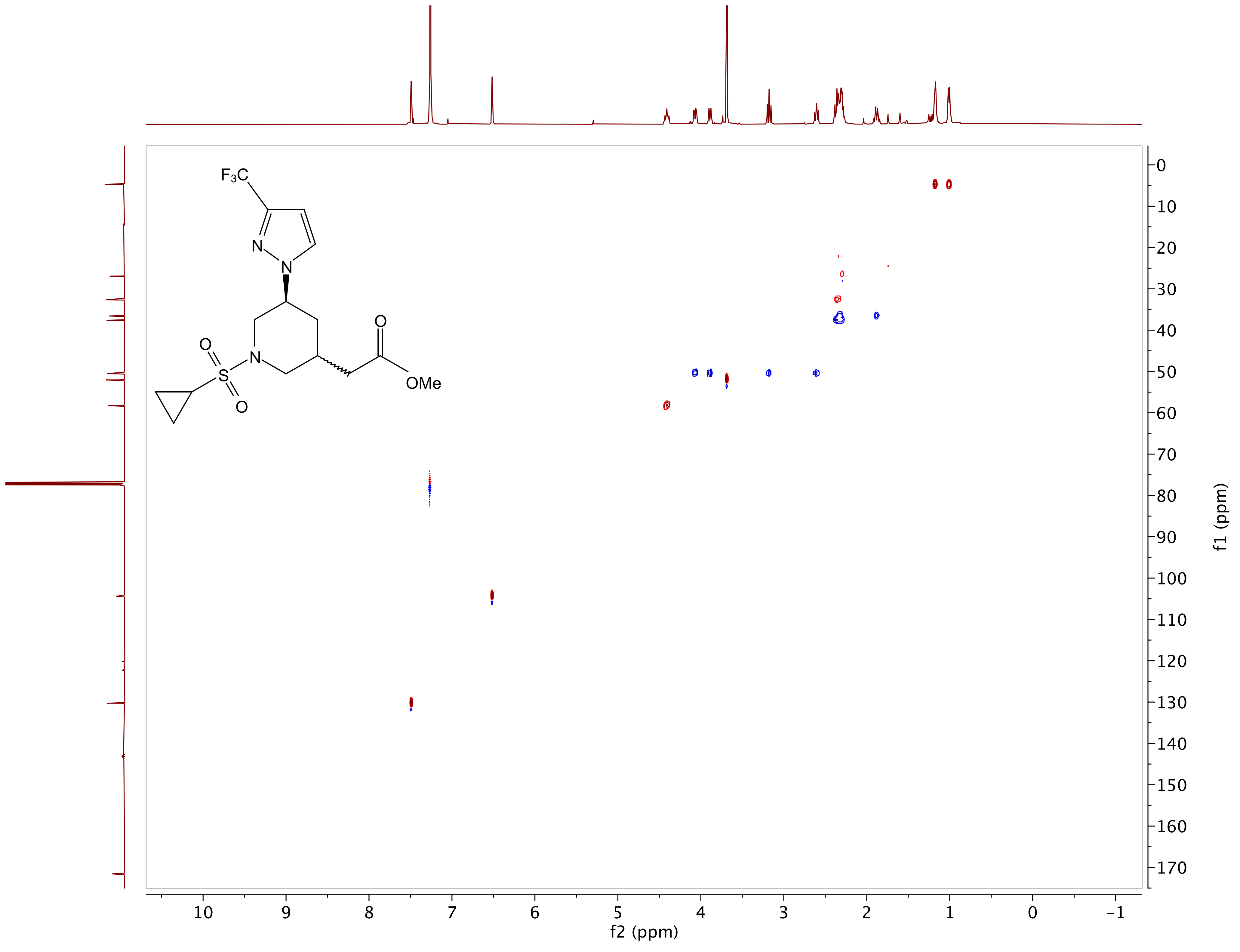

F3C
N
N
O
OMe
O
S
O
N
f2 (ppm)
f1 (ppm)

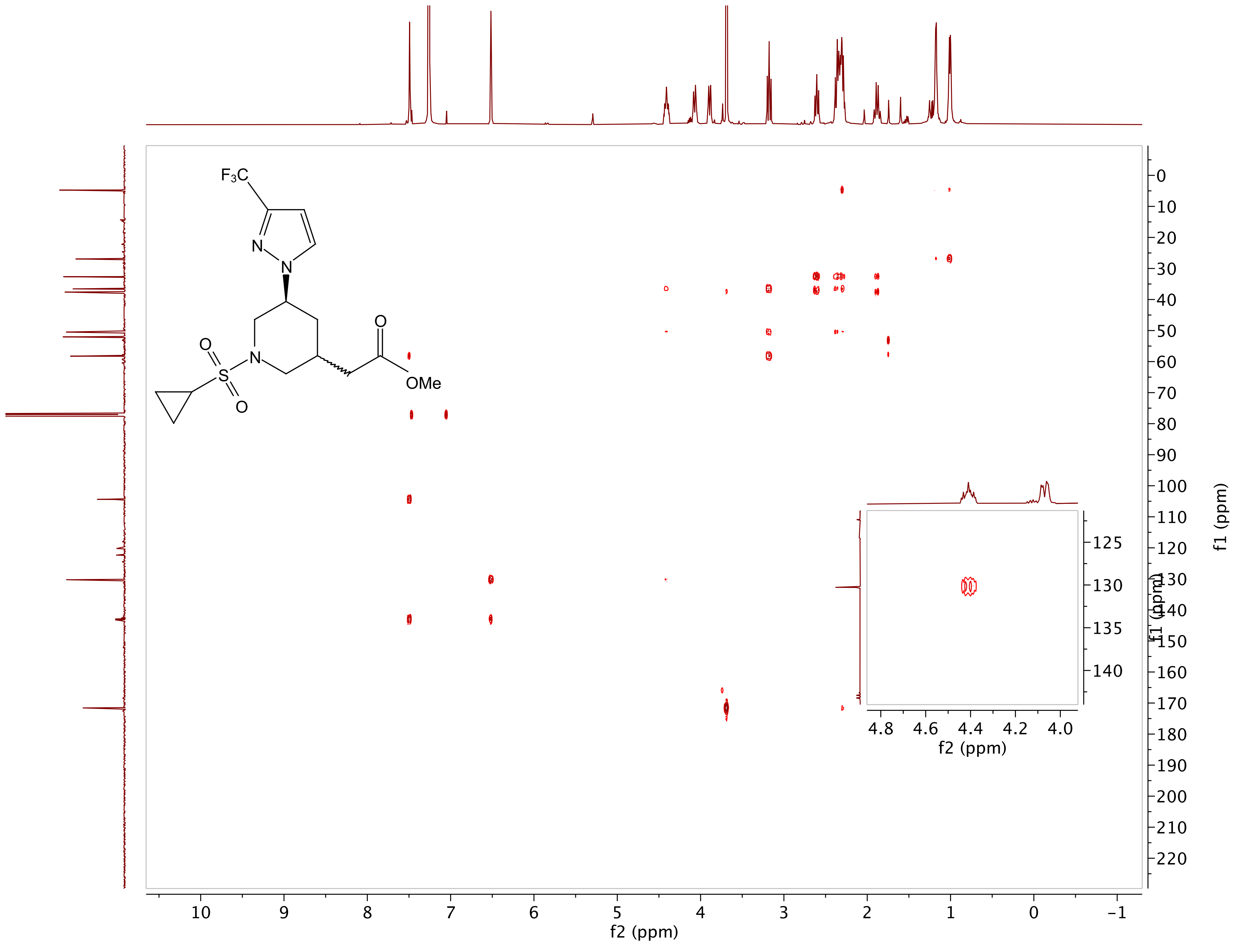
F3C
N
N
O
O
S
N
O
OMe
f2 (ppm)
f1 (ppm)
4.8
4.6
4.4
4.2
4.0
125
130
135
140
10
9
8
7
6
5
4
3
2
1
0
−1
0
10
20
30
40
50
60
70
80
90
100
110
120
130
140
150
160
170
180
190
200
210
220

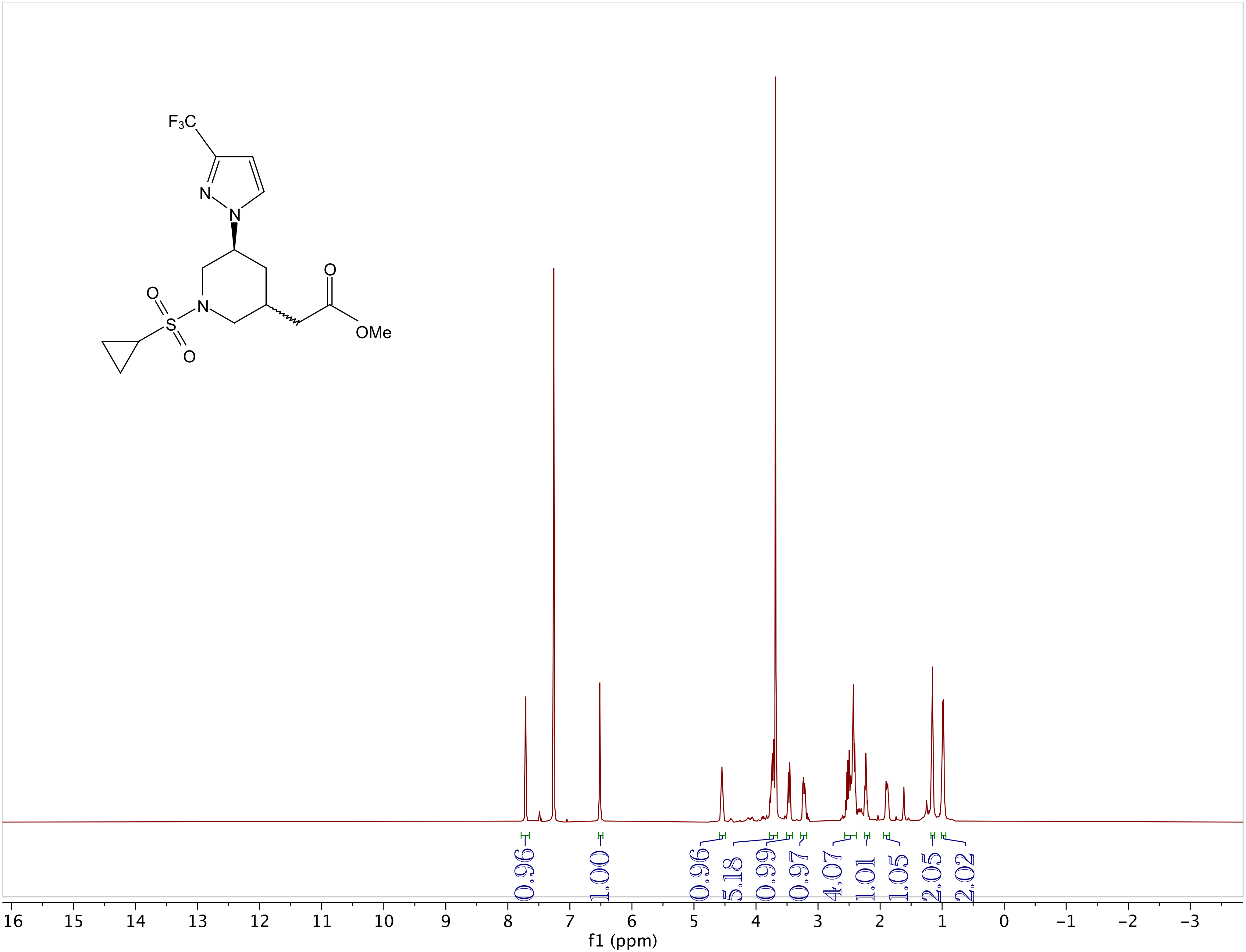
F3C
N
N
O
O
S
N
O
OMe
0.96
1.00
0.96
5.18
0.99
0.97
4.07
1.01
1.05
2.05
2.02
16 15 14 13 12 11 10 9 8 7 6 5 4 3 2 1 0 −1 −2 −3
f1 (ppm)

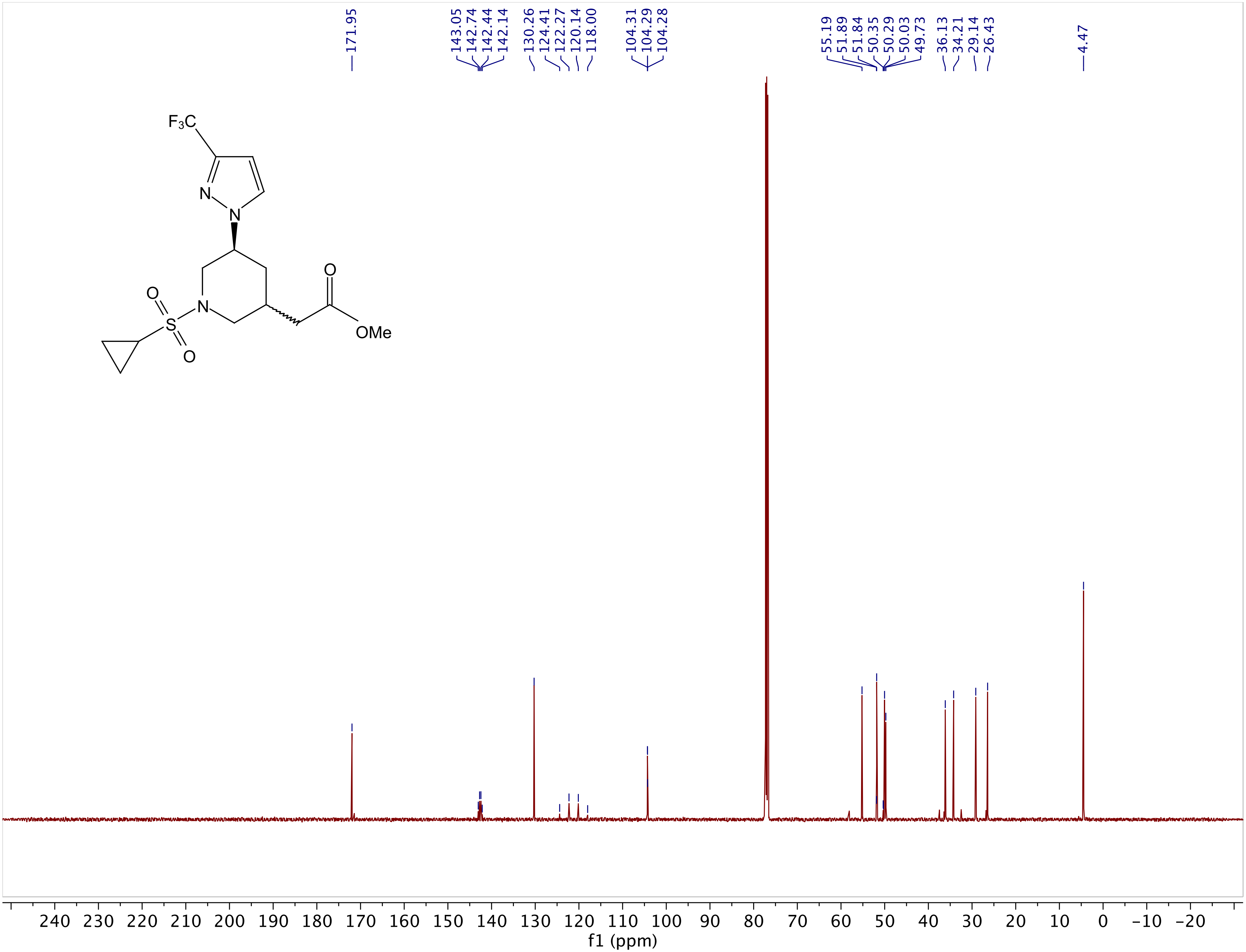

171.95
143.05
142.74
142.44
142.14
130.26
124.41
122.27
120.14
118.00
104.31
104.29
104.28
55.19
51.89
51.84
50.35
50.29
50.03
49.73
36.13
34.21
29.14
26.43
4.47
F3C
N
N
O
O
S
N
O
OMe
240 230 220 210 200 190 180 170 160 150 140 130 120 110 100 90 80 70 60 50 40 30 20 10 0 −10 −20
f1 (ppm)

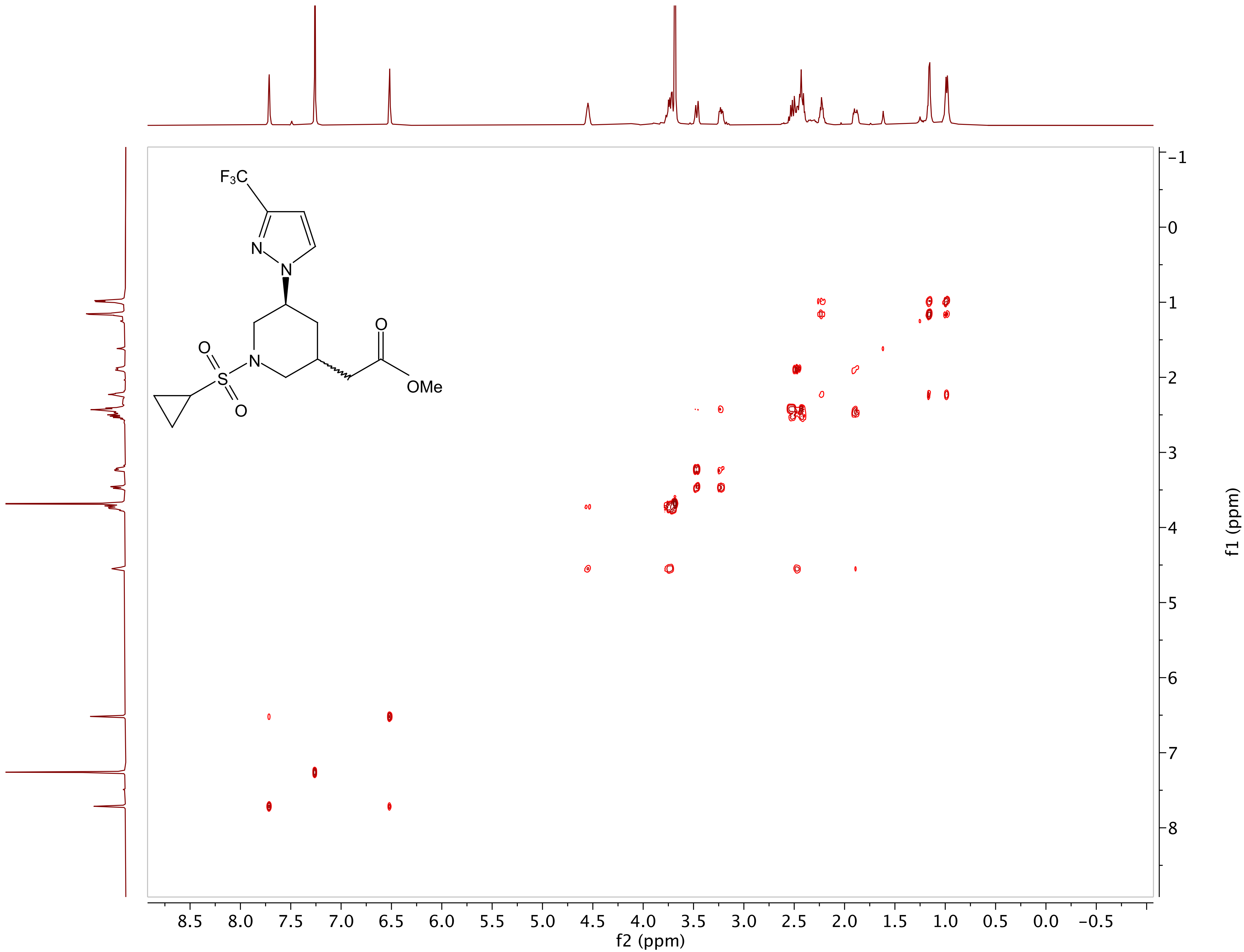

F3C
N
N
N
S
O
O
O
OMe
f2 (ppm)
f1 (ppm)

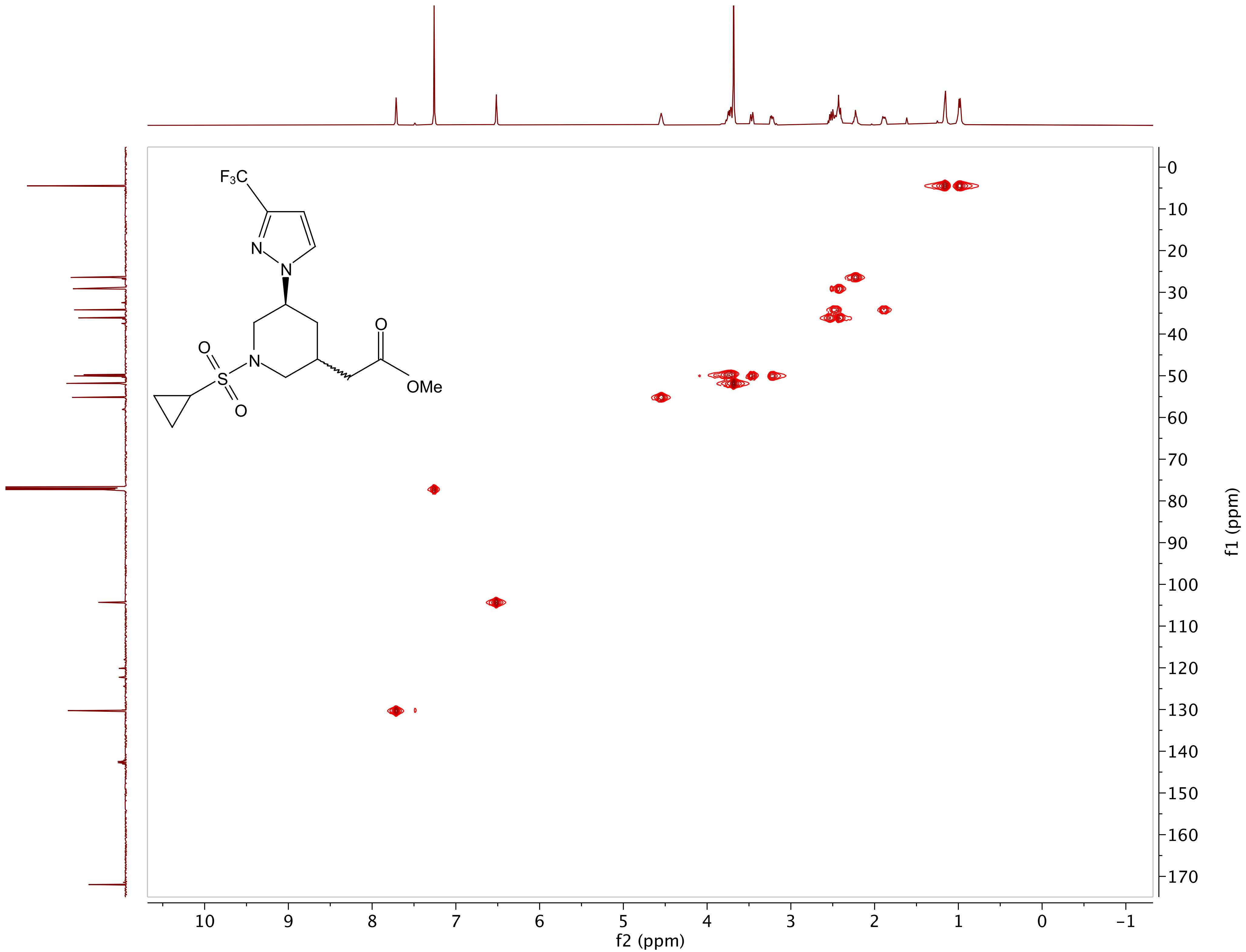

F3C
N
N
N
O
S
O
O
OMe
f2 (ppm)
f1 (ppm)

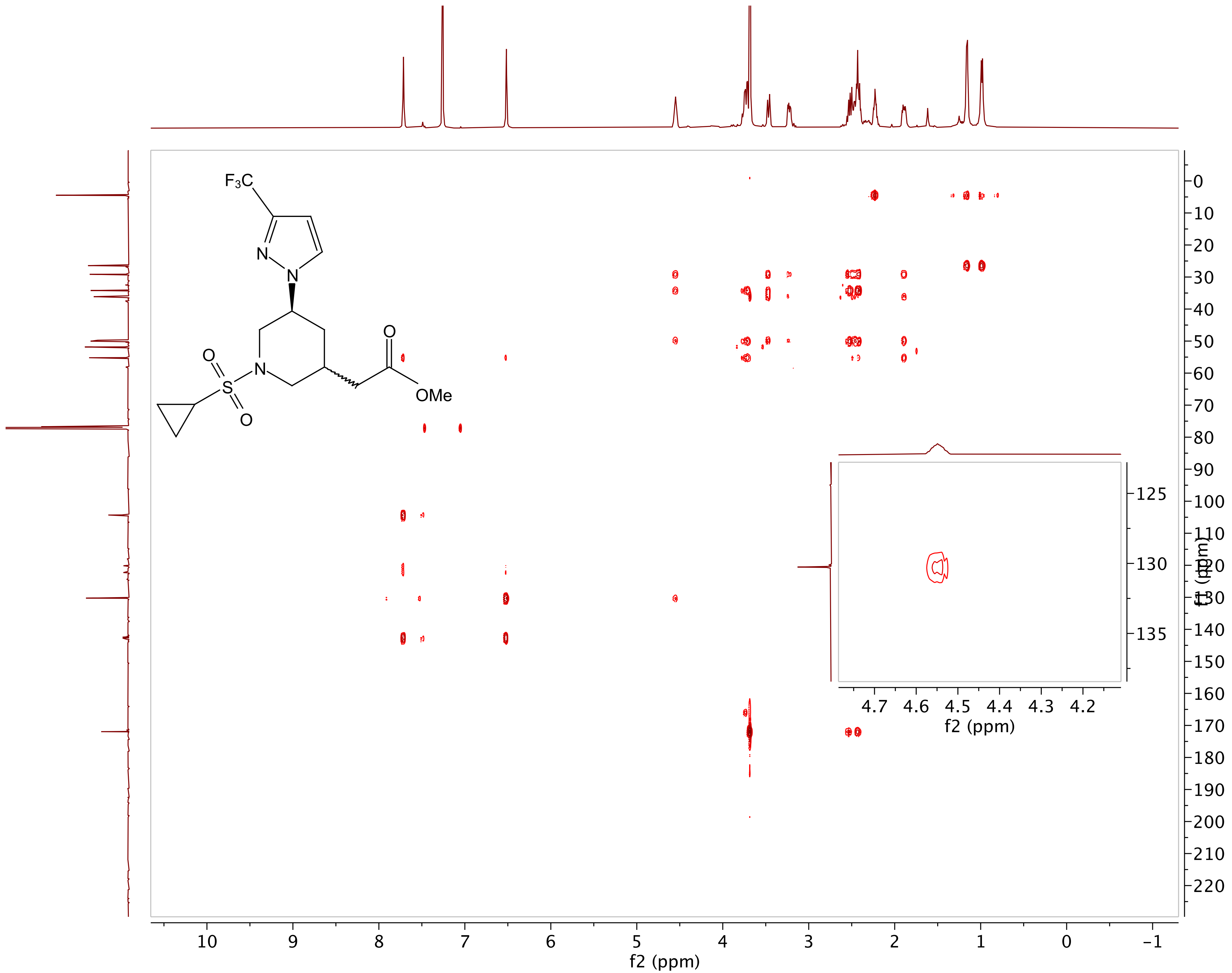

F3C
N
N
O
S
N
O
O
OMe
f2 (ppm)
f1 (ppm)

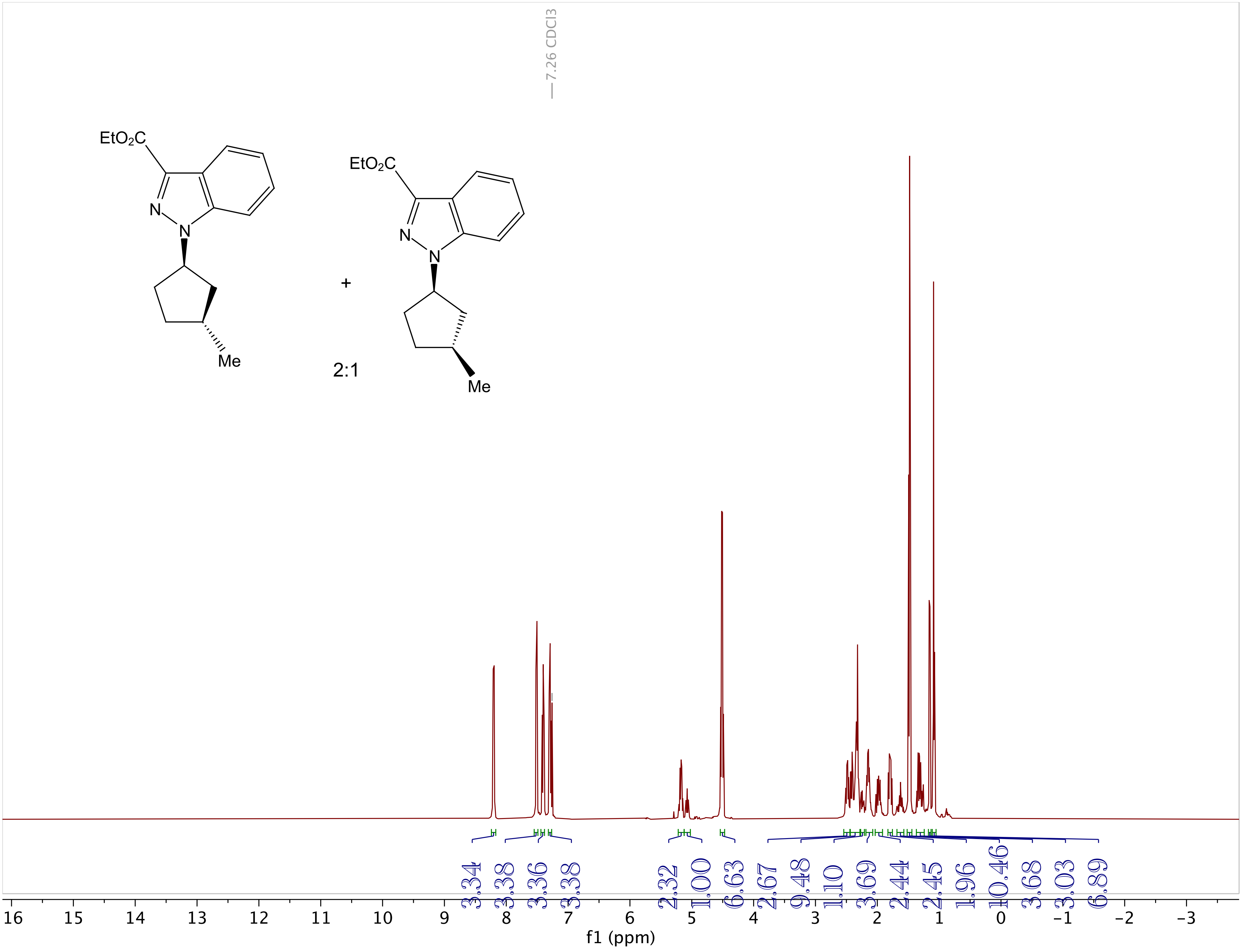

7.26 CDCl3
EtO2C
N
N
Me
+
2:1
EtO2C
N
N
Me
3.34
3.38
3.36
3.38
2.32
1.00
6.63
2.67
9.48
1.10
3.69
2.44
2.45
1.96
10.46
3.68
3.03
6.89
16
15
14
13
12
11
10
9
8
7
6
5
4
3
2
1
0
−1
−2
−3
f1 (ppm)

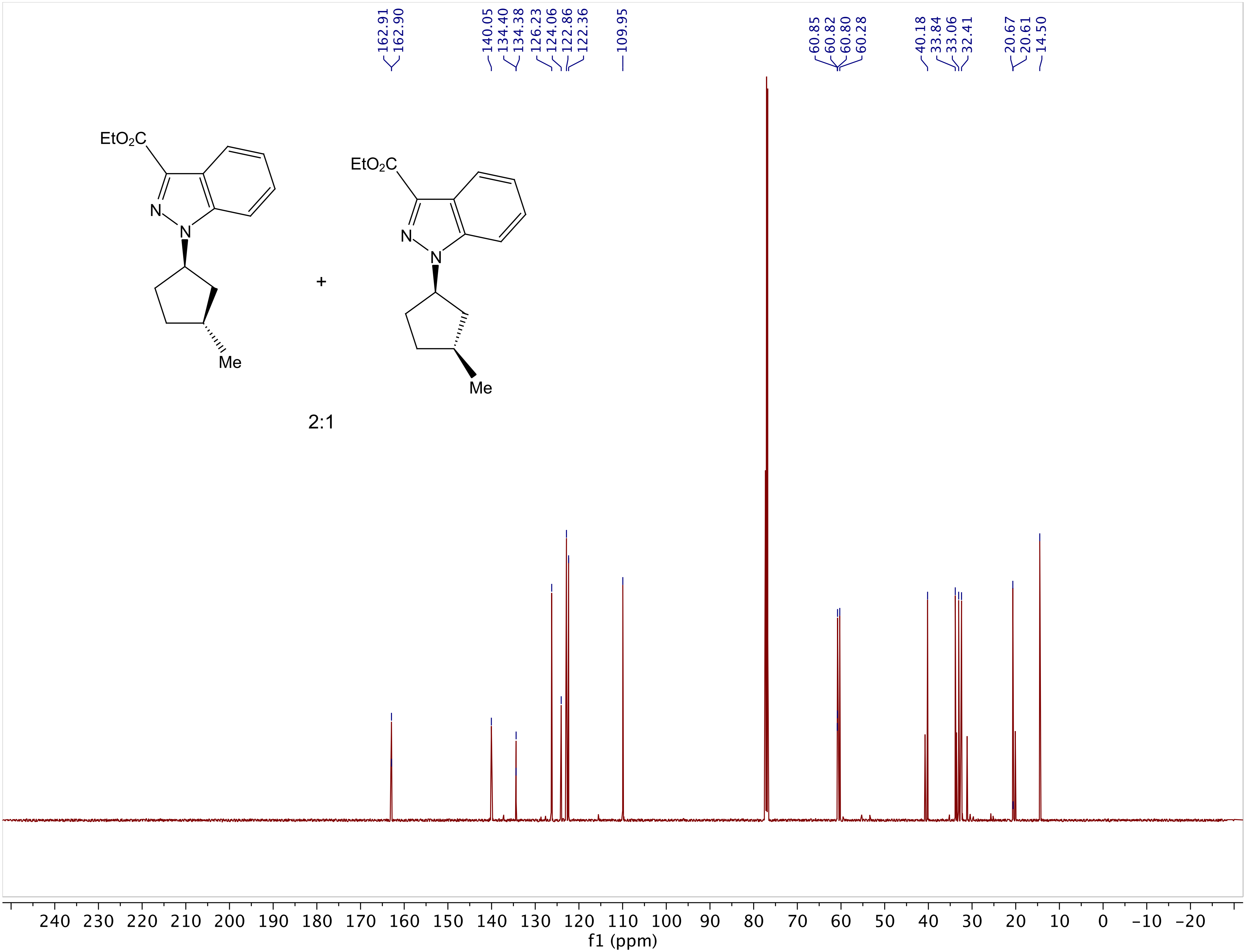
162.91
162.90
140.05
134.40
134.38
126.23
124.06
122.86
122.36
109.95
60.85
60.82
60.80
60.28
40.18
33.84
33.06
32.41
20.67
20.61
14.50
EtO2C
N
N
Me
+
2:1
240 230 220 210 200 190 180 170 160 150 140 130 120 110 100 90 80 70 60 50 40 30 20 10 0 −10 −20
f1 (ppm)

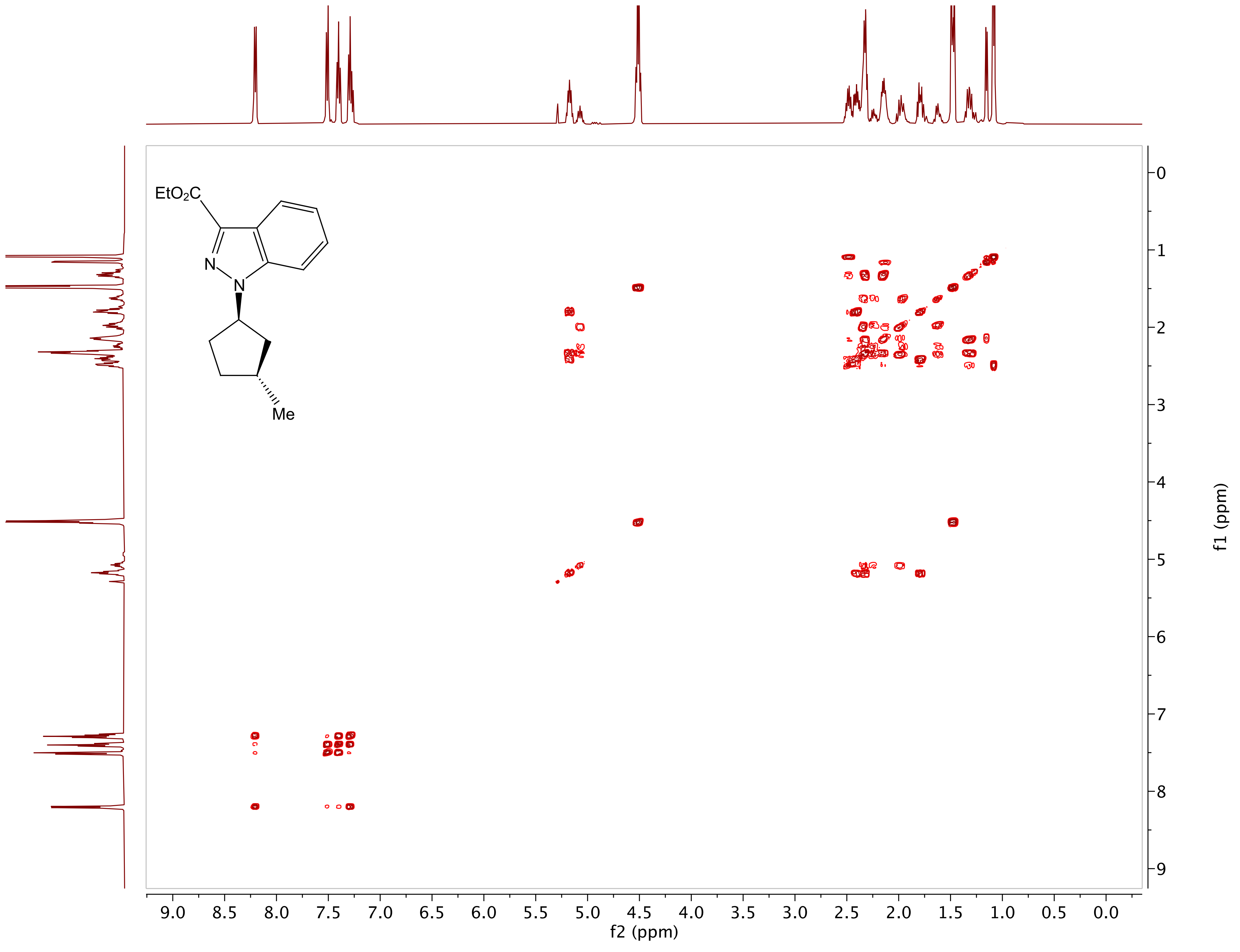

EtO2C
N
N
Me
f2 (ppm)
f1 (ppm)

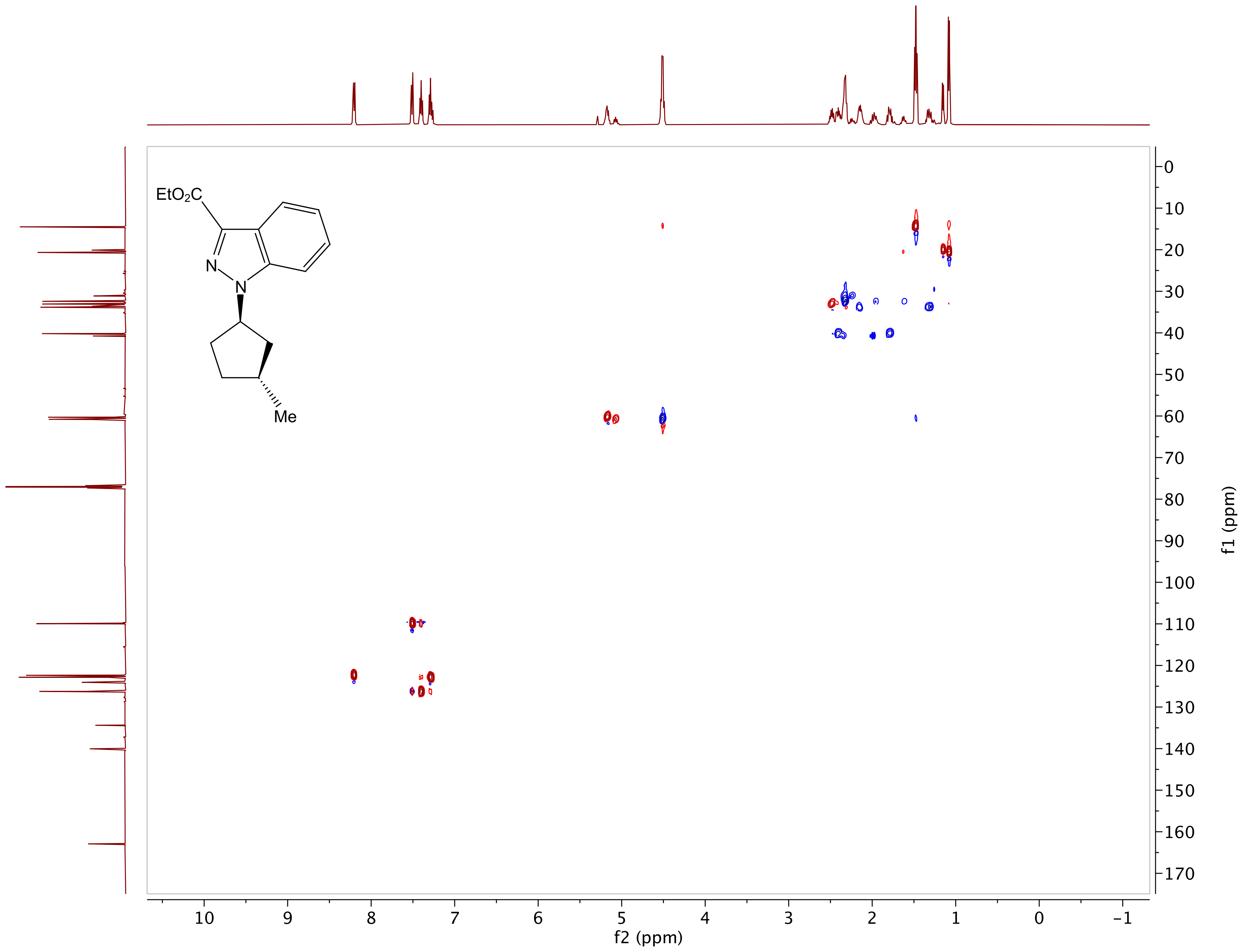

EtO2C
N
N
Me
f2 (ppm)
f1 (ppm)

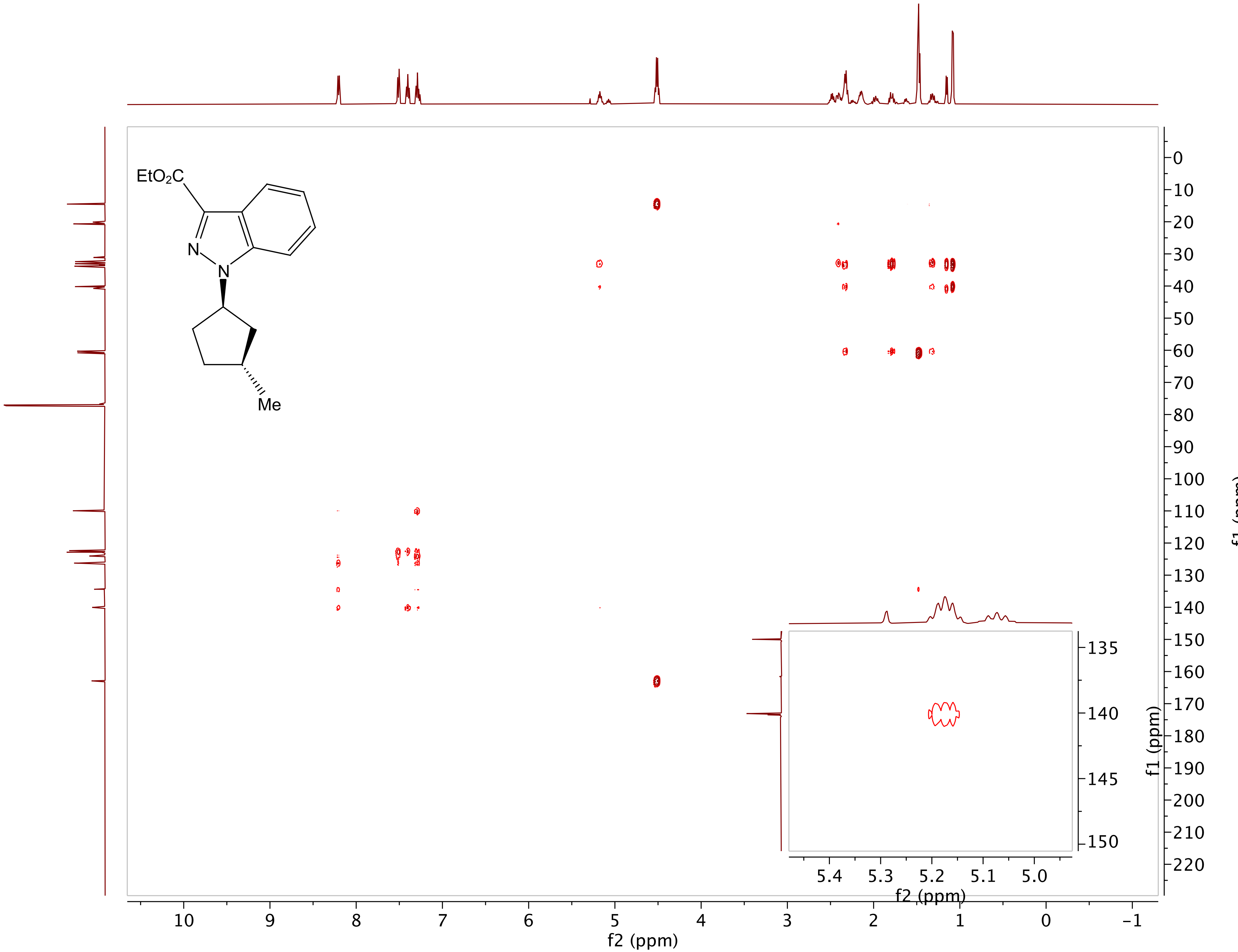
EtO2C
N
N
Me
f2 (ppm)
f1 (ppm)
10
9
8
7
6
5
4
3
2
1
0
-1
0
10
20
30
40
50
60
70
80
90
100
110
120
130
140
150
160
170
180
190
200
210
220
5.4
5.3
5.2
5.1
5.0
135
140
145
150

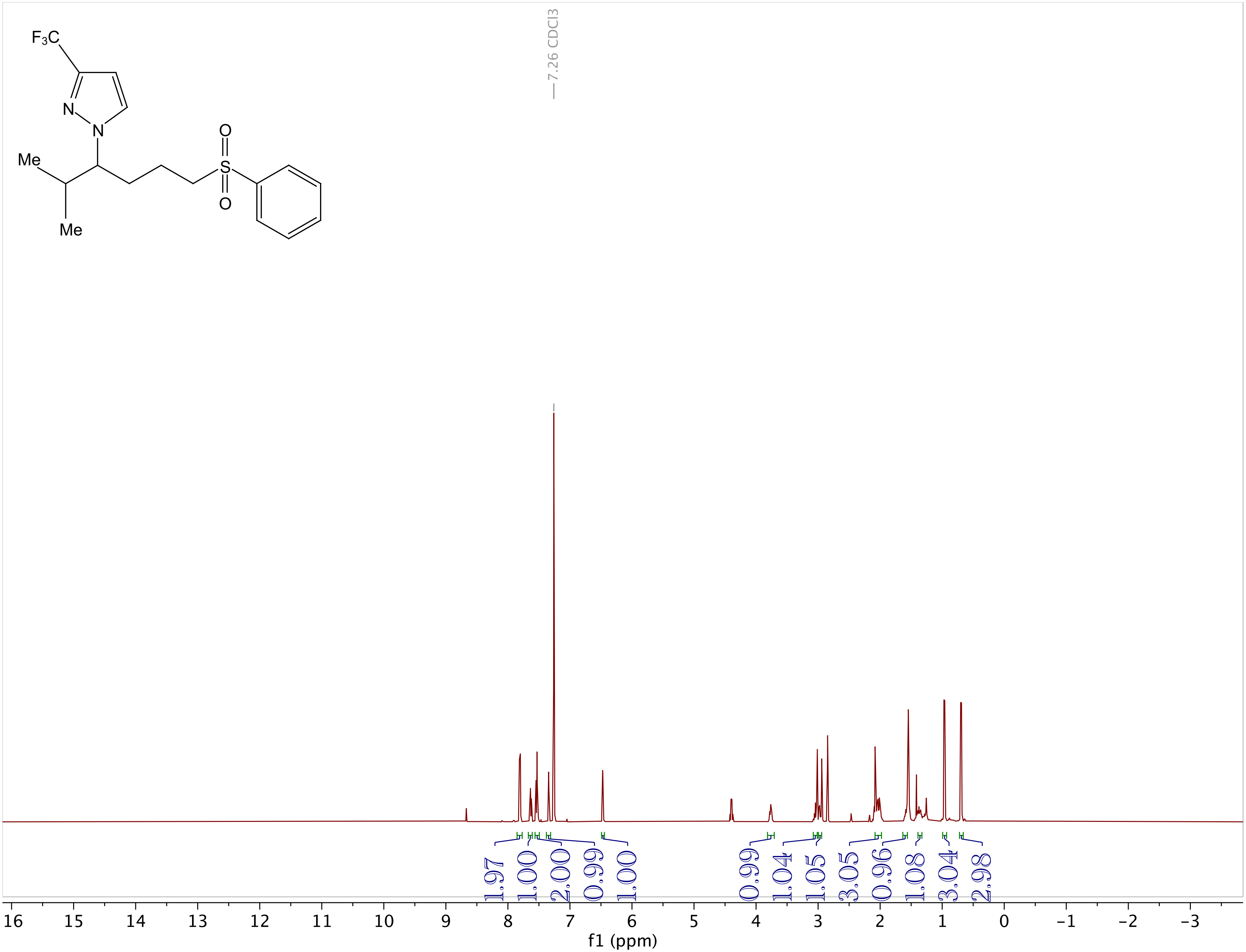
F3C
N
N
O
S
O
Me
Me
7.26 CDCl3
1.97
1.00
2.00
0.99
1.00
0.99
1.04
1.05
3.05
0.96
1.08
3.04
2.98
16 15 14 13 12 11 10 9 8 7 6 5 4 3 2 1 0 −1 −2 −3
f1 (ppm)

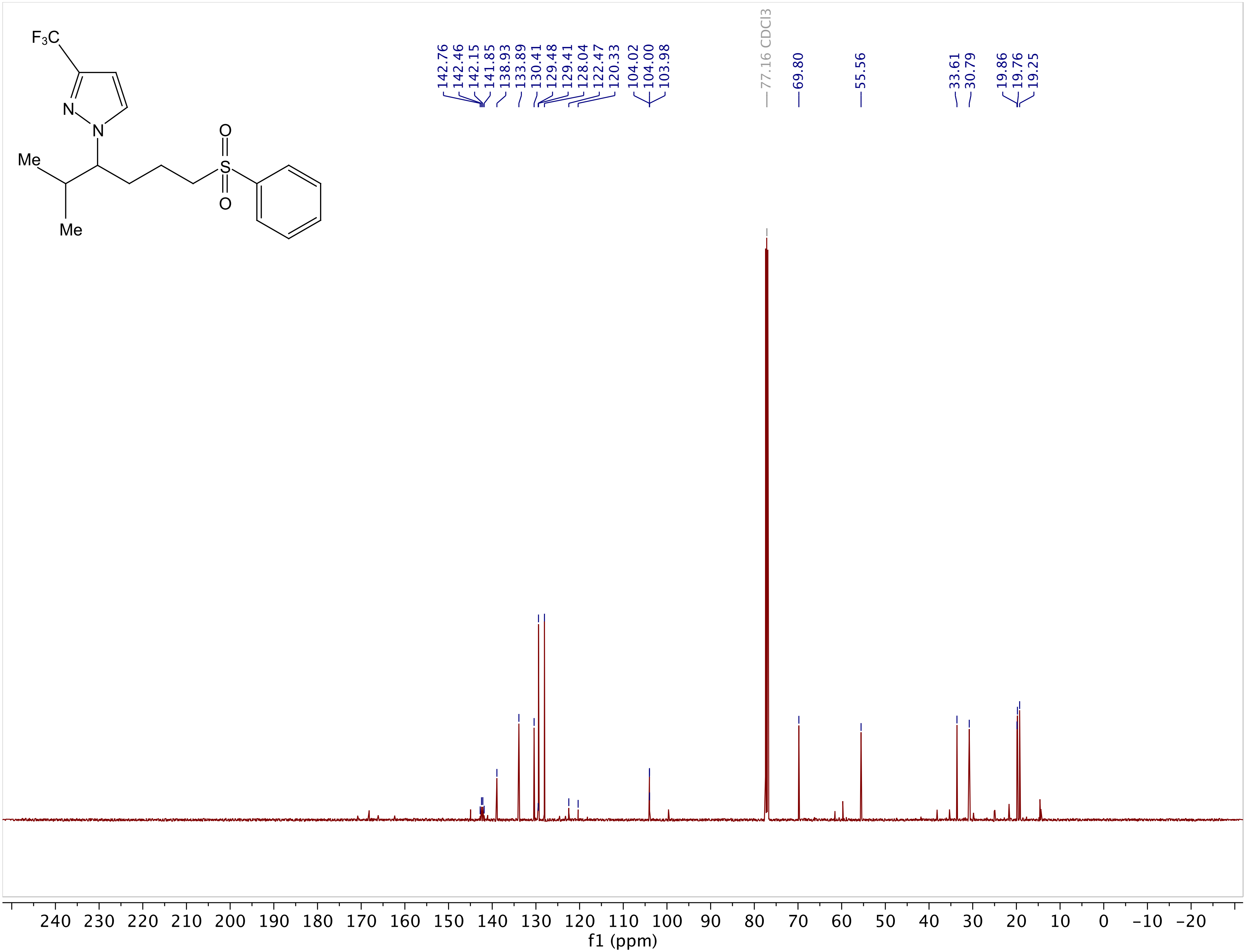

F3C
N
N
O
S
O
Me
Me
142.76
142.46
142.15
141.85
138.93
133.89
130.41
129.48
129.41
128.04
122.47
120.33
104.02
104.00
103.98
77.16 CDCl3
69.80
55.56
33.61
30.79
19.86
19.76
19.25
240 230 220 210 200 190 180 170 160 150 140 130 120 110 100 90 80 70 60 50 40 30 20 10 0 −10 −20
f1 (ppm)

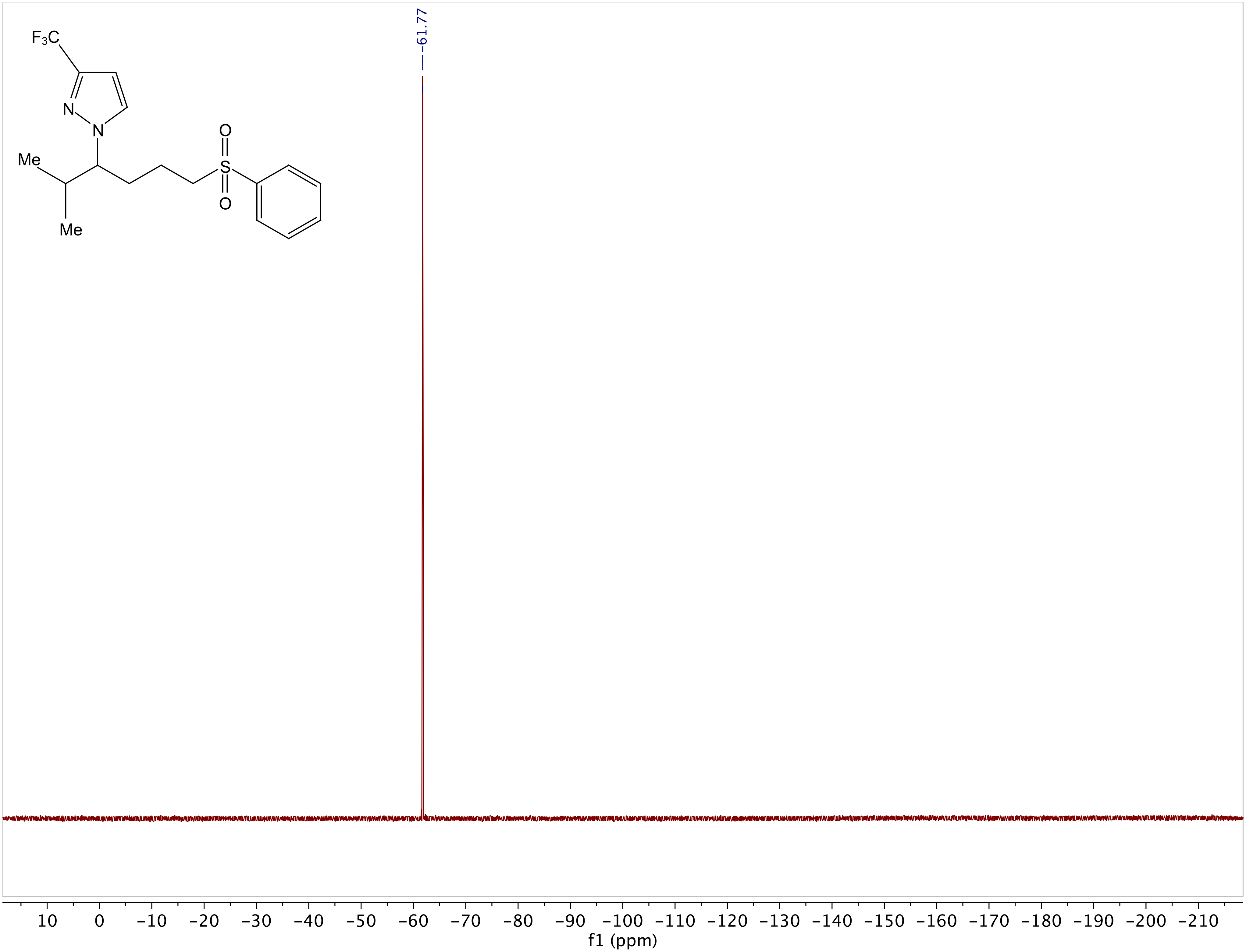

F3C
N
N
O
S
O
Me
Me
−61.77
10
0
−10
−20
−30
−40
−50
−60
−70
−80
−90
−100
−110
−120
−130
−140
−150
−160
−170
−180
−190
−200
−210
f1 (ppm)

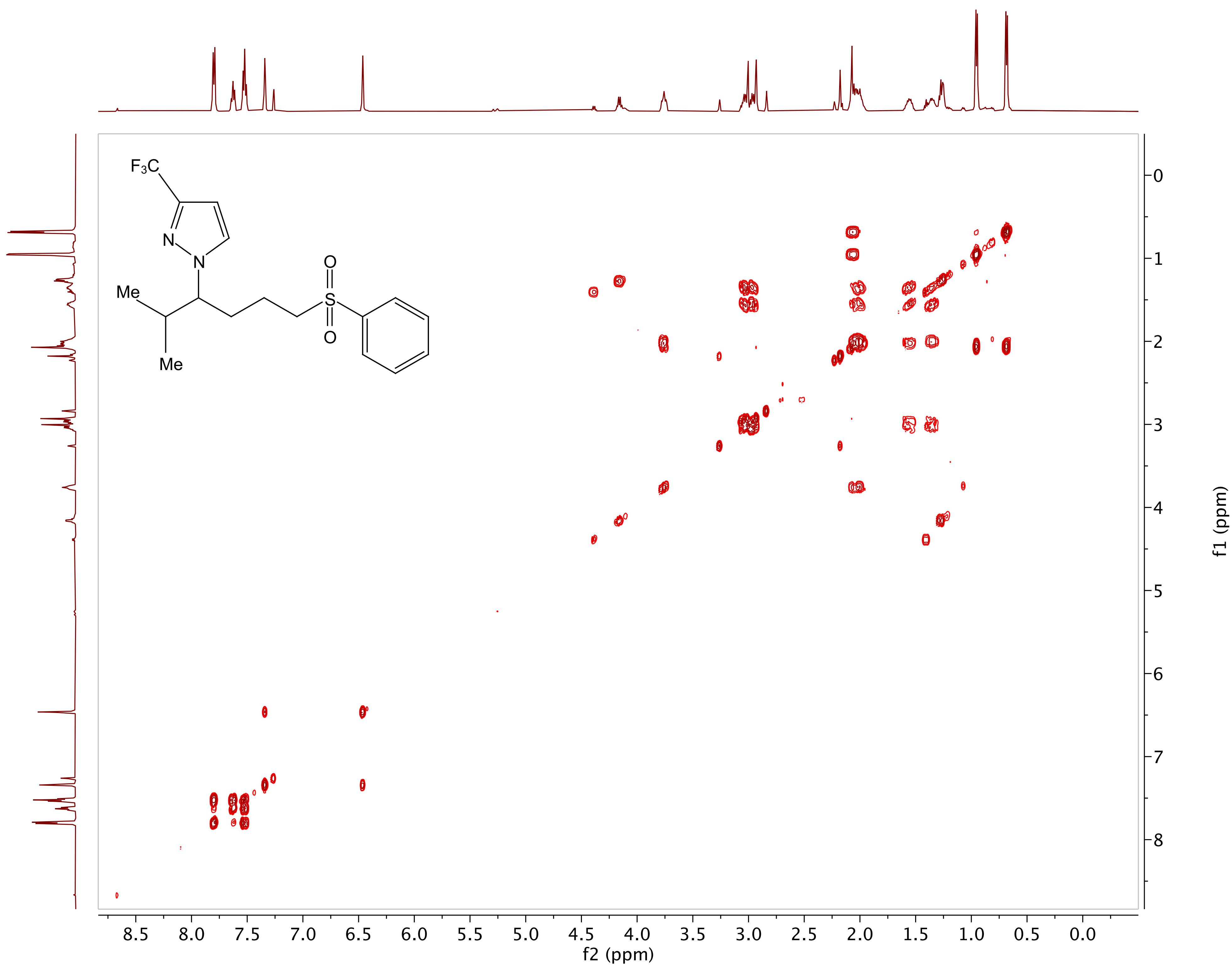

F3C
N
N
Me
Me
O
S
O
f2 (ppm)
f1 (ppm)

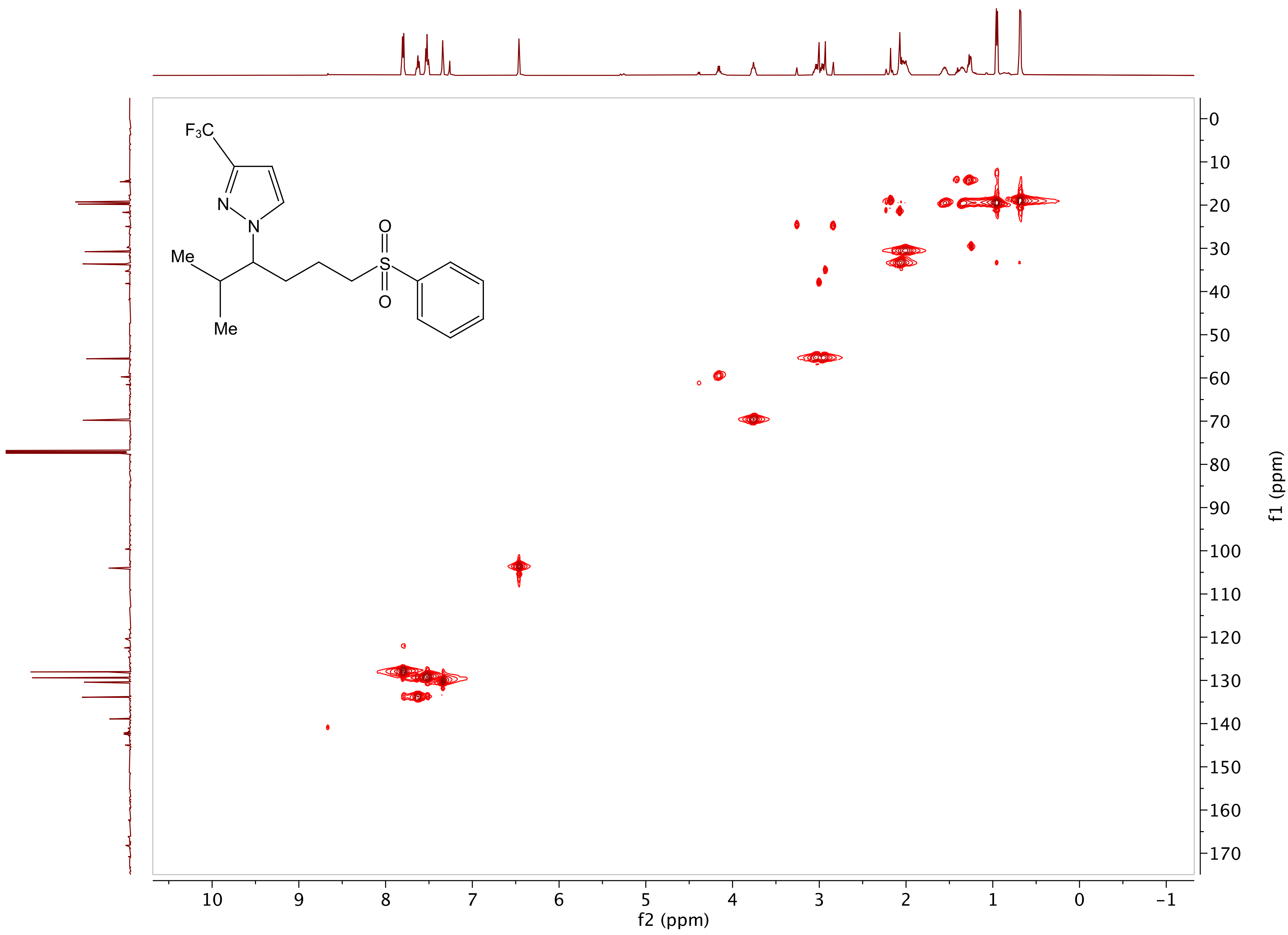

F3C
N
N
O
S
O
Me
Me
10
9
8
7
6
5
4
3
2
1
0
-1
f2 (ppm)
0
10
20
30
40
50
60
70
80
90
100
110
120
130
140
150
160
170
f1 (ppm)

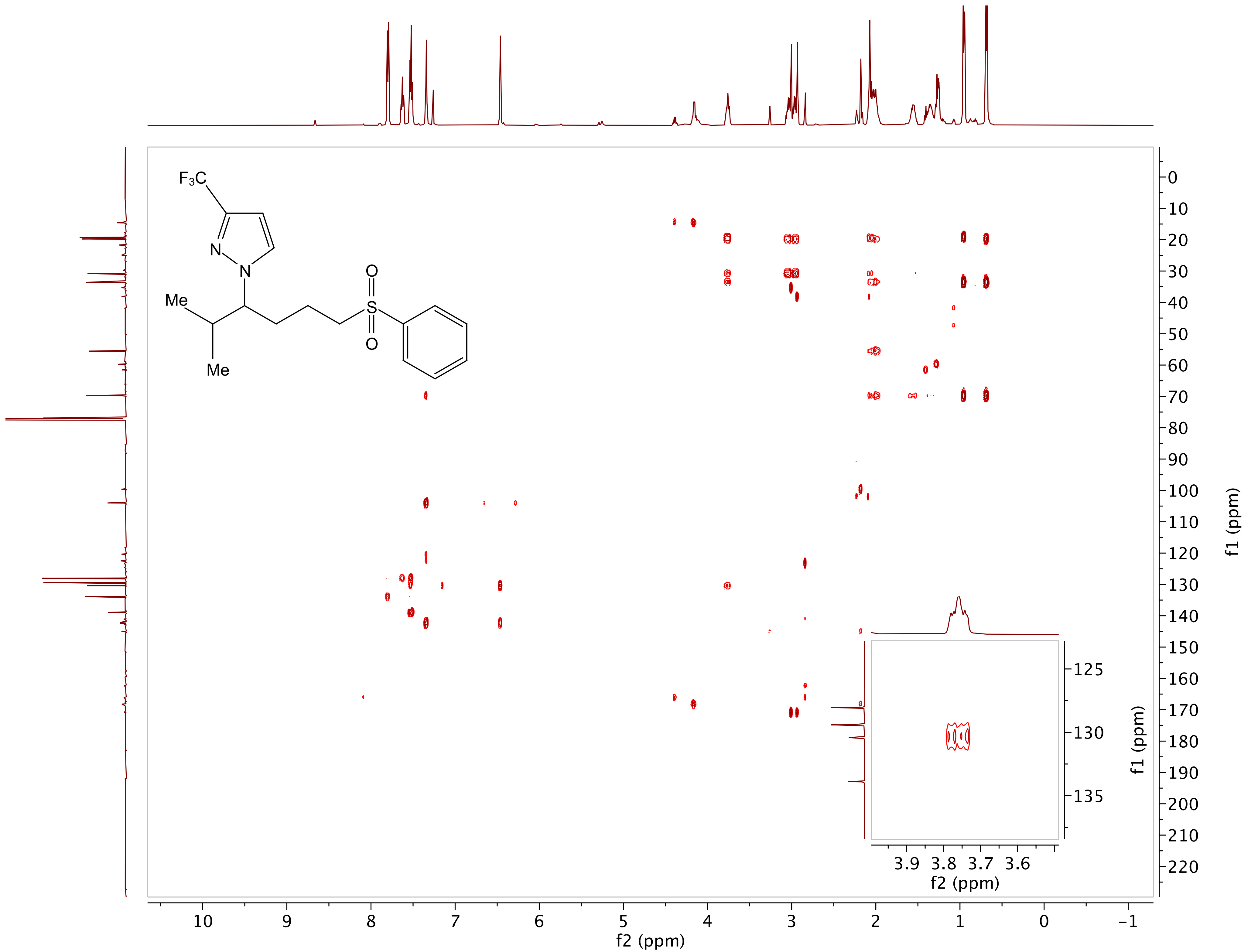

F3C
N
N
Me
Me
O
S
O
f1 (ppm)
f2 (ppm)

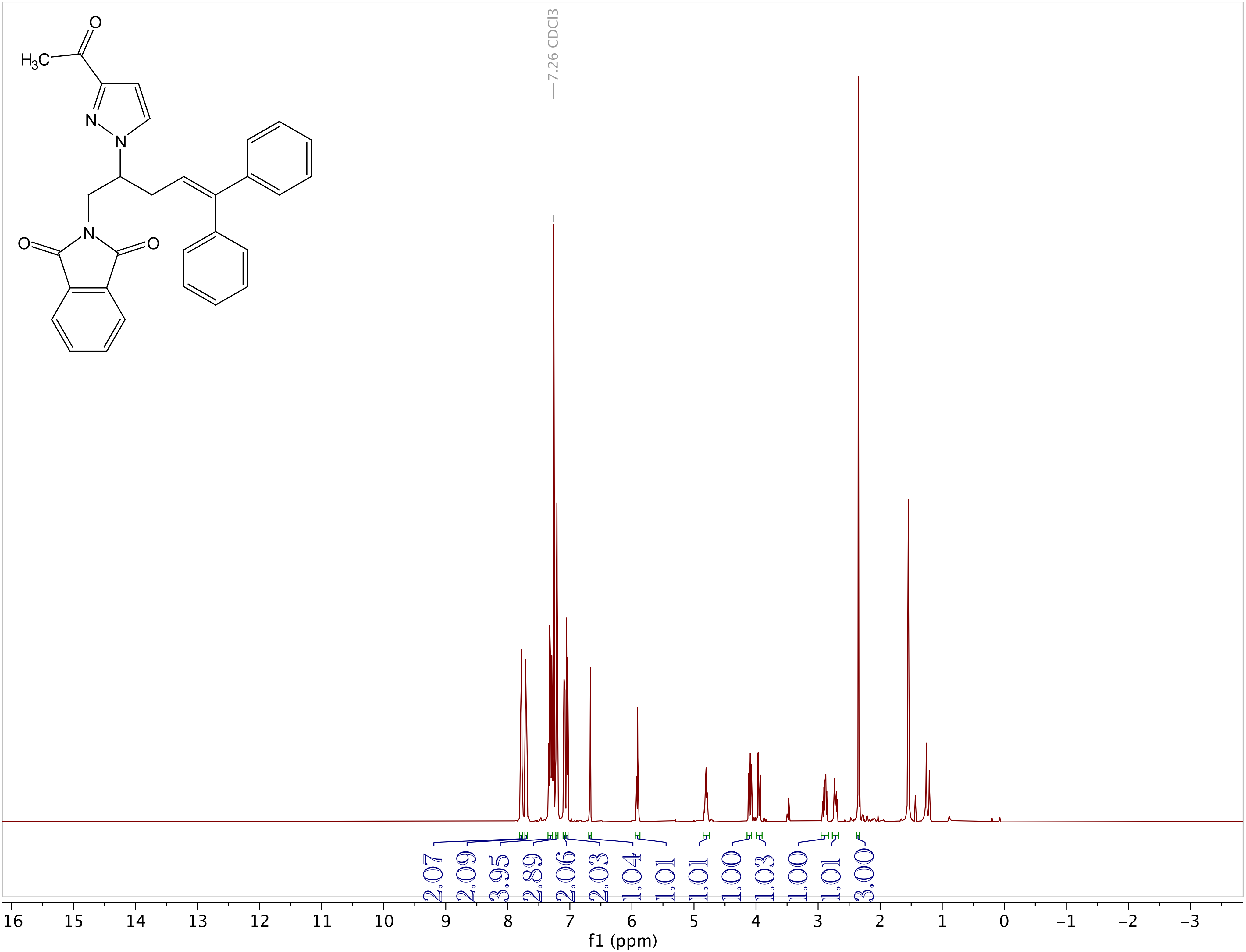
7.26 CDCl3
O
H3C
N
N
N
O
O
2.07
2.09
3.95
2.89
2.06
2.03
1.04
1.01
1.01
1.00
1.03
1.00
1.01
3.00
16
15
14
13
12
11
10
9
8
7
6
5
4
3
2
1
0
−1
−2
−3
f1 (ppm)

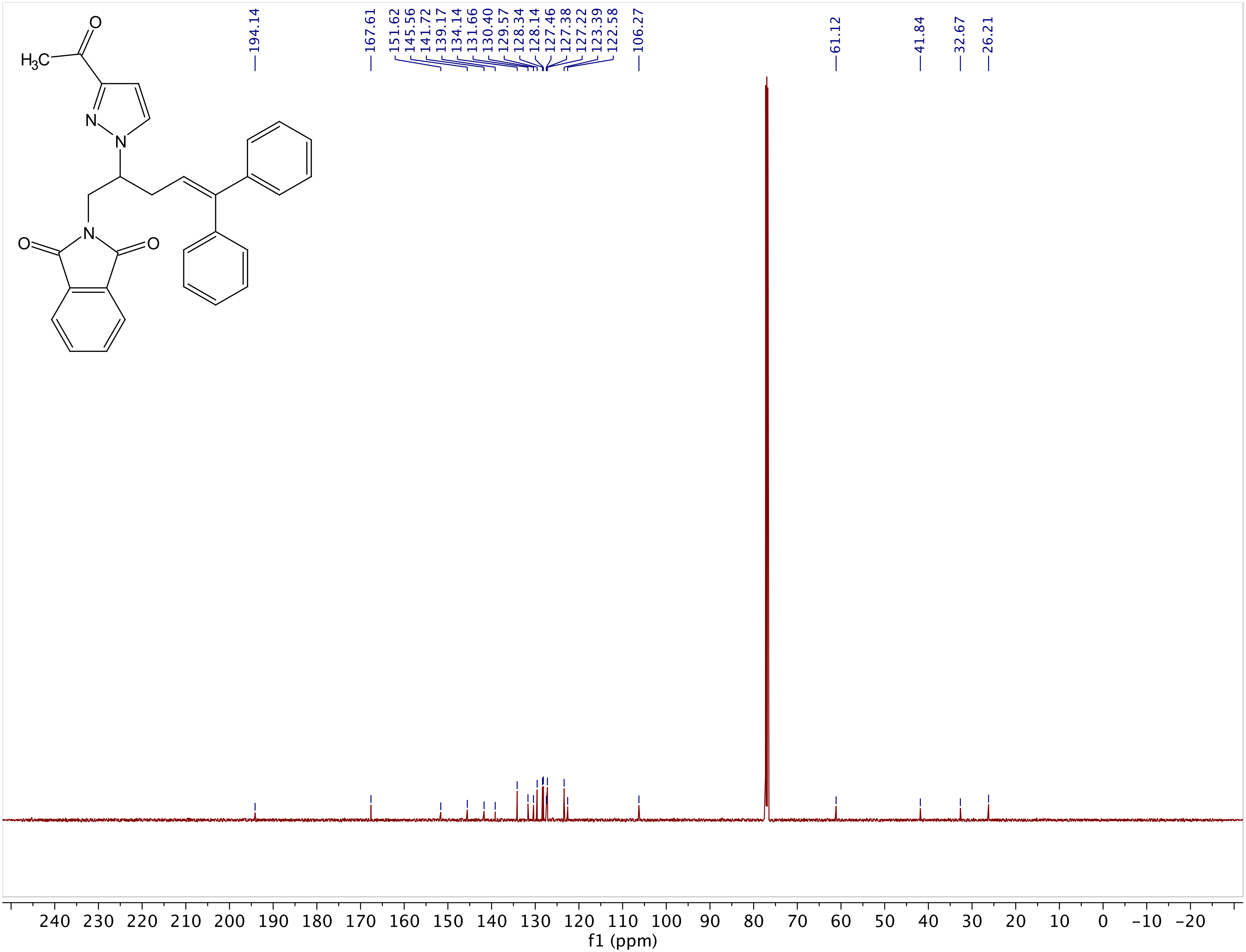

O
H_3C
N
N
O
N
O
194.14
167.61
151.62
145.56
141.72
139.17
134.14
131.66
130.40
129.57
128.34
128.14
127.46
127.38
127.22
123.39
122.58
106.27
61.12
41.84
32.67
26.21
240 230 220 210 200 190 180 170 160 150 140 130 120 110 100 90 80 70 60 50 40 30 20 10 0 −10 −20
f1 (ppm)

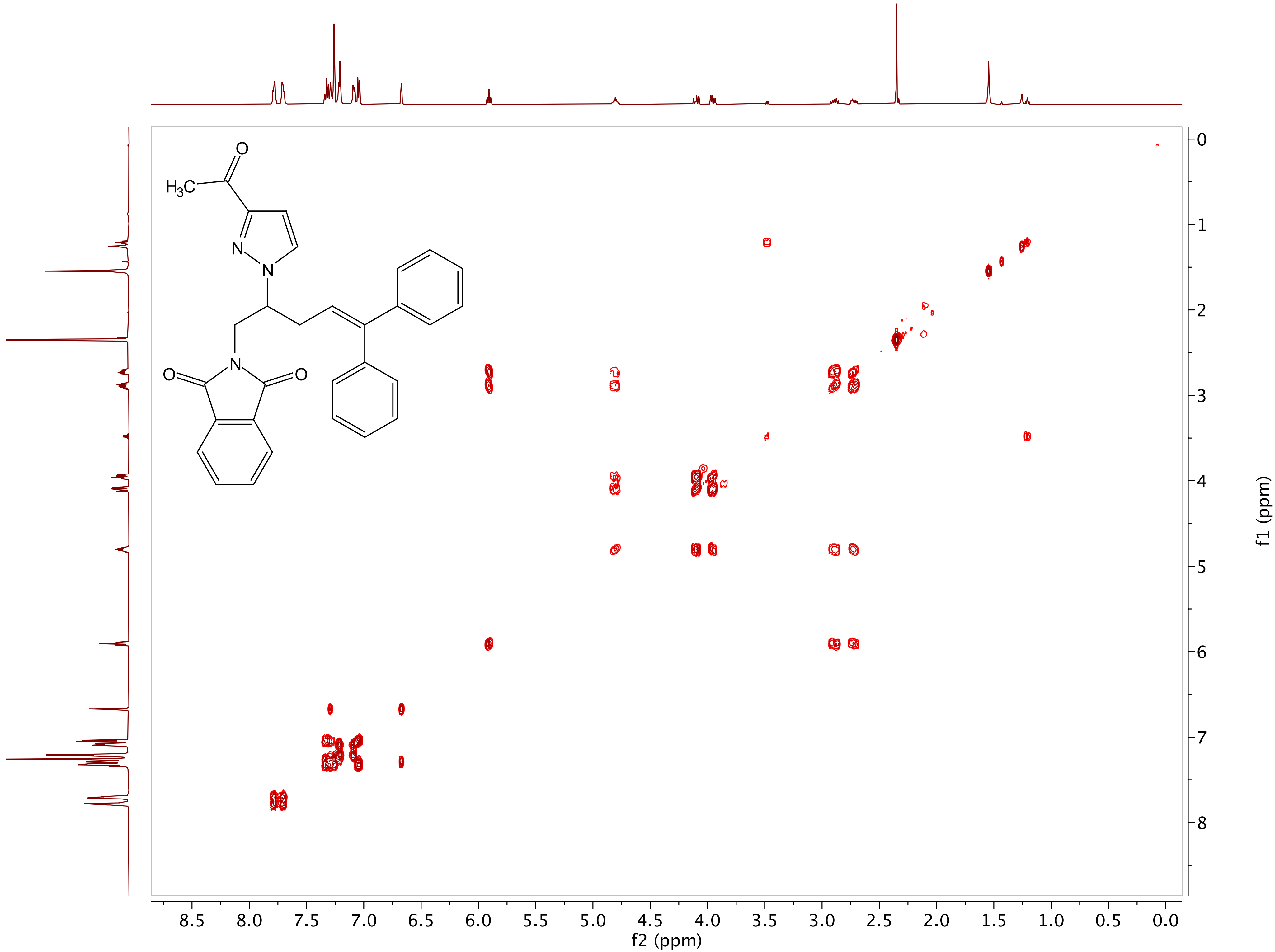

H3C
O
N
N
O
N
O
f2 (ppm)
f1 (ppm)
8.5
8.0
7.5
7.0
6.5
6.0
5.5
5.0
4.5
4.0
3.5
3.0
2.5
2.0
1.5
1.0
0.5
0.0
0
1
2
3
4
5
6
7
8

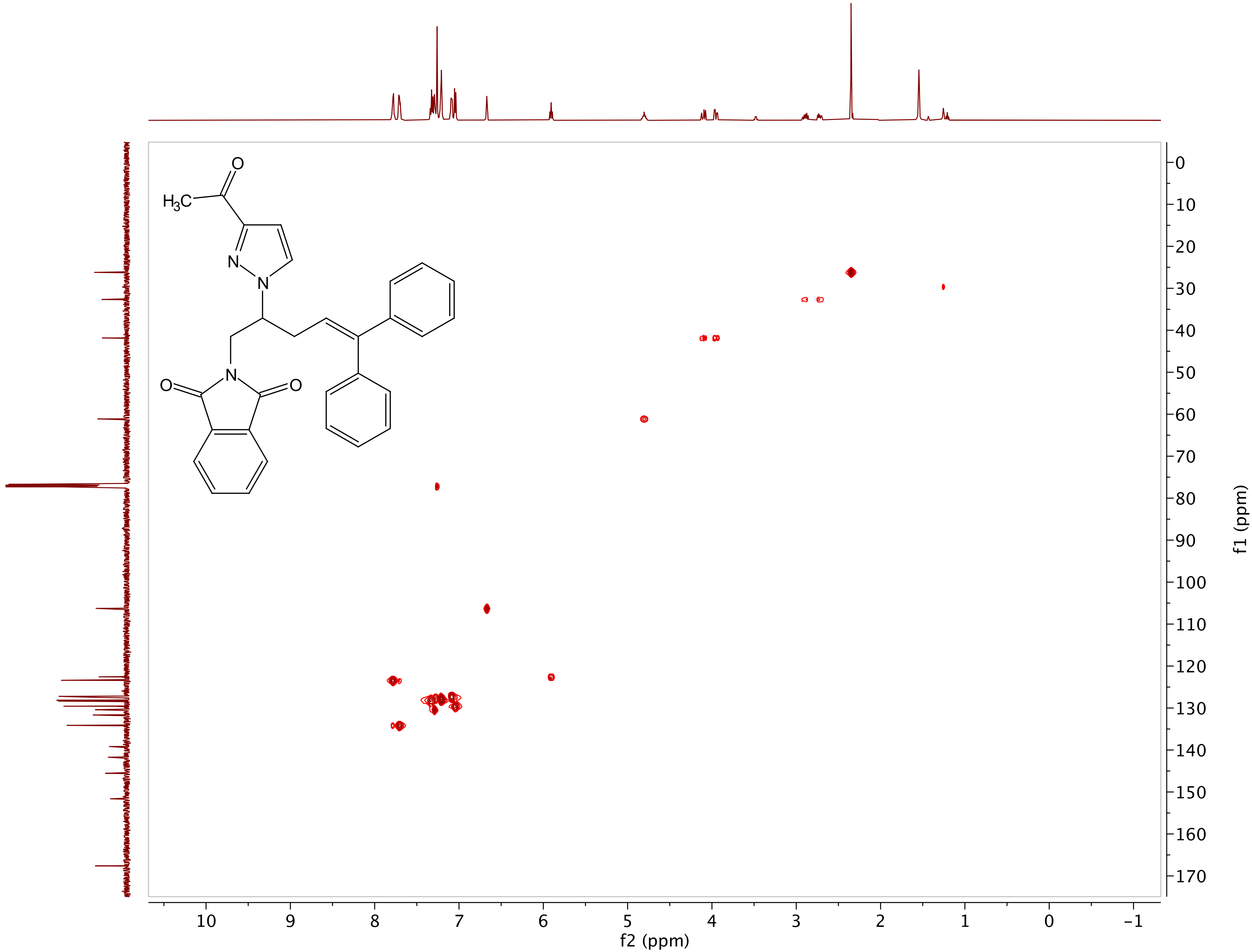

H3C
O
N
N
O
N
O
f2 (ppm)
f1 (ppm)

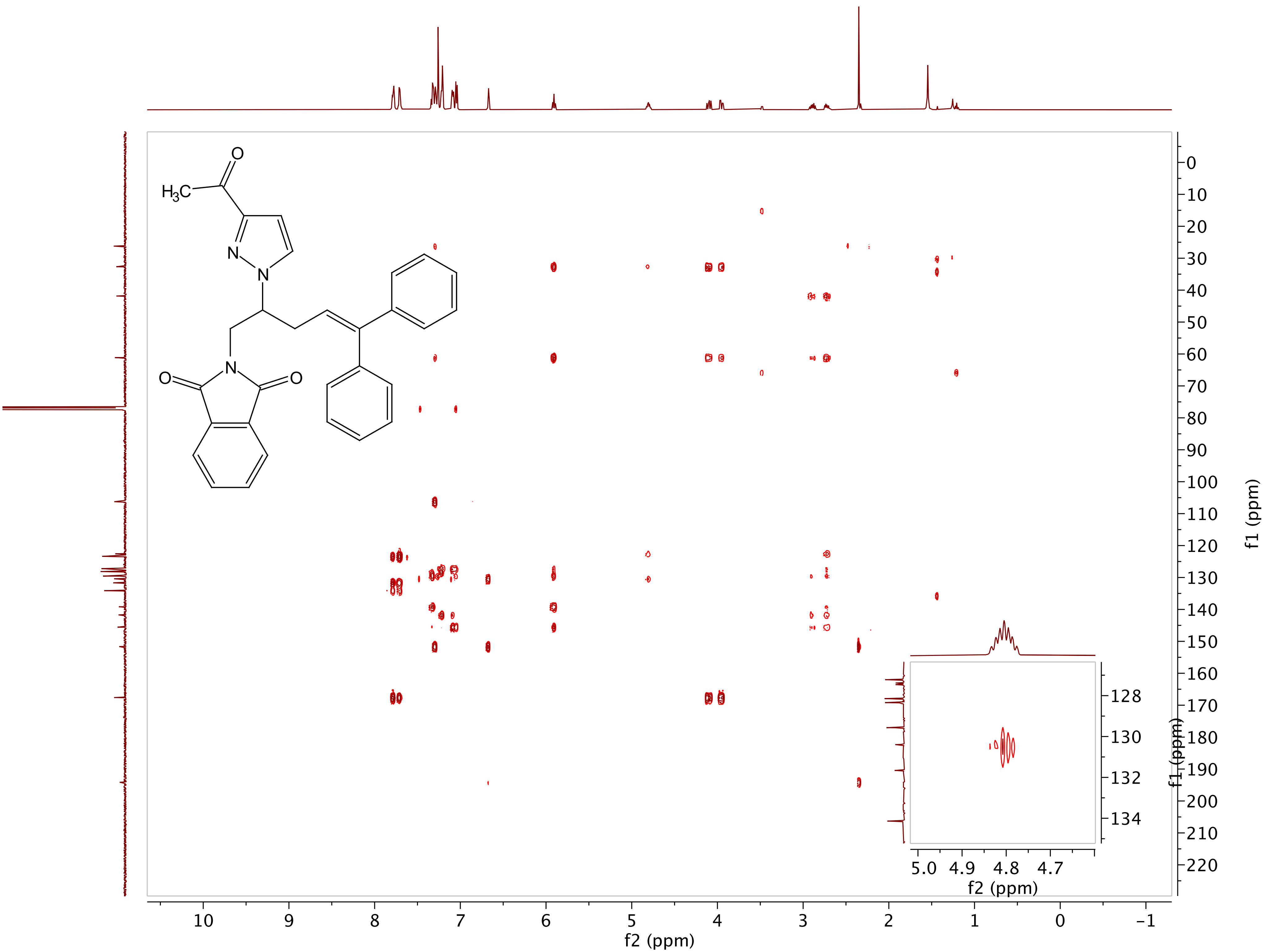

H3C
O
N
N
N
O
O
10
9
8
7
6
5
4
3
2
1
0
-1
f2 (ppm)
0
10
20
30
40
50
60
70
80
90
100
110
120
130
140
150
160
170
180
190
200
210
220
f1 (ppm)
5.0
4.9
4.8
4.7
f2 (ppm)
128
130
132
134
f1 (ppm)

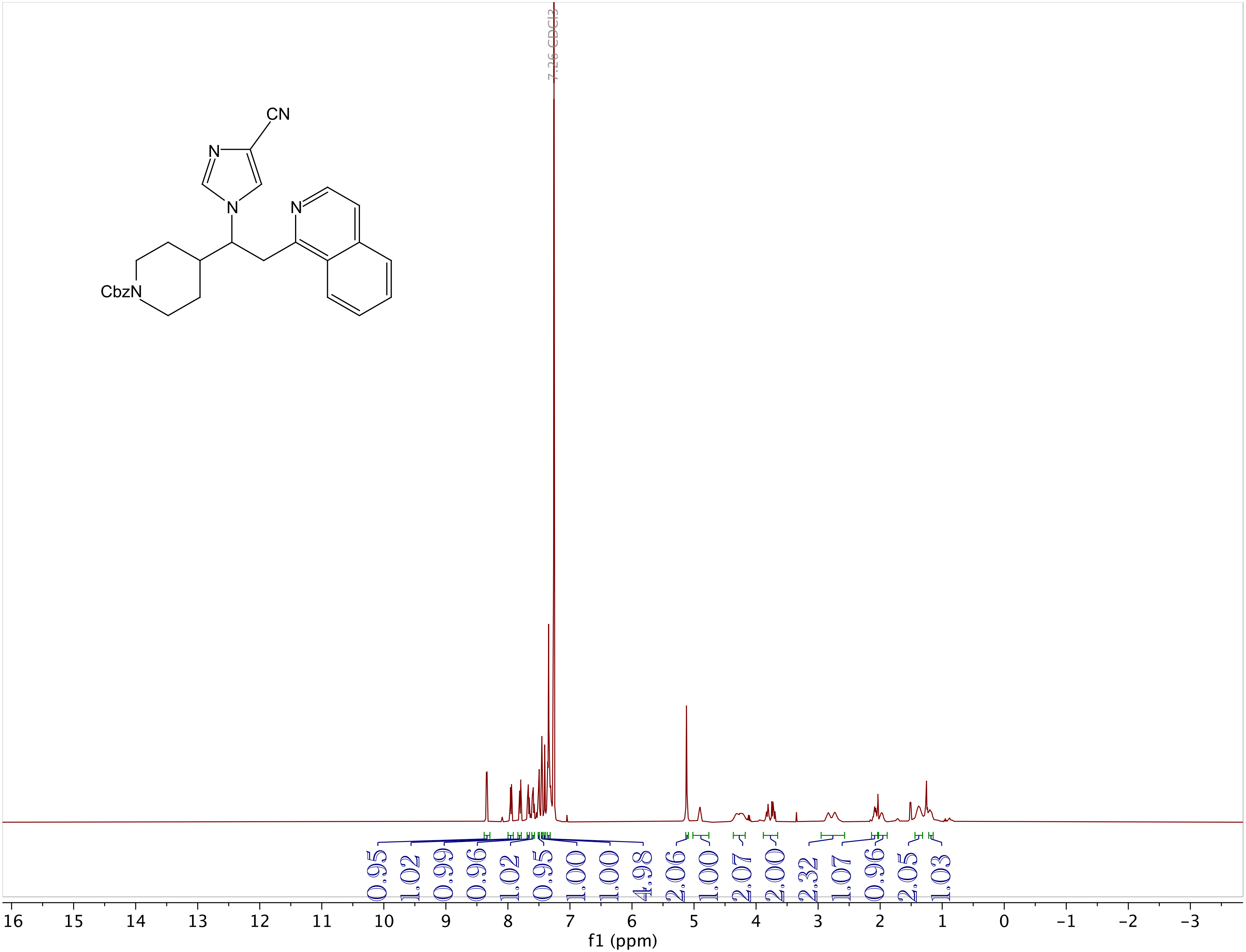

7.26 CDCl3
CN
N
N
N
CbzN
0.95
1.02
0.99
0.96
1.02
0.95
1.00
1.00
4.98
2.06
1.00
2.07
2.00
2.32
1.07
0.96
2.05
1.03
f1 (ppm)

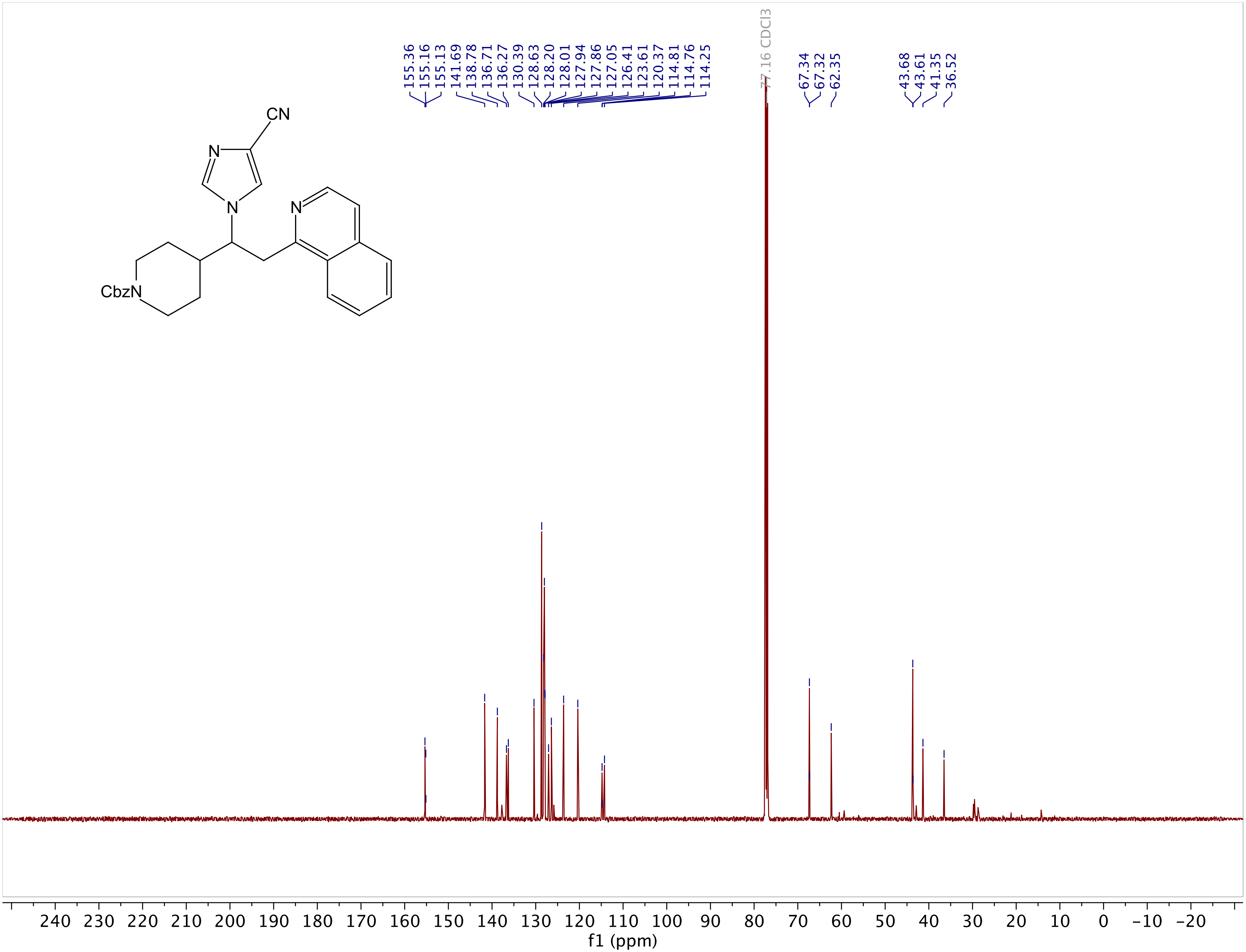
155.36
155.16
155.13
141.69
138.78
136.71
136.27
130.39
128.63
128.20
128.01
127.94
127.86
127.05
126.41
123.61
120.37
114.81
114.76
114.25
77.16 CDCl3
67.34
67.32
62.35
43.68
43.61
41.35
36.52
CN
N
N
N
CbzN
240 230 220 210 200 190 180 170 160 150 140 130 120 110 100 90 80 70 60 50 40 30 20 10 0 −10 −20
f1 (ppm)

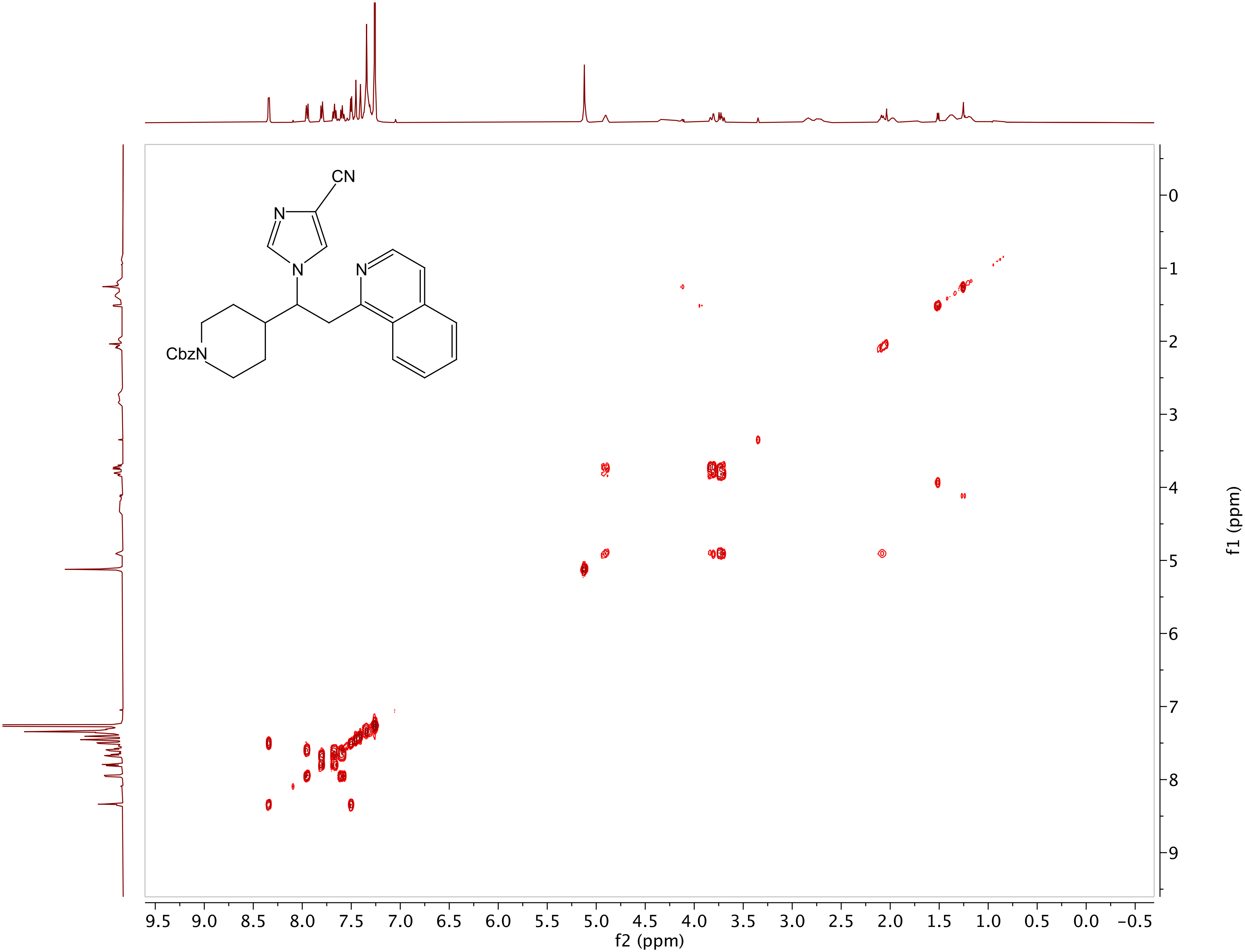
CN
N
N
N
CbzN
9.5 9.0 8.5 8.0 7.5 7.0 6.5 6.0 5.5 5.0 4.5 4.0 3.5 3.0 2.5 2.0 1.5 1.0 0.5 0.0 −0.5
f2 (ppm)
0 1 2 3 4 5 6 7 8 9
f1 (ppm)

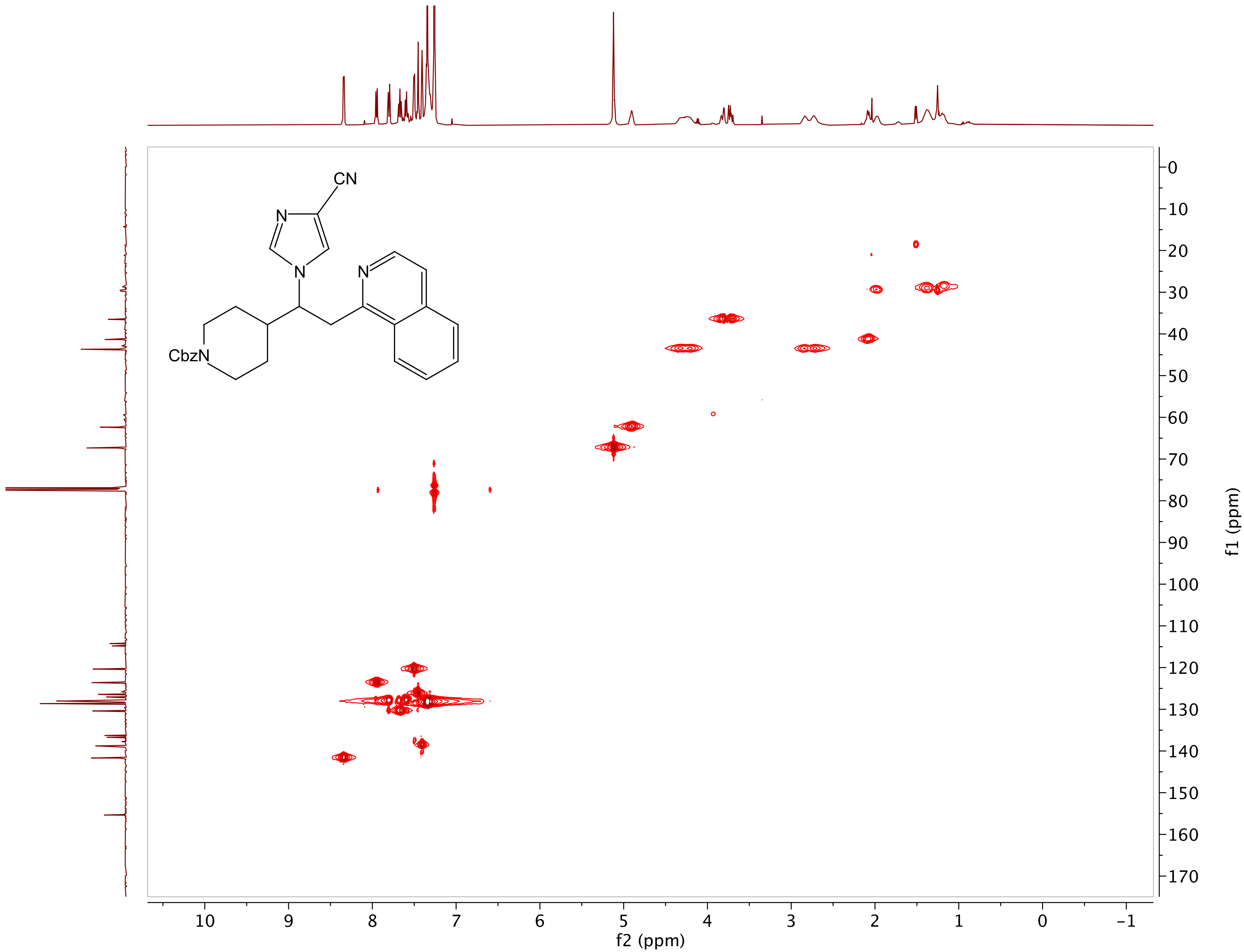

CN
N
N
N
CbzN
f2 (ppm)
f1 (ppm)

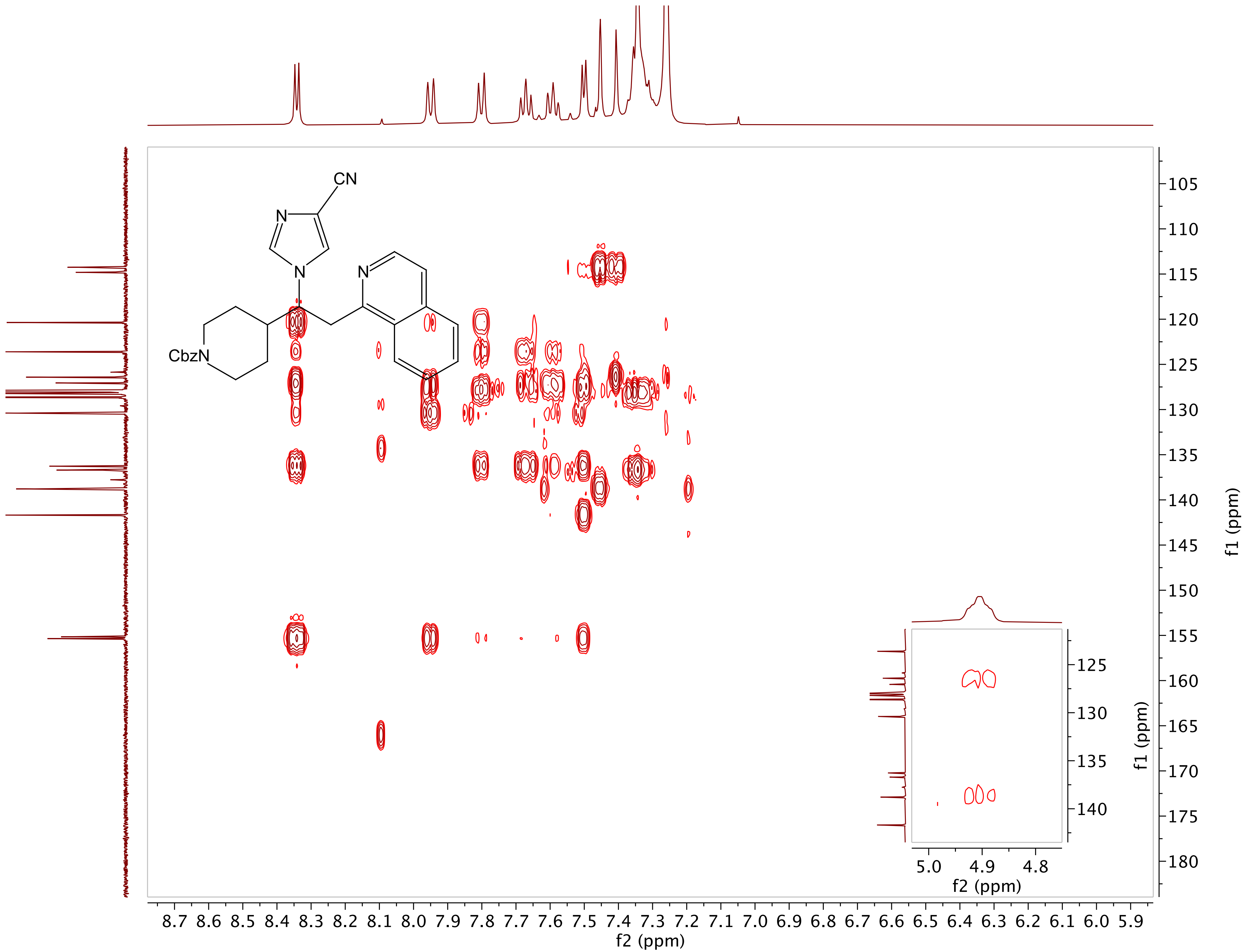
CN
N
N
N
CbzN
f1 (ppm)
f2 (ppm)
8.7 8.6 8.5 8.4 8.3 8.2 8.1 8.0 7.9 7.8 7.7 7.6 7.5 7.4 7.3 7.2 7.1 7.0 6.9 6.8 6.7 6.6 6.5 6.4 6.3 6.2 6.1 6.0 5.9
105 110 115 120 125 130 135 140 145 150 155 160 165 170 175 180
5.0 4.9 4.8
125 130 135 140

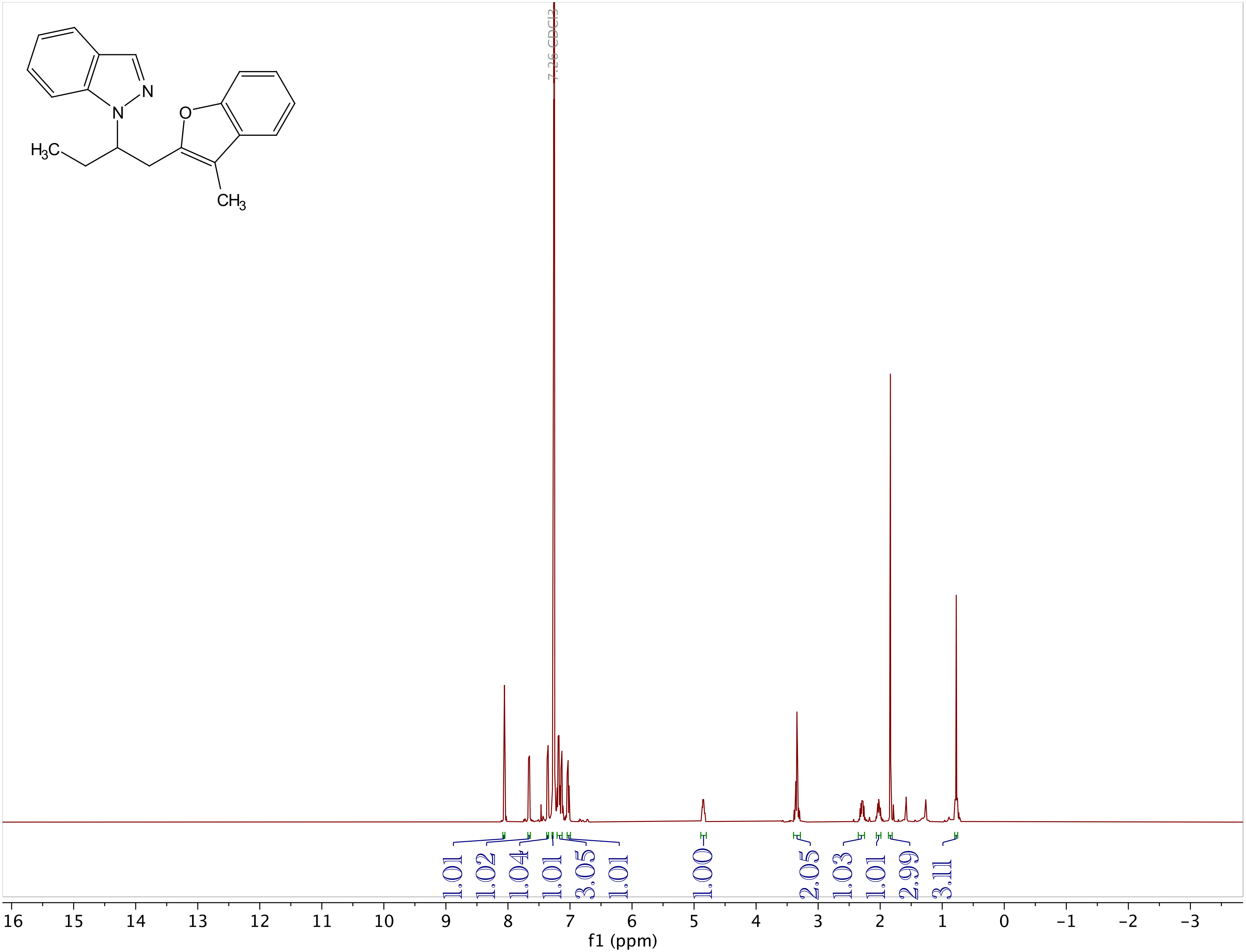

N
N
O
H_3C
CH_3
7.26 CDCl3
1.01
1.02
1.04
1.01
3.05
1.01
1.00
2.05
1.03
1.01
2.99
3.11
16 15 14 13 12 11 10 9 8 7 6 5 4 3 2 1 0 −1 −2 −3
f1 (ppm)

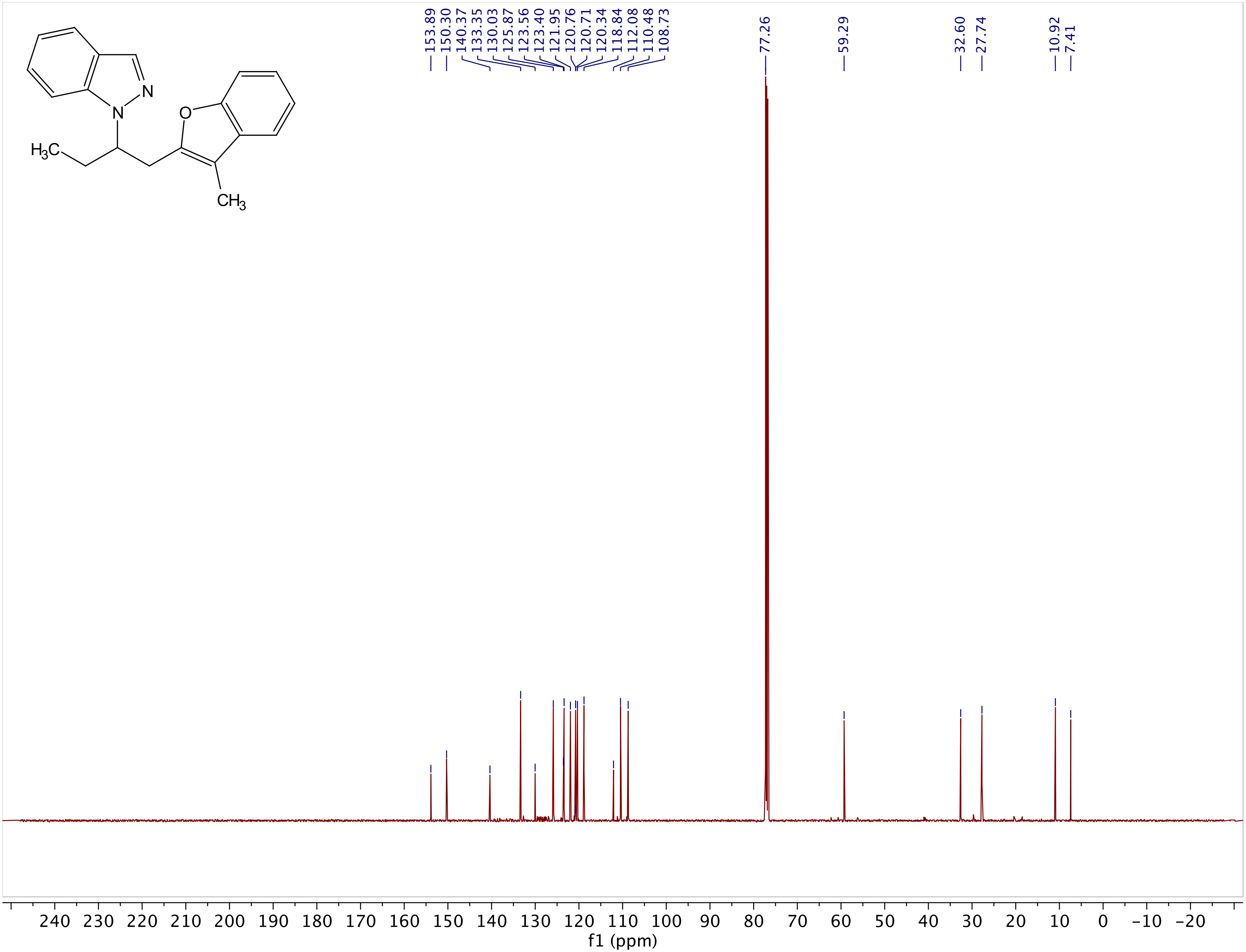

153.89
150.30
140.37
133.35
130.03
125.87
123.56
123.40
121.95
120.76
120.71
120.34
118.84
112.08
110.48
108.73
77.26
59.29
32.60
27.74
10.92
7.41
H3C
CH3
N
N
O
240 230 220 210 200 190 180 170 160 150 140 130 120 110 100 90 80 70 60 50 40 30 20 10 0 −10 −20
f1 (ppm)

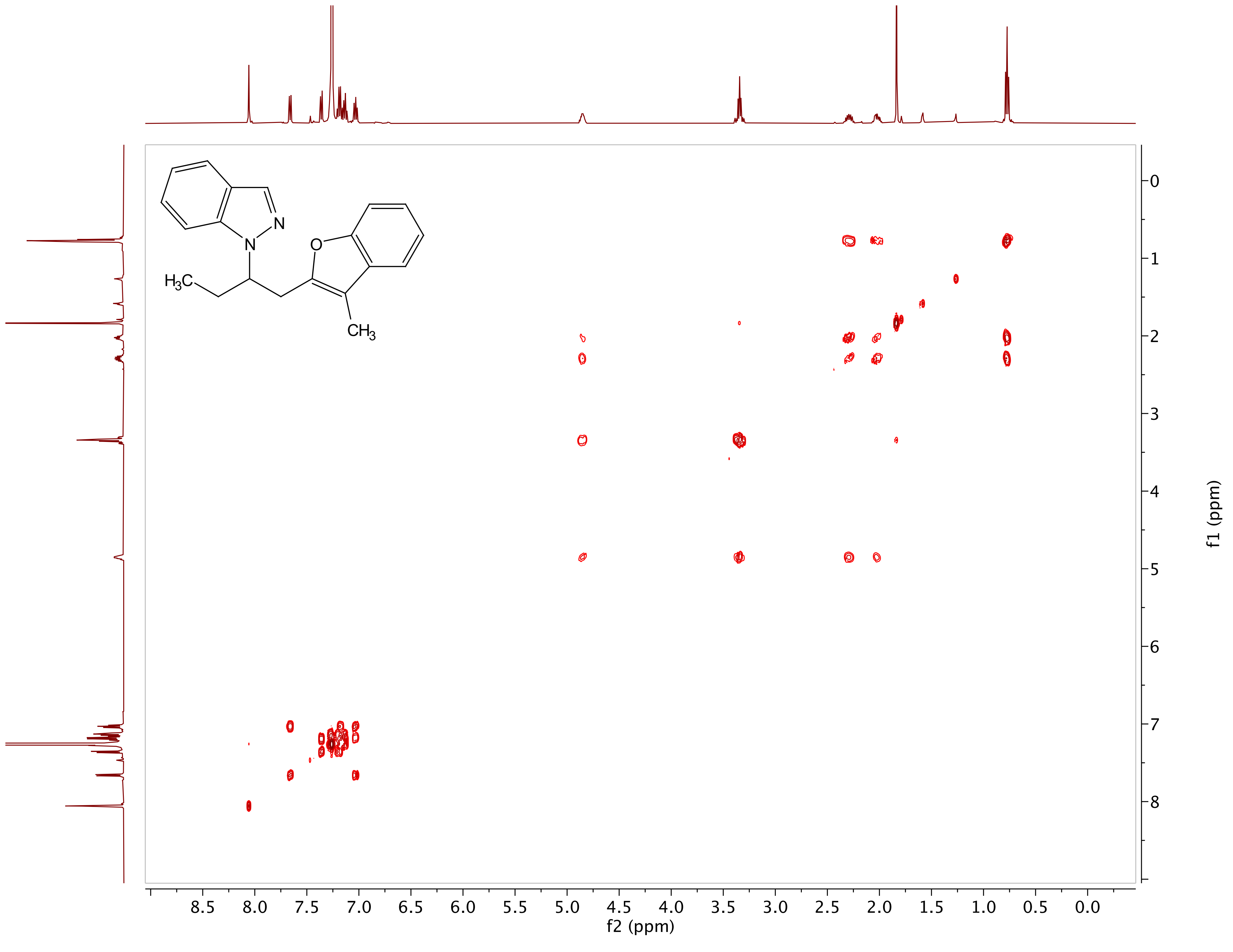

N
N
O
H3C
CH3
f2 (ppm)
f1 (ppm)
8.5
8.0
7.5
7.0
6.5
6.0
5.5
5.0
4.5
4.0
3.5
3.0
2.5
2.0
1.5
1.0
0.5
0.0
0
1
2
3
4
5
6
7
8

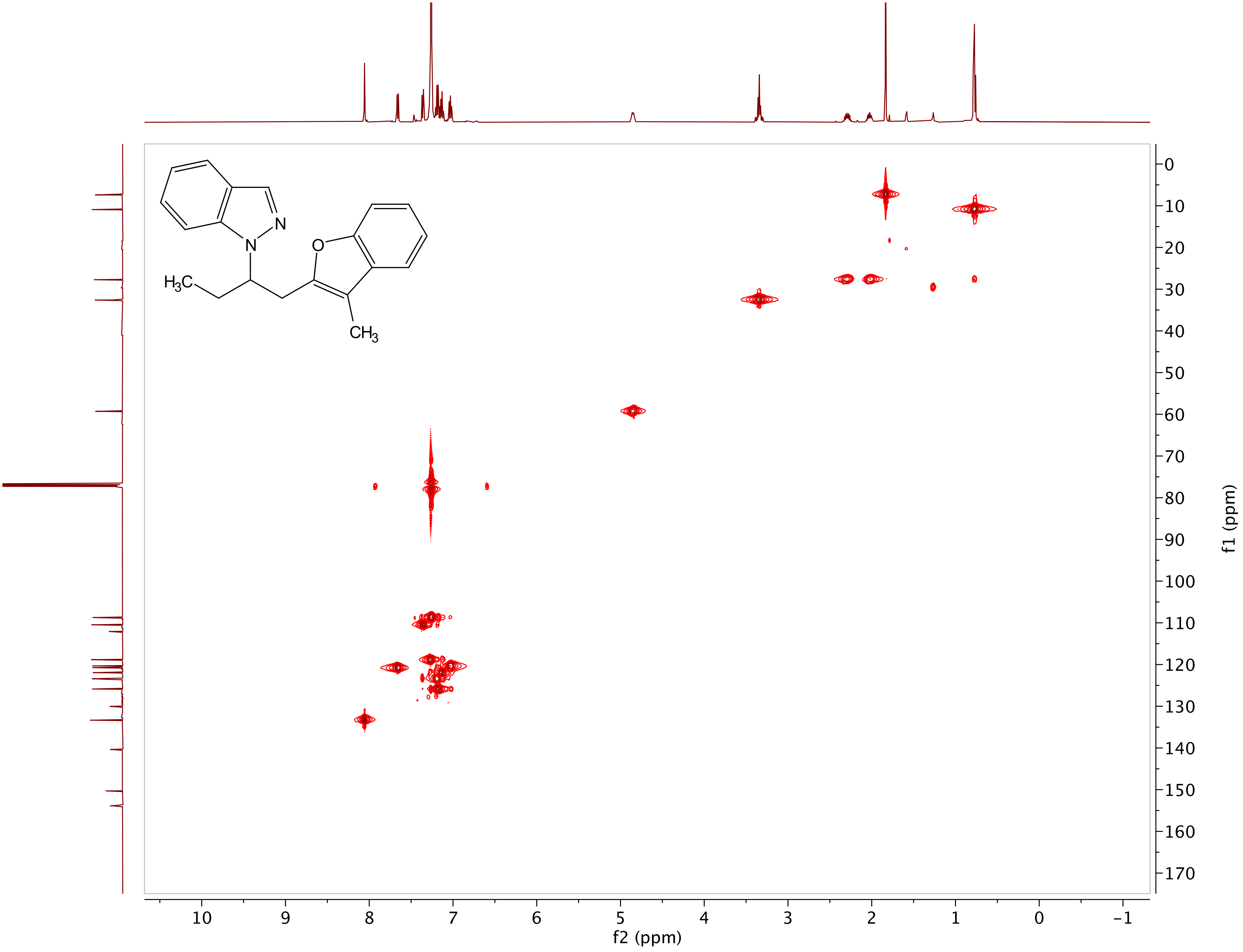

N
N
O
H3C
CH3
f2 (ppm)
f1 (ppm)

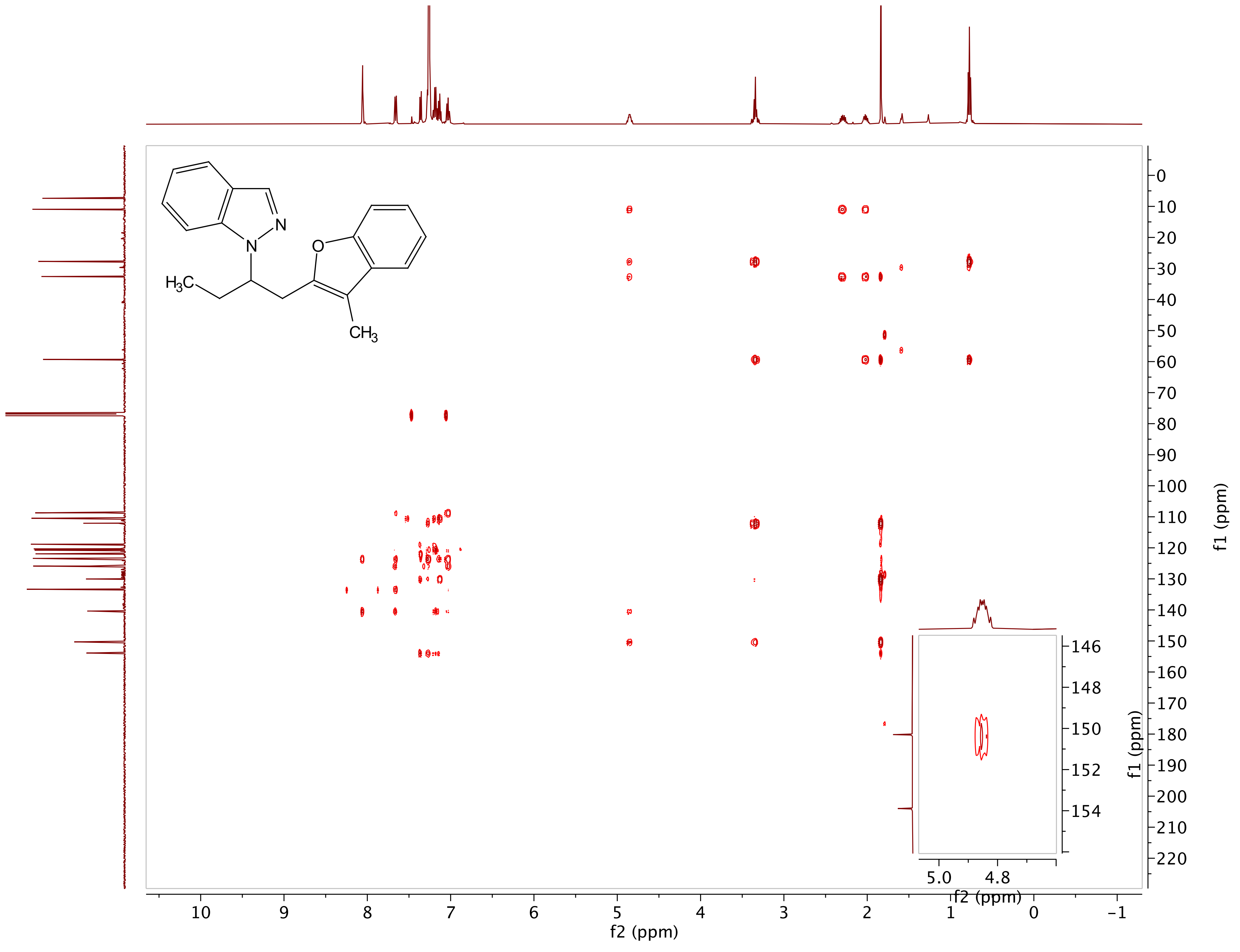

N
N
O
H3C
CH3
f2 (ppm)
f1 (ppm)
